# Network-assisted analysis of primary Sjögren’s syndrome GWAS data in Han Chinese

**DOI:** 10.1038/srep18855

**Published:** 2015-12-21

**Authors:** Kechi Fang, Kunlin Zhang, Jing Wang

**Affiliations:** 1 Key Laboratory of Mental Health, Institute of Psychology, Chinese Academy of Sciences, Beijing 100101, China

## Abstract

Primary Sjögren’s syndrome (pSS) is a complex autoimmune disorder. So far, genetic research in pSS has lagged far behind and the underlying biological mechanism is unclear. Further exploring existing genome-wide association study (GWAS) data is urgently expected to uncover disease-related gene combination patterns. Herein, we conducted a network-based analysis by integrating pSS GWAS in Han Chinese with a protein-protein interactions network to identify pSS candidate genes. After module detection and evaluation, 8 dense modules covering 40 genes were obtained for further functional annotation. Additional 31 MHC genes with significant gene-level P-values (sigMHC-gene) were also remained. The combined module genes and sigMHC-genes, a total of 71 genes, were denoted as pSS candidate genes. Of these pSS candidates, 14 genes had been reported to be associated with any of pSS, RA, and SLE, including *STAT4*, *GTF2I*, *HLA-DPB1*, *HLA-DRB1*, *PTTG1*, *HLA-DQB1*, *MBL2, TAP2, CFLAR*, *NFKBIE*, *HLA-DRA*, *APOM*, *HLA-DQA2* and *NOTCH4.* This is the first report of the network-assisted analysis for pSS GWAS data to explore combined gene patterns associated with pSS. Our study suggests that network-assisted analysis is a useful approach to gaining further insights into the biology of associated genes and providing important clues for future research into pSS etiology.

Sjögren’s syndrome (SS) is a chronic autoimmune disease characterized by exocrine gland dysfunction, specifically the salivary and lacrimal glands, resulting in oral and ocular dryness^1^. The disease may occur alone as primary Sjögren’s syndrome (pSS) or in connection with other systemic rheumatic conditions as secondary Sjögren’s syndrome (sSS)^1^. In China, the prevalence of pSS is estimated to be 0.77%^2^. Although pSS is one of the most common autoimmune diseases, scientific and medical research in pSS has lagged far behind and the pathogenic mechanisms of pSS are not yet fully known^3^. An interaction between genetic predisposition and environmental factors is believed to cause pSS^4^.

In recent years, genome-wide association studies (GWAS) have become a promising approach to unravelling common variants associated with human complex disorders including pSS^5^,^6^. The pSS GWASs have uncovered a few risk loci conferring susceptibility to pSS^5^,^6^. In spite of these successes, as with other complex diseases, GWAS analysis of pSS is limited by the use of a genome-wide significance cutoff SNP P-value of 5 × 10^−8^ needed for multiple testing correction^7^. Except the strongest genetic markers, many modest loci that each contributes in small part to the genetics of the disease may be ignored under this stringent strategy^8^. The reported loci by GWAS account for only a small proportion of pSS genetic risk. The underlying genes remain largely unknown, especially the interactions among these susceptibility genes are elusive. Moreover, how to translate the GWAS observations into any biological function is still a challenge for pSS. Hence there is an urgent need to apply new method that can integrate GWAS data with high-throughput datasets to examine the combined effect of multiple variants for pSS.

As human protein interaction data become more and more abundant, protein-protein interaction (PPI) networks are increasingly serving as tools to discover the molecular basis of diseases. PPI network provides a convenient framework for exploring relationships of disease-related genes and can be integrated with other various biological data. An integrative analysis of GWAS data with PPI network opens a new avenue for promoting the identification of true genetic signals and has been widely applied in many diseases^9^,^10^,^11^. The rationale behind network-assisted analysis is “guilt by association”^12^, *i.e.* different causal genes for the same phenotypes often interact, either directly or via common interaction partners.

Along these lines, the present study applied a network-assisted method by integrating pSS GWAS data in Han Chinese with human PPI network to investigate whether a set of genes, whose protein products closely interact with each other might collectively contribute to pSS risk. We highlighted 71 pSS candidate genes including 40 module genes identified by dense module searching (DMS) algorithm and additional 31 MHC genes with small gene-level P-values (sigMHC-genes). Of these candidates, 14 genes had been reported to be associated with any of pSS, RA, and SLE. The results also obtained gene-gene interactions among these candidates. Our network-assisted analysis of pSS GWAS would facilitate the understanding of genetic mechanism of pSS.

## Results

### Identification of sigMHC-genes and modules enriched for pSS-associated genes

To perform network-assisted analysis, pSS GWAS data in Han Chinese was applied and gene-level P-values were computed with VEGAS (see Methods). A total of 26,929 genes with P-values were obtained. Then, the gene P-values were integrated with a high confident PPI network (see Methods), resulting in a pSS specific node-weighted network of 9,203 proteins and 31,908 interactions. The involved interactions were listed in Supplementary Table S1.

Particularly, there were 31 genes located in MHC region and with gene P-values < 0.05, defined as sigMHC-genes (see Methods). In order to reduce the influence of sigMHC-genes on module searching and primarily focus on genes outside MHC, the sigMHC-genes were not set as seed nodes to search modules.

**Figure 1:**
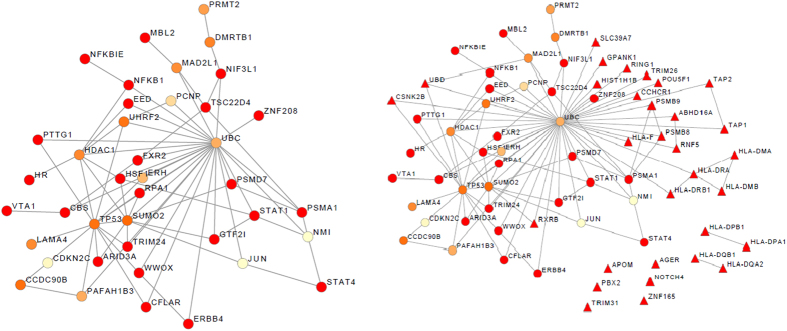
(**a**) The subnetwork formed by identified module genes; (**b**) the subnetwork formed by sigMHC-genes and identified module genes. The triangle-shaped nodes represent sigMHC-genes and circular-shaped nodes represent DMS-identified module genes. The color of the node was proportioned with the gene *P*-value. The most significant gene P-value was red color and the most non-significant gene P-value was yellow color.

Dense module searching was performed within the node-weighted pSS network to identify modules enriched for genes with significant pSS genetic signals. A total of 8,594 independent modules were preliminarily generated. After estimating the significance of module scores, 127 modules met the criterion of 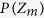 < 0.05 were left for further estimating the topological properties. As a result, eight modules were finally remained as significant modules. The union of resultant modules was finally computed, resulting in a single connected subnetwork of 40 non-redundant genes and 70 interactions (Fig. 1a). Of these 40 module genes, 24 had nominally significant gene P-values (<0.05). To further validate whether module genes were significantly physically interacted, DAPPLE was used^13^. The results showed that the direct PPI network of module genes had more significant edges than expected by chance (permutation P-value = 9.9 × 10^−5^) (Supplementary Figure S1), suggesting that the interactions among the module genes were statistically significantly connected. The detailed information for the DMS-identified module genes and sigMHC-genes were listed in Supplementary Table S2.

### Biological annotation for the identified module genes

To better understand the biological functions of the DMS-identified module genes, we conducted a Gene Ontology (GO) enrichment analysis by using DAVID. The enriched biological processes were related with negative regulation of protein metabolic process and proteasomal protein catabolic process. The enriched molecular functions were about transcription regulator activity and transcription factor activity. Further information of GO enrichment analysis of module genes was summarized in Supplementary Table S3.

We also computed the tissue specificity of module genes by using the Gene Enrichment Profiler. In the transcript expression heatmap (Supplementary Figure S2), approximately two-thirds of these genes were highly expressed in immune-related cell types (specifically, B, T and myeloid cells). In the transcript enrichment heatmap (Supplementary Figure S3), we found module genes were preferentially expressed in the immune cell types.

### Module genes and sigMHC-genes as candidates for pSS

To explore whether exist interactions between the DMS-identified module genes and sigMHC-genes, we extracted these genes and according interactions from the node-weighted pSS network, resulting in a subnetwork as shown in Fig. 1b. Most of sigMHC-genes directly or indirectly connected with module genes except 6 singletons and two isolated PPI pairs (*HLA-DPB1* vs. *HLA-DPA1*, and *HLA-DQB1* vs. *HLA-DQA2*). The combined module genes and sigMHC-genes were defined as candidate genes (71 genes) for pSS. Of these candidates, four genes (*STAT4*, *GTF2I*, *HLA-DPB1*, and *HLA-DRB1*) had been reported their association with pSS in the original GWAS dataset^6^, and gene *PTTG1* (pituitary tumor-transforming 1) had been reported as suggestive association (rs2431098, allelic meta P-value = 2.28 × 10^−7^; rs2431697, allelic meta P-value = 3.76 × 10^−6^) with SS in another GWAS study of SS in European descent^5^. In addition, three genes (*HLA-DQB1*^14^, *MBL2*^15^, and *TAP2*^16^) had been previously reported their association with SS/pSS by candidate gene studies.

**Figure 2:**
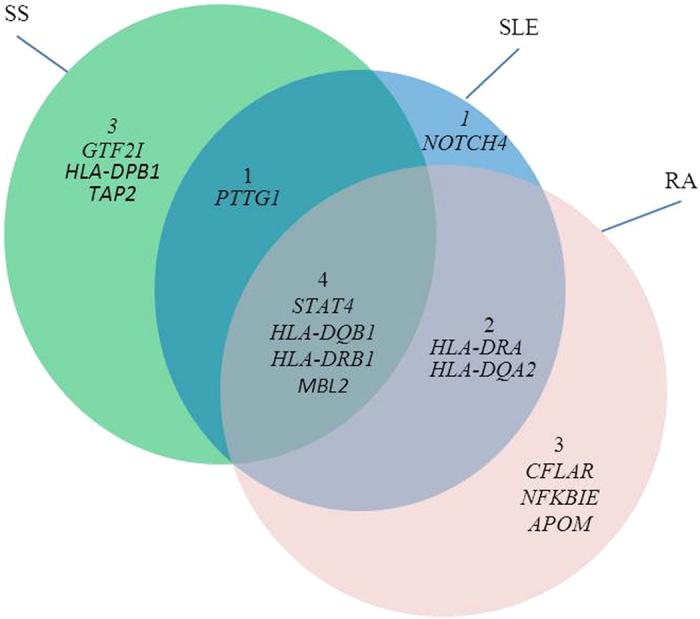
The overlaps of candidate pSS genes among other autoimmune diseases. The green, blue, and pink circles indicate the candidate pSS genes that have positive evidence to be associated with SS, SLE, and RA, respectively.

Given the overlap of certain clinical and serologic features between pSS and other autoimmune diseases (AIDs), such as Rheumatoid arthritis (RA) and Systemic lupus erythematosus (SLE), it is reasonably assumed that pSS might share some genetic signatures with other AIDs^17^. Hence, we also investigated how many candidate pSS genes had been reported their susceptibility to either SLE or RA by searching GWAS studies collected in GWAS Catalog^8^. The overlaps of candidate pSS genes among SLE and RA was shown in Fig. 2. Another six genes with positive evidence associated with SLE or RA were obtained, including five genes (*HLA-DRA*^18^, *HLA-DQA2*^19^, *CFLAR*^20^, *NFKBIE*^20^,^21^,^22^, and *APOM*^23^) reported to be associated with RA, and two genes (*HLA-DQA2*^24^,^25^ and *NOTCH4*^24^) reported to be associated with SLE. Gene *HLA-DQA2* was overlapped between RA and SLE. These six SLE and RA susceptibility genes might be the shared genetic signatures between AIDs. In addition, four genes (*STAT4*^6^,^20^,^22^,^24^,^25^,^26^,^27^, *HLA-DQB1*^14^,^19^,^24^,^27^,^28^,^29^, *HLA-DRB1*^6^,^20^,^26^,^27^, and *MBL2*^15^,^30^,^31^) were overlapped among SS, SLE and RA, and gene *PTTG1* was overlapped between SS and SLE^5^,^24^.

## Discussion

Unlike the extensive GWAS experiment in other AIDs, such as RA and SLE, there have been only two GWAS studies in SS/pSS until now^5^,^6^. Genetic studies of pSS have lagged behind. To further mining the existing genetic data, network-assisted analysis of pSS GWAS in Han Chinese was performed in order to explore the joint effects of multiple genetic association signals on pSS and discover additional candidate genes associated with pSS pathogenesis. First, all SNPs were first mapped into genes and gene-based association was performed by using VEGAS. Then, dense modules were dynamically searched in the context of the node-weighted pSS network via DMS, yielding thousands of functional modules. To avoid false positive results and the topology bias, a strict criterion was applied to select modules with significant genetic signals. After two stepwise significance tests (see Methods), 8 modules covering 40 genes were screened out. These 40 module genes had a high proportion of significant genes (60%) and preferentially expressed in immune-related cell types. The proteins encoded by the DMS-identified module genes were more closely interconnected than what would be expected by random cases, as suggested by DAPPLE analysis. In addition, there were 31 MHC genes with significant gene P-values, defined as sigMHC-genes. To avoid the results of module search focusing on MHC region, the sigMHC-genes were not set as “seed genes” (see methods) and directly remained as part of final results. By merging all module genes and sigMHC-genes, a total of 71 genes were involved and denoted as candidate pSS genes.

Of the 71 candidate pSS genes, eight genes had been previously reported their association with SS/pSS, as well as another six genes that had positive evidence associated with SLE or RA (Fig. 2). All the 14 reported genes had significant gene P-values (see Supplementary Table S2). Considering that pSS might share genetic signatures with SLE and RA to some extent, these six SLE or RA susceptibility genes were very likely associated with pSS. For example, one of reported RA susceptibility genes was *NFKBIE* (nuclear factor of kappa light polypeptide gene enhancer in B-cells inhibitor, epsilon)^20^,^21^,^22^, which is a non-MHC gene with gene-level P-value of 0.00801. *NFKBIE*, also known as *IκBε*, is part of the IκB family of proteins that regulates NF-κB-dependent transcription by inhibiting DNA binding and localizing these factors to the cell cytoplasm^32^. It has been demonstrated that *IκBα* (another IκB family of protein) promoter polymorphisms are associated with susceptibility to SS^33^. In addition, NF-κB plays an important role in inflammatory diseases and in the development of autoimmunity^34^. Experimental studies have shown an activation of NF-κB in pSS. These clues implied that *NFKBIE* might be also associated with pSS.

Due to disease genes execute their functions are not alone, the interactions among the candidate pSS genes might play an important role. It has been observed that causal genes for the same Mendelian disease often physically interact^35^,^36^. There were two direct interactions among the reported genes, *i.e.* (*HLA-DRB1* vs. *HLA-DRA*), and (*HLA-DQB1* vs. *HLA-DQA2*). It was worth noting that most of 14 reported genes were closely connected by gene *UBC* (ubiquitin C) except for some sigMHC-genes. As the theory of “guilt by association”, it is possible that a few highly connected nodes (hub genes) bring together several disease-associated genes, even though the hubs themselves are not relevant^37^. In this study, although the gene-level P-value of *UBC* was not significant (gene P-value = 0.463), it was involved by all the DMS-identified modules. As shown in Fig. 1a,b, *UBC* was centered both in the subnetwork formed by DMS-identified genes and in the subnetwork formed by the combined candidate genes. *UBC* directly or indirectly (via one or two nodes) connected with most of pSS candidate genes.

Of the rest of 57 genes, some genes with non-significant gene P-values might act as connectors to connect with other disease genes. For example, the gene P-value of *JUN* (jun proto-oncogene) was not significant (gene P-value = 0.918), but it interacted with *STAT4* (signal transducer and activator of transcription 4), which was the most significant gene in this study (gene P-value = 1E-07). In addition, *STAT4* was the only gene that had been confirmed its association with SS/pSS in both Han Chinese and European descent^5^,^6^. In addition, other novel candidate pSS genes with significant gene P-values were also valuable, such as *STAT1* (signal transducer and activator of transcription 1, 91kDa). *STAT1* (gene P-value = 0.00267) directly interacted with *GTF2I*, which was a reported pSS associated gene in the Chinese cohort^6^. *STAT1* phosphorylation at serine 708 is a key event in the interferon signalling pathway^38^. In many SS patient, interferon activation plays an important role in the immune attack and destruction of salivary and lacrimal glands at some stage in the course of the disease. These evidence suggested that *STAT1* was likely related with pSS.

The present study has some limitations that require consideration. First, only one pSS GWAS data in Han Chinese was available for this study. It would be more valuable if we could make a comparison between multiple GWAS datasets from different population. In our study, only gene *PTTG1* could be cross evaluated between two SS/pSS GWAS. *PTTG1* was only reported as suggestive association with SS in European descent^5^ and not found to be a risk factor in the Chinese cohort^6^. However, this gene was significant at gene-level (gene P-value = 0.00143) and identified by module search, and it was also reported to be associated with SLE^24^. These lines of evidence implied that *PTTG1* might be associated with pSS in Han Chinese. Second, calculation of gene-level P-value is a key step in the network-assisted analysis of GWAS. VEGAS is comparable with other tools to compute gene P-values, however, it could only deal with autosomal SNPs. Due to women are nine times more likely than men to be affected with SS, it would be interesting to evaluate SNPs located on the sex chromosomes (X and Y).

In summary, this is the first use of network analysis of pSS GWAS data to further mine genetic signals at a molecular level instead of analyzing each of single locus (SNP). Complementary to the traditional GWAS analysis, it was more powerful that gene-level P-value was considered by calculating the combined effect of all SNPs within a gene and subsequently integrated with a pSS-specific PPI network to search for gene combination patterns contributed to pSS. Our findings included 40 non-MHC genes identified by DMS algorithm and 31MHC-region genes with significant gene-level P-values. These candidates and interactions among them were more likely to be associated with pSS.

Deciphering the mechanism of pSS pathogenesis is still challenging, although certain progress has been made, much remains to be understood. Deriving a pSS-specific PPI network and identification of dense module genes and sigMHC-genes, as described herein, offers new targets for further functional assessment for this chronic and complex condition.

## Methods

The workflow of the network-assisted analysis for pSS GWAS data was shown in Supplementary Figure S4, and the sections below were labeled in correspondence with this figure.

### pSS GWAS dataset

The pSS GWAS data is composed of samples of Han Chinese^6^. There are 642,832 SNPs in 597 pSS cases and 1,090 controls genotyped with the Affymetrix Axiom Genome-Wide CHB 1 Array Plate. Details of this pSS GWAS data and process of quality control are provided in ref.^6^. After quality control filtering, a total of 542 cases, 1,050 controls and 556,134 autosomal SNPs were remained for subsequent analysis.

### Computing gene-level P-values

To perform network analysis and examine functional correlation between genes, gene-level P-value representing the significance of association with phenotype for each gene was needed to be considered. The gene-level P-value was calculated with VEGAS^39^, which can incorporate information from all SNPs mapped to a gene and take into account the linkage disequilibrium (LD) patterns between SNPs for the specific samples (a custom set of individuals) or ethnic background (HapMap data). In VEGAS, all SNPs were mapped to human protein-coding genes according to positions on the UCSC Genome Browser. In this study, an off-line version of VEGAS was applied and improved in some aspects. First, gene position was updated from original hg18 to hg19 downloaded from Ensembl (GRCh37.P11)^40^. Second, in order to capture SNPs in regulatory region and simultaneously avoid too many genes with overlapped SNPs, gene boundary was extended to 20kb upstream/downstream of the gene coordinates instead of 50kb by default. Third, when estimating the LD patterns, the pSS GWAS data was used.

### Building a node-weighted pSS interactome

A consolidated human protein-protein interaction (PPI) network data was obtained from iRefIndex database (version 13.0)^41^, which collected nine interaction databases and computed the union of data sets. Among this large network, many interactions were either predicted or supported by a single experiment. In order to reduce the rate of false positives, we included only those interactions supported by at least two publications for all subsequent analyses, resulting in a highly reliable network of 10,163 nodes (genes) and 36,680 interactions. Then, the nodes involved in this network was annotated with gene P-value as a node attribute, and extracted to derive a node-weighted pSS network.

### sigMHC-genes

Currently, the reported susceptibility genes associated with pSS mainly focused on immune-related genes and the MHC region^5^,^6^,^42^,^43^. To unravel more risk genes outside MHC region and avoid the complexity of the MHC region^44^, we did not assign gene P-values for nodes in pSS network if the corresponding genes located in MHC region and their gene P-values <0.05, named as significant MHC genes (sigMHC-genes). Since sigMHC-genes might interplay with other significant genes and play an important role in the disease-related biological functions, these genes were still left in the pSS network to maintain the integrity of the network.

### Module detection

A dense module search (DMS) method implemented in dmGWAS was applied to search for modules that were enriched with significant P-value genes in the context of the node-weighted pSS network^45^. DMS starts by transferring each gene P-value into a Z score (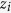) by using the inverse normal cumulative distribution function^46^. For a module with 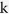 genes, the module score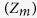 was computed by summing the 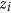 over all genes in the module, *i.e.* 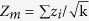. The detailed process of module search can be found in this study^45^. Briefly, for a given “seed gene”, module grows by adding the neighboring nodes that can generate the maximum increment of a module score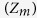. Module growth will stop if the increment is not greater than 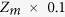. The process of module searching was conducted taking each node in the pSS interactome as the seed gene except for sigMHC-genes.

### Module evaluation

For the proper capture of the connection between genetic association and network topology, two steps of tests were performed.

First, to assess the significance of the resultant modules, module P-values were calculated based on the module scores by empirically estimating the null distribution, which is assumed to be a normal distribution^47^. Specifically, module scores were median-centered, and then the parameters of mean 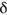 and standard deviation 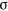 were estimated for the empirical null distribution using the R package *locfdr*. The standardized module scores 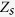 were computed and converted to P-values by using the normal cumulative density function. The modules with P-values 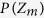 < 0.05 were selected for the further estimation.

Second, to avoid the bias that nodes with many interactors in the PPI are more probably to be chose by DMS, the topology of resultant modules were evaluated as suggested in^48^. All nodes in the network were divided into four groups according to nodes degree, *i.e.* 0–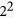, 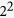
–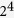, 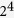
–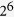, and >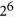. For a given module with k genes, 10,000 modules with the same number of genes were generated by considering which group each gene located in and then randomly picking one gene from the corresponding group (*i.e.* structurally equivalent random networks). An empirical P-value was calculated by 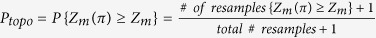, where 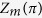 is the score of the random module for the 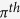 resample. The modules with P-values 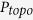 < 0.05 were selected as the final results.

### Assessment of the significance of connectivity between module genes

To evaluate whether module genes were densely connected via PPI network, a permutation test was performed to assess the significance of connectivity between module genes by using DAPPLE (Disease Association Protein-Protein Link Evaluator) algorithm^13^. Briefly, this approach first generated a random network that has nearly the exact same structure as the original one that is derived from the InWeb database^36^. The node labels (*i.e.* the protein names) were then randomly re-assigned to nodes of equal binding degree. DAPPLE assumes a null distribution of connectivity that is entirely a function of the binding degree of individual proteins. We built 10,000 different random networks and each of them had the same number of proteins, connectivity and per-protein binding degree as InWeb. The significance of our real PPI network formed by module genes was then assessed through permutation. For more details, please refer to the article^13^.

### GO enrichment and cell-specific expression of module genes

In order to discern biological attributes of the identified module genes, we performed Gene Ontology (GO) enrichment analysis by using DAVID^49^. DAVID bioinformatics resources consist of an integrated biological knowledgebase and analytic tools aimed at systematically extracting biologically meaning from a list of genes or proteins. Fisher Exact tests were conducted in DAVID to compute the P-value for each GO term. In this case study, only GO terms with an adjusted P-value (Benjamini & Hochberg) of less than 0.25 were selected.

Cell-specific expression was assessed with an online tool Gene Enrichment Profiler^50^. This tool computes the expression and enrichment of any set of query genes on the basis of a reference set obtained from 126 normal tissues and cell types (represented by 557 microarrays).

## Additional Information

**How to cite this article**: Fang, K. *et al.* Network-assisted analysis of primary Sjögren’s syndrome GWAS data in Han Chinese. *Sci. Rep.* **5**, 18855; doi: 10.1038/srep18855 (2015).

## Supplementary Material

Supplementary Information
