# Supplementary material for: Network-assisted analysis of primary Sjögren’s syndrome GWAS data in Han Chinese
Source: Sci Rep. 2015 Dec 21;5:18855. doi: 10.1038/srep18855 (PMC4685393; doi:10.1038/srep18855)
Supplement: Supplementary Information [file srep18855-s1.pdf]

Network-assisted analysis of primary Sjögren's syndrome GWAS data in Han Chinese

Kechi Fang, Kunlin Zhang, Jing Wang*

Address: Key Laboratory of Mental Health, Institute of Psychology, Chinese Academy of Sciences, Beijing 100101, China.

Email: Kechi Fang – fangkc@psych.ac.cn; Kunlin Zhang – zhangkl@psych.ac.cn; Jing Wang – wangjing@psych.ac.cn

*Corresponding author

Supplementary materials

Page 3 – Page 5: **Supplementary Figure S1.** The direct network formed by the module genes from DAPPLE.

Page 6: **Supplementary Figure S2**. Transcript expression heatmap.

Page 7: **Supplementary Figure S3**. Transcript enrichment heatmap.

Page 8: **Supplementary Figure S4**. Workflow of network-assisted analysis of pSS GWAS data to identify candidate genes**.**

Page 9 – Page 734: **Supplementary Table S1**. A full list of PPI pairs involved in the node-weighted pSS interactome.

Page 735 – Page 737: **Supplementary Table S2**. Detailed information about module genes and sigMHC-genes.

Page 738: **Supplementary Table S3**. GO terms enriched by module genes.

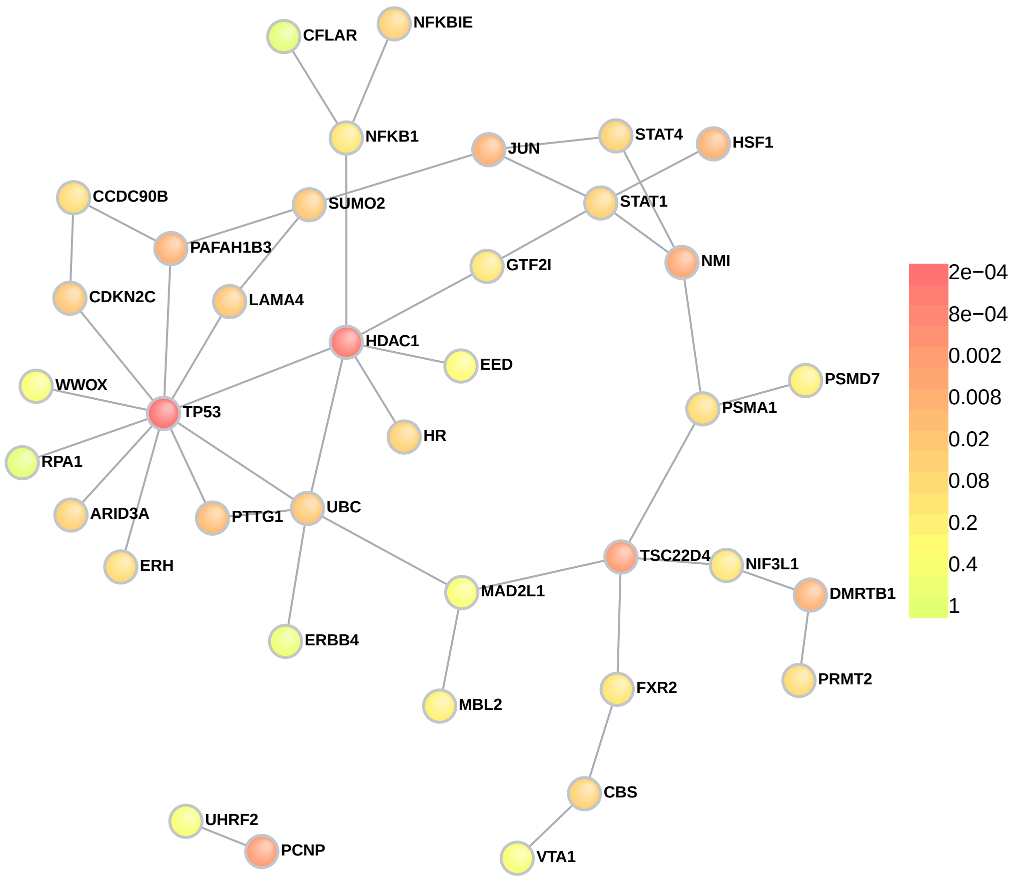
CFLAR
NFKBIE
NFKB1
JUN
STAT4
HSF1
CCDC90B
SUMO2
STAT1
PAFAH1B3
GTF2I
NMI
CDKN2C
LAMA4
HDAC1
EED
WWOX
TP53
PSMD7
PSMA1
HR
RPA1
ARID3A
PTTG1
UBC
TSC22D4
NIF3L1
ERH
MAD2L1
DMRTB1
ERBB4
MBL2
FXR2
PRMT2
CBS
UHRF2
PCNP
VTA1
2e−04
8e−04
0.002
0.008
0.02
0.08
0.2
0.4
1

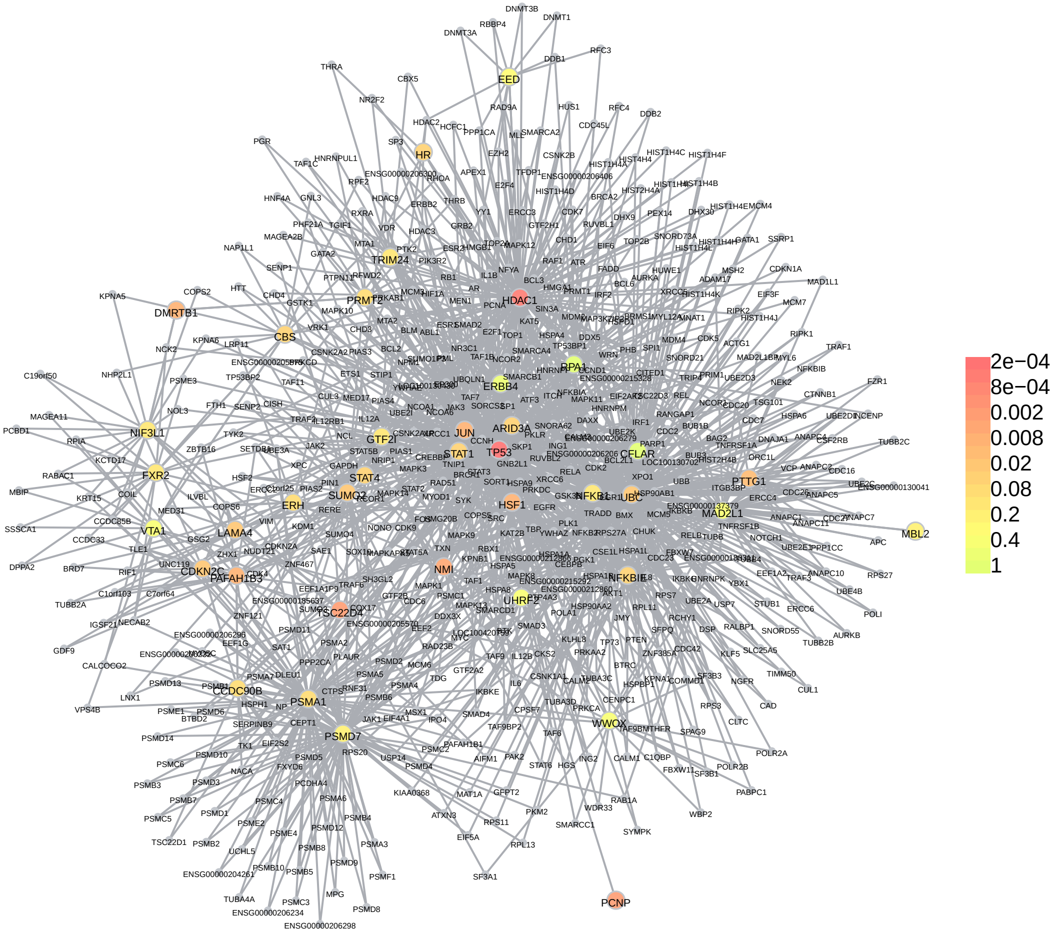
2e−04
8e−04
0.002
0.008
0.02
0.08
0.2
0.4
1
HDAC1
TP53
EED
HR
TRIM24
PRMT2
DMRTB1
CBS
NIF3L1
FXR2
VTA1
LAMA4
CDKN2C
PAFAH1B3
ERH
SUMO2
STAT4
GTF2I
JUN
STAT1
ARID3A
ERBB4
RPA1
CFLAR
HSF1
NFKB1
UBC
PTTG1
MAD2L1
MBL2
NMI
NFKBIE
UHRF2
TSC22D4
CCDC90B
PSMA1
PSMD7
WWOX
PCNP

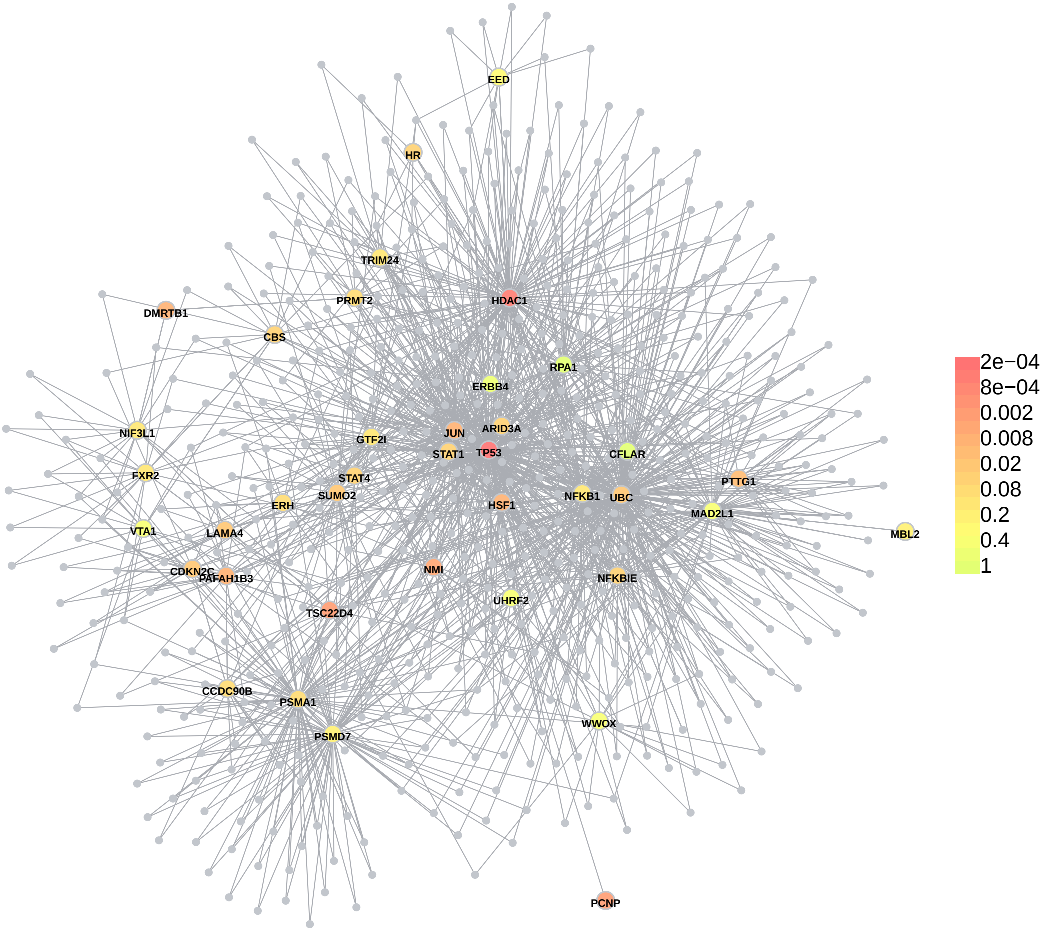
EED
HR
TRIM24
PRMT2
HDAC1
DMRTB1
CBS
RPA1
ERBB4
NIF3L1
GTF2I
JUN
ARID3A
STAT1
TP53
CFLAR
FXR2
STAT4
PTTG1
SUMO2
ERH
HSF1
NFKB1
UBC
MAD2L1
VTA1
LAMA4
MBL2
CDKN2C
PAFAH1B3
NMI
NFKBIE
UHRF2
TSC22D4
CCDC90B
PSMA1
PSMD7
WWOX
PCNP
2e−04
8e−04
0.002
0.008
0.02
0.08
0.2
0.4
1

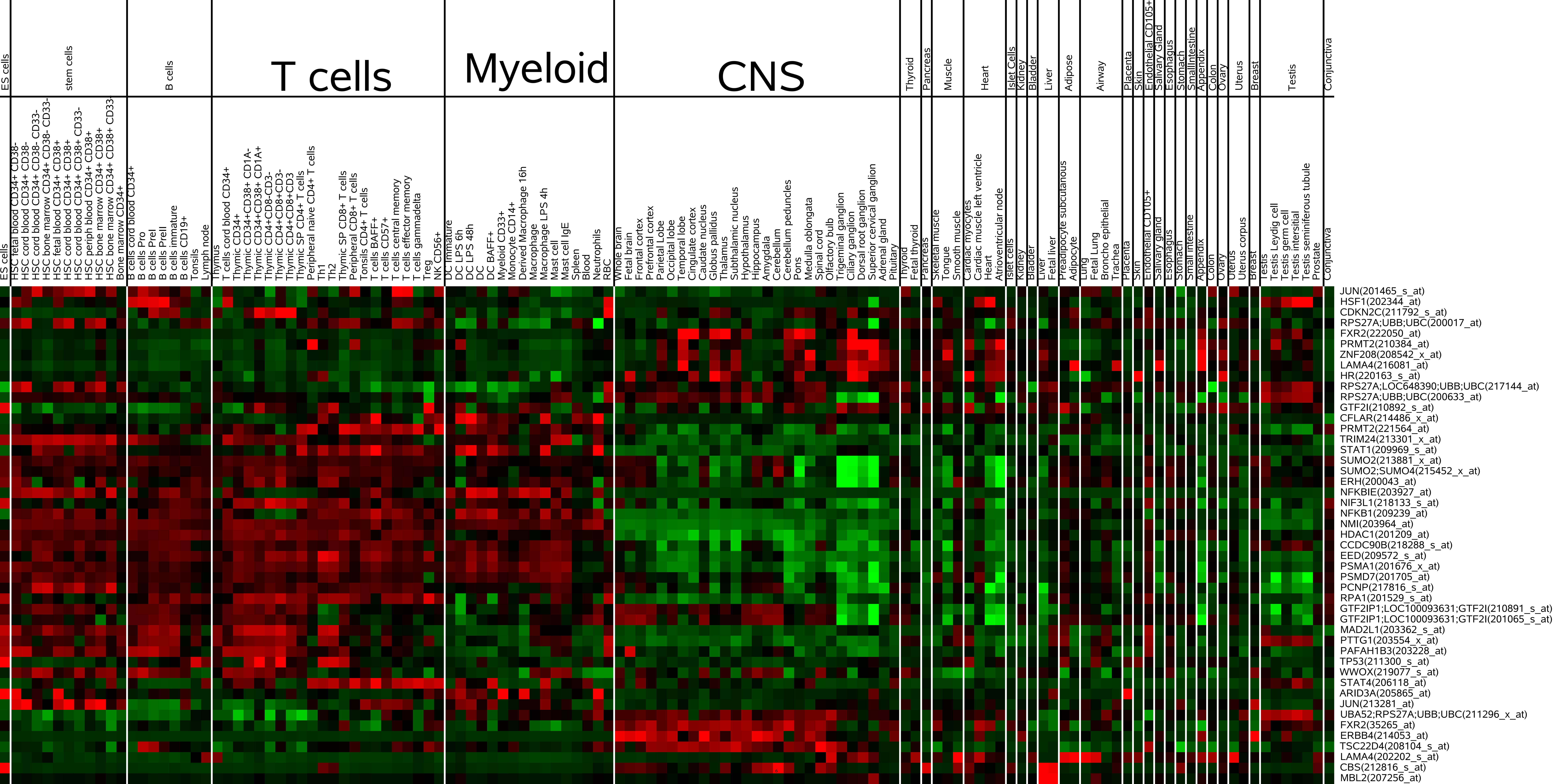

ES cells
stem cells
B cells
T cells
Myeloid
CNS
Thyroid
Pancreas
Muscle
Heart
Islet Cells
Kidney
Bladder
Liver
Adipose
Airway
Placenta
Skin
Endothelial CD105+
Salivary Gland
Esophagus
Stomach
SmallIntestine
Appendix
Colon
Ovary
Uterus
Breast
Testis
Conjunctiva
ES cells
HSC fetal blood CD34+ CD38-
HSC cord blood CD34+ CD38-
HSC cord blood CD34+ CD38- CD33-
HSC bone marrow CD34+ CD38- CD33-
HSC fetal blood CD34+ CD38+
HSC cord blood CD34+ CD38+
HSC cord blood CD34+ CD38+ CD33-
HSC periph blood CD34+ CD38+
HSC bone marrow CD34+ CD38+
HSC bone marrow CD34+ CD38+ CD33
Bone marrow CD34+
B cells cord blood CD34+
B cells Pro
B cells PreI
B cells PreII
B cells immature
B cells CD19+
Tonsils
Lymph node
Thymus
T cells cord blood CD34+
Thymic CD34+
Thymic CD34+CD38+ CD1A-
Thymic CD34+CD38+ CD1A+
Thymic CD4+CD8-CD3-
Thymic CD4+CD8+CD3-
Thymic CD4+CD8+CD3
Thymic SP CD4+ T cells
Peripheral naive CD4+ T cells
Th1
Th2
Thymic SP CD8+ T cells
Peripheral CD8+ T cells
Tonsils CD4+ T cells
T cells BAFF+
T cells CD57+
T cells central memory
T cells effector memory
T cells gammadelta
Treg
NK CD56+
DC immature
DC LPS 6h
DC LPS 48h
DC
DC BAFF+
Myeloid CD33+
Monocyte CD14+
Derived Macrophage 16h
Macrophage
Macrophage LPS 4h
Mast cell
Mast cell IgE
Spleen
Blood
Neutrophils
RBC
Whole brain
Fetal brain
Frontal cortex
Prefrontal cortex
Parietal Lobe
Occipital lobe
Temporal lobe
Cingulate cortex
Caudate nucleus
Globus pallidus
Thalamus
Subthalamic nucleus
Hypothalamus
Hippocampus
Amygdala
Cerebellum
Cerebellum peduncles
Pons
Medulla oblongata
Spinal cord
Olfactory bulb
Trigeminal ganglion
Ciliary ganglion
Dorsal root ganglion
Superior cervical ganglion
Adrenal gland
Pituitary
Thyroid
Fetal thyroid
Pancreas
Skeletal muscle
Tongue
Smooth muscle
Cardiac myocytes
Cardiac muscle left ventricle
Heart
Atrioventricular node
Islet cells
Kidney
Bladder
Liver
Fetal liver
Preadipocyte subcutanous
Adipocyte
Lung
Fetal Lung
Broncho epithelial
Trachea
Placenta
Skin
Endothelial CD105+
Salivary gland
Esophagus
Stomach
Small intestine
Appendix
Colon
Ovary
Uterus
Uterus corpus
Breast
Testis
Testis Leydig cell
Testis germ cell
Testis intersitial
Testis seminiferous tubule
Prostate
Conjunctiva
JUN(201465_s_at)
HSF1(202344_at)
CDKN2C(211792_s_at)
RPS27A;UBB;UBC(200017_at)
FXR2(222050_at)
PRMT2(210384_at)
ZNF208(208542_x_at)
LAMA4(216081_at)
HR(220163_s_at)
RPS27A;LOC648390;UBB;UBC(217144_at)
RPS27A;UBB;UBC(200633_at)
GTF2I(210892_s_at)
CFLAR(214486_x_at)
PRMT2(221564_at)
TRIM24(213301_x_at)
STAT1(209969_s_at)
SUMO2(213881_x_at)
SUMO2;SUMO4(215452_x_at)
ERH(200043_at)
NFKBIE(203927_at)
NIF3L1(218133_s_at)
NFKB1(209239_at)
NMI(203964_at)
HDAC1(201209_at)
CCDC90B(218288_s_at)
EED(209572_s_at)
PSMA1(201676_x_at)
PSMD7(201705_at)
PCNP(217816_s_at)
RPA1(201529_s_at)
GTF2IP1;LOC100093631;GTF2I(210891_s_at)
GTF2IP1;LOC100093631;GTF2I(201065_s_at)
MAD2L1(203362_s_at)
PTTG1(203554_x_at)
PAFAH1B3(203228_at)
TP53(211300_s_at)
WWOX(219077_s_at)
STAT4(206118_at)
ARID3A(205865_at)
JUN(213281_at)
UBA52;RPS27A;UBB;UBC(211296_x_at)
FXR2(35265_at)
ERBB4(214053_at)
TSC22D4(208104_s_at)
LAMA4(202202_s_at)
CBS(212816_s_at)
MBL2(207256_at)

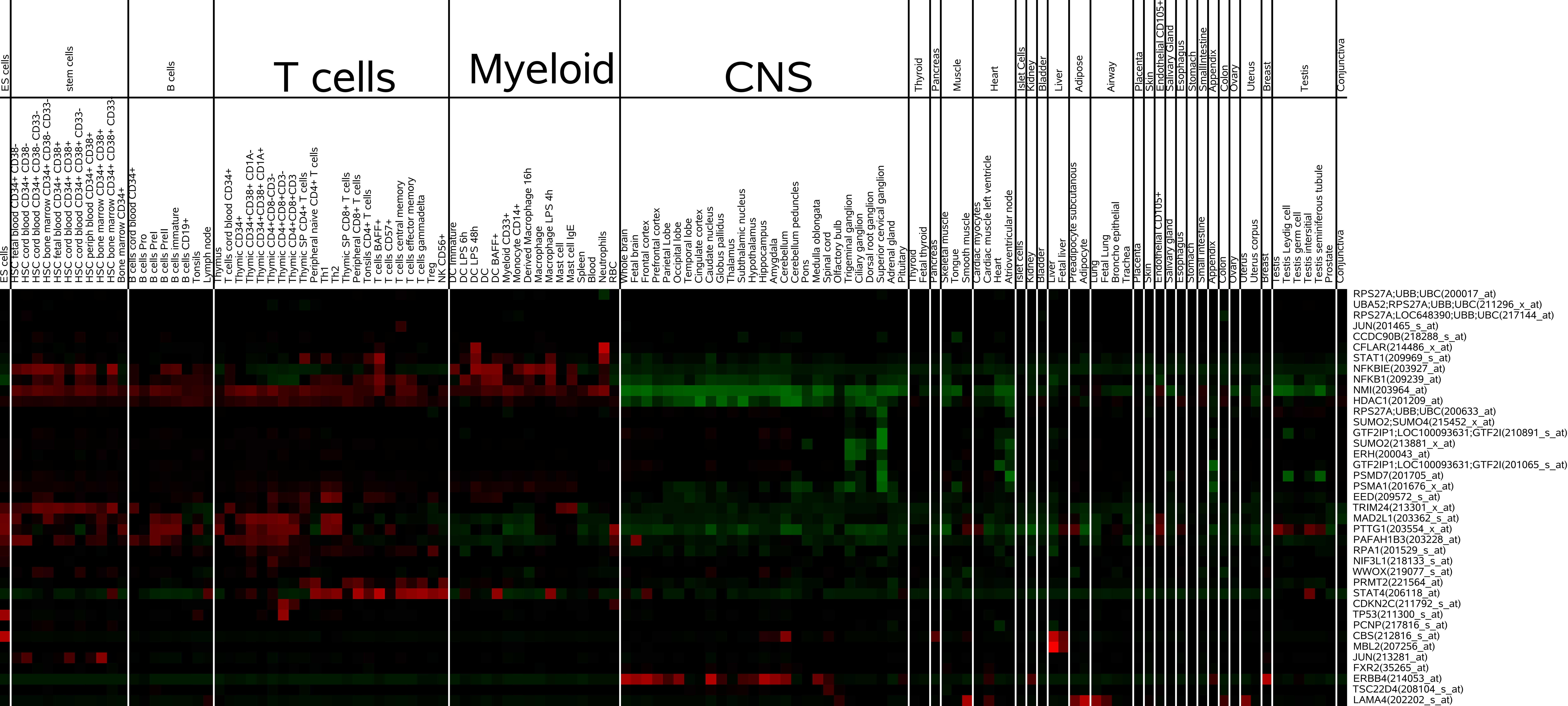
ES cells
stem cells
B cells
T cells
Myeloid
CNS
Thyroid
Pancreas
Muscle
Heart
Islet Cells
Kidney
Bladder
Liver
Adipose
Airway
Placenta
Skin
Endothelial CD105+
Salivary Gland
Esophagus
Stomach
SmallIntestine
Appendix
Colon
Ovary
Uterus
Breast
Testis
Conjunctiva
ES cells
HSC fetal blood CD34+ CD38-
HSC cord blood CD34+ CD38-
HSC cord blood CD34+ CD38- CD33-
HSC bone marrow CD34+ CD38- CD33-
HSC fetal blood CD34+ CD38+
HSC cord blood CD34+ CD38+
HSC cord blood CD34+ CD38+ CD33-
HSC periph blood CD34+ CD38+
HSC bone marrow CD34+ CD38+
HSC bone marrow CD34+ CD38+ CD33
Bone marrow CD34+
B cells cord blood CD34+
B cells Pro
B cells PreI
B cells PreII
B cells immature
B cells CD19+
Tonsils
Lymph node
Thymus
T cells cord blood CD34+
Thymic CD34+
Thymic CD34+CD38+ CD1A-
Thymic CD34+CD38+ CD1A+
Thymic CD4+CD8-CD3-
Thymic CD4+CD8+CD3-
Thymic CD4+CD8+CD3
Thymic SP CD4+ T cells
Peripheral naive CD4+ T cells
Th1
Th2
Thymic SP CD8+ T cells
Peripheral CD8+ T cells
Tonsils CD4+ T cells
T cells CD57+
T cells BAFF+
T cells central memory
T cells effector memory
T cells gammadelta
Treg
NK CD56+
DC immature
DC LPS 6h
DC LPS 48h
DC
DC BAFF+
Myeloid CD33+
Monocyte CD14+
Derived Macrophage 16h
Macrophage
Macrophage LPS 4h
Mast cell
Mast cell IgE
Spleen
Blood
Neutrophils
RBC
Whole brain
Fetal brain
Frontal cortex
Prefrontal cortex
Parietal Lobe
Occipital lobe
Temporal lobe
Cingulate cortex
Caudate nucleus
Globus pallidus
Thalamus
Subthalamic nucleus
Hypothalamus
Hippocampus
Amygdala
Cerebellum
Cerebellum peduncles
Pons
Medulla oblongata
Spinal cord
Olfactory bulb
Trigeminal ganglion
Ciliary ganglion
Dorsal root ganglion
Superior cervical ganglion
Adrenal gland
Pituitary
Thyroid
Fetal thyroid
Pancreas
Skeletal muscle
Tongue
Smooth muscle
Cardiac myocytes
Cardiac muscle left ventricle
Heart
Atrioventricular node
Islet cells
Kidney
Bladder
Liver
Fetal liver
Preadipocyte subcutanous
Adipocyte
Lung
Fetal Lung
Broncho epithelial
Trachea
Placenta
Skin
Endothelial CD105+
Salivary gland
Esophagus
Stomach
Small intestine
Appendix
Colon
Ovary
Uterus
Uterus corpus
Breast
Testis
Testis Leydig cell
Testis germ cell
Testis intersitial
Testis seminiferous tubule
Prostate
Conjunctiva
RPS27A;UBB;UBC(200017_at)
UBA52;RPS27A;UBB;UBC(211296_x_at)
RPS27A;LOC648390;UBB;UBC(217144_at)
JUN(201465_s_at)
CCDC90B(218288_s_at)
CFLAR(214486_x_at)
STAT1(209969_s_at)
NFKBIE(203927_at)
NFKB1(209239_at)
NMI(203964_at)
HDAC1(201209_at)
RPS27A;UBB;UBC(200633_at)
SUMO2;SUMO4(215452_x_at)
GTF2IP1;LOC100093631;GTF2I(210891_s_at)
SUMO2(213881_x_at)
ERH(200043_at)
GTF2IP1;LOC100093631;GTF2I(201065_s_at)
PSMD7(201705_at)
PSMA1(201676_x_at)
EED(209572_s_at)
TRIM24(213301_x_at)
MAD2L1(203362_s_at)
PTTG1(203554_x_at)
PAFAH1B3(203228_at)
RPA1(201529_s_at)
NIF3L1(218133_s_at)
WWOX(219077_s_at)
PRMT2(221564_at)
STAT4(206118_at)
CDKN2C(211792_s_at)
TP53(211300_s_at)
PCNP(217816_s_at)
CBS(212816_s_at)
MBL2(207256_at)
JUN(213281_at)
FXR2(35265_at)
ERBB4(214053_at)
TSC22D4(208104_s_at)
LAMA4(202202_s_at)

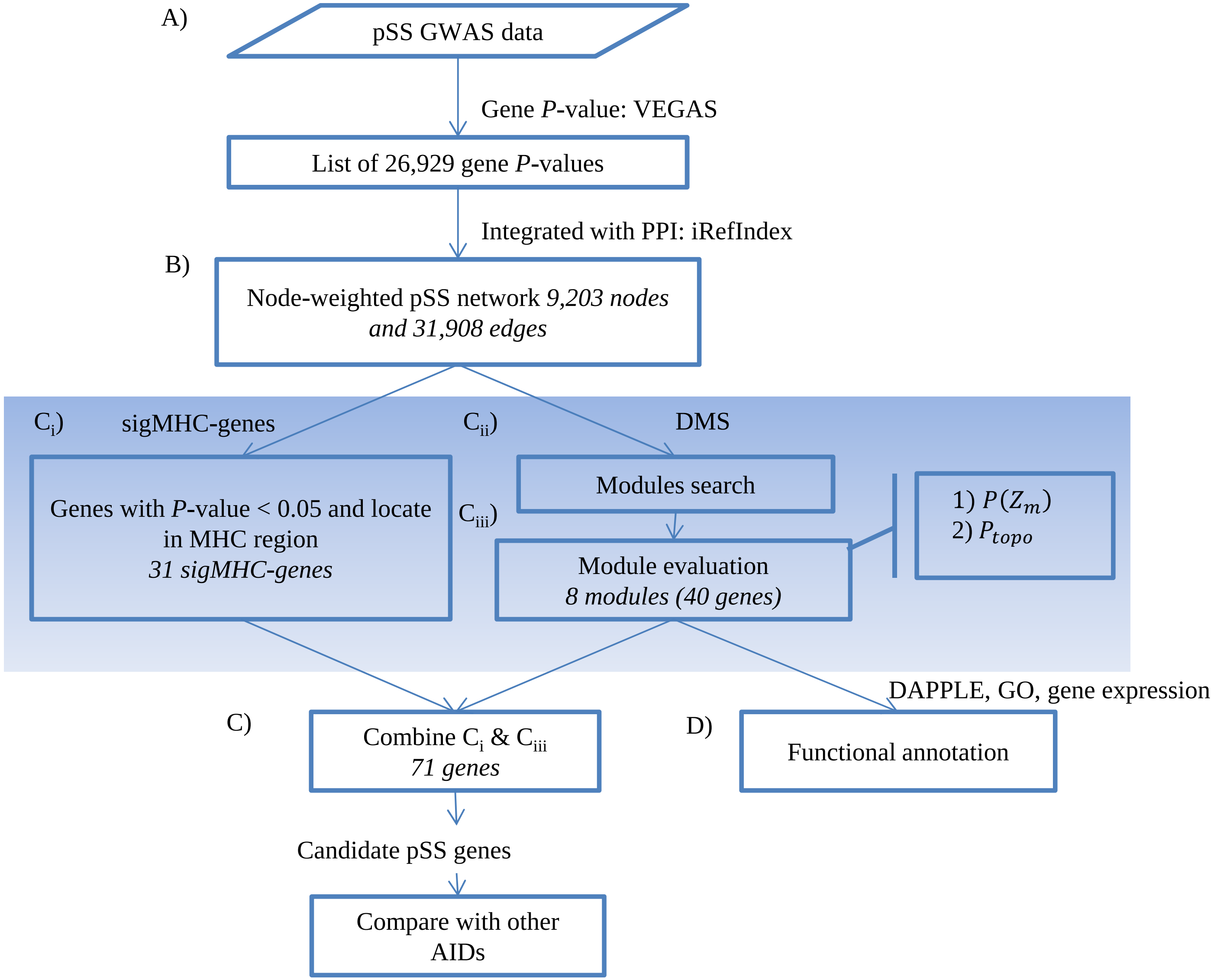

Supplementary Figure S4. Workflow of network-assisted analysis of pSS GWAS data to identify candidate genes**.** $P(Z_m)$ represents module P-value. P_{topo} represents the significance of the topology of identified modules. AIDs is short for autoimmune diseases.

Supplementary Table S1. A full list of PPI pairs involved in the node-weighted pSS interactome.

UBC	ZZEF1
SKIL	ZZEF1
PHYHIP	ZZEF1
HSPB3	ZZEF1
VAV1	ZYX
VASP	ZYX
UBC	ZYX
RPH3AL	ZYX
LASP1	ZYX
HIPK3	ZYX
ENAH	ZYX
BSPRY	ZYX
ACTN1	ZYX
UBC	ZYG11B
CUL2	ZYG11B
UBC	ZYG11A
ZW10	ZWINT
UBC	ZWINT
SPC24	ZWINT
NSL1	ZWINT
MIS12	ZWINT
DSN1	ZWINT
CASC5	ZWINT
ZW10	ZWILCH
UBC	ZWILCH
KNTC1	ZWILCH
UBC	ZW10
ZW10	RINT1
ZW10	KNTC1
UBC	ZUFSP
SHCBP1	ZUFSP
UBC	ZSWIM8
SMAD9	ZSCAN4
SMAD1	ZSCAN4
ZKSCAN7	ZSCAN32
SCAND1	ZSCAN32
SLC25A38	ZSCAN16
UBC	ZRANB3
U2AF1	ZRANB2
SRSF2	ZRANB2
SNRNP70	ZRANB2

UBE2N	ZNRF2
UBC	ZNRF2
UBE2N	ZNRF1
UBC	ZNRF1
UBC	ZNHIT6
SUMO2	ZNHIT6
SMAD2	ZNHIT6
RXRA	ZNHIT3
UBC	ZNHIT2
MAPK14	ZNHIT1
UBC	ZNFX1
UBC	ZNF93
UBC	ZNF91
UBC	ZNF865
TTC28	ZNF862
UBC	ZNF860
UBC	ZNF852
UBC	ZNF845
UBC	ZNF84
LURAP1	ZNF821
CCDC85B	ZNF821
UBC	ZNF816
UBC	ZNF813
UBC	ZNF808
SMAD5	ZNF8
SMAD2	ZNF8
NOTCH2	ZNF8
UBC	ZNF791
ZNF174	ZNF79
UBC	ZNF768
UBC	ZNF766
UBC	ZNF765
SUFU	ZNF764
CCDC85B	ZNF764
SMAD1	ZNF76
UBC	ZNF740
TRIM28	ZNF74
UBC	ZNF736
UBC	ZNF726
UBC	ZNF721
UBC	ZNF716
MDFI	ZNF707
UBC	ZNF701
UBC	ZNF682

UBC	ZNF680
LMO4	ZNF675
TRIP13	ZNF655
MTUS2	ZNF655
UBE2I	ZNF646
TRAF2	ZNF646
NINL	ZNF646
MDFI	ZNF646
UBC	ZNF644
UBC	ZNF638
KRT19	ZNF638
KRT15	ZNF638
CCDC85B	ZNF638
UBC	ZNF622
MYBL2	ZNF622
UBC	ZNF610
UBC	ZNF599
UBC	ZNF598
TRIM23	ZNF581
LZTS2	ZNF581
HNRNPC	ZNF581
DAZAP2	ZNF581
UBC	ZNF578
UBC	ZNF574
SMAD9	ZNF557
UBC	ZNF518B
UBC	ZNF518A
XPC	ZNF512B
VWF	ZNF512B
VPS41	ZNF512B
VHL	ZNF512B
SMAD1	ZNF512B
PTPRF	ZNF512B
PLCG2	ZNF512B
MYH10	ZNF512B
KDM3B	ZNF512B
KDM2A	ZNF512B
INVS	ZNF512B
IL36RN	ZNF512B
GLRX3	ZNF512B
CREBZF	ZNF512B
BTBD2	ZNF512B
AP1G1	ZNF512B
SMAD1	ZNF510

UBC	ZNF496
UBC	ZNF485
ZNF202	ZNF473
TRIM41	ZNF473
UBC	ZNF468
UBC	ZNF460
UBE2I	ZNF451
UBC	ZNF451
SUMO2	ZNF451
SNIP1	ZNF451
RAP1GDS1	ZNF451
FXR2	ZNF451
CALCOCO2	ZNF451
UBC	ZNF45
UBC	ZNF445
CSNK2B	ZNF44
UBC	ZNF430
SMAD4	ZNF423
SMAD1	ZNF423
EBF1	ZNF423
TP53	ZNF420
ZBTB43	ZNF417
TRIM37	ZNF417
MDFI	ZNF417
CEP72	ZNF417
CCDC85B	ZNF417
BEGAIN	ZNF417
ZNF330	ZNF408
UPF2	ZNF408
TOP3B	ZNF408
THAP1	ZNF408
RALYL	ZNF408
PDLIM4	ZNF408
MIF	ZNF408
MDFI	ZNF408
LSM2	ZNF408
GIPC1	ZNF408
EXOSC2	ZNF408
DDX56	ZNF408
CHAF1A	ZNF408
CEP70	ZNF408
CALCOCO2	ZNF408
DYNLL1	ZNF354A
TRIM28	ZNF350

NEK1 ZNF350
CCDC85B ZNF337
UBC ZNF330
UBC ZNF326
UBC ZNF320
UBC ZNF319
UBC ZNF318
SUMO2 ZNF318
UBC ZNF282
UBC ZNF28
UBC ZNF273
TRIM41 ZNF263
SUFU ZNF263
SPG21 ZNF263
SCAND1 ZNF263
PLEKHF2 ZNF263
NME7 ZNF263
EFEMP2 ZNF263
DVL2 ZNF263
SMAD1 ZNF251
ZSCAN32 ZNF24
ZNF174 ZNF24
ZBTB16 ZNF24
UNC119 ZNF24
TP53 ZNF24
SUMO2 ZNF24
SETDB1 ZNF24
SCAND1 ZNF24
RBM48 ZNF24
KAT5 ZNF24
HAP1 ZNF24
EEF1G ZNF24
COPS6 ZNF24
CCDC130 ZNF24
C14orf1 ZNF24
UBC ZNF229
UBC ZNF22
TRAF2 ZNF205
MDFI ZNF205
CCDC85B ZNF205
CALCOCO2 ZNF205
HAP1 ZNF20
VHL ZNF197
TRIM28 ZNF197

UBC	ZNF195
ZNF250	ZNF165
TCEB3B	ZNF165
SCAND1	ZNF165
PDLIM7	ZNF165
KAT7	ZNF165
FAM124A	ZNF165
DVL2	ZNF165
CCDC85B	ZNF165
CCDC130	ZNF165
UBC	ZNF160
UBC	ZNF148
UBC	ZNF146
MDFI	ZNF136
UBC	ZNF131
UBC	ZNF121
HIPK3	ZNF107
UBC	ZNF106
SKIL	ZNF106
TRIM28	ZNF10
TRIM24	ZNF10
ZFYVE9	ZMYND8
UBC	ZMYND8
SUMO2	ZMYND8
PPP1R37	ZMYND19
ZHX1	ZMYND11
TRAF6	ZMYND11
SMAD2	ZMYND11
PDLIM7	ZMYND11
DYNLL1	ZMYND11
TSC22D4	ZMYND10
SNIP1	ZMYM5
SKIL	ZMYM5
UBC	ZMYM4
ZNF512B	ZMYM2
UBC	ZMYM2
SUMO1	ZMYM2
SMAD4	ZMYM2
SKIL	ZMYM2
KDM1A	ZMYM2
HDAC2	ZMYM2
HDAC1	ZMYM2
UBC	ZMYM1
UBC	ZMPSTE24

PIN1	ZMIZ2
UBC	ZKSCAN1
SUMO2	ZKSCAN1
ZNF598	ZIC4
GLI2	ZIC2
GLI3	ZIC1
GLI2	ZIC1
ZHX2	ZHX3
ZHX1	WDR33
ZHX1	UTP3
ZHX1	TOLLIP
ZHX1	TNFRSF10C
ZHX1	TARDBP
ZHX1	SERPINB9
ZHX1	PSMD11
ZHX1	PNP
ZHX1	PIGC
ZHX1	MPHOSPH6
ZHX1	LAMA4
ZHX1	IDH1
ZHX1	HSPE1
ZHX1	GNG11
DYNLL1	ZHX1
ZHX1	DLST
ZHX1	DDX39B
ZHX1	BHLHE40
ZHX1	ATXN1
ZHX1	AKR1C3
ZFYVE9	UBXN11
ZFYVE9	SUMF2
ZFYVE9	SPG20
SMAD2	ZFYVE9
ZFYVE9	SCYL2
ZFYVE9	RNF11
ZFYVE9	RBBP6
ZFYVE9	PPP1CC
ZFYVE9	PPP1CA
ZFYVE9	NUP155
ZFYVE9	NIT1
ZFYVE9	JUP
ZFYVE9	ETS2
ZFYVE9	ERBB2IP
ZFYVE9	EIF4G1
DAZAP2	ZFYVE9

ZFYVE9	BMP4
ZFYVE9	BAG6
UBC	ZFYVE28
PXN	ZFYVE27
FKBP8	ZFYVE27
VPS45	ZFYVE20
RAB4A	ZFYVE20
UBC	ZFR
HNRNPA1	ZFR
ATP5J2	ZFPL1
UBC	ZFP91
C14orf1	ZFP64
UBC	ZFP36L1
NUP214	ZFP36
CCDC85B	ZFP36
UBC	ZFP14
UBC	ZFP1
NINL	ZFHX3
UBC	ZFC3H1
NINL	ZFC3H1
CCDC85B	ZFC3H1
UBC	ZFAND6
TRAF2	ZFAND6
UBC	ZFAND5
UBC	ZFAND2B
DAZAP2	ZFAND2B
UBC	ZFAND2A
UBC	ZER1
TCEB2	ZER1
CUL2	ZER1
UBC	ZEB2
SMAD5	ZEB2
SMAD2	ZEB2
SMAD1	ZEB2
COPS6	ZEB2
DRAP1	ZEB1
CTBP1	ZEB1
PBX2	ZDHHC7
UBC	ZDHHC6
UBC	ZDHHC5
SMAD1	ZDHHC3
UBC	ZDHHC13
SNIP1	ZCCHC7
UBC	ZCCHC6

UBC	ZCCHC3
UBC	ZCCHC11
TP53	ZCCHC10
THAP1	ZCCHC10
STAC3	ZCCHC10
PIN1	ZCCHC10
DPPA2	ZCCHC10
UBC	ZC3HC1
SKP1	ZC3HC1
CUL1	ZC3HC1
UBC	ZC3HAV1L
UBC	ZC3HAV1
UBC	ZC3H7B
UBC	ZC3H7A
UBC	ZC3H15
DRG1	ZC3H15
UBC	ZC3H14
KRT20	ZC3H14
EXOC7	ZC3H14
AHSP	ZC3H12A
ATXN1	ZC3H10
KRT20	ZC2HC1C
KRT15	ZC2HC1C
CCDC85B	ZC2HC1C
CALCOCO2	ZC2HC1C
UBC	ZBTB9
UBC	ZBTB8OS
GRAP2	ZBTB7B
NCOR2	ZBTB7A
SKIL	ZBTB6
NCOR2	ZBTB6
BCL6	ZBTB6
UBC	ZBTB5
ST3GAL3	ZBTB5
CCDC85B	ZBTB5
TRAF2	ZBTB43
ZBTB43	TCEB3B
ZBTB43	LNX1
ZBTB43	LMO3
SKIL	ZBTB3
TRAF2	ZBTB25
MDFI	ZBTB25
MDFI	ZBTB24
TOPBP1	ZBTB17

PGR ZBTB17
MYC ZBTB17
MSX2 ZBTB17
ITGB5 ZBTB17
HK3 ZBTB17
GTF2I ZBTB17
ESR1 ZBTB17
DNMT3A ZBTB17
DIS3 ZBTB17
CDKN2B ZBTB17
ZBTB16 WDR33
ZBTB16 VTA1
UBE2I ZBTB16
ZBTB16 TRIM27
TRAF2 ZBTB16
ZBTB16 TOLLIP
ZBTB16 SUMO1
ZBTB16 SPOP
ZBTB16 SIN3A
ZBTB16 RUNX1T1
ZBTB16 QTRT1
ZBTB16 PSMD11
ZBTB16 PMAIP1
ZBTB16 NCOR2
LSM2 ZBTB16
ZBTB16 LAMTOR5
ZBTB16 HDAC4
ZBTB16 HDAC1
ZBTB16 HBEGF
ZBTB16 GSTM4
ZBTB16 GCSH
ZBTB16 FSHR
ZBTB16 FCHO1
ZBTB16 FAM101B
ZBTB16 ENOX1
ZBTB16 DLST
ZBTB16 COQ6
ZBTB16 CIDEA
CEP72 ZBTB16
CEP70 ZBTB16
ZBTB16 CD81
CCDC85B ZBTB16
ZBTB16 CCDC130
ZBTB16 BCL6

ZBTB16	ANAPC5
ZBTB16	ACP5
UBC	ZBTB14
UBC	ZBTB10
SUMO2	ZBTB10
UBC	ZBTB1
SUMO2	ZBTB1
SMAD4	ZBED5
VAV1	ZAP70
UBE2L3	ZAP70
UBC	ZAP70
PTPN6	ZAP70
LCP2	ZAP70
LCK	ZAP70
LAT	ZAP70
ERBB3	ZAP70
EGFR	ZAP70
CRKL	ZAP70
CRK	ZAP70
CD247	ZAP70
CBLB	ZAP70
UBC	YY1AP1
SUMO2	YY1AP1
MAPK14	YY1AP1
ZRANB2	YY1
UCHL5	YY1
UBC	YY1
TP53	YY1
SP1	YY1
SMAD2	YY1
RUVBL2	YY1
RUVBL1	YY1
PRMT1	YY1
MYC	YY1
MDM2	YY1
INO80E	YY1
INO80	YY1
HDAC4	YY1
HDAC2	YY1
GTF2I	YY1
EED	YY1
E2F2	YY1
ATF7	YY1
ATF6	YY1

ACTR5	YY1
ACTL6A	YY1
ZNRF2	YWHAQ
YWHAG	YWHAQ
YWHAE	YWHAQ
YWHAB	YWHAQ
YAP1	YWHAQ
WDR77	YWHAQ
VIM	YWHAQ
USP8	YWHAQ
UBC	YWHAQ
TSC1	YWHAQ
TERT	YWHAQ
SSH1	YWHAQ
SRSF3	YWHAQ
SMAD9	YWHAQ
RPS3	YWHAQ
RGS7	YWHAQ
RAI14	YWHAQ
RAF1	YWHAQ
PRMT5	YWHAQ
PRKD1	YWHAQ
PIK3CB	YWHAQ
PI4KB	YWHAQ
PDE3A	YWHAQ
NPM1	YWHAQ
NFATC4	YWHAQ
NFATC2	YWHAQ
NEDD4L	YWHAQ
MYCBP2	YWHAQ
MDM2	YWHAQ
MAP3K5	YWHAQ
MAGOH	YWHAQ
LMNA	YWHAQ
KRT9	YWHAQ
KIF5B	YWHAQ
KIF23	YWHAQ
IRS2	YWHAQ
HSPA8	YWHAQ
HSPA1A	YWHAQ
HNRNPC	YWHAQ
HIST4H4	YWHAQ
HDAC7	YWHAQ
HDAC4	YWHAQ

FSHR	YWHAQ
FGR	YWHAQ
EPB41L3	YWHAQ
CRTC2	YWHAQ
CEP170	YWHAQ
CDKN1B	YWHAQ
CDK18	YWHAQ
CDC25B	YWHAQ
CALM1	YWHAQ
BRAF	YWHAQ
BAD	YWHAQ
ATP5A1	YWHAQ
AKT1S1	YWHAQ
ABL1	YWHAQ
ZFP36	YWHAH
YWHAQ	YWHAH
YWHAE	YWHAH
UBC	YWHAH
TSC1	YWHAH
TPH1	YWHAH
TNFAIP3	YWHAH
RAF1	YWHAH
PDK1	YWHAH
NCOA3	YWHAH
NCOA2	YWHAH
NCOA1	YWHAH
MED1	YWHAH
MARK4	YWHAH
MAP3K5	YWHAH
KLC3	YWHAH
KCNH2	YWHAH
HDAC7	YWHAH
HDAC4	YWHAH
ESR2	YWHAH
ESR1	YWHAH
EPB41L3	YWHAH
CSNK1E	YWHAH
CHRNA4	YWHAH
CDKN1B	YWHAH
CDC25B	YWHAH
BRAF	YWHAH
BAD	YWHAH
YWHAG	ZFP36
YWHAG	ZBTB21

YWHAG	YWHAE
YWHAG	YAP1
YWHAG	VIM
YWHAG	USP8
UBC	YWHAG
YWHAG	TUBB
YWHAG	TSC1
YWHAG	TRA2A
TP53	YWHAG
YWHAG	TNFAIP3
YWHAG	TMEM102
YWHAG	TJP2
YWHAG	TIAM1
YWHAG	THRAP3
YWHAG	SRSF3
YWHAG	SRPK1
YWHAG	SRGAP2
YWHAG	SNRNP200
SHCBP1	YWHAG
YWHAG	SH3BP5L
YWHAG	SH3BP4
YWHAG	SF3B3
YWHAG	SAMD4A
YWHAG	RNPS1
YWHAG	RASSF8
YWHAG	RASAL2
YWHAG	RALGPS2
YWHAG	RAI14
YWHAG	RAF1
YWHAG	RACGAP1
YWHAG	RABEP1
YWHAG	RAB11FIP2
YWHAG	PUF60
YWHAG	PRPF38B
YWHAG	PRKCG
YWHAG	PRKCD
YWHAG	PRKCB
YWHAG	PRKCA
YWHAG	PPIG
YWHAG	PNN
YWHAG	PIK3R1
YWHAG	PIK3C3
YWHAG	PI4KB
YWHAG	PARD3

YWHAG	OSBPL3
YWHAG	NOLC1
YWHAG	NEFL
YWHAG	MYCBP2
YWHAG	MICALL1
YWHAG	MFAP1
YWHAG	LSR
YWHAG	LBR
YWHAG	KIF5B
YWHAG	KIF23
YWHAG	KIF1C
YWHAG	KIF1B
YWHAG	IRS2
YWHAG	HSPB6
YWHAG	HSPA1A
YWHAG	HNRNPH1
YWHAG	HDAC7
HDAC4	YWHAG
YWHAG	GSK3A
YWHAG	FOXO1
YWHAG	ERC1
YWHAG	EPB41L2
YWHAG	EML3
EEF1G	YWHAG
YWHAG	EDC3
YWHAG	DYRK1A
YWHAG	DHX15
YWHAG	DFFA
YWHAG	DENND4A
DDX39B	YWHAG
YWHAG	CRTC2
YWHAG	CLK1
YWHAG	CDK17
YWHAG	CCNY
YWHAG	C1QBP
YWHAG	C16orf80
YWHAG	BRAF
YWHAG	BAIAP2
YWHAG	ATP5B
YWHAG	ATP5A1
YWHAG	ARHGEF7
YWHAG	ARHGEF2
YWHAG	AKAP13
YWHAG	ACIN1

YWHAE	WWTR1
YWHAE	USP8
UBC	YWHAE
YWHAE	TSC1
YWHAE	TNFAIP3
YWHAE	SORBS2
YWHAE	PRKAA1
YWHAE	NPM1
YWHAE	MAPK7
YWHAE	KIF1C
YWHAE	KCNH2
YWHAE	HIST4H4
YWHAE	HDAC7
HDAC4	YWHAE
GRAP2	YWHAE
YWHAE	GPRIN2
YWHAE	FOXO3
YWHAE	EXO1
YWHAE	CDC25A
ATXN1	YWHAE
YWHAB	ZFP36
YWHAE	YWHAB
YWHAB	YAP1
YWHAB	WEE1
YWHAB	USP8
UBC	YWHAB
YWHAB	TSC1
YWHAB	TNFAIP3
YWHAB	TJP2
YWHAB	TESK2
YWHAB	TESK1
YWHAB	SSH1
YWHAB	SRSF3
YWHAB	SRPK2
YWHAB	SRPK1
YWHAB	RPS6KA1
YWHAB	RNPS1
YWHAB	RIN1
YWHAB	RGS7
YWHAB	RALGPS2
YWHAB	RAI14
YWHAB	RAF1
YWHAB	RACGAP1
YWHAB	PIK3R4

YWHABPI4KB
YWHABPDE3B
YWHABPARD3
YWHABOSBPL3
YWHABMYCBP2
YWHABMICALL1
YWHABMAPT
YWHABMAPK7
YWHABMAP3K5
YWHABMAP2K1
YWHABLSR
YWHABKIF23
YWHABKIF1C
YWHABKCNK3
YWHABITGB2
YWHABITGB1
YWHABIRS2
YWHABIRS1
YWHABHSPA5
YWHABHSPA1A
YWHABHDAC7
HDAC4 YWHAB
YWHABFOXO3
YWHABEXO1
YWHABEDC3
YWHABDHX15
YWHABDENND4A
YWHABDAPK1
YWHABCRTC2
YWHABCDC25B
YWHABCDC25A
YWHABBID
YWHABBAD
YWHABALS2
YWHABAKAP13
YWHABACIN1
UBC YTHDF2
UBC YTHDF1
UBC YTHDC2
UBC YTHDC1
UBC YRDC
UBC YPEL5
UBC YOD1
UBC YME1L1

UBC	YLPM1
PRPF40A	YLPM1
PRMT1	YLPM1
PPP1CC	YLPM1
PPP1CA	YLPM1
CHGB	YLPM1
BMP1	YLPM1
APBB1	YLPM1
UBC	YKT6
YIPF3	YIPF4
UBC	YIPF4
UBC	YIPF3
UBC	YIPF1
UBC	YIF1B
YIPF5	YIF1A
UBC	YIF1A
ZNF512B	YES1
YAP1	YES1
UBC	YES1
TYRO3	YES1
TRPV4	YES1
PTPRE	YES1
OCLN	YES1
KHDRBS1	YES1
JAK2	YES1
GP6	YES1
CSF1R	YES1
CDCP1	YES1
CD46	YES1
CD36	YES1
CD2AP	YES1
ADAM12	YES1
UBC	YEATS4
NUMA1	YEATS4
UBC	YDJC
UBC	YBX2
NEDD4	YBX1
HABP4	YBX1
UBC	YARS
YAP1	RUNX2
YAP1	ERBB4
YY1	YAF2
UBC	YAF2
RNF2	YAF2

RING1	YAF2
MYCN	YAF2
CSNK2A2	YAF2
VIM	YAE1D1
UBC	XYLB
UBC	XXYLT1
UPF2	XRN2
UBC	XRN2
TOLLIP	XRN2
TIPARP	XRN2
TARDBP	XRN2
EXOSC10	XRN2
EIF5A	XRN2
EIF3L	XRN2
EEF1A1	XRN2
ZFP36	XRN1
WAPAL	XRN1
UPF1	XRN1
UBC	XRN1
STAM2	XRN1
SPATA20	XRN1
SMARCD2	XRN1
SKIV2L2	XRN1
PABPC4	XRN1
LSM4	XRN1
EXOSC8	XRN1
EXOSC1	XRN1
DPP7	XRN1
DCP1B	XRN1
CHD4	XRN1
CCT5	XRN1
BAG6	XRN1
AP1G2	XRN1
ZNF512B	XRCC6
WRN	XRCC6
WBP4	XRCC6
VAV1	XRCC6
UBC	XRCC6
TERT	XRCC6
TERF2IP	XRCC6
TERF2	XRCC6
SUMO2	XRCC6
SKIL	XRCC6
SIRT1	XRCC6

SERPINB9	XRCC6
RRAS2	XRCC6
RPLP1	XRCC6
PXN	XRCC6
PTTG1	XRCC6
PRPF40A	XRCC6
PRKDC	XRCC6
POR	XRCC6
PLGRKT	XRCC6
PIN1	XRCC6
PGAM1	XRCC6
PARP1	XRCC6
PAEP	XRCC6
NIT1	XRCC6
NCOA6	XRCC6
NCF4	XRCC6
NAA15	XRCC6
MSX2	XRCC6
LIG4	XRCC6
KAT2A	XRCC6
ILVBL	XRCC6
HOXC4	XRCC6
HN1L	XRCC6
HIST1H3A	XRCC6
H2AFX	XRCC6
GSE1	XRCC6
GAL3ST4	XRCC6
FMNL1	XRCC6
ESR1	XRCC6
EGFR	XRCC6
DNTT	XRCC6
CREBBP	XRCC6
CLU	XRCC6
CHD1L	XRCC6
CDCA5	XRCC6
BAX	XRCC6
ATP6V1E1	XRCC6
ARAP1	XRCC6
APEX1	XRCC6
ABL1	XRCC6
ABCD4	XRCC6
XRCC6	XRCC5
WRN	XRCC5
UBC	XRCC5

TERT	XRCC5
TERF2IP	XRCC5
TERF2	XRCC5
SUMO2	XRCC5
PXN	XRCC5
PRKDC	XRCC5
POU2F1	XRCC5
PARP1	XRCC5
ORC2	XRCC5
NFKB2	XRCC5
NCOA6	XRCC5
NAA15	XRCC5
MSX2	XRCC5
LIG4	XRCC5
KAT2A	XRCC5
HIST1H3A	XRCC5
H2AFX	XRCC5
ESR1	XRCC5
DHX9	XRCC5
CHD1L	XRCC5
APLF	XRCC5
VIM	XRCC4
UBC	XRCC4
NHEJ1	XRCC4
LIG4	XRCC4
IFFO1	XRCC4
CAAP1	XRCC4
APTX	XRCC4
APLF	XRCC4
ACTN1	XRCC4
UBC	XRCC3
FANCG	XRCC3
RAD51D	XRCC2
BLM	XRCC2
UBC	XPR1
UBC	XPOT
TNFRSF1B	XPOT
TNFRSF1A	XPOT
NUP214	XPOT
NUP153	XPOT
UBC	XPO7
DDX19B	XPO7
NUP62	XPO6
UBC	XPO5

TNFRSF1B	XPO5
SMAD4	XPO5
RAN	XPO5
NUP214	XPO5
NUP153	XPO5
ILF3	XPO5
FADD	XPO5
EEF1A1	XPO5
YWHAE	XPO1
UBC	XPO1
TUBB	XPO1
TP53	XPO1
TOP2A	XPO1
TNFSF12-TNFSF13	XPO1
TNFRSF1B	XPO1
SUMO2	XPO1
SNUPN	XPO1
SMURF1	XPO1
SMAD1	XPO1
RCC1	XPO1
RANBP1	XPO1
RAN	XPO1
PSMC2	XPO1
NUP62	XPO1
NUP214	XPO1
NUP153	XPO1
NPM1	XPO1
NMD3	XPO1
ERF	XPO1
EIF5A	XPO1
E2F5	XPO1
CRK	XPO1
CIITA	XPO1
CDKN1B	XPO1
ADAR	XPO1
UBC	XPNPEP3
UBC	XPNPEP1
POLA2	XPNPEP1
RPA1	XPA
XCL2	XCR1
XCL1	XCR1
UBC	XBP1
SUMO2	XBP1
FOS	XBP1

ESR1 XBP1
ATF6 XBP1
XAF1 HTRA2
UBC XAB2
SUMO2 XAB2
SNW1 XAB2
PRPF31 XAB2
PPIE XAB2
POLR2A XAB2
PLRG1 XAB2
CACHD1 XAB2
WWTR1 TEAD4
WWTR1 SLC9A3R2
VASP WWP2
UBE2L3 WWP2
UBE2E1 WWP2
UBC WWP2
TP53BP2 WWP2
SMAD2 WWP2
SF1 WWP2
SCNN1B WWP2
RNF11 WWP2
PTEN WWP2
POU5F1 WWP2
PDLIM7 WWP2
DAG1 WWP2
CPSF6 WWP2
ATN1 WWP2
ARRDC1 WWP2
ZNF638 WWP1
WBP1 WWP1
UBC WWP1
TP63 WWP1
SPG20 WWP1
SMAD7 WWP1
SMAD6 WWP1
SMAD5 WWP1
SMAD3 WWP1
SMAD2 WWP1
SMAD1 WWP1
RNF11 WWP1
RASA1 WWP1
LAPTM5 WWP1
KLF5 WWP1

KLF2	WWP1
EWSR1	WWP1
ERBB4	WWP1
DIDO1	WWP1
DAZAP2	WWP1
CPSF6	WWP1
ATN1	WWP1
WIPF2	WWOX
WDR33	WWOX
UBC	WWOX
TP53	WWOX
TNK2	WWOX
TCERG1	WWOX
SMARCC1	WWOX
SFPQ	WWOX
SF3B3	WWOX
SF3B1	WWOX
RPRD2	WWOX
POLR2B	WWOX
PABPC1	WWOX
NUDT21	WWOX
FIP1L1	WWOX
ERBB4	WWOX
CPSF7	WWOX
CPSF6	WWOX
CPSF3	WWOX
CPSF2	WWOX
UBC	WTAP
UBC	WSB1
TCEB1	WSB1
DIO2	WSB1
CUL5	WSB1
UBC	WRNIP1
PIN1	WRNIP1
UBC	WRB
UBC	WRAP73
FZD5	WNT5A
LRP6	WNT3A
SFRP1	WNT2
FZD9	WNT1
UBC	WNK4
SLC12A3	WNK4
UBC	WLS
OPRM1	WLS

UBC	WIZ
EHMT2	WIZ
CTBP1	WIZ
UBC	WIPI2
WIPF2	WASL
WIPF2	TCERG1
WIPF2	GRB2
APBB1	WIPF2
WBP4	WIPF1
WASL	WIPF1
NCK1	WIPF1
HCK	WIPF1
GRB2	WIPF1
CRKL	WIPF1
APBB1	WIPF1
HDAC2	WHSC1
HDAC1	WHSC1
TRIP13	WDYHV1
WDYHV1	SFN
WDYHV1	RPIA
WDYHV1	NECAB2
JUP	WDYHV1
WDYHV1	HSD17B14
WDYHV1	CBFA2T2
VCL	WDTC1
UBC	WDTC1
MED23	WDTC1
UBC	WDR92
UBC	WDR89
PHYHIP	WDR89
UBC	WDR82
UBC	WDR81
UBC	WDR77
WDR77	PRMT5
UBC	WDR76
UBC	WDR75
UBC	WDR74
UBC	WDR73
UBC	WDR70
MAPK9	WDR62
MAPK8	WDR62
UBC	WDR61
TGFBR1	WDR61
RIPK3	WDR61

LEO1	WDR61
CDC73	WDR61
ACVR1	WDR61
UBC	WDR59
USF1	WDR5
UBC	WDR5
TADA2A	WDR5
PAXIP1	WDR5
NCOA6	WDR5
MLLT1	WDR5
MEN1	WDR5
HIST3H3	WDR5
CHD8	WDR5
WDR20	WDR48
USP46	WDR48
USP1	WDR48
UBC	WDR48
ATAD5	WDR48
TCEA2	WDR47
FAM107A	WDR47
UBC	WDR45B
UBC	WDR43
SUMO2	WDR43
UBC	WDR36
SUMO2	WDR36
RRP9	WDR36
UBC	WDR34
TP53	WDR33
RBM48	WDR33
WDR33	PFN2
EEF1G	WDR33
UBC	WDR26
APPBP2	WDR24
WDR20	USP46
UBC	WDR12
PES1	WDR12
BOP1	WDR12
UBC	WDR11
UBC	WDHD1
KAT2A	WDHD1
UBC	WDFY3
SQSTM1	WDFY3
UBC	WBSCR22
WBP4	WBP11

WBP4	TCERG1
WBP4	SNRPA
SNRNP70	WBP4
WBP4	SFPQ
WBP4	SF3B4
WBP4	SF3B3
WBP4	SF3B1
WBP4	SF3A3
WBP4	SF3A2
WBP4	SF1
WBP4	SCAF4
WBP4	SART1
WBP4	PSPC1
WBP4	PRPF4
WBP4	KHDRBS1
WBP4	HLTF
WBP4	FUS
WBP4	EFTUD2
WBP4	DNM3
WBP4	DHX15
WBP4	DDX23
WBP4	CPSF7
WBP4	CPSF6
WBP4	CHERP
WBP4	CAPRIN1
WBP4	BUB3
WWOX	WBP2
UBC	WBP2
SEPT3	WBP2
NEDD4	WBP2
UBC	WBP1L
UBC	WBP11
PPP1CA	WBP11
WBP11	MCRS1
YAP1	WBP1
WASL	RHOQ
WASL	PFN1
WASL	PACSIN3
WASL	ITSN1
WASL	CDC42
UBC	WASF2
TCERG1	WASF2
PXN	WASF2
PSTPIP1	WASF2

GAS7	WASF2
CDC42	WASF2
BAIAP2	WASF2
PSTPIP1	WASF1
PFN1	WASF1
FYN	WASF1
CDC42	WASF1
BRK1	WASF1
ACTG1	WASF1
ABI2	WASF1
UBC	WARS
MDH2	WARS
WAPAL	SMC3
WAPAL	PDS5B
WAPAL	PDS5A
UBC	WAC
KRT15	WAC
UBC	VWA5A
UBC	VTI1B
TNK2	VTI1B
STX8	VTI1B
UBC	VTI1A
VTA1	VPS4A
VTA1	RPIA
VTA1	NECAB2
VTA1	KLHL12
VTA1	KCTD13
DPPA2	VTA1
VTA1	CDK4
VTA1	CBS
FAM131C	VSNL1
UBC	VRK2
MAP2K1	VRK2
UBC	VRK1
TP53	VRK1
SMAD9	VPS8
UBC	VPS53
VPS52	VPS51
UBC	VPS51
VPS4A	VPS4B
UBC	VPS4A
VPS4A	CHMP4B
VPS4A	CHMP1B
VPS41	VPS33A

VPS41	VPS18
VPS41	VPS16
VPS28	VPS37C
UBC	VPS37C
TSG101	VPS37C
VPS28	VPS37A
UBC	VPS37A
UBAP1	VPS37A
TSG101	VPS37A
VPS25	VPS36
UBC	VPS36
TSG101	VPS36
SNF8	VPS36
UBC	VPS33B
VPS33A	VPS18
VPS33A	VPS16
VPS28	VPS37B
VPS28	VPS25
UBC	VPS28
UBC	VPS26A
VPS18	VPS16
UBC	VPS13C
UBC	VPS13A
UBC	VPS11
IGLL1	VPREB1
UBC	VPRBP
TERT	VPRBP
PSMD7	VPRBP
NEDD8	VPRBP
COPS7A	VPRBP
COPS6	VPRBP
COPS5	VPRBP
COPS4	VPRBP
UBC	VOPP1
UBC	VMP1
UBC	VLDLR
SERPINE1	VLDLR
RELN	VLDLR
MYLIP	VLDLR
LRPAP1	VLDLR
LPL	VLDLR
DAB1	VLDLR
CLU	VLDLR
APOE	VLDLR

UBC	VKORC1L1
VPS33B	VIPAS39
VIPR2	VIP
VIPR1	VIP
MEOX2	VIP
VIM	VCL
VIM	UPP1
TRIM28	VIM
VIM	RAD51
VIM	PPL
VIM	PLA2G4A
VIM	PAK2
VIM	MICAL1
VIM	MEN1
VIM	GSK3B
VIM	GOPC
DYNLL1	VIM
VIM	DSP
VIM	BFSP1
VIM	ALS2CR11
VIM	ABLIM1
PLCG1	VIL1
ACTG1	VIL1
ACTA1	VIL1
UBC	VHL
VHL	TP53
VHL	TCP1
VHL	TCEB2
VHL	RPS3A
VHL	RPS3
VHL	PSMC3
VHL	OSR1
VHL	HSPA8
VHL	HDAC2
VHL	FN1
CUL2	VHL
VHL	COL4A1
VHL	CCT7
VHL	ACTB
UBC	VEZT
SMAD1	VEPH1
ACVR1	VEPH1
KDR	VEGFC
FLT4	VEGFC

SPARC	VEGFA
KDR	VEGFA
FLT1	VEGFA
FGF5	VEGFA
ZBTB16	VDR
UBC	VDR
TCF3	VDR
SNW1	VDR
SMARCA4	VDR
SMAD3	VDR
RXRG	VDR
RXRB	VDR
RXRA	VDR
PSMC5	VDR
PPP1CC	VDR
POLR2A	VDR
NCOR2	VDR
NCOA3	VDR
NCOA2	VDR
NCOA1	VDR
MED6	VDR
MED24	VDR
MED13	VDR
MED1	VDR
JUN	VDR
HR	VDR
GTF2B	VDR
CREBBP	VDR
CDK7	VDR
BAZ1B	VDR
UBC	VDAC2
PARK2	VDAC2
FAM162A	VDAC2
BAK1	VDAC2
UBC	VDAC1
TOMM20	VDAC1
SNCA	VDAC1
PRKCE	VDAC1
PARK2	VDAC1
HSPA9	VDAC1
HK1	VDAC1
GSN	VDAC1
DYNLT1	VDAC1
CYCS	VDAC1

BCL2L11	VDAC1
BCL2L1	VDAC1
BAX	VDAC1
BAK1	VDAC1
WRN	VCP
VIMP	VCP
VCPIP1	VCP
UBXN6	VCP
UBE4B	VCP
UBC	VCP
SVIP	VCP
STX5	VCP
SPRTN	VCP
PSMA2	VCP
NPLOC4	VCP
NGLY1	VCP
INSIG1	VCP
HTT	VCP
HSPA8	VCP
FAF2	VCP
DERL2	VCP
CUL2	VCP
COPS5	VCP
BAG6	VCP
ATXN3	VCP
ASPSCR1	VCP
APOB	VCP
ANKZF1	VCP
AMFR	VCP
VCL	TLN1
VCL	SNRPG
VCL	SNRPA
VCL	SLC16A5
SFPQ	VCL
VCL	SERPINH1
VCL	PSMA2
VCL	PFN1
NUDT21	VCL
VCL	MORC1
HSPE1	VCL
VCL	BIRC6
VCL	AVEN
VCL	ACTG1
VCL	ABI2

XCL1	VCAN
SELL	VCAN
PLA2G2A	VCAN
ITGB1	VCAN
HAPLN1	VCAN
FBLN1	VCAN
CCL5	VCAN
ITGB1	VCAM1
CCL22	VCAM1
ROS1	VAV3
PIK3R1	VAV3
EGFR	VAV3
ERBB3	VAV2
EGFR	VAV2
CD44	VAV2
CD19	VAV2
CBLB	VAV2
VAV1	TUBB
VAV1	LCK
VASP	TRIM9
VASP	PRKG1
VASP	PFN2
VASP	PFN1
UBC	VASN
VAMP2	VAPB
UBC	VAPB
UBC	VAPA
SIK1	VAPA
UBC	VANGL1
STX6	VAMP4
STX4	VAMP4
STX16	VAMP4
PACS1	VAMP4
AP1M1	VAMP4
UBC	VAMP3
NELFE	VAMP3
VAMP2	VAPA
VAMP2	VAMP8
UBC	VAMP2
VAMP2	STX4
VAMP2	STX1B
VAMP2	STX1A
VAMP2	STX11
VAMP2	SNAP25

VAMP2	NAPA
VAMP2	CPLX1
VAPB	VAMP1
VAPA	VAMP1
UBC	VAMP1
VPS33A	UVRAG
VPS18	UVRAG
UBC	UVRAG
UTS2	UTS2R
UBC	UTRN
PTPN12	UTRN
DTNB	UTRN
DAG1	UTRN
UBC	UTP6
CCDC85B	UTP6
UBC	UTP3
HAP1	UTP3
UBC	UTP20
UBC	UTP18
UBC	UTP11L
UBC	USP8
USP8	STAM2
GRAP2	USP8
EGFR	USP8
UVSSA	USP7
UHRF1	USP7
UBC	USP7
TRAF6	USP7
TRAF2	USP7
TRAF1	USP7
TP53	USP7
TANK	USP7
RNF220	USP7
RNF2	USP7
RING1	USP7
PTEN	USP7
MDM2	USP7
MARCH7	USP7
KAT5	USP7
GMPS	USP7
DNMT1	USP7
DAXX	USP7
ATXN1	USP7
UBC	USP6NL

UBC	USP54
UBC	USP5
TADA3	USP5
SUMO2	USP5
RNF11	USP5
RAD23A	USP5
DVL2	USP5
UBC	USP48
UBC	USP46
UBC	USP45
TGFBR1	USP45
SMAD1	USP45
USP15	USP4
UBC	USP4
TP53BP2	USP4
RIPK1	USP4
UBC	USP38
UBC	USP36
UBC	USP34
VHL	USP33
UBC	USP33
TCEB1	USP33
ROBO1	USP33
DIO2	USP33
DDX56	USP33
ARRB2	USP33
UBC	USP25
SKIL	USP25
UBC	USP24
UBC	USP22
TRRAP	USP22
TAF9	USP22
SUPT3H	USP22
SIRT1	USP22
PSME1	USP22
KAT2A	USP22
ENY2	USP22
ATXN7L3	USP22
ATXN7	USP22
UCHL1	USP21
UBC	USP21
TGFBR1	USP21
MARK1	USP21
EXOSC10	USP21

ACVR1	USP21
USP21	USP20
NEURL4	USP20
DIO2	USP20
UBC	USP2
SCNN1A	USP2
KRT15	USP2
FASN	USP2
CCDC85B	USP2
UBC	USP16
UBC	USP15
SMAD2	USP15
SMAD1	USP15
UBC	USP14
PSMD7	USP14
PSMD4	USP14
PSMD14	USP14
PSMD13	USP14
PSMD12	USP14
PSMC6	USP14
PSMC5	USP14
PSMC4	USP14
PSMC3	USP14
UBC	USP13
TRIM63	USP13
LAPTM5	USP13
WDR48	USP12
WDR20	USP12
UBC	USP10
G3BP1	USP10
CFTR	USP10
USP1	FANCD2
UBC	USO1
STX5	USO1
RAB1A	USO1
PLCG1	USO1
NSF	USO1
GABPB1	USO1
EEA1	USO1
COPB1	USO1
CASP3	USO1
UBC	USMG5
RIBC2	USHBP1
NOC4L	USHBP1

MED28	USHBP1
KRT19	USHBP1
KRT15	USHBP1
KANSL1	USHBP1
GCC1	USHBP1
FANCG	USHBP1
FAM107A	USHBP1
EXOC8	USHBP1
EXOC7	USHBP1
COPS4	USHBP1
CEP63	USHBP1
CCNK	USHBP1
C1orf216	USHBP1
C10orf10	USHBP1
BET1	USHBP1
ABLIM1	USHBP1
USH1C	USH1G
USF1	USF2
UBC	USF2
PPARGC1A	USF2
USF1	TRRAP
USF1	RBBP4
USF1	CHD8
UBC	UROD
ASCC2	URM1
POLR2E	URI1
UBC	URGCP
UBC	URB1
UBC	UQCRQ
ERCC8	UQCRQ
UBC	UQCRFS1
UBC	UQCRC2
UBC	UQCRB
UPF2	UPF3A
UPF1	UPF3A
SMG1	UPF3A
PIGT	UPF3A
GSK3B	UPF3A
ALYREF	UPF3A
UPF2	ZFP36
UPF2	UPF1
UPF2	SKIV2L2
UPF2	RPS15
UPF2	NOB1

UPF2	EXOSC10
UPF2	EXOSC1
UPF2	DCP2
UPF2	DCP1B
UBC	UPF1
UPF1	TRMT112
UPF1	SMG7
UPF1	SMG5
UPF1	RPRD2
UPF1	PLEKHB2
UPF1	HIRA
UPF1	GSPT1
EXOSC2	UPF1
UPF1	DCP2
UPF1	DCP1A
UPF1	ACSS2
UPF1	ABHD16A
UBC	UNG
UBC	UNC50
UBC	UNC45A
SMAD9	UNC45A
UBC	UNC13B
STX1B	UNC13B
STX1A	UNC13B
RPH3AL	UNC13B
RIMS1	UNC13B
DOC2B	UNC13B
DOC2A	UNC13B
UNC119	ZFP64
UNC119	UBQLN4
UNC119	TRDMT1
UNC119	TP53BP2
UNC119	TK1
UNC119	SULT1E1
UNC119	SERPINB9
UNC119	SEPHS1
UNC119	S100A8
UNC119	RUFY3
UNC119	RGL2
UNC119	RFC5
UNC119	QTRT1
UNC119	PSMD2
UNC119	PSMD11
UNC119	PPAP2C

UNC119 PPA1
UNC119 PHF10
UNC119 PFN1
UNC119 PAPSS1
UNC119 ORAI2
UNC119 MRPS12
UNC119 MPHOSPH6
UNC119 MAPK10
UNC119 LCK
UNC119 LAMA4
UNC119 KDM1A
UNC119 ITSN1
UNC119 HSPE1
HSPB3 UNC119
UNC119 HCK
UNC119 GSTM4
UNC119 GIPC2
UNC119 FYN
UNC119 FXYD6-FXYD2
UNC119 FKBPL
UNC119 ERH
UNC119 COX17
UNC119 CKS2
UNC119 CKMT2
UNC119 CETN3
UNC119 CDKN2C
UNC119 CDC42
UNC119 CD4
UNC119 CD247
UNC119 CCL18
UNC119 CBX8
UNC119 C16orf74
BTBD2 UNC119
UNC119 BRIX1
UNC119 BCR
UNC119 ARL4D
UNC119 ARL3
UNC119 ARL2
UNC119 ANXA3
UNC119 ALDH2
UNC119 AAGAB
UBC UMPS
DLST UMPS
CDC42EP3 UMPS

SETDB1	ULK2
POLA2	ULK2
NASP	ULK2
GABARAPL2	ULK2
GABARAP	ULK2
EEF1B2	ULK2
UBC	UIMC1
SKIL	UIMC1
MDC1	UIMC1
UBC	UHRF2
TP53	UHRF2
SMAD4	UHRF2
PCNP	UHRF2
HDAC1	UHRF2
CDK2	UHRF2
CCNE1	UHRF2
UBC	UHMK1
UBC	UGGT1
RTN1	UGCG
UFC1	UFM1
UBC	UFM1
UBA5	UFM1
UBC	UFL1
CDK5RAP3	UFL1
VCP	UFD1L
USP13	UFD1L
UBC	UFD1L
NPLOC4	UFD1L
FAF1	UFD1L
UBC	UFC1
UFC1	UBA5
UFC1	CDK5RAP3
UBC	UEVLD
YWHAQ	UCP3
YWHAB	UCP3
YWHAQ	UCP2
YWHAB	UCP2
CRHR1	UCN
UBC	UCK2
UBC	UCK1
UBC	UCHL5
UCHL5	RUVBL2
UCHL5	PSMD4
UCHL5	PSMD3

PSMD11	UCHL5
UCHL5	PSMD1
UCHL5	PSMC3
UCHL5	PSMC2
UCHL5	PSMA2
UCHL5	MCRS1
UCHL5	INO80E
UCHL5	ACTR5
UCHL5	ACTL6A
VCP	UBXN8
UBC	UBXN8
VCP	UBXN7
UBC	UBXN7
TCEB1	UBXN7
NEDD8	UBXN7
CUL2	UBXN7
CUL1	UBXN7
VCP	UBXN4
UBC	UBXN4
TOM1L1	UBXN4
UBC	UBXN2B
VCP	UBXN2A
UBC	UBXN2A
TRAF2	UBXN11
UBXN11	C6orf165
VCP	UBXN1
UBC	UBXN1
CUL2	UBXN1
UBC	UBTF
TBP	UBTF
TAF1C	UBTF
RUNX2	UBTF
POLR1A	UBTF
MYBBP1A	UBTF
KAT5	UBTF
IRS2	UBTF
IRS1	UBTF
CHGB	UBTF
CD3EAP	UBTF
CCNA2	UBTF
UBC	UBTD2
UBC	UBTD1
UBC	UBR7
UBE2D2	UBR5

UBC	UBR5
SUMO2	UBR5
SMAD2	UBR5
PGR	UBR5
PAIP2	UBR5
PAIP1	UBR5
MCRS1	UBR5
KPNA1	UBR5
UBC	UBR4
PRPF40A	UBR4
UBE2B	UBR3
UBC	UBR3
DNAJB11	UBR1
BTBD2	UBR1
UBQLN4	ZBTB22
UBC	UBQLN4
UBQLN4	SPP1
UBQLN4	SMARCB1
UBQLN4	SCG5
RXRA	UBQLN4
PSMD4	UBQLN4
ATXN1	UBQLN4
VCP	UBQLN1
UBQLN4	UBQLN1
UBC	UBQLN1
TFF1	UBQLN1
RIC8A	UBQLN1
PSMD4	UBQLN1
PSEN1	UBQLN1
GABRD	UBQLN1
EFEMP2	UBQLN1
APP	UBQLN1
UBE2L3	UBOX5
HIRA	UBN1
CABIN1	UBN1
UBC	UBLCP1
TSC22D4	UBLCP1
PSMD2	UBLCP1
UBE4B	UBL7
UBC	UBL7
RAD23A	UBL7
ERCC1	UBL7
BAG6	UBL7
UBC	UBFD1

UBE2D3	UBE4A
UBC	UBE4A
UBC	UBE3B
UBE2L6	UBE3A
UBE2L3	UBE3A
UBE2D3	UBE3A
UBE2D2	UBE3A
UBC	UBE3A
TP53	UBE3A
SCRIB	UBE3A
RARA	UBE3A
RAD23A	UBE3A
PSMD4	UBE3A
PGR	UBE3A
ESR1	UBE3A
CFTR	UBE3A
UBC	UBE2Z
UBA6	UBE2Z
TGFBR1	UBE2Z
SRA1	UBE2Z
SMAD4	UBE2Z
SMAD1	UBE2Z
PLEKHF2	UBE2Z
LPL	UBE2Z
UBE2W	STUB1
UBE2W	AMFR
UBE2N	UBE2V2
UBC	UBE2V2
SUV39H2	UBE2V2
BCL10	UBE2V2
RNF144B	UBE2U
UBC	UBE2T
FANCL	UBE2T
UBC	UBE2S
FZR1	UBE2S
BIRC2	UBE2S
UBC	UBE2Q1
UBE2N	RNF103
UBE2N	BCL10
UBE2N	AMFR
UBC	UBE2M
NEDD8	UBE2M
NAE1	UBE2M
DCUN1D4	UBE2M

DCUN1D1	UBE2M
CUL1	UBE2M
CPNE4	UBE2M
CPNE2	UBE2M
CPNE1	UBE2M
APP	UBE2M
UBC	UBE2L6
UBE2L6	RNF144B
UBE2L6	RNF144A
UBE2L3	PTEN
UBC	UBE2K
TP53	UBE2K
RNF2	UBE2K
RNF185	UBE2K
NFKB1	UBE2K
NEDD8	UBE2K
NEDD4L	UBE2K
MDM2	UBE2K
HTT	UBE2K
UBC	UBE2J2
UBC	UBE2J1
RNF5	UBE2J1
PARK2	UBE2J1
BIRC2	UBE2J1
UBE2I	UBE2K
UBC	UBE2I
UBE2I	UBA2
UBE2I	TRIM55
UBE2I	TRIM29
UBE2I	TRIM27
UBE2I	TP63
UBE2I	TP53
UBE2I	TOP1
UBE2I	TNFRSF1A
UBE2I	TDP2
UBE2I	SUMO3
UBE2I	SUMO1
UBE2I	SREBF2
UBE2I	SREBF1
UBE2I	SNIP1
UBE2I	SMAD4
UBE2I	SLC2A4
SKIL	UBE2I
UBE2I	SIAH2

UBE2I	SIAH1
UBE2I	SCNN1A
UBE2I	RIPK2
UBE2I	RANGAP1
UBE2I	RAD54B
UBE2I	RAD52
UBE2I	RAD51
UBE2I	PSMD9
UBE2I	PIAS3
UBE2I	PIAS1
UBE2I	PHC1
UBE2I	PARP1
UBE2I	NUDCD3
UBE2I	MYB
UBE2I	MITF
UBE2I	KCNK1
UBE2I	IPO13
UBE2I	IKZF3
UBE2I	IKZF1
UBE2I	HSPB1
UBE2I	HSF2
UBE2I	HNRNPC
UBE2I	HIPK2
UBE2I	HDAC4
UBE2I	FHIT
UBE2I	EXOSC9
UBE2I	ETS1
UBE2I	DNMT3A
UBE2I	DAXX
UBE2I	DACH1
UBE2I	CHMP4B
UBE2I	CASP8AP2
UBE2I	BRCA1
UBC	UBE2H
UBC	UBE2G2
PARK2	UBE2G2
AUP1	UBE2G2
AMFR	UBE2G2
UBE3A	UBE2G1
UBE2E1	UBE2G1
UBC	UBE2G1
PARK2	UBE2G1
ITCH	UBE2G1
DIO2	UBE2G1

CBL	UBE2G1
AMFR	UBE2G1
UBC	UBE2E3
RNF5	UBE2E3
RNF167	UBE2E3
NFKBIA	UBE2E3
CNOT4	UBE2E3
CHUK	UBE2E3
UBOX5	UBE2E2
UBC	UBE2E2
STUB1	UBE2E2
SIAH1	UBE2E2
RNF8	UBE2E2
RNF220	UBE2E2
DZIP3	UBE2E2
BRCA1	UBE2E2
UBE2E1	ZNRF1
UBE2E1	UBOX5
UBC	UBE2E1
UBE2E1	STUB1
UBE2E1	RNF5
UBE2E1	RNF167
NEDD4	UBE2E1
UBE2E1	ITCH
UBE2E1	IPO11
UBE2E1	CNOT4
ZNRF1	UBE2D4
UBC	UBE2D4
TRIM27	UBE2D4
RNF126	UBE2D4
RNF11	UBE2D4
RFWD3	UBE2D4
AMFR	UBE2D4
UBE2D3	ZNRF1
UBE2D3	UBE4B
UBC	UBE2D3
UBE2D3	TRIM31
TRIM27	UBE2D3
UBE2D3	STUB1
UBE2D3	SMURF2
UBE2D3	RNF5
UBE2D3	RNF168
UBE2D3	RNF126
UBE2D3	RNF125

UBE2D3	OTUB1
NEDD4	UBE2D3
MDM2	UBE2D3
UBE2D3	DTX3L
UBE2D3	BIRC2
UBE2D3	AMFR
UBE2D2	ZNRF1
UBE2D2	VPRBP
UBE2D2	UBE4B
UBC	UBE2D2
UBE2D2	TRIM5
UBE2D2	TRIM31
TRIM27	UBE2D2
UBE2D2	TRIM25
UBE2D2	TRIM17
UBE2D2	STUB1
UBE2D2	SIAH2
UBE2D2	SIAH1
UBE2D2	RNF5
UBE2D2	RNF38
UBE2D2	RNF26
RNF2	UBE2D2
UBE2D2	RNF126
UBE2D2	RNF115
RNF11	UBE2D2
UBE2D2	RFWD3
UBE2D2	RFWD2
UBE2D2	RCHY1
UBE2D2	PARK2
UBE2D2	OTUB1
NEDD4L	UBE2D2
NEDD4	UBE2D2
UBE2D2	MUL1
MDM2	UBE2D2
UBE2D2	MARCH5
LNX1	UBE2D2
UBE2D2	DZIP3
UBE2D2	DTX3L
UBE2D2	CNOT4
UBE2D2	BRCA1
UBE2D2	BIRC7
UBE2D2	BIRC3
UBE2D2	BIRC2
UBE2D2	AMFR

UBE2D1	ZNRF1
UBE2D1	UHRF2
UBE2D1	UFM1
UBE2D1	UBE3C
UBE2D1	UBE3A
UBC	UBE2D1
UBE2D1	UBA52
UBE2D1	TRIP12
UBE2D1	TRIM39
UBE2D1	TRIM32
UBE2D1	TRIM31
TRIM27	UBE2D1
UBE2D1	TRIM25
UBE2D1	TRIM2
UBE2D1	TRIM17
TP53	UBE2D1
UBE2D1	STUB1
SKP1	UBE2D1
UBE2D1	SIAH1
UBE2D1	RNF8
UBE2D1	RNF7
UBE2D1	RNF4
UBE2D1	RNF25
RNF2	UBE2D1
UBE2D1	RNF13
UBE2D1	RNF126
UBE2D1	RNF125
UBE2D1	RNF114
RNF11	UBE2D1
UBE2D1	RFWD3
UBE2D1	OTUB1
UBE2D1	NFKBIA
NEDD4L	UBE2D1
NEDD4	UBE2D1
UBE2D1	MYLIP
UBE2D1	MUL1
UBE2D1	MGRN1
UBE2D1	MDM4
MDM2	UBE2D1
UBE2D1	DZIP3
UBE2D1	DTX3L
UBE2D1	CUL3
CUL2	UBE2D1
UBE2D1	CBL

UBE2D1	BRCA1
UBE2D1	BIRC3
UBE2D1	BIRC2
UBE2D1	AMFR
UBC	UBE2C
FZR1	UBE2C
CDC20	UBE2C
CDC16	UBE2C
ANAPC7	UBE2C
ANAPC5	UBE2C
ANAPC4	UBE2C
ANAPC1	UBE2C
UBE2B	UBR2
UBE2B	UBR1
UBC	UBE2B
UBE2B	RAD18
VIM	UBD
VCP	UBD
USO1	UBD
UBC	UBD
UBA6	UBD
TP53	UBD
TGFBR1	UBD
SND1	UBD
PSMD4	UBD
PSMD2	UBD
PSMC3	UBD
PSMA6	UBD
PRMT5	UBD
PPIA	UBD
NUB1	UBD
NCL	UBD
MTHFD1	UBD
MAD2L1	UBD
IARS	UBD
HNRNPF	UBD
FUS	UBD
ENO1	UBD
EEF1G	UBD
EEF1A1	UBD
DHX9	UBD
COPB1	UBD
ALB	UBD
ACTN4	UBD

ACTB	UBD
ACLY	UBD
UBC	ZSCAN21
UBC	ZRANB1
UBC	ZNF600
UBC	ZNF347
UBC	ZNF281
UBC	ZNF208
UBC	ZKSCAN7
UBC	ZFYVE16
UBC	ZCCHC8
UBC	ZC3H11A
UBC	ZBTB11
UBC	YEATS2
UBC	YBX3
UBC	YBX1
UBC	YARS2
UBC	YAP1
UBC	XRCC1
UBC	XPC
UBC	WRN
UBC	WFS1
UBC	WEE1
UBC	WDR41
UBC	WDR35
UBC	WDR33
UBC	WDR3
UBC	WDR18
UBC	WDR1
UBC	VWA9
UBC	VPS52
UBC	VPS4B
UBC	VPS45
UBC	VPS41
UBC	VPS39
UBC	VPS37B
UBC	VPS33A
UBC	VPS25
UBC	VPS18
UBC	VPS16
UBC	VPS13B
UBC	VIMP
UBC	VIM
UBC	VDAC3

UBC	VCPIP1
UBC	VCL
UBC	VAV1
UBC	VAT1
UBC	VASP
UBC	VARS2
UBC	VARS
UBC	VAMP8
UBC	UTP15
UBC	USP47
UBC	USP42
UBC	USP37
UBC	USP32
UBC	USP3
UBC	USP28
UBC	USP18
UBC	USP1
UBC	USF1
UBC	URB2
UBC	UQCR10
UBC	UNC13D
UBC	UHRF1
UBC	UGDH
UBC	UCKL1
UBC	UCHL3
UBC	UCHL1
UBC	UBXN6
UBC	UBR2
UBC	UBR1
UBC	UBE4B
UBC	UBE3C
UBC	UBE2R2
UBC	UBE2O
UBC	UBE2N
UBC	UBE2L3
UBC	UBAP2
UBC	UAP1
UBC	UACA
UBC	TYRO3
UBC	TXNL4A
UBC	TXNDC5
UBC	TUSC3
UBC	TUFT1
UBC	TUBGCP5

UBC	TUBB8
UBC	TUBB6
UBC	TUBB3
UBC	TUBB
UBC	TUBA3E
UBC	TTK
UBC	TTI2
UBC	TTC8
UBC	TTC33
UBC	TTC3
UBC	TSSC1
UBC	TSFM
UBC	TSC1
UBC	TRRAP
UBC	TRPM2
UBC	TRPC4AP
UBC	TRNT1
UBC	TRMT1L
UBC	TRIP4
UBC	TRIM62
UBC	TRIM56
UBC	TRIM47
UBC	TRIM41
UBC	TRIM33
UBC	TRIM28
UBC	TRIM27
UBC	TRIM11
UBC	TRAPPC8
UBC	TRAPPC13
UBC	TRAPPC1
UBC	TRAIP
UBC	TRAF1
UBC	TPX2
UBC	TPD52L2
UBC	TP63
UBC	TP53
UBC	TOMM7
UBC	TOMM5
UBC	TOMM40
UBC	TOMM22
UBC	TOE1
UBC	TNPO2
UBC	TNK2
UBC	TNIK

UBC	TNFRSF9
UBC	TNFRSF1A
UBC	TNFRSF10D
UBC	TNFRSF10B
UBC	TNFRSF10A
UBC	TMPO
UBC	TMOD3
UBC	TMEM9B
UBC	TMEM87A
UBC	TMEM41B
UBC	TMEM38B
UBC	TMEM248
UBC	TMEM206
UBC	TMEM194A
UBC	TMEM192
UBC	TMEM135
UBC	TM9SF4
UBC	TM7SF3
UBC	TLN1
UBC	TJP1
UBC	TIMM50
UBC	TIMM21
UBC	TIMM17A
UBC	TIMELESS
UBC	TIAL1
UBC	THUMPD1
UBC	THOP1
UBC	THOC3
UBC	THOC1
UBC	TGFBR2
UBC	TFIP11
UBC	TFDP1
UBC	TEX10
UBC	TELO2
UBC	TEAD4
UBC	TEAD2
UBC	TCP1
UBC	TCF3
UBC	TCEB2
UBC	TBK1
UBC	TBCE
UBC	TBC1D17
UBC	TBC1D10A
UBC	TAP1

UBC	TAOK2
UBC	TANK
UBC	TAF9
UBC	TAF7
UBC	TADA1
UBC	TACC2
UBC	SYNJ2
UBC	SYNE1
UBC	SUPT6H
UBC	SUPT4H1
UBC	SUMF2
UBC	SULT1A1
UBC	SUFU
UBC	SUCLG1
UBC	SUB1
UBC	STX6
UBC	STX5
UBC	STX17
UBC	STT3A
UBC	STRAP
UBC	STON2
UBC	STMN1
UBC	STK24
UBC	STK17B
UBC	STIP1
UBC	STIL
UBC	STEAP3
UBC	STAT6
UBC	STAT1
UBC	STAMBPL1
UBC	STAM2
UBC	ST3GAL4
UBC	SSX2IP
UBC	SSR3
UBC	SRSF3
UBC	SRSF2
UBC	SRSF11
UBC	SRPK2
UBC	SRPK1
UBC	SRGAP2
UBC	SREBF1
UBC	SPTBN2
UBC	SPRYD7
UBC	SPRTN

UBC	SPRED2
UBC	SPHK1
UBC	SPECC1L
UBC	SOX10
UBC	SNX17
UBC	SNW1
UBC	SNRPN
UBC	SNRPG
UBC	SNRPB2
UBC	SNRPA
UBC	SNRNP70
UBC	SNRNP40
UBC	SNF8
UBC	SMURF2
UBC	SMEK1
UBC	SMC5
UBC	SMARCD3
UBC	SMARCB1
UBC	SMARCA2
UBC	SMAP1
UBC	SMAD5
UBC	SMAD2
UBC	SMAD1
UBC	SLU7
UBC	SLC9A3R2
UBC	SLC9A3R1
UBC	SLC46A1
UBC	SLC3A2
UBC	SLC39A7
UBC	SLC35B2
UBC	SLC33A1
UBC	SLC30A7
UBC	SLC27A4
UBC	SLC25A3
UBC	SLC1A4
UBC	SLC12A3
UBC	SLC12A2
UBC	SKIV2L2
UBC	SKIL
UBC	SKA3
UBC	SIRT2
UBC	SIL1
UBC	SIK1
UBC	SHCBP1

UBC	SH2D4A
UBC	SGTB
UBC	SFXN4
UBC	SFPQ
UBC	SF3A3
UBC	SET
UBC	SESN1
UBC	SERPINH1
UBC	SERPINB9
UBC	SERPINA1
UBC	SEPT4
UBC	SEC61A1
UBC	SEC24D
UBC	SEC24A
UBC	SEC14L4
UBC	SEC14L1
UBC	SDCCAG3
UBC	SDC4
UBC	SDC2
UBC	SDAD1
UBC	SCYL3
UBC	SCRN1
UBC	SCRIB
UBC	SCD
UBC	SCARB1
UBC	SCAP
UBC	SCAF8
UBC	SCAF11
UBC	SASH1
UBC	SART1
UBC	SAP18
UBC	SAMHD1
UBC	SAMD9
UBC	SACM1L
UBC	SAAL1
UBC	S100A11
UBC	RUVBL2
UBC	RUNX2
UBC	RTCA
UBC	RSL24D1
UBC	RSL1D1
UBC	RRP9
UBC	RRP15
UBC	RRAS

UBC	RQCD1
UBC	RPTOR
UBC	RPS6KC1
UBC	RPS6KA4
UBC	RPS6
UBC	RPS3A
UBC	RPS3
UBC	RPS14
UBC	RPS11
UBC	RPL8
UBC	RPL7L1
UBC	RPL35A
UBC	RPL26L1
UBC	RPL24
UBC	RPL22L1
UBC	RPL18
UBC	RPL17-C18orf32
UBC	RPL14
UBC	RPIA
UBC	RPA1
UBC	ROR2
UBC	RNMT
UBC	RNF5
UBC	RNF220
UBC	RNF168
UBC	RNF103
UBC	RNF10
UBC	RNASET2
UBC	RMDN1
UBC	RINT1
UBC	RIMKLB
UBC	RHPN2
UBC	RHOT1
UBC	RHOQ
UBC	RHOC
UBC	RHOB
UBC	RHO
UBC	RHEB
UBC	RFWD2
UBC	RFC5
UBC	REST
UBC	RERE
UBC	RDH11
UBC	RCC1

UBC	RBM39
UBC	RBFOX2
UBC	RBBP4
UBC	RAVER1
UBC	RASGRP2
UBC	RAP2B
UBC	RAP1GDS1
UBC	RAP1B
UBC	RANBP10
UBC	RALYL
UBC	RALGPS2
UBC	RAD51
UBC	RAD18
UBC	RAD1
UBC	RACGAP1
UBC	RABEP1
UBC	RAB7A
UBC	RAB33B
UBC	RAB12
UBC	RAB11FIP1
UBC	RAB11A
UBC	QPRT
UBC	PYROXD1
UBC	PYCARD
UBC	PVRL1
UBC	PUS7
UBC	PURB
UBC	PTTG1
UBC	PTRH2
UBC	PTP4A1
UBC	PTGES2
UBC	PTEN
UBC	PTBP3
UBC	PTBP1
UBC	PSMG3
UBC	PSMD4
UBC	PSMD3
UBC	PSMD11
UBC	PSMD1
UBC	PSMC3
UBC	PSMC2
UBC	PSMB4
UBC	PSMB1
UBC	PSMA6

UBC	PSMA5
UBC	PSMA2
UBC	PSAP
UBC	PRSS3
UBC	PRRC2C
UBC	PRMT5
UBC	PRMT3
UBC	PRKD2
UBC	PRKCD
UBC	PRKCA
UBC	PRKAR1A
UBC	PRKACB
UBC	PRIM1
UBC	PRDX5
UBC	PRDX2
UBC	PRADC1
UBC	PPP4R1
UBC	PPP2R5A
UBC	PPP2R2A
UBC	PPP2R1B
UBC	PPP1R3C
UBC	PPP1R15B
UBC	PPM1F
UBC	PPM1B
UBC	PPL
UBC	PPIA
UBC	POU5F1
UBC	POLR3A
UBC	POLR2E
UBC	POLR1B
UBC	POLR1A
UBC	POLH
UBC	POLE3
UBC	POLE2
UBC	POLD2
UBC	POLD1
UBC	POLA2
UBC	POGK
UBC	PODXL
UBC	PNPLA8
UBC	PNPLA2
UBC	PNP
UBC	PNMA2
UBC	PNMA1

UBC	PMVK
UBC	PLOD3
UBC	PLK1
UBC	PLIN3
UBC	PLEKHA1
UBC	PLCXD2
UBC	PLCB4
UBC	PLBD2
UBC	PLA2G4A
UBC	PKMYT1
UBC	PITHD1
UBC	PIP4K2A
UBC	PIP
UBC	PIN1
UBC	PIK3R4
UBC	PIK3CD
UBC	PIK3C2A
UBC	PIGX
UBC	PIGK
UBC	PHB
UBC	PGR
UBC	PGAM1
UBC	PFN2
UBC	PFN1
UBC	PFKFB3
UBC	PFDN5
UBC	PFAS
UBC	PEX5
UBC	PELO
UBC	PELI1
UBC	PDE3B
UBC	PDCD6IP
UBC	PDCD11
UBC	PDCD10
UBC	PCYOX1
UBC	PCGF6
UBC	PCBP3
UBC	PBK
UBC	PARP4
UBC	PARG
UBC	PARD6B
UBC	PARD3
UBC	PAN3
UBC	PAN2

UBC	PALLD
UBC	PAK2
UBC	PAIP2
UBC	PAICS
UBC	PAFAH1B1
UBC	PACS1
UBC	PABPC3
UBC	PABPC1L
UBC	OTUD6B
UBC	OTUD4
UBC	OTUD1
UBC	ORC5
UBC	ORC4
UBC	OLA1
UBC	OGFR
UBC	OGDH
UBC	OCIAD1
UBC	OBFC1
UBC	NXF1
UBC	NUSAP1
UBC	NUP98
UBC	NUP54
UBC	NUP205
UBC	NUP188
UBC	NUDT21
UBC	NUDT1
UBC	NUDCD2
UBC	NUDC
UBC	NUCB2
UBC	NSMCE2
UBC	NRSN2
UBC	NRAS
UBC	NQO2
UBC	NQO1
UBC	NPR2
UBC	NOTCH2
UBC	NOS1
UBC	NOP2
UBC	NOMO1
UBC	NOLC1
UBC	NOL6
UBC	NOL11
UBC	NOD2
UBC	NOB1

UBC	NMT1
UBC	NME3
UBC	NME2
UBC	NLN
UBC	NIT2
UBC	NIN
UBC	NGLY1
UBC	NFX1
UBC	NFKB2
UBC	NFE2L2
UBC	NFE2L1
UBC	NFATC2IP
UBC	NET1
UBC	NEMF
UBC	NELFE
UBC	NEIL2
UBC	NEDD4
UBC	NDUFS7
UBC	NDUFS3
UBC	NDUFB10
UBC	NDUFA9
UBC	NDUFA8
UBC	NDUFA7
UBC	NDUFA12
UBC	NDNL2
UBC	NDFIP2
UBC	NDFIP1
UBC	NCOR2
UBC	NCOA4
UBC	NCOA2
UBC	NCAPG
UBC	NBN
UBC	NBEA
UBC	NAT10
UBC	NAMPT
UBC	NAA15
UBC	N4BP1
UBC	MZT1
UBC	MYSM1
UBC	MYO9B
UBC	MYO1G
UBC	MYO1D
UBC	MYO1B
UBC	MYO18B

UBC	MYH9
UBC	MYH7B
UBC	MYD88
UBC	MYCN
UBC	MYC
UBC	MYB
UBC	MTMR9
UBC	MTMR6
UBC	MTMR14
UBC	MTHFD2
UBC	MTDH
UBC	MTA2
UBC	MSTO1
UBC	MSRA
UBC	MSI2
UBC	MRPS30
UBC	MRPS28
UBC	MRPS18B
UBC	MRPS15
UBC	MRPS12
UBC	MRPS11
UBC	MRPL39
UBC	MRPL21
UBC	MRPL15
UBC	MRI1
UBC	MRE11A
UBC	MPDU1
UBC	MMS22L
UBC	MLST8
UBC	MLF1
UBC	MLEC
UBC	MIS18A
UBC	MIOS
UBC	MINPP1
UBC	MICALL1
UBC	MICAL1
UBC	MIA3
UBC	MGST1
UBC	MFSD1
UBC	MFN2
UBC	METTL7A
UBC	METTL3
UBC	METTL2B
UBC	METTL13

UBC	MERTK
UBC	MEN1
UBC	MED4
UBC	MED30
UBC	MED22
UBC	MDN1
UBC	MDM2
UBC	MDC1
UBC	MCRS1
UBC	MCM4
UBC	MCAM
UBC	MC1R
UBC	MBLAC2
UBC	MAT1A
UBC	MARK4
UBC	MARK1
UBC	MARCH5
UBC	MAPT
UBC	MAPK6
UBC	MAP7D1
UBC	MAP3K6
UBC	MAP3K5
UBC	MAP3K14
UBC	MAP2K7
UBC	MAP1S
UBC	MAN2C1
UBC	MAN1B1
UBC	MAD2L1
UBC	MACF1
UBC	LZTR1
UBC	LYPLAL1
UBC	LTV1
UBC	LTBR
UBC	LSM5
UBC	LSM4
UBC	LRRC59
UBC	LRRC40
UBC	LRRC16A
UBC	LRRC1
UBC	LNX2
UBC	LMAN1
UBC	LLGL1
UBC	LIN9
UBC	LIN7C

UBC	LDLRAP1
UBC	LCK
UBC	LAPTM4A
UBC	LAP3
UBC	LANCL1
UBC	LAMP1
UBC	L3MBTL3
UBC	KYNU
UBC	KRT7
UBC	KRT6C
UBC	KRT6A
UBC	KRT5
UBC	KRT4
UBC	KRT25
UBC	KRT19
UBC	KRT17
UBC	KRT15
UBC	KRT14
UBC	KRT10
UBC	KPNA4
UBC	KPNA2
UBC	KPNA1
UBC	KNTC1
UBC	KLHL9
UBC	KLHL24
UBC	KLHL22
UBC	KLHL12
UBC	KIT
UBC	KIRREL
UBC	KIF5A
UBC	KIF23
UBC	KIF1A
UBC	KIAA1598
UBC	KIAA1551
UBC	KIAA0391
UBC	KIAA0020
UBC	KDM3B
UBC	KDM2A
UBC	KDELR2
UBC	KCTD9
UBC	KCTD20
UBC	KCNH2
UBC	KCMF1
UBC	KAT2B

UBC	JAM3
UBC	JAK3
UBC	JAK1
UBC	ITSN2
UBC	ITK
UBC	ITGB5
UBC	ITGB2
UBC	ITGB1
UBC	IRF2BPL
UBC	IRF2BP2
UBC	IREB2
UBC	IQGAP2
UBC	IPO8
UBC	IPO7
UBC	INTS2
UBC	INSIG1
UBC	INPPL1
UBC	INPP5B
UBC	INADL
UBC	IMPA2
UBC	ILK
UBC	IL6ST
UBC	IL2RB
UBC	IL1RAP
UBC	IL18
UBC	IK
UBC	IFT57
UBC	IFRD1
UBC	IFNAR1
UBC	IFIH1
UBC	IFI35
UBC	IDH2
UBC	IDE
UBC	ID3
UBC	ICAM1
UBC	HTT
UBC	HSPH1
UBC	HSPE1
UBC	HSPB11
UBC	HSPA8
UBC	HSPA6
UBC	HSF2
UBC	HRSP12
UBC	HNRNPC

UBC	HNRNPA3
UBC	HNRNPA1
UBC	HNRNPA0
UBC	HN1
UBC	HMGCR
UBC	HMGB2
UBC	HM13
UBC	HK1
UBC	HIVEP2
UBC	HIST1H2BK
UBC	HIST1H2BD
UBC	HIST1H2AI
UBC	HIST1H2AD
UBC	HIST1H1D
UBC	HIST1H1C
UBC	HIST1H1B
UBC	HIRA
UBC	HIP1R
UBC	HIBCH
UBC	HERC4
UBC	HERC1
UBC	HEATR3
UBC	HDAC11
UBC	HADHA
UBC	HACL1
UBC	H3F3C
UBC	H3F3A
UBC	H2AFY
UBC	GTSE1
UBC	GTPBP3
UBC	GTF3C5
UBC	GTF3C3
UBC	GTF3C2
UBC	GTF2I
UBC	GTF2H3
UBC	GTF2A2
UBC	GSTM1
UBC	GSTK1
UBC	GSPT1
UBC	GSK3B
UBC	GSK3A
UBC	GSE1
UBC	GRIN2D
UBC	GRIA1

UBC	GPR37
UBC	GPN3
UBC	GOLPH3L
UBC	GNL3
UBC	GNL2
UBC	GNL1
UBC	GNG7
UBC	GNB2
UBC	GNAT3
UBC	GNAQ
UBC	GMPPA
UBC	GMIP
UBC	GMFB
UBC	GLYR1
UBC	GLI3
UBC	GLG1
UBC	GINS4
UBC	GHR
UBC	GFPT2
UBC	GEMIN6
UBC	GCN1L1
UBC	GCLC
UBC	GBA
UBC	GATA3
UBC	GAN
UBC	GALE
UBC	GABRB3
UBC	GABRB2
UBC	GABRB1
UBC	G3BP2
UBC	FZR1
UBC	FURIN
UBC	FRG1
UBC	FOXRED1
UBC	FNIP1
UBC	FN3KRP
UBC	FN1
UBC	FMNL2
UBC	FMNL1
UBC	FLCN
UBC	FKBP8
UBC	FIBP
UBC	FERMT3
UBC	FER

UBC	FBXO42
UBC	FBXO21
UBC	FBXO18
UBC	FBP1
UBC	FASTKD2
UBC	FASTKD1
UBC	FASN
UBC	FARSA
UBC	FANCL
UBC	FANCD2
UBC	FAM96A
UBC	FAM83H
UBC	FAM63A
UBC	FAM208A
UBC	FAM188A
UBC	FAM175B
UBC	FAM162A
UBC	FAF2
UBC	FADD
UBC	EXOSC9
UBC	EXOSC7
UBC	EXOC7
UBC	EXOC4
UBC	EXOC1
UBC	EXO1
UBC	ETV6
UBC	ETHE1
UBC	ERLIN2
UBC	ERLEC1
UBC	ERH
UBC	ERCC4
UBC	ERBB4
UBC	EPT1
UBC	EPHB2
UBC	EPB41L5
UBC	EP400
UBC	ENY2
UBC	ENSA
UBC	ENO3
UBC	ENDOV
UBC	ENAH
UBC	EMC8
UBC	EMC4
UBC	EMC1

UBC	ELP6
UBC	ELP2
UBC	ELMO2
UBC	ELF1
UBC	EIF5A2
UBC	EIF5A
UBC	EIF4H
UBC	EIF4G3
UBC	EIF4E2
UBC	EIF4A1
UBC	EIF3L
UBC	EIF3J
UBC	EIF3I
UBC	EIF3F
UBC	EIF3A
UBC	EHMT2
UBC	EHMT1
UBC	EHD2
UBC	EHBP1L1
UBC	EFTUD2
UBC	EEF1G
UBC	EEF1B2
UBC	EEF1A1
UBC	EEA1
UBC	EDC3
UBC	ECH1
UBC	EAPP
UBC	DYNLL1
UBC	DYNC2H1
UBC	DYNC1I2
UBC	DUSP11
UBC	DTX3L
UBC	DTX1
UBC	DSP
UBC	DSN1
UBC	DSG1
UBC	DSC2
UBC	DSC1
UBC	DRAP1
UBC	DPP3
UBC	DPH2
UBC	DPH1
UBC	DOCK4
UBC	DOCK2

UBC	DNTT
UBC	DNM2
UBC	DNAJC7
UBC	DNAJB2
UBC	DNAJB12
UBC	DNAJB11
UBC	DNAJA4
UBC	DNAJA2
UBC	DLG1
UBC	DIS3
UBC	DIO2
UBC	DIEXF
UBC	DHX9
UBC	DHX36
UBC	DHX34
UBC	DHDDS
UBC	DGKA
UBC	DFFB
UBC	DDX50
UBC	DDX5
UBC	DDX47
UBC	DDX41
UBC	DDX39B
UBC	DDX39A
UBC	DDRGK1
UBC	DDR2
UBC	DCXR
UBC	DCUN1D5
UBC	DCP1B
UBC	DCLRE1A
UBC	DCAKD
UBC	DAZAP2
UBC	DAPK1
UBC	DAGLB
UBC	DAG1
UBC	DAD1
UBC	DAB2
UBC	CYHR1
UBC	CYFIP1
UBC	CYBA
UBC	CXCR4
UBC	CUL2
UBC	CUEDC2
UBC	CTH

UBC	CSTF3
UBC	CSPG4
UBC	CSNK1E
UBC	CSDE1
UBC	CRY1
UBC	CRNKL1
UBC	CREM
UBC	CPVL
UBC	CPT1A
UBC	CPSF7
UBC	CPSF3L
UBC	COX5A
UBC	COTL1
UBC	CORO1C
UBC	COPE
UBC	COPB1
UBC	COMMD1
UBC	COL1A1
UBC	COG4
UBC	CNOT2
UBC	CNNM3
UBC	CNNM2
UBC	CLSPN
UBC	CLPTM1L
UBC	CLPTM1
UBC	CLNS1A
UBC	CLINT1
UBC	CLEC16A
UBC	CLDND1
UBC	CLDN12
UBC	CHUK
UBC	CHTF18
UBC	CHPF
UBC	CHD8
UBC	CHD4
UBC	CHCHD2
UBC	CHAMP1
UBC	CHAC1
UBC	CERS2
UBC	CERS1
UBC	CEP95
UBC	CEP72
UBC	CEP44
UBC	CEP170

UBC	CENPN
UBC	CDK5RAP2
UBC	CDK18
UBC	CDK17
UBC	CDH3
UBC	CDCA2
UBC	CDC42BPB
UBC	CDC25A
UBC	CDC16
UBC	CDA
UBC	CD97
UBC	CD151
UBC	CCT8
UBC	CCT7
UBC	CCT4
UBC	CCNG1
UBC	CCNA2
UBC	CCDC58
UBC	CCDC47
UBC	CCDC14
UBC	CCDC115
UBC	CBWD1
UBC	CBS
UBC	CBLB
UBC	CBFB
UBC	CAPN2
UBC	CAP2
UBC	CAP1
UBC	CAND2
UBC	CAMLG
UBC	CALR
UBC	CA8
UBC	CA2
UBC	C6orf211
UBC	C6orf106
UBC	C3orf17
UBC	C2CD5
UBC	C16orf45
UBC	C15orf39
UBC	C11orf30
UBC	BUB1B
UBC	BTN3A3
UBC	BTG3
UBC	BTBD9

UBC	BTBD2
UBC	BTBD10
UBC	BTBD1
UBC	BRIP1
UBC	BRD7
UBC	BRAT1
UBC	BOP1
UBC	BOD1L1
UBC	BNIP3
UBC	BMPR1A
UBC	BLZF1
UBC	BLVRB
UBC	BLVRA
UBC	BLM
UBC	BIRC6
UBC	BIRC2
UBC	BCL2L13
UBC	BCL2L10
UBC	BCL10
UBC	BAMBI
UBC	BAIAP2
UBC	BAG6
UBC	B3GNT2
UBC	AXL
UBC	AVEN
UBC	ATXN3
UBC	ATP6V1F
UBC	ATP6V1B2
UBC	ATP5H
UBC	ATP2B2
UBC	ATP2B1
UBC	ATP2A3
UBC	ATOX1
UBC	ATM
UBC	ATG9A
UBC	ATG4C
UBC	ATF1
UBC	ASPSCR1
UBC	ASPM
UBC	ASF1B
UBC	ASCC2
UBC	ARPP19
UBC	ARPC1A
UBC	ARL8B

UBC	ARL8A
UBC	ARL2
UBC	ARID5B
UBC	ARID1A
UBC	ARHGEF7
UBC	ARHGEF40
UBC	ARHGAP29
UBC	ARHGAP1
UBC	ARG1
UBC	ARF6
UBC	ARF5
UBC	ARF4
UBC	ARAP1
UBC	APOBEC3B
UBC	APOB
UBC	APC
UBC	APBB2
UBC	APAF1
UBC	AP3S1
UBC	AP2S1
UBC	AP2M1
UBC	AP2A1
UBC	AP1M1
UBC	AP1G1
UBC	AP1B1
UBC	ANXA7
UBC	ANTXR2
UBC	ANTXR1
UBC	ANP32E
UBC	ANAPC5
UBC	ANAPC4
UBC	ANAPC1
UBC	AMPD3
UBC	AMIGO2
UBC	AMFR
UBC	ALYREF
UBC	ALMS1
UBC	ALG9
UBC	ALG2
UBC	ALG10
UBC	ALDOB
UBC	ALDOA
UBC	ALDH3A2
UBC	ALDH1B1

UBC	ALDH1A2
UBC	ALDH16A1
UBC	ALCAM
UBC	AKAP11
UBC	AGO3
UBC	AGK
UBC	AGFG1
UBC	AFF1
UBC	AES
UBC	ADIPOR1
UBC	ADI1
UBC	ADD1
UBC	ADCY5
UBC	ADCY4
UBC	ADAM9
UBC	ADA
UBC	ACTR1A
UBC	ACTL6B
UBC	ACTL6A
UBC	ACTG1
UBC	ACIN1
UBC	ABTB2
UBC	ABLIM1
UBC	ABI2
UBC	ABHD17A
UBC	ABCF1
UBC	ABCE1
UBC	ABCD3
UBC	ABCA1
UBC	AARS
UBC	AAAS
UCHL3	UBB
UBC	UBB
TRAF6	UBB
SNCAIP	UBB
RNF168	UBB
RNF11	UBB
NEDD8	UBB
H2AFX	UBB
DAZAP2	UBB
CDKN1B	UBB
CDC6	UBB
CDC25A	UBB
UBC	UBASH3B

EGFR	UBASH3B
UBC	UBASH3A
EGFR	UBASH3A
CBL	UBASH3A
VCL	UBAP2L
UBC	UBAP2L
PLXNA2	UBAP2L
VPS28	UBAP1
UBC	UBAP1
TSG101	UBAP1
UBC	UBAC2
LMO7	UBAC2
GET4	UBAC2
UBC	UBAC1
RNF11	UBAC1
LAPTM5	UBAC1
DAZAP2	UBAC1
CPSF6	UBAC1
UBC	UBA6
YWHAG	UBA52
UBA52	UBQLN1
UBE2D3	UBA52
UBE2D2	UBA52
UBE2B	UBA52
UBC	UBA52
TRAF6	UBA52
TRAF2	UBA52
TP53	UBA52
TNFRSF1B	UBA52
TNFRSF1A	UBA52
TGFBR1	UBA52
TAB2	UBA52
UBA52	STIP1
SQSTM1	UBA52
SMAD4	UBA52
SMAD2	UBA52
SMAD1	UBA52
RNF5	UBA52
UBA52	RBCK1
OTUB1	UBA52
NFKBIA	UBA52
UBA52	HIST3H2A
UBA52	CHEK2
UBA52	CFL1

ACVR1	UBA52
UBC	UBA5
GABARAPL2	UBA5
UBE2M	UBA3
UBC	UBA3
NEDD8	UBA3
NAE1	UBA3
CHD3	UBA3
UBC	UBA2
SUMO1	UBA2
UBA2	RAD23B
UBC	U2SURP
TBC1D4	U2SURP
SUMO2	U2SURP
SF3A2	U2SURP
WT1	U2AF2
VCL	U2AF2
UBC	U2AF2
THAP1	U2AF2
SUGP1	U2AF2
SRPK2	U2AF2
SMAD5	U2AF2
SFPQ	U2AF2
SCAF11	U2AF2
RBM39	U2AF2
LURAP1	U2AF2
GEMIN2	U2AF2
DPPA2	U2AF2
UBC	U2AF1L4
U2AF1	VCL
UBC	U2AF1
U2AF1	U2AF2
SRSF2	U2AF1
U2AF1	SRPK2
U2AF1	SF3A2
UBC	TYW1B
UBC	TYW1
KIR2DL3	TYROBP
DCT	TYR
UBC	TYMS
VAV1	TYK2
UBC	TYK2
STAT1	TYK2
STAM2	TYK2

PLAUR	TYK2
JAK1	TYK2
IL6ST	TYK2
IFNAR1	TYK2
GHR	TYK2
UBC	TXNRD1
TXNIP	TXNRD1
PPP1R16A	TXNRD1
APEX1	TXNRD1
UCHL5	TXNL1
UBC	TXNL1
PSMD4	TXNL1
PSMD14	TXNL1
UBC	TXNIP
SMURF2	TXNIP
UBC	TXNDC9
IKBKAP	TXNDC9
UBC	TXNDC17
UBC	TXNDC12
KRT15	TXNDC11
UBC	TXN2
UBC	TXN
TXNIP	TXN
NR3C1	TXN
NCF4	TXN
MAP3K5	TXN
COPS5	TXN
APEX1	TXN
UBC	TXLNA
ERBB3	TXK
BMP4	TWSG1
BMP1	TWSG1
SREBF1	TWIST2
TP53	TWIST1
TCF3	TWIST1
MTA2	TWIST1
KAT2B	TWIST1
GLI3	TWIST1
UBC	TWF1
VCL	TVP23C-CDRT4
UBC	TVP23C-CDRT4
UBC	TVP23B
UBC	TUT1
PIN1	TUT1

UBC	TULP3
UBC	TUFM
TNFRSF1B	TUFM
UBC	TUBGCP4
TUBG1	TUBGCP4
SDSL	TUBGCP4
FBF1	TUBGCP4
ADAMTSL4	TUBGCP4
UBC	TUBGCP3
UBC	TUBGCP2
NFKB2	TUBGCP2
UBC	TUBG1
TUBG1	TUBGCP3
TUBG1	RNF19A
TUBG1	RASSF1
RACGAP1	TUBG1
PXN	TUBG1
PIK3R1	TUBG1
PARK2	TUBG1
TUBG1	NUP85
MARK4	TUBG1
BRCA1	TUBG1
UBC	TUBD1
TNFRSF1A	TUBB6
VCL	TUBB4B
UBC	TUBB4B
TUBB2A	TUBB4B
TUBB	TUBB4B
PXN	TUBB4B
DYNLL1	TUBB4B
CDK9	TUBB4B
CDK2	TUBB4B
UBC	TUBB4A
TRAF6	TUBB4A
TRAF2	TUBB4A
TRAF1	TUBB4A
TNFRSF1B	TUBB4A
TNFRSF1A	TUBB4A
TBK1	TUBB4A
TAB2	TUBB4A
NFKB2	TUBB4A
NFKB1	TUBB4A
MAP3K14	TUBB4A
IKBKE	TUBB4A

CHUK	TUBB4A
UBC	TUBB2B
TRAF6	TUBB2B
TNFRSF1B	TUBB2B
TNFRSF1A	TUBB2B
TAB1	TUBB2B
NFKB2	TUBB2B
NFKB1	TUBB2B
MAP3K14	TUBB2B
UBC	TUBB2A
TUBB	TUBB2A
TK1	TUBB2A
RASSF1	TUBB2A
TUBB2A	NPHP1
TUBB2A	NCALD
TUBB2A	MAP1A
UBC	TUBB1
ESR1	TUBB1
TUBB	TNFRSF1A
UBC	TUBAL3
ZAP70	TUBA4A
UBC	TUBA4A
TUBB4B	TUBA4A
TUBB2A	TUBA4A
TUBB	TUBA4A
TUBA1C	TUBA4A
TUBA1A	TUBA4A
TBCE	TUBA4A
TBCB	TUBA4A
SIAH1	TUBA4A
RACGAP1	TUBA4A
PIGK	TUBA4A
MYC	TUBA4A
MAP1A	TUBA4A
GRM7	TUBA4A
FYN	TUBA4A
EGFR	TUBA4A
DCTN1	TUBA4A
CALB2	TUBA4A
UBC	TUBA1C
SUMO1	TUBA1C
VHL	TUBA1A
UBC	TUBA1A
TUBB6	TUBA1A

TUBB	TUBA1A
PXN	TUBA1A
NCALD	TUBA1A
MYC	TUBA1A
TUBA1A	MAP2
LCK	TUB
UBC	TTYH3
UBC	TTYH2
VIM	TTR
ST3GAL3	TTR
RBP4	TTR
PHYHIP	TTR
IKBKAP	TTR
HNRNPH3	TTR
FEZ1	TTR
DDR1	TTR
CHD3	TTR
ATF4	TTR
APP	TTR
VAV2	TTN
UBC	TTN
TRIM63	TTN
TCAP	TTN
OBSL1	TTN
NEB	TTN
MYBPC3	TTN
CAPN3	TTN
CALM1	TTN
ANKRD1	TTN
ACTN1	TTN
ACTA1	TTN
UBC	TTLL5
UBC	TTLL12
RUVBL2	TTI2
UBC	TTI1
TRRAP	TTI1
TELO2	TTI1
SMG1	TTI1
RUVBL2	TTI1
RUVBL1	TTI1
PRKDC	TTI1
POLR2E	TTI1
PIH1D1	TTI1
MTOR	TTI1

HSPA4 TTI1
ATRTTI1
UBC TTF2
TACC3 TTF2
SMAD1 TTF2
SKIL TTF2
PDCD6IP TTF2
MYB TTF2
HNRNPA2B1TTF2
DRAP1 TTF2
DCP1A TTF2
KAT2B TTF1
UBC TTC9C
UBC TTC7A
UBC TTC4
HSP90AA1 TTC4
UBC TTC37
UBC TTC28
UBC TTC27
UBC TTC21B
UBC TTC19
UBC TTC17
ACVR1 TTC17
CCDC85B TTC14
UBC TTC12
UBC TTC1
GNAQ TTC1
GNA15 TTC1
UBC TTBK2
UBC TSTD2
UBC TSTA3
PRSS1 TST
TGFBR1TSSK4
CREB1 TSSK4
SMAD4 TSSK3
SPTAN1 TSSC4
UBC TSR3
UBC TSR1
SUMO2 TSR1
RIOK2 TSR1
PNO1 TSR1
MAP3K14 TSR1
BYSL TSR1
UBC TSPYL5

UBC TSPYL4
NINL TSPYL4
CCDC85B TSPYL4
UBC TSPYL1
HAP1 TSPYL1
ITGB1 TSPAN4
ITGA3 TSPAN4
UBC TSPAN3
UBC TSPAN14
UBC TSNAX
TSNAXIP1 TSNAX
TSNTSNAX
MRFAP1L1 TSNAX
C1DTSNAX
UBC TSN
SKIL TSKU
TSSK2 TSKS
GNAS TSHR
GNAQ TSHR
FN1 TSHR
CANX TSHR
TSG101 VPS37B
VPS28 TSG101
UBC TSG101
TP53 TSG101
TSG101 SNF8
TSG101 PPFIBP2
TSG101 PDCD6IP
MDM2 TSG101
KRT15 TSG101
TSG101 ALG2
UBC TSEN54
UBC TSEN34
LMO4 TSEN15
TSC22D4 ZNF580
TSC22D4 ABLIM1
UBC TSC22D2
UBC TSC22D1
SETDB1 TSC22D1
RGS1 TSC22D1
MAP3K12 TSC22D1
CCDC90B TSC22D1
APLP1 TSC22D1
DTNB TSACC

TRRAP	TELO2
S100A10	TRPV6
UBC	TRPV4
TRPV4	MAP7
TRPV4	HCK
TRPV4	FYN
UBC	TRPV2
TRAF2	TRPT1
UBC	TRPM4
TRPC3	TRPC6
ITPR3	TRPC6
FYN	TRPC6
TNFRSF1A	TRPC4AP
TRPC1	TRPC4
ITPR3	TRPC4
ITPR2	TRPC4
TRPC3	TRPC1
TRPC1	STIM1
TRPC1	ITPR2
UBC	TROVE2
PUF60	TROVE2
UBC	TROAP
BYSL	TROAP
UBC	TRMT61A
TRMT6	TRMT61A
UBC	TRMT6
UBC	TRMT5
UBC	TRMT44
UBC	TRMT112
UBC	TRMT10C
UBC	TRMT1
VCL	TRIP6
UBC	TRIP6
SRC	TRIP6
SMAD1	TRIP6
PTPN13	TRIP6
PTK2	TRIP6
PRKAA2	TRIP6
PPP1R16A	TRIP6
ODF1	TRIP6
NUP210	TRIP6
NCK2	TRIP6
LPAR2	TRIP6
KIAA1683	TRIP6

HOXA9	TRIP6
HOXA1	TRIP6
FHL3	TRIP6
FAM46C	TRIP6
FAM124B	TRIP6
CATSPER1	TRIP6
C5orf55	TRIP6
BCAR1	TRIP6
AQP1	TRIP6
TRIP4	THRB
UBC	TRIP13
TRIP13	STAMBPL1
TRIP13	SIGLEC5
TRIP13	SEC24A
TRIP13	PLSCR4
TRIP13	GPRIN2
TRIP13	GLYCTK
TRIP13	CPSF3L
TRIP13	CARD14
UBC	TRIP12
TRIP12	THRB
SMAD9	TRIP12
TRIP12	CLSTN1
UBC	TRIP11
WBP11	TRIP10
UBC	TRIP10
RXRA	TRIP10
DNM2	TRIP10
CDC42	TRIP10
VIM	TRIOBP
USP7	TRIM8
UBE2D2	TRIM8
UBC	TRIM6-TRIM34
UBC	TRIM68
UBC	TRIM65
PSMD4	TRIM63
TRIM63	MYOZ1
SMAD2	TRIM62
SKIL	TRIM62
TRIM5	TRIM6
UBC	TRIM59
UBE2D3	TRIM55
TRIM63	TRIM55
TRIM63	TRIM54

UBC	TRIM5
TRIM5	TRIM34
AK5	TRIM46
UBC	TRIM44
UBC	TRIM4
UBC	TRIM39-RPP21
TRIM39	UBTD1
TRIM39	UBE2E2
UBE2E1	TRIM39
TRIM39	UBE2D4
UBE2D3	TRIM39
UBE2D2	TRIM39
UBC	TRIM39
TRIM17	TRIM39
TRIM39	PLCD4
KDM1A	TRIM39
DCUN1D1	TRIM39
UBC	TRIM38
RIPK1	TRIM38
HSPA1A	TRIM38
TRIM37	ZNF655
UBC	TRIM37
TRIM37	TRAF6
TRIM37	TRAF5
TRAF2	TRIM37
TRIM37	TRAF1
TRIM37	TCEB3B
TRIM37	RIBC2
TRIM37	RHPN1
TRIM37	PNKP
TRIM37	PBK
TRIM37	NUDT18
TRIM37	MCRS1
FXR2	TRIM37
TRIM37	FAM124B
TRIM37	FAM107A
TRIM37	DLGAP5
TRIM37	COPB1
TRIM37	BYSL
UBC	TRIM34
SMAD2	TRIM33
TRIM32	UBQLN1
UBE2N	TRIM32
UBE2E1	TRIM32

UBE2D3 TRIM32
UBE2D2 TRIM32
UBC TRIM32
TRIM27 TRIM32
SFN TRIM32
TRIM32 PDE9A
UBE2K TRIM3
MYO5A TRIM3
ACTN4 TRIM3
TRIM29 TSGA10
SMAD9 TRIM29
LZTS2 TRIM29
GCC1 TRIM29
FXR2 TRIM29
TRIM29 EFCAB4B
CEP70 TRIM29
TRIM28 STAT1
TRIM28 MDM2
TRIM27 KCTD15
TRIM27 FXYD6-FXYD2
UBC TRIM26
KIF5A TRIM26
UBE2L6 TRIM25
UBE2D3 TRIM25
UBC TRIM25
SFN TRIM25
TRIM25 PITX2
TRIM25 DDX58
UBC TRIM24
TRIM28 TRIM24
TP53 TRIM24
TRIM24 TAF7
RXRA TRIM24
TRIM24 RARA
TRIM23 ZNF250
TRIM23 UBE2E1
UBC TRIM23
TRIM23 TRIM27
TRIM23 SF1
TRIM23 GEM
UBC TRIM22
YWHAG TRIM21
USP4 TRIM21
UBE2D2 TRIM21

UBE2D1	TRIM21
UBD	TRIM21
UBC	TRIM21
TROVE2	TRIM21
NIF3L1	TRIM21
NCOA3	TRIM21
IRF3	TRIM21
UBE2D3	TRIM17
UBC	TRIM17
UBC	TRIM14
UBC	TRIM13
RBM48	TRIB3
EEF1G	TRIB3
ATF5	TRIB3
TRHR	TRH
UBC	TRERF1
RYR1	TRDN
HRC	TRDN
CASQ2	TRDN
ASPH	TRDN
STX1A	TRDMT1
RBM48	TRDMT1
TRDMT1	DDX24
COPS6	TRDMT1
LCK	TRAT1
CD247	TRAT1
UBC	TRAPPC9
UBC	TRAPPC6B
UBC	TRAPPC5
UBC	TRAPPC3
TRAPPC8	TRAPPC3
TRAPPC3	TRAPPC6B
TRAPPC3	TRAPPC6A
TRAPPC3	TRAPPC5
TRAPPC3	TRAPPC2L
TRAPPC1	TRAPPC3
UBC	TRAPPC2L
TRAPPC8	TRAPPC2L
UBC	TRAPPC12
UBC	TRAPPC11
TRAPPC3	TRAPPC11
UBC	TRAP1
TNFRSF1A	TRAP1
EXT2	TRAP1

EXT1	TRAP1
UBC	TRAM1
GABRB2	TRAK2
SKIL	TRAK1
RHOT1	TRAK1
TRAF6	ZRANB1
TRAF6	VCP
TRAF6	USP2
TRAF6	UCHL1
UBE2N	TRAF6
TRAF6	UBE2L3
TRAF6	UBE2D3
UBC	TRAF6
TRAF6	TUBB6
TRAF6	TUBB
TRAF2	TRAF6
TRAF6	TRAF1
TRAF6	TNFRSF1B
TRAF6	TNFRSF13B
TRAF6	TNFAIP3
TRAF6	TAB2
TRAF6	STUB1
TRAF6	STRADB
TRAF6	RNF126
TRAF6	PSMD7
TRAF6	PSMD4
TRAF6	PSMD3
TRAF6	PSMD13
TRAF6	PSMD11
TRAF6	PSMD1
TRAF6	PSMC3
TRAF6	PSMC2
TRAF6	PSEN1
TRAF6	PELI1
TRAF6	OTUD7B
TRAF6	OTUB1
TRAF6	MYD88
TRAF6	MAP3K5
TRAF6	MAP3K14
TRAF6	IRAK4
TRAF6	IPO7
TRAF6	IL17RB
TRAF6	HSPA8
TRAF6	HSPA1L

TRAF6	HADHA
TRAF6	FANCD2
TRAF6	CD40
TRAF6	BIRC2
USP7	TRAF5
UBC	TRAF5
TRAF6	TRAF5
TRAF2	TRAF5
TRAF5	TNFRSF8
TRAF5	TNFRSF4
TRAF5	TNFRSF19
TRAF5	TNFRSF14
TRAF5	TNFRSF13B
TRAF5	TNFRSF11A
TDP2	TRAF5
RIPK2	TRAF5
RIPK1	TRAF5
TRAF5	NUMBL
MAP3K5	TRAF5
LTBR	TRAF5
TRAF5	CD40
TRAF5	CD27
UBC	TRAF3IP2
TRAF6	TRAF3IP2
TRAF2	TRAF3IP2
CHUK	TRAF3IP2
CD40	TRAF3IP2
USP7	TRAF3
USO1	TRAF3
UBC	TRAF3
TRIM37	TRAF3
TRAIP	TRAF3
TRAF5	TRAF3
TRAF3IP2	TRAF3
TRAF3IP1	TRAF3
TRAF2	TRAF3
TNFRSF9	TRAF3
TNFRSF8	TRAF3
TNFRSF4	TRAF3
TNFRSF18	TRAF3
TNFRSF14	TRAF3
TNFRSF13C	TRAF3
TNFRSF11A	TRAF3
TICAM1	TRAF3

TBK1	TRAF3
TANK	TRAF3
SRCTRAF3	
RIPK1	TRAF3
NFKB2	TRAF3
MAVS	TRAF3
MAP3K14	TRAF3
MAP2K1	TRAF3
LTBR	TRAF3
IKBKE	TRAF3
CD40	TRAF3
CD27	TRAF3
CASP3	TRAF3
CAP1	TRAF3
BIRC3	TRAF3
BIRC2	TRAF3
ACTA1	TRAF3
TRAF2	USP53
TRAF2	USP2
UBE2N	TRAF2
UBC	TRAF2
TRAF2	TUBB
TRAF2	TRPC4AP
TRAF2	TRIM31
TRAF2	TRAIP
TRAF2	TRAF1
TRAF2	TNIK
TRAF2	TNFRSF9
TRAF2	TNFRSF1B
TRAF2	TNFRSF1A
TRAF2	TNFRSF18
TRAF2	TNFRSF14
TRAF2	TNFRSF13B
TRAF2	TNFRSF12A
TRAF2	TNFRSF10B
TRAF2	TNFAIP3
TRAF2	TCEB3B
TRAF2	TCEA2
TRAF2	TBK1
TRAF2	TANK
TRAF2	TAB2
TRAF2	STUB1
TRAF2	SPHK1
TRAF2	SOX30

TRAF2	RUVBL2
TRAF2	PIN1
TRAF2	NUDT18
TRAF2	MAP3K5
TRAF2	MAP3K14
TRAF2	LTBR
TRAF2	KANSL1
TRAF2	ITPK1
TRAF2	HSPA8
TRAF2	FBXO28
TRAF2	FADD
EGFR	TRAF2
TRAF2	DTNB
TRAF2	CRY1
TRAF2	CHUK
TRAF2	CD40
TRAF2	CD27
TRAF2	BIRC2
TRAF2	BCL10
TRAF2	BANP
TRAF2	ABLIM1
TRAF2	AASDHPPT
TUBB	TRAF1
TRAF1	TRAIP
TRAF1	TNFRSF9
TNFRSF1A	TRAF1
TRAF1	TANK
SFPQ	TRAF1
TRAF1	DDX5
UBC	TRABD2B
UBC	TRABD
YWHAG	TRA2B
UBC	TRA2B
SRSF3	TRA2B
RNPS1	TRA2B
NGFR	TRA2B
LUM	TRA2B
IVNS1ABP	TRA2B
UBC	TRA2A
UBC	TPT1
TP53	TPT1
UBC	TPRKB
OSGEP	TPRKB
UBC	TPRA1

UBC	TPR
NUP98	TPR
UBC	TPP2
UBC	TPM4
UBC	TPM3
RIPK3	TPM3
IKBKE	TPM3
HSP90AA1	TPM3
CNN1	TPM3
CHUK	TPM3
ACTA1	TPM3
UBC	TPM2
TPM1	TPM2
SKIL	TPM2
UBC	TPM1
CNN1	TPM1
UBD	TPI1
UBC	TPI1
SETDB1	TPI1
LIPT1	TPI1
DDX24	TPI1
CCL11	TPI1
TPH1	HTT
UBC	TPGS2
UBC	TPGS1
YAE1D1	TPD52L1
TPD52L2	TPD52L1
GPR135	TPD52L1
EIF3J	TPD52L1
UBC	TPD52
TPD52L2	TPD52
TPD52L1	TPD52
YAP1	TP73
UBC	TP73
TP63	TP73
TP53	TP73
PIN1	TP73
NFYB	TP73
MYC	TP73
MDM2	TP73
ITCH	TP73
HIPK2	TP73
CHUK	TP73
SMAD2	TP63

MDM2	TP63
UBC	TP53RK
TPRKB	TP53RK
OSGEP	TP53RK
HSP90AA1	TP53RK
CALU	TP53RK
CALCOCO2	TP53RK
C14orf142	TP53RK
TP53	TP53INP1
HIPK2	TP53INP1
UBC	TP53I11
UBC	TP53BP2
TP53	TP53BP2
PPP1CA	TP53BP2
TP53BP2	NAE1
TP53BP2	KIF5A
TP53BP2	BCL2L2
TP53BP2	BCL2L1
USP28	TP53BP1
UBC	TP53BP1
TP53	TP53BP1
RPA1	TP53BP1
RNF8	TP53BP1
RNF168	TP53BP1
PAXIP1	TP53BP1
NEK1	TP53BP1
MDC1	TP53BP1
HIST4H4	TP53BP1
HIST1H3A	TP53BP1
ATM	TP53BP1
TP53	YBX1
TP53	WT1
TP53	WRN
TP53	UCHL1
UBE2N	TP53
TP53	TRRAP
TRIM28	TP53
TP53	TP63
TP53	THRB
TP53	THAP8
TP53	TEP1
TP53	TAF9
TP53	SULT1E1
TP53	STX5

SMAD2	TP53
TP53	SERPINB9
TP53	S100B
TP53	RPA1
TP53	RFWD2
TP53	RFFL
TP53	RAD51
TP53	RAB4A
TP53	PTTG1
TP53	PTEN
TP53	PSMD11
TP53	PRKCA
TP53	PRIM1
TP53	PLK1
TP53	PHB
TP53	PBK
TP53	MTA2
TP53	MPHOSPH6
TP53	MED1
TP53	MDM2
TP53	KPNA2
TP53	ING5
TP53	ING1
TP53	HTT
TP53	GSK3B
TP53	GNL3
TP53	FXYD6-FXYD2
TP53	EHMT1
TP53	DDX5
TP53	CSNK1E
TP53	COX17
TP53	CDC14B
TP53	CCNG1
TP53	CCNA2
BTBD2	TP53
TP53	BRD7
TP53	BANP
TP53	BAK1
TP53	ATM
ZNF512B	TOX4
HIPK3	TOX4
UBC	TOR4A
UBC	TOR3A
UBC	TOR1AIP2

COPS5	TOR1AIP2
UBC	TOR1AIP1
TOR1A	TOR1AIP1
PPP1CA	TOR1AIP1
LMNA	TOR1AIP1
UBC	TOR1A
TOR1A	TOR1AIP2
UBE2D3	TOPORS
UBE2D1	TOPORS
UBC	TOPORS
TP53	TOPORS
TOP1	TOPORS
SUMO2	TOPORS
SUMO1	TOPORS
SNIP1	TOPORS
SATB1	TOPORS
PLK1	TOPORS
UBC	TOPBP1
UBC	TOP3B
TOP3B	RECQL5
TOP3B	FBLN5
TOP3B	CHIC2
RPA1	TOP3A
RMI2	TOP3A
FANCM	TOP3A
BLM	TOP3A
XPO1	TOP2B
UBC	TOP2B
TP53	TOP2B
TOP2A	TOP2B
SUMO2	TOP2B
SUMO1	TOP2B
HDAC1	TOP2B
ESR1	TOP2B
CSNK2B	TOP2B
BAZ1B	TOP2B
YWHAE	TOP2A
UBC	TOP2A
TP53	TOP2A
SUMO1	TOP2A
TOP2A	SNRPA
PRKCG	TOP2A
PRKCB	TOP2A
PRKCA	TOP2A

PIN1	TOP2A
HDAC2	TOP2A
HDAC1	TOP2A
DHX9	TOP2A
TOP2A	DFFB
TOP2A	COPS5
UBC	TOP1
TP53	TOP1
TOP1	SUPT16H
TOP1	SRSF11
SFPQ	TOP1
HNRNPC	TOP1
BTBD2	TOP1
TOP1	BTBD1
UBC	TONSL
SUPT16H	TONSL
SSRP1	TONSL
MMS22L	TONSL
MCM6	TONSL
MCM4	TONSL
MCM2	TONSL
ASF1B	TONSL
ASF1A	TONSL
UBC	TOMM70A
TOMM40	TOMM70A
TOMM7	TOMM40
UBC	TOMM34
TOMM7	TOMM22
TOMM40	TOMM22
UBC	TOMM20
TOMM20	TOMM40
TOMM20	TOMM22
TOMM20	PTN
TOMM20	PPHLN1
TOMM20	PINK1
HAP1	TOMM20
TOMM20	FEZ1
TOMM20	DHFR
TOMM20	BCL2
BAG6	TOMM20
TOMM20	APOA1
UBC	TOM1L2
TOLLIP	TOM1L2
UBC	TOM1L1

TOLLIP TOM1L1
TOM1L1PHYH
TOM1L1NOMO1
FYN TOM1L1
TOM1L1FAM208A
TOM1L1CCDC47
ZNF512B TOM1
ZFYVE16 TOM1
UBC TOM1
TOLLIP TOM1
UBC TOLLIP
TOLLIP IL1RAP
DAZAP2 TOLLIP
PABPC1 TOB2
CNOT7 TOB2
SMAD5 TOB1
SMAD1 TOB1
SCMH1 TOB1
CNOT7 TOB1
UBC TNS4
ERBB3 TNS4
EGFR TNS4
NPHP1 TNS1
CAPN2 TNS1
CNOT7 TNRC6C
CNOT2 TNRC6C
AGO3 TNRC6C
AGO1 TNRC6C
UBC TNRC6B
AGO4 TNRC6B
AGO3 TNRC6B
AGO1 TNRC6B
UBC TNRC6A
PABPC1 TNRC6A
AGO4 TNRC6A
AGO3 TNRC6A
AGO2 TNRC6A
AGO1 TNRC6A
UBC TNRC18
VCAN TNR
SIGLEC1 TNR
UBC TNPO3
SMURF2 TNPO3
TNPO2 NXF1

UBC	TNPO1
RPS7	TNPO1
NXF1	TNPO1
NUP98	TNPO1
HIST3H3	TNPO1
SNUPN	TNNT3
HAP1	TNNT3
TNNI3	TNNT2
TNNC1	TNNT2
PRKCA	TNNT2
ZMYND19	TNNT1
TRIM63	TNNT1
TPM1	TNNT1
TNNC1	TNNT1
PSMC5	TNNT1
PPFIA1	TNNT1
PNMA1	TNNT1
PLEKHF1	TNNT1
NINL	TNNT1
FXR2	TNNT1
CCDC85B	TNNT1
PRKCA	TNNI3
CCDC85B	TNNI1
UBC	TNNC1
TNNI3	TNNC1
TNNC1	TNNI1
ZNF512B	TNKS2
UBC	TNKS2
TNKS	TNKS2
LNPEP	TNKS2
GRB14	TNKS2
UBC	TNKS1BP1
TNKS2	TNKS1BP1
TNKS	TNKS1BP1
UBC	TNKS
TNKS	POT1
NUMA1	TNKS
UBC	TNK1
UBC	TNIP3
UBC	TNIP2
TNFAIP3	TNIP2
NFKB2	TNIP2
MAP3K8	TNIP2
UBC	TNIP1

TNFAIP3 TNIP1
OPTN TNIP1
NFKB2 TNIP1
CCNG1 TNIP1
TRAF2 TNFSF9
TRAF1 TNFSF9
TNFRSF9 TNFSF9
ANG TNFSF8
TRAF2 TNFSF4
TNFRSF4 TNFSF4
TNFRSF18 TNFSF18
TNFRSF14 TNFSF14
LTBR TNFSF14
TNFSF12-TNFSF13 TNFSF13B
TNFRSF13B TNFSF13B
TNFRSF14 TNFSF13
TNFRSF13B TNFSF13
TNFRSF10D TNFSF10
TNFRSF10B TNFSF10
TNFRSF10A TNFSF10
RBM48 TNFSF10
FADD TNFSF10
TRAF2 TNFRSF8
TRAF1 TNFRSF8
TNFRSF8 TNFSF8
TNFSF15 TNFRSF6B
TNFSF14 TNFRSF6B
FASLG TNFRSF6B
TRAF2 TNFRSF4
TNFRSF1B XPNPEP3
VIM TNFRSF1B
UBC TNFRSF1B
TUBB TNFRSF1B
TRIP13 TNFRSF1B
TNFRSF1B TRAIP
TNFRSF1B TRAF1
TNFRSF1B TIMM50
TNFRSF1B TCP1
TNFRSF1B SLC25A3
TNFRSF1B SEC61A1
RPS3 TNFRSF1B
TNFRSF1B PFKP
TNFRSF1B MYH9
MYH10 TNFRSF1B

TNFRSF1B	MAD2L1
TNFRSF1B	LTA
TNFRSF1B	IPO8
TNFRSF1B	IPO7
HSPA8	TNFRSF1B
TNFRSF1B	HSPA6
HNRNPC	TNFRSF1B
TNFRSF1B	GCN1L1
TNFRSF1B	FANCD2
TNFRSF1B	EMC2
TNFRSF1B	COPB1
TNFRSF1B	ATAD3A
TRAF2	TNFRSF19
TRAF1	TNFRSF19
TNFRSF19	RTN4R
TRAF1	TNFRSF18
TRAF2	TNFRSF17
TRAF1	TNFRSF17
TNFSF13B	TNFRSF17
TNFSF12-TNFSF13	TNFRSF17
VIM	TNFRSF14
TRAF1	TNFRSF14
TNFRSF14	LTA
HDAC4	TNFRSF14
TNFRSF14	BTLA
TNFRSF13C	TNFSF13B
CAMLG	TNFRSF13B
TRAF1	TNFRSF12A
TNFSF12-TNFSF13	TNFRSF12A
TNFSF11	TNFRSF11B
TNFSF10	TNFRSF11B
TRAF6	TNFRSF11A
TRAF2	TNFRSF11A
TRAF1	TNFRSF11A
TNFRSF11A	TNFSF11
TAB2	TNFRSF11A
TNFRSF10C	TNFSF10
TNFRSF10C	FAM101B
TNFRSF10B	TNFRSF10A
BCL10	TNFRSF10A
MED4	TNFAIP8
UBC	TNFAIP3
TNFAIP3	TRAF1
TNFAIP3	TBK1

TNFAIP3	CHUK
UBC	TNFAIP1
CUL3	TNFAIP1
UBC	TNF
TRAF2	TNF
TNFRSF1B	TNF
TNFRSF1A	TNF
TNFAIP3	TNF
TAB2	TNF
LTBR	TNF
TNFLTA	
TNFDCN	
PXN	TNC
PTPRB	TNC
NCAN	TNC
ITGB6	TNC
ITGA5	TNC
FN1	TNC
EGFR	TNC
UBC	TMX3
UBC	TMX1
UBC	TMUB2
UBC	TMUB1
UBC	TMSB10
UBC	TMPRSS3
RXRA	TMPRSS3
SMARCA2	TMF1
FER	TMF1
UBC	TMEM97
UBC	TMEM9
UBC	TMEM70
UBC	TMEM66
UBC	TMEM64
UBC	TMEM63B
UBC	TMEM59L
UBC	TMEM59
UBC	TMEM57
UBC	TMEM55B
UBC	TMEM55A
UBC	TMEM50A
C7orf43	TMEM50A
UBC	TMEM45A
UBC	TMEM43
FDFT1	TMEM43

UBC	TMEM39B
UBC	TMEM39A
UBC	TMEM30A
UBC	TMEM259
UBC	TMEM246
UBC	TMEM237
UBC	TMEM230
UBC	TMEM223
UBC	TMEM222
PRSS23	TMEM222
ABHD16A	TMEM222
UBC	TMEM214
UBC	TMEM209
UBC	TMEM205
UBC	TMEM2
UBC	TMEM185B
UBC	TMEM183A
UBC	TMEM18
UBC	TMEM173
TBK1	TMEM173
UBC	TMEM170A
UBC	TMEM165
UBC	TMEM161A
UBC	TMEM147
ABI3	TMEM147
ABHD16A	TMEM147
SMURF2	TMEM139
UBC	TMEM132A
UBC	TMEM131
UBC	TMEM127
UBC	TMEM11
SCMH1	TMEM11
BNIP3L	TMEM11
BNIP3	TMEM11
UBC	TMEM109
UBC	TMEM106B
UBC	TMEM104
UBC	TMED9
TMED2	TMED9
TMED10	TMED9
UBC	TMED7-TICAM2
TLR4	TMED7-TICAM2
TICAM1	TMED7-TICAM2
UBC	TMED5

UBC	TMED4
UBC	TMED2
TMED2	TMED10
TMED2	F2RL1
UBC	TMED10
TMED10	COPZ1
UBQLN1	TMCO6
UBC	TMCO3
UBC	TMCO1
TMCC2	PTOV1
UBC	TMCC1
UBC	TMC6
UBC	TMBIM6
UBC	TM9SF3
SMAD4	TM9SF2
PXN	TM7SF2
UBC	TM4SF1
DNAJA1	TM4SF1
DDR1	TM4SF1
TLE1	TLX3
YWHAH	TLX2
PPP1CC	TLX1
MEIS1	TLX1
MYD88	TLR9
UBC	TLR4
TOLLIP	TLR4
TLR4	TLR1
TLR4	TIRAP
MYD88	TLR4
TLR4	LY96
TLR4	HSP90B1
TRAF6	TLR3
TICAM1	TLR3
TAB2	TLR3
PIK3R1	TLR3
MYD88	TLR3
TOLLIP	TLR2
TLR6	TLR2
TLR1	TLR2
TIRAP	TLR2
PIK3R1	TLR2
MYD88	TLR2
LY96	TLR2
HSP90B1	TLR2

CD14 TLR2
UBC TLN2
PIP5K1C TLN2
ITGB2 TLN1
UBC TLK2
ASF1B TLK2
ASF1A TLK2
UBC TLK1
ASF1B TLK1
ASF1A TLK1
UBC TLE4
UBC TLE3
SIX3 TLE3
FOXG1 TLE3
UBC TLE2
TLE1 TLE2
UBC TLE1
TLE1 SIX6
TLE1 SIX1
TLE1 FOXG1
TLE1 EN1
DAZAP2 TLE1
AESTLE1
TRIP13 TLDC1
NFIC TKTL2
UBC TKT
TALDO1TKT
UBC TK1
TP53 TK1
TK1TLE1
RBM48 TK1
TK1LRIF1
TK1GDF9
TK1FZR1
DRAP1 TK1
COPS6 TK1
TK1CCDC90B
C14orf1 TK1
OCLN TJP3
UBC TJP2
TJP2 TJP1
TJP1 KIRREL
UBC TIRAP
TRAF6 TIRAP

MYD88	TIRAP
IRAK4	TIRAP
TIRAP	EIF2AK2
UBC	TIPRL
PPP6C	TIPRL
PPP4C	TIPRL
UBC	TIPIN
TIMELESS	TIPIN
POT1	TINF2
LMO4	TINAGL1
MMP9	TIMP3
UBC	TIMM9
TIMM10	TIMM9
UBC	TIMM8B
TRAF1	TIMM50
TNFRSF1A	TIMM50
NFKB2	TIMM50
UBC	TIMM44
TIMM17A	TIMM44
UBC	TIMM23
TIMM50	TIMM23
TIMM17A	TIMM23
HAP1	TIMM17A
UBC	TIMM13
UBC	TIMM10
UBC	TIGD5
UBC	TIFA
TRAF2	TIFA
NUDT18	TIFA
AP1M1	TIFA
UBC	TICRR
UBC	TICAM1
TRAF6	TICAM1
TRAF2	TICAM1
TRAF1	TICAM1
TBK1	TICAM1
RIPK1	TICAM1
TIAM1	PRKCD
TIAM1	PRKCA
TIAM1	PARD3
MYC	TIAM1
JAK3	TIAF1
UBC	THYN1
LCK	THY1

UBC	THUMPD3
UBC	THTPA
SKIL	THSD7A
ASAP2	THSD7A
NCOR2	THRB
MED1	THRB
UBC	THRAP3
THRAP3	SFPQ
THRAP3	RNPS1
PIN1	THRAP3
THRAP3	NEDD4
TDG	THRA
RXRA	THRA
NCOR2	THRA
NCOA6	THRA
NCOA1	THRA
MED24	THRA
MED21	THRA
MED1	THRA
GRIP1	THRA
COPS2	THRA
CDK7	THRA
AKAP13	THRA
MPHOSPH6	THOP1
UBC	THOC7
DDX39B	THOC7
ALYREF	THOC7
UBC	THOC6
THOC5	THOC6
DDX39B	THOC6
ALYREF	THOC6
THOC5	THOC7
THOC3	THOC5
THOC1	THOC5
DDX39B	THOC5
ALYREF	THOC5
THOC3	THOC1
DDX39B	THOC3
ALYREF	THOC3
DDX39B	THOC1
ALYREF	THOC1
ZBTB16	THNSL2
ARPC3	THNSL2
UBC	THG1L

UBC	THEM6
THBS2	THBS3
MMP9	THBS2
THBS2	ITGA4
TNFAIP6	THBS1
TGFB1	THBS1
SPARC	THBS1
SCARB2	THBS1
PLG	THBS1
MMP9	THBS1
MMP2	THBS1
LAMB3	THBS1
ITGB3	THBS1
IGFBP5	THBS1
HRG	THBS1
FN1	THBS1
FGA	THBS1
F2	THBS1
DCN	THBS1
CTSG	THBS1
CORO1A	THBS1
COL5A1	THBS1
COL3A1	THBS1
COL2A1	THBS1
COL1A1	THBS1
CD47	THBS1
CD36	THBS1
SERPINE1	THBD
SERPINA5	THBD
F2	THBD
DYNLL1	THAP8
TRAF2	THAP7
TAF1B	THAP7
SET	THAP7
MDFI	THAP7
LURAP1	THAP7
EHMT2	THAP7
CCDC85B	THAP7
SKIL	THAP5
MOB4	THAP4
THAP1	RALYL
UBC	THADA
MAPKAPK2	TH
UBC	TGS1

NCOA6	TGS1
EP300	TGS1
CREBBP	TGS1
AP3M1	TGOLN2
AP2M1	TGOLN2
AP1M2	TGOLN2
AP1M1	TGOLN2
CALML5	TGM3
ZNF512B	TGM2
UBC	TGM2
SPARC	TGM2
SMAD2	TGM2
SERPINF2	TGM2
PXN	TGM2
PLCD1	TGM2
KPNA4	TGM2
HTT	TGM2
FN1	TGM2
SEMG2	TGM1
HDAC1	TGIF2-C20orf24
SMAD3	TGIF1
SMAD2	TGIF1
PML	TGIF1
MED31	TGIF1
HDAC1	TGIF1
CTBP1	TGIF1
UBC	TGFBRAP1
TGFBR2	TGFBRAP1
TGFBR1	TGFBRAP1
SMAD4	TGFBRAP1
SMAD2	TGFBRAP1
BMPR1A	TGFBRAP1
ALOX5	TGFBRAP1
ACVRL1	TGFBRAP1
ACVR2B	TGFBRAP1
TGFBR2	TGFBR3
TGFB3	TGFBR3
ACVRL1	TGFBR3
ACVR2A	TGFBR3
TGFBR2	TGFB3
TGFBR2	STRAP
WDR33	TGFBR1
TGFBR1	VEPH1
TGFBR1	UBE2E3

UBC	TGFBR1
TGFBR1	TTPAL
TGFBR1	TTC27
TGFBR1	TTC1
TGFBR1	TSSK1B
TGFBR1	TRAP1
TRAF6	TGFBR1
TGFBR1	TGFBR2
TGFBR1	TGFB3
TGFBR1	STYXL1
TGFBR1	STX8
TGFBR1	STUB1
TGFBR1	STRAP
TGFBR1	STK35
TGFBR1	SQSTM1
TGFBR1	SOCS6
TGFBR1	SNTG1
TGFBR1	SMURF2
SMAD4	TGFBR1
SMAD2	TGFBR1
SMAD1	TGFBR1
TGFBR1	SKAP2
TGFBR1	RRH
TGFBR1	RPAP3
TGFBR1	RNF5
TGFBR1	RNF130
TGFBR1	RHPN2
TGFBR1	RHOJ
TGFBR1	RHOBTB1
TGFBR1	RHEBL1
TGFBR1	RGS19
TGFBR1	RASL12
TGFBR1	RASD2
TGFBR1	RAP2A
TGFBR1	RAB6B
TGFBR1	RAB3B
TGFBR1	RAB33B
TGFBR1	PRPF4
TGFBR1	PPP6C
TGFBR1	PPP3CC
TGFBR1	PPP2R2B
TGFBR1	PLEKHB1
PIK3R1	TGFBR1
TGFBR1	OXSR1

TGFBR1	NUP37
TGFBR1	NRP1
TGFBR1	NEK6
TGFBR1	LIMS1
TGFBR1	KATNB1
TGFBR1	ITK
TGFBR1	ILKAP
TGFBR1	HSP90AA1
TGFBR1	GNB3
TGFBR1	GNB2
TGFBR1	FKBP1B
TGFBR1	ENC1
TGFBR1	EIF2AK1
TGFBR1	DAPK2
TGFBR1	DAB2
CLU	TGFBR1
TGFBR1	CHUK
TGFBR1	CHN2
TGFBR1	CDK6
TGFBR1	CDK4
CDK17	TGFBR1
TGFBR1	CDC42EP4
TGFBR1	CDC20
BTBD2	TGFBR1
TGFBR1	ARL8B
TGFBR1	ARHGAP31
TGFBR1	ARHGAP15
ANAPC5	TGFBR1
TGFBR1	ACVRL1
TGFBR1	ACVR1
FN1	TGFBI
COL4A2	TGFBI
COL4A1	TGFBI
COL1A2	TGFBI
COL1A1	TGFBI
A2M	TGFBI
VTN	TGFB2
TGFBR3	TGFB2
TGFBR2	TGFB2
TGFBR1	TGFB2
TGFB3	TGFB2
TGFB1	TGFB2
FMOD	TGFB2
BMP2	TGFB2

APP TGFB2
YWHAE TGFB1
TGFB1 VTN
UBC TGFB1
TGFB1 TGFBR3
TGFBR2 TGFB1
TGFBR1 TGFB1
TGFB1 MMP2
TGFB1 LTBP1
TGFB1 FNTA
DCN TGFB1
TGFB1 COL2A1
APP TGFB1
TGFB1 ACVRL1
NKD2 TGFA
GORASP1 TGFA
EGFR TGFA
UBC TGDS
HSPA5 TG
HSP90B1 TG
VCL TFRC
UBC TFRC
TF TFRC
RAB5B TFRC
OPTN TFRC
IKBKE TFRC
GABARAP TFRC
CD247 TFRC
UBC TFR2
UCHL5 TFPT
RUVBL2 TFPT
INO80E TFPT
INO80 TFPT
SMURF2 TFPI2
SKIL TFPI2
PRSS3 TFPI
F10 TFPI
UBC TFG
TRIM25 TFG
PLSCR1 TFG
PIN1 TFG
NFKB2 TFG
MED21 TFG
GRB2 TFG

GBP2	TFG
C14orf1	TFG
TACR3	TFF2
TACR2	TFF2
RBL1	TFDP2
RB1	TFDP2
E2F6	TFDP2
E2F3	TFDP2
E2F2	TFDP2
TP53	TFDP1
PXN	TFCP2
TFAM	TFB2M
UBC	TFAP4
TP53	TFAP2C
CITED4	TFAP2C
VPS11	TFAP2B
UBC	TFAP2B
SYT4	TFAP2B
CITED4	TFAP2B
YBX1	TFAP2A
WWP2	TFAP2A
UBC	TFAP2A
TP53	TFAP2A
SUMO2	TFAP2A
RB1	TFAP2A
LDLRAP1	TFAP2A
EPN1	TFAP2A
CITED2	TFAP2A
UBC	TFAM
TFAM	TFB1M
TF	IGFBP6
TF	IGFBP2
CCDC112	TEX9
TRIP13	TEX37
TBC1D4	TET2
UBC	TESC
VASP	TES
UBC	TES
TLN1	TES
ENAH	TES
UBC	TERT
TERT	TEP1
UBC	TERF2IP
TERF2IP	TINF2

TERF2IP	SLX4
RAF1	TERF2IP
TERF2IP	POT1
TERF2IP	NTRK1
TERF2IP	MRE11A
WRN	TERF2
UBC	TERF2
TERF2	TINF2
TERF2IP	TERF2
TERF2	POT1
TERF2	NBN
TERF2	MRE11A
TERF2	H2AFX
TERF2	GTF2A2
TERF2	DCLRE1B
TERF2	BLM
ZHX1	TERF1
ZBTB16	TERF1
YWHAG	TERF1
UBC	TERF1
TNKS2	TERF1
TNKS	TERF1
TINF2	TERF1
TERF2	TERF1
STUB1	TERF1
SMAD9	TERF1
SH3GL3	TERF1
RIC8A	TERF1
POT1	TERF1
PINX1	TERF1
PFN2	TERF1
NME2	TERF1
NME1	TERF1
MAPRE1	TERF1
GNL3	TERF1
FBXO4	TERF1
FANCD2	TERF1
DLG5	TERF1
CAPNS1	TERF1
ATM	TERF1
SMAD4	TENM3
UBC	TENM2
ERBB3	TENC1
OBFC1	TEN1

KRT15	TEKT4
LCK	TEK
GRB14	TEK
DOK2	TEK
HLFTEF
BATF3	TEF
UBC	TECR
VAV1	TEC
YAP1	TEAD4
WWTR1	TEAD3
UBC	TEAD3
YAP1	TEAD2
YAP1	TEAD1
UBC	TEAD1
SRF	TEAD1
MYH7	TEAD1
MEF2A	TEAD1
MAX	TEAD1
GABPB1	TDRD7
CDK17	TDRD7
FUS	TDRD3
UBC	TDP2
TRAF6	TDP2
TRAF2	TDP2
TNFRSF1B	TDP2
SUMO1	TDP2
SKIL	TDP2
TDP2	ETS1
TDP2	CD40
UBC	TDP1
SUMO2	TDP1
BAG6	TDGF1
ANG	TDGF1
UBC	TDG
SKIL	TDG
DDX39B	TDG
UBC	TCP11L1
MXD4	TCP10L
TBK1	TCP1
TCP1	PPP2R2C
TCP1	GBA
TCP1	CCT7
TNPO2	TCL1B
AKT2	TCL1B

UBC TCIRG1
VIM TCHP
NME7 TCHP
KRT15 TCHP
CCDC85B TCHP
UBC TCF7L2
PSEN1 TCF7L2
JUP TCF7L2
CTNNB1 TCF7L2
UBC TCF4
TCF3 TCF4
TAL1 TCF4
MYOD1 TCF4
ID3 TCF4
ID2 TCF4
DAXX TCF4
CTNNB1 TCF4
CALM1 TCF4
ASCL1 TCF4
TCF3 MSC
TCF3 ID3
TCF3 HAND1
UBC TCF25
LMO4 TCF21
UBC TCF20
SMAD1 TCF20
UBC TCF12
TCF3 TCF12
TAL1 TCF12
PTF1A TCF12
PRSS23 TCF12
HAND1 TCF12
CREBBP TCF12
UBC TCERG1
TCERG1U2AF2
TCERG1SFPQ
SF3B3 TCERG1
TCERG1SF3A3
TCERG1NUDT21
TCERG1HTT
FMNL1 TCERG1
TCEB2 TCEB3
TCEB1 TCEB3
NINL TCEB3

CUL5	TCEB3
TCEB1	ZER1
VHL	TCEB1
UBC	TCEB1
TCEB2	TCEB1
TCEB1	SPSB4
TCEB1	SPSB2
TCEB1	RAB40B
TCEB1	GPR75-ASB3
TCEB1	GNB2L1
CUL2	TCEB1
COPS6	TCEB1
TCEB1	ASB2
TCEB1	ASB1
UBR5	TCEA1
UBC	TCEA1
TBP	TCEA1
SF3B2	TCEA1
POLR2A	TCEA1
PAF1	TCEA1
GTF2H1	TCEA1
GTF2F1	TCEA1
GTF2E1	TCEA1
GTF2B	TCEA1
CDK8	TCEA1
TRIM63	TCAP
TCAP	MYOZ1
TCAP	MSTN
ATXN1	TCAP
FAM46A	TBX4
UBC	TBRG4
PTCD3	TBPL1
PLIN3	TBPL1
IRAK4	TBPL1
GTF2B	TBPL1
GTF2A2	TBPL1
ABCF3	TBPL1
TBP	ZNF76
UBC	TBP
TBP	TRIP4
TP53	TBP
TAF9	TBP
TBP	TAF7
TBP	TAF6

TBPTAF5
TBPTAF4
TBPTAF3
TBPTAF1D
TBPTAF1C
TBPTAF1B
TBPTAF12
TBPTAF11
TBPSUB1
TBPSPIB
TBPSPI1
RXRA TBP
RUVBL2 TBP
TBPREL
PSMC2 TBP
TBPPAX6
TBPNFYB
NCOA1 TBP
MYC TBP
TBPMSX1
MDM2 TBP
TBPMCM2
TBPJUND
TBPIKZF1
HTT TBP
TBPGTF2F2
TBPGTF2E2
TBPGTF2E1
GTF2B TBP
TBPGTF2A2
TBPFOXF2
FOSTBP
ESR1 TBP
DRAP1 TBP
DHX9 TBP
TBPCREM
TBPBRF2
UBC TBL3
SFNTBL3
APPBP2 TBL3
UBC TBL2
UBC TBL1XR1
HIST4H4 TBL1XR1
HIST2H2BE TBL1XR1

UBC	TBKBP1
TBK1	TBKBP1
TUBB	TBK1
TBK1	TANK
TBK1	SFPQ
RUVBL2	TBK1
TBK1	CCT7
XRCC6	TBCD
UBC	TBCD
TUBB2A	TBCD
TBCE	TBCD
SMAD9	TBCD
DEXI	TBCD
ARL2	TBCD
UBC	TBCB
UBC	TBCA
UBC	TBC1D7
TBC1D5	AP2M1
GABARAPL1	TBC1D2B
UBC	TBC1D23
ASCC2	TBC1D17
UBC	TBC1D15
UBC	TBC1D10B
YWHAG	TBC1D1
YWHAE	TBC1D1
TCEA2	TAX1BP3
RTKN	TAX1BP3
UBC	TAX1BP1
TRAF6	TAX1BP1
TNFAIP3	TAX1BP1
TBC1D17	TAX1BP1
UBC	TATDN1
UBC	TARSL2
UBC	TARS2
UBD	TARS
UBC	TARS
SARS	TARS
UBC	TARDBP
TARDBP	HNRNPA1
TARDBP	EXOSC10
EIF2AK2	TARBP2
UBC	TARBP1
UBC	TAPT1
UBC	TAPBP

TAP1	TAPBP
COPB1	TAPBP
CALR	TAPBP
UBC	TAP2
TAPBP	TAP2
TAP1	TAP2
HLA-G	TAP2
CALR	TAP2
TAP1	HLA-G
UBC	TAOK1
MARK1	TAOK1
MAP2K3	TAOK1
UBC	TANGO6
UBC	TALDO1
UBC	TAL1
TCF3	TAL1
STUB1	TAL1
TAL1	SSBP3
TAL1	SSBP2
TAL1	ELSPBP1
UBC	TAGLN2
ACTA1	TAGLN
RUVBL2	TAF9
RBM48	TAF9
TBP	TAF8
TAF6	TAF8
TAF5	TAF8
TAF4	TAF8
TAF9	TAF7
UBC	TAF6L
TAF9	TAF6L
STX11	TAF6L
KAT2B	TAF6L
USF1	TAF6
UBC	TAF6
TAF9	TAF6
TAF6	TAF12
HIST3H3	TAF6
UBC	TAF5L
KAT2B	TAF5L
TAF9	TAF5
TAF7	TAF5
TAF6	TAF5
TAF5	TAF4

TAF5	TAF3
TAF5	TAF12
TAF5	TAF11
HIST3H3	TAF5
TAF5	GTF2F2
TAF9	TAF4
TAF7	TAF4
HTT	TAF4
UBC	TAF2
TBP	TAF2
TAF9	TAF2
UBC	TAF1L
TBP	TAF1L
TAF1C	TAF1D
HAP1	TAF1D
UBC	TAF1C
UBC	TAF1B
TAF1B	TAF1D
TAF1C	TAF1B
HIST4H4	TAF1B
TAF1B	HIST2H2BE
TBP	TAF1A
TAF1D	TAF1A
TAF1B	TAF1A
SET	TAF1A
HIST4H4	TAF1A
UBC	TAF15
TBP	TAF13
TAF9	TAF13
TAF6	TAF13
TAF5	TAF13
TAF4	TAF13
TAF11	TAF13
TAF9	TAF12
TAF4	TAF12
TAF12	TAF11
TRRAP	TAF10
TBP	TAF10
TAF9	TAF10
TAF8	TAF10
TAF7	TAF10
TAF6L	TAF10
TAF6	TAF10
TAF5L	TAF10

TAF5	TAF10
TAF4	TAF10
TAF3	TAF10
TAF2	TAF10
TAF13	TAF10
TAF12	TAF10
SUPT7L	TAF10
SUPT3H	TAF10
SETD7	TAF10
SAP130	TAF10
KAT2B	TAF10
KAT2A	TAF10
FTL	TAF10
ATXN7	TAF10
TADA3	ZNF7
TP53	TADA3
TADA2A	TADA3
TADA3	SUPT3H
TADA3	SAP18
RXRA	TADA3
TADA3	RARA
TADA3	KAT2B
ESR1	TADA3
TADA3	EIF3J
TADA3	CCDC101
TADA3	ADPGK
UBC	TADA2B
TADA2A	KAT2B
TACR3	TAC1
TACR2	TAC1
TAC1	TACR1
UBC	TACC3
UBC	TACC1
SNRPG	TACC1
TACR3	TAC4
TACR1	TAC4
UBR1	TAC3
TACR3	TAC3
TACR2	TAC3
TAC1	TAC3
PTN	TAC3
IKBKAP	TAC3
FEZ1	TAC3
CCDC90B	TAC3

FN1	TAC1
BAG6	TAC1
ARHGEF40	TAC1
UBC	TAB2
TUBB	TAB2
TAB2	TIMM50
RUVBL2	TAB2
TAB2	NR2C2
HSPA8	TAB2
UBC	TAB1
TAB1	TUBB4A
TUBB	TAB1
TRAF6	TAB1
TRAF2	TAB1
TAB2	TAB1
RUVBL1	TAB1
MAPK14	TAB1
HSPA8	TAB1
TAB1	HSPA6
HSPA5	TAB1
TAB1	HSPA1L
TAB1	DNAJC7
VIMP	SYVN1
VCP	SYVN1
UBE2D3	SYVN1
UBC	SYVN1
TP53	SYVN1
SEL1L	SYVN1
OS9	SYVN1
HERPUD1	SYVN1
GPR37	SYVN1
ERLIN2	SYVN1
DERL2	SYVN1
DERL1	SYVN1
AMFR	SYVN1
SYT4	SYT1
SYT4	SYNCRIP
STX4	SYT4
STX1A	SYT4
SYT4	SGTA
TSC22D4	SYT17
NINL	SYT17
CHMP4A	SYT17
CCDC85B	SYT17

UBC	SYT11
CSNK1A1	SYT11
APPBP2	SYT11
UBC	SYT1
STON2	SYT1
SNAP25	SYT1
SYT1	NRXN1
SYT1	FGF1
CPLX1	SYT1
UBC	SYNRG
AP1G1	SYNRG
UBC	SYNPO
UBC	SYNM
GRB2	SYNM
DES	SYNM
AMPH	SYNM
UBC	SYNJ2BP
RALBP1	SYNJ2BP
ACVR2B	SYNJ2BP
ACVR2A	SYNJ2BP
ACTB	SYNJ2BP
SORBS2	SYNJ1
SH3GL3	SYNJ1
SH3GL2	SYNJ1
SH3GL1	SYNJ1
UBC	SYNGR2
UBC	SYNGR1
EGFR	SYNGAP1
DLGAP4	SYNGAP1
UBC	SYNE2
ACTC1	SYNE2
UBC	SYNCRIP
SYNCRIP	SYT5
SYNCRIP	SYT2
SYT1	SYNCRIP
PRMT1	SYNCRIP
SYNCRIP	HNRNPD
HNRNPA1	SYNCRIP
SYNCRIP	A1CF
SYN3	SYN2
WWOX	SYMPK
UBC	SYMPK
SUMO2	SYMPK
HTT	SYMPK

VAV1	SYK
USP25	SYK
UBC	SYK
UBASH3B	SYK
TRAF6	SYK
STAT3	SYK
SNCA	SYK
SLC4A1	SYK
SIT1	SYK
PXN	SYK
PTPN6	SYK
PTK2B	SYK
PLCG2	SYK
PLCG1	SYK
PIK3R1	SYK
LCP2	SYK
LCK	SYK
ITGB2	SYK
FYN	SYK
FGR	SYK
FCGR2A	SYK
ERBB4	SYK
ERBB3	SYK
EGFR	SYK
CSF3R	SYK
CRKL	SYK
CD79A	SYK
CD33	SYK
CBLB	SYK
BLNK	SYK
UBC	SYCP1
SH3PXD2A	SVIL
MYH9	SVIL
MIF4GD	SVIL
UBC	SV2A
WDR77	SUZ12
UBC	SUZ12
SUMO2	SUZ12
SIRT1	SUZ12
RBBP4	SUZ12
JARID2	SUZ12
HDAC2	SUZ12
EZH2	SUZ12
EZH1	SUZ12

EED	SUZ12
AEBP2	SUZ12
UBC	SUSD1
UBC	SURF4
PLAUR	SURF2
B4GALT3	SURF2
TAF9	SUPT7L
ZNF512B	SUPT5H
ZFYVE9	SUPT5H
UBC	SUPT5H
SUPT4H1	SUPT5H
RPL8	SUPT5H
PRMT5	SUPT5H
POLR2C	SUPT5H
POLR2A	SUPT5H
PIN1	SUPT5H
CDK9	SUPT5H
CDK7	SUPT5H
CCNT1	SUPT5H
TRRAP	SUPT3H
TAF9	SUPT3H
SUPT3H	TAF12
SUPT3H	TADA1
SUPT3H	SUPT7L
SUPT3H	SAP130
MYC	SUPT3H
SUPT3H	KAT2B
KAT2A	SUPT3H
SUPT3H	ATXN7
MAPK14	SUPT20H
UBC	SUPT16H
H3F3A	SUPT16H
UBC	SUN2
UBC	SUN1
VIM	SUMO3
UBC	SUMO3
UBA2	SUMO3
TRIM28	SUMO3
TP53	SUMO3
SUMO3	TDG
SUMO1	SUMO3
SUMO3	RANGAP1
SUMO3	PIAS1
NPM1	SUMO3

SUMO3	NFKBIA
FOS	SUMO3
SUMO2	ZNF687
SUMO2	ZNF644
SUMO2	ZNF281
SUMO2	ZNF148
SUMO2	ZNF131
SUMO2	ZNF106
SUMO2	ZMYM4
SUMO2	ZMYM2
SUMO2	ZMYM1
SUMO2	ZHX1
SUMO2	ZEB1
SUMO2	ZC3H11A
SUMO2	ZBTB9
SUMO2	ZBTB7A
SUMO2	ZBTB4
SUMO2	ZBTB21
SUMO2	ZBTB2
SUMO2	YY1
SUMO2	YEATS2
SUMO2	XRCC1
SUMO2	XPC
SUMO2	WRN
SUMO2	WDR3
SUMO2	WDHD1
SUMO2	WAPAL
SUMO2	VPS72
SUMO2	VIM
SUMO2	VCP
SUMO2	USPL1
SUMO2	USP7
SUMO2	USP39
SUMO2	USP28
SUMO2	USP1
SUMO2	UIMC1
SUMO2	UHRF1
SUMO2	UBXN7
SUMO2	UBTF
SUMO2	UBN2
SUMO2	UBE2T
SUMO2	UBE2K
SUMO2	UBE2I
UBC	SUMO2

SUMO2 UBA2
SUMO2 TUBB
SUMO2 TTLL12
SUMO2 TRIM63
SUMO2 TRIM33
SUMO2 TRIM28
SUMO2 TRIM24
SUMO2 TPR
SUMO2 TP53BP1
SUMO2 TP53
SUMO2 TOP2A
SUMO2 TOP1
SUMO2 TMPO
SUMO2 THRAP3
SUMO2 THOC1
SUMO2 TFRC
SUMO2 TFIP11
SUMO2 TFAP2C
SUMO2 TERF2IP
SUMO2 TEAD4
SUMO2 TDP2
SUMO2 TDG
SUMO2 TCF20
SUMO2 TCERG1
SUMO2 TARDBP
SUMO2 TAF9
SUMO2 SUPT7L
SUMO2 SUPT16H
SUMO2 SUMO3
SUMO2 SUMO1
SUMO2 SUDS3
SUMO2 SPTBN1
SUMO2 SP3
SUMO2 SNRPA1
SUMO2 SNRNP200
SUMO2 SNIP1
SUMO2 SND1
SUMO2 SMARCE1
SUMO2 SMARCC1
SUMO2 SMARCAD1
SUMO2 SLX4
SUMO2 SLC3A2
SUMO2 SKIV2L2
SUMO2 SIN3B

SUMO2	SFPQ
SUMO2	SF3B1
SUMO2	SET
SUMO2	SERBP1
SUMO2	SENP3
SUMO2	SCAF11
SUMO2	SART1
SUMO2	SARNP
SUMO2	SAP30BP
SUMO2	SAP18
SUMO2	SALL4
SUMO2	RXRB
SUMO2	RUVBL2
SUMO2	RUVBL1
SUMO2	RSF1
SUMO2	RRP15
SUMO2	RREB1
SUMO2	RPSA
SUMO2	RPS5
SUMO2	RPS27A
SUMO2	RPLP1
SUMO2	RPL3
SUMO2	RPL26
SUMO2	RPA1
SUMO2	RNF169
SUMO2	RLF
SUMO2	RFC1
SUMO2	RBM6
SUMO2	RBM39
SUMO2	RBM25
SUMO2	RBM15
SUMO2	RANGAP1
SUMO2	RAD18
SUMO2	RABGGTB
SUMO2	PUS7
SUMO2	PUF60
SUMO2	PTMA
SUMO2	PTBP1
SUMO2	PSMD7
SUMO2	PSMD4
SUMO2	PSMD1
SUMO2	PSIP1
SUMO2	PRPF8
SUMO2	PRPF6

SUMO2	PPP2R5D
SUMO2	PPM1G
SUMO2	POLR3D
SUMO2	POLD3
SUMO2	POGZ
SUMO2	PNN
SUMO2	PIAS3
SUMO2	PIAS1
SUMO2	PHIP
SUMO2	PHF10
SUMO2	PES1
SUMO2	PELP1
SUMO2	PDCD4
SUMO2	PDCD11
SUMO2	PCBP2
SUMO2	PBRM1
SUMO2	PARP1
SUMO2	P4HB
SUMO2	OGFR
SUMO2	OGFOD1
SUMO2	NUP93
SUMO2	NUP155
SUMO2	NUP153
SUMO2	NUMA1
SUMO2	NSUN2
SUMO2	NSMCE2
SUMO2	NPM3
SUMO2	NPM1
SUMO2	NPAT
SUMO2	NOP58
SUMO2	NOP2
SUMO2	NOLC1
SUMO2	NOL9
SUMO2	NFRKB
SUMO2	NFIL3
SUMO2	NFE2L2
SUMO2	NFAT5
SUMO2	NEDD8
SUMO2	NCOR2
SUMO2	NCL
SUMO2	NACC1
SUMO2	NAB2
SUMO2	NAB1
SUMO2	MYSM1

SUMO2	MYL6
SUMO2	MYBL2
SUMO2	MYBBP1A
SUMO2	MYB
SUMO2	MTA2
SUMO2	MSANTD4
SUMO2	MRE11A
SUMO2	MORC3
SUMO2	MKL1
SUMO2	MKI67
SUMO2	MITF
SUMO2	MEF2D
SUMO2	MED8
SUMO2	MDC1
SUMO2	MCM7
SUMO2	MCM4
SUMO2	MCM3
SUMO2	MCM2
SUMO2	MBD1
SUMO2	MASTL
SUMO2	MAFK
SUMO2	MAFF
SUMO2	LRPPRC
SUMO2	LMNA
SUMO2	LIN54
SUMO2	LIN37
SUMO2	LIG3
SUMO2	LEO1
SUMO2	L3MBTL2
SUMO2	KPNA2
SUMO2	KNOP1
SUMO2	KDM5B
SUMO2	KDM3B
SUMO2	KDM2B
SUMO2	KDM1A
SUMO2	KCTD15
SUMO2	JUND
SUMO2	JUNB
SUMO2	IWS1
SUMO2	IRF2BPL
SUMO2	IRF2BP1
SUMO2	IPO7
SUMO2	IPO5
SUMO2	INTS4

SUMO2	INTS3
SUMO2	ILF2
SUMO2	IK
SUMO2	HSPD1
SUMO2	HSPA8
SUMO2	HSPA5
SUMO2	HSPA1A
SUMO2	HSF2
SUMO2	HP1BP3
SUMO2	HOXC10
SUMO2	HNRNPR
SUMO2	HNRNPL
SUMO2	HNRNPH1
SUMO2	HNRNPF
SUMO2	HNRNPC
SUMO2	HMBOX1
SUMO2	HIST4H4
SUMO2	HIST3H2BB
SUMO2	HIST1H1C
SUMO2	HIPK2
SUMO2	HDAC4
SUMO2	HDAC1
SUMO2	GTF3C4
SUMO2	GTF3C3
SUMO2	GTF3C2
SUMO2	GTF2IRD1
SUMO2	GTF2I
SUMO2	GON4L
SUMO2	GMPS
SUMO2	GLYR1
SUMO2	GATAD2B
SUMO2	GATAD2A
SUMO2	FOXP1
SUMO2	FOXM1
SUMO2	FOXC1
SUMO2	FOXA1
SUMO2	FOSL2
SUMO2	FOS
SUMO2	FEN1
SUMO2	FANCD2
SUMO2	FAM192A
SUMO2	EXOSC9
SUMO2	EXOSC10
SUMO2	ETV6

SUMO2	ETV5
SUMO2	ETS1
SUMO2	ESF1
SUMO2	ERCC4
SUMO2	ENO1
SUMO2	ELF3
SUMO2	ELF2
SUMO2	ELAC2
SUMO2	EIF5A
SUMO2	EIF4A1
SUMO2	EIF3K
SUMO2	EIF3E
SUMO2	EFTUD2
SUMO2	EEF2
SUMO2	EEF1G
EEF1A1	SUMO2
SUMO2	DNTTIP2
SUMO2	DNMT1
SUMO2	DNAJC8
SUMO2	DIS3
SUMO2	DHX38
SUMO2	DEK
SUMO2	DDX5
SUMO2	DDX47
SUMO2	DDX46
SUMO2	DDX39A
SUMO2	DDX23
SUMO2	DDX21
SUMO2	DDX20
SUMO2	DDX18
SUMO2	DCD
SUMO2	DAXX
SUMO2	CTNND1
SUMO2	CRABP2
SUMO2	COIL
SUMO2	CLTC
SUMO2	CLSPN
SUMO2	CHD8
SUMO2	CHD4
SUMO2	CHD3
SUMO2	CHAMP1
SUMO2	CHAF1A
SUMO2	CENPB
SUMO2	CEBPB

SUMO2 CEBPA
SUMO2 CDK11B
SUMO2 CDC73
SUMO2 CD3EAP
SUMO2 CBX4
SUMO2 CASP8AP2
SUMO2 CANX
SUMO2 C17orf85
SUMO2 C15orf39
SUMO2 C11orf84
SUMO2 C11orf30
SUMO2 BYSL
SUMO2 BTAF1
SUMO2 BRE
SUMO2 BRD8
SUMO2 BRD4
SUMO2 BRCA1
SUMO2 BRAT1
SUMO2 BLM
SUMO2 BHLHE40
SUMO2 BEND3
SUMO2 BCLAF1
SUMO2 BBX
SUMO2 BAZ2A
SUMO2 BAZ1B
SUMO2 BAZ1A
SUMO2 ATXN2L
SUMO2 ATP6V1A
SUMO2 ATF7
SUMO2 ATAD5
SUMO2 ASH1L
SUMO2 ARID4B
SUMO2 ARID4A
SUMO2 ARID3A
SUMO2 ARHGDIA
SUMO2 ANXA5
SUMO2 ANP32E
SUMO2 ANAPC7
SUMO2 ANAPC4
SUMO2 ALDOA
SUMO2 AK2
SUMO2 AHNAK
SUMO2 ADNP
SUMO2 ADAR

SUMO2	ACTBL2
SUMO2	ACTB
SUMO2	ACIN1
SUMO1	ZNF131
SUMO1	WRN
SUMO1	VIM
SUMO1	VCP
UBC	SUMO1
SUMO1	TUBB
SUMO1	TRPS1
TRIM28	SUMO1
TRAF6	SUMO1
TP53	SUMO1
SUMO1	TOP1
SUMO1	TNFRSF1A
SUMO1	TDG
SUMO1	SFPQ
SUMO1	SF3A2
SUMO1	SCAPER
SUMO1	SART1
SUMO1	RPSA
SUMO1	RPS3
SUMO1	RPL8
SUMO1	RPL3
SUMO1	RAD52
SUMO1	RAD51
SUMO1	PTBP1
SUMO1	PSMC3
SUMO1	PPARG
SUMO1	PARK7
SUMO1	NPM1
SUMO1	NFKBIA
SUMO1	NFE2L2
SUMO1	NCOR2
SUMO1	MYB
SUMO1	MDM2
SUMO1	KHSRP
SUMO1	HTT
SUMO1	HSP90B1
SUMO1	HNRNPH3
SUMO1	HNRNPH1
SUMO1	HNRNPA3
SUMO1	HNRNPA1
SUMO1	HIST4H4

SUMO1	HIST1H1D
SUMO1	HIST1H1C
SUMO1	HIST1H1B
SUMO1	HIPK2
SUMO1	HDAC4
SUMO1	ETV6
SUMO1	EIF4A2
EEF1G	SUMO1
SUMO1	DDX5
SUMO1	DDX39B
SUMO1	DAXX
SUMO1	C11orf65
SUMO1	BUB1B
SUMO1	ATXN3
SUMO1	ANXA2
ACTN1	SUMO1
SULT1E1	SULT2B1
ALS2CR12	SULT2B1
RBM48	SULT1E1
C14orf1	SULT1E1
UBC	SUGT1
SKP1	SUGT1
NOD1	SUGT1
NLRP2	SUGT1
DSN1	SUGT1
SUFU	ZNF219
SUFU	SAP18
SUFU	QPRT
SUFU	GLI3
UBC	SUCLG2
SUCLG1	SUCLG2
UBC	SUCLA2
SUCLG1	SUCLA2
UBC	STYXL1
STX4	STXBP6
STX1A	STXBP6
SNAP25	STXBP6
STX1A	STXBP5L
UBC	STXBP3
UBC	STXBP2
USO1	STXBP1
UBC	STXBP1
STX3	STXBP1
STX1A	STXBP1

SNAP25	STXBP1
PRKCG	STXBP1
PRKCB	STXBP1
PRKCA	STXBP1
DOC2B	STXBP1
DOC2A	STXBP1
CDK5	STXBP1
APBA2	STXBP1
UBC	STX8
STX8	NSF
VTI1B	STX7
VPS18	STX7
VPS11	STX7
VAMP8	STX7
UBC	STX7
STX8	STX7
STX6	STX7
GOPC	STX6
HAP1	STX5
STX4	VAMP8
STX4	VAMP3
UBC	STX4
STX4	STXBP4
STX4	STXBP3
STX4	SNAP25
STX4	SEPT5
RAB4A	STX4
RAB11A	STX4
STX4	NSF
VCL	STX3
STX3	VAMP3
UBC	STX3
STX3	STXBP2
SNAP25	STX3
STXBP2	STX2
VIM	STX1A
VAMP8	STX1A
STX1A	SYT1
STX1A	STXBP2
STX8	STX1A
STX1A	SNAP25
STX1A	SLC6A4
STX1A	SLC6A1
STX1A	SEPT2

SCNN1B	STX1A
STX1A	SCNN1A
STX1A	NSF
STX1A	KCNB1
STX1A	CPLX1
STX1A	CFTR
STX1A	CDK5
STX1A	APBA1
UBC	STX18
UBC	STX16
PTPN12	STX16
UBC	STX12
STX11	SNAP25
PSMC3	STX11
STX11	CCNK
UBE2N	STUB1
UBC	STUB1
TP53	STUB1
SMAD1	STUB1
PSMD4	STUB1
STUB1	NOS1
MAPT	STUB1
MAP3K5	STUB1
STUB1	MAP3K14
HSPA8	STUB1
STUB1	GPR37
STUB1	CYP3A4
ATXN3	STUB1
ATXN1	STUB1
UBC	STT3B
RPN1	STT3B
ZRANB1	STRN3
UBC	STRN3
STRN	STRN3
STK25	STRN3
STK24	STRN3
PPP2CB	STRN3
PPP2CA	STRN3
MOB4	STRN3
CALM1	STRN3
ZRANB1	STRN
UBC	STRN
STK24	STRN
PPP2R1B	STRN

MOB4	STRN
UBC	STRIP2
STK24	STRIP2
MOB4	STRIP2
ZRANB1	STRIP1
UBC	STRIP1
STK24	STRIP1
MOB4	STRIP1
UBC	STRBP
THAP1	STRBP
SMAD2	STRAP
CAB39	STRADA
FANCA	STRA13
APITD1-CORT	STRA13
UBC	STOX1
UBC	STOML2
UBC	STOM
UBC	STMN3
UBC	STMN2
STMN2	RGS6
STMN2	MAPK3
UBC	STK4
TP53	STK4
STK3	STK4
SAV1	STK4
RASSF5	STK4
RASSF4	STK4
RASSF2	STK4
RASSF1	STK4
PRKRIR	STK4
HIST2H2BE	STK4
CASP3	STK4
WNK4	STK39
UBC	STK39
SLC12A2	STK39
MOB2	STK38L
MOB1A	STK38L
UBC	STK38
S100B	STK38
MOB2	STK38
MOB1A	STK38
SUFU	STK36
MAST2	STK36
GLI3	STK36

GLI2	STK36
GLI1	STK36
CDK5RAP2	STK36
UBC	STK3
STK3	SAV1
STK3	RASSF6
STK3	RASSF5
STK3	RASSF4
STK3	RASSF2
RASSF1	STK3
RAF1	STK3
STK3	LATS2
UBC	STK25
TCP1	STK25
STRN	STK25
STK25	STRIP2
STK24	STK25
STK25	SLMAP
PPP2R1B	STK25
PDCD10	STK25
MOB4	STK25
STK25	FGFR1OP2
TUBB	STK24
STK24	PDCD10
STK24	CTTNBP2
STK24	CCT8
SF3B4	STK19
POLR2G	STK19
POLE	STK19
UBC	STK17A
RPIA	STK16
KCTD17	STK16
ADAMTSL4	STK16
UBC	STK11IP
STK11	STK11IP
SMAD4	STK11IP
STK11	TNIP2
STRADB	STK11
STK11	STRADA
RPS6KB1	STK11
PRKAA2	STK11
MOB4	STK11
MARK1	STK11
HSP90AA1	STK11

STK11	FKBP5
STK11	CDC37
CAB39	STK11
ATM	STK11
UBC	STK10
UBC	STEAP4
UBC	STEAP1
UBC	STC2
ARRB2	STC2
ARHGEF40	STC2
ADAM11	STC2
UBC	STAU2
UPF1	STAU1
UBC	STAU1
PPP1CA	STAU1
MAP3K14	STAU1
EIF2AK2	STAU1
OVGP1	STATH
UBC	STAT5B
NCOR2	STAT5B
IL2RB	STAT5B
GHR	STAT5B
EGFR	STAT5B
CD247	STAT5B
UBC	STAT5A
TSLP	STAT5A
STAT5B	STAT5A
PRTFDC1	STAT5A
PPIB	STAT5A
NCOR2	STAT5A
JAK3	STAT5A
JAK1	STAT5A
IL2RB	STAT5A
GHR	STAT5A
ERBB4	STAT5A
EGFR	STAT5A
CD247	STAT5A
NMI	STAT4
IL12RB2	STAT4
UBC	STAT3
STAT3	TSLP
STAT1	STAT3
NFKB1	STAT3
MAPK9	STAT3

JAK1	STAT3
HCK	STAT3
GTF2I	STAT3
GHR	STAT3
STAT3	FGFR4
FER	STAT3
EGFR	STAT3
STAT3	CSF3R
BHLHE40	STAT3
UBC	STAT2
STAT1	STAT2
IFNAR2	STAT2
IFNAR1	STAT2
UBC	STARD4
UBC	STARD3NL
SMAD1	STARD13
LIPE	STAR
FEZ1	STAR
PTK6	STAP2
BMP4	STAP2
TEC	STAP1
UBC	STAMBP
STAM2	STAMBP
GRAP2	STAMBP
CHMP4B	STAMBP
CHMP1B	STAMBP
STAM2	JAK3
DAZAP2	STAM2
USP8	STAM
UBC	STAM
STAMBP	STAM
PPP1CA	STAM
JAK3	STAM
JAK2	STAM
CXCR4	STAM
CLTC	STAM
UBC	STAG1
SMC3	STAG1
SMAD2	STAG1
PDS5B	STAG1
PDS5A	STAG1
UBC	ST7
ST3GAL3	RPL8
UBC	ST20-MTHFS

TSC22D4 ST20-MTHFS
UBC ST14
SPINT1 ST14
UBC ST13
SMAD2 ST13
OLFM2 ST13
HSPA8 ST13
HSPA1A ST13
CD101 ST13
UBC SSU72
SSTR1 SSTR5
SST SSTR5
CORT SSTR5
ADCY10 SSTR5
TP53 SSTR3
SST SSTR3
CORT SSTR3
SST SSTR4
SST SSTR2
SSTR1 SST
VTA1 SSSCA1
UBC SSSCA1
TRAPPC6A SSSCA1
RNF115 SSSCA1
NOC4L SSSCA1
FXR2 SSSCA1
CCT5 SSSCA1
CCT3 SSSCA1
CCNH SSSCA1
ALS2CR11 SSSCA1
UBC SSRP1
SUPT16H SSRP1
SUMO2 SSRP1
SUMO1 SSRP1
SSRP1 SRF
SSRP1 SPI1
SSRP1 ROBO2
SSRP1 NEK9
SSRP1 MYCBP
SSRP1 MCM7
SSRP1 MCM6
SSRP1 MCM5
MCM4 SSRP1
SSRP1 MCM3

SSRP1	MCM2
H3F3A	SSRP1
H2AFX	SSRP1
EXOSC10	SSRP1
DHX9	SSRP1
SSRP1	CHD1
UBC	SSR2
UBC	SSR1
SSR1	NUP54
UBC	SSFA2
UBC	SSBP1
PXN	SSBP1
POLG	SSBP1
UBC	SSB
SSB	MAP3K14
SSB	GZMB
UBC	SS18L2
SMARCA2	SS18
YBX1	SRSF9
UBC	SRSF9
TRA2B	SRSF9
SAFB	SRSF9
UBC	SRSF8
UBC	SRSF7
NXF1	SRSF7
NEDD4	SRSF7
UBC	SRSF6
SRSF4	SRSF6
SRPK1	SRSF6
SREK1	SRSF6
RNPS1	SRSF6
UBC	SRSF5
TNK2	SRSF5
SRPK1	SRSF5
PPARGC1A	SRSF5
OLFM2	SRSF5
CD101	SRSF5
UBC	SRSF4
SRPK1	SRSF4
HAP1	SRSF4
SRSF2	SRSF11
SNRNP70	SRSF11
UBC	SRSF1
U2AF2	SRSF1

U2AF1	SRSF1
TOP1	SRSF1
TNPO3	SRSF1
SRPK2	SRSF1
SRPK1	SRSF1
SREK1	SRSF1
SNRNP70	SRSF1
SF3A2	SRSF1
SCAF11	SRSF1
RNPS1	SRSF1
PRPF4	SRSF1
NXF1	SRSF1
LNX1	SRSF1
DIDO1	SRSF1
CLK1	SRSF1
CDC5L	SRSF1
VIM	SRRT
UBC	SRRT
CHD3	SRRT
YWHAG	SRRM2
YWHAE	SRRM2
YWHAB	SRRM2
UBC	SRRM2
SUMO2	SRRM2
SRRM1	SRRM2
SNRPC	SRRM2
SNRPA1	SRRM2
SF3B4	SRRM2
SF3B3	SRRM2
SEPT2	SRRM2
RBM39	SRRM2
PRPF6	SRRM2
PNN	SRRM2
PIN1	SRRM2
HSPA1A	SRRM2
HNRNPH1	SRRM2
HNRNPA1	SRRM2
EEF1A1	SRRM2
DEK	SRRM2
DDX5	SRRM2
YWHAG	SRRM1
UBC	SRRM1
SRRM1	SRSF6
SRSF4	SRRM1

SRSF3 SRRM1
SRRM1 SNRPE
RBM39 SRRM1
RBM25 SRRM1
PRPF8 SRRM1
UBC SRR
UBC SRPRB
CDK5RAP3 SRPRB
RUSC2 SRPR
SNRNP70 SRPK2
SRPK2 RBM39
SRSF3 SRPK1
SNRNP70 SRPK1
UBC SRP9
SRP14 SRP9
UBC SRP72
SKIL SRP72
UBC SRP68
SRP72 SRP68
UBC SRP54
SRP19 SRP54
IPO8 SRP19
UBC SRP14
UBC SRM
UBC SRI
SMAD2 SRI
GCA SRI
CALCOCO2 SRI
ANXA7 SRI
GZMB SRGN
ROBO1 SRGAP1
CDC42 SRGAP1
BMPR1B SRGAP1
UBC SRFBP1
SRF SRFBP1
RXRG SRF
RXRA SRF
RPS6KA1 SRF
SRF PRRX1
SRF NKX3-1
SRF NKX2-3
SRF MYOCD
SRF MKL1
MAPKAPK2 SRF

SRF HOPX
GTF2I SRF
SRF GATA4
SRF ELK4
CREB1 SRF
SRSF3 SREK1
SRSF2 SREK1
SRPK1 SREK1
UBC SREBF2
SUMO1 SREBF2
SP1 SREBF2
SREBF2 SCAP
SREBF2 RAB11B
SREBF2 MAPK1
INSIG1 SREBF2
SREBF2 HNF4A
CASP3 SREBF2
MED1 SREBF1
UBC SRCAP
RUVBL2 SRCAP
KAT2B SRCAP
EP300 SRCAP
DMAP1 SRCAP
CREBBP SRCAP
ATG9A SRCAP
SRC YWHAH
YWHAG SRC
YWHAE SRC
YWHAB SRC
SRC VDR
UBC SRC
TYRO3 SRC
SRC TUB
TRPV4 SRC
SRC TRPC6
TRAF6 SRC
TRAF1 SRC
TNK2 SRC
TNFRSF1A SRC
TIAM1 SRC
SRC THRB
SRC SYK
SRC STAT5A
SRC STAT3

STAT1	SRC
SRCSRF
SRCSMARCE1
SMARCB1	SRC
SRCSLC9A2
SRCSKAP1
SRCSHB
SRCSH3PXD2A
SRCSH3BP1
RXRA	SRC
SRCRGS16
SRCRET
RASA1	SRC
RARA	SRC
RAF1	SRC
PXN	SRC
PTPRE	SRC
SRCPTPRA
PTPN6	SRC
SRCPTPN21
SRCPTPN18
SRCPTPN1
SRCPTK2B
PRKCE	SRC
PLCG1	SRC
PIK3R1	SRC
PGR	SRC
SRCPELP1
SRCPDGFRB
SRCPDE6G
SRCPDE4D
PDCD6IP	SRC
PAK2	SRC
SRCNR3C2
NR3C1	SRC
SRCNPHS1
NMT1	SRC
NCOA6	SRC
SRCMUC1
SRCMPZL1
SRCMET
MED28	SRC
KIT SRC
SRCKIFAP3

KHDRBS1 SRC
KDR SRC
SRCKCNA5
SRCIL6R
SRCIKBKB
HSP90AA1 SRC
SRCHRAS
SRCHNRNPK
SRCHNF1A
SRCHLA-A
SRCHDAC3
SRCGUCY2C
GTF2I SRC
SRCGRIN2B
SRCGRIN2A
GRB2 SRC
SRCGNB2L1
SRCGJA1
SRCGFAP
SRCGAB2
FYN SRC
FOXO1 SRC
FGR SRC
SRCFASLG
SRCFAS
ESR2 SRC
ESR1 SRC
ERBB3 SRC
SRCERBB2
SRCEPS8
EPHB2 SRC
ENO1 SRC
EGFR SRC
SRCDOK1
SRCDNM1
DGKA SRC
DDR2 SRC
DAG1 SRC
DAB2 SRC
DAB1 SRC
CTNNB1 SRC
CRK SRC
CREBBP SRC
CHUK SRC

SRCCDH5
SRCCDH1
CDCP1 SRC
SRCCD59
CD44 SRC
CD36 SRC
CD2AP SRC
SRCCBLL1
SRCCBLC
CBL SRC
SRCCAV1
BCR SRC
SRCBCAR1
AXL SRC
SRCASAP1
ARRB2 SRC
SRCARRB1
SRCARHGAP35
ARHGAP1 SRC
SRCANXA2
SRCAFAP1
SRCADRBK1
SRCADRB3
ADAM12 SRC
ABL1 SRC
UBC SQSTM1
SQSTM1 TRIM63
TRAF6 SQSTM1
TARDBP SQSTM1
SMAD4 SQSTM1
SMAD2 SQSTM1
SMAD1 SQSTM1
SQSTM1 RIPK1
SQSTM1 RAD23A
SQSTM1 NTRK2
SQSTM1 NTRK1
MAPK14 SQSTM1
SQSTM1 MAP2K5
LCK SQSTM1
SQSTM1 GABARAPL2
SQSTM1 BAG3
SQSTM1 ARHGEF28
ACVR1 SQSTM1
UBC SQLE

UBC	SPTY2D1
UBC	SPTLC2
SPTLC1	SPTLC2
UBC	SPTLC1
UBC	SPTBN4
DISC1	SPTBN4
SPTBN2	ACTR1A
ZNF512B	SPTBN1
UBC	SPTBN1
SUMO1	SPTBN1
SNCA	SPTBN1
SMAD9	SPTBN1
IKBKE	SPTBN1
ACTR1A	SPTBN1
UBC	SPTB
AP1G1	SPTB
ANK1	SPTB
ADD1	SPTB
UBC	SPTAN1
TAB2	SPTAN1
SPTAN1	SPTBN1
SPTAN1	SPTB
RIPK3	SPTAN1
PTP4A1	SPTAN1
SPTAN1	PTOV1
NEFL	SPTAN1
IKBKE	SPTAN1
FANCG	SPTAN1
SPTAN1	FANCC
ESR1	SPTAN1
ERCC4	SPTAN1
SPTAN1	DDX24
SPTB	SPTA1
SLC9A2	SPTA1
FANCC	SPTA1
ADD1	SPTA1
TCEB1	SPSB1
UBC	SPRYD3
TESK1	SPRY4
UBC	SPRY2
TRAF2	SPRY2
GRB2	SPRY2
GPRIN2	SPRY2
ADAMTSL4	SPRY2

GRB2	SPRY1
SPRTN	RAD18
UBC	SPRED1
PPP1CA	SPRED1
UBC	SPR
UBC	SPOP
SPOP	RXRB
SPOP	RTN3
SPOP	NCOA3
SPOP	MYD88
SPOP	HCST
DPPA2	SPOP
SPOP	DAXX
UBC	SPINT2
RABL6	SPINK7
MRPS12	SPINK7
KLK5	SPINK5
UBC	SPIN1
UBC	SPICE1
SPIB	SPI1
CREM	SPI1
ATF1	SPI1
SPHK1	RPS6KC1
TRIM28	SPG7
UBC	SPG21
TRAF2	SPG21
SPG21	MTMR9
UBC	SPG20
SPG20	SMURF2
SPG20	PLIN3
SPG20	ITCH
UBC	SPG11
NINL	SPERT
CEP44	SPERT
UBC	SPEN
RBPJ	SPEN
RARA	SPEN
NCOR2	SPEN
MTA2	SPEN
MSX2	SPEN
HDAC2	SPEN
PLEKHF2	SPEF1
UBC	SPECC1
GPS2	SPDL1

NKX3-1	SPDEF
UBC	SPCS3
UBC	SPCS2
TEFM	SPCS2
STMN3	SPCS2
UBC	SPC25
SPC24	SPC25
MIS12	SPC25
UBC	SPC24
UBC	SPATA5L1
UBC	SPATA5
TRAF2	SPATA2
DUT	SPATA2
CEP72	SPATA2
UBC	SPAG9
NFKB1	SPAG9
ITGB7	SPAG9
ITGB2	SPAG9
ARF6	SPAG9
UBC	SPAG7
ABHD16A	SPAG7
UBC	SPAG5
SNAPIN	SPAG5
MGEA5	SPAG5
UBC	SPAG1
ROPN1	SPA17
SP3	STK19
SP1	SP3
RBBP4	SP3
HDAC2	SP3
HDAC1	SP3
ESR1	SP3
UBC	SP140L
UBE2I	SP100
UBC	SP100
SUMO2	SP100
SUMO1	SP100
SNIP1	SP100
PML	SP100
ETS1	SP100
CBX5	SP100
VHL	SP1
UBC	SP1
TP53	SP1

SP1	TAL1
SP1	TAF4
SUMO1	SP1
SP1	STAT3
SP1	SREBF1
SP1	SP4
SMAD4	SP1
SMAD2	SP1
RXRA	SP1
SP1	RBBP4
SP1	RARA
SP1	PRKCD
SP1	PPARG
PGR	SP1
SP1	NFYB
SP1	NFKB1
MYC	SP1
SP1	MSX1
SP1	MEF2D
SP1	HTT
SP1	HSPA8
SP1	HNF4A
SP1	HMGA1
HDAC4	SP1
HDAC2	SP1
SP1	GATA4
SP1	ELF1
SP1	CCNA2
BCL6	SP1
UBC	SOX9
TRAF2	SOX9
SPEN	SOX9
NR5A1	SOX9
MAF	SOX9
KPNB1	SOX9
HSPA1A	SOX9
EP300	SOX9
UBC	SOX8
SMAD5	SOX5
SMAD1	SOX5
RBBP4	SOX2
DNAJA2	SOX2
ARID1A	SOX2
UBC	SOWAHC

BMP7 SOSTDC1
UBC SOS1
RRAS SOS1
PLCG1 SOS1
PIK3R1 SOS1
MUC1 SOS1
ITSN2 SOS1
ITSN1 SOS1
HRAS SOS1
HCK SOS1
GRB2 SOS1
GRAP SOS1
GAB1 SOS1
FYN SOS1
ERBB3 SOS1
EGFR SOS1
CSF1R SOS1
CRKL SOS1
CRK SOS1
ABI1 SOS1
UBC SORT1
UBC SORD
ZBTB25 SORBS3
WASL SORBS3
PAK2 SORBS3
KIAA1217 SORBS3
KIAA0408 SORBS3
HNRNPK SORBS3
EFS SORBS3
DVL2 SORBS3
DLG5 SORBS3
DDX17 SORBS3
CPSF7 SORBS3
CBLB SORBS3
ADAMTSL4 SORBS3
SORBS2 SEMA6A
VCL SORBS1
SEMA6A SORBS1
INSM1 SORBS1
INPPL1 SORBS1
YWHAG SON
UBC SON
SNIP1 SON
PRPF40A SON

UBC	SOGA1
UBC	SOD2
RPS3A	SOD2
RAB4A	SOD2
NOL12	SOD2
GET4	SOD2
UBC	SOD1
STUB1	SOD1
RNF19A SOD1
CCSSOD1
UBC	SOCS6
TCEB2	SOCS6
TCEB1	SOCS6
KIT SOCS6
ACVR1	SOCS6
EGFR	SOCS4
UBC	SOCS3
TCEB2	SOCS3
TCEB1	SOCS3
RASA1	SOCS3
PTPN11	SOCS3
PTK2B	SOCS3
PIN1	SOCS3
LEPR	SOCS3
JAK3	SOCS3
JAK2	SOCS3
JAK1	SOCS3
IRS1	SOCS3
IL6ST	SOCS3
IGF1R	SOCS3
GHR	SOCS3
EPOR	SOCS3
EGFR	SOCS3
CXCR4	SOCS3
CUL5	SOCS3
CSF1R	SOCS3
TCEB1	SOCS2
GHR	SOCS2
EPOR	SOCS2
CUL5	SOCS2
VAV2	SOCS1
VAV1	SOCS1
TRIM8	SOCS1
TECSOCS1

TCEB2	SOCS1
TCEB1	SOCS1
RELA	SOCS1
PDGFRB	SOCS1
NCK1	SOCS1
MAP3K5	SOCS1
KIT	SOCS1
JAK3	SOCS1
JAK2	SOCS1
JAK1	SOCS1
ITK	SOCS1
IRS1	SOCS1
IL2RB	SOCS1
GRB2	SOCS1
GHR	SOCS1
FYN	SOCS1
EGFR	SOCS1
CSF1R	SOCS1
UBC	SOAT1
UBC	SNX9
TNK2	SNX9
MPHOSPH6	SNX9
SNX9	DNM2
SNX9	CLTC
SNX9	ADAM9
UBC	SNX8
UBC	SNX6
SNX1	SNX6
UBC	SNX5
ITSN1	SNX5
UBC	SNX4
SNX2	SNX4
PDGFRA	SNX4
LEPR	SNX4
EGFR	SNX4
AGK	SNX4
SNX9	SNX33
UBC	SNX3
STX11	SNX3
UBC	SNX27
SH3GL3	SNX24
KLHL12	SNX20
UBC	SNX2
SNX2	SNX6

SNX2	SNX1
SNX2	PDGFRA
FNBP1	SNX2
EGFR	SNX2
SKIL	SNX17
UBC	SNX14
UBC	SNX11
UBC	SNX1
PDGFRB	SNX1
EGFR	SNX1
SMAD2	SNW1
NCOR2	SNW1
SNW1	CIR1
SNW1	ASCC2
UBC	SNUPN
SNUPN	PEX5
UTRN	SNTB2
UBC	SNTB2
KCNJ12	SNTB2
ERBB4	SNTB2
ABCA1	SNTB2
UTRN	SNTB1
MAPK12	SNTB1
KCNJ12	SNTB1
ADRA1D	SNTB1
ABCA1	SNTB1
XRCC6	SNTA1
UTRN	SNTA1
TGFA	SNTA1
SCN4A	SNTA1
NOS1	SNTA1
MED8	SNTA1
KCNJ4	SNTA1
KCNJ12	SNTA1
KCNA4	SNTA1
GRB2	SNTA1
GLS2	SNTA1
GLS	SNTA1
DGKZ	SNTA1
CALM1	SNTA1
ADRA1D	SNTA1
ABCA1	SNTA1
VCL	SNRPF
UBC	SNRPF

SNRPG	SNRPF
SNRPE	SNRPF
SF3A2	SNRPF
LSM5	SNRPF
DDX20	SNRPF
CLNS1A	SNRPF
CD2BP2	SNRPF
VCL	SNRPE
UBC	SNRPE
SNRPG	SNRPE
SF3A2	SNRPE
LSM5	SNRPE
LSM4	SNRPE
LSM2	SNRPE
GEMIN6	SNRPE
DDX20	SNRPE
CLNS1A	SNRPE
UBC	SNRPD3
SF3A2	SNRPD3
PRPF8	SNRPD3
PRPF6	SNRPD3
LSM7	SNRPD3
GEMIN7	SNRPD3
GEMIN6	SNRPD3
DDX20	SNRPD3
CLNS1A	SNRPD3
UBC	SNRPD2
SUMO2	SNRPD2
SNRPF	SNRPD2
SNRPE	SNRPD2
SF3A2	SNRPD2
LSM6	SNRPD2
LSM2	SNRPD2
KAT5	SNRPD2
GEMIN7	SNRPD2
GEMIN6	SNRPD2
EGFR	SNRPD2
EEF1G	SNRPD2
CLNS1A	SNRPD2
CDK2	SNRPD2
CDC5L	SNRPD2
UBC	SNRPC
SNRNP70	SNRPC
UBC	SNRPB

TOP3B SNRPB
SNRPE SNRPB
SNRPD3 SNRPB
SNRNP200 SNRPB
SFPQ SNRPB
SF3A2 SNRPB
LGALS1 SNRPB
GEMIN7 SNRPB
GEMIN6 SNRPB
DHX9 SNRPB
CTDP1 SNRPB
COIL SNRPB
CLNS1A SNRPB
CD2BP2 SNRPB
UBC SNRPA1
SNRPB2 SNRPA1
SNRPA SNRPA1
SF3A3 SNRPA1
SF3A2 SNRPA1
SMAD5 SNRPA
SFPQ SNRPA
SNRNP70 SMAD5
SNRNP70 SMAD2
SMAD1 SNRNP70
SKIL SNRNP70
SNRNP70 PRKD2
SNRNP70 GEMIN2
SRPK2 SNRNP27
YWHAB SNRNP200
UBC SNRNP200
SNRNP200 SNRNP40
SNRNP200 SMNDC1
SNRNP200 PRPF8
SNRNP200 PRPF6
PIN1 SNRNP200
MYC SNRNP200
SNRNP200 EFTUD2
SNRNP200 CD2BP2
JUN SNRK
SNIP1 ZMYM2
SNIP1 TDG
SNIP1 SNW1
SNIP1 SMAD4
SMAD2 SNIP1

SMAD1	SNIP1
SNIP1	SF3B1
SNIP1	HIPK1
SNIP1	FAF1
VPS25	SNF8
UBC	SND1
SND1	STAT6
MYB	SND1
BUB1B	SNCG
UBC	SNCAIP
SEPT4	SNCAIP
AES	SNCAIP
YWHAE	SNCA
UBC	SNCA
SNCA	TUBA1A
SNCA	STUB1
SNCA	SNCAIP
SNCA	SLC6A4
SNCA	SLC6A3
SNCA	SLC6A2
SNCA	SIAH2
SNCA	SIAH1
SEPT4	SNCA
SNCA	RAB3A
PSMC3	SNCA
SNCA	PRKCE
SNCA	PLK2
SNCA	PARK7
PARK2	SNCA
MAPT	SNCA
SNCA	MAPK8IP1
SNCA	MAPK1
HSPA8	SNCA
HSPA1A	SNCA
FYN	SNCA
SNCA	ENSA
BAD	SNCA
SNCA	APP
SNCA	ADRBK1
UBC	SNAPIN
SNAP25	SNAPIN
SMAD2	SNAPIN
RGS7	SNAPIN
SNAPIN	LAMC1

KAT7	SNAPIN
KAT5	SNAPIN
IKBKAP	SNAPIN
HAP1	SNAPIN
EEF1G	SNAPIN
SNAPIN	DTNBP1
DDR1	SNAPIN
SNAPIN	BLOC1S2
TBP	SNAPC4
BRF2	SNAPC4
UBC	SNAPC3
UBC	SNAPC1
TBP	SNAPC1
SNAPC3	SNAPC1
RB1	SNAPC1
UBC	SNAP47
UBC	SNAP29
STX7	SNAP29
STX1A	SNAP29
LURAP1	SNAP29
VAMP8	SNAP25
TRIM9	SNAP25
VAMP8	SNAP23
VAMP3	SNAP23
VAMP2	SNAP23
UBC	SNAP23
STXBP5L	SNAP23
STX6	SNAP23
STX4	SNAP23
STX3	SNAP23
STX2	SNAP23
STX1A	SNAP23
STX11	SNAP23
SNAPIN	SNAP23
SCGN	SNAP23
NAPA	SNAP23
FXR2	SNAP23
ABI3	SNAP23
ABI2	SNAP23
HDAC1	SNAI2
UBC	SNAI1
MDM2	SNAI1
GSK3B	SNAI1
UBC	SMYD5

UBC	SMYD3
TP53	SMYD2
HSP90AA1	SMYD2
EPB41L3	SMYD2
UBE2L3	SMURF2
SNRNP70	SMURF2
SMAD5	SMURF2
SMAD2	SMURF2
SMAD1	SMURF2
SKIL	SMURF2
DAZAP2	SMURF2
SMURF2	ARHGAP5
SMURF1	UBE2D3
UBC	SMURF1
SMURF1	TGFBR1
SMURF1	SMURF2
SMAD5	SMURF1
SMAD2	SMURF1
SMAD1	SMURF1
RNF11	SMURF1
SMURF1	PLEKHO1
TRIP13	SMUG1
UBC	SMU1
UBC	SMTN
UBC	SMPD4
PTCH2	SMO
PTCH1	SMO
UBC	SMIM14
LSM4	SMIM14
UBC	SMG9
SMG8	SMG9
SMG1	SMG9
UBC	SMG8
UBC	SMG7
UPF1	SMG6
TERT	SMG6
UBC	SMG5
UPF2	SMG1
UPF1	SMG1
UBC	SMG1
SMG1	TELO2
SMG1	SMG8
MAGOH	SMG1
SMG1	CDH5

UBC	SMEK2
PPP4C	SMEK2
FEZ1	SMEK2
UBC	SMCR8
UBC	SMCHD1
SUMO2	SMCHD1
UBC	SMC6
SMC5	SMC6
NSMCE2	SMC6
SMC5	NSMCE2
UBC	SMC4
SUMO2	SMC4
SMC2	SMC4
NCAPH2	SMC4
NCAPH	SMC4
NCAPG	SMC4
MYC	SMC4
UBC	SMC3
SMC3	PDS5B
SMC3	PDS5A
SMC3	KIFAP3
CDCA5	SMC3
UBC	SMC2
SMC2	NCAPH2
NCAPG	SMC2
UBC	SMARCE1
SMARCD3	SMARCE1
SMARCD2	SMARCE1
SMARCC1	SMARCE1
SMARCB1	SMARCE1
SMARCA2	SMARCE1
SMAD1	SMARCE1
PHF10	SMARCE1
SMARCE1	PBRM1
NCOA1	SMARCE1
ESR2	SMARCE1
ESR1	SMARCE1
SMARCE1	DPF2
SMARCE1	CHD7
BRD7	SMARCE1
ARID1A	SMARCE1
ACTL6A	SMARCE1
ACTB	SMARCE1
UBC	SMARCD2

SMARCD2	SMARCA2
USHBP1	SMARCD1
UBC	SMARCD1
SMARCE1	SMARCD1
SMARCD2	SMARCD1
SMARCC2	SMARCD1
SMARCC1	SMARCD1
SMARCB1	SMARCD1
SMARCA4	SMARCD1
SMARCA2	SMARCD1
RELB	SMARCD1
PHF10	SMARCD1
PBRM1	SMARCD1
NR3C1	SMARCD1
KRT15	SMARCD1
JUN	SMARCD1
FOS	SMARCD1
DPF2	SMARCD1
CCDC85B	SMARCD1
CALCOCO2	SMARCD1
ARID2	SMARCD1
ARID1B	SMARCD1
ARID1A	SMARCD1
UBC	SMARCC2
SUMO2	SMARCC2
SMARCE1	SMARCC2
SMARCD2	SMARCC2
SMARCC1	SMARCC2
SMARCB1	SMARCC2
SMARCA4	SMARCC2
SMARCA2	SMARCC2
SMAD2	SMARCC2
RELB	SMARCC2
POLR2C	SMARCC2
POLR2A	SMARCC2
PHYHIP	SMARCC2
PHF10	SMARCC2
PBRM1	SMARCC2
NR3C1	SMARCC2
SMARCC2	NOVA1
MAP3K3	SMARCC2
ITSN1	SMARCC2
DPF2	SMARCC2
CHD7	SMARCC2

BAZ1B	SMARCC2
ARRB2	SMARCC2
SMARCC2	ARID1B
ARID1A	SMARCC2
ACTL6A	SMARCC2
UBC	SMARCC1
TP53	SMARCC1
SMARCC1	SMARCD3
SMARCC1	SMARCB1
SMARCC1	SMARCA2
SMAD2	SMARCC1
SIN3A	SMARCC1
SMARCC1	PHF10
SMARCC1	PBRM1
NEDD4	SMARCC1
MYC	SMARCC1
SMARCC1	DPF2
CHD4	SMARCC1
SMARCC1	CEBPB
SMARCC1	CCNE1
SMARCC1	BAZ1B
SMARCC1	ARID1A
ACTL6A	SMARCC1
TP53	SMARCB1
SMARCB1	TACC2
SMARCB1	SOX2
SMARCB1	SMARCA2
MYC	SMARCB1
SMARCB1	ARID1A
UBC	SMARCAD1
TRIM28	SMARCAD1
UBC	SMARCA5
SUMO2	SMARCA5
RUVBL2	SMARCA5
RSF1	SMARCA5
RBBP4	SMARCA5
POLR3A	SMARCA5
POLR1B	SMARCA5
POLR1A	SMARCA5
POLE3	SMARCA5
MYBBP1A	SMARCA5
HIST4H4	SMARCA5
HIST1H3A	SMARCA5
HDAC2	SMARCA5

CTNNB1 SMARCA5
BAZ2A SMARCA5
BAZ1B SMARCA5
BAZ1A SMARCA5
UBC SMARCA4
TP53 SMARCA4
SUMO2 SMARCA4
SMARCA4 STK11
SMARCA4 STAT2
SMARCA4 STAT1
SMARCA4 SRF
SP1 SMARCA4
SMARCA4 SMARCE1
SMARCA4 SMARCD3
SMARCD2 SMARCA4
SMARCC1 SMARCA4
SMARCA4 SMARCB1
SMARCA4 SMARCA2
SMAD2 SMARCA4
SIN3A SMARCA4
SMARCA4 REST
SMARCA4 RELB
SMARCA4 RB1
PRMT5 SMARCA4
SMARCA4 PHF10
SMARCA4 PHB
SMARCA4 PBRM1
SMARCA4 MYOD1
MYC SMARCA4
SMARCA4 MED17
SMARCA4 JUNB
HIST1H3A SMARCA4
SMARCA4 HDAC3
HDAC2 SMARCA4
HDAC1 SMARCA4
H2AFX SMARCA4
SMARCA4 FANCA
ESR1 SMARCA4
SMARCA4 DPF2
SMARCA4 CTNNB1
CREBBP SMARCA4
SMARCA4 CHD7
CHD4 SMARCA4
SMARCA4 CEBPB

SMARCA4 CCNE1
SMARCA4 CBX5
SMARCA4 BRCA1
SMARCA4 BAZ1B
SMARCA4 ARID1A
ACTL6A SMARCA4
ACTB SMARCA4
RBBP4 SMARCA2
SMARCA2 PHB
SMARCA2 ARID1A
UBC SMAGP
SMAD9 ZNF8
SMAD9 ZEB2
SMAD9 VCPIP1
SMAD9 UBQLN4
SMAD9 UBQLN1
SMAD9 UBA6
SMAD9 TTC37
SMAD9 TMEM57
SMAD9 TINAGL1
SMAD9 SNRNP70
SMAD9 SMAD4
SMAD9 SMAD2
SMAD9 SIL1
SMAD9 SECISBP2
SMAD9 RRBP1
SMAD9 PSAP
SMAD9 PPP2R5E
SMAD9 PPARD
SMAD9 PNPLA2
SMAD9 PKP2
SMAD9 PAPPA
SMAD9 OTUB1
SMAD9 NAGK
SMAD9 MAN1C1
SMAD9 LMO4
SMAD9 HEY1
SMAD9 EXPH5
SMAD9 EVC2
SMAD9 EIF3F
SMAD9 DNAJC7
SMAD9 CYP11A1
SMAD9 CXXC5
SMAD9 CSH1

SMAD9	CHPF
SMAD9	BAZ1A
SMAD9	ASB2
SMAD9	ARNT
SMAD9	AP2A1
SMAD9	AFF1
SMAD9	ACTB
SMAD7	ZNF107
SMAD7	ZBTB11
YAP1	SMAD7
XRCC6	SMAD7
SMAD7	WWP2
UBC	SMAD7
SMAD7	TTF2
SMAD7	TGFBR2
SMAD7	TGFBR1
TAB1	SMAD7
SMAD7	STRAP
SMAD7	STAMBP
SMAD7	SOX7
SMAD7	SOX5
SMAD7	SOX13
SMAD7	SMURF2
SMURF1	SMAD7
SMAD4	SMAD7
SMAD7	RNF111
SMAD7	PIAS1
PARD3	SMAD7
NEDD4L	SMAD7
NEDD4	SMAD7
SMAD7	MYOD1
SMAD7	MKL2
MAPK14	SMAD7
MAP3K7	SMAD7
KDM2A	SMAD7
SMAD7	ITCH
SMAD7	HDAC3
HDAC2	SMAD7
HDAC1	SMAD7
ERBB2IP	SMAD7
SMAD7	CXXC5
SMAD7	CTNNB1
SMAD7	BMPR1B
SMAD7	BMPR1A

SMAD7	ASH1L
SMAD7	ACVR1B
UBC	SMAD6
SMAD6	TGFBR1
TAB1	SMAD6
SMAD6	STRAP
SMAD6	STAMBP
SMAD6	SMURF2
SMURF1	SMAD6
SMAD4	SMAD6
SMAD1	SMAD6
MAP3K7	SMAD6
SMAD6	HOXC8
SMAD6	ACVR1
SMAD5	SOX7
SMAD5	SFPQ
SMAD5	CXXC5
SMAD4	ZNF8
SMAD4	USP15
SMAD4	USB1
UBC	SMAD4
SMAD4	TRIM33
SMAD4	TOB1
SMAD4	TDG
SMAD4	SUMO1
SMAD4	STUB1
SMAD4	STK35
SNRNP70	SMAD4
SMAD5	SMAD4
SMAD2	SMAD4
SMAD1	SMAD4
SKIL	SMAD4
SMAD4	RPL28
SMAD4	RHOJ
SMAD4	RHEBL1
SMAD4	RASL12
SMAD4	RASD2
SMAD4	RAB3B
SMAD4	PSMD11
SMAD4	PSG9
SMAD4	PBK
SMAD4	NRAS
SMAD4	NEK6
SMAD4	MAPK13

SMAD4 ITK
SMAD4 ILKAP
SMAD4 HOXC8
SMAD4 HOXA9
SMAD4 HMG20A
SMAD4 GPNMB
SMAD4 GLI3
SMAD4 FOXO3
SMAD4 FN1
SMAD4 FBLN1
SMAD4 ERBB2IP
EEF1A1 SMAD4
SMAD4 DNAJB2
SMAD4 DCP1B
SMAD4 DCP1A
SMAD4 CSH1
SMAD4 COPS5
SMAD4 CD59
SMAD3 ZNF8
SMAD3 ZNF329
SMAD3 ZMYM2
ZFYVE9 SMAD3
SMAD3 ZEB2
SMAD3 ZEB1
ZBTB16 SMAD3
YY1 SMAD3
XRCC6 SMAD3
SMAD3 WWP2
WEE1 SMAD3
SMAD3 USP7
SMAD3 USP15
UBC SMAD3
SMAD3 TRIM33
TP53 SMAD3
SMAD3 TOB1
SMAD3 TMED1
SMAD3 TGM2
SMAD3 TGFBR1
SMAD3 ST6GALNAC2
SMAD3 SQSTM1
SP1 SMAD3
SNW1 SMAD3
SNRNP70 SMAD3
SMAD3 SMURF2

SMURF1	SMAD3
SMAD9	SMAD3
SMAD4	SMAD3
SMAD2	SMAD3
SMAD1	SMAD3
SKIL	SMAD3
SMAD3	SECISBP2L
SMAD3	SAP130
SMAD3	RUNX3
RUNX2	SMAD3
RRAS2	SMAD3
SMAD3	RIT1
SMAD3	RBL1
SMAD3	RASL12
SMAD3	RASD2
SMAD3	RAB38
PSMD11	SMAD3
SMAD3	PRDM4
SMAD3	PPP2R2D
SMAD3	PPP2R2B
SMAD3	PLAGL1
SMAD3	PLAG1
PIN1	SMAD3
SMAD3	PEX6
SMAD3	NEDD9
NEDD4L	SMAD3
NEDD4	SMAD3
NCOA6	SMAD3
MYC	SMAD3
SMAD3	MTF2
SMAD3	MEN1
SMAD3	MECOM
SMAD3	MAX
SMAD3	MAPK1
SMAD3	LEF1
KDM2A	SMAD3
SMAD3	KAT2B
SMAD3	KANSL1
SMAD3	JUND
SMAD3	JUNB
SMAD3	HIF1A
SMAD3	HEY1
HDAC1	SMAD3
SMAD3	GSK3B

SMAD3 GSC
GLI3 SMAD3
SMAD3 GGA1
FOXO3 SMAD3
FOXO1 SMAD3
SMAD3 FOXH1
FOS SMAD3
SMAD3 ETS1
ESR1 SMAD3
ERBB2IP SMAD3
SMAD3 EPAS1
SMAD3 EBI3
SMAD3 DAB2
SMAD3 CYP11A1
SMAD3 CXXC5
CUL1 SMAD3
SMAD3 CTNNB1
SMAD3 CSNK1D
CREBBP SMAD3
SMAD3 CFDP1
SMAD3 CEBPB
SMAD3 CDK4
SMAD3 CDC27
CDC25A SMAD3
SMAD3 C10orf54
SMAD3 BRCA1
SMAD3 AXIN1
SMAD3 ANAPC10
SKIL SMAD2
SMAD2 RUNX2
SMAD2 NAGK
SMAD2 BTBD2
SMAD2 ANTXR2
SMAD1 ZNF8
SMAD1 YAP1
SMAD1 XPC
SMAD1 SMAD5
SMAD1 SMAD2
SKIL SMAD1
SMAD1 RUNX2
SMAD1 PSMD11
SMAD1 PSMD1
SMAD1 PRKD2
SMAD1 NOTCH2

SMAD1 GSC
SMAD1 GLI3
SMAD1 FHL5
SMAD1 BTBD2
UBC SLX4
SLX4 SLX4IP
PLK1 SLX4
ERCC4 SLX4
UBC SLTM
PLNSLN
ZRANB1 SLMAP
STK24 SLMAP
MOB4 SLMAP
UBC SLK
ROBO1 SLIT2
UBC SLIRP
UBC SLFN5
UBC SLFN11
UBC SLCO4A1
SLC9A3R2 ATP2B2
YAP1 SLC9A3R1
PTEN SLC9A3R1
SLC9A3R1 CLCN3
SLC9A3R2 SLC9A3
SLC9A3R1 SLC9A3
PDZK1 SLC9A3
LRP2 SLC9A3
CHP2 SLC9A3
CHP1 SLC9A3
SPTAN1 SLC9A2
RASA1 SLC9A2
PIK3R1 SLC9A2
ABL1 SLC9A2
YWHAB SLC9A1
UBC SLC9A1
TESC SLC9A1
MDFI SLC9A1
MAP4K4 SLC9A1
CHP2 SLC9A1
CALM1 SLC9A1
CA2SLC9A1
CAV3 SLC8A1
UBC SLC7A5
SLC3A2 SLC7A5

UBC SLC7A2
UBC SLC7A11
SLC3A2 SLC7A11
BAAT SLC7A11
UBC SLC7A1
UBC SLC6A9
UBC SLC6A3
TGFB1I1 SLC6A3
NEDD4 SLC6A3
UBC SLC6A17
UBC SLC5A3
HSPA1A SLC5A1
UBC SLC4A8
SLC9A3R1 SLC4A8
YWHAB SLC4A7
UBC SLC4A7
SLC4A4 SLC4A7
CFTR SLC4A7
CA2SLC4A4
UBC SLC4A2
UBC SLC4A1AP
SUMO2 SLC4A1AP
SLC4A1 SLC4A1AP
SLC4A1 RHAG
SLC4A1 CA4
CA2SLC4A1
SLC4A1 ANK1
UBC SLC44A1
UBC SLC43A3
UBC SLC43A2
UBC SLC43A1
UBC SLC40A1
SLC3A2 SLC7A7
UBC SLC39A14
UBC SLC39A10
PLEKHM2 SLC39A1
UBC SLC38A2
STX11 SLC38A2
UBC SLC38A1
ACAP3 SLC35G2
UBC SLC35F6
UBC SLC35F5
UBC SLC35F2
UBC SLC35E1

UBC	SLC35C2
UBC	SLC35A3
UBC	SLC30A6
UBC	SLC30A1
UBC	SLC2A4
DAXX	SLC2A4
SLC2A4	AKT2
UBC	SLC2A3
PDX1	SLC2A2
KPNA2	SLC2A2
UBC	SLC2A14
UBC	SLC2A12
UBE2I	SLC2A1
UBC	SLC2A1
SUMO2	SLC2A1
SUMO1	SLC2A1
STOM	SLC2A1
PTK2B	SLC2A1
PDIA3	SLC2A1
COPS6	SLC2A1
CANX	SLC2A1
CALR	SLC2A1
UBC	SLC29A2
UBC	SLC29A1
STK19	SLC29A1
UBC	SLC27A3
UBC	SLC27A2
UBC	SLC26A2
UBC	SLC26A11
UBC	SLC25A46
UBC	SLC25A4
TNFRSF1B	SLC25A4
NFKBIA	SLC25A4
UBC	SLC25A31
TNFRSF1A	SLC25A3
UBC	SLC25A24
HN1L	SLC25A20
UBC	SLC25A19
UBC	SLC25A17
PEX19	SLC25A17
UBC	SLC25A15
UBC	SLC25A13
UBC	SLC25A12
UBC	SLC25A11

TNFRSF1B	SLC25A11
UBC	SLC25A10
MDFI	SLC25A10
MAPRE3	SLC25A10
DYNLT1	SLC25A10
DYNLL1	SLC25A10
VCL	SLC25A1
UBC	SLC25A1
TNFRSF1B	SLC25A1
UBC	SLC23A2
ATP6V1B1	SLC23A2
UBC	SLC22A5
POU2AF1	SLC22A2
UBC	SLC22A18
POU2AF1	SLC22A1
CERS2	SLC22A1
UBC	SLC20A2
UBC	SLC20A1
UBC	SLC1A5
TNFRSF1B	SLC1A5
TNFRSF1A	SLC1A5
TBC1D17	SLC1A5
UBC	SLC1A3
TNFRSF1B	SLC1A3
PRKCA	SLC1A1
UBC	SLC19A2
UBC	SLC18B1
UBC	SLC17A5
UBC	SLC16A9
UBC	SLC16A7
UBC	SLC16A3
UBC	SLC16A10
UBC	SLC16A1
UBC	SLC15A4
UBC	SLC12A9
UBC	SLC12A7
UBC	SLC12A6
SLC12A2	OXSR1
UBC	SLC11A2
NDFIP1	SLC11A2
UBC	SLC10A4
USP8	SLBP
EGFR	SLA2
CD247	SLA2

CBL	SLA2
VAV1	SLA
UBC	SKP2
TCF3	SKP2
SMAD4	SKP2
SKP1	SKP2
SKP2	SIRT3
SKP2	RBL2
SKP2	RASSF1
MYC	SKP2
MYBL2	SKP2
SKP2	KPNA1
SKP2	FZR1
FOXO3	SKP2
FOXO1	SKP2
CUL5	SKP2
CUL1	SKP2
COPS6	SKP2
COPS5	SKP2
SKP2	CDKN1C
CDKN1B	SKP2
SKP2	CDC34
SKP2	CDC20
SKP2	CDC14B
SKP2	CCNE1
SKP2	CCNA2
SKP2	BTG2
UBC	SKP1
SKP1	MYC
KDM2A	SKP1
SKP1	FBXO7
SKP1	FBXO32
SKP1	FBXO31
SKP1	FBXO27
SKP1	FBXO25
SKP1	FBXO18
SKP1	FBXL5
SKP1	FBXL2
COPS6	SKP1
SKP1	CDC25A
SKP1	CCNA2
MPHOSPH6	SKIV2L2
UBC	SKIV2L
SKIL	PSMC2

SKIL	FBLN1
SKIL	CDC16
UBC	SKI
SNW1	SKI
SMAD4	SKI
SMAD3	SKI
SMAD2	SKI
SMAD1	SKI
SKIL	SKI
SIN3A	SKI
RARA	SKI
HDAC3	SKI
SKAP1	PTPRC
SKAP1	LYN
LCK	SKAP1
GRB2	SKAP1
FYN	SKAP1
SKA2	SKA1
BLOC1S6	SKA1
AESSIX6	
SUMO2	SIX4
EYA3	SIX4
EYA2	SIX4
TLE1	SIX3
SIX3	PAX6
SIX3	NR4A3
AESSIX3	
UBC	SIVA1
CD27	SIVA1
BCL2L1	SIVA1
LCK	SIT1
VIM	SIRT6
UBC	SIRT6
ELF5	SIRT6
UBC	SIRT3
PEX5	SIRT3
SIRT3	DNM1L
UBC	SIRT1
TP53	SIRT1
HDAC4	SIRT1
UBC	SIRPA
TRIM2	SIRPA
PTPN6	SIRPA
JAK2	SIRPA

CD47	SIRPA
UBC	SIPA1L1
NRXN1	SIPA1L1
UBC	SIN3B
REST	SIN3B
HDAC2	SIN3B
UBC	SIN3A
TP53	SIN3A
SIN3A	TCF3
SIN3A	TAL1
SIN3A	SUDS3
SIN3A	SMARCB1
SIN3A	SMARCA2
SIN3A	SIN3B
SIN3A	SFPQ
SIN3A	SAP30L
SIN3A	SAP18
SIN3A	REST
SIN3A	RBBP4
NCOR2	SIN3A
SIN3A	MTA2
SIN3A	MORF4L1
SIN3A	KLF16
SIN3A	KLF11
SIN3A	ING2
SIN3A	ING1
SIN3A	HTT
SIN3A	HDAC9
SIN3A	HDAC4
HDAC2	SIN3A
SIN3A	ETV6
SIN3A	COPS2
SIN3A	BRMS1
BCL6	SIN3A
UBC	SIMC1
UBC	SIK3
UBC	SIK2
RPN1	SIGIRR
VAV1	SIAH2
SIAH2	UBE2L6
UBC	SIAH2
TRAF2	SIAH2
SIAH2	PSMD4
SIAH2	POU2AF1

UBE2D3	SIAH1
UBC	SIAH1
TNK2	SIAH1
SIAH1	SNCAIP
SLC9A3R2	SIAH1
PUF60	SIAH1
SIAH1	POU2AF1
SIAH1	KIF22
SIAH1	HIPK2
SIAH1	CACYBP
SIAH1	AFF4
DYNLL1	SHROOM3
UBC	SHQ1
UBC	SHPRH
RAD18	SHPRH
UBC	SHPK
UBC	SHOC2
NRAS	SHOC2
HRAS	SHOC2
UBC	SHMT1
UBC	SHKBP1
UBC	SHH
PTCH2	SHH
PSMD7	SHFM1
PSMD4	SHFM1
PSMD3	SHFM1
PSMD14	SHFM1
PSMD13	SHFM1
PSMD11	SHFM1
PSMD1	SHFM1
PSMC2	SHFM1
PSMC1	SHFM1
PCID2	SHFM1
RET	SHC3
NTRK2	SHC3
GRB2	SHC3
EGFR	SHC3
NTRK2	SHC2
NTRK1	SHC2
KDR	SHC2
FLT1	SHC2
VAV1	SHB
JAK3	SHB
NCK1	SHANK3

GRB2 SHANK3
SSTR2 SHANK2
SSTR2 SHANK1
SPTAN1 SHANK1
LPHN1 SHANK1
DNM2 SHANK1
DLG4 SHANK1
BAIAP2 SHANK1
ARHGEF7 SHANK1
VIM SH3YL1
GFAP SH3YL1
UBC SH3RF1
SEMA6A SH3RF1
AKT2 SH3RF1
UBC SH3PXD2B
UBC SH3PXD2A
UBC SH3GLB2
SH3GLB1 SH3GLB2
CCDC158 SH3GLB2
UBC SH3GLB1
DNM2 SH3GLB1
WDR33 SH3GL3
RPL35A SH3GL3
PDCD6IP SH3GL3
HTT SH3GL3
SH3GL3 CD101
SH3GL3 C11orf68
AESSH3GL3
UBC SH3GL2
TCEA2 SH3GL2
SH3GL3 SH3GL2
PDCD6IP SH3GL2
ITCH SH3GL2
ERH SH3GL2
EGFR SH3GL2
DNM2 SH3GL2
ADAM9 SH3GL2
UBC SH3GL1
SH3GL3 SH3GL1
SH3GL2 SH3GL1
SH2D4A SH3GL1
PDCD6IP SH3GL1
UBC SH3BP5
MAPK9 SH3BP5

MAPK10	SH3BP5
UBC	SH3BP4
ZAP70	SH3BP2
YWHAQ	SH3BP2
VAV2	SH3BP2
VAV1	SH3BP2
TNKS2	SH3BP2
TF	SH3BP2
SYK	SH3BP2
PLCG2	SH3BP2
PLCG1	SH3BP2
LAT	SH3BP2
GRB2	SH3BP2
UBC	SH3BGRL3
EGFR	SH3BGRL3
ERBB2	SH2D3A
EGFR	SH2D3A
BCAR1	SH2D3A
LCK	SH2D2A
SLAMF1	SH2D1B
CD84	SH2D1B
LCK	SH2B3
SORBS1	SH2B2
JAK2	SH2B2
GRB2	SH2B2
CBL	SH2B2
NTRK1	SH2B1
KIAA0319	SH2B1
JAK2	SH2B1
GRB2	SH2B1
UBC	SGTA
SGTA	SRGN
SGTA	SPPL2A
SPP1	SGTA
SGTA	SEC61B
PFN2	SGTA
NME3	SGTA
HSPA8	SGTA
GHR	SGTA
EFEMP2	SGTA
BAG6	SGTA
UBC	SGSM3
PDCD6IP	SGSM3
RGMA	SGSM2

PTNSGSM2
PNO1 SGSM2
ILF2 SGSM2
EGFR SGSM2
CHD3 SGSM2
CA12 SGSM2
UBC SGPL1
UBC SGOL1
PPP2R5E SGOL1
PPP2R5B SGOL1
PPP2R5A SGOL1
PPP2R1B SGOL1
UBC SGMS2
UBC SGK1
STUB1 SGK1
SLC9A3R2 SGK1
NEDD4L SGK1
NEDD4 SGK1
MAPK7 SGK1
KPNA2 SGK1
UBC SGCE
SGCG SGCD
UBC SGCB
SGCD SGCB
SGCA SGCB
UBC SFXN3
UBC SFXN2
UBC SFXN1
MFAP4 SFTPD
DCN SFTPD
UBC SFT2D2
SFRP1 WNT1
SFPQ HN1
SFPQ GAS7
ZFP36 SFN
UBC SFN
TP53 SFN
PLEKHF2 SFN
MDM2 SFN
HDAC4 SFN
GPRIN2 SFN
SFNFAM189A2
PHC1 SFMBT1
ACP1 SFMBT1

KRT19	SFI1
UBC	SF3B5
SF3B1	SF3B5
SF3A2	SF3B5
UBC	SF3B4
SF3B4	SS18L1
ILF3	SF3B4
UBC	SF3B3
SF3B3	SF3A3
SF3B3	EFTUD2
CUL2	SF3B3
UBC	SF3B2
THOC1	SF3B2
TCERG1	SF3B2
SUMO2	SF3B2
SF3B4	SF3B2
SF3B3	SF3B2
SF3B1	SF3B2
SF3A2	SF3B2
SF3B2	RBM7
PRPF40A	SF3B2
APBB1	SF3B2
UBC	SF3B1
SF3B1	U2AF2
U2AF1	SF3B1
SF3B1	TOP1
TCERG1	SF3B1
SMAD9	SF3B1
SMAD5	SF3B1
SMAD1	SF3B1
SF3B1	SF3B4
SF3B3	SF3B1
SF3B1	SF3A2
PPP1CA	SF3B1
CCDC85B	SF3A3
UBC	SF3A2
SF3A2	U2AF2
TCERG1	SF3A2
SF3A2	SNRPG
SF3A2	SNRPB2
SF3A2	SMURF2
SF3A2	SMNDC1
SF3B4	SF3A2
SF3B3	SF3A2

SF3A3 SF3A2
SF3A2 SART1
SF3A2 PRPF8
WWOX SF3A1
WBP4 SF3A1
UBC SF3A1
TCERG1 SF3A1
SUMO2 SF3A1
SP1 SF3A1
SF3A3 SF3A1
SF3A2 SF3A1
PRPF40A SF3A1
HNRNPR SF3A1
GAS7 SF3A1
APBB1 SF3A1
UBC SF1
SF1 U2AF2
SF1 TCERG1
SF1 GAS7
SF1 FUS
SF1 EFTUD2
TNK2 SEZ6
UBC SETX
SUMO2 SETX
TOP2A SETMAR
SETDB1 ZFP64
UBE2I SETDB1
UBC SETDB1
SETDB1 TXNDC9
SETDB1 TTR
TRIM28 SETDB1
SETDB1 TRIM16
SETDB1 TRIB3
SETDB1 TRDMT1
SETDB1 TOLLIP
SETDB1 TOB1
SETDB1 TK1
SETDB1 THAP8
SETDB1 TARDBP
SUMO2 SETDB1
SETDB1 SULT1E1
SNIP1 SETDB1
SETDB1 SLC38A3
SKIL SETDB1

SETDB1	SERPINB9
SETDB1	S100A10
SETDB1	RNF10
SETDB1	QTRT1
SETDB1	PSMD11
SETDB1	PRKRA
SETDB1	PPP1R8
SETDB1	PPA1
SETDB1	POLA2
SETDB1	PLEKHA4
SETDB1	PHF10
SETDB1	ORAI2
SETDB1	OLFML3
SETDB1	MOB4
SETDB1	MBD1
SETDB1	MAD2L1BP
SETDB1	KDM1A
HSPB3	SETDB1
SETDB1	HIST1H3A
SETDB1	GSTO1
SETDB1	GIPC2
SETDB1	ERH
SETDB1	DAP
SETDB1	CLSTN1
CHAF1A	SETDB1
SETDB1	CDK4
SETDB1	CCDC106
SETDB1	CBX8
SETDB1	C11orf1
BTBD2	SETDB1
SETDB1	BHLHE40
SETDB1	BAG6
UBC	SETD8
HIST4H4	SETD8
DTL	SETD8
TP53	SETD7
TAF7	SETD7
SETD7	RELA
HIST4H4	SETD7
HIST3H3	SETD7
HIST1H3A	SETD7
DNMT1	SETD7
UBC	SETD6
TRAF2	SETD5

DPPA2 SETD5
CCDC85B SETD5
UBC SETD4
UBC SETD3
WDR82 SETD1B
WDR5 SETD1B
RBBP5 SETD1B
CXXC1 SETD1B
ASH2L SETD1B
WDR82 SETD1A
WDR5 SETD1A
USF1 SETD1A
CXXC1 SETD1A
UBC SESN3
USHBP1 SERTAD3
UBC SERTAD2
TRIM28 SERTAD2
CREBBP SERTAD1
CDK4 SERTAD1
UBQLN1 SERPINI2
MASP2 SERPING1
F2 SERPING1
C1S SERPING1
PLGSERPINF2
SERPINF2 KLK2
PNPLA2 SERPINF1
SERPINE1 VTN
SERPINE1 PLG
SERPINE1 PLAU
SERPINE1 ORM1
F2 SERPIND1
SDC2 SERPINC1
PLGSERPINC1
F2 SERPINC1
SERPINB9 UBR1
SERPINB9 GZMB
PRSS1 SERPINB8
F2 SERPINB8
CAP1 SERPINB8
UBC SERPINB6
KLK2 SERPINB6
TNFRSF1B SERPINB3
CTSK SERPINB3
PLAU SERPINB2

IRF3	SERPINB2
CTSK	SERPINB13
CTSG	SERPINB13
UBC	SERPINB1
PRTN3	SERPINB1
SERPINA5	SEMG2
SERPINA5	KLK2
SERPINA5	FGB
F2	SERPINA5
COPS6	SERPINA5
KLK2	SERPINA3
F5	SERPINA3
CELA1	SERPINA3
APP	SERPINA3
F10	SERPINA10
SERPINA1	PRSS3
SERPINA1	ERLEC1
SERPINA1	CELA1
UBC	SERINC1
UBC	SERBP1
UBC	SEPT9
SEPT7	SEPT9
UBC	SEPT8
SEPT5	SEPT8
UBC	SEPT7
SEPT5	SEPT7
SEPT2	SEPT7
SEPT7	SEPT11
SEPT5	SEPT2
PFN2	SEPT5
PARK2	SEPT5
KAT7	SEPT5
UBC	SEPT3
UBC	SEPT2
EEF1A1	SEPT2
DAZAP2	SEPT2
UBC	SEPT11
SEPT5	SEPT11
SEPT2	SEPT11
UBC	SEPT10
UBC	SEPT1
TOLLIP	SEPT1
UBC	SEPHS1
SEPHS1	C9orf9

C14orf1	SEPHS1
SUMO2	SENP6
SUMO1	SENP6
RUVBL2	SENP6
UBC	SENP3
SENP3	PELP1
SUMO3	SENP2
SUMO1	SENP2
NUP153	SENP2
CDK5RAP2	SENP2
SUMO3	SENP1
SUMO2	SENP1
SUMO1	SENP1
RUVBL2	SENP1
UBC	SEMG1
SEMG2	SEMG1
KLK3	SEMG1
TRIP13	SEMA4G
PTPRC	SEMA4D
UBC	SEMA4C
DLG1	SEMA4C
VEGFA	SEMA3F
UBC	SEMA3F
SLC35G2	SEMA3F
TPST1	SELPLG
SELP	SELPLG
SELE	SELPLG
VCAN	SELP
SELP	SERPING1
SELP	GP1BA
SELP	FUT4
TRIP13	SELENBP1
RPA1	SELENBP1
VCL	SELE
SELE	SERPING1
SELL	SELE
PXN	SELE
PLCG1	SELE
GLG1	SELE
CD44	SELE
VCL	SEL1L3
TNFRSF1A	SEL1L
ERLEC1	SEL1L
UBC	SEH1L

UBC	SEC63
UBC	SEC62
SEC63	SEC62
UBC	SEC61B
SEC61A1	SEC61B
EGFR	SEC61B
BAG6	SEC61B
AMFR	SEC61B
UBC	SEC61A2
TNFRSF1A	SEC61A1
UBC	SEC31A
LMO4	SEC24D
UBC	SEC24C
SEC23A	SEC24B
UBC	SEC23IP
UBC	SEC23B
SEC24D	SEC23B
SEC24C	SEC23B
UBC	SEC23A
TTC1	SEC23A
TMED2	SEC23A
SEC24D	SEC23A
SEC23A	SEC24C
UBC	SEC22B
STX5	SEC22B
SEC13	SEC16B
NFKB2	SEC16A
MAP3K3	SEC16A
UBC	SEC13
TAB2	SEC13
SEC13	SEC31A
UBC	SEC11C
UBC	SEC11A
UBC	SDSL
UBC	SDR39U1
UBC	SDPR
PRKCA	SDPR
PLEKHF2	SDPR
NUDT18	SDPR
PRPF40A	SDK1
UBC	SDHC
UQCRFS1	SDHB
UBC	SDHB
SDHA	SDHB

UBC SDHA
SDHA SLC27A2
ZNF512B SDF4
UBC SDF4
UBC SDF2L1
UBC SDE2
ZCCHC17 SDCBP2
TIFA SDCBP2
PRR13 SDCBP2
DPPA2 SDCBP2
C1orf35 SDCBP2
UBC SDCBP
SOX4 SDCBP
SDC2 SDCBP
SDC1 SDCBP
RNF11 SDCBP
NRXN1 SDCBP
IL5RA SDCBP
GRM3 SDCBP
GRIK2 SDCBP
GRIA4 SDCBP
GRIA1 SDCBP
EPHA7 SDCBP
EFNB2 SDCBP
PRKCA SDC4
CCL5 SDC4
TUBB2A SDC3
TUBA1A SDC3
PTNSDC3
FYN SDC3
CTTN SDC3
SPARC SDC2
UBC SDC1
CCL5 SDC1
VIPR1 SCT
SCTR SCT
UBC SCRN3
UBC SCPEP1
UBC SCP2
UBC SCO2
UBC SCO1
USP2 SCNN1G
UBC SCNN1G
STX1A SCNN1G

SCNN1D	SCNN1G
SCNN1B	SCNN1G
NEDD4L	SCNN1G
NEDD4	SCNN1G
UBC	SCNN1D
COMMD1	SCNN1D
UBC	SCNN1B
SCNN1B	SCNN1D
SCNN1B	SCNN1A
NEDD4L	SCNN1B
NEDD4	SCNN1B
COMMD1	SCNN1B
UBC	SCNN1A
NEDD4	SCNN1A
UBC	SCN5A
NEDD4L	SCN5A
CALM1	SCN5A
SCN1B	SCN2B
SCN1B	ANK3
HK1	SCMH1
UBC	SCLY
FN1	SCGB1A1
UBC	SCFD2
VCL	SCFD1
USP22	SCFD1
USO1	SCFD1
UBC	SCFD1
PPBP	SCFD1
DLST	SCFD1
UBC	SCD5
UBC	SCCPDH
AP3S1	SCARB2
UBC	SCARA3
UBC	SCAPER
SCAND1	ZSCAN20
SCAND1	ZNF202
ZKSCAN7	SCAND1
UBC	SCAMP4
UBC	SCAMP3
EGFR	SCAMP3
UBC	SCAMP2
ST3GAL3	SCAMP2
SCAMP1	SCAMP2
UBC	SCAMP1

SCAMP1	SYNRG
ITSN1	SCAMP1
UBC	SCAF4
SRSF3	SCAF11
SRSF2	SCAF11
UBC	SCAF1
UBC	SC5D
UBC	SBNO1
UBC	SBF2
UBC	SBF1
UBC	SBDS
UBE2I	SATB1
SUMO1	SATB1
SATB1	SMARCA5
PIAS3	SATB1
PIAS1	SATB1
MTA2	SATB1
HDAC1	SATB1
SATB1	CBX4
TGFBR1	SAT2
SKIL	SASH1
USP4	SART3
UBC	SART3
PRPF4	SART3
PRPF3	SART3
MEPCE	SART3
LSM7	SART3
UBC	SARS2
UBC	SARS
UBC	SARNP
DDX39B	SARNP
DDX39A	SARNP
UBC	SARM1
UBC	SAR1B
UBC	SAR1A
HDAC2	SAP30L
UBC	SAP30BP
YY1	SAP30
SIN3A	SAP30
RBP1	SAP30
RBBP4	SAP30
NCOR2	SAP30
MXD1	SAP30
ING1	SAP30

HIST2H2BE	SAP30
HDAC2	SAP30
HDAC1	SAP30
CIR1	SAP30
ARID4A	SAP30
SAP18	RBM39
ACIN1	SAP18
HDAC2	SAP130
UBC	SAMM50
TOMM40	SAMM50
MEOX2	SAMM50
YWHAQ	SAMD4B
YWHAG	SAMD4B
YWHAB	SAMD4B
UBC	SALL2
UBE2I	SALL1
SUMO1	SALL1
RHO	SAG
UBC	SAFB2
SUMO2	SAFB2
CHGB	SAFB2
UBC	SAFB
SUMO3	SAFB
SUMO2	SAFB
SRPK1	SAFB
SAFB	SAFB2
HNRNPD	SAFB
HNRNPA1	SAFB
SAFB	HDAC3
ESR1	SAFB
CYCS	SAFB
CHD3	SAFB
ABHD16A	SAFB
UBE2I	SAE1
UBC	SAE1
UBA2	SAE1
TP53	SAE1
SUMO3	SAE1
SUMO2	SAE1
SUMO1	SAE1
UBC	SACS
COL4A1	SAA2
HTR1A	S1PR3
GNAQ	S1PR3

PDGFRB	S1PR1
HTR1A	S1PR1
UBC	S100PBP
S100A1	S100P
EZR	S100P
AGER	S100P
S100A11	S100B
S100B	S100A1
MAPT	S100B
UBC	S100A9
ILK	S100A9
TP53	S100A8
S100A8	S100A9
S100A8	RAB17
PRMT1	S100A8
S100A8	PPIA
S100A8	PDCD11
S100A8	IGSF21
CHGB	S100A8
C14orf1	S100A8
UBC	S100A6
S100B	S100A6
CACYBP	S100A6
ANXA2	S100A6
UNC119	S100A4
UBC	S100A4
TP53	S100A4
S100A1	S100A4
NOV	S100A4
MYH9	S100A4
BAG6	S100A4
UBC	S100A2
UBC	S100A16
UBC	S100A14
S100A16	S100A14
VCL	S100A13
UBC	S100A13
SYT1	S100A13
PHYHIP	S100A13
FGF1	S100A13
UBC	S100A10
S100A10	PLG
S100A10	PLA2G4C
KCNK3	S100A10

FSHR	S100A10
S100A10	CTSB
COPS6	S100A10
BAD	S100A10
ANXA7	S100A10
S100A10	ANXA2
FKBP1A	RYR3
UBC	RYR2
SRI	RYR2
SMAD5	RYR2
RYR3	RYR2
RYR1	RYR2
PRKAR2A	RYR2
HOMER1	RYR2
FKBP1B	RYR2
CACNA1C	RYR2
AKAP6	RYR2
UBC	RYR1
RYR1	S100A1
PRKG1	RYR1
RYR1	HOMER3
RYR1	HOMER2
RYR1	HOMER1
RYR1	FKBP1A
RYR1	CAMK2G
UBC	RYK
EPHB3	RYK
EPHB2	RYK
YY1	RYBP
UBC	RYBP
RNF2	RYBP
RING1	RYBP
FADD	RYBP
E2F6	RYBP
CSNK2A2	RYBP
UBC	RXRG
RXRG	THRB
RXRG	RARA
RXRG	PPARA
RXRG	NR1H2
CHGB	RXRG
UBC	RXRB
RXRB	THRB
RXRB	RARA

RXRB	NR1H2
NCOA3	RXRB
UBC	RXRA
RXRA	TRIP4
RXRA	THRB
SMAD2	RXRA
RXRA	PRKD2
RXRA	NR1H2
RXRA	NCOR2
RXRA	NCOA4
RXRA	NCOA2
RXRA	MED1
RLN2	RXFP2
INSL3	RXFP2
RLN3	RXFP1
RLN2	RXFP1
NUDT18	RWDD1
DRG1	RWDD1
RUVBL2	TELO2
RUVBL1	VPS72
UCHL5	RUVBL1
UBC	RUVBL1
RUVBL1	TRRAP
TRAF2	RUVBL1
RUVBL1	TNFRSF1A
RUVBL1	TELO2
RUVBL1	TBK1
RUVBL1	TAB2
RUVBL2	RUVBL1
RUVBL1	NFKB2
RUVBL1	NFKB1
RUVBL1	NEDD4
MYC	RUVBL1
RUVBL1	MAP3K14
KAT5	RUVBL1
RUVBL1	INO80E
RUVBL1	INO80
RUVBL1	EP400
RUVBL1	DPCD
RUVBL1	ACTR5
RUVBL1	ACTL6A
RUVBL1	ACTB
UBC	RUNX3
TLE1	RUNX3

CBFB	RUNX3
RUNX1T1	WBP11
SIN3A	RUNX1T1
NCOR2	RUNX1T1
RUNX1T1	HSP90AA1
HDAC1	RUNX1T1
RUNX1T1	GFI1
RUNX1T1	CBFA2T2
RUNX1T1	ABI3
YAP1	RUNX1
WWTR1	RUNX1
UBC	RUNX1
TLE1	RUNX1
TAL1	RUNX1
SMAD3	RUNX1
RUNX1T1	RUNX1
PML	RUNX1
PAX5	RUNX1
KAT6B	RUNX1
KAT6A	RUNX1
JUN	RUNX1
HDAC3	RUNX1
HDAC2	RUNX1
HDAC1	RUNX1
FOS	RUNX1
EP300	RUNX1
CEBPB	RUNX1
CBFB	RUNX1
CBFA2T3	RUNX1
CBFA2T2	RUNX1
RAP2B	RUNDC3A
EIF3E	RUNDC3A
AP2M1	RUNDC3A
AP1M1	RUNDC3A
UBC	RUFY1
RAB4A	RUFY1
UBC	RTTN
WBP11	RTN4IP1
EXOC7	RTN4IP1
UQCRC2	RTN4
UBC	RTN4
SPG21	RTN4
RTN4	RTN4R
RTN4	RTN3

PLEKHF2	RTN4
NAPA	RTN4
LRPAP1	RTN4
RTN4	LRCH4
FXR2	RTN4
RTN4	COL4A3BP
UBC	RTN3
RTN3	PTPN9
PLEKHF2	RTN3
RTN3	COL4A3BP
BCL2L1	RTN1
UBC	RTF1
SUMO2	RTF1
PAF1	RTF1
LEO1	RTF1
CTR9	RTF1
UBC	RTEL1
SMAD4	RTDR1
AP2M1	RTDR1
UBC	RSU1
LIMS1	RSU1
ILK	RSU1
UBC	RSPRY1
UBC	RSF1
UBC	RSC1A1
UBC	RSBN1L
UBC	RSBN1
UBC	RRP1B
UBC	RRP12
MAP3K14	RRP12
AP2M1	RRP12
UBC	RRP1
UBC	RRN3
TAF1C	RRN3
TAF1B	RRN3
POLR1B	RRN3
POLR1A	RRN3
EIF3L	RRN3
UBC	RRM2B
TP53	RRM2B
RRM1	RRM2B
UBC	RRM2
TP53	RRM2
RRM1	RRM2

UBC	RRM1
UBC	RRBP1
PRKD2	RRBP1
UBC	RRAS2
SMAD4	RRAS2
RIN1	RRAS2
RRAS2	RAPGEF4
RAF1	RRAS2
PIK3R1	RRAS2
RRAS2	ACVR1
UBC	RRAGD
RRAGA	RRAGD
RPTOR	RRAGD
UBC	RRAGC
RRAGA	RRAGC
RPTOR	RRAGC
LAMTOR1	RRAGC
UBC	RRAGA
RPTOR	RRAGA
PRKCB	RRAD
PRKCA	RRAD
NME1	RRAD
CALM1	RRAD
RUVBL2	RQCD1
RPTOR	EIF4EBP1
UBC	RPSA
RPS3A	RPSA
RPS3	RPSA
RPSA	RPS28
RPSA	RPS13
RPL18	RPSA
UBC	RPS9
RPSA	RPS9
RPS7	RPS9
RPS6	RPS9
RPS3A	RPS9
RPS3	RPS9
RPS28	RPS9
RPS24	RPS9
RPS23	RPS9
RPS2	RPS9
RPS16	RPS9
RPS14	RPS9
RPS13	RPS9

MAP3K14	RPS9
UBC	RPS8
TBK1	RPS8
SUMO2	RPS8
RPSA	RPS8
RPS9	RPS8
RPS7	RPS8
RPS6	RPS8
RPS5	RPS8
RPS3A	RPS8
RPS3	RPS8
RPS29	RPS8
RPS28	RPS8
RPS27L	RPS8
RPS24	RPS8
RPS23	RPS8
RPS21	RPS8
RPS2	RPS8
RPS16	RPS8
RPS15A	RPS8
RPS14	RPS8
RPS13	RPS8
RPS10	RPS8
RPL7A	RPS8
RPL12	RPS8
NOP56	RPS8
NFKB2	RPS8
NFKB1	RPS8
NEDD8	RPS8
MAP3K14	RPS8
ESR1	RPS8
UBC	RPS7
RPS6	RPS7
RPS3	RPS7
RPS7	RPS2
RPS14	RPS7
RPS7	RPS13
RPS11	RPS7
RPS7	RPS10
PXN	RPS7
MDM2	RPS7
IPO7	RPS7
RPS7	IPO5
RPTOR	RPS6KB1

RPS6	RPS6KB1
PPP2R2A	RPS6KB1
PDK1	RPS6KB1
RPS6KB1	NEK6
MAPT	RPS6KB1
RPS6KB1	MAPK1
CDC42	RPS6KB1
MAPK14	RPS6KA5
HMGN1	RPS6KA5
HIST3H2BB	RPS6KA5
CREB1	RPS6KA5
ATF1	RPS6KA5
MAPK3	RPS6KA2
MAPK1	RPS6KA2
MAP2K1	RPS6KA2
CREB1	RPS6KA2
UBC	RPS6KA1
RPS6KA1	RPS6
RPS6KA1	CREB1
RPS6KA1	CCT4
RPS6	OLFM2
UBC	RPS5
TBK1	RPS5
RPSA	RPS5
RPS7	RPS5
RPS6	RPS5
RPS3A	RPS5
RPS3	RPS5
RPS5	RPS24
RPS5	RPS23
RPS5	RPS2
RPS16	RPS5
RPS5	RPS15A
RPS14	RPS5
RPS5	RPS13
RPS11	RPS5
RPS5	RPL5
NFKB2	RPS5
MAP3K14	RPS5
RPS3A	SAP18
RPS3A	RPS6
RPS3	RPS3A
RPS3A	RPS14
NFKB2	RPS3A

NEDD4	RPS3A
RPS3	TRAF1
RPS3	TNFRSF1A
RPS3	RPS6
RPS3	RPS14
RPS3	RPL8
RPS3	NFKB2
UBC	RPS29
RPS9	RPS29
RPS5	RPS29
RPS3	RPS29
RPS23	RPS29
RPS2	RPS29
RPS16	RPS29
RPS15	RPS29
RPS14	RPS29
RPS29	RPS10
RPS6	RPS28
RPS3	RPS28
RPL24	RPS28
EEF1G	RPS28
UBC	RPS27L
TNFRSF1B	RPS27L
RPS28	RPS27L
RPS13	RPS27L
RPL24	RPS27L
GNB2L1	RPS27L
UCHL3	RPS27A
UBC	RPS27A
SMAD2	RPS27A
SMAD1	RPS27A
MDM2	RPS27A
UBC	RPS27
TNFRSF1B	RPS27
TNFRSF1A	RPS27
TAB2	RPS27
PXN	RPS27
NFKB2	RPS27
RPS27	ENOPH1
VCL	RPS24
UBC	RPS24
RPS7	RPS24
RPS6	RPS24
RPS3A	RPS24

RPS3	RPS24
RPS24	RPS2
RPS14	RPS24
RPS13	RPS24
RPS11	RPS24
RPS24	RPS10
PXN	RPS24
NFKB2	RPS24
MAP3K14	RPS24
UBC	RPS23
RPSA	RPS23
RPS7	RPS23
RPS6	RPS23
RPS3A	RPS23
RPS3	RPS23
RPS28	RPS23
RPS24	RPS23
RPS23	RPS2
RPS15	RPS23
RPS14	RPS23
RPS13	RPS23
RPS11	RPS23
RPL8	RPS23
PXN	RPS23
MAP3K14	RPS23
VCL	RPS21
UBC	RPS21
RPSA	RPS21
RPS5	RPS21
RPS3A	RPS21
RPS3	RPS21
RPS28	RPS21
RPS27L	RPS21
RPS2	RPS21
RPS16	RPS21
RPS21	RPS15A
RPS13	RPS21
VCL	RPS20
UBC	RPS20
RPSA	RPS20
RPS8	RPS20
RPS6	RPS20
RPS3A	RPS20
RPS3	RPS20

RPS28	RPS20
RPS23	RPS20
RPS2	RPS20
RPS20	RPS19
RPS16	RPS20
RPS15A	RPS20
RPS13	RPS20
NEDD8	RPS20
MPHOSPH6	RPS20
MDM2	RPS20
MAP3K14	RPS20
UBC	RPS2
RPS6	RPS2
RPS3A	RPS2
RPS3	RPS2
RPS28	RPS2
RPS14	RPS2
RPS13	RPS2
RPS11	RPS2
RPS2	RPS10
RPL8	RPS2
PRMT3	RPS2
UBC	RPS19
RPS9	RPS19
RPS8	RPS19
RPS7	RPS19
RPS6	RPS19
RPS5	RPS19
RPS3A	RPS19
RPS3	RPS19
RPS28	RPS19
RPS24	RPS19
RPS23	RPS19
RPS2	RPS19
RPS16	RPS19
RPS15A	RPS19
RPS15	RPS19
RPS14	RPS19
RPS13	RPS19
RPS10	RPS19
NFKB2	RPS19
UBC	RPS18
TNFRSF1B	RPS18
TBK1	RPS18

SUMO1	RPS18
RPS5	RPS18
RPS3	RPS18
RPS2	RPS18
RPS15	RPS18
NFKB2	RPS18
MRPL3	RPS18
MRPL2	RPS18
MAP3K14	RPS18
ESR1	RPS18
UBC	RPS16
TNFRSF1B	RPS16
TNFRSF1A	RPS16
RPS16	RPSA
RPS16	RPS7
RPS6	RPS16
RPS3A	RPS16
RPS3	RPS16
RPS16	RPS28
RPS16	RPS24
RPS16	RPS23
RPS16	RPS2
RPS15	RPS16
RPS14	RPS16
RPS16	RPS13
RPS16	RPS10
RPL8	RPS16
RPL18	RPS16
NFKB2	RPS16
MAP3K14	RPS16
VHL	RPS15A
UBC	RPS15A
RPS7	RPS15A
RPS6	RPS15A
RPS3A	RPS15A
RPS3	RPS15A
RPS24	RPS15A
RPS23	RPS15A
RPS2	RPS15A
RPS16	RPS15A
RPS15	RPS15A
RPS14	RPS15A
RPS13	RPS15A
RPS15A	RPS10

NFKB2	RPS15A
NEDD8	RPS15A
MAP3K14	RPS15A
ELF5	RPS15A
DDX39B	RPS15A
UBC	RPS15
RPS3	RPS15
RPS15	RPS2
RPS15	RPS14
RPS15	MRPL3
TAF9	RPS14
SMAD2	RPS14
RPS6	RPS14
MDM2	RPS14
UBC	RPS13
RPS6	RPS13
RPS3A	RPS13
RPS3	RPS13
RPS28	RPS13
RPS14	RPS13
NFKB2	RPS13
MAP3K14	RPS13
UBC	RPS12
RPS8	RPS12
RPS6	RPS12
RPS5	RPS12
RPS3A	RPS12
RPS3	RPS12
RPS28	RPS12
RPS24	RPS12
RPS23	RPS12
RPS2	RPS12
RPS19	RPS12
RPS16	RPS12
RPS15A	RPS12
RPS13	RPS12
RPS11	RPS12
RPL24	RPS12
RPL18A	RPS12
MAP3K14	RPS12
RPS3	RPS11
NFKB2	RPS11
UBC	RPS10
RPS3A	RPS10

RPS3	RPS10
RPS14	RPS10
RPS13	RPS10
RPS11	RPS10
PTTG1	RPS10
PFN2	RPS10
NFKB2	RPS10
MAP3K14	RPS10
HAP1	RPS10
UBC	RPRD2
UBC	RPRD1A
UBC	RPP40
UBC	RPP30
RPP14	RPP30
UBC	RPP25L
UBC	RPN2
EZH2	RPN2
ESR1	RPN2
CLN3	RPN2
RPN1	WSB2
UBC	RPN1
RPN1	OS9
UBC	RPLP1
RPLP1	PFDN1
RPLP1	NUDT21
RPLP1	NSF
RPLP1	C15orf39
RPLP1	BRD7
RPLP1	ASCC2
RPLP1	ANKRD13B
UBC	RPL9
RPL9	RPS5
RPS3	RPL9
RPL9	RPS2
RPS15	RPL9
RPL8	RPL9
RPL9	RPL5
RPL9	RPL19
PXN	RPL9
MAP3K14	RPL9
ESR1	RPL9
RPL8	DUSP23
UBC	RPL7A
RPL7A	RPL36

RPL7A	RPL13
NFKB2	RPL7A
MAP3K14	RPL7A
CCDC47	RPL7A
ZNF7	RPL7
UBC	RPL7
TBK1	RPL7
RPS3	RPL7
PXN	RPL7
NFKB2	RPL7
MAP3K14	RPL7
UPF2	RPL5
UBC	RPL5
TP53	RPL5
RPL5	TNPO1
RPS9	RPL5
RPS3	RPL5
RPS23	RPL5
RPS2	RPL5
RPS16	RPL5
RPS15A	RPL5
RPS14	RPL5
RPS11	RPL5
RPL8	RPL5
RPL5	RPL7
RPL5	RPL36
PXN	RPL5
NOP56	RPL5
NEDD8	RPL5
MDM2	RPL5
MAP3K14	RPL5
IPO7	RPL5
IPO5	RPL5
HSP90AA1	RPL5
EIF5A	RPL5
UBC	RPL4
TRIM9	RPL4
RPS5	RPL4
RPS3	RPL4
RPS2	RPL4
RPL8	RPL4
RPL5	RPL4
RPL19	RPL4
PXN	RPL4

PRSS23	RPL4
PIN1	RPL4
PBX2	RPL4
NOP56	RPL4
MAP3K14	RPL4
EEF1G	RPL4
UBC	RPL3L
UBC	RPL38
TNFRSF1B	RPL38
PXN	RPL38
UBC	RPL37A
UBC	RPL37
UBC	RPL36AL
UBC	RPL36
UBC	RPL35
RPS11	RPL35
PRKAA1	RPL35
MAP3K14	RPL35
EIF1B	RPL35
UBC	RPL34
UBC	RPL32
RPS3	RPL32
RPS2	RPL32
RPS15A	RPL32
RPL9	RPL32
RPL8	RPL32
RPL7	RPL32
RPL5	RPL32
RPL4	RPL32
RPL23	RPL32
RPL19	RPL32
MAP3K14	RPL32
EIF1B	RPL32
UBC	RPL31
SUMO2	RPL31
NOP56	RPL31
NEDD8	RPL31
CDK2	RPL31
UBC	RPL30
RPS5	RPL30
RPS23	RPL30
RPS11	RPL30
RPL18A	RPL30
NFKB2	RPL30

MAP3K14	RPL30
UBC	RPL3
PXN	RPL3
MAP3K14	RPL3
RPL3	EIF1B
UBC	RPL28
PXN	RPL28
UBC	RPL27A
SUMO1	RPL27A
RPS3	RPL27A
RPS2	RPL27A
RPS16	RPL27A
RPL8	RPL27A
RPL5	RPL27A
RPL35	RPL27A
RPL19	RPL27A
RPL12	RPL27A
PRKACB	RPL27A
MAP3K14	RPL27A
UBC	RPL27
RPL19	RPL27
PXN	RPL27
MAP3K14	RPL27
UBC	RPL26
RPS3	RPL26
RPL26	RPL35
MDM2	RPL26
UBC	RPL23
TRAF2	RPL23
TNFRSF1B	RPL23
TNFRSF1A	RPL23
TAB2	RPL23
RPS3	RPL23
RPL8	RPL23
RPL5	RPL23
RPL19	RPL23
RIPK1	RPL23
PXN	RPL23
MDM2	RPL23
MAP3K14	RPL23
RPL23	BCCIP
UBC	RPL22
PXN	RPL22
MAP3K14	RPL22

UBC RPL21
RPL7A RPL21
RPL21 RPL36
RPL17-C18orf32 RPL21
RPL21 RPL13
PXN RPL21
HNRNPA1 RPL21
UBC RPL19
RPS3 RPL19
RPS2 RPL19
RPS15A RPL19
RPS11 RPL19
RPL8 RPL19
RPL5 RPL19
RPL19 RPL13
PXN RPL19
UBC RPL18A
TNK2 RPL18A
RPL18A RPL36
NOP56 RPL18A
NEDD4 RPL18A
AESRPL18A
RPS14 RPL18
RPL24 RPL18
NFKB2 RPL18
RPL18 MAP3K14
RPL17-C18orf32 OXSR1
UBC RPL15
RPL7A RPL15
RPL37A RPL15
RPL3 RPL15
RPL24 RPL15
RPL21 RPL15
RPL13 RPL15
PXN RPL15
NOP56 RPL15
COPS6 RPL15
UBC RPL13A
FBXO18 RPL13A
UBC RPL13
TBK1 RPL13
RPL13 RPL36
RPL24 RPL13
RPL18 RPL13

PXN	RPL13
NFKB2	RPL13
MAP3K14	RPL13
ILK	RPL13
UBC	RPL12
RPS5	RPL12
RPS3	RPL12
RPS2	RPL12
RPL8	RPL12
RPL7A	RPL12
RPL12	RPL5
RPL12	MRPL12
UBC	RPL11
TP53	RPL11
TBK1	RPL11
RPS5	RPL11
RPS3	RPL11
RPS2	RPL11
RPL8	RPL11
RPL5	RPL11
RPL35	RPL11
RPL27A	RPL11
RPL23	RPL11
RPL19	RPL11
PXN	RPL11
NOP56	RPL11
NFKB2	RPL11
NEDD8	RPL11
MRPL12	RPL11
MDM2	RPL11
MAP3K14	RPL11
ESR1	RPL11
UBC	RPL10L
MAP3K14	RPL10L
UBC	RPL10A
RPS5	RPL10A
RPS3	RPL10A
RPS2	RPL10A
RPL8	RPL10A
RPL5	RPL10A
MAP3K14	RPL10A
RAB3B	RPH3A
RAB3A	RPH3A
UBC	RPF1

EXOSC10 RPE
RPAP3 WDR92
RPAP3 URI1
RUVBL2 RPAP3
RUVBL1 RPAP3
RPAP3 POLR2E
RPAP3 PIH1D1
NEDD4 RPAP3
RPAP3 ITCH
NEDD4 RPAP2
ITCH RPAP2
UBC RPAP1
POLR2J RPAP1
UBC RPA3
RPA1 RPA3
XPARPA2
UNG RPA2
UNC119 RPA2
UBC RPA2
RPLP1 RPA2
RPA3 RPA2
RPA1 RPA2
RFWD3 RPA2
RBM48 RPA2
RAD52 RPA2
RAD51 RPA2
PRKDC RPA2
ORC2 RPA2
MEN1 RPA2
MED31 RPA2
MCM3 RPA2
MCM2 RPA2
HERPUD1 RPA2
GOLM1 RPA2
FANCM RPA2
CRMP1 RPA2
COPS6 RPA2
CHD1L RPA2
CCNO RPA2
ATRRPA2
ACP5 RPA2
WRN RPA1
RPA1 TCEA2
RPA1 RAD51

RPA1	FANCM
RPA1	BRIP1
RPA1	ASCC2
APC	RP1
NCOA2	RORA
UBC	ROR1
NGF	ROR1
UBC	ROCK2
UBC	ROCK1
SMAD2	ROCK1
RND3	ROCK1
PFN2	ROCK1
DPYSL2	ROCK1
CDC25A	ROCK1
OLFM2	ROBO2
UBC	ROBO1
UBC	RNPS1
U2AF1	RNPS1
RNPS1	SRSF4
RNPS1	SRSF11
RNPS1	SRPK2
SRPK1	RNPS1
RNPS1	SAP18
PIN1	RNPS1
RNPS1	ACIN1
UBC	RNPEP
SMAD2	RNPC3
UBC	RNH1
ANG	RNH1
UBC	RNGTT
UBC	RNFT1
UBE2N	RNF8
UBE2D3	RNF8
UBC	RNF8
RNF8	NBN
MDC1	RNF8
RNF8	HIST2H2BE
EXOSC2	RNF8
RNF8	CEP44
CCDC85B	RNF8
UBC	RNF44
UBE2W	RNF4
UBE2I	RNF4
RNF4	UBE2D4

UBE2D3	RNF4
UBE2D2	RNF4
UBC	RNF4
RNF4	TRPS1
RNF4	TCF20
TBP	RNF4
SUMO2	RNF4
SUMO1	RNF4
SP1	RNF4
SKIL	RNF4
RNF4	PATZ1
RNF4	HMGA1
RNF4	GSC2
ESR1	RNF4
UBC	RNF38
TP53	RNF38
APP	RNF32
UBC	RNF31
UBA52	RNF31
TNFAIP3	RNF31
TNF	RNF31
STAT2	RNF31
STAT1	RNF31
RBCK1	RNF31
NOD2	RNF31
UBE2W	RNF26
UBE2D3	RNF26
RNF25	UBE2E3
UBE2E1	RNF25
RNF25	UBE2D4
UBE2D3	RNF25
UBE2D2	RNF25
UBC	RNF25
UBC	RNF24
UBC	RNF219
UBC	RNF217
UBC	RNF216
UBC	RNF214
UBC	RNF213
UBC	RNF20
HIST2H2BE	RNF20
RNF2	UBE2D3
UBC	RNF2
TP53	RNF2

RNF2	TFCP2
RNF2	SCMH1
RNF2	RING1
RNF2	PHC1
RNF2	PCGF6
RNF2	PCGF3
RNF2	HIST2H2AA4
RNF2	CBX8
RNF2	CBX7
RNF2	CBX2
UBC	RNF19B
VCP	RNF19A
UBE2L6	RNF19A
UBE2L3	RNF19A
UBC	RNF19A
UBC	RNF187
UBC	RNF185
IFFO1	RNF183
FBP1	RNF183
UBC	RNF170
UBC	RNF169
UBE2N	RNF168
UBC	RNF167
UBC	RNF166
UBC	RNF149
PFN2	RNF146
UBE2L3	RNF144B
UBE2E3	RNF14
UBE2E2	RNF14
UBE2E1	RNF14
UBC	RNF14
SMAD4	RNF14
SMAD1	RNF14
NCOA4	RNF14
FAM46A	RNF14
VHL	RNF139
UBC	RNF139
INSIG1	RNF139
COPS5	RNF139
UBC	RNF130
RNF130	ARPC4-TTLL3
UBC	RNF13
UBC	RNF126
BAG6	RNF126

UBC	RNF125
UBC	RNF122
UBC	RNF115
UBC	RNF114
RNF114	AK5
UBE2W	RNF111
UBE2I	RNF111
UBE2E3	RNF111
UBE2E1	RNF111
UBE2D4	RNF111
UBE2D3	RNF111
UBE2D2	RNF111
UBE2D1	RNF111
UBC	RNF111
SUMO2	RNF111
SUMO1	RNF111
SMURF2	RNF111
SMAD6	RNF111
SMAD3	RNF111
SMAD2	RNF111
SKIL	RNF111
RNF111	SKI
PML	RNF111
RNF11	ZFYVE16
RNF11	UBQLN4
UBE2N	RNF11
RNF11	UBE2D3
UBC	RNF11
RNF11	TOM1L2
RNF11	TNIP1
RNF11	TNFAIP3
RNF11	TAX1BP1
RNF11	STAM2
RNF11	SPRTN
SNRNP70	RNF11
RNF11	SMURF2
RNF11	RPS27A
RNF11	RNF216
RNF11	RNF168
RNF11	PDCD6IP
RNF11	OPTN
RNF11	NEDD4L
RNF11	NEDD4
RNF11	ITCH

RNF11	HERC1
RNF11	GGA3
RNF11	EPN3
RNF11	EPN1
RNF11	CBLB
CALCOCO2	RNF11
RNF11	AUP1
RNF11	ASCC2
RNF11	ANKRD13A
STAMBP	RNF103-CHMP3
SMAD5	RNF103-CHMP3
SMAD1	RNF103-CHMP3
CHMP4B	RNF103-CHMP3
PDE6D	RND1
ABCE1	RNASEL
UBC	RNASEH2C
UBC	RNASEH2B
UBC	RNASEH2A
RNH1	RNASE1
SMAD4	RMND5B
UBC	RMND5A
SMAD9	RMND5A
GID8	RMND5A
UBC	RMI1
TOP3A	RMI1
RMI2	RMI1
FANCM	RMI1
BLM	RMI1
YWHAG	RMDN3
YWHAB	RMDN3
UBC	RLF
VCL	RIT2
SMAD2	RIT1
RLF	RIT1
PTN	RIT1
KLHL12	RIT1
IFRD1	RIT1
AP2A1	RIT1
VIM	RIPK3
RIPK3	TUBB4A
RIPK3	TUBB2B
TUBB	RIPK3
TRAF2	RIPK3
RIPK3	TPM1

TNFRSF1A	RIPK3
RIPK3	TIMM50
RIPK3	TICAM1
RIPK3	TIAF1
RIPK3	SPTBN1
RIPK3	SPECC1L
RIPK3	SLC25A3
RIPK3	SCIN
RUVBL2	RIPK3
RUVBL1	RIPK3
RIPK3	RPS27
RIPK3	RPL23
RIPK3	RIPK1
RAI14	RIPK3
RIPK3	PPP1R9B
PPP1CC	RIPK3
RIPK3	MYO1D
RIPK3	MYO1B
RIPK3	MYL6B
RIPK3	MYL12A
MYH10	RIPK3
RIPK3	IQGAP1
HSPA8	RIPK3
HSPA5	RIPK3
RIPK3	HSPA1L
RIPK3	HSP90AA1
HNRNPH1	RIPK3
HNRNPA1	RIPK3
RIPK3	HADHA
RIPK3	GNG12
RIPK3	GNB4
RIPK3	GNB2
RIPK3	GNAI3
FADD	RIPK3
RIPK3	DDX5
DAPK1	RIPK3
RIPK3	CORO1C
RIPK3	CLTC
RIPK3	CDC37
RIPK3	CCT7
CCT5	RIPK3
RIPK3	CAPZB
CALM1	RIPK3
C1QBP	RIPK3

RIPK3	ATAD3A
RIPK3	AIF1L
RIPK3	ACTN4
VIM	RIPK2
UBC	RIPK2
RIPK2	TUBB4A
RIPK2	TUBB2B
TUBB	RIPK2
TRAF6	RIPK2
TRAF2	RIPK2
RIPK2	TRAF1
TNFRSF1A	RIPK2
SMAD4	RIPK2
RIPK2	NOD2
RIPK2	NOD1
RIPK2	MYO1D
RIPK2	MYH9
MYH10	RIPK2
RIPK2	MAP3K7
HSPA8	RIPK2
RIPK2	HSPA6
RIPK2	HSPA2
RIPK2	HSPA1L
RIPK2	HSP90AA1
RIPK2	ENO1
RIPK2	CFLAR
RIPK2	CD40
RIPK2	CASP1
RIPK2	CARD16
CALM1	RIPK2
RIPK2	BIRC3
RIPK2	BIRC2
UBE2N	RIPK1
UBC	RIPK1
RIPK1	TUBB4A
TUBB	RIPK1
TRAF2	RIPK1
TRAF1	RIPK1
TNFRSF1A	RIPK1
RIPK1	TNFRSF10B
RIPK1	TNFRSF10A
TNFAIP3	RIPK1
RIPK1	TNF
RIPK1	TIMM50

RIPK1	TAX1BP1
TAB2	RIPK1
RIPK1	RNF216
RIPK1	OPTN
RIPK1	MAP3K14
HSPA8	RIPK1
HSPA5	RIPK1
RIPK1	HSPA1L
FADD	RIPK1
EGFR	RIPK1
RIPK1	CRADD
RIPK1	CFLAR
RIPK1	CCDC50
BIRC2	RIPK1
UBC	RIOK3
UBC	RIOK2
RIOK2	DIMT1
RIOK2	BYSL
UBC	RIOK1
UBC	RING1
RING1	PHC1
RING1	PCGF6
RING1	PCGF3
RING1	E2F6
RING1	CBX8
RING1	CBX7
YWHAE	RIN1
UBC	RIN1
RIN1	HRAS
ERBB3	RIN1
EGFR	RIN1
RIN1	ABL2
YWHAH	RIMS2
RAPGEF4	RIMS2
RABAC1	RIMS2
RAB8A	RIMS2
RAB3D	RIMS2
RAB3B	RIMS2
RAB3A	RIMS2
RAB7A	RILP
UBC	RIF1
SUMO2	RIF1
PPP1CC	RIF1
PPP1CA	RIF1

UBC	RICTOR
PRR5L	RICTOR
PREX1	RICTOR
MLST8	RICTOR
MAPKAP1	RICTOR
GNAQ	RIC8B
UBC	RIC8A
RIC8A	GNAQ
VIM	RIBC2
TRIM23	RIBC2
TRAF2	RIBC2
RIBC2	RAD54B
RIBC2	NRBP1
NINL	RIBC2
MTUS2	RIBC2
KRT15	RIBC2
JUP	RIBC2
RIBC2	GTPBP3
RIBC2	GFAP
EFEMP2	RIBC2
CCDC85B	RIBC2
KRT15	RHPN1
EFEMP2	RHPN1
CALCOCO2	RHPN1
PAK1	RHOU
SMAD2	RHOJ
TGFBR1	RHOH
KCNH2	RHOH
ARHGDIB	RHOH
ARHGDIA	RHOH
VAV3	RHOG
VAV2	RHOG
UBC	RHOG
TGFBR1	RHOG
SMAD4	RHOG
SMAD1	RHOG
MAP3K11	RHOG
KTN1	RHOG
ARHGDIG	RHOG
UBC	RHOD
RHOC	RTKN
ARHGAP1	RHOC
UBC	RHOBTB3
CUL3	RHOBTB3

SMAD2	RHOBTB1
SMAD1	RHOBTB1
ACVR1	RHOBTB1
RHPN2	RHOB
RHOB	PDE6D
TXNL4A	RHEBL1
SMAD2	RHEBL1
SMAD1	RHEBL1
RPTOR	RHEB
RHEB	PDE6D
EFNB3	RHBDL2
EFNB2	RHBDL2
UBC	RHBDD3
UBC	RHBDD2
RGS7BP	RGS9
RGS7BP	GNB5
RGS7	GNB5
GNAQ	RGS5
PTAFR	RGS4
GNAQ	RGS4
GNAI2	RGS4
ERBB3	RGS4
YWHAQ	RGS3
YWHAE	RGS3
YWHAB	RGS3
UBC	RGS3
HIPK3	RGS3
GNAI3	RGS3
GNA11	RGS3
GNAI3	RGS20
WDR74	RGS2
PRKCSH	RGS2
PRKCG	RGS2
PRKCB	RGS2
PRKCA	RGS2
PARK2	RGS2
NIPSNAP1	RGS2
NINL	RGS2
LRFN1	RGS2
HSPA8	RGS2
GNAS	RGS2
GNAQ	RGS2
GNA15	RGS2
DYNLL1	RGS2

DDR1	RGS2
CTSB	RGS2
COPB2	RGS2
COPB1	RGS2
ADCY5	RGS2
UBC	RGS19
PRKCA	RGS19
GNAQ	RGS18
GNAI3	RGS18
GNAI2	RGS18
EGFR	RGS16
TRAF2	RGS14
ITGB7	RGS12
ITGB3	RGS12
ITGB1	RGS12
GRM5	RGS12
GRM2	RGS12
GNAI3	RGS12
GNAI2	RGS12
GNAI1	RGS12
CXCR2	RGS12
GNB5	RGS11
UBC	RGS10
GNAI3	RGS10
UBC	RGPD4
UBC	RGPD3
UBC	RGPD2
UBC	RGP1
HRAS	RGL4
RGL2	RAP1B
KAT7	RGL2
KAT5	RGL2
RGL2	HRAS
EEF1G	RGL2
RGL2	CCDC90B
RFXANK	RFXAP
RFX5	RFXAP
UBC	RFXANK
RFXANK	RFX5
HDAC4	RFXANK
CIITA	RFXANK
SS18L1	RFX6
LMO3	RFX6
CCNK	RFX6

CIITA	RFX5
TRAF2	RFX3
RFX6	RFX3
RFX2	RFX3
RFX6	RFX2
RFX2	RFX4
UBE2D3	RFWD3
UBC	RFWD3
RFWD3	RPA3
RPA1	RFWD3
UBC	RFTN1
UBC	RFT1
DNAJB9	RFK
RBM48	RFC5
UBC	RFC4
RFC5	RFC4
RFC3	RFC4
RFC2	RFC4
RFC1	RFC4
RAD17	RFC4
HDAC1	RFC4
CHTF18	RFC4
UBC	RFC3
RFC1	RFC3
RFC3	RAD17
CHTF18	RFC3
UBC	RFC2
RFC5	RFC2
RFC1	RFC2
PRKAR1A	RFC2
CHTF18	RFC2
UBC	RFC1
RFC5	RFC1
POLD1	RFC1
UBC	REXO4
UBC	REXO2
REV1	REV3L
MAD2L2	REV3L
MAD2L1	REV3L
UBC	REV1
POLH	REV1
REV1	MAD2L2
UBC	RETSAT
SF3A2	RETN

UBC RET
STAT3 RET
PLCG1 RET
RETNRTN
GRB2 RET
RETGFRA1
RETDOK5
RETDOK1
EHMT2 REST
UBC RER1
SH3GL3 RER1
HAP1 RER1
M6PR REN
KCTD15REN
CTSB REN
RELN LRP8
UBC RELL1
RELB USP7
UBC RELB
RELB TUBB4A
TUBB RELB
RELB TIMM50
RELB SMARCE1
SMARCD2 RELB
SMARCC1 RELB
RELB SMARCB1
SFPQ RELB
RUVBL1 RELB
RELB RPS5
RELB RPS11
RELB RPS10
RELB RPL30
RELB RELA
PRKDC RELB
RELB PFKL
NUDT21RELB
RELB NQO2
RELB NFKBIA
NFKB2 RELB
RELB NFKB1
RELB KPNA6
RELB KPNA2
RELB KPNA1
HSPA9 RELB

HSPA8 RELB
RELB HSPA6
HSPA5 RELB
RELB HSPA1L
RELB HSD17B4
DSP RELB
RELB DPF2
RELB DDX1
RELB DAXX
CPSF6 RELB
RELB CLTC
RELB CHUK
CALM1 RELB
C1QBP RELB
ACTL6A RELB
UBC RELA
RELA TWIST1
TUBB4A RELA
RELA TUBB2B
TRIP4 RELA
TP53BP2 RELA
TNIP2 RELA
TLE1 RELA
TIMM50 RELA
TERT RELA
TBP RELA
TBK1 RELA
STAT3 RELA
SP1 RELA
RELA SETD6
RUVBL2 RELA
RUVBL1 RELA
RPS3 RELA
RELA RPL23
RPL18 RELA
RFC1 RELA
REL RELA
RELA PYCR2
PPP4C RELA
RELA PPP1R13L
RELA PPARA
PIAS1 RELA
PGR RELA
PARP1 RELA

NPM1 RELA
RELA NFKBIB
NFKBIA RELA
NFKB2 RELA
NFKB1 RELA
NCOR2 RELA
MYC RELA
MEN1 RELA
MAP3K8 RELA
LGALS3BP RELA
RELA LARS
KPNA2 RELA
IQGAP2 RELA
ILF2 RELA
HSPH1 RELA
RELA HEXIM1
HDAC2 RELA
HDAC1 RELA
GTF2B RELA
GCN1L1 RELA
FOS RELA
ETHE1 RELA
ESR1 RELA
EFTUD2 RELA
EEF1A2 RELA
RELA DPF2
DHX9 RELA
DDX21 RELA
COMMD1 RELA
CHUK RELA
CEBPB RELA
RELA CARM1
CALM1 RELA
BRCA1 RELA
AES RELA
REL TNIP2
SP1 REL
PPP4C REL
REL NFKBIB
NFKBIA REL
NFKB2 REL
NFKB1 REL
MAP3K8 REL
HSPA8 REL

SDC2	REG3A
REG1A	REG3A
FN1	REG3A
BAG6	REG1B
FAM160A2	REEP6
UBC	REEP5
E2F6	REEP5
UBC	REEP4
UBC	REEP3
RECQL5	TOP3A
RECQL5	STAMBP
PLK1	RECQL5
RECQL5	MTSS1
RECQL5	MAPK8IP1
EEF1G	RECQL5
CREB1	RECQL5
UBC	RECQL
KPNA4	RECQL
KPNA2	RECQL
MMP9	RECK
UBC	RDX
TSC1	RDX
SLC9A3R2	RDX
SLC9A3R1	RDX
ICAM2	RDX
EZR	RDX
CPNE4	RDX
CPNE2	RDX
CPNE1	RDX
ARHGDIA	RDX
ABCC2	RDX
WRN	RDM1
RLBP1	RDH5
UBC	RDH14
UBC	RDH13
UBC	RDH12
UBC	RDH10
UBC	RCOR3
KRT15	RCOR3
GSE1	RCOR3
ZNF217	RCOR1
UBC	RCOR1
SUMO2	RCOR1
SNAI1	RCOR1

REST	RCOR1
PHF21A	RCOR1
KDM1A	RCOR1
HDAC2	RCOR1
HDAC1	RCOR1
ESCO2	RCOR1
CTBP1	RCOR1
UBC	RCN3
SUFU	RCN3
HID1	RCN3
UBC	RCN2
TRAF6	RCN2
TRAF1	RCN2
TNFRSF1B	RCN2
TNFRSF1A	RCN2
RIPK1	RCN2
MAP3K1	RCN2
GABARAPL2	RCN2
BAG6	RCN2
UBC	RCN1
UBC	RCL1
UBC	RCHY1
TP53	RCHY1
RCHY1	POLH
MDM2	RCHY1
KAT5	RCHY1
RCHY1	GORAB
CDKN1B	RCHY1
RCHY1	AIG1
UBC	RCCD1
UBC	RCC2
PRPF40A	RCC2
COPS4	RCBTB2
PPP3CA	RCAN2
UBC	RCAN1
PPP3CA	RCAN1
EYA2	RBPMS
SNW1	RBPJ
SND1	RBPJ
RBPJ	NOTCH3
NCOR2	RBPJ
KAT2B	RBPJ
RBPJ	CIR1
ALB	RBP4

ING1	RBP1
UBC	RBMXL2
TRA2B	RBMXL2
UBC	RBMS1
UBC	RBM4B
RBM48	SERPINB9
RBM48	PSMD11
RBM48	NUDT21
RBM48	MPHOSPH6
RBM48	MAP7D1
BTBD2	RBM48
UBC	RBM42
UBC	RBM4
SMAD5	RBM4
UBC	RBM28
SUMO2	RBM28
NOP56	RBM28
ESR1	RBM28
UBC	RBM27
UBC	RBM26
SUMO2	RBM26
UBC	RBM25
RBM39	RBM25
UBC	RBM23
SRPK2	RBM23
PRMT5	RBM23
UBC	RBM22
SF3B1	RBM17
SF3A2	RBM17
SF1	RBM17
PSMD14	RBM15B
NXF1	RBM15
ALYREF	RBM15
UBC	RBM14-RBM4
SUMO2	RBM12B
UBC	RBM12
TBC1D4	RBM12
UBC	RBL2
TFDP1	RBL2
SNW1	RBL2
RAF1	RBL2
PHB	RBL2
LIN9	RBL2
E2F5	RBL2

CCNE1	RBL2
CCNA2	RBL2
UBC	RBL1
TFDP1	RBL1
SNW1	RBL1
SMAD4	RBL1
SMAD2	RBL1
CCNA2	RBL1
DAZAP2	RBFOX2
ATXN1	RBFOX2
ATXN2	RBFOX1
UBE2L3	RBCK1
UBE2D3	RBCK1
UBC	RBCK1
TNFAIP3	RBCK1
RBCK1	TNF
PSMD4	RBCK1
PRKCB	RBCK1
NOD2	RBCK1
IREB2	RBCK1
ESR1	RBCK1
UBC	RBBP9
RBL2	RBBP9
WDYHV1	RBBP8
RBL2	RBBP8
RBL1	RBBP8
RB1	RBBP8
PIN1	RBBP8
LMO4	RBBP8
LIMS1	RBBP8
IKZF1	RBBP8
HID1	RBBP8
FXR2	RBBP8
CTBP1	RBBP8
BRCA1	RBBP8
ATM	RBBP8
AATF	RBBP8
UBC	RBBP6
WDR5	RBBP5
USF1	RBBP5
UBC	RBBP5
RBBP5	SETD1A
RB1	RBBP5
PAXIP1	RBBP5

NCOA6 RBBP5
MEN1 RBBP5
ESR1 RBBP5
RBBP5 CXXC1
CTNNB1 RBBP5
RBBP4 LIN9
RBBP4 ING1
CHAF1A RBBP4
UBC RB1CC1
SMARCB1 RB1CC1
USP4 RB1
RB1 UBTF
UBC RB1
RB1 TRIP11
TRAP1 RB1
TOP2A RB1
TMPO RB1
THOC1 RB1
RB1 TGM2
TFDP1 RB1
TCF3 RB1
TBP RB1
RB1 TAL1
RB1 SPIB
SP1 RB1
RB1 SNAPC3
RB1 SERPINB2
RING1 RB1
RB1 RBBP9
RBBP4 RB1
RAF1 RB1
RB1 PRDM2
PPP1CA RB1
PPIA RB1
RB1 PIK3R3
PIK3R1 RB1
PHB RB1
RB1 PELP1
RB1 MYOD1
RB1 MRFAP1
RB1 MORF4L1
MDM2 RB1
RB1 MCM7
MAPK9 RB1

MAPK14	RB1
LMNA	RB1
LIN9	RB1
RB1	KDM5B
KAT2B	RB1
RB1	INS
HSPA8	RB1
RB1	HDAC3
HDAC2	RB1
HDAC1	RB1
RB1	HBP1
GTF3C2	RB1
RB1	GNB2L1
RB1	FRK
FOS	RB1
ENC1	RB1
RB1	EID1
RB1	E2F3
E2F2	RB1
DNMT1	RB1
CTBP1	RB1
RB1	CEBPE
RB1	CEBPB
RB1	CEBPA
CDK6	RB1
CDK4	RB1
RB1	CDK3
RB1	CCNT2
CCNE1	RB1
RB1	CCNB1
CCNA2	RB1
RB1	CBX4
CASP7	RB1
CASP3	RB1
BRCA1	RB1
RB1	ARID4A
RB1	ARID3B
ABL1	RB1
PAX6	RAX
UBC	RASSF2
UBC	RASSF1
SUV39H2	RASSF1
RASSF1	SAV1
RASSF1	RASSF5

MAP1S	RASSF1
RASSF1	HRAS
CDC20	RASSF1
SMAD2	RASL12
SMAD1	RASL12
UBC	RASIP1
PRKCB	RASGRP3
USP8	RASGRF1
TNK2	RASGRF1
TNK2	RASGEF1C
UBC	RASEF
SMAD2	RASD2
SMAD1	RASD2
PLSCR1	RASD1
GNAI2	RASD1
UBC	RASAL2
RASAL2	NFKBIB
UBC	RASA4B
UBC	RASA3
HCK	RASA3
GNB2L1	RASA3
GNA12	RASA2
RASA1	YES1
RASA1	WWP2
UBC	RASA1
RASA1	RAB5A
RASA1	PTPRC
RASA1	PTK2B
RASA1	PLCG1
PIK3R1	RASA1
RASA1	PDGFRB
RASA1	PDE6D
RASA1	NCK1
RASA1	MAP4K4
RASA1	LYN
KHDRBS1	RASA1
RASA1	HTT
RASA1	HRAS
RASA1	HCK
RASA1	GNB2L1
RASA1	G3BP1
RASA1	FES
ERBB4	RASA1
ERBB3	RASA1

RASA1	EPHB3
RASA1	EPHB2
EGFR	RASA1
RASA1	DOK2
RASA1	DOK1
CSF1R	RASA1
RASA1	CAV1
RASA1	BCR
RASA1	ARHGAP5
RASA1	ARHGAP35
RASA1	ARHGAP1
RASA1	ANXA6
ABL1	RASA1
UBC	RARS
TNFRSF1A	RARS
TANK	RARS
NFKB2	RARS
MAP3K14	RARS
LARS	RARS
UBC	RARRES3
MAVS	RARRES3
RXRG	RARG
RXRA	RARG
NCOR2	RARG
NCOA3	RARG
SMAD2	RARB
RXRG	RARB
RXRB	RARB
PRKD2	RARB
NCOR2	RARB
NCOA1	RARB
UBC	RARA
RARA	TRIP4
SNW1	RARA
RXRA	RARA
RARA	NKX2-1
NCOR2	RARA
NCOA6	RARA
NCOA3	RARA
NCOA2	RARA
MED1	RARA
RARA	KAT2B
SMAD1	RAPSN
MUSK	RAPSN

KHDRBS1 RAPSN
GRB2 RAPSN
DAG1 RAPSN
RAP1A RAPGEF5
UBC RAPGEF2
RAP2B RAPGEF2
RAP1A RAPGEF2
PDGFRA RAPGEF1
HRAS RAPGEF1
HCK RAPGEF1
GRB2 RAPGEF1
CRKL RAPGEF1
CRK RAPGEF1
CBL RAPGEF1
BCAR1 RAPGEF1
ABL1 RAPGEF1
RAP2B PDE6D
UBC RAP2A
SMAD1 RAP2A
RAP2A RGS14
RAP2A RAPGEF6
RAF1 RAP2A
RAP1B RAP1GAP
UBC RAP1A
RAP1A RGS14
RASSF5 RAP1A
RAP1A RAPGEF6
RAF1 RAP1A
PDE6D RAP1A
UBC RANGRF
RANBP1 RANGRF
RAN RANGRF
UBC RANGAP1
SUMO1 RANGAP1
RANGAP1 IPO8
UCHL1 RANBP9
UBC RANBP9
TFRC RANBP9
SMAD9 RANBP9
SMAD3 RANBP9
SMAD2 RANBP9
S100A7 RANBP9
RAN RANBP9
PPP1CC RANBP9

PPP1CA	RANBP9
OPRM1	RANBP9
MET	RANBP9
ITGB2	RANBP9
ITGAL	RANBP9
HIPK2	RANBP9
GID8	RANBP9
DYRK1B	RANBP9
DISC1	RANBP9
CALB1	RANBP9
UBC	RANBP6
SMAD2	RANBP6
XPO1	RANBP3
UBC	RANBP3
RCC1	RANBP3
RAN	RANBP3
UBC	RANBP17
UBC	RANBP1
RAN	XPOT
UBC	RAN
RAN	TNPO1
RAN	TGFBR1
SMAD2	RAN
SMAD1	RAN
RAN	RCC1
RAN	RANGAP1
RAN	RANBP1
RAN	NXF1
RAN	IPO7
RAN	IPO5
RAN	IPO13
RAN	IPO11
ESR1	RAN
ABL1	RAN
PDCD6IP	RAMP3
HSPB3	RAMP3
CALCRL	RAMP3
CALCRL	RAMP2
CALCRL	RAMP1
UBC	RALY
SPG7	RALY
PIK3CD	RALY
HNRNPH1	RALY
HNRNPD	RALY

HNRNPC	RALY
RRAS2	RALGDS
RRAS	RALGDS
RIT1	RALGDS
RAP2B	RALGDS
RAP2A	RALGDS
RAP1B	RALGDS
RAP1A	RALGDS
MRAS	RALGDS
KRAS	RALGDS
HRAS	RALGDS
ARRB2	RALGDS
ARRB1	RALGDS
UBC	RALGAPB
UBC	RALBP1
RALBP1	TAX1BP1
RALBP1	RALB
RALBP1	RALA
RALBP1	CCL20
UBC	RALB
UBC	RALA
SMAD4	RALA
HRAS	RALA
EXOC8	RALA
EXOC1	RALA
CALM1	RALA
UBC	RAI14
TESC	RAI1
PIN1	RAI1
RAG2	RAG1
KPNA2	RAG1
KPNA1	RAG1
YWHAE	RAF1
VAV1	RAF1
UBC	RAF1
RAF1	STUB1
RAF1	SHOC2
RAF1	SGK1
RAF1	SFN
RAF1	RRAS
RAF1	RHEB
RAF1	PRKCE
RAF1	PRKAA1
RAF1	PHB

RAF1	PDGFRB
RAF1	NRAS
MYC	RAF1
RAF1	MAPK7
RAF1	MAP3K5
RAF1	MAP2K2
RAF1	MAP2K1
LCK	RAF1
RAF1	JAK1
RAF1	HSPA8
RAF1	HRAS
RAF1	FYN
RAF1	CFLAR
RAF1	CDC25A
UBC	RAE1
NXF1	RAE1
NUP98	RAE1
COPS6	RAE1
YWHAB	RADIL
ASCC2	RADIL
TOPBP1	RAD9A
RAD17	RAD9A
RAD1	RAD9A
HUS1B	RAD9A
HUS1	RAD9A
HDAC1	RAD9A
DNAJC7	RAD9A
CLSPN	RAD9A
CHEK2	RAD9A
BCL2L1	RAD9A
ATM	RAD9A
ABL1	RAD9A
UBC	RAD54L2
SUMO2	RAD54L2
SUMO1	RAD54L2
RAD54B	ZNF580
UBC	RAD54B
RAD54B	TRAPPC6A
RAD51	RAD54B
WRN	RAD52
RAD52	RPA3
RPA1	RAD52
RAD51	RAD52
EEF1G	RAD52

RAD51D	BLM
XRCC3	RAD51C
XRCC2	RAD51C
UBC	RAD51C
RAD51D	RAD51C
RAD51	RAD51C
RAD51D	RAD51B
RAD51C	RAD51B
HIST1H3A	RAD51B
HIST1H2BD	RAD51B
RAD51	RAD51AP1
DMC1	RAD51AP1
RAD51	RAD54L
RAD51	PALB2
UBC	RAD50
TERF2IP	RAD50
TERF2	RAD50
SUMO2	RAD50
RINT1	RAD50
PAXIP1	RAD50
NBN	RAD50
MRE11A	RAD50
MDC1	RAD50
H2AFX	RAD50
CSNK1E	RAD50
BRCA1	RAD50
XPC	RAD23B
UBC	RAD23B
RAD23B	TDG
RAD23A	RAD23B
PSMD4	RAD23B
PSMD13	RAD23B
PSMD1	RAD23B
PSMC2	RAD23B
RAD23B	NUBP2
NPLOC4	RAD23B
ATXN3	RAD23B
XPC	RAD23A
UBC	RAD23A
TRAF2	RAD23A
TP53	RAD23A
RAD23A	PSMD4
RAD23A	PSMD1
PSMC2	RAD23A

RAD23A	PDE6D
EEF1A1	RAD23A
ATXN3	RAD23A
WAPAL	RAD21
UBC	RAD21
SUMO2	RAD21
STAG1	RAD21
SMC3	RAD21
SMARCA5	RAD21
PRKDC	RAD21
PDS5B	RAD21
PDS5A	RAD21
PCNA	RAD21
CDCA5	RAD21
CAPN1	RAD21
RFC5	RAD17
RFC2	RAD17
RFC1	RAD17
RAD1	RAD17
POLE	RAD17
RAD17	HUS1
ATM	RAD17
VAV1	RACGAP1
RACGAP1	RND2
KIF23	RACGAP1
UBC	RAC3
NRBP1	RAC3
HNF1A	RAC3
ESR1	RAC3
ARHGDIB	RAC3
ARHGDIA	RAC3
UBC	RAC2
SMAD4	RAC2
SMAD1	RAC2
ICMT	RAC2
DOCK2	RAC2
CDC42	RAC2
ARHGDIA	RAC2
ACTB	RAC2
WASF1	RAC1
VAV1	RAC1
UBC	RAC1
TUBA4A	RAC1
TRIO	RAC1

TIAM1 RAC1
SYNJ2 RAC1
SH3RF1 RAC1
SET RAC1
RAP1GDS1 RAC1
PRKCA RAC1
PLXNB1 RAC1
PIK3R1 RAC1
PARD6G RAC1
PAK2 RAC1
PAK1 RAC1
NOXA1 RAC1
NOS2 RAC1
NCKAP1 RAC1
NCK1 RAC1
NCF2 RAC1
MCF2L RAC1
IQGAP2 RAC1
IQGAP1 RAC1
IL1RAP RAC1
HACE1 RAC1
DOCK2 RAC1
DOCK1 RAC1
CYFIP1 RAC1
CYBA RAC1
BAIAP2 RAC1
ARHGEF4 RAC1
ARHGEF2 RAC1
ARHGDIB RAC1
ARHGDIA RAC1
ARHGAP32 RAC1
ARHGAP31 RAC1
ARHGAP15 RAC1
ARFIP2 RAC1
ACTB RAC1
UBC RABL3
CEP19 RABL2B
UBC RABGGTB
RAB7A RABGGTB
RABGGTB PTP4A2
UBC RABGGTA
YWHAG RABGEF1
YWHAB RABGEF1
UBC RABGEF1

RNF11	RABGEF1
RABEP1	RABGEF1
RAB5A	RABGEF1
GGA1	RABGEF1
RAB6A	RABGAP1
ITGB1	RABGAP1
GABARAPL1	RABGAP1
UBC	RABEP2
RAB4A	RABEP1
RABEP1	EEA1
AP1G1	RABEP1
WDYHV1	RABAC1
VTA1	RABAC1
VIM	RABAC1
VAMP2	RABAC1
UBC	RABAC1
SPG21	RABAC1
RABAC1	SNX15
RABAC1	RAP1A
RAB7A	RABAC1
RABAC1	RAB5C
RABAC1	RAB5A
RAB4A	RABAC1
RAB3A	RABAC1
RABAC1	RAB22A
RAB1A	RABAC1
PLEKHF2	RABAC1
NUDT18	RABAC1
NIF3L1	RABAC1
LNX1	RABAC1
FXR2	RABAC1
RABAC1	FAM160A2
RABAC1	DUSP12
RABAC1	ADCK3
UBC	RAB8B
UBC	RAB8A
RPH3AL	RAB8A
RAB8A	RAB3IP
OPTN	RAB8A
RAB8A	MAP4K2
BAG6	RAB8A
UBC	RAB6B
SMAD1	RAB6B
UBC	RAB6A

DCTN1	RAB6A
RAB6A	BICD1
RAB5C	ZFYVE20
UBC	RAB5C
RAB5C	RIN3
UBC	RAB5B
EEA1	RAB5B
RAB5A	ZFYVE20
VPS45	RAB5A
UBC	RAB5A
STX4	RAB5A
RIN1	RAB5A
RABEP1	RAB5A
RAB7A	RAB5A
PIK3C3	RAB5A
EEA1	RAB5A
RAB5A	CHML
RAB5A	AGTR1
DYNLL1	RAB4A
RAB3IP	RAB3IL1
RAB11A	RAB3IL1
NUDT18	RAB3IL1
UBC	RAB3GAP2
UBC	RAB3GAP1
RPH3AL	RAB3D
RIMS2	RAB3C
UBC	RAB3B
SMAD1	RAB3B
RPH3AL	RAB3B
RPH3AL	RAB3A
RIMS1	RAB3A
RABIF	RAB3A
RAB3A	RABGGTB
UBC	RAB39A
TGFBR1	RAB38
SMAD4	RAB38
SMAD2	RAB38
SMAD1	RAB38
UBC	RAB37
UBC	RAB35
TBPL1	RAB35
PTPRS	RAB35
IDH3B	RAB35
GNPAT	RAB35

CBR3	RAB35
C2orf44	RAB35
UBC	RAB32
SMAD1	RAB30
UBC	RAB2B
SMAD4	RAB2B
SMAD1	RAB2B
UBC	RAB2A
GDI2	RAB2A
RPH3AL	RAB27B
ZBTB16	RAB27A
UBC	RAB27A
SYTL3	RAB27A
SYTL2	RAB27A
SYTL1	RAB27A
RPH3AL	RAB27A
RBM48	RAB27A
MYO5A	RAB27A
MLPH	RAB27A
EXPH5	RAB27A
COPS6	RAB27A
C14orf1	RAB27A
TGFBR1	RAB25
SMAD4	RAB25
MYO5B	RAB25
MED31	RAB25
ACVR1	RAB25
UBC	RAB24
UBC	RAB23
UBC	RAB22A
EEA1	RAB22A
UBC	RAB21
UBC	RAB1A
RAB1A	GDI2
UBC	RAB18
TOM1L1	RAB18
PDE6D	RAB18
ACVR1	RAB17
UBC	RAB14
UBC	RAB13
TGFBR1	RAB13
SMAD4	RAB13
PDE6D	RAB13
UBC	RAB11FIP5

RAB25	RAB11FIP5
RAB11A	RAB11FIP5
NAPG	RAB11FIP5
RAB11A	RAB11FIP4
RAB11A	RAB11FIP3
ARF5	RAB11FIP3
ARF4	RAB11FIP3
RAB11FIP2	RAB25
RAB11FIP2	RAB11A
RAB11FIP2	MYO5B
UBC	RAB11B
RAB7A	RAB11B
OPTN	RAB11B
OLFM2	RAB11B
RAB11A	RAB3IP
RAB11A	RAB11FIP1
UBC	RAB10
UBC	QTRTD1
QTRT1	SH3GL3
COPS6	QTRT1
UBC	QSER1
SUMO2	QSER1
UBC	QKI
RBFOX2	QKI
PTBP2	QKI
PTBP1	QKI
HNRNPK	QKI
TGFB2	PZP
MMP9	PZP
UBC	PYGM
MAP3K3	PYGM
UBC	PYGL
UBC	PYGB
PYCARD	PYDC2
UBC	PYCRL
UBC	PYCR2
SKIL	PYCR2
PYCARD	PRKAR1A
PYCARD	NLRP2
PXN	YBX1
PXN	VIM
PXN	VCL
UBC	PXN
PXN	TUBB

PXN	TMPO
PXN	TLN1
PXN	SRSF3
PXN	SLC3A2
PXN	SFPQ
PXN	SERPINH1
PXN	RPS6
PXN	RPL8
PXN	RPL17-C18orf32
PXN	RPL14
PXN	PTPRH
PXN	PTEN
PXN	PCOLCE
PXN	MYH9
PXN	MICAL1
PXN	LCK
PXN	ITGB1
PXN	ILK
PXN	HSPA8
PXN	FYN
PXN	FN1
PXN	DNAH3
PXN	COL4A1
PXN	COL12A1
PXN	CCDC80
PXN	ARHGEF7
PXN	ACTG1
PEX19	PXMP2
UBC	PWP2
TOM1L1	PWP2
UBC	PWP1
NOP56	PWP1
PVRL1	PVRL4
UBC	PVRL3
PVRL1	PVRL3
UBC	PVRL2
PVRL3	PVRL2
MLLT4	PVRL2
MDFI	PVRL2
VTN	PVR
UBC	PVR
PVRL3	PVR
UBC	PUS7L
YBX1	PURA

UBC	PURA
SP1 PURA
PURB	PURA
CCNT1	PURA
ADAP1	PURA
UBC	PUM2
MED23	PUM2
DAZL	PUM2
UBC	PUM1
SMAD1	PUM1
VIM	PUF60
UBC	PUF60
PUF60	U2AF2
PUF60	SF3A2
PUF60	SAP30BP
HNRNPC	PUF60
PUF60	CD101
UBC	PTTG1IP
PTTG1	PTTG1IP
PTTG1	FZR1
PTTG1	CDC16
ANAPC5	PTTG1
PTTG1	ANAPC4
UBC	PTS
PRKG2	PTS
ZNF148	PTRF
UBC	PTRF
TTF1	PTRF
EGFR	PTRF
UBC	PTPRU
CTNNB1	PTPRU
UNC119	PTPRS
UBR1	PTPRS
UBC	PTPRS
SETDB1	PTPRS
PTNPTPRS
PPFIA1	PTPRS
PFN2	PTPRS
MOB4	PTPRS
KAT5	PTPRS
FEZ1	PTPRS
EEF1G	PTPRS
CHD3	PTPRS
MAPK7	PTPRR

MAPK3	PTPRR
MAPK1	PTPRR
SNTB2	PTPRN
NOS1	PTPRN
GNB2L1	PTPRM
CTNND1	PTPRM
TK1	PTPRK
PSMD11	PTPRK
JUP	PTPRK
GSTK1	PTPRK
CTNNB1	PTPRK
UBC	PTPRJ
SDCBP	PTPRJ
PDGFRB	PTPRJ
NOP2	PTPRJ
MET	PTPRJ
MAPK3	PTPRJ
GAB1	PTPRJ
FLT1	PTPRJ
EGFR	PTPRJ
CTNND1	PTPRJ
CTNNB1	PTPRJ
UBC	PTPRG
DLG4	PTPRG
CTNNB1	PTPRG
UBC	PTPRF
SKIL	PTPRF
PTPRE	KCNA5
VAV1	PTPRC
UBC	PTPRC
PTPRC	PTPRCAP
PTPN6	PTPRC
PTPRC	PRKCSH
PPFIBP2	PTPRC
LCK	PTPRC
PTPRC	DPP4
PTPRC	CD2
PTN	PTPRB
PTPRB	MAPK3
GHR	PTPRB
CTNNB1	PTPRB
PTPRB	CNTN1
UBC	PTPRA
PTPRA	KCNA2

GRB2	PTPRA
FYN	PTPRA
BCAR1	PTPRA
UBC	PTPN6
PTPN6	TYK2
PTPN6	TNFRSF1A
PTPN6	SSTR2
PTPN6	ROS1
PTPN6	PRKCD
PTPN6	LCP2
PTPN6	LCK
PTPN6	KLRC1
PTPN6	KIT
PTPN6	KIR2DL3
PTPN6	JAK1
PTPN6	IL6ST
PTPN6	IL4R
PTPN6	IFNAR1
PTPN6	GHR
PTPN6	GAB2
PTPN6	FCGR2B
EGFR	PTPN6
PTPN6	CUZD1
PTPN6	CEACAM1
PTPN6	CD33
PTPN6	CD300LF
PTPN6	BTLA
PTPN6	BLNK
PTPN6	ACTN4
ZZEF1	PTPN4
KAT5	PTPN4
EEF1G	PTPN4
ZNF512B	PTPN3
ZAP70	PTPN3
YWHAG	PTPN3
YWHAB	PTPN3
VCP	PTPN3
PRKD2	PTPN3
UBC	PTPN23
GRAP2	PTPN23
DDX24	PTPN23
CHMP4B	PTPN23
LCK	PTPN22
TEC	PTPN21

KIF1C	PTPN21
STAT5B	PTPN2
STAT5A	PTPN2
STAT3	PTPN2
STAT1	PTPN2
PDGFRB	PTPN2
JAK3	PTPN2
JAK1	PTPN2
EGFR	PTPN2
TEC	PTPN18
PSTPIP1	PTPN18
PTPN18	ARHGAP5
ABL1	PTPN18
YWHAE	PTPN14
UBC	PTPN14
TJP1	PTPN14
MYC	PTPN14
JUP	PTPN14
UBC	PTPN13
PTPN13	RAPGEF6
PTPN13	PKN2
PDLIM4	PTPN13
NFKBIA	PTPN13
ARHGAP29	PTPN13
PTPN12	THAP2
PTPN12	TGFB1I1
PTPN12	TEX10
SMAD5	PTPN12
PTPN12	RBM19
PXN	PTPN12
PTPN12	IFT20
EGFR	PTPN12
PTPN12	CSH1
BAG6	PTPN12
UBC	PTPN11
TNFRSF1A	PTPN11
PTPN11	TIE1
PTPN11	TEK
STAT5A	PTPN11
STAT3	PTPN11
PTPN11	SOS1
PTPN11	SLAMF1
SIT1	PTPN11
PTPN11	SELE

PXN	PTPN11
PTK2B	PTPN11
PIK3R1	PTPN11
PDGFRB	PTPN11
PTPN11	PDGFRA
PTPN11	PAG1
PTPN11	MVP
MPZL1	PTPN11
MET	PTPN11
PTPN11	LIFR
PTPN11	LEPR
PTPN11	LAIR1
PTPN11	KLRC1
KIT	PTPN11
JAK2	PTPN11
JAK1	PTPN11
IRS1	PTPN11
PTPN11	INPP5D
IL6ST	PTPN11
PTPN11	IL4R
GRB2	PTPN11
GHR	PTPN11
GAB2	PTPN11
GAB1	PTPN11
FYN	PTPN11
PTPN11	FRS3
ERBB3	PTPN11
EGFR	PTPN11
CSF3R	PTPN11
PTPN11	CSF2RB
CRKL	PTPN11
PTPN11	CEACAM1
CDH5	PTPN11
CD33	PTPN11
CBL	PTPN11
CAV1	PTPN11
PTPN11	CAT
PTPN11	C6orf25
BTLA	PTPN11
PTPN11	BDKRB2
BCR	PTPN11
ARHGAP1	PTPN11
UBC	PTPN1
PTPN1	TRPV6

STAT3 PTPN1
RRAS2 PTPN1
PIN1 PTPN1
PTPN1 PDGFRB
PTPN1 MET
JAK2 PTPN1
IRS1 PTPN1
GRB2 PTPN1
EGFR PTPN1
CTNNB1 PTPN1
CSNK2A2 PTPN1
PTPN1 CLK2
CLK1 PTPN1
PTPN1 CDH2
CAV1 PTPN1
BCAR1 PTPN1
UBC PTPLAD1
TNFRSF1B PTPLAD1
UBC PTP4A2
UBC PTOV1
PIN1 PTOV1
PTNWFDC2
UTP3 PTN
UBQLN4 PTN
PTNTXNDC9
TP53BP2 PTN
PTNTBRG4
PTNTAF1D
PTNSUPT7L
PTNSTC2
SSR1 PTN
PTNSPIN1
SNCAIP PTN
PTNSLC25A36
PTNSGTA
PTNSDC1
RPLP1 PTN
PTNPTPRZ1
PSMD11 PTN
PTNP4HB
PTNNCAN
PTNMYCBP
PTNMKKS
PTNMAD2L1BP

LAMA4 PTN
ITSN2 PTN
PTNGTF2F1
PTNGSTP1
PTNGPD1
PTNGNB2L1
GIPC2 PTN
EIF3F PTN
PTNDUSP12
DNAJB11 PTN
PTNDFNA5
PTNCSTA
PTNCRCT1
CHD3 PTN
CDK5RAP2 PTN
PTNCDH10
PTNCCDC53
PTNCACNB4
CAAP1 PTN
BTBD2 PTN
PTNBLOC1S1
PTNBCCIP
PTNATP5C1
PTNAPOBEC3G
PTNALK
ACTB PTN
UBC PTMS
UBC PTMA
SET PTMA
HIST4H4 PTMA
CASP3 PTMA
UBC PTK7
EGFR PTK6
ZAP70 PTK2B
VAV1 PTK2B
UBC PTK2B
TLN1 PTK2B
TGFB1I1 PTK2B
SNCA PTK2B
PXN PTK2B
PTPN6 PTK2B
PTPN12 PTK2B
PRKCD PTK2B
PTK2B PITPNM1

PIK3R1	PTK2B
NPHP1	PTK2B
MCAM	PTK2B
LPXN	PTK2B
LCK	PTK2B
JAK3	PTK2B
JAK1	PTK2B
ITGB3	PTK2B
ITGB2	PTK2B
FYN	PTK2B
EGFR	PTK2B
CRK	PTK2B
PTK2B	CCR5
UBC	PTK2
TP53	PTK2
TLN1	PTK2
PTK2	TGFB1I1
PTK2	SYK
STAT1	PTK2
SRCPTK2	
PTK2	SOCS3
PTK2	SOCS2
PXN	PTK2
PTPN12	PTK2
PTK2	PTPN11
PTEN	PTK2
PTK2	PPP1CB
PLCG1	PTK2
PIK3R1	PTK2
PTK2	PDGFRB
PTK2	NEDD9
PTK2	NCK2
NCK1	PTK2
MICAL1	PTK2
PTK2	LYN
LPXN	PTK2
JAK2	PTK2
ITGB5	PTK2
PTK2	ITGB3
ITGB2	PTK2
ITGB1	PTK2
GRB2	PTK2
FYN	PTK2
FLT1	PTK2

FGR	PTK2
EPHB2	PTK2
EGFR	PTK2
CRK	PTK2
PTK2	CIB1
CD4PTK2
PTK2	CCR5
CBL	PTK2
PTK2	BCAR1
PTK2	ATG12
ARRB1	PTHLH
PTHPTH2R
YWHAH	PTH1R
SLC9A3R1	PTH1R
PTHLH	PTH1R
PTHPTH1R
ARRB1	PTH1R
PTHMEP1A
UBC	PTGS2
TP53	PTGS2
NUCB1	PTGS2
CAV1	PTGS2
UBC	PTGR1
GNAQ	PTGIR
CD151	PTGFRN
UBC	PTGES3L-AARSD1
UBC	PTGES3
TERT	PTGES3
NR3C1	PTGES3
HSP90AB1	PTGES3
HSP90AA1	PTGES3
ESR1	PTGES3
ARNT	PTGES3
UBC	PTER
UBC	PTDSS1
UBC	PTCD3
UBC	PTBP2
SNRPA	PTBP1
SFPQ	PTBP1
HNRNPA1	PTBP1
PSTPIP1 PTPN12
PSTPIP1 DNM2
PSTPIP1 CD2
RETPSPN

UBC	PSPH
UBC	PSPC1
TCERG1	PSPC1
SFPQ	PSPC1
PSMA2	PSMG3
UBC	PSMG2
POMP	PSMG2
UBC	PSMG1
PSMG2	PSMG1
UBC	PSMF1
UBC	PSME4
PSMA2	PSME4
UBC	PSME2
PSMA2	PSME2
VIM	PSME1
VCL	PSME1
UBC	PSME1
PSME1	PSME2
PSME1	PSMA6
UBC	PSMD9
PSMC3	PSMD9
UCHL5	PSMD8
UBC	PSMD8
TRAF6	PSMD8
SUMO2	PSMD8
SMAD9	PSMD8
SMAD2	PSMD8
SHFM1	PSMD8
PSMD7	PSMD8
PSMD6	PSMD8
PSMD4	PSMD8
PSMD3	PSMD8
PSMD2	PSMD8
PSMD14	PSMD8
PSMD13	PSMD8
PSMD11	PSMD8
PSMD1	PSMD8
PSMC6	PSMD8
PSMC5	PSMD8
PSMC4	PSMD8
PSMC3	PSMD8
PSMC2	PSMD8
PSMC1	PSMD8
PSMB6	PSMD8

PSMB4	PSMD8
PSMB3	PSMD8
PSMB1	PSMD8
PSMA7	PSMD8
PSMA6	PSMD8
PSMA4	PSMD8
PSMA2	PSMD8
PSMA1	PSMD8
PAAF1	PSMD8
UCHL5	PSMD7
UBC	PSMD7
PSMD7	PSMD4
PSMD7	PSMD3
PSMD11	PSMD7
PSMD7	PSMD1
PSMD7	PSMC3
PSMC2	PSMD7
PSMD7	PSMB4
PSMD7	PSMB3
PSMD7	PSMB1
PSMD7	PSMA6
PSMD7	PSMA2
UCHL5	PSMD6
UBC	PSMD6
TRAF6	PSMD6
SHFM1	PSMD6
PSMD7	PSMD6
PSMD4	PSMD6
PSMD3	PSMD6
PSMD14	PSMD6
PSMD13	PSMD6
PSMD11	PSMD6
PSMD1	PSMD6
PSMC6	PSMD6
PSMC3	PSMD6
PSMC2	PSMD6
PSMC1	PSMD6
PSMB4	PSMD6
PSMD6	PSMB3
PSMB1	PSMD6
PSMD6	PSMA7
PSMA6	PSMD6
PSMD6	PSMA4
PSMA2	PSMD6

PSMD6	PSMA1
PSMD6	PAAF1
UBC	PSMD5
PSMD2	PSMD5
PSMD1	PSMD5
PSMC2	PSMD5
PSMC1	PSMD5
RASSF8	PSMD4
PSMD4	PSMD3
PSMD11	PSMD4
PSMD4	PSMD1
PSMC3	PSMD4
PSMC2	PSMD4
PSMD4	PSMB4
PSMD4	PSMB1
PSMD4	PSMA6
PSMD4	PSMA5
PSMA2	PSMD4
NEDD4	PSMD4
MDM2	PSMD4
PSMD4	FBXO25
PSMD11	PSMD3
PSMD3	PSMD1
PSMC2	PSMD3
ZBTB16	PSMD2
UCHL5	PSMD2
PSMD2	UBXN7
UBC	PSMD2
TRAF6	PSMD2
TNFRSF1A	PSMD2
SUMO2	PSMD2
PSMD2	SHFM1
PSMD2	PTN
PSMD7	PSMD2
PSMD2	PSMD6
PSMD4	PSMD2
PSMD14	PSMD2
PSMD13	PSMD2
PSMD2	PSMD1
PSMC6	PSMD2
PSMC3	PSMD2
PSMC2	PSMD2
PSMD2	PSMC1
PSMA2	PSMD2

PSMD2	PSMA1
PSMD2	NSFL1C
NFKB2	PSMD2
MYC	PSMD2
MDM2	PSMD2
FKBP8	PSMD2
FAF2	PSMD2
UCHL5	PSMD14
UBC	PSMD14
PSMD7	PSMD14
PSMD4	PSMD14
PSMD14	PSMD3
PSMD14	PSMD13
PSMD11	PSMD14
PSMD14	PSMD1
PSMC3	PSMD14
PSMC2	PSMD14
PSMD14	PSMC1
PSMD14	PSMB4
PSMD14	PSMB3
PSMD14	PSMB1
PSMD14	PSMA7
PSMD14	PSMA6
PSMA2	PSMD14
PSMD14	PLEKHB2
PSMD14	PAAF1
UCHL5	PSMD13
UBC	PSMD13
PSMD7	PSMD13
PSMD4	PSMD13
PSMD13	PSMD3
PSMD11	PSMD13
PSMD13	PSMD1
PSMC3	PSMD13
PSMC2	PSMD13
PSMD13	PSMB4
PSMD13	PSMB3
PSMD13	PSMB1
PSMD13	PSMA6
PSMA2	PSMD13
NFKB2	PSMD13
UCHL5	PSMD12
UBC	PSMD12
TRAF6	PSMD12

PSMD12	SHFM1
PSMD7	PSMD12
PSMD12	PSMD6
PSMD4	PSMD12
PSMD12	PSMD3
PSMD14	PSMD12
PSMD13	PSMD12
PSMD11	PSMD12
PSMD12	PSMD1
PSMD12	PSMC6
PSMC5	PSMD12
PSMD12	PSMC4
PSMC2	PSMD12
PSMD12	PSMC1
PSMA2	PSMD12
PSMD12	PAAF1
SMAD5	PSMD11
SMAD2	PSMD11
PSMD11	PSMD1
PSMD11	PSMC2
HAP1	PSMD11
UCHL5	PSMC6
UBC	PSMC6
TRAF6	PSMC6
PSMC6	SHFM1
PSMC6	PSMD9
PSMD7	PSMC6
PSMD4	PSMC6
PSMC6	PSMD3
PSMD14	PSMC6
PSMD13	PSMC6
PSMD11	PSMC6
PSMC6	PSMD1
PSMC3	PSMC6
PSMC2	PSMC6
PSMC6	PSMC1
PSMC6	PAAF1
CRK	PSMC6
CCDC85B	PSMC6
PSMC6	C1orf216
UCHL5	PSMC5
UBC	PSMC5
TRAF6	PSMC5
TP53	PSMC5

PSMC5	THRB
SP1	PSMC5
PSMC5	SHFM1
RXRA	PSMC5
PSMC5	RORA
PSMC5	RARA
PSMC5	RAD23B
PSMC5	RAD23A
PSMC5	PSME1
PSMD7	PSMC5
PSMC5	PSMD6
PSMC5	PSMD4
PSMC5	PSMD3
PSMC5	PSMD2
PSMC5	PSMD14
PSMC5	PSMD13
PSMD11	PSMC5
PSMC5	PSMD1
PSMC5	PSMC6
PSMC3	PSMC5
PSMC2	PSMC5
PSMC5	PSMC1
PSMC5	PSMB4
PSMC5	PSMB3
PSMC5	PSMB1
PSMC5	PSMA7
PSMC5	PSMA6
PSMC5	PSMA4
PSMC5	PSMA2
PSMC5	PSMA1
PSMC5	PARK2
PSMC5	PAAF1
KRT15	PSMC5
FOS	PSMC5
ESR1	PSMC5
PSMC5	ERCC3
CIITA	PSMC5
PSMC5	ADRM1
UCHL5	PSMC4
UBC	PSMC4
PSMD7	PSMC4
PSMC4	PSMD6
PSMD4	PSMC4
PSMC4	PSMD3

PSMC4	PSMD2
PSMD14	PSMC4
PSMD13	PSMC4
PSMD11	PSMC4
PSMC4	PSMD1
PSMC6	PSMC4
PSMC5	PSMC4
PSMC3	PSMC4
PSMC2	PSMC4
PSMC4	PSMC1
PSMA2	PSMC4
PSMC4	PAAF1
NPM1	PSMC4
PSMC3	STIP1
PSMC3	PSMD3
PSMD11	PSMC3
PSMC3	PSMD1
PSMC2	PSMC3
MYC	PSMC3
PSMC2	PSMD1
VCP	PSMC1
UCHL5	PSMC1
UBC	PSMC1
TRAF6	PSMC1
PSMD7	PSMC1
PSMD4	PSMC1
PSMD3	PSMC1
PSMD13	PSMC1
PSMD11	PSMC1
PSMD1	PSMC1
PSMC3	PSMC1
PSMC2	PSMC1
PSMC1	PSMB3
PSMB1	PSMC1
PSMC1	PSMA7
PSMA6	PSMC1
PSMA2	PSMC1
PSMC1	PAAF1
FKBP8	PSMC1
CCDC85B	PSMC1
UBC	PSMB9
PSMA7	PSMB9
PSMA2	PSMB9
UBC	PSMB8

PSMB9	PSMB8
PSMB7	PSMB8
PSMB4	PSMB8
PSMB3	PSMB8
PSMB2	PSMB8
PSMB1	PSMB8
PSMA6	PSMB8
PSMA5	PSMB8
PSMA4	PSMB8
PSMA2	PSMB8
PSMA1	PSMB8
POMP	PSMB8
UBC	PSMB7
RAD23B	PSMB7
PSMD6	PSMB7
PSMD4	PSMB7
PSMD1	PSMB7
PSMB9	PSMB7
PSMB4	PSMB7
PSMB3	PSMB7
PSMB7	PSMB2
PSMB1	PSMB7
PSMB7	PSMA8
PSMA7	PSMB7
PSMA6	PSMB7
PSMA5	PSMB7
PSMA4	PSMB7
PSMA2	PSMB7
PSMA1	PSMB7
POMP	PSMB7
UCHL5	PSMB6
UBC	PSMB6
TRAF6	PSMB6
PSMD7	PSMB6
PSMD6	PSMB6
PSMD4	PSMB6
PSMD14	PSMB6
PSMD13	PSMB6
PSMC5	PSMB6
PSMC3	PSMB6
PSMC2	PSMB6
PSMC1	PSMB6
PSMB6	PSMB8
PSMB6	PSMB7

PSMB4	PSMB6
PSMB6	PSMB3
PSMB6	PSMB2
PSMB6	PSMB10
PSMB1	PSMB6
PSMB6	PSMA8
PSMB6	PSMA7
PSMA6	PSMB6
PSMA5	PSMB6
PSMB6	PSMA4
PSMA2	PSMB6
PSMB6	PSMA1
POMP	PSMB6
EZH2	PSMB6
UCHL5	PSMB5
UBC	PSMB5
TRAF6	PSMB5
PSMD6	PSMB5
PSMC5	PSMB5
PSMC4	PSMB5
PSMC3	PSMB5
PSMC2	PSMB5
PSMC1	PSMB5
PSMB9	PSMB5
PSMB8	PSMB5
PSMB7	PSMB5
PSMB6	PSMB5
PSMB4	PSMB5
PSMB3	PSMB5
PSMB2	PSMB5
PSMB1	PSMB5
PSMA7	PSMB5
PSMA6	PSMB5
PSMA5	PSMB5
PSMA4	PSMB5
PSMA2	PSMB5
PSMA1	PSMB5
POMP	PSMB5
LSM1	PSMB5
SMAD1	PSMB4
PSMD11	PSMB4
PSMC2	PSMB4
PSMB4	PSMB1
PSMB4	PSMA5

PSMA2	PSMB4
UCHL5	PSMB3
UBC	PSMB3
PSMD4	PSMB3
PSMD11	PSMB3
PSMC2	PSMB3
PSMB4	PSMB3
PSMB1	PSMB3
PSMA6	PSMB3
PSMA5	PSMB3
PSMA2	PSMB3
UBC	PSMB2
TRAF6	PSMB2
PSMB4	PSMB2
PSMB3	PSMB2
PSMB1	PSMB2
PSMA7	PSMB2
PSMA6	PSMB2
PSMA5	PSMB2
PSMA2	PSMB2
POMP	PSMB2
UBC	PSMB10
PTN	PSMB10
PSMB4	PSMB10
PSMB3	PSMB10
PSMA2	PSMB10
PSMA1	PSMB10
VCL	PSMB1
PSMD11	PSMB1
PSMC3	PSMB1
PSMC2	PSMB1
PSMB1	PSMA5
PSMA2	PSMB1
DCP2	PSMB1
UBC	PSMA8
UBC	PSMA7
PSMD7	PSMA7
PSMD4	PSMA7
PSMD13	PSMA7
PSMD11	PSMA7
PSMD1	PSMA7
PSMC3	PSMA7
PSMC2	PSMA7
PSMB4	PSMA7

PSMB3	PSMA7
PSMB1	PSMA7
PSMA6	PSMA7
PSMA5	PSMA7
PSMA2	PSMA7
PLK1	PSMA7
MDM2	PSMA7
PSMD11	PSMA6
PSMD1	PSMA6
PSMC3	PSMA6
PSMC2	PSMA6
PSMA6	PSMB4
PSMA6	PSMB1
PSMA6	PSMA5
PSMA2	PSMA6
UBC	PSMA4
TRAF6	PSMA4
PSMD7	PSMA4
PSMD4	PSMA4
PSMD14	PSMA4
PSMD13	PSMA4
PSMD11	PSMA4
PSMC2	PSMA4
PSMC1	PSMA4
PSMB4	PSMA4
PSMB3	PSMA4
PSMA4	PSMB2
PSMB1	PSMA4
PSMA7	PSMA4
PSMA6	PSMA4
PSMA5	PSMA4
PSMA2	PSMA4
PSMA4	PSMA1
POMP	PSMA4
PARK2	PSMA4
CUL1	PSMA4
UBC	PSMA3
STX11	PSMA3
STUB1	PSMA3
SNRPF	PSMA3
PSMD6	PSMA3
PSMD13	PSMA3
PSMD1	PSMA3
PSMC4	PSMA3

PSMC3	PSMA3
PSMC1	PSMA3
PSMB8	PSMA3
PSMB7	PSMA3
PSMB6	PSMA3
PSMB5	PSMA3
PSMB4	PSMA3
PSMB3	PSMA3
PSMB2	PSMA3
PSMB1	PSMA3
PSMA7	PSMA3
PSMA6	PSMA3
PSMA5	PSMA3
PSMA4	PSMA3
PSMA2	PSMA3
PSMA1	PSMA3
POMP	PSMA3
CSNK2A1	PSMA3
AURKB	PSMA3
PSMA2	PSMD3
PSMD11	PSMA2
PSMA2	PSMD1
PSMC3	PSMA2
PSMC2	PSMA2
PSMA2	PSMA5
VIM	PSMA1
UBQLN1	PSMA1
UBC	PSMA1
TSC22D4	PSMA1
TRAF6	PSMA1
RAD54B	PSMA1
PSME1	PSMA1
PSMD7	PSMA1
PSMD4	PSMA1
PSMD14	PSMA1
PSMD13	PSMA1
PSMD11	PSMA1
PSMC3	PSMA1
PSMC2	PSMA1
PSMC1	PSMA1
PSMA1	PSMB9
PSMB4	PSMA1
PSMB3	PSMA1
PSMA1	PSMB2

PSMB1	PSMA1
PSMA7	PSMA1
PSMA6	PSMA1
PSMA5	PSMA1
PSMA2	PSMA1
NMI	PSMA1
NECAB2	PSMA1
MTUS2	PSMA1
MCM6	PSMA1
PSMA1	LDB1
KRT15	PSMA1
CCNH	PSMA1
CCDC85B	PSMA1
CALCOCO2	PSMA1
UBC	PSIP1
MEN1	PSIP1
SMAD2	PSG9
PTPN12	PSG9
UBQLN1	PSEN2
UBC	PSEN2
TRAF6	PSEN2
SRI	PSEN2
PSENEN	PSEN2
PSEN1	PSEN2
NCSTN	PSEN2
KCNIP3	PSEN2
CTNND2	PSEN2
CSNK1D	PSEN2
CFLAR	PSEN2
CASP8	PSEN2
CASP7	PSEN2
CASP6	PSEN2
CASP3	PSEN2
CASP1	PSEN2
CAPN1	PSEN2
BCL2L1	PSEN2
APP	PSEN2
APH1B	PSEN2
APH1A	PSEN2
UBC	PSEN1
PSEN1	PSENEN
MAPT	PSEN1
PSEN1	BACE1
PSEN1	APP

UBC	PSD3
UBC	PSAT1
SMAD2	PSAP
PSAP	GBA
RCN3	PRUNE2
EWSR1	PRUNE2
GSK3B	PRUNE
TNF	PRTN3
PRTN3	SERPINB13
ITGB2	PRTN3
PRTN3	IL1B
PRTN3	ELN
UBC	PRTFDC1
DTNB	PRTFDC1
PRSS23	SHMT1
RACGAP1	PRSS23
PPIA	PRSS23
PRSS23	PHF23
PRSS23	PDDC1
NOC4L	PRSS23
PRSS23	GRK6
PRSS23	FXR1
PRSS23	EFNA3
PRSS23	B2M
UBC	PRSS21
UBC	PRSS1
PRSS1	SPINK5
PRSS1	SERPINF2
APP	PRSS1
SNAP25	PRRT2
VCL	PRRG2
PXN	PRRG2
UBC	PRRC2B
EHMT2	PRRC2B
UBC	PRRC2A
GRB2	PRRC2A
PIN1	PRRC1
UBC	PRR5-ARHGAP8
UBC	PRR14L
PPIA	PRR13
KLHL12	PRR13
UBC	PRR12
UBC	PRR11
UBC	PRPSAP2

ENDOV	PRPSAP2
UBC	PRPSAP1
PLEKHF2	PRPSAP1
DDX19B	PRPSAP1
UBC	PRPS1L1
ROM1	PRPH2
UBC	PRPH
PRPH2	PRPH
KRT15	PRPH
UBC	PRPF8
SNRNP40	PRPF8
SF3B3	PRPF8
SART1	PRPF8
PIN1	PRPF8
NFKB2	PRPF8
NEDD4	PRPF8
EFTUD2	PRPF8
DHX9	PRPF8
PRPF8	CD2BP2
UBC	PRPF6
TXNL4A	PRPF6
SF3B3	PRPF6
SART1	PRPF6
PRPF8	PRPF6
MYC	PRPF6
PRPF6	CD2BP2
YWHAG	PRPF4B
UBC	PRPF4B
POLR3F	PRPF4B
HSPB3	PRPF4B
PRPF40A	ZNF451
PRPF40A	ZCCHC8
YWHAG	PRPF40A
PRPF40A	YBX1
PRPF40A	WASF2
PRPF40A	VCL
VASP	PRPF40A
PRPF40A	UBQLN4
PRPF40A	UBQLN1
UBE2I	PRPF40A
UBC	PRPF40A
PRPF40A	U2AF2
PRPF40A	STIM2
SNRNP70	PRPF40A

SMAD4	PRPF40A
PRPF40A	SFPQ
PRPF40A	SF3B4
SF3B3	PRPF40A
PRPF40A	SF3B1
PRPF40A	SF3A3
PRPF40A	SF3A2
PRPF40A	SF1
SERPINB9	PRPF40A
PRPF40A	RPL4
PRPF40A	RLF
PTPRF	PRPF40A
PRPF40A	PSG5
PRPF40A	PRKAR1A
PRPF40A	PIAS3
PRPF40A	PIAS1
PRPF40A	PFKL
PRPF40A	NCKAP1L
PRPF40A	IL1RAP
PRPF40A	HTT
PRPF40A	HNRNPUL1
PRPF40A	HNRNPF
PRPF40A	HIPK1
PRPF40A	FMNL2
PRPF40A	FMNL1
PRPF40A	DHX9
PRPF40A	DDX46
PRPF40A	DAAM1
PRPF40A	CPSF6
PRPF40A	CDK13
PRPF40A	CDK12
PRPF40A	CCT7
PRPF40A	CCT4
PRPF40A	CAP2
PRPF40A	BRD4
BAG6	PRPF40A
PRPF40A	ASXL2
PRPF40A	APBB1IP
PRPF40A	ABI1
UBC	PRPF4
SART1	PRPF4
PRPF31	PRPF4
UBC	PRPF31
SF3B3	PRPF31

PRPF31	SF3A3
PRPF31	PRPF6
WBP4	PRPF3
USP4	PRPF3
UBC	PRPF3
U2AF2	PRPF3
SUMO2	PRPF3
SF3A2	PRPF3
SART1	PRPF3
PRPF8	PRPF3
PRPF6	PRPF3
PRPF4	PRPF3
GTPBP3	PRPF3
DVL2	PRPF3
CCDC85B	PRPF3
YWHAG	PRPF19
USB1	PRPF19
UBC	PRPF19
U2AF1	PRPF19
SF3A3	PRPF19
RBM5	PRPF19
PSMB4	PRPF19
PRPF8	PRPF19
PRPF3	PRPF19
PLRG1	PRPF19
EXOC7	PRPF19
CDC5L	PRPF19
BCAS2	PRPF19
HNF4A	PROX1
EP300	PROX1
TYRO3	PROS1
THBD	PROC
SERPINA5	PROC
PROS1	PROC
PROCR	PROC
PF4	PROC
F5	PROC
F2	PROC
CP	PROC
STIP1	PRNP
PLG	PRNP
HSPA5	PRNP
BAG6	PRNP
SYNCRIP	PRMT8

PRMT1 PRMT8
UBC PRMT7
PRMT5 OLA1
PRMT5 CLNS1A
ESR1 PRMT2
DMRTB1 PRMT2
CPSF7 PRMT2
WDR33 PRMT1
PRMT1 USF1
UBC PRMT1
PRMT1 STAT1
PRMT1 ILF3
PRMT1 IFNAR1
PRMT1 IDH3B
HNRNPA1 PRMT1
PRMT1 FUS
PRMT1 DHX9
VAV1 PRLR
UBC PRLR
TECPRLR
SOCS3 PRLR
SOCS2 PRLR
PTPN11 PRLR
PRLPRLR
PPIA PRLR
JAK2 PRLR
GH1 PRLR
PRLHR PRLH
PPIB PRL
UBC PRKRIR
PRKRIR EIF2S1
UBC PRKRA
PRKRA TARBP2
EIF2AK2 PRKRA
COPS6 PRKRA
GTF2I PRKG1
YWHAQ PRKDC
YWHAG PRKDC
PRKDC XRCC4
WRN PRKDC
UBC PRKDC
TP53 PRKDC
PRKDC TOP1
PRKDC TNFRSF1B

PRKDC	TNFRSF1A
PRKDC	TELO2
SUMO2	PRKDC
PRKDC	RPA1
PRKDC	RIPK3
PRKCD	PRKDC
PRKDC	PARP1
PRKDC	NFKB2
PRKDC	NFKB1
PRKDC	MAPK9
PRKDC	LYN
PRKDC	LIG4
PRKDC	ILF3
PRKDC	ILF2
PRKDC	HIST1H3A
PRKDC	H2AFX
PRKDC	GZMB
ESR1	PRKDC
PRKDC	DCLRE1C
PRKDC	CHD1L
PRKDC	C1D
PRKDC	ATM
PRKDC	AKT2
ABL1	PRKDC
UBC	PRKD3
GSK3A	PRKD3
UBC	PRKD1
PRKD1	PRKCE
PLCG2	PRKD1
EGFR	PRKD1
YWHAB	PRKCZ
WWC1	PRKCZ
VHL	PRKCZ
UBC	PRKCZ
SQSTM1	PRKCZ
SP1	PRKCZ
RELA	PRKCZ
RAF1	PRKCZ
PRKCA	PRKCZ
PRG2	PRKCZ
PARD6G	PRKCZ
PARD6B	PRKCZ
NCL	PRKCZ
MAP2K5	PRKCZ

KRT10	PRKCZ
IKBKB	PRKCZ
HSP90AA1	PRKCZ
FADD	PRKCZ
CDC42	PRKCZ
C1QBP	PRKCZ
AKT3	PRKCZ
ADCY5	PRKCZ
ADAP1	PRKCZ
UBC	PRKCSH
VAV1	PRKCQ
RASGRP3	PRKCQ
MAP3K7	PRKCQ
LYN	PRKCQ
LCK	PRKCQ
GLRX3	PRKCQ
FYN	PRKCQ
CHUK	PRKCQ
BCL10	PRKCQ
UBC	PRKCI
SQSTM1	PRKCI
SMG1	PRKCI
PARD6B	PRKCI
PARD3	PRKCI
MAP2K5	PRKCI
HSP90AA1	PRKCI
CHUK	PRKCI
CDC42	PRKCI
UBC	PRKCH
PRKD1	PRKCH
TIAM1	PRKCG
PRKCG	GSK3A
PRKCG	AFAP1
PRKCE	TRPV1
TIAM1	PRKCE
PRKCE	COPB1
PRKCE	CNN1
PRKCE	CFTR
UBC	PRKCDBP
SDPR	PRKCDBP
CAV1	PRKCDBP
PRKCD	STAT1
PRKCD	HIST1H1B
UBC	PRKCB

PRKCB	MARCKS
PRKCB	MAPK6
PRKCB	GSK3B
PRKCB	GSK3A
PRKCB	GNB2L1
PRKCB	GCNT1
PRKCB	ADRBK1
PRKCA	TEP1
PRKCA	RHO
PRKCA	HIST1H1B
PRKCA	GSK3A
PRKCA	GRM5
UBC	PRKAR2B
NBEA	PRKAR2B
AKAP9	PRKAR2B
AKAP11	PRKAR2B
AKAP10	PRKAR2B
PRKAR2A	WASF1
UBC	PRKAR2A
PRKAR2A	RAB32
PRKACB	PRKAR2A
NBEA	PRKAR2A
MAP2	PRKAR2A
PRKAR2A	KPNA6
EZRPRKAR2A	
CFTR	PRKAR2A
PRKAR2A	CBFA2T3
PRKAR2A	AKAP9
PRKAR2A	AKAP6
PRKAR2A	AKAP5
PRKAR2A	AKAP3
PRKAR2A	AKAP2
AKAP13	PRKAR2A
PRKAR2A	AKAP12
AKAP11	PRKAR2A
PRKAR2A	AKAP10
PRKAR2A	AKAP1
WRN	PRKAR1B
UBC	PRKAR1B
PRKAR1A	PRKAR1B
SMAD2	PRKAR1A
UBC	PRKAG2
PRKAG1	PRKAG2
PRKAB2	PRKAG2

PRKAB1	PRKAG2
PRKAA1	PRKAG2
UBC	PRKAG1
PRKAG1	PRKAB2
PRKAA1	PRKAG1
RPS6	PRKACB
VTN	PRKACA
VIM	PRKACA
VASP	PRKACA
UBC	PRKACA
TH	PRKACA
STMN2	PRKACA
STMN1	PRKACA
STK11	PRKACA
RYR1	PRKACA
RRAD	PRKACA
RELA	PRKACA
RAF1	PRKACA
PRKAR1A	PRKACA
PRKACB	PRKACA
PLNPRKACA
PFKFB2	PRKACA
PDE4D	PRKACA
PDE3B	PRKACA
PDC	PRKACA
NOS1	PRKACA
MAPT	PRKACA
LIPE	PRKACA
KCNH2	PRKACA
ITPKA	PRKACA
HSPD1	PRKACA
HNRNPD	PRKACA
HMGN1	PRKACA
HDAC1	PRKACA
GSK3A	PRKACA
GRIA4	PRKACA
GNMT	PRKACA
GFAP	PRKACA
FOSPRKACA
ETV1	PRKACA
EGFR	PRKACA
DNAJC5PRKACA
CFTR	PRKACA
CACNB2	PRKACA

BCL2	PRKACA
BAD	PRKACA
APC	PRKACA
ADD2	PRKACA
UBC	PRKAB2
UBC	PRKAB1
PRKAG1	PRKAB1
PRKAB2	PRKAB1
PRKAA1	PRKAB1
NDUFA7	PRKAB1
F10	PRKAB1
UBC	PRKAA2
PRKAA2	PRKAG1
PRKAA2	PRKAB2
PRKAA2	PRKAB1
PRKAA2	ACACA
UBC	PRKAA1
PRKAA1	PRKAB2
PRKAA1	GRIK2
PRKAA1	CDC37
UBC	PRIM2
PRIM1	PRIM2
POLA2	PRIM2
UBC	PRICKLE2
UBC	PRG2
RBM48	PRG2
PAPPA	PRG2
GZMB	PRF1
RPTOR	PREX1
UBC	PREPL
UBC	PREP
NID1	PRELP
HSPG2	PRELP
FN1	PRELP
FBLN2	PRELP
COL2A1	PRELP
COL1A1	PRELP
UBC	PRELID1
UBC	PREB
TGFBR1	PREB
SMAD1	PREB
UBC	PRDX6
CDK2	PRDX6
UBC	PRDX3

RPS6KC1	PRDX3
UBC	PRDX1
PRDX1	SESN2
RIPK3	PRDX1
PXN	PRDX1
PRDX2	PRDX1
IRF3	PRDX1
EGFR	PRDX1
CDK2	PRDX1
PRMT5	PRDM1
HDAC2	PRDM1
EHMT2	PRDM1
UBC	PRCP
USHBP1	PRC1
UBC	PRC1
TRIM37	PRC1
KRT20	PRC1
CCDC85B	PRC1
UBC	PRAMEF12
UBC	PRAME
TCEB2	PRAME
TCEB1	PRAME
STK19	PRAME
CUL2	PRAME
SKAP2	PRAM1
UBC	PQLC1
UBC	PPWD1
UBC	PPT1
UBC	PPP6R3
PPP6C	PPP6R3
UBC	PPP6R1
PPP6C	PPP6R1
UBC	PPP6C
PPP6C	PPP6R2
UBC	PPP5C
NR3C1	PPP5C
MAPT	PPP5C
MAP3K5	PPP5C
HSP90AB1	PPP5C
HSP90AA1	PPP5C
GNA13	PPP5C
GNA12	PPP5C
CSE1L	PPP5C
CRY2	PPP5C

CPNE4	PPP5C
CPNE2	PPP5C
CPNE1	PPP5C
CDC27	PPP5C
CDC16	PPP5C
PPP4C	PPP4R4
UBC	PPP4R2
SMEK1	PPP4R2
PPP4C	PPP4R2
UBC	PPP4C
TCP1	PPP4C
SMEK1	PPP4C
PPP4R1	PPP4C
CCT8	PPP4C
CCT7	PPP4C
PPP4C	CCT6A
CCT4	PPP4C
BAG6	PPP4C
UBC	PPP3R1
PPP3CA	PPP3R1
PPIA	PPP3R1
CABP2	PPP3R1
UBC	PPP3CC
UBC	PPP3CB
NFATC2	PPP3CB
CABIN1	PPP3CB
UBC	PPP3CA
PPIA	PPP3CA
FKBP8	PPP3CA
UBC	PPP2R5E
PPP2R1B	PPP2R5E
UBC	PPP2R5D
PPP2R5D	SGOL1
PPP4C	PPP2R5D
PPP2R1B	PPP2R5D
UBC	PPP2R5C
SGOL1	PPP2R5C
PPP2R1B	PPP2R5C
PPP2R5A	APC
UBC	PPP2R4
SET	PPP2R4
CCNG2	PPP2R4
CCNG1	PPP2R4
UBC	PPP2R3C

SMAD2 PPP2R2D
CCT7 PPP2R2C
UBC PPP2R2B
SMAD2 PPP2R2B
PPP2R1B PPP2R2B
STK24 PPP2R1B
PPP2R5A PPP2R1B
PPP2R1B PPP2R2C
PPP2R2A PPP2R1B
UBC PPP2R1A
STRN3 PPP2R1A
STRN PPP2R1A
STRIP1 PPP2R1A
STK25 PPP2R1A
STK24 PPP2R1A
SGOL1 PPP2R1A
PPP4C PPP2R1A
PPP2R5E PPP2R1A
PPP2R5D PPP2R1A
PPP2R5C PPP2R1A
PPP2R5A PPP2R1A
PPP2R2D PPP2R1A
PPP2R2C PPP2R1A
PPP2R2B PPP2R1A
PPP2R2A PPP2R1A
PPP2R1B PPP2R1A
PPP2CB PPP2R1A
PPP2CA PPP2R1A
PPME1 PPP2R1A
PPFIA1 PPP2R1A
MOB4 PPP2R1A
MAP3K3 PPP2R1A
HSP90AA1 PPP2R1A
CFTR PPP2R1A
UBC PPP2CB
PPP2CB TIPRL
TCP1 PPP2CB
STRN PPP2CB
PPP2CB STRIP1
STK24 PPP2CB
PPP2CB SGOL1
PPP2R5D PPP2CB
PPP2R2A PPP2CB
PPP2R1B PPP2CB

MOB4	PPP2CB
MAPT	PPP2CB
CCT8	PPP2CB
CCT7	PPP2CB
PPP2CB	CCT6A
CCT5	PPP2CB
CCT4	PPP2CB
PPP2CB	CCT3
PPP2CB	CCT2
AXIN1	PPP2CB
UBC	PPP2CA
TRIP13	PPP2CA
TP53	PPP2CA
PPP2CA	TIPRL
TCP1	PPP2CA
STRN	PPP2CA
PPP2CA	STRIP1
STK25	PPP2CA
STK24	PPP2CA
SPRY2	PPP2CA
RPS6KB1	PPP2CA
PPP2CA	RELA
RBL2	PPP2CA
RAF1	PPP2CA
PRKAA1	PPP2CA
PPP2CA	PPP2R5E
PPP2R5D	PPP2CA
PPP2CA	PPP2R5C
PPP2CA	PPP2R5B
PPP2R5A	PPP2CA
PPP2CA	PPP2R4
PPP2CA	PPP2R2D
PPP2R2C	PPP2CA
PPP2R2B	PPP2CA
PPP2R2A	PPP2CA
PPP2R1B	PPP2CA
PPP2CA	PPME1
PPFIA1	PPP2CA
MYC	PPP2CA
MOB4	PPP2CA
MAPT	PPP2CA
PPP2CA	IKBKB
HDAC4	PPP2CA
PPP2CA	EIF4EBP1

CHEK2 PPP2CA
CDK2 PPP2CA
CCT8 PPP2CA
CCT7 PPP2CA
PPP2CA CCT6A
CCT5 PPP2CA
CCT4 PPP2CA
PPP2CA CCT3
PPP2CA CCT2
CAV1 PPP2CA
BCL2 PPP2CA
PPP1CA PPP1R9B
PPP1R9B DRD2
UBC PPP1R9A
RPS6KB1 PPP1R9A
PPP1CA PPP1R9A
ACTG1 PPP1R9A
PPP1R8 STC2
RBM48 PPP1R8
PPP1CC PPP1R8
PPP1R8 PPP1CB
PPP1CA PPP1R8
HDAC2 PPP1R8
GBP2 PPP1R8
EED PPP1R8
C14orf1 PPP1R8
UBC PPP1R7
PPP1CA PPP1R3B
PLNPPP1R3A
PPP1CA PPP1R26
PPP1CA PPP1R2
ROBO2 PPP1R1B
PPP1CA PPP1R1B
UBC PPP1R18
PPP1CC PPP1R18
PPP1R16A RGMA
PUF60 PPP1R16A
PAPSS1 PPP1R16A
PPP1R16A LY6H
PPP1R16A IDO1
PPP1R16A FSD1
FAM101B PPP1R16A
ZNF512B PPP1R15A
SMARCB1 PPP1R15A

PPP1CA	PPP1R15A
KIF3A	PPP1R15A
HSPA1A	PPP1R15A
EIF2S1	PPP1R15A
CSNK1A1	PPP1R14A
UBC	PPP1R13L
TP53	PPP1R13L
TP73	PPP1R13B
TP63	PPP1R13B
PPP1CC	PPP1R13B
PPP1CA	PPP1R13B
LATS2	PPP1R13B
KHDRBS1	PPP1R13B
PPP1R12A	ROCK2
ROCK1	PPP1R12A
RIPK3	PPP1R12A
PPP1R12A	PPP1CB
ILK	PPP1R12A
UBC	PPP1R10
SUMO1	PPP1R10
PPP1CA	PPP1R10
UBC	PPP1CC
PPP1CC	TP53BP2
PPP1CC	SH3RF2
PPP1CC	PPP1R9B
PPP1CC	PPP1R7
PPP1CC	PPP1R2
PPP1CC	PPP1R15B
PPP1CC	PPP1R15A
PPP1CC	LMTK2
PPP1CC	HSPA8
HDAC1	PPP1CC
PPP1CC	EIF2S1
PPP1CC	CSRNP1
PPP1CC	COPS5
PPP1CC	CNST
PPP1CC	C1QA
PPP1CC	C15orf59
ZFYVE9	PPP1CB
UBC	PPP1CB
PPP1CB	PPP1R7
PPP1CB	PPP1R15A
PPP1CA	ZFYVE16
UBC	PPP1CA

PPP1CA	TUSC3
TP53	PPP1CA
PPP1CA	SYTL2
PPP1CA	SH3RF2
PPP1CA	RRP1B
PPP1R37	PPP1CA
PPP1CA	PPP1R15B
PPP1CA	PPP1R13L
PPP1CA	HSPA8
PPP1CA	GSK3B
PPP1CA	CD2BP2
PPP1CA	AKAP11
PPP1CA	ACTB
IMMT	PPOX
HAP1	PPOX
UBC	PPME1
UBC	PPM1G
UNG	PPM1D
UBC	PPM1D
UBC	PPM1A
SKIL	PPL
UBC	PPIL4
SMAD5	PPIL4
UBC	PPIL3
SLU7	PPIL3
UBC	PPIL2
UBC	PPIL1
SNW1	PPIL1
UBC	PPIH
PRPF4	PPIH
PRPF18	PPIH
UBC	PPIF
SLC25A4	PPIF
ATP6V1E1	PPIF
UBC	PPIE
PHYHIP	PPIE
UBC	PPID
SLC25A4	PPID
KAT7	PPID
HSP90AA1	PPID
HAP1	PPID
UBC	PPIB
TRAF1	PPIB
SGTB	PPIB

DNM2	PPIB
CAMLG	PPIB
APOB	PPIB
ITK	PPIA
UBC	PPHLN1
YWHAQ	PPFIBP1
YWHAG	PPFIBP1
UBC	PPFIBP1
PTPRU	PPFIA3
PPFIA2	PPFIA3
ERC2	PPFIA3
UBC	PPFIA2
PPFIA2	ERC2
UBC	PPFIA1
PTPRF	PPFIA1
PPFIA1	PPP2R5D
PPFIA1	ERC2
CALM1	PPEF2
UBC	PPDPF
UBC	PPCS
PMAIP1	PPBP
PPBP	EFNA1
PPBP	CXCR1
COX17	PPBP
COPS7A	PPBP
PPBP	CELF1
UBC	PPAT
UBC	PPARGC1A
PPARGC1A	THRB
RXRA	PPARGC1A
PPARGC1A	PPARG
PPARGC1A	PPARA
MED1	PPARGC1A
PPARGC1A	ESRRG
UBC	PPARG
RXRA	PPARG
NCOR2	PPARG
NCOA2	PPARG
MED24	PPARG
MED1	PPARG
NCOR2	PPARD
BCL6	PPARD
RXRA	PPARA
NCOR2	PPARA

MED24	PPARA
MED1	PPARA
UBC	PPAP2C
BAG6	PPAP2C
UBC	PPAP2B
FXYD1	PPAP2A
UBC	PPAN
NOP56	PPAN
CUL1	PPAN
UBC	PPA2
UBC	PPA1
TP53	PPA1
POU5F1	SOX2
ESR1	POU4F2
SOX11	POU3F3
SOX11	POU3F2
HMGA1	POU3F1
UBC	POU2F3
UBC	POU2F2
TBP	POU2F2
POU2AF1	POU2F2
NR3C1	POU2F2
XRCC6	POU2F1
UBC	POU2F1
POU2F1	TBP
POU2F1	STAT5B
SP1	POU2F1
POU2F1	SOX2
POU2F1	SNAPC4
RXRA	POU2F1
PRKDC	POU2F1
POU2F1	POU2AF1
PARP1	POU2F1
POU2F1	NPAT
NCOR2	POU2F1
POU2F1	LIFR
HOXC4	POU2F1
POU2F1	BRCA1
SUB1	POU2AF1
NR1I3	POU1F1
GATA2	POU1F1
ETS1	POU1F1
UBC	POR
POR	CYP2E1

POR	CYP2C9
POR	CYP2C19
UBC	POP7
RPP40	POP7
RPP25	POP7
HSPB1	POP7
UBC	POP5
RPP14	POP5
UBC	POP4
RPP38	POP4
RPP30	POP4
RPP25	POP4
POP5	POP4
UBC	POP1
RPP38	POP1
RPP30	POP1
UBC	PON2
UBC	POMZP3
UBC	POMT1
POMT2	POMT1
UBC	POMP
POMP	PSMB9
PSMB4	POMP
POMP	PSMB3
PSMB1	POMP
POMP	PSMA7
PSMA6	POMP
VTN	POMC
MC5R	POMC
MC4R	POMC
MC2R	POMC
MC1R	POMC
TFB2M	POLRMT
TFB1M	POLRMT
NFKB2	POLRMT
POLR3A	POLR3H
POLR2H	POLR3H
POLR3C	POLR3GL
POLR3F	WDR89
UBC	POLR3F
STC2	POLR3F
POLR3F	POLR3G
POLR3D	POLR3F
POLR3F	POLR3C

KAT5	POLR3F
C14orf1	POLR3F
POLR3D	POLR3H
POLR3D	POLR3G
POLR3A	POLR3D
POLR3D	POLR2H
UBC	POLR3C
POLR3C	POLR3H
POLR3C	POLR3G
GTF3C4	POLR3C
UBC	POLR3B
POLR3H	POLR3B
POLR3D	POLR3B
POLR3A	POLR3B
POLR2H	POLR3B
POLR1D	POLR3B
UBC	POLR2L
POLR2H	POLR2L
POLR2D	POLR2L
POLR1A	POLR2L
NFKBIB	POLR2L
MED26	POLR2L
CCDC85B	POLR2L
UBC	POLR2J
POLR2E	POLR2J
INTS3	POLR2J
POLR2H	POLR2I
POLR2D	POLR2I
POLR2C	POLR2I
POLR2B	POLR2I
MED26	POLR2I
UBC	POLR2H
POLR3A	POLR2H
POLR2E	POLR2H
POLR1A	POLR2H
UBC	POLR2G
POLR2G	POLR2J
POLR2G	POLR2H
POLR2G	POLR2F
POLR2E	POLR2G
POLR2G	MED26
GTF2F2	POLR2G
UBC	POLR2F
POLR2F	POLR2K

RUVBL2	POLR2E
UBC	POLR2D
POLR2J	POLR2D
POLR2H	POLR2D
POLR2G	POLR2D
POLR2D	MED26
UBE2W	POLR2C
UBC	POLR2C
POLR2C	STC2
POLR2C	POLR2L
POLR2C	POLR2J
POLR2C	POLR2H
POLR2C	POLR2G
POLR2C	POLR2F
POLR2E	POLR2C
POLR2C	POLR2D
POLR2C	POLR1D
POLR2C	MED26
GTF2F2	POLR2C
C14orf1	POLR2C
ATF4	POLR2C
UBC	POLR2B
POLR2B	RECQL5
POLR2B	POLR2J
POLR2B	POLR2H
POLR2B	POLR2G
POLR2B	POLR2E
POLR2B	POLR2D
POLR2B	POLR2C
POLR2B	MED28
POLR2B	MED26
POLR2B	GTF2F2
POLR2B	CDK7
POLR2A	ZNF74
POLR2A	WWOX
VHL	POLR2A
UPF1	POLR2A
UBC	POLR2A
POLR2A	TCERG1
POLR2A	TBP
POLR2A	TAF11
POLR2A	SUPT6H
POLR2A	SMARCE1
POLR2A	SMARCC1

POLR2ASMARCB1
POLR2ASMARCA4
POLR2ASMARCA2
POLR2ASAFB
POLR2ARECQL5
POLR2APOLR2J
POLR2APOLR2I
POLR2APOLR2H
POLR2APOLR2G
POLR2APOLR2F
POLR2APOLR2E
POLR2APOLR2D
POLR2APOLR2C
POLR2APOLR2B
PIN1 POLR2A
NEDD4 POLR2A
MYC POLR2A
POLR2AMEN1
POLR2AMED28
POLR2AMED26
POLR2AMED21
POLR2AMDC1
POLR2AMCM7
POLR2AGTF2H1
POLR2AGTF2F2
POLR2AGTF2F1
POLR2AGTF2E1
POLR2AGTF2B
POLR2AERCC6
POLR2AERCC5
POLR2AENTHD2
DHX9 POLR2A
POLR2ACUL5
POLR2ACTDP1
POLR2ACSTF3
POLR2ACSTF1
CREBBP POLR2A
POLR2ACPSF3
POLR2ACDK9
POLR2ACDK8
POLR2ACDK7
POLR2ABRCA1
POLR2AAGO1
POLR2AACTB

UBTF	POLR1E
UBC	POLR1E
POLR2E	POLR1E
POLR1B	POLR1E
POLR1A	POLR1E
NFKBIB	POLR1E
DDX6	POLR1E
UBC	POLR1D
POLR3H	POLR1D
POLR3D	POLR1D
POLR3A	POLR1D
POLR1A	POLR1D
NFKBIB	POLR1D
UBC	POLR1C
POLR1E	POLR1C
POLR1D	POLR1C
POLR1A	POLR1C
NME1	POLR1C
NFKBIB	POLR1C
POLR1A	POLR1B
POLR1A	POLR3A
POLR2E	POLR1A
RAD18	POLN
FANCD2	POLN
UBC	POLK
REV1	POLK
RAD18	POLK
PCNA	POLK
UBC	POLI
RNF11	POLI
REV1	POLI
POLH	POLI
PCNA	POLI
DVL2	POLI
DAZAP2	POLI
CALCOCO2	POLI
RAD18	POLH
DYNLL1	POLH
POLG	POLG2
UBC	POLG
POLE3	POLE4
DRAP1	POLE3
POLE3	POLE2
C14orf1	POLE2

UBC	POLE
POLE3	POLE
POLE2	POLE
MYC	POLE
MDM2	POLE
UBC	POLDIP3
UBC	POLD4
POLD1	POLD4
UBC	POLD3
POLD2	POLD3
POLD2	POLD1
XRCC1	POLB
UBC	POLB
PARP1	POLB
NEIL1	POLB
KAT7	POLB
APEX1	POLB
POLA2	PRIM1
POLA2	DCP2
POLA2	ASCC2
UBC	POGZ
SP1	POGZ
UBC	PODXL2
SLC9A3R2	PODXL
UBC	POC5
ZBTB16	PNRC2
RXRA	PNRC2
RARA	PNRC2
NR5A1	PNRC2
ESRRG	PNRC2
ESRRA	PNRC2
ESR1	PNRC2
DCP1A	PNRC2
UBC	PNPLA6
PHYHIP	PNPLA2
TP53	PNP
RBM48	PNP
PNP	CCDC90B
WDR3	PNO1
UBC	PNO1
RXRG	PNO1
PNO1	IGFBP5
PNO1	GSTP1
CSNK1E	PNO1

PNO1	BYSL
PNO1	ATP5C1
UBC	PNN
PNN	SRSF4
RNPS1	PNN
PNN	PNISR
KRT19	PNN
UBC	PNMAL1
CLPS	PNLIP
PNKP	XRCC4
XRCC1	PNKP
SMAD5	PNKP
MYOZ1	PNKP
PNKP	MCM3
LIG4	PNKP
PNKP	LIG3
UBC	PNKD
UBC	PMS2
MSH6	PMS2
MLH1	PMS2
FAN1	PMS2
EXO1	PMS2
BRIP1	PMS2
UBC	PMS1
FAN1	PMS1
UBC	PMPCB
PMPCA	PMPCB
FXN	PMPCB
UBC	PMPCA
UBC	PMM2
UBC	PMM1
RAB6A	PMM1
ZFYVE9	PML
ZBTB16	PML
USP7	PML
UBE2I	PML
UBC	PML
TP53	PML
TOPBP1	PML
PML	THRA
TDG	PML
SUMO3	PML
SUMO2	PML
SUMO1	PML

SP1 PML
PML SKI
SATB1 PML
RXRA PML
RNF4 PML
PML RB1
RARA PML
PIN1 PML
PML NR2C1
NCOR2 PML
MYC PML
MDM2 PML
PML MAPK1
PML KLHL20
JUN PML
HIPK2 PML
HDAC7 PML
HDAC2 PML
HDAC1 PML
DAXX PML
CTNNB1 PML
PML CSNK2A3
CREBBP PML
PML CCNT1
BANP PML
UBC PMF1-BGLAP
NFE2L2 PMF1-BGLAP
MIS12 PMF1-BGLAP
DSN1 PMF1-BGLAP
UBC PMEPA1
NEDD4 PMEPA1
UBC PMAIP1
PMAIP1 IRF3
UBC PM20D2
SEMA7A PLXNC1
PLXNB1 SEMA4D
RND3 PLXNB1
RND2 PLXNB1
PLXNB1 RND1
ARHGEF12 PLXNB1
ARHGEF11 PLXNB1
PLXNA2 IFT80
UBC PLXNA1
NRP1 PLXNA1

UBC PLXDC2
UBC PLSCR3
TRIP13 PLSCR3
PLSCR1 ZNF764
ZNF638 PLSCR1
PLSCR1 ZNF581
ZBTB16 PLSCR1
YIPF3 PLSCR1
VASP PLSCR1
UBC PLSCR1
SPRY2 PLSCR1
PLSCR1 SPG7
PLSCR1 SMCP
SF1 PLSCR1
PLSCR4 PLSCR1
PLSCR1 PLSCR3
PLSCR1 PHLDA1
PLSCR1 PGLS
OGDH PLSCR1
PLSCR1 NTN4
NOC4L PLSCR1
PLSCR1 NECAP2
LASP1 PLSCR1
KIAA1683 PLSCR1
PLSCR1 IP6K2
HOXA9 PLSCR1
HOXA1 PLSCR1
PLSCR1 HEY2
GPRIN2 PLSCR1
PLSCR1 GNAI2
GLRX3 PLSCR1
PLSCR1 FAM103A1
EGFR PLSCR1
EFEMP2 PLSCR1
PLSCR1 DLK2
PLSCR1 DHRS1
DAZAP2 PLSCR1
CRY1 PLSCR1
CRK PLSCR1
CPSF6 PLSCR1
CPSF3L PLSCR1
PLSCR1 CDC42EP1
PLSCR1 CCER1
PLSCR1 C10orf62

PLSCR1 C10orf54
C10orf10 PLSCR1
PLSCR1 ATG12
ADAMTSL4 PLSCR1
ABL1 PLSCR1
UBC PLS1
UBC PLRG1
PLRG1 SF3B1
EHMT2 PLOD3
DPPA2 PLOD3
CCDC85B PLOD3
UBC PLOD2
UBC PLOD1
PLNS100A1
TGFBR1PLK4
SMAD4 PLK4
SFNPLK4
MTUS2 PLK4
FAM46CPLK4
TRIP13 PLK3
TP53 PLK3
CHEK2 PLK3
UBC PLK2
TANK PLK1
RACGAP1 PLK1
PSMB1 PLK1
PSMA5 PLK1
PSMA2 PLK1
PLK1 NUDC
PLK1 KLHL22
PLK1 CSN2
PLK1 CLSPN
PLK1 BUB1B
PLK1 ASPM
SMAD2 PLIN3
UBC PLIN2
SPARC PLG
PLGF10
PLGCPB2
PLGCP
PLGCLEC3B
UBC PLEKHO2
UBC PLEKHM3
SERPINB9 PLEKHM1

TGFBR1	PLEKHJ1
ACVR1	PLEKHJ1
UBC	PLEKHG4B
PLEKHF2	PRKAR1A
CCDC85B	PLEKHF2
SMAD1	PLEKHB1
UBC	PLEKHA8
UBC	PLEKHA7
EEF1G	PLEKHA4
UBC	PLEK2
TGFBR1	PLEK
ACVR1	PLEK
VIM	PLEC
UBC	PLEC
SPTAN1	PLEC
SPTA1	PLEC
PAN2	PLEC
ITGB4	PLEC
ITGA6	PLEC
UBC	PLD3
UBC	PLD2
PRKCD	PLD2
PRKCA	PLD2
PLCG1	PLD2
PIP5K1A	PLD2
PEA15	PLD2
LCK	PLD2
GRB2	PLD2
FYN	PLD2
EGFR	PLD2
CAV1	PLD2
ALDOA	PLD2
ACTB	PLD2
UBC	PLD1
RALA	PLD1
PRKCA	PLD1
PKN3	PLD1
PKN1	PLD1
PIP5K1A	PLD1
PEA15	PLD1
CAV1	PLD1
PPP1CA	PLCL2
DSCAML1	PLCL2
PLCG2	LCK

PLCG2	GAB1
PLCG2	FLT1
PLCG2	CBLB
PLCG1	ZAP70
VAV1	PLCG1
UBC	PLCG1
PLCG1	TUB
PLCG1	TRPC3
PLCG1	SOS2
PLCG1	SNAP91
PLCG1	SHB
PRKD1	PLCG1
PLCG2	PLCG1
PLCG1	PKN2
PLCG1	PITPNA
PLCG1	PDGFRB
PLCG1	PDGFRA
PLCG1	NTRK2
PLCG1	NTRK1
PLCG1	LIFR
LCP2	PLCG1
LCK	PLCG1
LAT	PLCG1
PLCG1	KIT
PLCG1	KDR
PLCG1	ITK
IRS2	PLCG1
HCK	PLCG1
PLCG1	GNB2L1
PLCG1	GAB1
PLCG1	FLT1
ERBB3	PLCG1
EGFR	PLCG1
EEF1A1	PLCG1
PLCG1	BAG3
PLCG1	ALK
UBC	PLCD3
SKIL	PLCD3
UBC	PLCD1
UBC	PLCB3
SLC9A3R2	PLCB3
SLC9A3R1	PLCB2
MAP2K3	PLCB2
GNAQ	PLCB2

SLC9A3R1	PLCB1
GNAQ	PLCB1
PFN2	PLAUR
SERPINA5	PLAU
PLGPLAU
PLAUR	PLAU
SPARC	PLAT
SERPINI1	PLAT
SERPINE1	PLAT
LAMA3	PLAT
FGA	PLAT
CLEC3B	PLAT
ANXA2	PLAT
KPNA2	PLAG1
TRIP13	PLAC8
VCP	PLAA
UBC	PLAA
PLA2G1B	PLAA
PLA2G10	PLA2R1
UBQLN1	PLA2G16
UBC	PLA2G16
ANXA2	PLA1A
UBC	PKP4
PSEN1	PKP4
PGAM1	PKP4
PDZD2	PKP4
GABARAP	PKP4
ERBB2IP	PKP4
DSPPKP4
CDH5	PKP4
DSG2	PKP3
DSC3	PKP3
DSC2	PKP3
DSC1	PKP3
UBC	PKP2
KRT5	PKP2
DSPPKP2
DSG1	PKP2
DSC2	PKP2
DSC1	PKP2
DSG1	PKP1
PBX2	PKNOX1
UBC	PKN3
UBC	PKN2

PDK1	PKN2
VIM	PKN1
PKN1	NEUROD2
NEFL	PKN1
PKN1	NEFH
MARCKS	PKN1
MAPT	PKN1
CCDC85B	PKN1
AKAP9	PKN1
ACTN1	PKN1
XPO1	PKIA
UBE2D2	PJA2
UBC	PJA2
PITX2	PROP1
POU1F1	PITX1
NR5A1	PITX1
EGR1	PITX1
DVL3	PITX1
UBC	PITRM1
PTK2B	PITPNM2
CPNE4	PITPNM2
CPNE1	PITPNM2
PLK1	PITPNM1
AGTRAP	PITPNC1
UBC	PITPNB
LMO4	PITPNB
UBC	PITPNA
EGFR	PITPNA
UBC	PISD
UBC	PIP5K1C
ARF6	PIP5K1C
UBC	PIP5K1B
UBC	PIP5K1A
UBC	PIP4K2B
TNFRSF1A	PIP4K2B
TERT	PINX1
PINX1	MPZ
UBC	PINK1
TRAF6	PINK1
PINK1	PARK7
PARK2	PINK1
PINK1	CDC37
TSC22D4	PIN1
TP53	PIN1

PIN1	TBC1D4
SMAD2	PIN1
PIN1	SFPQ
PIN1	REPS1
PIN1	RAB4A
PIN1	PLK1
PIN1	PKMYT1
PIN1	MYC
PIN1	MED1
PIN1	MAPT
PIN1	MAP1S
PIN1	HADHA
PIN1	G3BP2
PIN1	ETV6
PIN1	EFTUD2
PIN1	DDX5
PIN1	CHPF
PIN1	CHAMP1
UBC	PIM1
SNX6	PIM1
SND1	PIM1
SHMT1	PIM1
NFATC1	PIM1
MDM2	PIM1
HSP90AA1	PIM1
DCTN1	PIM1
CDC25A	PIM1
CBX1	PIM1
PTPN6	PILRA
TEC	PIK3R3
IRS1	PIK3R3
ERBB3	PIK3R3
EGFR	PIK3R3
DRAP1	PIK3R3
PIK3CD	PIK3R2
KIT	PIK3R2
EGFR	PIK3R2
CD28	PIK3R2
AXL	PIK3R2
VAV1	PIK3R1
UBC	PIK3R1
PIK3R1	TYK2
PIK3R1	TRAT1
PIK3R1	TOM1L1

PIK3R1	TIE1
PIK3R1	SHB
PTPN6	PIK3R1
PIK3R1	PIK3CD
PIK3CB	PIK3R1
PIK3R1	PDGFRB
PIK3R1	PDGFRA
PIK3R1	NFKBIA
PIK3R1	MME
LCP2	PIK3R1
LCK	PIK3R1
LAT	PIK3R1
PIK3R1	KIT
PIK3R1	JAK1
IRS2	PIK3R1
PIK3R1	IRS1
PIK3R1	IL2RB
PIK3R1	IL1RAP
PIK3R1	IL13
PIK3R1	IFNAR1
PIK3R1	HRAS
PIK3R1	GHR
PIK3R1	GAB2
PIK3R1	GAB1
PIK3R1	FLT1
PIK3R1	FCGR2A
PIK3R1	ERBB4
ERBB3	PIK3R1
EGFR	PIK3R1
PIK3R1	CSF1R
CRKL	PIK3R1
CRK	PIK3R1
PIK3R1	CDC42
PIK3R1	CD28
CBLB	PIK3R1
PIK3R1	ARHGAP1
PIK3R1	ADAM12
SEC14L2	PIK3CG
PIK3R5	PIK3CG
TNPO2	PIK3CD
ASCC2	PIK3CD
PIK3CB	PRKCE
PIK3CB	MME
PIK3CB	HCK

PRKCD	PIK3CA
PIK3R3	PIK3CA
PIK3R2	PIK3CA
PIK3R1	PIK3CA
NRAS	PIK3CA
LCK	PIK3CA
IRS1	PIK3CA
HRAS	PIK3CA
GRB2	PIK3CA
GNAQ	PIK3CA
CBL	PIK3CA
APPL1	PIK3CA
UBC	PIK3C3
PIK3C3	PITPNA
PIK3C3	PIK3R4
PIK3C3	ATG14
UBC	PIK3C2B
EGFR	PIK3C2B
GRB2	PIK3AP1
UBC	PIH1D1
TSC22D4	PIH1D1
RUVBL2	PIH1D1
RUVBL1	PIH1D1
UBC	PIGW
UBC	PIGU
UBC	PIGT
PIGT	PIGK
UBC	PIGS
RAB3B	PIGR
CD79A	PIGR
SMAD1	PIGQ
UBC	PIGO
PIGQ	PIGH
PIGC	PIGH
UBC	PIEZO1
UBC	PICK1
PRKCA	PICK1
GRM7	PICK1
GRIK2	PICK1
EPHB2	PICK1
ASIC1	PICK1
UBC	PICALM
CLTC	PICALM
UBC	PIBF1

ZNF512B	PIAS4
UBE2I	PIAS4
UBC	PIAS4
TP53	PIAS4
TCF4	PIAS4
SUMO3	PIAS4
SUMO2	PIAS4
SUMO1	PIAS4
SNIP1	PIAS4
SMAD7	PIAS4
SMAD4	PIAS4
SMAD3	PIAS4
SMAD2	PIAS4
SMAD1	PIAS4
SKIL	PIAS4
SATB1	PIAS4
LEF1	PIAS4
HDAC1	PIAS4
ETS1	PIAS4
CALCOCO2	PIAS4
BRCA1	PIAS4
UBC	PIAS3
SUMO1	PIAS3
PIAS3	STAT3
SKIL	PIAS3
PGR	PIAS3
PIAS3	MITF
PIAS3	GFI1
ESR1	PIAS3
UBE2I	PIAS2
UBC	PIAS2
TP53	PIAS2
SUMO2	PIAS2
SUMO1	PIAS2
PML	PIAS2
PARK7	PIAS2
NAV2	PIAS2
MYC	PIAS2
DNMT3A	PIAS2
DDX39B	PIAS2
AKT1	PIAS2
UBC	PIAS1
TP53	PIAS1
SUMO1	PIAS1

PIAS1	STAT1
SNIP1	PIAS1
SMAD4	PIAS1
SMAD1	PIAS1
SKIL	PIAS1
PGR	PIAS1
PIAS1	MBD1
DNMT3A	PIAS1
PIAS1	CSRP2
PIAS1	CHUK
UBC	PI4KA
UBC	PI4K2B
UBC	PI4K2A
CELA1	PI3
PDE9A	PHYHIPL
PHYHIP	PRMT5
PHYHIP	MED8
PHYHIP	HNRNPA1
PHYHIP	HDAC11
PHYHIP	C14orf1
UBC	PHRF1
USP46	PHLPP1
USP1	PHLPP1
UBC	PHLPP1
SUGT1	PHLPP1
UBC	PHLDB2
MAPT	PHKG1
CALM1	PHKG1
HNRNPC	PHKB
UBC	PHIP
UBC	PHGDH
TNFRSF1B	PHGDH
UBC	PHF5A
SF3B1	PHF5A
SF3A2	PHF5A
PRPF31	PHF5A
LPXN	PHF21A
HDAC2	PHF21A
UBC	PHF20L1
HIST4H4	PHF20
HIST1H3A	PHF20
UBC	PHF2
UBC	PHF14
SUMO2	PHF14

UBC	PHF12
SIN3A	PHF12
MORF4L1	PHF12
UBC	PHF10
PHF10	SMARCA2
TP53	PHF1
THAP1	PHF1
SUZ12	PHF1
EZH2	PHF1
TMEM70	PHC2
SIAH1	PHC2
SFMBT1	PHC2
SCMH1	PHC2
RPL7	PHC2
RNF2	PHC2
RING1	PHC2
PHC1	PHC2
PCGF2	PHC2
MCRS1	PHC2
MCM2	PHC2
MAPKAPK2	PHC2
LMO3	PHC2
FHL3	PHC2
CBX8	PHC2
CBX7	PHC2
CBX6	PHC2
CBX4	PHC2
CBX2	PHC2
BSDC1	PHC2
AFG3L2	PHC2
CBX8	PHC1
SUMO2	PHAX
PPP1CA	PHACTR3
PDLIM1	PGS1
GABARAP	PGS1
UBC	PGRMC2
NCOR2	PGR
UBC	PGP
UTRN	PGM5
UBC	PGM3
UBC	PGM2
UBC	PGM1
S100B	PGM1
S100A1	PGM1

UBC	PGLS
MDFI	PGLS
UBC	PGK2
RHOB	PGGT1B
VEGFA	PGF
FLT1	PGF
UBC	PGD
GBE1	PGD
AHSA1	PGD
UBC	PGAP1
ZBTB16	PGAM5
UBC	PGAM5
SETDB1	PGAM5
KEAP1	PGAM5
HSPA1A	PGAM5
CUL3	PGAM5
CDK2	PGAM5
BCL2L1	PGAM5
VCL	PGAM2
UBC	PGAM2
PFN2	IK
RHOQ	PFN1
C14orf1	PFN1
UBC	PFKP
DYNLL1	PFKP
UBC	PFKM
PFKP	PFKM
PFKL	PFKM
DYNLL1	PFKM
CAV3	PFKM
UBC	PFKL
TRAF2	PFKL
TNFRSF1A	PFKL
TAB2	PFKL
COPS6	PFKL
YWHAQ	PFKFB2
UBC	PFKFB2
PRKAA2	PFKFB2
PRKAA1	PFKFB2
UBC	PFDN6
PFDN6	RPAP3
PFDN6	PFDN5
PFDN6	PFDN4
PFDN6	PFDN2

MYC	PFDN5
UBC	PFDN4
UBC	PFDN2
PFDN2	RPAP3
PFDN5	PFDN2
PFDN4	PFDN2
PFDN2	PFDN1
UBC	PFDN1
PFDN1	SYNDIG1
PSME1	PFDN1
PFDN5	PFDN1
MED31	PFDN1
HAP1	PFDN1
PFDN1	DEFA1
ACTB	PFDN1
THBD	PF4
SRGN	PF4
SDC2	PF4
PHYH	PEX7
PEX5	PEX7
PEX13	PEX7
PEX6	PEX1
PEX5	HACL1
PEX5	GSTK1
PEX5	ECI2
PEX5	DDO
UBC	PEX3
PEX19	PEX3
SUFU	PEX26
C14orf1	PEX2
UBC	PEX19
SMAD2	PEX19
PEX19	PXMP4
PEX19	PEX2
PEX19	PEX16
PEX19	PEX13
PEX19	PEX12
PEX19	PEX11A
PEX19	PEX10
ABCD3	PEX19
PEX19	ABCD2
ZHX1	PEX14
UBC	PEX14
SMARCC2	PEX14

PEX7	PEX14
PEX5	PEX14
PEX19	PEX14
PEX13	PEX14
MED8	PEX14
HDAC1	PEX14
PEX5	PEX13
PEX5	PEX12
PEX12	PEX10
UBC	PEX11B
PEX19	PEX11B
PEX5	PEX10
PEX2	PEX10
UBC	PEX1
UBC	PET100
UBC	PES1
PES1	BOP1
CSNK1E	PER3
CRY1	PER3
TIMELESS	PER2
CSNK1E	PER2
CSNK1D	PER2
CRY2	PER2
CRY1	PER2
TIMELESS	PER1
PER2	PER1
CSNK1E	PER1
CSNK1D	PER1
CRY2	PER1
CRY1	PER1
UBC	PEPD
UBC	PELP1
UBC	PELI3
TRAF6	PELI3
UBC	PELI2
IRAK4	PELI2
PELI1	IRAK4
SIAH2	PEG3
SIAH1	PEG3
ZNF512B	PEG10
UBC	PEG10
PEX5	PECR
RAF1	PEBP4
UBC	PEBP1

STC2	PEBP1
RAF1	PEBP1
PRKCZ	PEBP1
PRKCG	PEBP1
PRKCD	PEBP1
PRKCB	PEBP1
PRKCA	PEBP1
MED10	PEBP1
MAPK1	PEBP1
MAP3K7	PEBP1
MAP2K1	PEBP1
ADRBK1	PEBP1
UBC	PEA15
FADD	PEA15
PDZK1	PDZK1IP1
COPS6	PDZK1IP1
PDZK1	SLC22A5
PDZK1	SLC22A4
PDZK1	SLC22A11
PDZK1	SLC17A1
SCARB1	PDZK1
PEX5	PDZK1
GFAP	PDZK1
CLCN3	PDZK1
CFTR	PDZK1
PDZK1	AKAP10
UBC	PDZD8
CFTR	PDZD3
UBC	PDXK
UBC	PDXDC1
PDX1	NEUROD1
UBC	PDS5B
CDCA5	PDS5B
UBC	PDS5A
CDCA5	PDS5A
UBC	PDRG1
RPAP3	PDRG1
UBC	PDPR
UBC	PDP2
UBE2I	PDLIM7
UBC	PDLIM7
PDLIM7	SPP1
PDLIM7	SMURF1
PDLIM7	PRKD2

PDLIM7	GFAP
PDLIM7	GEM
UBC	PDLIM5
TRIM39	PDLIM5
CACNA1B	PDLIM5
UBC	PDLIM4
VIM	PDLIM1
UBC	PDLIM1
AGK	PDLIM1
ACTN4	PDLIM1
ACTN1	PDLIM1
UBC	PDK4
PDK1	PDK2
PDHX	PDK2
HTT	PDK2
HIC2	PDK2
UBC	PDIK1L
UBC	PDIA6
UBC	PDIA4
APOB	PDIA4
VCL	PDIA3
UBC	PDIA3
TAP1	PDIA3
PXN	PDIA3
CALR	PDIA3
APP	PDIA3
VAV1	PDGFRB
UBC	PDGFRB
PDGFRB	TYK2
STAT1	PDGFRB
SLC9A3R1	PDGFRB
SHB	PDGFRB
PTPN12	PDGFRB
PDGFRB	PIK3R2
JAK1	PDGFRB
ITGB3	PDGFRB
GRB14	PDGFRB
FYN	PDGFRB
EGFR	PDGFRB
COPB1	PDGFRB
UBC	PDGFRA
SLC9A3R1	PDGFRA
SHB	PDGFRA
PDGFRB	PDGFRA

ITGB3	PDGFRA
GRB14	PDGFRA
CRK	PDGFRA
PDGFRA	PDGFC
PDGFRB	PDGFB
PDGFRA	PDGFB
HSPG2	PDGFB
HRAS	PDGFB
COL1A1	PDGFB
PDE6D	PDE6B
ARL2	PDE6D
UBC	PDE4D
SPTAN1	PDE4D
PDE4D	PIK3CG
PDE4D	LYN
GRB2	PDE4D
GNB2L1	PDE4D
FYN	PDE4D
ARRB2	PDE4D
PDE4D	ARRB1
ABL1	PDE4D
WDR3	PDE10A
UBC	PDCL3
TCP1	PDCL
PSMC5	PDCL
GNB1	PDCL
ADRBK1	PDCL
VPS4B	PDCD6IP
PDCD6IP	ALG2
UBC	PDCD6
PDCD6	TIMM22
PDCD6	SEC31A
PDCD6IP	PDCD6
MAP3K5	PDCD6
CPNE4	PDCD6
CPNE2	PDCD6
CPNE1	PDCD6
UBC	PDCD5
KAT5	PDCD5
EEF1G	PDCD5
UBC	PDCD4
EIF4A1	PDCD4
UBC	PDCD2L
UBC	PDCD2

PDCD1	PDCD1LG2
PDCD10	CCM2
PDCD1	CD274
UBC	PDAP1
PDGFRB	PDAP1
PDGFB	PDAP1
UBC	PCYT1A
HSPA5	PCSK7
UBC	PCSK6
SCG5	PCSK2
REN	PCSK1
FBP1	PCNXL4
UBC	PCNXL3
UBC	PCNX
UBC	PCNT
TUBG1	PCNT
DISC1	PCNT
CALM1	PCNT
AKAP9	PCNT
UBC	PCNP
YBX1	PCNA
XRCC6	PCNA
PCNA	XRCC5
XRCC1	PCNA
WRN	PCNA
USP1	PCNA
UNG	PCNA
UBE2B	PCNA
UBD	PCNA
UBC	PCNA
UBA52	PCNA
SUMO1	PCNA
SPRTN	PCNA
PCNA	SETD8
RPA1	PCNA
RNF8	PCNA
RFC5	PCNA
PCNA	RFC4
RFC3	PCNA
RFC2	PCNA
RFC1	PCNA
REV1	PCNA
PCNA	RAD9A
RAD18	PCNA

PRKDC	PCNA
PCNA	POLN
PCNA	POLL
POLH	PCNA
POLE	PCNA
POLD3	PCNA
POLD2	PCNA
POLD1	PCNA
PCNA	POLB
PCNA	PMS2
PARP1	PCNA
MYBBP1A	PCNA
PCNA	MSH6
PCNA	MSH3
MDM2	PCNA
PCNA	LIG1
PCNA	KIAA0101
KCTD13	PCNA
ING1	PCNA
HUS1	PCNA
HLTF	PCNA
HDAC1	PCNA
PCNA	GADD45B
PCNA	GADD45A
FEN1	PCNA
EXO1	PCNA
PCNA	ERCC5
PCNA	DSCC1
DNTTIP2	PCNA
DNMT1	PCNA
DHX9	PCNA
DDX17	PCNA
PCNA	CUL4A
CUL2	PCNA
CHTF18	PCNA
CHAF1A	PCNA
CDKN1C	PCNA
CDK6	PCNA
CDK5	PCNA
CDK4	PCNA
CDK2	PCNA
CDC6	PCNA
PCNA	CDC25C
PCNA	CDAN1

CCNO	PCNA
PCNA	CCND3
CCNB1	PCNA
APEX1	PCNA
ANXA2	PCNA
UBC	PCMTD2
UBC	PCMT1
CHD3	PCMT1
UBC	PCM1
PCNT	PCM1
KRT19	PCM1
KRT15	PCM1
KIAA0368	PCM1
ING5	PCM1
HAP1	PCM1
EXOC8	PCM1
EPS8	PCM1
CEP72	PCM1
CCDC53	PCM1
BBS4	PCM1
ABI2	PCM1
UBC	PCK2
UBC	PCK1
UBC	PCID2
RNF2	PCGF5
RING1	PCGF5
CSNK2A2	PCGF5
UBE2I	PCGF2
UBC	PCGF2
RNF2	PCGF2
RING1	PCGF2
PCGF2	PHC3
PHC1	PCGF2
CBX8	PCGF2
CBX7	PCGF2
CBX4	PCGF2
CBX2	PCGF2
UBC	PCGF1
SKP1	PCGF1
RYBP	PCGF1
RNF2	PCGF1
RING1	PCGF1
KDM2B	PCGF1
CALCOCO2	PCGF1

UBC PCDHB5
UBC PCDHB2
SMAD3 PCDH1
UBC PCBP4
UBC PCBP2
WDYHV1 PCBD1
UBC PCBD1
NUDT18PCBD1
NIF3L1 PCBD1
LNX1 PCBD1
HNF1A PCBD1
FXR2 PCBD1
DVL2 PCBD1
PBX2 SMYD3
PBX2 SKIV2L2
PBX2 RPS6KC1
PBX2 MCRS1
PBX2 HOXA9
PBX2 BAIAP2
PBX2 PBX1
PAX6 PBX1
MEIS2 PBX1
MEIS1 PBX1
HOXB1 PBX1
HOXA9 PBX1
ZNF512B PBRM1
UBC PBRM1
TRIP13 PBLD
SMAD2 PAXIP1
PAXIP1 PAX2
MSX1 PAX9
KDM5B PAX9
NKX2-1 PAX8
PAX6 SOX2
PAX5 TLE4
ID3 PAX5
ETS1 PAX5
DAXX PAX5
UBC PAX3
TRIM28 PAX3
SOX10 PAX3
MEOX2 PAX3
DAXX PAX3
MEOX2 PAX1

MEOX1	PAX1
WT1	PAWR
UBC	PAWR
RRAS2	PAWR
PRKCZ	PAWR
PRKCI	PAWR
DAPK3	PAWR
ATP5C1	PAWR
BRIP1	PATZ1
UBC	PASK
UBC	PARVG
ILK	PARVB
UBC	PARVA
ILK	PARVA
UBC	PARPBP
UBC	PARP9
PARP1	PARP3
UBC	PARP2
UBC	PARP14
UBC	PARP12
GTPBP3	PARP11
UBC	PARP10
MYC	PARP10
PARP1	ZNF423
PARP1	XRCC1
WRN	PARP1
UBC	PARP1
TRAF6	PARP1
TRAF2	PARP1
TP53	PARP1
PARP1	TOP1
PARP1	THRA
SUMO1	PARP1
RXRA	PARP1
PARP1	RPS3A
PARP1	POLA2
PARP1	NFKB2
PARP1	NFKB1
MYBL2	PARP1
PARP1	MED6
PARP1	HIST1H3A
HDAC2	PARP1
HDAC1	PARP1
PARP1	H3F3A

PARP1	H2AFY
ESR1	PARP1
PARP1	CHD1L
PARP1	CENPB
PARP1	CENPA
PARP1	CDK8
PARP1	CASP3
BUB3	PARP1
PARP1	APTX
PARP1	APLF
UBC	PARN
MPHOSPH6	PARN
UBC	PARK7
PTEN	PARK7
MAP3K5	PARK7
PARK2	VDAC3
VCL	PARK2
UBE2N	PARK2
PARK2	UBE2L6
UBE2L3	PARK2
UBC	PARK2
TRIP13	PARK2
TRAF2	PARK2
PARK2	STUB1
PARK2	SNCAIP
SEPT4	PARK2
PARK2	RHOT1
PARK2	RAD1
PARK2	PSMD4
PARK2	PARK7
PARK2	MFN2
MAPT	PARK2
HSPA8	PARK2
PARK2	GPR37
PARK2	FBP1
PARK2	ATXN3
PARK2	APP
PARD6G	PRKCI
PARD3	PARD6G
CDC42	PARD6G
RHOQ	PARD6B
PARD3	PARD6B
YWHAG	PARD3B
SMAD2	PARD3

DYNLL1	PARD3
UBC	PAQR7
UBC	PAPSS2
UBC	PAPSS1
ZNF512B	PAPPA2
SMAD4	PAPPA2
IGFBP5	PAPPA2
PLGPAPPA	
UBC	PAPOLA
SNRPA	PAPOLA
SMAD2	PAPOLA
RELPAPOLA	
CPSF3	PAPOLA
UBC	PANX1
UBC	PANK3
UBC	PANK2
PAN3	PAN2
CCT7	PAN2
UBC	PAM
RASSF9	PAM
UBC	PALD1
LZTS2	PAK7
FHL3	PAK7
CDC42	PAK7
CDC42	PAK6
YWHAQ	PAK4
YWHAG	PAK4
UBC	PAK4
LIMK1	PAK4
GATAD2A	PAK4
DLST	PAK4
CDC42	PAK4
UBC	PAK1
TGFBR1	PAK1
SMAD4	PAK1
SMAD2	PAK1
SMAD1	PAK1
RHOJ	PAK1
RAF1	PAK1
PXN	PAK1
PPM1F	PAK1
PLCG1	PAK1
PAK1	PAK1IP1
FOXO1	PAK1

EGFR	PAK1
DYNLL1	PAK1
COL1A1	PAK1
CDC42	PAK1
BAD	PAK1
ARHGEF7	PAK1
ARHGDIA	PAK1
ACVR1	PAK1
ABI3	PAK1
UBC	PAIP1
PAXIP1	PAGR1
ZAP70	PAG1
VAV1	PAG1
SYK	PAG1
SLC9A3R1	PAG1
RASA1	PAG1
PTPN6	PAG1
LYN	PAG1
LCP2	PAG1
LCK	PAG1
GRB2	PAG1
FYN	PAG1
ABL1	PAG1
ZHX1	PAFAH1B3
ZBTB16	PAFAH1B3
XRCC6	PAFAH1B3
UNC119	PAFAH1B3
UBC	PAFAH1B3
TP53	PAFAH1B3
SETDB1	PAFAH1B3
SDCBP2	PAFAH1B3
RBM48	PAFAH1B3
PLEKHM1	PAFAH1B3
PAFAH1B2	PAFAH1B3
PAFAH1B1	PAFAH1B3
MED31	PAFAH1B3
LNX1	PAFAH1B3
IGSF21	PAFAH1B3
GBP2	PAFAH1B3
ECH1	PAFAH1B3
DDX24	PAFAH1B3
COPS6	PAFAH1B3
CHMP7	PAFAH1B3
CCDC90B	PAFAH1B3

C14orf1 PAFAH1B3
BRD7 PAFAH1B3
UBC PAFAH1B2
PAFAH1B1 PAFAH1B2
PAFAH1B1 NDEL1
PAF1 WDR61
UBC PAF1
SUPT16H PAF1
POLR2A PAF1
LEO1 PAF1
PAF1 CTR9
CDC73 PAF1
COPS6 PAEP
TP53 PADI4
HIST4H4 PADI4
UBC PACSIN3
UBC PACSIN2
PACSIN3 PACSIN2
WASL PACSIN1
SYNJ1 PACSIN1
PACSIN3 PACSIN1
PACSIN2 PACSIN1
KHDRBS1 PACSIN1
KAT7 PACSIN1
HTT PACSIN1
DNM1 PACSIN1
ARHGAP17 PACSIN1
UBC PABPN1
SNW1 PABPN1
UBC PABPC4
SMAD9 PABPC4
PABPC4 PHLDA1
PABPC4 EIF2AK2
YWHAG PABPC1
WBP4 PABPC1
PABPC1 VCL
UPF1 PABPC1
UBC PABPC1
PABPC1 TOB1
PABPC1 TNRC6C
PABPC1 SYCP3
SUMO2 PABPC1
SNRNP200 PABPC1
PIN1 PABPC1

PABPC1	PCBP2
PABPC1	PAN3
PABPC1	PAN2
PABPC1	PAIP2
PABPC1	PAIP1
NFKB2	PABPC1
PABPC1	MAP3K14
PABPC1	HNRNPD
HNRNPA1	PABPC1
GSPT1	PABPC1
PABPC1	GNB2L1
ESR1	PABPC1
PABPC1	EIF4G3
EIF4G1	PABPC1
DHX15	PABPC1
PABPC1	BRCA1
APBB1	PABPC1
PSMD7	PAAF1
PSMD11	PAAF1
PSMC3	PAAF1
PSMC2	PAAF1
UBC	PA2G4
SUMO1	PA2G4
MDM2	PA2G4
HDAC2	PA2G4
ERBB3	PA2G4
UBQLN1	P4HB
UBC	P4HB
P4HB	TG
PXN	P4HB
P4HB	PDIA3
P4HB	P4HA3
P4HB	ERO1LB
COL1A1	P4HB
P4HB	COL10A1
UBC	P4HA2
ITGB5	P2RY2
ITGB3	P2RY2
CD47	P2RY2
SLC9A3R1	P2RY1
ADORA1	P2RY1
CDH5	P2RX6
UBC	OXA1L
MDFI	OTX1

CHIC2	OTX1
UBC	OTUD7B
UBC	OTUB2
TRAF6	OTUB2
UBE2N	OTUB1
UBC	OTUB1
OTUB1	OTUD6B
UBC	OSTM1
RGS19	OSTM1
RGS17	OSTM1
UBC	OSTF1
UBC	OSTC
UBC	OSMR
OSM	OSMR
JAK2	OSMR
JAK1	OSMR
IL6ST	OSMR
IL6ST	OSM
COL4A1	OSM
UBC	OSBPL9
UBC	OSBPL8
UBC	OSBPL5
UBC	OSBPL3
UBC	OSBPL2
UBC	OSBPL11
UBC	OSBPL10
VIM	OSBP2
UBC	OSBP
ZNF512B	OS9
UBC	OS9
SMAD2	OS9
SERPINA1	OS9
SEL1L	OS9
HSP90B1	OS9
OS9	EGLN1
DERL2	OS9
UBC	ORMDL3
UBC	ORMDL2
ORC5	ORC4
UBC	ORC3
ORC5	ORC3
ORC4	ORC3
MCM7	ORC3
ORC3	CDC45

ORC2	RPA1
ORC2	ORC5
ORC2	ORC4
ORC2	ORC3
ORC2	MCM4
ORC2	MCM3
ORC2	MCM2
ORC2	LRWD1
ORC2	HMGA1
ORC2	CDC6
ORC2	CDC45
ORAI2	MED31
COPS6	ORAI2
STIM1	ORAI1
UBC	OPTN
TBK1	OPTN
TBC1D17	OPTN
RAB11A	OPTN
HTT	OPTN
OSGEP	OPTC
UBC	OPRM1
RGS19	OPRL1
PNOC	OPRL1
GNA14	OPRL1
SLC9A3R1	OPRK1
GNA15	OPRK1
UBC	OPRD1
OPRM1	OPRD1
OPRK1	OPRD1
GNAI2	OPRD1
GNAI1	OPRD1
GNA15	OPRD1
CANX	OPRD1
UBC	OPA1
KAT2B	ONECUT1
UBC	OLR1
ASCC2	OLA1
UBC	OIP5
PRKCA	OGG1
CHGB	OGG1
UBC	OGFOD1
UBC	ODF2
ODF1	SPAG4
ODF1	ODF2

UBC	ODC1
UBC	OCLN
TJP2	OCLN
OCLN	TJP1
OCLN	TGFBR1
SMAD4	OCLN
UBC	OCIAD2
TTN	OBSCN
ANK2	OBSCN
ANK1	OBSCN
ODC1	OAZ2
UBC	OAZ1
ODC1	OAZ1
UBC	OAT
EIF1B	OAT
UBC	OASL
UBC	OARD1
UBC	NYNRIN
UBC	NXT1
NXF1	NXT1
NRXN1	NXPH1
UBC	NXN
UBC	NVL
UBC	NUPL2
UBC	NUPL1
NXF1	NUP98
UBC	NUP93
MYC	NUP93
UBC	NUP88
NUP98	NUP88
NUP214	NUP88
VIM	NUP85
UBC	NUP85
EEF1G	NUP85
UBC	NUP62
THAP1	NUP62
PBX2	NUP62
NUP62	NXF1
NUP62	NUPL1
NUP62	NUP54
NUP62	IK
NUP62	DTNB
NUP62	CCDC53
UBC	NUP50

NUP153	NUP50
KPNA2	NUP50
UBC	NUP37
UBC	NUP35
SMAD2	NUP214
NUP214	NXF1
NUP214	NUP98
UBC	NUP210
UBC	NUP160
EXOSC1	NUP160
UBC	NUP155
UBC	NUP153
SMAD2	NUP153
NUP153	NXF1
UBC	NUP133
NUP98	NUP133
UBC	NUP107
NUP107	SEH1L
SEC13	NUP107
NUP98	NUP107
NUP85	NUP107
NUP37	NUP107
NUP214	NUP107
NUP107	NUP160
NUP107	NUP133
TRAF6	NUMBL
TAB2	NUMBL
UBC	NUMB
TP53	NUMB
SIAH1	NUMB
MDM2	NUMB
LNX2	NUMB
LNX1	NUMB
ITCH	NUMB
EGFR	NUMB
DPYSL2	NUMB
APP	NUMB
AP2A1	NUMB
UBC	NUMA1
NUMA1	ACTR1A
UBC	NUFIP2
UBC	NUF2
UBC	NUDT6
UBC	NUDT5

SOX30	NUDT3
RPLP1	NUDT3
RPL8	NUDT3
RAB17	NUDT3
ASB13	NUDT3
UBC	NUDT19
NUDT18	PAICS
KRT15	NUDT18
NUDT18	KCTD13
NUDT18	HSD17B14
NUDT18	GSE1
UBC	NUDT16L1
UBC	NUDCD3
UBC	NUDCD1
PAFAH1B1	NUDC
UBC	NUBP1
NUBP2	NUBP1
NDN	NUBP1
UBC	NUB1
NUB1	SNCAIP
PSMD4	NUB1
NEDD8	NUB1
TGFBR1	NUAK2
SMAD4	NUAK2
SMAD2	NUAK2
ACVR1	NUAK2
NTS	NTSR1
NTS	NTSR2
NTF3	NTRK3
MAPK1	NTRK3
UBC	NTRK2
NTRK2	NTF3
DYNLL1	NTRK2
UBC	NTRK1
TRAF6	NTRK1
NEDD4	NTRK1
NTRK1	KIDINS220
CRK	NTRK1
UBC	NTPCR
ADAMTSL4	NTN4
UNC5C	NTN1
NEO1	NTN1
UBC	NTMT1
DIS3	NTMT1

NTRK2	NTF4
FN1	NT5E
UBC	NT5DC2
TRAF2	NT5DC2
TRAF1	NT5DC2
UBC	NT5DC1
UBC	NT5C3A
UBC	NT5C2
NUDT18	NT5C2
NOC4L	NT5C2
NME7	NT5C2
MOB3B	NT5C2
FXR2	NT5C2
UBC	NSUN5
MTERFD2	NSUN4
UBC	NSUN2
PFDN5	NSUN2
UBC	NSMCE4A
NDNL2	NSMCE4A
UBC	NSMCE1
SMC6	NSMCE1
SMC5	NSMCE1
NDNL2	NSMCE1
EID3	NSMCE1
TNFRSF1A	NSMAF
UBC	NSL1
NSL1	SUGT1
NSL1	MIS12
NSL1	DSN1
NSL1	BUB1
VCPIP1	NSFL1C
VCP	NSFL1C
UBC	NSFL1C
TARDBP	NSFL1C
SRP14	NSFL1C
NSFL1C	RPL22
NSFL1C	FAM32A
UBC	NSF
NSF	CD28
C14orf1	NSF
ZNF496	NSD1
UBC	NSD1
DDX24	NRXN2
NRXN1	PDZD2

NRXN1	MYO16
ANKZF1	NRSN2
VEGFA	NRP2
NRP1	VEGFA
UBC	NRP1
TGFBR2	NRP1
NRP1	SEMA3A
KDR	NRP1
YWHAH	NRIP1
UBC	NRIP1
TRAF2	NRIP1
TNFRSF14	NRIP1
NRIP1	THRA
RXRB	NRIP1
RXRA	NRIP1
RARA	NRIP1
NRIP1	PPARG
NRIP1	PPARA
NRIP1	NR5A1
NRIP1	NR1H3
KAT2B	NRIP1
HDAC1	NRIP1
NRIP1	ESRRG
ESR2	NRIP1
ESR1	NRIP1
CTBP1	NRIP1
CEP70	NRIP1
CCDC85B	NRIP1
ERBB4	NRG2
ERBB3	NRG2
ERBB4	NRG1
PPRC1	NRF1
PPARGC1A	NRF1
MAFK	NRF1
MAFF	NRF1
CXCR4	NRF1
IGSF21	NREP
UBC	NRDE2
UBC	NRD1
MOB1A	NRD1
HBEGF	NRD1
PTPRB	NRCAM
MAGI3	NRCAM
CNTN2	NRCAM

UBC NRBP1
TSC22D4 NRBP1
MLF1 NRBP1
RASGRP2 NRAS
ACTB NRAP
UIMC1 NR6A1
GNB5 NR6A1
SP1 NR5A1
NCOA1 NR5A1
RXRB NR4A2
RXRA NR4A2
UBC NR4A1
RXRA NR4A1
NCOR2 NR4A1
NR4A1 FXYD3
BCL2 NR4A1
UBC NR3C2
TRIM24 NR3C2
NR3C1 YWHAH
UBE3A NR3C1
UBC NR3C1
TSG101 NR3C1
TRIM28 NR3C1
NR3C1 TP53BP1
TP53 NR3C1
TBP NR3C1
SUMO2 NR3C1
SUMO1 NR3C1
STUB1 NR3C1
NR3C1 STAT5B
NR3C1 STAT5A
NR3C1 STAT3
NR3C1 SMARCE1
SMARCC1 NR3C1
SMARCA4 NR3C1
SMARCA2 NR3C1
SMAD3 NR3C1
NR3C1 SLC25A4
SFN NR3C1
NR3C1 SELENBP1
NR3C1 RELA
RAF1 NR3C1
NR3C1 PTMS
PPARGC1A NR3C1

POU2F1	NR3C1
NR3C1	ONECUT1
NR3C1	NRIP1
NR3C1	NR2F6
NFKB1	NR3C1
NCOR2	NR3C1
NCOA6	NR3C1
NCOA3	NR3C1
NCOA2	NR3C1
NCOA1	NR3C1
NCL	NR3C1
MED1	NR3C1
NR3C1	MAPK1
JUN	NR3C1
IDE	NR3C1
HSPA1A	NR3C1
NR3C1	HSP90AA1
NR3C1	HOXB1
HDAC1	NR3C1
NR3C1	FKBP4
DAXX	NR3C1
NR3C1	DAP3
CREBBP	NR3C1
NR3C1	CREB1
NR3C1	CEBPB
NR3C1	CEBPA
CALR	NR3C1
UBC	NR2F6
NCOA1	NR2F6
ESR1	NR2F6
NR2F6	ANGPTL1
UBC	NR2F2
SQSTM1	NR2F2
PIAS1	NR2F2
NR2F6	NR2F2
MYOD1	NR2F2
HIPK3	NR2F2
HDAC1	NR2F2
FAM46A	NR2F2
UBC	NR2F1
SP1	NR2F1
RXRA	NR2F1
ISL1	NR2F1
HNF4A	NR2F1

GTF2B	NR2F1
ESR1	NR2F1
BCL11B	NR2F1
RXRB	NR2E3
RARA	NR2E3
NR2C2	NR2C1
HDAC4	NR2C1
ESR1	NR2C1
RXRA	NR1I3
RXRB	NR1I2
RXRA	NR1I2
PPARGC1A	NR1I2
POU1F1	NR1I2
NCOR2	NR1I2
NCOA1	NR1I2
RXRA	NR1H4
RXRG	NR1H3
RXRA	NR1H3
RARA	NR1H3
PPARA	NR1H3
NCOA6	NR1H3
NCOR2	NR1H2
HDAC1	NR1D2
NCOR2	NR1D1
PYY	NPY2R
NPY	NPY2R
PYY	NPY1R
NPY	NPY1R
LSM7	NPY1R
NPY	PMCH
NR1H2	NPY
NPY	NPY5R
MC4R	NPY
NPY	GHRL
NPBWR1	NPW
UBC	NPTN
UBC	NPRL3
UBC	NPRL2
UBC	NPR3
NPPC	NPR3
NPPB	NPR3
PRKG1	NPR1
NPPB	NPR1
NPR2	NPPC

NPR2	NPPB
NPR3	NPPA
NPR2	NPPA
NPR1	NPPA
MME	NPPA
NPM1	NPM3
NPM1	YBX1
UBC	NPM1
TP53	NPM1
MDM2	NPM1
NPM1	MAP3K14
NPM1	HMGA1
UBC	NPLOC4
KIRREL	NPHS2
NPHS1	YES1
NPHS1	NPHS2
NCK1	NPHS1
NPHS1	LYN
KIRREL	NPHS1
NPHS1	IQGAP1
FYN	NPHS1
CD2AP	NPHS1
UBC	NPHP4
NPHP1	NPHP4
NPHP1	NPHP3
TNK2	NPHP1
INVS	NPHP1
UBC	NPEPPS
DHDDS	NPC2
UBC	NPC1
NPW	NPBWR2
UBC	NPAT
TRRAP	NPAT
CCNE1	NPAT
ARNTL	NPAS2
NOXA1	NOXO1
CYBA	NOXO1
ITGB1	NOV
NOV	INS
NOV	IGF2
FN1	NOV
UBC	NOTCH3
SNW1	NOTCH3
KAT2B	NOTCH3

YY1	NOTCH1
UBC	NOTCH1
UBA52	NOTCH1
SNW1	NOTCH1
RELA	NOTCH1
RBPJ	NOTCH1
PSEN1	NOTCH1
PIK3CG	NOTCH1
NUMB	NOTCH1
NOV	NOTCH1
NFKB1	NOTCH1
MFNG	NOTCH1
MDM2	NOTCH1
MAML1	NOTCH1
LEF1	NOTCH1
LCK	NOTCH1
KAT5	NOTCH1
KAT2B	NOTCH1
JAG2	NOTCH1
JAG1	NOTCH1
ITCH	NOTCH1
GSK3B	NOTCH1
FBXW7	NOTCH1
DTX1	NOTCH1
CNTN1	NOTCH1
UBC	NOSIP
NOS3	NOSIP
PPP2R5A	NOS3
NOS3	NOSTRIN
UCHL5	NOS2
UBC	NOS2
SPSB4	NOS2
SPSB2	NOS2
NOS2	SPSB1
SLC9A3R1	NOS2
HSP90AA1	NOS2
FOS	NOS2
CUL5	NOS2
PRKCA	NOS1
DYNLL1	NOS1
NOS1	ARG1
UBC	NOP9
UBC	NOP58
NOP58	FTSJ3

UBC	NOP56
U2AF1	NOP56
NOP56	RPL7
RPL18	NOP56
NOP58	NOP56
NOLC1	NOP56
GNL3	NOP56
EEF1A1	NOP56
NOP56	EBNA1BP2
DHX9	NOP56
UBC	NOP16
UBC	NOP14
PAXIP1	NOP14
PPP1CA	NOM1
NOLC1	POLR1A
UBC	NOL9
UBC	NOL7
SNAPC5	NOL4
SLC25A38	NOL12
SF3B3	NOL12
SAP18	NOL12
CDK4	NOL12
UBC	NOL10
BMP7	NOG
BMP5	NOG
BMP4	NOG
BMP2	NOG
UBC	NOD1
CASP9	NOD1
NOD1	CASP4
NOD1	CASP1
VIM	NOC4L
UBC	NOC4L
TSC22D4	NOC4L
NOC4L	NOP58
NECAB2	NOC4L
KRT15	NOC4L
DAZAP2	NOC4L
UBC	NOC3L
EIF2AK2	NOC3L
UBC	NOC2L
TP53	NOC2L
SUMO2	NOC2L
LRIF1	NOC2L

WDR18	NOA1
UBC	NOA1
NFU1	NOA1
COL9A2	NOA1
ABCF3	NOA1
ABCB8	NOA1
UBC	NNT
UBC	NNMT
NMU	NMUR2
NMU	NMUR1
UBC	NMRAL1
UBC	NMI
STAT6	NMI
STAT5B	NMI
NMI	STAT5A
STAT3	NMI
STAT1	NMI
NMI	SNAPC5
NMI	NECAP2
MYCN	NMI
MYC	NMI
LSM4	NMI
IFI35	NMI
NMI	HHLA3
GCSH	NMI
UBC	NME7
NME7	TNIP1
NME7	FKBP6
DYDC2	NME5
TRIP13	NME4
NME1	NME4
VIM	NME2
VCL	NME2
NME3	NME2
WDYHV1	NME1
UBC	NME1
TIAM1	NME1
NME1	PRUNE
NME3	NME1
NME2	NME1
ITGB1	NME1
UBC	NMD3
NMB	NMBR
SUGT1	NLRP4

PYCARD	NLRP3
UBC	NLRP11
NOD1	NLRP1
CASP9	NLRP1
CASP1	NLRP1
BCL2L1	NLRP1
APAF1	NLRP1
PYCARD	NLRC4
NOD2	NLRC4
NLRP1	NLRC4
CASP1	NLRC4
BCL10	NLRC4
UBC	NLK
MYB	NLK
NRXN1	NLGN1
UBC	NLE1
EWSR1	NLE1
TBX5	NKX2-5
SRF	NKX2-5
GATA4	NKX2-5
UBC	NKIRAS2
UBC	NKIRAS1
TGFBR1	NKIRAS1
NFKBIB	NKIRAS1
BMPR1B	NKIRAS1
ACVR1	NKIRAS1
UBC	NIT1
SETDB1	NIPSNAP3A
IMMT	NIPSNAP3A
IKBKAP	NIPSNAP3A
HAP1	NIPSNAP3A
GOLM1	NIPSNAP3A
EPHX2	NIPSNAP3A
UBC	NIPSNAP1
TBC1D17	NIPSNAP1
PRSS23	NIPBL
UBC	NIP7
NINL	ZNF250
NINL	SH3BP5L
NINL	RCOR3
NINL	KAT7
NINL	KANSL1
NINL	FAM107A
VIM	NIF3L1

UBC	NIF3L1
TSC22D4	NIF3L1
TRIP13	NIF3L1
TRAF2	NIF3L1
NIF3L1	TIFA
SNF8	NIF3L1
NIF3L1	S100A1
RPIA	NIF3L1
NIF3L1	PRTFDC1
NUDT21	NIF3L1
NIF3L1	MAPRE2
KRT15	NIF3L1
NIF3L1	GNMT
ENOX1	NIF3L1
NIF3L1	DMRTB1
NIF3L1	DCTD
CCDC85B	NIF3L1
SKIL	NID2
LAMC1	NID2
HSPG2	NID2
ELN	NID2
COL13A1	NID2
SKIL	NID1
PTPRF	NID1
NID1	MATN2
LGALS3BP	NID1
LAMC1	NID1
NID1	LACRT
ITGB3	NID1
FGB	NID1
FGA	NID1
NID1	FBLN2
FBLN1	NID1
COL4A1	NID1
COL1A1	NID1
NID1	COL13A1
XRCC6	NHP2L1
UBC	NHP2L1
RAD17	NHP2L1
PRPF4	NHP2L1
POLA2	NHP2L1
NOP56	NHP2L1
CDKN2C	NHP2L1
UBC	NHP2

SUMO2	NHP2
UBC	NHLRC2
UBC	NHLRC1
STUB1	NHLRC1
PPP1R3C	NHLRC1
LMO2	NHLH1
LMO1	NHLH1
CSRP3	NHLH1
UBC	NHEJ1
UBC	NGFR
TRAF6	NGFR
TRAF2	NGFR
NGFR	SORT1
NGFR	RTN4R
RIPK2	NGFR
PTPN13	NGFR
NGFR	NTRK1
NGFR	NTF4
NGFR	NGF
NGFR	KIDINS220
APP	NGFR
NGF	SORT1
PLG	NGF
NTRK1	NGF
A2M	NGF
ATP5C1	NGEF
UBC	NGDN
UBC	NFYC
TP73	NFYC
SUB1	NFYC
NFYB	NFYC
ATF6	NFYC
YBX1	NFYB
TP53	NFYB
MYC	NFYB
NFYB	HMGA1
HDAC4	NFYB
ELF1	NFYB
DRAP1	NFYB
NFYB	CNTN2
CIITA	NFYB
ZHX1	NFYA
TP53	NFYA
SP1	NFYA

NFYC	NFYA
NFYB	NFYA
JUN	NFYA
HMGA1	NFYA
BRCA1	NFYA
APPBP2	NFYA
UBC	NFXL1
UBC	NFS1
UCHL5	NFRKB
UBC	NFRKB
RUVBL2	NFRKB
RUVBL1	NFRKB
NFKB1	NFRKB
INO80E	NFRKB
INO80	NFRKB
STAT3	NFKBIZ
NFKB1	NFKBIZ
RELB	NFKBIE
RELA	NFKBIE
REL	NFKBIE
PPP6R2	NFKBIE
PPP6R1	NFKBIE
PPP6C	NFKBIE
NFKB2	NFKBIE
NFKB1	NFKBIE
JUP	NFKBIE
IL1A	NFKBIE
CUL1	NFKBIE
CHUK	NFKBIE
C1QBP	NFKBIE
ANKRD28	NFKBIE
ANKHD1-EIF4EBP3	NFKBIE
RXRA	NFKBIB
NFKBIB	POLR2H
POLR1A	NFKBIB
NFKBIB	PDCD2
NFKBIA	NFKBIB
NFKB2	NFKBIB
NFKB1	NFKBIB
NFKBIB	MCC
KPNA2	NFKBIB
IQGAP2	NFKBIB
CHUK	NFKBIB
VCP	NFKBIA

UBC	NFKBIA
SKP1	NFKBIA
RPS6KA1	NFKBIA
NFKB2	NFKBIA
NFKB1	NFKBIA
NCOR2	NFKBIA
NFKBIA	MCM7
NFKBIA	IKBKAP
DYNLL1	NFKBIA
COMMD1	NFKBIA
NFKBIA	CHUK
NFKBIA	CAPN1
NFKB2	TUBB6
TUBB	NFKB2
NFKB2	SFPQ
RUVBL2	NFKB2
NFKB2	PSMD1
NFKB2	KRT10
NFKB2	EFTUD2
NFKB2	DHX9
UBC	NFKB1
NFKB1	TUBB6
NFKB1	TRIP4
NFKB1	TNIP2
NFKB1	TNIP1
NFKB1	TIMM50
NFKB1	STAT6
SFPQ	NFKB1
RUVBL2	NFKB1
NFKB2	NFKB1
NCOR2	NFKB1
NFKB1	MYH9
MYH10	NFKB1
MEN1	NFKB1
NFKB1	MAP3K8
NFKB1	LYZ
KPNA1	NFKB1
NFKB1	IFNB1
NFKB1	ICAM1
NFKB1	HMGA2
NFKB1	HMGA1
GSK3B	NFKB1
NFKB1	ELF1
COPS5	NFKB1

NFKB1 CHUK
NFIL3 CREB3L3
BATF3 NFIL3
ATF1 NFIL3
ZKSCAN7 NFIC
UBC NFIC
NFIC TPD52L3
NFE2 NFE2L3
MAFK NFE2L3
MYC NFE2L2
PHYHIP NFE2
NFE2L2 NFE2
MAFK NFE2
ITCH NFE2
NFE2 CREB5
NFE2 CREB3L1
VIM NFATC2
NFATC2 MEF2D
NFATC2 MED31
NFATC2 EGR4
PRKCA NFATC1
XPO1 NF2
VPRBP NF2
UBC NF2
TSC1 NF2
TARBP2 NF2
SLC9A3R1 NF2
SDCBP NF2
PAK1 NF2
EZRNF2
CUL4A NF2
UBC NF1
IRF8 NF1
HDAC1 NF1
UBC NEXN
SMAD1 NEUROG1
UBC NEURL4
NEURL4 CEP97
NEURL4 CCP110
PLSCR1 NEU4
MDFI NEU4
UBC NEU3
GLB1 NEU1
GALNS NEU1

CTSA	NEU1
NET1	MAGI1
UBC	NES
UBC	NEO1
CASP3	NEO1
UBE3A	NELFCD
UBC	NELFCD
SUMO2	NELFCD
SMAD4	NELFCD
SERF2	NELFCD
NELFE	NELFCD
NELFB	NELFCD
NELFA	NELFCD
HDAC11	NELFCD
GSTM4	NELFCD
FRMD5	NELFCD
ENDOV	NELFCD
DNAJA1	NELFCD
ARPC4-TTLL3	NELFCD
UBC	NELFB
NELFE	NELFB
UBC	NELFA
NELFE	NELFA
NELFB	NELFA
UBC	NEK9
NEK6	NEK9
GABARAPL2	NEK9
UBC	NEK7
NEK9	NEK7
SGK1	NEK6
CCDC85B	NEK6
UBC	NEK2
PPP1R2	NEK2
PPP1CC	NEK2
PPP1CA	NEK2
NEK11	NEK2
NDC80	NEK2
UBC	NEFM
UBC	NEFL
TSC1	NEFL
UBC	NEDD9
SMAD1	NEDD9
NCK1	NEDD9
CRKL	NEDD9

CRK	NEDD9
BCAR1	NEDD9
ABL1	NEDD9
UBC	NEDD8-MDP1
VHL	NEDD8
NEDD8	UCHL3
NEDD8	UCHL1
UBC	NEDD8
TP53	NEDD8
TCEB2	NEDD8
TCEB1	NEDD8
SKP1	NEDD8
NEDD8	SENP8
RPS3	NEDD8
NEDD8	RPS23
NEDD8	RPS16
NEDD8	RPS14
NEDD8	RPL8
NEDD8	PSMD4
NEDD8	PARK2
NEDD8	NAE1
NEDD8	MCM4
NEDD8	KBTBD6
NEDD8	HIST2H2BE
EEF1G	NEDD8
EEF1A1	NEDD8
NEDD8	DCAF11
CUL2	NEDD8
CUL1	NEDD8
NEDD8	COPS7A
COPS6	NEDD8
NEDD8	COPS5
NEDD8	COPS4
NEDD8	COPS2
NEDD8	CDC34
UBE2L3	NEDD4L
UBC	NEDD4L
SMAD2	NEDD4L
NEDD4L	SCNN1A
NEDD4L	NDFIP2
UBE2L3	NEDD4
SMAD5	NEDD4
SMAD1	NEDD4
NEDD4	RAPGEF6

NEDD4	PTEN
NEDD4	NUDT21
NEDD4	MYZAP
NEDD4	ARID1A
UBC	NEDD1
TRAF2	NECAP2
NECAB2	RCOR3
NECAB2	NEK6
FXR2	NECAB2
DGCR6L	NECAB2
DFFA	NECAB2
NECAB2	CENPO
NECAB2	CCNK
ZYX	NEBL
PHYHIP	NDUFV3
SP110	NDUFV2
KAT5	NDUFV2
CRMP1	NDUFV2
UBC	NDUFS8
KCNH2	NDUFS6
UBC	NDUFS5
UBC	NDUFS2
NDUFS3	NDUFS2
MYC	NDUFS2
UBC	NDUFS1
MDH2	NDUFS1
CASP3	NDUFS1
UBC	NDUFC2
UBC	NDUFB9
SH3GL3	NDUFB9
IKBKAP	NDUFB9
HAP1	NDUFB9
FEZ1	NDUFB9
UBC	NDUFB8
PFN2	NDUFB8
UBC	NDUFB7
UBC	NDUFB6
UBC	NDUFB4
UBC	NDUFAF4
UBC	NDUFAF2
NDUFA7	STARD9
UBC	NDUFA5
NDUFS3	NDUFA5
NDUFB1	NDUFA5

DAZAP2	NDUFA5
CCDC85B	NDUFA5
UBC	NDUFA4
UBC	NDUFA11
UBC	NDUFA10
UBC	NDRG3
UBC	NDRG1
S100B	NDRG1
PHYHIP	NDRG1
UBC	NDN
TP53	NDN
NUCB2	NDN
NGFR	NDN
NDN	IL1A
NEDD4	NDFIP2
PTEN	NDFIP1
NEDD4	NDFIP1
PAFAH1B1	NDE1
NDC80	ZWINT
USHBP1	NDC80
UBC	NDC80
TOM1L1	NDC80
NDC80	SPC25
SPC24	NDC80
NDC80	SKA1
RB1	NDC80
NDC80	NUF2
NSL1	NDC80
MIS12	NDC80
MAD2L1	NDC80
KLHL12	NDC80
CCDC53	NDC80
NDC80	CALCOCO1
BLOC1S6	NDC80
NDC80	AMOTL2
UBC	NCSTN
PSENEN	NCSTN
PSEN1	NCSTN
APP	NCSTN
UBC	NCS1
PI4KB	NCS1
DTX2	NCS1
DRD5	NCS1
DRD2	NCS1

TYROBP	NCR2
ZMYND11	NCOR1
ZBTB7A	NCOR1
ZBTB6	NCOR1
ZBTB16	NCOR1
VDR	NCOR1
UBC	NCOR1
TXNRD2	NCOR1
TRIM28	NCOR1
THRB	NCOR1
THRA	NCOR1
TCF3	NCOR1
TBL1XR1	NCOR1
TAB2	NCOR1
SUMO2	NCOR1
SPEN	NCOR1
SOX2	NCOR1
SKIL	NCOR1
SKI	NCOR1
SIRT1	NCOR1
SIN3A	NCOR1
SAP30	NCOR1
SAFB	NCOR1
RXRA	NCOR1
RUNX1T1	NCOR1
RARA	NCOR1
PPARG	NCOR1
PPARA	NCOR1
POU1F1	NCOR1
PML	NCOR1
PHB	NCOR1
PDCD2	NCOR1
NR6A1	NCOR1
NR3C1	NCOR1
NR1H3	NCOR1
NR1H2	NCOR1
NR1D2	NCOR1
NR1D1	NCOR1
NELFE	NCOR1
NCOR2	NCOR1
KDM4A	NCOR1
HEY2	NCOR1
HESX1	NCOR1
HDAC9	NCOR1

HDAC7	NCOR1
HDAC4	NCOR1
HDAC3	NCOR1
GPS2	NCOR1
ETV6	NCOR1
ESR1	NCOR1
DACH1	NCOR1
CORO2A	NCOR1
CBFA2T3	NCOR1
BCL6	NCOR1
ESR2	NCOA7
ESR1	NCOA7
NCOA6	THRB
SMAD2	NCOA6
RXRA	NCOA6
NCOA6	RBM39
NCOA6	PPARG
NCOA6	PAXIP1
NCOA6	NR1H2
NCOA6	MED23
NCOA6	GHR
UBC	NCOA3
NCOA3	THRB
SMAD2	NCOA3
RXRA	NCOA3
NCOA3	PPARG
PGR	NCOA3
NCOR2	NCOA3
NCOA3	NCOA2
NCOA3	KAT2B
NCOA3	CHUK
NCOA2	THRB
NCOA1	TRIP4
NCOA1	THRB
NCOA1	TEAD2
NCOA1	STAT6
NCOA1	STAT3
NCOA1	RXRB
RXRA	NCOA1
NCOA1	RARA
NCOA1	PPARGC1A
NCOA1	PPARG
NCOA1	PPARA
PGR	NCOA1

NCOA1 NR1I3
NCOA1 NR1H3
NCOA1 NFKB1
NCOA2 NCOA1
NCOA1 KAT2B
NCOA1 ESRRG
NCOA1 COPS5
UBC NCLN
UBC NCL
TP53 NCL
TOP1 NCL
SSB NCL
NCL S100A11
PPARGC1A NCL
NCL NPM3
NPM1 NCL
NFKB2 NCL
MYC NCL
MDM2 NCL
NCL MAP3K14
LMNA NCL
BAIAP2 NCKIPSD
UBC NCKAP5L
NCK1 NCKAP5
UBC NCKAP1L
GAS7 NCKAP1L
YWHAB NCKAP1
NCKAP1 WASF2
WASF1 NCKAP1
UBC NCKAP1
NCK1 NCKAP1
GAS7 NCKAP1
CYFIP1 NCKAP1
BRK1 NCKAP1
ABI1 NCKAP1
WBP11 NCK2
NCK2 SOS1
SF3B4 NCK2
NCK2 RHOU
NCK2 PKN2
NCK2 PDGFRB
NCK2 PAK1
NCK2 NTRK2
NCK2 NCKIPSD

NCK2	LIMS1
NCK2	KIAA1217
ILK	NCK2
FXR2	NCK2
ERBB3	NCK2
EGFR	NCK2
NCK2	EFNB2
EEF1G	NCK2
NCK2	DOK1
NCK2	DOCK1
CPSF7	NCK2
CPSF6	NCK2
CBLB	NCK2
BCR	NCK2
ABI2	NCK2
NCK2	ABI1
WASL	NCK1
TNK2	NCK1
NCK1	TNIK
TBK1	NCK1
NCK1	SYNJ2
NCK1	SOS1
NCK1	RRAS
PXN	NCK1
NCK1	PKN2
NCK1	PDGFRB
NCK1	PAK2
NCK1	PAK1
NCK1	NTRK2
NCK1	NCKIPSD
LCP2	NCK1
KHDRBS1	NCK1
NCK1	KDR
NCK1	FASLG
ERBB3	NCK1
NCK1	EPHB2
NCK1	EPHB1
EGFR	NCK1
NCK1	DRD4
NCK1	DOK2
NCK1	DOK1
NCK1	DNM1
NCK1	CSNK1G2
CRK	NCK1

NCK1	BLNK
NCK1	BCAR1
NCK1	ASAP1
ARHGEF7	NCK1
ABL1	NCK1
NCK1	ABI1
NCF4	CYBA
NCF2	RAC2
NCF4	NCF2
CYBA	NCF2
CDC42	NCF2
UBC	NCDN
UBC	NCBP2
HNRNPH1	NCBP2
UPF1	NCBP1
UBC	NCBP1
NCBP2	NCBP1
HNRNPH1	NCBP1
HNRNPF	NCBP1
BNIP3	NCBP1
ALYREF	NCBP1
UBC	NCAPH2
UBC	NCAPH
SMC2	NCAPH
NCAPG	NCAPH
UBC	NCAPG2
SMC2	NCAPG2
NCAPH2	NCAPG2
NCAPD3	NCAPG2
UBC	NCAPD3
SMC2	NCAPD3
PRSS23	NCAPD3
NCAPH2	NCAPD3
UBC	NCAPD2
SMC4	NCAPD2
SMC2	NCAPD2
NCAPH	NCAPD2
NCAPG	NCAPD2
UBC	NCAM1
ST8SIA4	NCAM1
ST8SIA2	NCAM1
NCAN	NCAM1
UBC	NCALD
ACTB	NCALD

VTA1	NBR1
UBC	NBR1
SQSTM1	NBR1
GABARAPL2	NBR1
GABARAP	NBR1
TSEN15	NBPF3
UBC	NBPF11
WRN	NBN
MDM2	NBN
UBC	NBEAL2
VCL	NBEA
UBC	NBAS
OLFM2	NBAS
UBC	NAT9
SMAD1	NAT9
C14orf1	NAT9
UBC	NASP
HIST1H3A	NASP
NASP	HIST1H1A
NASP	H3F3A
NASP	ASF1A
UBC	NARS
PXN	NARS
LARS	NARS
AARS	NARS
UBC	NAPRT1
STX8	NAPG
NSF	NAPG
UBC	NAPEPLD
UBC	NAPB
VAMP8	NAPA
UBC	NAPA
STX8	NAPA
NAPA	STX7
STX5	NAPA
STX4	NAPA
STX1A	NAPA
NAPA	STX12
NAPA	NSF
PTN	NAP1L5
KAT5	NAP1L5
HAP1	NAP1L5
UBC	NAP1L4
NAP1L1	NAP1L4

UBC	NAP1L1
TRAF2	NAP1L1
TRAF1	NAP1L1
TNFRSF1A	NAP1L1
TANK	NAP1L1
PSMD4	NAP1L1
NAP1L1	NOP56
NAP1L1	IPO9
NAP1L1	FSD1
DLST	NAP1L1
UBC	NANS
UBC	NAGLU
UBC	NAF1
NOP10	NAF1
UBQLN4	NAE1
UBC	NAE1
APP	NAE1
UPF1	NADSYN1
UBC	NADSYN1
CUTC	NADSYN1
UBC	NADK2
YWHAG	NADK
NUDT18	NADK
LNX1	NADK
GLRX3	NADK
EEF1G	NADK
UBC	NACC1
UBC	NACAD
INTS3	NABP1
UBC	NAA50
UBC	NAA35
UBC	NAA25
UBC	NAA20
UBC	NAA16
UBC	NAA11
TRMT112	N6AMT1
SMURF2	N4BP3
NEDD4	N4BP3
UBC	N4BP2
SUMO1	MZF1
PIN1	MYT1
CDK1	MYT1
UBC	MYRF
NEB	MYPN

TCAP	MYOZ2
PPP3CB	MYOZ2
PPP3CA	MYOZ2
ACTN4	MYOZ2
TTN	MYOM2
TTN	MYOM1
TCF3	MYOG
SRF	MYOG
SP1	MYOG
MEF2A	MYOG
MDFI	MYOG
ELSPBP1	MYOG
CSRP3	MYOG
CALM1	MYOG
UBC	MYOF
UBC	MYOD1
MYOD1	TWIST1
TCF3	MYOD1
MYOD1	STAT3
SRF	MYOD1
SP1	MYOD1
SMAD4	MYOD1
RXRA	MYOD1
MYOD1	RORA
PSMD4	MYOD1
PRMT5	MYOD1
NCOR2	MYOD1
MYOD1	MOS
MYOD1	MEF2C
MEF2A	MYOD1
KAT2B	MYOD1
IFRD1	MYOD1
ID3	MYOD1
HSP90AA1	MYOD1
MYOD1	HEY1
HDAC1	MYOD1
MYOD1	HAND1
MYOD1	ELSPBP1
MYOD1	CSRP3
CREBBP	MYOD1
MYOD1	CDKN1C
CDK4	MYOD1
MYOD1	CDC34
CALM1	MYOD1

MYOD1 BHLHE41
CDC42 MYO9A
USH1C MYO7A
MYRIP MYO7A
UBC MYO6
TNFRSF1B MYO6
TNFRSF1A MYO6
RNF11 MYO6
RIPK3 MYO6
OPTN MYO6
MAP3K3 MYO6
LMTK2 MYO6
IKBKE MYO6
GIPC1 MYO6
DLG1 MYO6
DAB2 MYO6
CALM1 MYO6
UBC MYO5C
RAB11B MYO5B
RAB11A MYO5B
TGFBR1 MYO3A
UBC MYO1F
UBC MYO1E
UBC MYO1C
RIPK3 MYO1C
MAP3K3 MYO1C
ESR1 MYO1C
CBL MYO1C
ACTG1 MYO1A
USP13 MYO15A
EPS8 MYO15A
UBC MYO10
PLA2G10 MYO10
ITGB5 MYO10
ITGB3 MYO10
ITGB1 MYO10
DYNLL1 MYO10
CALM1 MYO10
SRCMYLK
PAK2 MYLK
PAK1 MYLK
MAPK3 MYLK
CAMK2G MYLK
CALM1 MYLK

UBC	MYLIP
UBC	MYL6B
MYO5A	MYL6B
VCL	MYL6
UBC	MYL6
PXN	MYL6
NUDT21	MYL6
MYH9	MYL6
MOB4	MYL6
UBC	MYL12B
UBC	MYL12A
TNFRSF1A	MYL12A
IKBKE	MYL12A
CHUK	MYL12A
UBC	MYL1
ACTA2	MYL1
ACTA1	MYL1
VCL	MYH9
TNFRSF1A	MYH9
ASCC2	MYH9
S100A4	MYH4
PRKCE	MYH2
UBC	MYH14
CHUK	MYH14
UBC	MYH11
UBC	MYH10
MYH10	TNFRSF1A
CSRP3	MYF6
ID3	MYF5
ELSPBP1	MYF5
CALM1	MYF5
COPS6	MYEOV2
COPS5	MYEOV2
MYCBP	MYCBPAP
UBC	MYCBP2
MYC	MYCBP2
UBC	MYCBP
COPS6	MYCBP
ARHGEF40	MYCBP
MYC	USP28
MYC	TRRAP
MYC	RUVBL2
MYC	PAK2
MYC	EFTUD2

EEF1G	MYC
TRIM63	MYBPC3
MYH9	MYBPC3
MYBPH	MYBPC2
MYBL2	SND1
MYBL2	RBL1
MYBL2	NCOR2
MYBL2	LIN9
MYBL2	LIN52
MYBL2	CDC34
MYBL2	CCNA2
UBC	MYBBP1A
POLR1A	MYBBP1A
NFKB2	MYBBP1A
MYC	MYBBP1A
MYBBP1A	MYB
MYBBP1A	MAP3K14
UBC	MXRA7
UBC	MXI1
MAX	MXI1
UBC	MXD4
MAX	MXD4
UBC	MXD3
NFKB1	MXD3
MAX	MXD3
SIN3B	MXD1
SIN3A	MXD1
MYCN	MXD1
MAX	MXD1
HDAC2	MXD1
HDAC1	MXD1
CD2	MXD1
BLM	MX1
UBC	MVP
PXN	MVP
PTEN	MVP
PARP4	MVP
ESR1	MVP
UBC	MVD
HSPA9	MVD
GNB5	MVD
EIF4EBP1	MVD
EEF1G	MVD
BTAF1	MVD

UBC	MVB12B
UBC	MVB12A
UBC	MUTYH
PCNA	MUTYH
LRSAM1	MUTYH
APEX1	MUTYH
MMAA	MUT
SLX4	MUS81
FEN1	MUS81
EME1	MUS81
CHEK2	MUS81
UBE2D3	MUL1
UBC	MUL1
PRKCD	MUC1
ERBB4	MUC1
UBC	MTX2
HSP90B1	MTTP
APOB	MTTP
UBC	MTR
DYNLL1	MTR
UBC	MTPN
UBC	MTPAP
YWHAQ	MTOR
UBQLN1	MTOR
UBC	MTOR
TRAF2	MTOR
TELO2	MTOR
MTOR	STAT3
STAT1	MTOR
RPTOR	MTOR
MTOR	RPS6KB1
MTOR	RICTOR
RHEB	MTOR
MTOR	PRR5
PRKCD	MTOR
MLST8	MTOR
MTOR	MAPKAP1
IRS1	MTOR
MTOR	GPHN
MTOR	FKBP1A
MTOR	EIF4EBP1
MTOR	EIF3B
MTOR	DEPTOR
CHUK	MTOR

MTOR	ATG13
AKT1S1	MTOR
ABL1	MTOR
UBC	MTO1
MTNR1B	MTNR1A
GNAI3	MTNR1A
GNAI2	MTNR1A
GNA13	MTNR1A
GNA11	MTNR1A
MTMR9	MTMR6
UBC	MTMR12
SMAD9	MTMR11
SMAD9	MTMR10
SMAD5	MTMR10
NFKBIB	MTIF2
UBC	MTHFD1
UBC	MTFR2
UBC	MTFR1L
PIN1	MTFR1
PRKD2	MTF2
AP1M1	MTF1
UBC	MTERFD2
UBC	MTCH2
UBC	MTCH1
UBC	MTBP
MDM2	MTBP
UBC	MTAP
MYLIP	MTAP
UBC	MTA3
KDM1A	MTA3
HDAC2	MTA3
HDAC1	MTA3
CHD4	MTA3
RBBP4	MTA2
SPINK7	MT2A
PRKD1	MT2A
SPINK7	MT1H
SPINK7	MT1G
SUMO1	MSX1
RGS7	MSX1
MSX2	MSX1
MSX1	LHX2
ACVR2B	MSTN
UBC	MSMO1

MSL1	MSL2
UBC	MSI1
PNMA1	MSI1
XRCC6	MSH6
UBC	MSH6
SUMO2	MSH6
MYC	MSH6
MLH1	MSH6
BRCA1	MSH6
BLM	MSH6
MSH4	MSH5
ABL1	MSH5
UBC	MSH3
RPA1	MSH3
MSH6	MSH3
MLH1	MSH3
EXO1	MSH3
BRCA1	MSH3
XRCC6	MSH2
UBC	MSH2
TP53	MSH2
SUMO2	MSH2
SLX4	MSH2
PMS2	MSH2
PCNA	MSH2
MYC	MSH2
MSH6	MSH2
MSH3	MSH2
MLH1	MSH2
MAX	MSH2
EXO1	MSH2
ESR1	MSH2
CHEK2	MSH2
BRCA1	MSH2
BLM	MSH2
BARD1	MSH2
ATRMSH2	
SYK	MS4A2
LYN	MS4A2
PRKG1	MRVI1
UBC	MRTO4
RSL24D1	MRTO4
UBC	MRPS9
RBM4B	MRPS9

MRPS2	MRPS9
MRPL3	MRPS9
MPHOSPH6	MRPS9
HAP1	MRPS9
CCNDBP1	MRPS9
UBC	MRPS7
UBC	MRPS5
MRPS10	MRPS5
UBC	MRPS36
UBC	MRPS35
UBC	MRPS34
UBC	MRPS31
UBC	MRPS27
UBC	MRPS22
UBC	MRPS2
UBC	MRPS18C
UBC	MRPS17
C14orf1	MRPS12
UBC	MRPL50
UBC	MRPL48
UBC	MRPL45
VIM	MRPL44
UBC	MRPL44
SETDB1	MRPL44
IMMT	MRPL44
IKBKAP	MRPL42
UBC	MRPL4
EIF2B1	MRPL4
DDX24	MRPL4
UBC	MRPL38
PRIM1	MRPL38
HSPB3	MRPL38
C14orf1	MRPL38
UBC	MRPL37
MRPL12	MRPL37
UBC	MRPL3
UBC	MRPL27
UBC	MRPL22
USP13	MRPL20
UBC	MRPL20
TP53BP2	MRPL20
ITSN1	MRPL20
EPS8	MRPL20
CALM1	MRPL20

ACP1	MRPL20
UBC	MRPL2
RPS15	MRPL2
MRPL2	MRPL13
UBC	MRPL18
LYST	MRPL17
UBC	MRPL13
MRPL3	MRPL13
MRPL15	MRPL13
MRPL12	TMEM51
RPS2	MRPL12
MRPL3	MRPL12
MRPL15	MRPL12
USP22	MRPL10
UBC	MRPL1
CORT	MRGPRX2
RUVBL2	MRGBP
RUVBL1	MRGBP
MORF4L1	MRGBP
MEAF6	MRGBP
KAT5	MRGBP
DMAP1	MRGBP
BRD8	MRGBP
ACTL6A	MRGBP
ACTB	MRGBP
UBC	MRFAP1L1
UBC	MRFAP1
MORF4L1	MRFAP1
UBC	MREG
NEK1	MRE11A
NBN	MRE11A
MRE11A	ATM
UBC	MRC2
RAF1	MRAS
UBC	MPZL1
UBC	MPST
YWHAQ	MPRIP
YWHAG	MPRIP
YWHAB	MPRIP
UBC	MPRIP
RIPK3	MPRIP
PPP1R12C	MPRIP
PPP1R12A	MPRIP
MAP3K3	MPRIP

UBC MPP7
UBC MPP6
SKIV2L2 MPP6
LIN7A MPP6
LIN7A MPP5
CRB3 MPP5
TRIM41 MPP3
SF3B3 MPP3
RPS2 MPP3
RAB31 MPP3
PLK2 MPP3
NOA1 MPP3
MDH1 MPP3
MCM2 MPP3
KBTBD4 MPP3
INSM1 MPP3
HAP1 MPP3
GNG7 MPP3
CRABP1MPP3
CP MPO
THPO MPL
JAK2 MPL
ATXN2LMPL
UBC MPI
UBC MPHOSPH10
SUMO2 MPHOSPH10
PPP1CA MPHOSPH10
IMP4 MPHOSPH10
UBC MPDZ
PLEKHA1 MPDZ
UBC MPC2
RPLP1 MPC2
UBC MOV10
EIF2AK2 MOV10
CBX8 MOV10
UBC MORF4L1
TRRAP MORF4L1
SIN3B MORF4L1
RUVBL2 MORF4L1
RUVBL1 MORF4L1
MORF4L1 PALB2
MRFAP1L1 MORF4L1
KAT5 MORF4L1
HIST2H2BE MORF4L1

HDAC2 MORF4L1
MORF4L1 EPC2
EP400 MORF4L1
BRD8 MORF4L1
ACTL6A MORF4L1
UBC MORC3
UBC MON2
TNFRSF1B MON2
TNFRSF1A MON2
JUN MOK
CCNB1 MOK
UBC MOGS
CHGB MOGS
XRN2 MOCS3
URM1 MOCS3
UBC MOCS3
UBC MOCS2
RPL9 MOCS2
MOCS3 MOCS2
ABCF3 MOCS2
UBC MOCOS
MOB4 UBE2V2
STK24 MOB4
SCMH1 MOB4
MOB4 PLEKHM1
PAICS MOB4
MYO9B MOB4
FADD MOB4
MOB4 CTTNBP2
CCDC85B MOB4
MOB4 ARHGEF15
UBC MOB1B
STK38 MOB1B
LATS2 MOB1B
UBC MOB1A
MOB1A LATS2
CCDC85B MOB1A
UBC MOAP1
DPPA2 MOAP1
CCDC85B MOAP1
BCL2L1 MOAP1
BAX MOAP1
MAX MNT
UBC MND1

UBC	MNAT1
RBM48	MNAT1
RB1	MNAT1
POU5F1	MNAT1
POU2F1	MNAT1
MNAT1	HPS6
GTF2H3	MNAT1
GTF2H1	MNAT1
ESR1	MNAT1
ERCC3	MNAT1
MNAT1	ERCC2
COPS6	MNAT1
CDK7	MNAT1
CCNH	MNAT1
BRCA1	MNAT1
UBC	MMS19
MLF2	MMS19
ILK	MMS19
CIAO1	MMS19
FN1	MMP9
COL4A1	MMP9
COL1A1	MMP9
TIMP2	MMP8
CCL2	MMP8
ACAN	MMP8
PLG	MMP7
NGF	MMP7
HBEGF	MMP7
BCAN	MMP7
TIMP3	MMP3
CCL7	MMP3
CCL2	MMP3
BCAN	MMP3
MMP2	MMP25
CLU	MMP25
MMP2	TIMP4
MMP2	TIMP3
MMP2	TIMP2
THBS2	MMP2
MMP2	LCN2
MMP2	BACE1
MMP2	MMP17
LRP1	MMP17
UBC	MMP15

TIMP3	MMP14
TIMP2	MMP14
SDC1	MMP14
MMP2	MMP14
CLDN1	MMP14
C1QBP	MMP14
ACAN	MMP14
TIMP3	MMP13
CCL7	MMP13
BCAN	MMP13
UBC	MLX
MXD4	MLX
MXD1	MLX
MNT	MLX
MLXIPL	MLX
MLXIP	MLX
GABARAPL2	MLX
RPTOR	MLST8
MYO5A	MLPH
GHRL	MLNR
YWHAQ	MLLT4
YWHAG	MLLT4
YWHAB	MLLT4
MLLT4	TJP1
TERF2IP	MLLT4
MLLT4	SORBS1
SMAD2	MLLT4
RRAS2	MLLT4
MLLT4	RIT1
MLLT4	RAP1A
MLLT4	PVRL3
MLLT4	PVRL1
PFN1	MLLT4
MLLT4	MRAS
MLLT4	LMO2
MLLT4	HRAS
MLLT4	EPHB6
MLLT4	EPHB3
MLLT4	EPHB2
MLLT4	EPHA7
MLLT4	CTNNA1
RBM48	MLLT3
PTN	MLLT3
MLLT1	MLLT3

MCM6	MLLT3
ELL2	MLLT3
ELL	MLLT3
EAF1	MLLT3
CDK9	MLLT3
CCNT2	MLLT3
CCNT1	MLLT3
CBX8	MLLT3
APPBP2	MLLT3
AFF4	MLLT3
AFF3	MLLT3
AFF1	MLLT3
UBC	MLLT11
MLLT1	MLLT10
DOT1L	MLLT10
MLLT1	CBX8
MLLT1	AFF1
PBX2	MLKL
MLH1	MLH3
ZC3H11A	MLH1
UBOX5	MLH1
UBC	MLH1
TRIM29	MLH1
MLH1	PMS1
MYC	MLH1
MSH4	MLH1
MLH1	MBD4
MLH1	FAN1
EXO1	MLH1
BRIP1	MLH1
BRCA1	MLH1
BLM	MLH1
UBC	MLF2
UBC	MKRN2
UBC	MKRN1
MAPK1	MKNK2
ESR2	MKNK2
ESR1	MKNK2
PLA2G4A	MKNK1
MAPK14	MKNK1
MAPK1	MKNK1
EIF4G3	MKNK1
EIF4G2	MKNK1
EIF4G1	MKNK1

UBC	MKLN1
RANBP9	MKLN1
SRF	MKL2
SMAD1	MKL2
UBC	MKL1
UBC	MKI67
UBC	MIS18BP1
MIS12	SUGT1
SPC24	MIS12
MIS12	NUF2
MIS12	DSN1
MIS12	BUB1B
MIS12	BUB1
UBC	MINK1
UBC	MINA
MIF	VCL
UBC	MIF
MIF	COPS5
MIF	CD74
MIF	BNIPL
UBC	MIER1
UBC	MICU2
UBC	MICU1
UBC	MICB
UBC	MICAL2
UBE2N	MIB1
UBC	MIB1
DAPK1	MIB1
DAB2	MIB1
UBC	MGST3
F10	MGST3
UBC	MGRN1
TSG101	MGRN1
MGRN1	MC1R
UBC	MGMT
CHGB	MGLL
UBC	MGEA5
HAP1	MGEA5
CDC37	MGEA5
SMAD9	MGAT1
UBC	MGA
SMAD1	MGA
MAX	MGA
UBC	MFSD10

NOTCH2	MFNG
MFNG	JAG1
MFNG	DLK1
MARCH5	MFN2
UBC	MFN1
PARK2	MFN1
MAVS	MFN1
MARCH5	MFN1
UBC	MFGE8
UBC	MFF
FBN2	MFAP5
UBC	MFAP3L
UBC	MFAP3
ELN	MFAP2
UBC	MFAP1
UBE2D3	MEX3B
UBC	MEX3B
SMAD4	MEX3B
UBC	METTL9
UBC	METTL6
UBC	METTL5
UBC	METTL2A
PHYHIP	METTL18
UBC	METTL14
METTL3	METTL14
WDR4	METTL1
UBC	METTL1
UBC	METAP2
UBC	METAP1
SMAD9	METAP1
UBC	MET
STAT3	MET
MET	PLXNB1
PLCG1	MET
PIK3R1	MET
MET	HGF
GRB2	MET
MET	GAB1
EGFR	MET
MET	DNAJA3
CTNNB1	MET
CBL	MET
LMO4	MERTK
UBC	MEPCE

PRPF4	MEPCE
MEPCE	CAND1
PYY	MEP1B
MEP1A	MEP1B
FN1	MEP1B
FN1	MEP1A
MEOX2	TULP2
MEOX2	RND2
NFKB2	MEN1
UBC	MEMO1
UBC	MELK
PBX2	MEIS1
MEIS1	HOXD13
MEIS1	HOXD12
MEIS1	HOXB13
MEIS1	HOXB1
HOXA9	MEIS1
MEIS1	HOXA7
MEIS1	HOXA13
MEIS1	HOXA11
MEIS1	HOXA10
CREB1	MEIS1
RANBP10	MEGF10
AP2M1	MEGF10
PYCARD	MEFV
CASP1	MEFV
UBC	MEF2D
MAPK7	MEF2D
HDAC4	MEF2D
CABIN1	MEF2D
UBC	MEF2C
MEF2C	TWIST2
SP1	MEF2C
MEF2D	MEF2C
MAPK7	MEF2C
MAPK14	MEF2C
HDAC9	MEF2C
HDAC7	MEF2C
HDAC4	MEF2C
CABIN1	MEF2B
UBC	MEF2A
MEF2A	THRA
SUMO2	MEF2A
SUMO1	MEF2A

MEF2A	MEF2D
MAPK7	MEF2A
MAPK14	MEF2A
MEF2A	HDAC9
HDAC7	MEF2A
HDAC4	MEF2A
MEF2A	ASCL1
MED8	MED9
MED7	MED9
MED6	MED9
MED4	MED9
MED31	MED9
MED30	MED9
MED28	MED9
MED26	MED9
MED25	MED9
MED24	MED9
MED22	MED9
MED18	MED9
MED1	MED9
CDK8	MED9
TCEB2	MED8
MED1	MED8
SREBF1	MED7
CDK8	MED7
UBC	MED6
MED6	SREBF1
SMAD2	MED6
SMAD1	MED6
MED6	MED7
MED6	MED30
MED6	MED28
MED6	MED26
MED6	MED25
MED6	MED21
MED6	MED10
MED1	MED6
MED6	CDK8
MED1	MED4
MCRS1	MED4
MED4	AP2M1
MED31	ZBTB45
TINAGL1	MED31
SERPINB9	MED31

MED31	SELENBP1
RFC5	MED31
PSMD11	MED31
PRMT1	MED31
MOB4	MED31
MED28	MED31
MED31	MED26
MED31	MED18
MED31	MED10
LAMA4	MED31
HSPB3	MED31
MED31	HNRNPUL1
GSTM4	MED31
ERH	MED31
MED31	EPN1
MED31	EGR2
EEF1G	MED31
CPE	MED31
MED31	CDK8
CCT7	MED31
MED31	CACNB4
ASCC2	MED31
MED31	AGR2
AES	MED31
MED1	MED30
UBC	MED29
POLR2J	MED29
MED9	MED29
MED8	MED29
MED6	MED29
MED4	MED29
MED31	MED29
MED28	MED29
MED26	MED29
MED25	MED29
MED24	MED29
MED21	MED29
MED20	MED29
MED18	MED29
MED17	MED29
MED10	MED29
MED1	MED29
MED28	MED8
MED28	MED4

MED28	MED30
MED24	MED28
MED23	MED28
MED28	MED22
MED1	MED28
LCK	MED28
KLHL12	MED28
FYN	MED28
MED28	CCNC
UBC	MED27
MED28	MED27
MED26	MED27
MED10	MED27
MED1	MED27
SREBF1	MED26
POLR2J	MED26
POLR2H	MED26
MED26	POLR2F
POLR2E	MED26
MED8	MED26
MED26	MED7
MED4	MED26
MED30	MED26
MED28	MED26
MED23	MED26
MED1	MED26
CDK8	MED26
UBC	MED25
SREBF1	MED25
MED28	MED25
MED26	MED25
MED10	MED25
MED1	MED25
CDK8	MED25
UBC	MED24
MED24	SREBF1
SMAD2	MED24
SMAD1	MED24
RXRA	MED24
MED1	MED24
MED24	CDK8
UBC	MED23
MED23	SREBF1
MED1	MED23

MED23	CDK8
MED1	MED22
UBC	MED21
THRB	MED21
MED30	MED21
MED28	MED21
MED21	MED26
MED21	MED18
MED21	MED10
MED1	MED21
MED21	CDK8
MED28	MED20
MED26	MED20
MED10	MED20
MED1	MED20
CDK8	MED20
UBC	MED18
MED8	MED18
MED4	MED18
MED28	MED18
MED26	MED18
MED24	MED18
MED18	MED20
MED10	MED18
MED1	MED18
UBC	MED17
TP53	MED17
SREBF1	MED17
MED30	MED17
MED28	MED17
MED17	MED26
MED17	MED25
MED17	MED18
MED17	MED10
MED1	MED17
CDK8	MED17
UBC	MED15
SREBF1	MED15
SMAD4	MED15
SMAD3	MED15
SMAD2	MED15
SMAD1	MED15
PLSCR1	MED15
MLLT6	MED15

MED6	MED15
MED28	MED15
MED26	MED15
MED25	MED15
MED10	MED15
MED1	MED15
FHL3	MED15
CDK8	MED15
ATP5C1	MED15
MED26	MED13L
UBC	MED13
MED13	SREBF1
MED13	MED9
MED13	MED4
MED13	MED29
MED13	MED28
MED13	MED26
MED23	MED13
MED13	MED18
MED13	MED10
MED1	MED13
MED13	CDK8
MED9	MED11
MED8	MED11
MED29	MED11
MED28	MED11
MED26	MED11
MED22	MED11
MED10	MED11
MED1	MED11
UBC	MED10
MOB4	MED10
MED8	MED10
MED10	MED7
MED4	MED10
MED30	MED10
MED28	MED10
MED26	MED10
MED24	MED10
MED23	MED10
MED22	MED10
MED1	MED10
CDK8	MED10
MED10	CCNC

KAT2B	MECOM
HDAC4	MECOM
CTBP1	MECOM
PLEKHF2	MEAF6
L3MBTL2	MEAF6
ING5	MEAF6
FXR2	MEAF6
CCDC85B	MEAF6
UBC	ME1
YWHAQ	MDM4
YWHAG	MDM4
YWHAE	MDM4
YWHAB	MDM4
UBC	MDM4
MDM4	TP73
TP53	MDM4
SUMO1	MDM4
SMAD3	MDM4
MDM4	RB1
MDM2	MDM4
MDM4	CSNK1A1
MDM4	ATM
MDM2	PSMC3
MDM2	GNL3
UBC	MDH2
MDH2	PC
MDH2	GOT2
MDH2	FH
UBC	MDH1
MDFI	ZNF764
MDFI	ZNF679
MDFI	ZNF607
MDFI	ZNF581
MDFI	ZNF559
MDFI	ZNF440
MDFI	ZNF439
MDFI	ZNF101
MDFI	WDYHV1
MDFI	VPS72
MDFI	TRAF3IP2
MDFI	TINAGL1
MDFI	THEG
MDFI	TAP1
MDFI	SPG7

MDFI	SIX1
MDFI	SFI1
MDFI	SERF2
MDFI	RFX2
MDFI	RABL6
MDFI	PTPMT1
MDFI	PIN1
MDFI	PHLDA1
MDFI	NR1H2
MDFI	METTL17
MDFI	LRRN4
MDFI	LRCH4
MDFI	LMO3
LASP1	MDFI
MDFI	IQUB
MDFI	HOXB9
MDFI	HOXA1
MDFI	GPRIN2
MDFI	GNAI2
MDFI	FZD9
MDFI	FAM124B
MDFI	EBI3
MDFI	DUSP6
MDFI	DNPEP
MDFI	DKK1
MDFI	CRY1
MDFI	CRX
MDFI	CPSF3L
MDFI	CNNM3
MDFI	CHIC2
MDFI	CCER1
MDFI	CCDC116
MDFI	CBX2
MDFI	CBFA2T2
MDFI	CATSPER1
MDFI	C8orf48
MDFI	C6orf165
MDFI	C10orf62
MDFI	ATG12
MDFI	AQP1
MDFI	AASDHPPT
MDC1	NBN
MDC1	MRE11A
MDM2	MDC1

MDC1	CDC16
MDC1	ATM
ANAPC5	MDC1
UBC	MCU
MICU1	MCU
RALYL	MCRS1
RAD51	MCPH1
TRIM27	MCOLN1
UBC	MCMBP
MCM5	MCMBP
DDIT3	MCMBP
UBC	MCM9
UBC	MCM7
ORC4	MCM7
MYC	MCM7
MCM6	MCM7
MCM4	MCM7
UBC	MCM6
MMS22L	MCM6
MCM4	MCM6
ING5	MCM6
IL36RN	MCM6
ASF1B	MCM6
ALS2CR11	MCM6
UBC	MCM5
STAT1	MCM5
ORC5	MCM5
NFKBIA	MCM5
NEDD8	MCM5
MDFI	MCM5
MCM7	MCM5
MCM6	MCM5
MCM4	MCM5
MCM3	MCM5
MCM2	MCM5
UBC	MCM3AP
TMEM216	MCM3AP
SMAD9	MCM3AP
SMAD3	MCM3AP
NUDT5	MCM3AP
MCM3	MCM3AP
ENY2	MCM3AP
CRMP1	MCM3AP
CLSTN1	MCM3AP

UBC	MCM3
STAT1	MCM3
ORC5	MCM3
ORC4	MCM3
MCM7	MCM3
MCM6	MCM3
MCM4	MCM3
MCM2	MCM3
MCM3	CDC7
CDC6	MCM3
MCM3	CDC45
UBC	MCM2
MCM2	RPA3
RPA1	MCM2
ORC5	MCM2
MCM2	MOB4
MCM2	MCM7
MCM6	MCM2
MCM4	MCM2
HIST3H3	MCM2
MCM2	GNB5
MCM2	CDK6
ZBTB43	MCM10
UBC	MCM10
TRIM37	MCM10
ORC4	MCM10
ORC3	MCM10
ORC2	MCM10
NINL	MCM10
MCM7	MCM10
MCM6	MCM10
MCM3	MCM10
MCM2	MCM10
KRT15	MCM10
DPPA2	MCM10
CEP72	MCM10
CEP70	MCM10
CDK6	MCM10
CDC7	MCM10
CDC6	MCM10
CDC5L	MCM10
CDC45	MCM10
CCND3	MCM10
CCDC85B	MCM10

CALCOCO2 MCM10
UBC MCL1
MCL1 TPT1
MCL1 TNKS
PMAIP1 MCL1
MCL1 HRK
CASP3 MCL1
MCL1 BOK
MCL1 BMF
MCL1 BIK
BID MCL1
BCL2L11 MCL1
BAX MCL1
BAK1 MCL1
BAD MCL1
PMCH MCHR2
PMCH MCHR1
LMAN1 MCFD2
CDC42 MCF2L
UBC MCCC2
CSNK1E MCCC1
MC1R MC4R
MC4R ASIP
ASIP MC3R
MC2R MRAP
MC2R ASIP
UBC MBTPS1
ATF6B MBTPS1
PRKCA MBP
UBC MBOAT7
UBC MBNL1
PTPRC MBL2
MASP2 MBL2
MAD2L1 MBL2
KRT1 MBL2
CALR MBL2
WDR5 MBIP
VTA1 MBIP
UBC MBIP
SUMO2 MBIP
RAP1GDS1 MBIP
PPFIA1 MBIP
PLEKHF2 MBIP
PET117 MBIP

NINL	MBIP
NDEL1	MBIP
MCM7	MBIP
LMO3	MBIP
FXR2	MBIP
EPB41L3	MBIP
DGCR6L	MBIP
COPS4	MBIP
CEP63	MBIP
CCDC85B	MBIP
UBC	MBD4
TRIM27	MBD4
FADD	MBD4
RBBP4	MBD2
PRMT5	MBD2
MTA2	MBD2
HDAC2	MBD2
HDAC1	MBD2
GPN1	MBD2
GATAD2B	MBD2
GATAD2A	MBD2
CREBBP	MBD2
CHD3	MBD2
UBC	MBD1
CHAF1A	MBD1
UBC	MB21D1
TRRAP	MAX
PLEKHF2	MAX
PCGF6	MAX
MYCN	MAX
MYC	MAX
EP400	MAX
UBC	MAVS
TRAF6	MAVS
TRAF2	MAVS
MAVS	TMEM173
TBK1	MAVS
MFN2	MAVS
IRF3	MAVS
IKBKE	MAVS
IFIH1	MAVS
FADD	MAVS
DDX58	MAVS
CHUK	MAVS

UBC	MATR3
TTF2	MATR3
SUMO2	MATR3
NFKB2	MATR3
NFKB1	MATR3
MATN1	MATN3
MATN2	MATN4
MATN2	MATN1
FN1	MATN2
MATN2	FBN2
COL4A2	MATN2
COL4A1	MATN2
COL1A1	MATN2
MATN1	MATN4
UBC	MATK
PXN	MATK
PTK2B	MATK
LYN	MATK
KIT	MATK
CD36	MATK
UBC	MAT2B
UBC	MAT2A
MAT2B	MAT2A
UBC	MASTL
SMAD1	MAST4
MASP2	FCN2
SERPING1	MASP1
UBC	MARVELD2
UBC	MARS
RARS	MARS
PPM1F	MARS
NFKB2	MARS
MAPK14	MARS
MAFK	MARS
LARS	MARS
IARS	MARS
HSP90AA1	MARS
MARS	HNRNPUL1
YWHAQ	MARK3
YWHAG	MARK3
YWHAE	MARK3
YWHAB	MARK3
VPS11	MARK3
USP21	MARK3

UBC	MARK3
TTR	MARK3
TCEA2	MARK3
SFN	MARK3
SAAL1	MARK3
PRKCQ	MARK3
PNPLA2	MARK3
PMPCB	MARK3
PKP2	MARK3
NOL11	MARK3
KSR1	MARK3
FANCF	MARK3
DYNLL1	MARK3
CPSF4	MARK3
CHGB	MARK3
CDC25C	MARK3
ASB13	MARK3
ANKRD7	MARK3
YWHAQ	MARK2
YWHAH	MARK2
YWHAG	MARK2
YWHAE	MARK2
YWHAB	MARK2
UTRN	MARK2
USP21	MARK2
UBC	MARK2
STK11	MARK2
PRKCI	MARK2
MAP3K3	MARK2
KIF13B	MARK2
ARHGEF2	MARK2
MAPT	MARK1
SCGB3A2	MARCO
SCGB3A1	MARCO
UBC	MARCKS
TOB1	MARCKS
PRKCA	MARCKS
UBC	MARCH7
UBC	MARCH6
UBC	MARCH1
UBC	MARC2
UBC	MARC1
UBC	MAPRE3
UBC	MAPRE2

MAPRE3	MAPRE2
MAPRE2	LMO2
APC	MAPRE2
UBC	MAPRE1
TUBB	MAPRE1
PIK3R4	MAPRE1
APP	MAPRE1
APC	MAPRE1
PLA2G4A	MAPKAPK5
MAPK9	MAPKAPK5
MAPK6	MAPKAPK5
MAPK13	MAPKAPK5
UBC	MAPKAPK3
TCF3	MAPKAPK3
MAPK14	MAPKAPK3
MAPK13	MAPKAPK3
UBC	MAPK9
TP53	MAPK9
MAPK9	MAPK8IP1
MAP2K7	MAPK9
MAPK9	MAP2K4
MAPK9	JDP2
MAPK9	DUSP10
MAPK9	DUSP1
RIC8A	MAPK8IP3
RAF1	MAPK8IP3
MAPK9	MAPK8IP3
MAPK8IP1	MAPK8IP3
MAPK10	MAPK8IP3
MAP3K5	MAPK8IP3
MAP2K7	MAPK8IP3
MAP2K4	MAPK8IP3
MAP2K1	MAPK8IP3
PRSS23	MAPK8IP2
MAPK8IP1	MAPK8IP2
MAPK13	MAPK8IP2
MAP3K11	MAPK8IP2
MAP2K7	MAPK8IP2
MAP2K3	MAPK8IP2
EGFR	MAPK8IP2
TIAM1	MAPK8IP1
MAP2K7	MAPK8IP1
EGFR	MAPK8IP1
APP	MAPK8IP1

UBC	MAPK8
TP53	MAPK8
MAPK8	STAT3
MAPK8	SPIB
SMAD3	MAPK8
MAPK8	SH3BP5
MAPK8	RPS6KB1
MAPK8	REL
PRKDC	MAPK8
MAPK8	PAX2
MAPK8	NR4A1
MAPK8	NR3C1
MAPK8	MAPK8IP3
MAPK8	MAPK8IP2
MAPK8	MAPK8IP1
MAP3K7	MAPK8
MAPK8	MAP3K1
MAP2K7	MAPK8
MAPK8	MAP2K4
MAPK8	KRT8
MAPK8	JUND
MAPK8	JUNB
MAPK8	JUN
MAPK8	JDP2
IRS1	MAPK8
MAPK8	IL27RA
MAPK8	IKBKAP
MAPK8	HRAS
H2AFX	MAPK8
MAPK8	GSTP1
MAPK8	DUSP16
MAPK8	DUSP10
MAPK8	DUSP1
CRK	MAPK8
MAPK8	BCL2L11
MAPK8	BCL2
MAPK8	APP
MYC	MAPK7
UBC	MAPK3
TP53	MAPK3
STMN1	MAPK3
STAT3	MAPK3
SREBF2	MAPK3
SPIB	MAPK3

SP1	MAPK3
SMAD2	MAPK3
RPS6KA4	MAPK3
RPS6KA1	MAPK3
PTPRE	MAPK3
MAPK3	PTPN7
MAPK3	PTPN5
PRKCE	MAPK3
MAPK3	PEA15
MAPK3	MBP
MAPKAPK2	MAPK3
MAPK14	MAPK3
MAP2K3	MAPK3
MAPK3	MAP2K2
MAP2K1	MAPK3
LCK	MAPK3
HDAC4	MAPK3
GTF2I	MAPK3
MAPK3	GAB2
ETS1	MAPK3
MAPK3	ELK4
MAPK3	DUSP5
MAPK3	DUSP4
MAPK3	DUSP3
MAPK3	DUSP1
MAPK3	CCDC6
CASP9	MAPK3
C1QBP	MAPK3
MAPK3	ADRBK1
MAPK14	ZFP36L1
UBC	MAPK14
MAPK14	TP53
MAPK14	RPS6KA4
MAPK14	RPL22
MAPK14	PTPN7
MAPK14	NCOA3
MAPK14	MBP
MAPK14	MAPKAPK5
MAPK14	MAPKAPK2
MAPK14	MAPK1
MAPK14	MAP2K4
MAPK14	MAP2K3
MAPK14	MAP2K1
MAPK14	JDP2

MAPK14	HTRA2
MAPK14	FKBP8
MAPK14	ELK4
MAPK14	DYRK1B
MAPK14	DUSP4
MAPK14	DUSP10
MAPK14	DUSP1
MAPK14	COPS5
MAPK13	MBP
MAPT	MAPK12
MAPK12	MAPKAPK5
MAPK12	DUSP1
MAPK10	MAP2K4
ZFP36	MAPK1
VAV1	MAPK1
UBC	MAPK1
TP53	MAPK1
MAPK1	TOB1
MAPK1	TNIP1
STAT5B	MAPK1
MAPK1	STAT5A
STAT3	MAPK1
SREBF1	MAPK1
SP1	MAPK1
SMAD1	MAPK1
RXRA	MAPK1
RPTOR	MAPK1
RPS6KA1	MAPK1
RAF1	MAPK1
MAPK1	PTPRH
MAPK1	PTPN7
MAPK1	PTPN5
PRKCE	MAPK1
MAPK1	PEA15
NCOA3	MAPK1
MYC	MAPK1
MAPK1	MBP
MAPKAPK2	MAPK1
MAPK1	MAP2K2
MAP2K1	MAPK1
LCK	MAPK1
MAPK1	LAMTOR3
MAPK1	KLF11
JUND	MAPK1

MAPK1	IQGAP1
IFNAR1	MAPK1
MAPK1	IER3
HDAC4	MAPK1
MAPK1	GAB2
ETS1	MAPK1
MAPK1	EIF4EBP1
MAPK1	DYRK1B
MAPK1	DUSP4
MAPK1	DUSP3
MAPK1	DUSP1
CASP9	MAPK1
C1QBP	MAPK1
ARRB2	MAPK1
UBC	MAP4K5
TRAF2	MAP4K5
UBC	MAP4K4
NCK1	MAP4K4
MAP3K7	MAP4K4
ITGB1	MAP4K4
MAP4K4	GBP3
SH3GL2	MAP4K3
GRB2	MAP4K3
UBC	MAP4K2
UBC	MAP4K1
NCK1	MAP4K1
MAP3K7	MAP4K1
MAP3K11	MAP4K1
GRB2	MAP4K1
GRAP2	MAP4K1
EGFR	MAP4K1
DBNL	MAP4K1
CRKL	MAP4K1
CRK	MAP4K1
ABL1	MAP4K1
UBC	MAP4
PXN	MAP4
TRAF2	MAP3K8
NFKB2	MAP3K8
MAP3K14	MAP3K8
MAP3K8	MAP2K4
MAP2K1	MAP3K8
CHUK	MAP3K8
MAP3K7	VRK2

UBC MAP3K7
MAP3K7 TUBB4A
MAP3K7 TUBB2B
TUBB MAP3K7
TRAF6 MAP3K7
MAP3K7 TRAF3IP2
TRAF2 MAP3K7
TNFRSF1A MAP3K7
MAP3K7 TLR3
MAP3K7 TGFBR1
TAB2 MAP3K7
TAB1 MAP3K7
MAP3K7 STAT3
RUVBL2 MAP3K7
RUVBL1 MAP3K7
RPS3 MAP3K7
MAP3K7 RIPK1
MAP3K7 PPP6C
MAP3K7 PPM1B
MAP3K7 PELI3
MAP3K7 NUDT21
NOD2 MAP3K7
MAP3K7 MAPK8IP1
MAP3K5 MAP3K7
MAP3K7 MAP2K4
MAP3K7 IARS
HSPA8 MAP3K7
HSPA5 MAP3K7
MAP3K7 HSPA1L
MAP3K7 HSP90AA1
MAP3K7 FKBP5
MAP3K7 ENO1
MAP3K7 DDX5
MAP3K7 CLTC
MAP3K7 CHUK
MAP3K7 CDC37
CALM1 MAP3K7
MAP3K7 BCL10
MAP3K5 MAP3K6
MAP3K5 MAP2K4
MAP3K5 GSTM1
MAP3K5 CDC25A
MAP3K4 MAP2K4
ZBTB16 MAP3K3

YWHAQ	MAP3K3
MAP3K3	YWHAH
YWHAG	MAP3K3
YWHAE	MAP3K3
YWHAB	MAP3K3
MAP3K3	XPOT
VIM	MAP3K3
MAP3K3	VCP
MAP3K3	URI1
MAP3K3	UBA52
MAP3K3	UACA
MAP3K3	TUBB6
MAP3K3	TUBB4A
MAP3K3	TUBB3
MAP3K3	TUBB2B
TUBB	MAP3K3
TRAF6	MAP3K3
MAP3K3	TMOD3
MAP3K3	TIMM50
MAP3K3	TFG
TCP1	MAP3K3
MAP3K3	SUGT1
MAP3K3	STUB1
MAP3K3	ST13
MAP3K3	SPTBN1
MAP3K3	SPTAN1
MAP3K3	SCRIB
MAP3K3	SCO2
MAP3K3	SCIN
MAP3K3	S100A8
MAP3K3	S100A7
RUVBL2	MAP3K3
RUVBL1	MAP3K3
MAP3K3	RQCD1
MAP3K3	RPS27L
MAP3K3	RPS27
MAP3K3	RPS16
MAP3K3	RPL23
MAP3K3	RPAP3
MAP3K3	RNF126
MAP3K3	RIPK1
RAI14	MAP3K3
PRKDC	MAP3K3
MAP3K3	PPP2R1B

MAP3K3	PPP1R12A
PPP1CC MAP3K3
PPP1CA MAP3K3
MAP3K3	PLOD1
MAP3K3	PIH1D1
MAP3K3	PHGDH
MAP3K3	PFKL
PFDN6	MAP3K3
PFDN5	MAP3K3
PFDN4	MAP3K3
PFDN2	MAP3K3
MAP3K3	PFDN1
MAP3K3	PDRG1
MAP3K3	NUDC
MAP3K3	MYO1D
MAP3K3	MYO1B
MAP3K3	MYO1A
MAP3K3	MYL12A
MAP3K3	MYH9
MYH10	MAP3K3
MAP3K3	MCC
MAP3K3	MATR3
MAP3K3	MARS
MAP3K7	MAP3K3
MAP3K5	MAP3K3
MAP3K3	MAP3K2
MAP3K3	MAP2K5
MAP3K3	LYN
MAP3K3	LONP1
MAP3K3	LIMA1
MAP3K3	LGALS7
MAP3K3	IQGAP2
MAP3K3	IQGAP1
MAP3K3	IKBKB
MAP3K3	IARS
HSPA9	MAP3K3
HSPA8	MAP3K3
MAP3K3	HSPA6
HSPA5	MAP3K3
MAP3K3	HSPA2
MAP3K3	HSPA1L
MAP3K3	HSP90AB1
MAP3K3	HSP90AA1
HNRNPH1	MAP3K3

MAP3K3	HAX1
MAP3K3	GNB2
MAP3K3	GNAI3
MAP3K3	GNAI2
MAP3K3	GAB1
MAP3K3	FASN
MAP3K3	EEF1A2
MAP3K3	DNAJC7
MAP3K3	DNAJB4
MAP3K3	DNAJA3
MAP3K3	DNAJA1
MAP3K3	DLG1
MAP3K3	CPVL
MAP3K3	CORO1C
MAP3K3	CLTC
MAP3K3	CHUK
MAP3K3	CFL2
MAP3K3	CDC37
MAP3K3	CCT8
CCT7	MAP3K3
MAP3K3	CCT6A
CCT5	MAP3K3
MAP3K3	CCT4
MAP3K3	CCT3
MAP3K3	CCT2
MAP3K3	CAPZB
CALM1	MAP3K3
C1QBP	MAP3K3
MAP3K3	BRCA1
MAP3K3	BAG5
MAP3K3	BAG2
ATP5A1	MAP3K3
MAP3K3	ALK
MAP3K3	ACTN4
YWHAG	MAP3K2
YWHAE	MAP3K2
YWHAB	MAP3K2
UBC	MAP3K2
PKN2	MAP3K2
MAP3K5	MAP3K2
MAP2K7	MAP3K2
MAP2K5	MAP3K2
MAP2K4	MAP3K2
YBX3	MAP3K14

YBX1	MAP3K14
TUBB	MAP3K14
TRAF1	MAP3K14
TIMM50	MAP3K14
SFPQ	MAP3K14
RUVBL2	MAP3K14
RSL1D1	MAP3K14
RPS6	MAP3K14
RPS3A	MAP3K14
RPS3	MAP3K14
RPS11	MAP3K14
RPL8	MAP3K14
RPL24	MAP3K14
RPL14	MAP3K14
RAP1B	MAP3K14
NFKB2	MAP3K14
HSPA8	MAP3K14
MAP3K14	HSPA6
HNRNPA1	MAP3K14
DHX9	MAP3K14
MAP3K14	DDX5
CHUK	MAP3K14
BIRC2	MAP3K14
PRDX3	MAP3K13
MAPK8IP1	MAP3K13
IKBKB	MAP3K13
UBC	MAP3K12
MAP3K12	RPL18A
RGS1	MAP3K12
MAP3K12	MAP3K11
MAP2K7	MAP3K12
MAP3K12	MAP2K4
MAP3K12	FTL
UBC	MAP3K11
TRAF6	MAP3K11
TRAF2	MAP3K11
MAPK8IP1	MAP3K11
MAP2K7	MAP3K11
MAP2K1	MAP3K11
MAP3K11	KIF3A
CHUK	MAP3K11
CDC42	MAP3K11
YWHAE	MAP3K10
RACGAP1	MAP3K10

PHB	MAP3K10
MAP2K4	MAP3K10
KIFAP3	MAP3K10
KIF3B	MAP3K10
KIF3A	MAP3K10
DNM1L	MAP3K10
CLTC	MAP3K10
CDC42	MAP3K10
YWHAE	MAP3K1
VIM	MAP3K1
UBE2I	MAP3K1
UBC	MAP3K1
MAP3K1	TUBB8
MAP3K1	TUBB6
MAP3K1	TUBB4A
MAP3K1	TUBB2B
TUBB	MAP3K1
TRAF2	MAP3K1
MAP3K1	TIMM50
TCP1	MAP3K1
MAP3K1	SNRPF
MAP3K1	SLC1A5
RUVBL2	MAP3K1
MAP3K1	RPS9
MAP3K1	RPS8
MAP3K1	RPS6
MAP3K1	RPS27L
MAP3K1	RPS27
MAP3K1	RPS23
MAP3K1	RPS16
MAP3K1	RPS13
MAP3K1	RPS11
MAP3K1	RPL7
MAP3K1	RPL4
MAP3K1	RPL37A
MAP3K1	RPL35A
MAP3K1	RPL35
MAP3K1	RPL30
MAP3K1	RPL3
MAP3K1	RPL24
MAP3K1	RPL18
MAP3K1	RPL14
MAP3K1	RPL13
MAP3K1	RPL10A

MAP3K1	NOP2
MAP3K1	NCL
MAP3K1	MYO1D
MYH10	MAP3K1
MAP3K1	MARS
MAPK9	MAP3K1
MAP3K1	MAPK1
MAP3K1	MAP4K2
MAP2K7	MAP3K1
MAP3K1	MAP2K4
MAP2K1	MAP3K1
MAP3K1	LGALS3BP
MAP3K1	IKBKB
MAP3K1	HSPH1
MAP3K1	HSPBP1
HSPA9	MAP3K1
HSPA8	MAP3K1
MAP3K1	HSPA6
HSPA5	MAP3K1
MAP3K1	HSPA1L
MAP3K1	HSP90AB1
MAP3K1	HSP90AA1
MAP3K1	HADHA
MAP3K1	GLUD1
MAP3K1	FKBP5
MAP3K1	FASN
ESR1	MAP3K1
MAP3K1	EEF1A2
MAP3K1	DNAJA3
MAP3K1	DNAJA1
DHX9	MAP3K1
MAP3K1	DCAF7
MAP3K1	CHUK
MAP3K1	CDC37
MAP3K1	CCT8
MAP3K1	CCT6A
CCT5	MAP3K1
MAP3K1	CCT4
MAP3K1	CCT3
CALM1	MAP3K1
BRAF	MAP3K1
MAP3K1	BAG5
MAP3K1	BAG2
AXIN1	MAP3K1

MAP2K7	MAP3K5
MAP2K7	DUSP19
UBC	MAP2K6
SMAD7	MAP2K6
PLCB2	MAP2K6
MAPK14	MAP2K6
MAP3K7	MAP2K6
MAP3K5	MAP2K6
MAP3K4	MAP2K6
LRRK2	MAP2K6
MAPK7	MAP2K5
UBC	MAP2K3
TAOK2	MAP2K3
MAP3K5	MAP2K3
MAP3K4	MAP2K3
MAP2K3	DYRK1B
UBC	MAP2K2
COPS5	MAP2K2
UBC	MAP2K1
MAP2K1	LAMTOR3
MAP2K1	IL6ST
MAP2K1	HRAS
MAP2K1	CPNE4
SQSTM1	MAP1LC3C
NBR1	MAP1LC3C
UBC	MAP1LC3B2
SQSTM1	MAP1LC3B
RAD1	MAP1LC3B
OPTN	MAP1LC3B
NBR1	MAP1LC3B
KBTBD7	MAP1LC3B
FYCO1	MAP1LC3B
ATG7	MAP1LC3B
ATG4B	MAP1LC3B
ATG3	MAP1LC3B
UBC	MAP1B
STK4	MAP1B
SNCA	MAP1B
MAPK8	MAP1B
MAP1LC3B	MAP1B
MAP1A	MAP1B
MAGOH	MAP1B
MAG	MAP1B
GAN	MAP1B

GABRR1	MAP1B
DYNLL1	MAP1B
ANP32A	MAP1B
MAP1A	DLG2
DLG1	MAP1A
AP1G1	MAP1A
UBC	MANF
VIM	MAN2A2
IKBKAP	MAN2A2
SMAD9	MAN1A2
NOTCH1	MAML3
RBPJ	MAML1
MAML1	NOTCH4
NOTCH3	MAML1
NOTCH2	MAML1
UBC	MALT1
TRAF6	MALT1
TRAF2	MALT1
BCL10	MALT1
UBC	MALSU1
TPD52L2	MAL2
TPD52L1	MAL2
TPD52	MAL2
UBC	MAK16
YWHAG	MAGOHB
UBC	MAGOHB
UBC	MAGOH
MAGOH	RNPS1
MAGOH	NXF1
MAGOH	IPO13
MAGOH	ALYREF
DSCAML1	MAGI3
TRIM27	MAGEL2
UBC	MAGEF1
RTN4R	MAG
MAG	COL9A3
COL9A2	MAG
UBC	MAFK
NFE2L2	MAFK
NFE2L1	MAFK
BRIP1	MAFK
BATF3	MAFK
PSAP	MAFF
NFE2L2	MAFF

NFE2L1 MAFF
NFE2L1 MAFB
MAF MAFB
FOSL1 MAFB
PDX1 MAFA
UBC MAEA
UBC MADD
UBC MAD2L2
CDC20 MAD2L2
ADAM9 MAD2L2
UBC MAD2L1BP
TRIP13 MAD2L1BP
TP53 MAD2L1BP
MAD2L1 MAD2L1BP
KRT15 MAD2L1BP
KAT5 MAD2L1BP
CDC20 MAD2L1BP
BUB1B MAD2L1BP
TSC22D4 MAD2L1
CDC16 MAD2L1
MAD2L1 BUB1B
MAD2L1 APC
ANAPC4 MAD2L1
UBC MAD1L1
TRIM29 MAD1L1
SIN3B MAD1L1
SIN3A MAD1L1
NINL MAD1L1
NDC80 MAD1L1
MAD2L1 MAD1L1
FAM131C MAD1L1
AMOTL2 MAD1L1
SKIL MACF1
PLIN3 M6PR
GGA3 M6PR
LZTS2 ZNF512B
UBC LZTS2
TRAF2 LZTS2
NINL LZTS2
CCDC85B LZTS2
UBC LYZ
LYZLCN1
YWHAQ LYST
YWHABLYST

UBC	LYST
ATN1	LYST
UBC	LYRM4
UBC	LYPLA2
SCMH1	LYPLA2
MED31	LYPLA2
UBC	LYPLA1
ANXA2	LYPLA1
UBC	LYN
LYN	TYK2
TRPV4	LYN
TEC	LYN
SYK	LYN
SNCA	LYN
SLC4A1	LYN
SKAP2	LYN
RGS16	LYN
PTPRC	LYN
PTPN6	LYN
PTK2B	LYN
LYN	PRAM1
LYN	PPP1R15A
PLCG2	LYN
PLCG1	LYN
PLAUR	LYN
LYN	PIK3CG
LYN	PDE4A
NMT1	LYN
LYN	MME
LCP2	LYN
KIT	LYN
KHDRBS1	LYN
JAK2	LYN
ITGB1	LYN
LYN	IL7R
LYN	HCLS1
GP6	LYN
GAB2	LYN
LYN	FOLR1
LYN	FCGR2B
EGFR	LYN
DOK1	LYN
LYN	CTLA4
CSF3R	LYN

LYN	CSF2RB
CD19	LYN
TCF3	LYL1
UBC	LYAR
MYBBP1A	LYAR
AP2M1	LY9
CD247	LY6E
C19orf10	LY6E
UBC	LUZP1
LURAP1	CCDC112
LUM	COL1A2
YWHAG	LUC7L3
UBC	LUC7L3
RNPS1	LUC7L3
UBE2D2	LTN1
UBC	LTN1
PIK3R1	LTK
PIK3C2B	LTK
UBC	LTF
LYZ	LTF
LCN1	LTF
CP	LTF
CD14	LTF
MGST1	LTC4S
LTBR	LTA
TGFB1	LTBP4
TGFB3	LTBP3
TGFB2	LTBP3
PRPF40A	LTBP3
ITGB5	LTBP3
ITGB5	LTBP1
FN1	LTBP1
GNA15	LTB4R
TNFRSF1A	LTB
LTA	LTB
UBC	LTA4H
TNFRSF1A	LTA
UBC	LSS
UBC	LSR
UBC	LSM7
LSM7	TACC1
LSM7	SNRPF
LSM7	MYH13
LSM7	LSM6

LSM5	LSM7
LSM4	LSM7
LSM2	LSM7
POLD2	LSM6
MCRS1	LSM6
LSM5	LSM6
LSM4	LSM6
XRN2	LSM3
XPC	LSM3
SNRPE	LSM3
SNRPD3	LSM3
LSM7	LSM3
LSM6	LSM3
LSM5	LSM3
LSM4	LSM3
LSM2	LSM3
CEP95	LSM3
UBC	LSM2
LSM2	RSRC1
LSM2	LSM6
LSM2	LSM5
LSM2	LSM4
LSM2	EXOSC10
LSM2	C14orf1
UBC	LSM14B
UBC	LSM14A
UBC	LSM12
LSM3	LSM10
VPS11	LSM1
UPF2	LSM1
LSM1	RRP1B
LSM1	NARS
N4BP1	LSM1
LSM7	LSM1
LSM6	LSM1
LSM5	LSM1
LSM4	LSM1
LSM1	LSM3
LSM2	LSM1
HSP90AA1	LSM1
LSM1	GRHPR
LSM1	FAM136A
EXOSC8	LSM1
CLIC1	LSM1

LSM1 ADIPOR2
UBC LSG1
UBC LRWD1
UBE2N LRSAM1
UBC LRSAM1
TSG101 LRSAM1
TNFRSF10C LRSAM1
RXRG LRSAM1
PHF23 LRSAM1
NINL LRSAM1
LRSAM1 MYL2
LRSAM1 MGAT4B
GABARAPL2 LRSAM1
VCL LRRN2
YWHAQ LRRK2
VIM LRRK2
UBC LRRK2
TUBB LRRK2
TUBA1A LRRK2
STUB1 LRRK2
PRKDC LRRK2
MBP LRRK2
MAP2K7 LRRK2
MAP2K3 LRRK2
LRRK2 LRRK1
HSPA8 LRRK2
HSP90AB1 LRRK2
HSP90AA1 LRRK2
EIF4EBP1 LRRK2
DNM1L LRRK2
CDC42 LRRK2
CDC37 LRRK2
AGO1 LRRK2
MYD88 LRRFIP2
FLII LRRFIP2
FLII LRRFIP1
UBC LRRC8E
UBC LRRC8A
CTNND2 LRRC7
CDH2 LRRC7
CAMK2A LRRC7
ACTN4 LRRC7
VCL LRRC59
TNFRSF1A LRRC59

UBC	LRRC58
NTNG1	LRRC4C
UBC	LRRC42
UBC	LRRC41
TCEB2	LRRC41
TCEB1	LRRC41
MYO9B	LRRC41
CUL5	LRRC41
UBC	LRRC28
KCTD17	LRRC18
UBC	LRR1
TCEB2	LRR1
TCEB1	LRR1
CUL2	LRR1
COPS6	LRR1
COPS5	LRR1
UBC	LRPPRC
LRPPRC	NFKBIB
VCL	LRPAP1
UBC	LRPAP1
LRPAP1	SORL1
PXN	LRPAP1
LRPAP1	PPM1G
POLA2	LRPAP1
LRPAP1	LRP8
UBC	LRP8
SNX17	LRP8
LRP8	APOH
LRP6	WNT1
UBC	LRP6
LRP6	SOST
LRP6	GSK3B
LRP6	DKK2
UBC	LRP5
THBS1	LRP5
SMAD9	LRP5
DKK1	LRP5
AXIN1	LRP5
DFNB31	LRP4
LRP2	TTR
LRP2	TG
SERPINE1	LRP2
LRP2	SCGB1A1
LRP2	PLAU

LRP2	MAPK8IP2
MAPK8IP1	LRP2
LRP2	MAGI1
LRPAP1	LRP2
LPL	LRP2
LDLRAP1	LRP2
LRP2	ITGB1BP1
LRP2	INS
GIPC1	LRP2
LRP2	DLG4
DLG1	LRP2
DAB2	LRP2
DAB1	LRP2
LRP2	CUBN
CLU	LRP2
LRP2	APOH
APOE	LRP2
LRP2	ANKRA2
ALB	LRP2
PLAUR	LRP1B
VCL	LRP1
UBC	LRP1
LRP1	THBS1
SRC	LRP1
SKIL	LRP1
SERPINE1	LRP1
SERPINA3	LRP1
SERPINA1	LRP1
PXN	LRP1
PLAUR	LRP1
LRP1	PLAT
PDGFB	LRP1
LRP1	MMP16
LRP1	MMP15
LRP1	MMP14
LRP1	MMP13
MAPK8IP1	LRP1
LRP1	LTF
LRPAP1	LRP1
LPL	LRP1
LRP1	ITGB1BP1
HSP90B1	LRP1
LRP1	GTPBP1
FURIN	LRP1

DLG4	LRP1
DAB1	LRP1
CTSG	LRP1
LRP1	CD82
CANX	LRP1
CALR	LRP1
LRP1	C4BPA
LRP1	C3
APP	LRP1
APOE	LRP1
APBB1	LRP1
A2M	LRP1
ERBB4	LRIG1
UBC	LRIF1
CA8	LRCH4
UBC	LRCH3
UBC	LRBA
RBP2	LRAT
RBP1	LRAT
UBC	LPXN
LPXN	LIMS1
LPXN	ITGA4
VASP	LPP
LPL	RPL18A
LPL	PTPN4
COPS6	LPL
LPL	ASCC2
LPL	APOC2
LPHN1	SHANK2
UBC	LPGAT1
UBC	LPCAT3
UBC	LPCAT2
UBC	LPCAT1
TRIP13	LOXL4
APMAP	LOXL3
VIM	LOR
PEX5	LONP2
UBC	LONP1
PUF60	LOH12CR1
TSC22D4	LNX1
TRIP13	LNX1
TRIM23	LNX1
TRAF2	LNX1
LNX1	TIFA

LNX1	RPIA
LNX1	RAD54B
LNX1	PBK
LNX1	PAICS
LNX1	NECAB2
LNX1	NAGK
KRT15	LNX1
LNX1	KLHL12
LNX1	KCTD13
LNX1	KCNA4
LNX1	FHL3
LNX1	FBP1
LNX1	ENOX1
LNX1	EHMT2
LNX1	DCTD
LNX1	CUTC
CEP72	LNX1
CCDC85B	LNX1
CALCOCO2	LNX1
LNX1	APIP
LNX1	AKIRIN2
TNKS	LNPEP
ISL1	LMX1A
ZBTB16	LMTK3
RALYL	LMO3
LMO2	TAL2
TAL1	LMO2
STAT1	LMO2
MAPRE3	LMO2
LMO2	LYL1
ISL1	LMO2
GATA3	LMO2
EHMT2	LMO2
DAZAP2	LMO2
LMO1	TAL2
TAL1	LMO1
LMO1	LYL1
GATA3	LMO1
UBC	LMNB2
UBC	LMNB1
TMPO	LMNB1
PRPH	LMNB1
PRKCD	LMNB1
PRKCB	LMNB1

PRKCA	LMNB1
LMNA	LMNB1
LBR	LMNB1
CASP6	LMNB1
CASP3	LMNB1
UBC	LMNA
LMNA	TMPO
LMNA	SYNE1
SUMO1	LMNA
LMNA	SREBF1
LMNA	PRKCA
LMNA	NARF
MYC	LMNA
KAT5	LMNA
LMNA	CASP1
UBC	LMF2
UBC	LMBR1
UBC	LMAN2
NFIC	LLPH
UBC	LLGL2
PRKCI	LLGL2
PARD6B	LLGL2
UBC	LITAF
SMAD2	LITAF
UBC	LIPT1
EXOSC5	LIPG
LRP1	LIPC
RTN4R	LINGO1
NGFR	LINGO1
FBXW7	LINGO1
EFEMP2	LINGO1
LIN7C	KCNJ12
KCNJ12	LIN7B
BAIAP2	LIN7B
APBA1	LIN7B
ABCA1	LIN7B
KCNJ12	LIN7A
ABCA1	LIN7A
RBL2	LIN54
RBBP4	LIN54
MYBL2	LIN54
LIN9	LIN54
LIN54	LIN52
LIN9	LIN52

LIN37	RBL2
RBBP4	LIN37
MYBL2	LIN37
LIN9	LIN37
LIN37	LIN54
LIN37	LIN52
ILK	LIMS2
UBC	LIMS1
ILK	LIMS1
EHMT2	LIMS1
ROCK1	LIMK2
HIPK3	LIMK2
CFL1	LIMK2
ROCK1	LIMK1
PAK1	LIMK1
LIMK1	CFL2
CDKN1C	LIMK1
LIMK1	CDC42BPA
UBC	LIMA1
PTPN6	LILRB4
PTPN6	LILRB2
HLA-G	LILRB2
LIG4	NHEJ1
XRCC1	LIG3
UBC	LIG3
LIG3	NEIL1
UBC	LIG1
UBC	LIFR
LIFR	OSM
IL6ST	LIFR
LIFR	LIF
IL6ST	LIF
SMAD2	LHX9
ISL1	LHX3
CITED2	LHX3
TSHR	LHCGR
LHB	LHCGR
GNAI2	LHCGR
CGB	LHCGR
CGA	LHCGR
UBC	LGMN
UBC	LGALS8
ITGB1	LGALS8
ITGA5	LGALS8

ITGA4	LGALS8
ITGA3	LGALS8
CALCOCO2	LGALS8
CA12	LGALS7
RRAS2	LGALS4
ARPC3	LGALS4
VCL	LGALS3BP
UBC	LGALS3BP
TBK1	LGALS3BP
TAB2	LGALS3BP
NFKB2	LGALS3BP
ITGB1	LGALS3BP
FN1	LGALS3BP
SUFU	LGALS3
MUC1	LGALS3
LIM2	LGALS3
LGALS3BP	LGALS3
ITGB1	LGALS3
ELN	LGALS3
EGFR	LGALS3
CUBN	LGALS3
CSNK2A2	LGALS3
CSNK1A1	LGALS3
LTA	LGALS2
VCL	LGALS1
UBC	LGALS1
SPP1	LGALS1
PXN	LGALS1
LGALS1	PTPRC
LGALS3BP	LGALS1
HRAS	LGALS1
EGFR	LGALS1
LGALS1	CD8A
LGALS1	CD2
KLRG1	LEPROTL1
UBC	LEPRE1
SNX2	LEPR
LEPR	SNX1
PIN1	LEPR
JAK2	LEPR
LEPR	DGKZ
CLU	LEPR
LEPR	APOD
PRKAA2	LEP

LEPR	LEP
HK3	LEP
GHRL	LEP
CLU	LEP
UBC	LEO1
LEO1	CDC73
UBC	LENG9
PNMA1	LENG1
UBC	LEMD3
SMAD9	LEMD3
SMAD2	LEMD3
SMAD1	LEMD3
UBTF	LEF1
UBC	LEF1
TLE1	LEF1
SMAD4	LEF1
SMAD2	LEF1
SMAD1	LEF1
LEF1	NLK
MITF	LEF1
KPNA2	LEF1
KPNA1	LEF1
LEF1	ALX4
UBC	LDLR
SNX17	LDLR
PF4 LDLR	
PCSK9	LDLR
MYLIP	LDLR
LRPAP1	LDLR
LDLRAP1	LDLR
HSPA5	LDLR
DAB1	LDLR
APOE	LDLR
APOB	LDLR
AP1M2	LDLR
CSRP3	LDHD
UBC	LDHB
TBK1	LDHB
UBC	LDHAL6A
XRN1	LDHA
VCL	LDHA
UBC	LDHA
NDRG1	LDHA
LDHB	LDHA

HNRNPD	LDHA
LMO4	LDB2
LHX4	LDB2
UBC	LDB1
TRIM33	LDB1
TOLLIP	LDB1
TAL1	LDB1
LDB1	SSBP4
SSBP2	LDB1
LMO4	LDB1
LMO3	LDB1
LDB1	LMO2
VAV1	LCP2
LCP2	SHB
LCP2	PRAM1
PLCG2	LCP2
LCP2	ITK
GRAP2	LCP2
UBC	LCP1
GCA	LCP1
MMP9	LCN2
UBC	LCMT2
FXR2	LCMT1
UBC	LCLAT1
LCK	STAT1
UBC	LBR
SRPK1	LBR
YAP1	LATS2
YAP1	LATS1
WWTR1	LATS1
UBC	LATS1
STK3	LATS1
MOB1B	LATS1
MOB1A	LATS1
LIMK1	LATS1
ITCH	LATS1
HTRA2	LATS1
CDK1	LATS1
UBC	LAT2
PLCG2	LAT2
PLCG1	LAT2
GRB2	LAT2
VAV1	LAT
UBC	LAT

LAT	SLA
LAT	SHB
PLCG2	LAT
LCP2	LAT
LCK	LAT
LAT	ITK
GRAP2	LAT
LAT	GRAP
LAT	GAB2
LAT	FCGR2A
UBC	LASP1
LASP1	TRIP13
UBC	LARS
TANK	LARS
IARS	LARS
AARS	LARS
MEPCE	LARP7
HNRNPA1	LARP7
CDK9	LARP7
UBC	LARP4B
GNB2L1	LARP4B
UBC	LARP4
UBC	LARP1B
YWHAQ	LARP1
YWHAG	LARP1
YWHAE	LARP1
YWHAB	LARP1
UBC	LARP1
UBC	LAPTM5
LAPTM5	TOM1L2
TNFAIP3	LAPTM5
LAPTM5	SMURF2
LAPTM5	RNF168
NEDD4L	LAPTM5
NEDD4	LAPTM5
LAPTM5	KRT7
LAPTM5	HEY1
LAPTM5	HERC1
LAPTM5	EPN3
LAPTM5	EPN1
UBC	LANCL2
LAMTOR5	RABIF
LAMTOR5	LAMTOR4
EEF1A1	LAMTOR5

UBC LAMTOR3
SUFU LAMTOR3
LAMTOR3 LAMTOR2
UBC LAMTOR1
ATP6V1B1 LAMTOR1
AP1M1 LAMP1
BMP1 LAMC2
UBC LAMC1
LAMC1 CCDC53
UBC LAMB3
BMP1 LAMB3
UBC LAMB1
LAMA1 LAMB1
UBC LAMA5
SMAD2 LAMA5
MEP1A LAMA5
LAMC1 LAMA5
FBLN2 LAMA5
DAG1 LAMA5
BTBD10 LAMA5
LAMA4 UBR1
TP53 LAMA4
COPS6 LAMA4
LAMA4 BRD7
SDC2 LAMA3
PLGLAMA3
BMP1 LAMA3
DAG1 LAMA2
UBC LAMA1
PLGLAMA1
PLAT LAMA1
MEP1A LAMA1
ITGB1 LAMA1
LAMA1 ITGA1
FBLN2 LAMA1
DAG1 LAMA1
PTPN6 LAIR1
CENPJ LAG3
CEBPZ LAG3
COL17A1 LAD1
UBC LACTB2
UBC LACRT
STAM2 L3MBTL2
RNF2 L3MBTL2

PHF10	L3MBTL2
PCGF6	L3MBTL2
HIST4H4	L3MBTL2
HIST4H4	L3MBTL1
ZNF512B	L1TD1
ZBTB25	KXD1
UTP6	KXD1
TPM3	KXD1
TPM1	KXD1
TCEB3B	KXD1
STX11	KXD1
RNF183	KXD1
RABGEF1	KXD1
NUDT18	KXD1
NIF3L1	KXD1
MOB1A	KXD1
MCRS1	KXD1
MAP1LC3B	KXD1
LNX1	KXD1
LMO3	KXD1
ING5	KXD1
IFT20	KXD1
FXR2	KXD1
EXOC7	KXD1
CEP63	KXD1
CEP19	KXD1
CCDC33	KXD1
UBC	KTN1
IKBKE	KTN1
UBC	KTI12
KSR1	VRK2
RAF1	KSR1
MAP2K2	KSR1
MAP2K1	KSR1
HOXA1	KRTAP4-12
TRIP13	KRTAP3-2
KRT9	TANK
UBC	KRT86
UBC	KRT84
SKIL	KRT81
KXD1	KRT81
KRT15	KRT81
UBC	KRT8
PNN	KRT8

KRT8	PLAT
KRT8	PKP1
MAPK3	KRT8
MAPK14	KRT8
MAPK1	KRT8
HSPA5	KRT8
EGFR	KRT8
DSP	KRT8
BYSL	KRT8
KRT8	ANXA1
UBC	KRT79
UBC	KRT78
UBC	KRT77
UBC	KRT76
UBC	KRT75
UBC	KRT74
UBC	KRT73
UBC	KRT72
UBC	KRT71
UBC	KRT6B
KRT19	KRT6B
KRT15	KRT6B
KRT6A	KRT17
KRT15	KRT6A
DSP	KRT6A
CCDC85B	KRT6A
KRT5	KRT14
UBC	KRT3
KRT20	VIM
KRT15	KRT20
FXR2	KRT20
KRT20	EXOC8
KRT20	EIF4E2
CCDC85B	KRT20
KRT20	C9orf16
KRT20	AMOTL2
KRT20	ABI2
UBC	KRT2
KRT19	KRT15
KRT19	C10orf10
KRT19	ABI2
YWHAQ	KRT18
YWHAH	KRT18
YWHAG	KRT18

YWHAE	KRT18
YWHAB	KRT18
UBC	KRT18
TNFRSF1B	KRT18
SFN	KRT18
RHPN2	KRT18
PRKCE	KRT18
PRKCA	KRT18
PNN	KRT18
PKP2	KRT18
NME7	KRT18
MAPK14	KRT18
KRT8	KRT18
KRT5	KRT18
KRT15	KRT18
HSPA5	KRT18
EXOC8	KRT18
EGFR	KRT18
DSP	KRT18
DEDD	KRT18
COL17A1	KRT18
CCDC85B	KRT18
CBL	KRT18
CASP3	KRT18
KRT7	KRT17
UBC	KRT16
TANK	KRT16
KRT15	C1orf216
KRT15	ABI3
KRT15	ABI2
UBC	KRT12
UBC	KRT1
TANK	KRT1
PRKCE	KRT1
DSP	KRT1
UBC	KRR1
RAP1A	KRIT1
ITGB1BP1	KRIT1
CCM2	KRIT1
UBC	KRAS
KRAS	SHOC2
RASGRP2	KRAS
RAF1	KRAS
KRAS	PIK3CG

HNRNPC	KRAS
CALM1	KRAS
UBC	KPNB1
TRAF6	KPNB1
TNFRSF1B	KPNB1
KPNB1	TERF1
SNUPN	KPNB1
SMAD2	KPNB1
RELB	KPNB1
RANBP1	KPNB1
RAN	KPNB1
KPNB1	PTHLH
NXF1	KPNB1
NUP98	KPNB1
NUP62	KPNB1
NUP153	KPNB1
KPNB1	NCBP2
KPNB1	KPNA6
KPNA4	KPNB1
KPNB1	KPNA3
KPNA2	KPNB1
KPNA1	KPNB1
IPO8	KPNB1
IPO7	KPNB1
UBC	KPNA6
SKP2	KPNA6
UBC	KPNA5
UBC	KPNA3
RCC1	KPNA3
HNRNPC	KPNA3
MYC	KPNA2
ITK	KPNA2
KPNA1	STAT1
LMO4	KPNA1
UBC	KNSTRN
MPHOSPH6	KNSTRN
VTN	KNG1
THBS1	KNG1
PLG	KNG1
PLAUR	KNG1
KRT1	KNG1
KLK1	KNG1
ITGB2	KNG1
GP5	KNG1

F2	KNG1
F11	KNG1
CTSK	KNG1
CTSG	KNG1
CD93	KNG1
CAPN2	KNG1
CAPN1	KNG1
APP	KNG1
HLA-G	KLRD1
TYROBP	KLRC4
MICA	KLRC4
KLRD1	KLRC3
TYROBP	KLRC2
KLRD1	KLRC2
HLA-E	KLRC2
KLRC1	KLRD1
SERPINA5	KLKB1
KNG1	KLKB1
HGF	KLKB1
UBC	KLK7
SERPINA5	KLK3
KLK3	SERPINA3
SERPINA1	KLK3
KLK3	PTHLH
KLK3	IGFBP3
FN1	KLK3
EPOR	KLK3
A2M	KLK3
SPINK5	KLK14
SERPINF2	KLK13
SERPINA5	KLK1
KLK1	SERPINA4
UBC	KLHL8
COPS6	KLHL8
UBC	KLHL7
UBC	KLHL6
CUL3	KLHL5
USHBP1	KLHL42
UBQLN1	KLHL42
UBC	KLHL42
SERTAD1	KLHL42
RCN3	KLHL42
CUL3	KLHL42
COPS6	KLHL42

UBC	KLHL41
WNK4	KLHL3
WNK2	KLHL3
CUL3	KLHL3
UBC	KLHL26
UBC	KLHL21
CUL3	KLHL21
UBC	KLHL20
PIN1	KLHL20
GBP2	KLHL20
KLHL20	ECT2
C14orf1	KLHL20
KLHL12	KLHL2
CUL3	KLHL2
EEF1G	KLHL18
COPS6	KLHL18
COPS5	KLHL18
UBC	KLHL11
UBC	KLHDC4
CUL2	KLHDC3
UBC	KLHDC2
CUL5	KLHDC2
CUL2	KLHDC2
CUL2	KLHDC10
PGR	KLF9
UBC	KLF6
SP1	KLF6
POLA2	KLF6
PNO1	KLF6
KLF4	KLF6
GTF3C1	KLF6
EHMT2	KLF6
CELSR2	KLF6
UBC	KLF5
KLF5	TRIM25
SMAD2	KLF5
KLF5	RARA
KLF5	NFKB1
KLF5	GTF2F2
KLF5	GTF2E2
KLF5	GSK3B
KLF5	FBXW7
UBC	KLF4
SP1	KLF4

HDAC2	KLF4
CREBBP	KLF4
UBC	KLF2
UBC	KLF13
SERPINH1	KLF13
KAT2B	KLF13
EHMT2	KLF12
ZNF512B	KLF10
UBC	KLF10
SP1	KLF10
SMARCC1	KLF1
HDAC1	KLF1
CSNK2A2	KLF1
CSNK2A1	KLF1
CREBBP	KLF1
KLC1	KLC4
KLC3	KIF5A
YWHAB	KLC1
UBC	KLC1
MAPK8IP1	KLC1
LRSAM1	KLC1
KLC3	KLC1
KIF5B	KLC1
KIF5A	KLC1
APP	KLC1
UBC	KITLG
KIT	KITLG
TEC	KIT
STAT1	KIT
SPRED2	KIT
KIT	GRAP
NPHS2	KIRREL3
HLA-A	KIR3DL2
HLA-C	KIR2DL1
RPA1	KIN
UBC	KIFC1
UBC	KIFAP3
KIFAP3	KIF3B
APC	KIFAP3
UBC	KIF5C
SNAPIN	KIF5C
YWHAB	KIF5B
UBC	KIF5B
RAB4A	KIF3B

LMO4	KIF3B
KIF3A	KIF3C
KIF3A	KIF3B
UBC	KIF2C
SUMO2	KIF2C
MAPRE1	KIF2C
UBC	KIF2A
UBC	KIF22
UBC	KIF20B
UBC	KIF1B
PPP1CA	KIF18A
VIM	KIF15
MKI67	KIF15
CHD3	KIF15
UBC	KIF14
DLG1	KIF13B
GTF2H1	KIF13A
UBC	KIF11
PINK1	KIF11
IKBKE	KIF11
DCTN1	KIF11
UBC	KIDINS220
MOB4	KIDINS220
UBC	KIAA1731
UBC	KIAA1586
MYO5B	KIAA1549
MBD5	KIAA1549
ITSN2	KIAA1549
CADPS	KIAA1549
UBC	KIAA1524
UBC	KIAA1468
UBC	KIAA1432
UBC	KIAA1429
PIN1	KIAA1429
ZNF24	KIAA1377
YAE1D1	KIAA1377
TXNDC9	KIAA1377
TOMM20	KIAA1377
TFG	KIAA1377
STAU2	KIAA1377
SPDL1	KIAA1377
SNRPG	KIAA1377
RUVBL1	KIAA1377
RPA2	KIAA1377

RAN	KIAA1377
RAB27A	KIAA1377
PTPRS	KIAA1377
POLD1	KIAA1377
PFDN1	KIAA1377
PDCD5	KIAA1377
PBK	KIAA1377
NUDT21	KIAA1377
NSF	KIAA1377
NPM3	KIAA1377
NAT9	KIAA1377
NAP1L5	KIAA1377
MRPS6	KIAA1377
MAD2L1BP	KIAA1377
LRRC1	KIAA1377
LPL	KIAA1377
LAMTOR5	KIAA1377
KLHL20	KIAA1377
KIF15	KIAA1377
HMOX2	KIAA1377
GSTO1	KIAA1377
GOLGB1	KIAA1377
GET4	KIAA1377
FXR1	KIAA1377
EPN1	KIAA1377
EIF2S2	KIAA1377
DUSP12	KIAA1377
DNM1	KIAA1377
CRCT1	KIAA1377
CDKN2B	KIAA1377
ATP6V1F	KIAA1377
ARIH2	KIAA1377
AKTIP	KIAA1377
ZNF670	KIAA1279
ZNF638	KIAA1279
UBC	KIAA1279
TCP10L	KIAA1279
RGR	KIAA1279
PLEKHF1	KIAA1279
DOK2	KIAA1279
CEP76	KIAA1279
KRT15	KIAA1217
GCC1	KIAA1217
FXR2	KIAA1217

UBC	KIAA1109
UBC	KIAA1033
SMAD2	KIAA1033
FAM21C	KIAA1033
YWHAG	KIAA0930
YWHAB	KIAA0930
U2AF2	KIAA0907
UBC	KIAA0754
UBC	KIAA0753
UBC	KIAA0430
YWHAG	KIAA0408
VIM	KIAA0408
TRIM37	KIAA0408
TRAF2	KIAA0408
SFN	KIAA0408
MDFI	KIAA0408
KRT15	KIAA0408
GFAP	KIAA0408
CEP72	KIAA0408
CCDC85B	KIAA0408
KIAA0408	CARD9
BEGAIN	KIAA0408
ABI3	KIAA0408
UBC	KIAA0368
CLN3	KIAA0368
KIAA0319	FEM1B
UBC	KIAA0196
KIAA1033	KIAA0196
UBC	KIAA0195
UBC	KIAA0101
UBC	KIAA0100
UBC	KHSRP
HNRNPA1	KHSRP
UBC	KHNYN
RNF11	KHNYN
UBC	KHK
ZNF408	KHDRBS3
SRSF9	KHDRBS3
SIAH1	KHDRBS3
SGSM2	KHDRBS3
RBM7	KHDRBS3
PSMF1	KHDRBS3
NUDT18	KHDRBS3
LNX1	KHDRBS3

KHDRBS2	KHDRBS3
KHDRBS1	KHDRBS3
HNRNPK	KHDRBS3
CCDC33	KHDRBS3
BAHD1	KHDRBS3
UBC	KHDRBS2
KHDRBS1	VCL
VAV1	KHDRBS1
UBC	KHDRBS1
SUMO1	KHDRBS1
KHDRBS1	STUB1
KHDRBS1	STAT3
SMAD2	KHDRBS1
KHDRBS1	PTK6
PRMT1	KHDRBS1
PLCG2	KHDRBS1
KHDRBS1	PLCG1
PIK3R1	KHDRBS1
LCK	KHDRBS1
KHDRBS1	ITK
IRS1	KHDRBS1
KHDRBS1	HCK
GRAP2	KHDRBS1
KHDRBS1	GAS7
KHDRBS1	FYN
KHDRBS1	DHX9
CRK	KHDRBS1
KHDRBS1	ACTB
UBC	KEAP1
TSC22D4	KEAP1
SQSTM1	KEAP1
SNRPN	KEAP1
KEAP1	SLK
RELA	KEAP1
NFE2L2	KEAP1
NFE2L1	KEAP1
MAD2L1	KEAP1
FAM46A	KEAP1
KEAP1	ETF1
DPP3	KEAP1
CUL3	KEAP1
UBC	KDR
KDR	SHB
KDR	SH2D2A

PTPN6 KDR
PLCG2 KDR
KDR FLT4
KDR FLT1
CRK KDR
KDR CDH5
UBC KDM5B
FOXG1 KDM5B
UBC KDM5A
TBP KDM5A
RB1 KDM5A
RARA KDM5A
LMO2 KDM5A
ESR1 KDM5A
UBC KDM4B
MED10 KDM4B
UBC KDM4A
HIST4H4 KDM4A
HIST1H3A KDM4A
UBC KDM3A
SKP1 KDM2B
KDM2B RYBP
RNF2 KDM2B
UBC KDM1B
BAP1 KDM1B
KDM1A ZNF217
UBC KDM1A
TP53 KDM1A
KDM1A SNAI1
KDM1A RCOR3
KDM1A RBBP4
KDM1A PHF21A
KDM1A NR2C2
KDM1A NMI
KDM1A MTA2
KDM1A HMG20A
KDM1A HIST3H3
KDM1A HIST1H3A
HDAC2 KDM1A
KDM1A HDAC1
KDM1A GSE1
KDM1A DNMT1
KDM1A CTBP1
KDM1A CCDC90B

KDM1A	ASCC2
UBC	KDELR1
COPB1	KDELR1
UBC	KCTD6
UBC	KCTD3
KCTD17	RWDD4
KCTD17	PSMA1
NUDT18	KCTD17
NIF3L1	KCTD17
LNX1	KCTD17
AQP1	KCTD17
UBC	KCTD16
ENSA	KCTD15
UBC	KCTD14
KCTD13	ZMYND19
UBC	KCTD13
KCTD13	POLD2
UBC	KCTD12
TNFRSF1B	KCTD12
RELA	KCTD12
CUL3	KCTD11
UBC	KCTD10
SUMO2	KCTD1
FXR2	KCNRG
DGCR6L	KCNRG
CCNK	KCNRG
AP5B1	KCNRG
KCNQ3	KCNQ4
KCNQ3	KCNQ2
CALM1	KCNQ2
ARIH2	KCNQ2
UBC	KCNQ1
TRAF6	KCNQ1
KCNE2	KCNQ1
AKAP9	KCNQ1
UBC	KCNN4
CALM1	KCNN4
UBC	KCNN3
UBC	KCNN2
YWHAB	KCNK9
UBC	KCNK5
UBC	KCNK1
KCNJ11	KCNJ8
KCNJ9	KCNJ6

KCNJ3	KCNJ6
KCNJ3	KCNJ5
LIN7C	KCNJ4
KCNJ4	GNGT2
DLG1	KCNJ4
KCNJ9	KCNJ3
KCNJ8	KCNJ2
DLG1	KCNJ2
KCNJ16	KCNJ15
DLG1	KCNJ12
KCNJ16	KCNJ10
DLG1	KCNJ10
SLC9A3R2	KCNJ1
SLC9A3R1	KCNJ1
PSEN1	KCNIP3
CREM	KCNIP3
KCND2	KCNIP1
KCNB1	KCNG1
KCNH1	KCNF1
KCNH2	KCNE2
KCND2	KCNIP2
UBC	KCNAB2
KCNA2	KCNAB2
CNTNAP2	KCNAB2
DLG1	KCNAB1
DLG1	KCNA5
ERBB2IP	KCNA4
KCNA4	DLGAP1
DLG1	KCNA4
DLG1	KCNA3
PTK2B	KCNA2
KCNA2	KCNAB1
KCNA5	KCNA2
KCNA4	KCNA2
KCNA2	ETV1
CTTN	KCNA2
KCNE4	KCNA1
KCNAB2	KCNA1
DLG4	KCNA1
UBC	KBTBD8
UBC	KBTBD7
GABARAPL2	KBTBD7
UBC	KBTBD6
UBC	KBTBD4

UBC	KBTBD2
KAT7	KATNBL1
HAP1	KATNBL1
CA12	KATNBL1
UBC	KATNAL1
COX17	KATNAL1
UBC	KATNA1
KATNB1	KATNA1
KAT7	WDR33
KAT7	VIM
UBC	KAT7
KAT7	RPS10
KAT7	MCM2
KAT7	KCTD13
KAT7	ING5
KAT7	CBX8
KAT7	CAAP1
UBC	KAT6B
RUNX2	KAT6B
ING5	KAT6A
HIST3H3	KAT6A
KAT5	UHRF1
UBC	KAT5
KAT5	TRRAP
KAT5	TRIB3
TP53	KAT5
KAT5	TELO2
KAT5	SIRT1
KAT5	RUVBL2
KAT5	POLE2
KAT5	PLEKHA4
KAT5	PLA2G4A
KAT5	MYC
KAT5	MDM2
KAT5	IK
KAT5	HNRNPH3
KAT5	GEMIN7
KAT5	FAM101B
KAT5	EP400
KAT5	EFNA1
CDK5RAP2	KAT5
KAT5	CDC42
KAT5	CCT7
KAT5	CCDC106

KAT5	CBX8
KAT5	BRD8
KAT5	ATM
KAT5	APP
KAT5	ACTL6A
KAT5	ACTB
TRRAP	KAT2B
TP53	KAT2B
TAF9	KAT2B
SMAD2	KAT2B
PGR	KAT2B
NCOA4	KAT2B
MYC	KAT2B
MDM2	KAT2B
KAT2B	H3F3A
UBC	KAT2A
KAT2A	TRRAP
KAT2A	TAF9
KAT2A	TADA2A
SIRT1	KAT2A
MYC	KAT2A
MED1	KAT2A
HIST4H4	KAT2A
UBC	KARS
TANK	KARS
SOD1	KARS
PAFAH1B1	KARS
NARS	KARS
MARS	KARS
FRS3	KARS
EEF1G	KARS
AIMP2	KARS
AIMP1	KARS
KRT15	KANSL1
CCDC85B	KANSL1
YWHAQ	KANK1
PPP1CA	KANK1
KIF21A	KANK1
UBC	JUP
JUP	PSEN1
JUP	PKP2
JUP	MUC1
JUP	DSP
JUP	DSG2

JUP DSG1
JUP DSC1
CUL2 JUP
JUP CTNNA1
JUP CDH5
JUP CDH2
JUP BIRC2
JUP ARHGDIA
JUP APC
UBC JUND
SMAD4 JUND
RFWD2 JUND
NFE2L1 JUND
MEN1 JUND
JUND MAFK
JUND FOSL1
COPS5 JUND
BCL6 JUND
BATF3 JUND
UBC JUNB
SMAD4 JUNB
NINL JUNB
NFE2L1 JUNB
MAPK9 JUNB
MAPK14 JUNB
MAFK JUNB
JUNB FOSL1
BCL6 JUNB
BATF3 JUNB
UBE2I JUN
UBC JUN
JUN UBB
JUN TRIP4
JUN TPM2
TOP2A JUN
JUN TCF20
JUN TBP
SUMO2 JUN
SUMO1 JUN
JUN STAT4
JUN SPIB
JUN SPI1
SP1 JUN
SMAD4 JUN

SMAD3 JUN
SMAD2 JUN
JUN SKI
SIRT1 JUN
RUNX2 JUN
JUN RNF187
JUN RFWD2
JUN RELA
JUN RB1
PRKD1 JUN
JUN POU1F1
JUN PLEKHO1
PIN1 JUN
JUN PACS1
JUN NRIP1
JUN NR5A1
JUN NOS2
JUN NFE2L2
JUN NFE2L1
NFATC2 JUN
NCOA1 JUN
JUN MYOD1
JUN MYBBP1A
MAPK9 JUN
JUN MAPK10
JUN MAPK1
JUN MAFK
JUN MAF
JUN JUND
JUN JUNB
JUN ITGAX
JUN ITCH
JUN IL1B
JUN IFNG
JUN IFNB1
JUN HSP90AA1
JUN HMGA1
JUN HLF
JUN HIF1A
JUN GTF2F2
GSK3B JUN
JUN GATA2
JUN FOSL2
JUN FOSL1

JUN	FOSB
FOS	JUN
ETS2	JUN
JUN	ETS1
ESR1	JUN
JUN	ERG
JUN	DDX21
JUN	DDIT3
JUN	DACH1
JUN	CTNNB1
CREBBP	JUN
JUN	CREB5
COPS5	JUN
JUN	COPS2
JUN	CEBPE
JUN	BRCA1
BCL6	JUN
JUN	BATF3
JUN	BATF2
JUN	BATF
JUN	BACH2
JUN	ATF4
JUN	ATF1
JUN	APLP2
ABL1	JUN
UBC	JOSD2
UBC	JOSD1
UBC	JMJD6
RSRC1	JMJD6
BRD4	JMJD6
UBC	JMJD4
RXRA	JMJD1C
PGR	JDP2
MAP3K7	JAZF1
TJP1	JAM2
JAM3	JAM2
F2RL2	JAM2
TRIM29	JAKMIP2
LCK	JAK3
JAK3	CXCR4
JAK3	ALK
JAK2	VCP
VAV1	JAK2
UBC	JAK2

JAK2 TUB
JAK2 TNFRSF1A
JAK2 TEC
JAK2 STAT5B
JAK2 STAT5A
JAK2 STAT3
JAK2 STAT1
JAK2 STAM2
RAF1 JAK2
PTPN6 JAK2
JAK2 PTPN12
JAK2 PTK2B
PRMT5 JAK2
JAK2 PPP2R5A
JAK2 PPIA
JAK2 PLCG1
PIK3R1 JAK2
JAK2 PDGFRB
JAK2 KIT
JAK2 JAK3
IRS1 JAK2
JAK2 IL5RA
JAK2 IFNGR2
JAK2 IFNGR1
HSPA8 JAK2
GTF2I JAK2
JAK2 GHR
JAK2 FYN
ERBB3 JAK2
EGFR JAK2
JAK2 CXCR4
JAK2 CTLA4
JAK2 CSF3R
JAK2 CSF2RB
JAK2 CCR5
JAK2 AGTR1
TNFRSF1A JAK1
TECJAK1
STAT6 JAK1
STAT1 JAK1
STAM2 JAK1
JAK1 SHB
JAK1 PLAUR
JAK3 JAK1

JAK1 IL6ST
JAK1 IL2RB
JAK1 IL10RA
JAK1 IFNAR1
JAK1 GHR
UBC JAG1
NOTCH3 JAG1
NOTCH2 JAG1
VIM IVNS1ABP
UBC IVNS1ABP
PFN2 IVNS1ABP
SPRR3 IVL
ITSN2 SYNJ2
ITSN2 SEMA6A
UBC ITSN1
ITSN1 SNAP25
LMO4 ITSN1
ITSN1 KIF5A
ITSN1 EPHB2
ITSN1 DNM2
UBC ITPRIPL1
UBC ITPRIP
UBC ITPR3
TRPC3 ITPR3
ITPR3 TRPC1
ITPR3 CABP1
ITPR3 ANK1
UBC ITPR2
UBC ITPR1
TRPC4 ITPR1
TRPC3 ITPR1
PRKACA ITPR1
HOMER2 ITPR1
GRM1 ITPR1
FKBP1AITPR1
ERLIN2 ITPR1
EPB41L1 ITPR1
CNN1 ITPR1
CABP1 ITPR1
CA8ITPR1
PRKCA ITPKA
UBC ITPA
UBC ITM2C
STT3A ITM2C

RNF7	ITM2C
UBC	ITM2B
LCK	ITK
ATP5C1	ITIH2
TGFB1	ITGB8
ITGAV	ITGB8
ITGA4	ITGB7
ITGB7	GULP1
FN1	ITGB7
EED	ITGB7
FN1	ITGB6
UBC	ITGB4
PRKCA	ITGB4
MET	ITGB4
ITGB4	ITGA6
ITGB4	COL17A1
ITGB4	CLCA1
THRA	ITGB3BP
RXRA	ITGB3BP
RELA	ITGB3BP
RECQL5	ITGB3BP
NFKB1	ITGB3BP
ITGB3	ITGB3BP
DDX24	ITGB3BP
COL4A3BP	ITGB3BP
CCNA2	ITGB3BP
ARFIP2	ITGB3BP
TLN1	ITGB3
ITGB3	GULP1
ITGB1	ITGB1BP1
ITGB1	TLN1
ITGB1	SPP1
ITGB1	SLC3A2
PRKCA	ITGB1
ITGB5	ITGB1
ITGB1	ITGB3
ITGB1	ILK
ITGB1	GULP1
ITGB1	FN1
ITGB1	CD151
ITGB1	ARHGAP5
ITGB2	ITGAX
ITGAX	FCER2
UBC	ITGAV

TGFB1	ITGAV
ITGB6	ITGAV
ITGB5	ITGAV
ITGB3	ITGAV
ITGB1	ITGAV
ITGAV	F2R
ITGAV	AZGP1
ITGB2	ITGAM
ICAM1	ITGAM
HP	ITGAM
ICAM1	ITGAL
ITGB7	ITGAE
EED	ITGAE
CHFR	ITGAE
ITGB2	ITGAD
PXN	ITGA9
ITGB1	ITGA9
ITGB1	ITGA8
UBC	ITGA6
PXN	ITGA6
ITGB1	ITGA6
ITGA6	COL17A1
UBC	ITGA5
ITGB5	ITGA5
ITGA5	ITGB3
ITGB1	ITGA5
GIPC1	ITGA5
FN1	ITGA5
ITGA5	F2
EGFR	ITGA5
COL1A1	ITGA5
ITGA5	CD9
VCAN	ITGA4
UBC	ITGA4
PXN	ITGA4
ITGB1	ITGA4
FN1	ITGA4
EED	ITGA4
CXCR4	ITGA4
UBC	ITGA3
ITGA3	TIMP2
RABIF	ITGA3
ITGB1	ITGA3
CD151	ITGA3

CALR	ITGA3
VWF	ITGA2B
RNF181	ITGA2B
ITGB3	ITGA2B
GRB2	ITGA2B
FGA	ITGA2B
F2	ITGA2B
COL2A1	ITGA2B
CIB1	ITGA2B
UBC	ITGA2
MMP1	ITGA2
ITGB1	ITGA2
COL1A1	ITGA2
ITGB1	ITGA11
ITGB1	ITGA10
TLN1	ITGA1
MATN1	ITGA1
ITGB1	ITGA1
UBC	ITFG3
TNK2	ITFG2
UBC	ITFG1
UBE2L3	ITCH
UBC	ITCH
TP63	ITCH
TP53	ITCH
ITCH	HNRNPUL1
ERBB4	ITCH
ITCH	DTX1
ITCH	CXCR4
UBC	IST1
CHMP1B	IST1
UBC	ISOC2
PDK2	ISOC2
EML1	ISG20L2
UBC	ISCU
RARA	IRX4
YWHAE	IRS2
UBC	IRS2
IRS2	TYK2
IRS2	SIRT1
IRS2	JAK3
IRS2	JAK1
IRS2	BCL2L1
YWHAE	IRS1

UBC	IRS1
IRS1	TYK2
IRS1	TUB
IRS1	RPTOR
PTPRF	IRS1
PRKCD	IRS1
IRS1	PIK3R2
IRS1	MAPK9
IRS1	JAK3
IRS1	JAK1
EGFR	IRS1
CRK	IRS1
IRS1	AP3S1
IRS1	ALK
STAT2	IRF9
IRF1	IRF9
IFI6	IRF9
UBC	IRF8
SPI1	IRF8
NCF2	IRF8
IRF8	IRF1
IL1B	IRF8
IRF8	IL12A
COPS2	IRF8
CIITA	IRF8
UBC	IRF7
TRAF6	IRF7
MYD88	IRF7
IRF5	IRF7
IRF3	IRF7
IRF1	IRF7
IKBKE	IRF7
IFNB1	IRF7
IFNA14	IRF7
IFNA1	IRF7
UBC	IRF5
TBK1	IRF5
IRF3	IRF5
IL12A	IRF5
IKBKE	IRF5
IRF5	GPS1
TRAF2	IRF4
SPIB	IRF4
SPI1	IRF4

NFKB1	IRF4
NFATC2	IRF4
IL1B	IRF4
FCER2	IRF4
CIITA	IRF4
BCL6	IRF4
UBC	IRF3
TBK1	IRF3
IRF3	IFNB1
IRF3	IFNA1
IRF3	IFIT1
CCL5	IRF3
UBC	IRF2BP1
SMAD1	IRF2BP1
SUMO2	IRF2
RELA	IRF2
KAT2B	IRF2
IRF8	IRF2
IRF2BP2	IRF2
IRF2BP1	IRF2
IFNB1	IRF2
CIITA	IRF2
UBC	IRF1
TRIM28	IRF1
STUB1	IRF1
STAT1	IRF1
SPI1	IRF1
NFKB1	IRF1
KAT2B	IRF1
IRF1	IL12A
IFNB1	IRF1
IRF1	IFNA1
HMGA1	IRF1
CIITA	IRF1
YBX1	IREB2
IREB2	FBXL5
MYD88	IRAK4
TRAF6	IRAK3
IRAK2	IRAK3
TRAF6	IRAK2
TIRAP	IRAK2
SMAD2	IRAK2
MYD88	IRAK2
IRAK2	IL1R1

HRAS	IRAK2
UBC	IQGAP3
UBC	IQGAP1
S100B	IQGAP1
NFKB2	IQGAP1
KDR	IQGAP1
IQGAP1	CLIP1
CDC42	IQGAP1
ACTB	IQGAP1
UBC	IQCB1
UBC	IPP
UBC	IPO9
TNFRSF1A	IPO8
UBC	IPO5
TANK	IPO5
IPO5	RAG2
NUP98	IPO5
NUP214	IPO5
UBC	IPO4
HIST1H3A	IPO4
ASF1B	IPO4
IPO13	PAX6
UBC	IPO11
TRAF2	IPO11
IPO11	PLCD4
UBC	IP6K2
EXOSC7	IP6K1
UBC	INTS9
UBC	INTS8
UBC	INTS7
UBC	INTS6
INTS10	INTS6
INTS10	INTS5
UBC	INTS4
UBC	INTS3
INTS3	INTS10
SUMO2	INTS12
UBC	INTS1
INTS10	INTS1
DEAF1	INTS1
VAV1	INSR
UBC	INSR
STAT5B	INSR
SORBS1	INSR

SOCS6 INSR
SOCS3 INSR
SOCS1 INSR
SNX1 INSR
SH2B2 INSR
SH2B1 INSR
RASA1 INSR
PTPRF INSR
PTPN6 INSR
PTPN2 INSR
PTPN11 INSR
PTPN1 INSR
PTK2 INSR
PRKCD INSR
PRKCA INSR
PLCG1 INSR
PIK3R1 INSR
MAD2L1 INSR
KHDRBS1 INSR
JAK2 INSR
JAK1 INSR
IRS2 INSR
IRS1 INSR
INSRR INSR
INS INSR
INPPL1 INSR
GRB14 INSR
GRB10 INSR
GAB1 INSR
FRS2 INSR
FABP4 INSR
CSK INSR
CEACAM1 INSR
CBL INSR
CALM1 INSR
UBC INSIG2
SCAP INSIG2
INSIG1 SCAP
IDE INS
CPE INS
ARL6IP1 INPP5K
UBC INPP5D
INPP5D SLAMF1
INPP5D IL4R

INPP5D	FCGR2B
INPP5D	DOK3
DOK2	INPP5D
UBC	INPP5A
UBC	INPP4B
SMAD1	INPP4A
CAPN1	INPP4A
LIG1	INPP1
RUVBL2	INO80E
INO80E	MCRS1
INO80E	ACTR5
INO80E	ACTL6A
INO80	INO80D
UBC	INO80C
INO80E	INO80C
INO80	INO80C
UIMC1	INO80B
INO80E	INO80B
INO80	INO80B
CD81	INO80B
UCHL5	INO80
RUVBL2	INO80
INO80	MCRS1
INO80E	INO80
INO80	ACTR5
INTS3	INIP
INHBA	INHBC
ACVR2B	INHBC
INHBC	INHBB
INHBA	INHBB
ACVR2B	INHBB
ACVR2A	INHBB
TGFBR3	INHA
INHBB	INHA
ACVR2A	INHA
ING5	NAV2
TP53	ING4
RELA	ING4
PPFIA1	ING4
KAT7	ING4
HIST3H3	ING4
EGLN1	ING4
TRRAP	ING3
RUVBL1	ING3

MORF4L1 ING3
KAT5 ING3
ACTL6A ING3
SMAD1 ING2
UBC INF2
UBC INCENP
CDCA8 INCENP
CBX5 INCENP
BIRC5 INCENP
AURKA INCENP
UBC IMPDH1
UBC IMPAD1
UBC IMPACT
UBC IMPA1
UBC IMP4
UBC IMP3
MPHOSPH10IMP3
LPXN IMP3
UBC IMMT
TXNDC9 IMMT
STX5 IMMT
IMMT STMN4
IMMT SRRT
SNAPIN IMMT
IMMT RER1
IMMT PLA2G15
IMMT PFDN1
IMMT NDUFB9
KIF22 IMMT
KDM1A IMMT
IMMT HJURP
CHD3 IMMT
CCT5 IMMT
IMMT CCDC113
IMMT C10orf88
BAG6 IMMT
ASCC2 IMMT
AKTIP IMMT
UBC ILVBL
UBC ILKAP
PDK1 ILK
ILK ITGB3
ITGB2 ILK
UBC ILF3

ILF3	ILF2
DHX9	ILF3
UBC	ILF2
NFKB2	ILF2
MAP3K14	ILF2
EEF1G	ILF2
DHX9	ILF2
UBC	IL7R
JAK3	IL7R
JAK1	IL7R
IL7R	IL7
FYN	IL7R
VAV1	IL6ST
STAT1	IL6ST
PRKCD	IL6ST
IL6ST	PIK3CG
STAT3	IL6R
JAK1	IL6R
IL6ST	IL6R
IL6R	CNTF
ZBTB16	IL6
SH3GL2	IL6
PTHLH	IL6
OSM	IL6
IL6ST	IL6
IL6R	IL6
HRH1	IL6
CEBPB	IL6
IL5RA	IL5
CSF2RB	IL5
UBC	IL4R
STAT6	IL4R
JAK3	IL4R
JAK1	IL4R
IL4R	IL4
IL36RN	SSBP4
IL36RN	SSBP2
SKIL	IL36RN
CSF1R	IL34
IL1RL1	IL33
UBC	IL32
CSF2RB	IL3
IL2RB	SHB
LCK	IL2RB

JAK3	IL2RB
STAT5B	IL2RA
STAT3	IL2RA
IL2RB	IL2RA
ICAM1	IL2RA
STAT1	IL27RA
IL17RB	IL25
IL22RA2	IL22
IL22RA1	IL22
IL10RB	IL22
JAK1	IL21R
IL24	IL20RA
IL20	IL20RA
IL19	IL20RA
NFATC2	IL2
NFATC1	IL2
JUN	IL2
IL2RB	IL2
IL2RA	IL2
IFNA1	IL2
FOXK2	IL2
FOS	IL2
CREB1	IL2
CD53	IL2
IL1R2	IL1RN
IL18	IL1RL2
IRAK4	IL1RL1
MYD88	IL1RAP
IL1RAP	IL1R2
TRAF6	IL1R1
TOLLIP	IL1R1
PIK3R1	IL1R1
MYD88	IL1R1
IL1R1	IL1RN
IL1RAP	IL1R1
IL1B	IL1R1
IL1RAP	IL1B
IL1B	IL1R2
IL1B	CASP4
CASP1	IL1B
A2M	IL1B
IL1A	S100A13
IL1RAP	IL1A
IL1A	IL1R2

IL1A IL1R1
IL1A HAX1
IL18 IL18RAP
IL1RAP IL18R1
IL18 IL18R1
TRAF6 IL17RA
TRAF3IP2 IL17RA
IL17F IL17RA
IL17A IL17RA
IL17F IL17RC
IL17RB IL17B
IL17A IL17RC
PPP1R12B IL16
PPP1R12A IL16
KCNJ15 IL16
KCNJ10 IL16
GRIN2D IL16
EFEMP2 IL16
DMRTB1 IL16
CD4IL16
CASP3 IL16
IL15 IL15RA
IL2RB IL15
IL4R IL13
JAK2 IL12RB2
IL12RB2IL12RB1
IL12RB2IL12B
IL12RB1IL23A
IL12RB1IL12B
IL12B IL23A
IRF3 IL12A
IL12A IL12B
EBI3 IL12A
IL6ST IL11RA
IL11IL11RA
IL6ST IL11
IL10RA IL10RB
IL10RB IL10
IL10RB IFNL3
IL10RB IFNL2
IL10RB IFNL1
IL10RA IL10
IKZF1 IKZF4
HDAC2 IKZF4

CHD4 IKZF4
SIN3A IKZF3
BCL2L1 IKZF3
IKZF1 SIN3B
SIN3A IKZF1
IKZF3 IKZF1
IKZF1 IKZF2
HDAC2 IKZF1
CTBP1 IKZF1
CHD4 IKZF1
IKZF1 AP2M1
UBC IKBKE
TUBB IKBKE
TRAF2 IKBKE
TNFAIP3 IKBKE
IKBKE TBKBP1
TBK1 IKBKE
TANK IKBKE
RUVBL1 IKBKE
IKBKE PFKL
NUDT21IKBKE
NFKBIAIKBKE
IKBKE MYL6
MYH9 IKBKE
MYH10 IKBKE
IKBKE LGALS3BP
IKBKE IRF3
HSPA8 IKBKE
HNRNPH1 IKBKE
IKBKE FKBP5
DSPIKBKE
IKBKE CDC37
IKBKE AZI2
UBC IKBKB
TUBB4A IKBKB
IKBKB TUBB2B
TUBB IKBKB
TRAF3IP2 IKBKB
TRAF2 IKBKB
TRAF1 IKBKB
TP53 IKBKB
TNFRSF1A IKBKB
TNFAIP3 IKBKB
TNFIKBKB

TIMM50	IKBKB
TGFBR1	IKBKB
TANK	IKBKB
SMARCC1	IKBKB
RIPK1	IKBKB
IKBKB	RELA
IKBKB	PRKCQ
PPP1CA	IKBKB
PARP1	IKBKB
IKBKB	NFKBIB
NFKBIA	IKBKB
NFKB2	IKBKB
NFKB1	IKBKB
NCOA3	IKBKB
MYH10	IKBKB
MAVS	IKBKB
MAP3K7	IKBKB
MAP3K14	IKBKB
IKBKB	MAP3K11
IKBKB	LYZ
IKBKB	KEAP1
IRS1	IKBKB
IKBKB	IQGAP1
IKBKE	IKBKB
IKBKAP	IKBKB
HSPA9	IKBKB
HSPA8	IKBKB
HSPA5	IKBKB
HSPA1L	IKBKB
IKBKB	HSP90AB1
HSP90AA1	IKBKB
FOXO3	IKBKB
FANCA	IKBKB
ERC1	IKBKB
EIF2AK2	IKBKB
IKBKB	EGLN3
CTNNB1	IKBKB
COPS5	IKBKB
IKBKB	COPS3
IKBKB	CNTN2
CLTC	IKBKB
CHUK	IKBKB
IKBKB	CFLAR
CDC37	IKBKB

IKBKB CASP8
CALM1 IKBKB
C1QBP IKBKB
BCL10 IKBKB
ACVR1 IKBKB
UBC IKBKAP
MAP3K14 IKBKAP
ELP2 IKBKAP
CHUK IKBKAP
BAG6 IKBKAP
UBC IKBIP
THOC1 IK
PTCH2 IHH
ASCC2 IGSF9
UBC IGSF8
CD9IGSF8
IGSF21 UBE2V2
SULT1E1 IGSF21
SERPINB9 IGSF21
SEPHS1 IGSF21
IGSF21 RPP14
PSMD11 IGSF21
PRMT1 IGSF21
PAEP IGSF21
MAD2L1BP IGSF21
GSTM4 IGSF21
ETHE1 IGSF21
ERH IGSF21
BTBD2 IGSF21
ANXA3 IGSF21
VEGFA IGFBP7
SDC1 IGFBP7
INS IGFBP7
IGF2 IGFBP7
IGF1 IGFBP7
CCL5 IGFBP7
CCL21 IGFBP7
IGFBP6 IGF1
IGFBP5 VTN
IGFBP5 TF
SPP1 IGFBP5
IGFBP5 IGF2
IGFBP5 IGF1
BAG6 IGFBP5

ADAM12 IGFBP5
TF IGFBP4
PAPPA IGFBP4
IGF2 IGFBP4
IGF1 IGFBP4
TF IGFBP3
RXRA IGFBP3
POLA2 IGFBP3
PLGIGFBP3
IGFBP3 PAPPA2
IGFBP3 IGFALS
IGF2 IGFBP3
IGFBP3 IGF1
FN1 IGFBP3
COL1A1 IGFBP3
ADAM12 IGFBP3
IGFBP2 IGF1
TF IGFBP1
ITGA5 IGFBP1
IGF2 IGFBP1
IGF1 IGFBP1
PDCD6 IGFALS
IGFBP5 IGFALS
IGF1 IGFALS
UBC IGF2R
PLIN3 IGF2R
PACS1 IGF2R
IGF2 IGF2R
GGA3 IGF2R
GGA2 IGF2R
GGA1 IGF2R
CREG1 IGF2R
AGA IGF2R
UBC IGF2BP3
UBC IGF2BP2
UBC IGF2BP1
VTN IGF2
IGFBP6 IGF2
IGFBP2 IGF2
IDE IGF2
YWHAG IGF1R
YWHAE IGF1R
YWHAB IGF1R
IGF1R VAV3

UBC	IGF1R
IGF1R	SOCS2
IGF1R	SOCS1
RASA1	IGF1R
PTPN11	IGF1R
PTPN1	IGF1R
IGF1R	PIK3R3
IGF1R	PIK3R2
PIK3R1	IGF1R
NEDD4	IGF1R
MDM2	IGF1R
JAK2	IGF1R
JAK1	IGF1R
ITGB1	IGF1R
IRS2	IGF1R
IRS1	IGF1R
IGF1R	INS
IGF1R	IGFBP3
IGF1R	IGF2
IGF1R	IGF1
IGF1R	GRB10
GNB2L1	IGF1R
IGF1R	GNAI2
IGF1R	GNAI1
ESR1	IGF1R
EGFR	IGF1R
CRK	IGF1R
ARRB2	IGF1R
IGF1R	ARHGEF12
IFT57	GSTK1
HSPB11	IFT27
ABI2	IFT20
UBC	IFT140
UBC	IFT122
IFNAR1	IFNW1
IFNLR1	IFNL1
IFNGR2	IFNGR1
UBC	IFNGR1
STAT1	IFNGR1
JAK1	IFNGR1
PDIA3	IFNG
IFNG	IFNGR2
IFNG	IFNGR1
CREB1	IFNG

IFNAR1	IFNB1
STAT1	IFNAR2
JAK1	IFNAR2
IFNAR2	IFNB1
IFNAR1	IFNAR2
IFNAR2	IFNA8
GNB2L1	IFNAR2
IFNAR1	AP2M1
IFNAR1	IFNA8
IFNAR2	IFNA5
IRF7	IFNA2
IRF3	IFNA2
IFNAR2	IFNA2
IFNAR1	IFNA2
IRF3	IFNA14
UBC	IFITM3
IFIT5	IFIT3
IFIT1	IFIT3
IFIT3	IFIT2
IRF1	IFI6
UBC	IFI44
CDK4	IFI27
UBC	IFI16
TP53BP1	IFI16
TP53	IFI16
UBC	IFFO2
UBC	IFFO1
UBC	IER5L
UBC	IER3IP1
UBC	IER2
MPP3	IDUA
DDX24	IDO1
UBC	IDI1
UBC	IDH3B
UBC	IDH3A
PSMD4	IDH3A
UBC	IDH1
SREBF1	ID3
UNC119	ID2
UBC	ID2
TCF3	ID2
ID2	TCF12
RBM48	ID2
ID2	RBL2

MYOD1 ID2
ID2 MYF6
ID2 MYF5
ID2 MSC
CDK2 ID2
MRPS28 ICT1
MRPS18B ICT1
MRPL55 ICT1
MRPL51 ICT1
MRPL41 ICT1
MRPL4 ICT1
MRPL39 ICT1
MRPL3 ICT1
MRPL23 ICT1
MRPL13 ICT1
MRPL12 ICT1
MRPL10 ICT1
KARS ICT1
DAP3 ICT1
CLPX ICT1
ICOS ICOSLG
SMAD1 ICK
MAPK1 ICK
ITGB2 ICAM4
ITGB1 ICAM4
ZBTB16 ICAM3
ITGB2 ICAM3
ITGAL ICAM3
EZRICAM3
CD209 ICAM3
ITGB2 ICAM2
ICAM2 ITGAM
ITGB2 ICAM1
UBC IBA57
UBC IARS2
UBC IARS
TANK IARS
NFKB2 IARS
IARS MAP3K14
NAA15 HYPK
UBC HYOU1
C14orf1 HYLS1
UBC HYI
UBC HUS1

RAD1 HUS1
HDAC1 HUS1
RABEP1 HUNK
HTT ZDHHC17
TUBB HTT
HAP1 HTT
UBC HTRA2
HTRA2 CSN2
HTRA2 BIRC6
HTRA2 BIRC2
FAM46A HTRA1
GNAQ HTR2B
JAK2 HTR2A
GNAQ HTR2A
GNA11 HTR2A
S1PR1 HTR1D
HTR1B HTR1D
HTR1A HTR1D
HTR1A HTR1B
UBC HTATIP2
PAFAH1B1 HSPH1
ILK HSPH1
TTR HSPG2
SPARC HSPG2
NID1 HSPG2
HSPG2 LAMA1
ITGB1 HSPG2
HSPG2 GRN
HSPG2 FGFBP1
HSPG2 FBN1
HSPG2 FBLN2
HSPG2 COL13A1
UBC HSPD1
HSPD1 SIRT3
RAD52 HSPD1
HSPD1 PRNP
HSPD1 MPST
ITGB1 HSPD1
HSPE1 HSPD1
HSPA1A HSPD1
HSP90AA1 HSPD1
FHIT HSPD1
CASP3 HSPD1
CA2 HSPD1

BAK1	HSPD1
APP	HSPD1
UBC	HSPBP1
STUB1	HSPBP1
HSPA8	HSPBP1
HSPA2	HSPBP1
HSPA1A	HSPBP1
PTN	HSPBAP1
BAG3	HSPB8
HSPB8	HSPB7
HSPB3	RBM48
HSPB3	OLFML3
HSPB3	MED8
UBC	HSPB1
TP53	HSPB1
SNCA	HSPB1
RBM48	HSPB1
PRKD1	HSPB1
PPA1	HSPB1
MYC	HSPB1
HSPB1	MED31
HSPB1	MAPKAPK5
HSPB1	MAPKAPK3
HSPB1	MAPKAPK2
HSPB1	IGSF21
HSPB1	HSPB8
HSPB1	HNRNPD
ESR1	HSPB1
EIF4G1	HSPB1
DAXX	HSPB1
HSPB1	CRYGC
HSPB1	CRYBB2
HSPB1	CRYAA
CFTR	HSPB1
YWHAG	HSPA9
UBC	HSPA9
TRAF6	HSPA9
TRAF2	HSPA9
HSPA9	TRAF1
TP53	HSPA9
TNFRSF1B	HSPA9
TNFRSF1A	HSPA9
TBK1	HSPA9
HSPA9	TANK

TAB2	HSPA9
TAB1	HSPA9
SUMO2	HSPA9
RIPK3	HSPA9
HSPA9	RIPK1
NFKB2	HSPA9
HSPA9	NFKB1
MAP3K7	HSPA9
HSPA9	MAP3K14
HSPA9	IKBKE
HSPA9	HSP90B1
HSPA9	FGF1
HSPA8	VCL
HSPA8	UCHL1
HSPA8	TRAF1
TP53	HSPA8
HSPA8	TNFRSF1A
HSPA8	TBK1
HSPA8	TANK
HSPA8	STIP1
HSPA8	NFKB2
HSPA8	MAPT
HSPA8	HTT
HSPA8	APOB
TNFRSF1A	HSPA6
UBC	HSPA5
TRAF6	HSPA5
TRAF2	HSPA5
HSPA5	TNFRSF1B
HSPA5	TNFRSF1A
HSPA5	TBK1
HSPA5	TANK
HSPA5	TAB2
HSPA5	STMN1
PXN	HSPA5
HSPA5	PPIB
HSPA5	OS9
HSPA5	NFKB2
HSPA5	MAP3K14
HSPA5	LCT
HSPA5	HSPA1L
HSPA1A	HSPA5
HSPA5	GRIA1
HSPA5	ERLEC1

EGFR	HSPA5
HSPA5	DPYSL5
HSPA5	DPH1
HSPA5	DDX24
HSPA5	APOB
UBC	HSPA4L
TAB1	HSPA4L
VCP	HSPA4
UBE3A	HSPA4
UBC	HSPA4
TRAF6	HSPA4
TRAF2	HSPA4
TP53	HSPA4
HSPA4	TOX4
TAB1	HSPA4
STUB1	HSPA4
STIP1	HSPA4
HSPA4	SOD1
SNCA	HSPA4
SMAD3	HSPA4
HSPA4	SGTA
HSPA4	RICTOR
HSPA4	RELA
HSPA4	REL
HSPA4	PRSS23
HSPA4	PINK1
HSPA4	PARK7
PARK2	HSPA4
HSPA4	NR3C2
NFKBIA	HSPA4
HSPA4	MRGBP
MCL1	HSPA4
MAPT	HSPA4
MAP3K3	HSPA4
HSPA4	HSPBP1
HSPA8	HSPA4
HSPA4	HSP90AA1
HSPA4	HNRNPD
HSPA4	HIF1A
HAP1	HSPA4
HSPA4	GZMB
GSK3B	HSPA4
HSPA4	FANCC
FAF1	HSPA4

ESR1	HSPA4
EGFR	HSPA4
CUL5	HSPA4
CUL3	HSPA4
CUL1	HSPA4
CFTR	HSPA4
CDK5RAP3	HSPA4
HSPA4	BAG5
HSPA4	BAG3
APAF1	HSPA4
ABL1	HSPA4
UBC	HSPA2
TAB2	HSPA2
UBC	HSPA1L
TRAF2	HSPA1L
TRAF1	HSPA1L
TNFRSF1B	HSPA1L
TNFRSF1A	HSPA1L
TANK	HSPA1L
NFKB2	HSPA1L
NFKB1	HSPA1L
MAP3K14	HSPA1L
UBC	HSPA1A
TP53	HSPA1A
HSPA1A	TOMM70A
SUMO1	HSPA1A
HSPA1A	STUB1
HSPA1A	STIP1
HSPA1A	NQO1
HSPA1A	NFKB1
HSPA1A	MAPT
MAP3K5	HSPA1A
HSPA1A	KRT7
HSPA8	HSPA1A
HSPA1A	HSPA6
HSPA1A	FANCC
HSPA1A	DNAJB11
HSPA1A	DNAJA2
HSPA1A	BAG4
HSPA1A	BAG3
HSPA1A	BAG2
HSPA1A	APOB
HSPA1A	APAF1
UBC	HSPA14

STIP1	HSPA14
HSPA13	UBQLN1
NRCAM	HSPA12A
UBC	HSP90B1
TNFRSF1B	HSP90B1
HSP90B1	FANCC
EGFR	HSP90B1
APOB	HSP90B1
UBC	HSP90AB1
TRAF2	HSP90AB1
HSP90AB1	TP53RK
TP53	HSP90AB1
TBK1	HSP90AB1
TAB1	HSP90AB1
SUMO2	HSP90AB1
SUMO1	HSP90AB1
STUB1	HSP90AB1
STIP1	HSP90AB1
STAT3	HSP90AB1
RPS6KA1	HSP90AB1
RPS6	HSP90AB1
RPAP3	HSP90AB1
RIPK3	HSP90AB1
RIPK1	HSP90AB1
RELA	HSP90AB1
RAF1	HSP90AB1
PRKCE	HSP90AB1
PRKAA1	HSP90AB1
HSP90AB1	PPID
PINK1	HSP90AB1
PHB	HSP90AB1
HSP90AB1	NOS3
NFKB2	HSP90AB1
NFKB1	HSP90AB1
MYH9	HSP90AB1
MCM5	HSP90AB1
MAP3K8	HSP90AB1
MAP3K7	HSP90AB1
MAP3K14	HSP90AB1
ILK	HSP90AB1
IKBKE	HSP90AB1
HSPA1A	HSP90AB1
HSP90AA1	HSP90AB1
HSP90AB1	GRK6

HSP90AB1	FKBP4
ESR1	HSP90AB1
EGFR	HSP90AB1
DDX24	HSP90AB1
CUL2	HSP90AB1
CUL1	HSP90AB1
CSNK1E	HSP90AB1
CHUK	HSP90AB1
CDK7	HSP90AB1
CDK4	HSP90AB1
CDC37	HSP90AB1
BRAF	HSP90AB1
BAG3	HSP90AB1
AKT2	HSP90AB1
HSP90AB1	AHSA1
ZNF512B	HSP90AA1
YWHAE	HSP90AA1
VCP	HSP90AA1
UCHL1	HSP90AA1
UBC	HSP90AA1
TUBA1A	HSP90AA1
TRAF2	HSP90AA1
TRAF1	HSP90AA1
TP53	HSP90AA1
HSP90AA1	TOMM34
TNK2	HSP90AA1
TNFRSF1A	HSP90AA1
TGFBR2	HSP90AA1
TERT	HSP90AA1
TBK1	HSP90AA1
TAB2	HSP90AA1
TAB1	HSP90AA1
SUMO1	HSP90AA1
HSP90AA1	SUGT1
STUB1	HSP90AA1
STIP1	HSP90AA1
HSP90AA1	STAT3
SP1	HSP90AA1
RPS6KA1	HSP90AA1
RPS6	HSP90AA1
RPS3A	HSP90AA1
HSP90AA1	RPL7
RPAP3	HSP90AA1
RIPK1	HSP90AA1

RAF1	HSP90AA1
PRKACB	HSP90AA1
PRKAA1	HSP90AA1
HSP90AA1	PPARD
HSP90AA1	PPARA
POLR2E	HSP90AA1
HSP90AA1	PINK1
PGR	HSP90AA1
PARK2	HSP90AA1
PAFAH1B1	HSP90AA1
NUDC	HSP90AA1
HSP90AA1	NTRK1
HSP90AA1	NOS3
NOS1	HSP90AA1
HSP90AA1	NOD1
NFKB2	HSP90AA1
MDM2	HSP90AA1
HSP90AA1	MAPK1
MAP3K14	HSP90AA1
LCK	HSP90AA1
KCNH2	HSP90AA1
HSP90AA1	IRF3
ILK	HSP90AA1
IKBKE	HSP90AA1
HSPA8	HSP90AA1
HSPA1A	HSP90AA1
GSK3B	HSP90AA1
HSP90AA1	GRK6
HSP90AA1	GNA12
FYN	HSP90AA1
FKBPL	HSP90AA1
FKBP8	HSP90AA1
HSP90AA1	FKBP5
HSP90AA1	FKBP4
HSP90AA1	FES
HSP90AA1	FBXO6
FASN	HSP90AA1
HSP90AA1	EIF2AK1
EGFR	HSP90AA1
DNAJC7	HSP90AA1
CUL2	HSP90AA1
CUL1	HSP90AA1
CHUK	HSP90AA1
HSP90AA1	CHORDC1

CFTR	HSP90AA1
CDK4	HSP90AA1
HSP90AA1	CDC37
AXL	HSP90AA1
APAF1	HSP90AA1
HSP90AA1	ALK
HSP90AA1	AHSA1
YWHAE	HSF1
XRCC6	HSF1
UBC	HSF1
TBP	HSF1
TAF9	HSF1
SYMPK	HSF1
SUMO3	HSF1
SUMO2	HSF1
SUMO1	HSF1
SUB1	HSF1
STUB1	HSF1
STAT1	HSF1
SMARCA4	HSF1
PTGES3	HSF1
NCOA6	HSF1
MAPK9	HSF1
MAPK3	HSF1
HSPA8	HSF1
HSPA4	HSF1
HSPA1A	HSF1
HSP90AA1	HSF1
HSF2	HSF1
HDAC2	HSF1
HDAC1	HSF1
GTF2A2	HSF1
FKBP4	HSF1
CREBBP	HSF1
CEBPB	HSF1
CDC20	HSF1
UBC	HSDL2
UBC	HSDL1
UBC	HSD17B7
UBC	HSD17B4
PEX5	HSD17B4
UBC	HSD17B12
UBC	HSD17B11
CTBP1	HSD11B2

CCDC53	HSBP1
UBC	HRNR
PLGHRG	
HRG	C1QB
HRG	C1QA
ZHX2	HRAS
UBC	HRAS
TIAM1	HRAS
HRAS	SOS2
RASSF5	HRAS
HRAS	PIK3CG
PIK3CD	HRAS
HRAS	PDE6D
FYN	HRAS
CDC25A	HRAS
UBC	HPS6
MARK4	HPS6
KIAA0020	HPS6
UBC	HPS5
HPS1	HPS4
UBC	HPD
DTX2	HPCAL4
UBC	HPCAL1
HMG20A	HPCAL1
UBC	HPCA
UBC	HP1BP3
PKNOX1	HOXD9
UBC	HOXD13
MAFF	HOXD12
MEIS1	HOXD11
SMAD1	HOXC8
UBC	HOXC6
PKNOX1	HOXB7
NFKBIA	HOXB7
CREBBP	HOXB7
PLSCR1	HOXB6
PKNOX1	HOXB6
KRT15	HOXB6
CREBBP	HOXB6
HOXB1	PKNOX1
PAX6	HOXB1
PTPN6	HOXA10
HOXA1	TRAPPC6A
HOXA1	PKNOX1

LPXN	HOXA1
EFEMP2	HOXA1
HOXA1	CHIC2
ZNF638	HOOK2
ZNF250	HOOK2
USP2	HOOK2
RIBC2	HOOK2
RALBP1	HOOK2
MCM10	HOOK2
KIAA0408	HOOK2
KAT7	HOOK2
FAM107A	HOOK2
EPS8	HOOK2
CCDC33	HOOK2
AKTIP	HOOK2
ABLIM1	HOOK2
ZNF250	HOOK1
UBC	HOOK1
HOMER1	HOMER3
GRIA1	HOMER1
ABI3	HOMER1
UBC	HNRNPUL2
UBC	HNRNPUL1
NXF1	HNRNPUL1
NEDD4	HNRNPUL1
C14orf1	HNRNPUL1
YBX1	HNRNPU
WBP4	HNRNPU
UBC	HNRNPU
TNFRSF1B	HNRNPU
TIAL1	HNRNPU
TCERG1	HNRNPU
TANK	HNRNPU
SYNCRIP	HNRNPU
SUMO1	HNRNPU
SND1	HNRNPU
RPS6	HNRNPU
RFC4	HNRNPU
RELB	HNRNPU
RBM39	HNRNPU
PTBP1	HNRNPU
PRPF40A	HNRNPU
POLR2A	HNRNPU
PLCG1	HNRNPU

NR3C1	HNRNPU
NPM1	HNRNPU
NFKB2	HNRNPU
NEDD4	HNRNPU
NDN	HNRNPU
MAP3K14	HNRNPU
IKBKB	HNRNPU
HNRNPA1	HNRNPU
GTF2H1	HNRNPU
GTF2F1	HNRNPU
GAS7	HNRNPU
EIF4A3	HNRNPU
DHX9	HNRNPU
CR2	HNRNPU
CDK7	HNRNPU
UBC	HNRNPR
PRMT1	HNRNPR
HNRNPA1	HNRNPR
FARSA	HNRNPR
UBD	HNRNPM
UBC	HNRNPM
TCERG1	HNRNPM
TBK1	HNRNPM
TANK	HNRNPM
SUMO2	HNRNPM
SUMO1	HNRNPM
SRRM2	HNRNPM
RPL19	HNRNPM
RELA	HNRNPM
RBM4B	HNRNPM
PXN	HNRNPM
NFKB2	HNRNPM
NFKB1	HNRNPM
MAP3K14	HNRNPM
MAP3K1	HNRNPM
LYST	HNRNPM
LMO3	HNRNPM
HNRNPA1	HNRNPM
ESR1	HNRNPM
DDX1	HNRNPM
UBC	HNRNPL
SUMO1	HNRNPL
PTBP1	HNRNPL
PCBP2	HNRNPL

HNRNPA2B1 HNRNPL
HNRNPA1 HNRNPL
YBX1 HNRNPK
VAV1 HNRNPK
UBE2I HNRNPK
UBD HNRNPK
UBC HNRNPK
U2AF2 HNRNPK
TP53 HNRNPK
SYNCRIP HNRNPK
SUMO2 HNRNPK
SUMO1 HNRNPK
SRSF3 HNRNPK
SAFB HNRNPK
HNRNPK RBM7
HNRNPK RBM42
PTBP1 HNRNPK
PRMT1 HNRNPK
PCBP2 HNRNPK
PABPC1 HNRNPK
NCK2 HNRNPK
MDM2 HNRNPK
HNRNPK MATR3
MAPK10 HNRNPK
HNRNPK KHDRBS2
KHDRBS1 HNRNPK
ITK HNRNPK
HNRNPL HNRNPK
HNRNPA1 HNRNPK
HNRNPA0 HNRNPK
GRB2 HNRNPK
GRAP2 HNRNPK
ESR1 HNRNPK
HNRNPK DDX1
CNNM3 HNRNPK
CEBPB HNRNPK
VCL HNRNPH3
UBC HNRNPH3
HNRNPH3 STK24
HNRNPH3 HNRNPUL1
HNRNPH1 HNRNPH3
HNRNPA1 HNRNPH3
EEF1G HNRNPH3
C14orf1 HNRNPH3

HNRNPH1	VCL
UBC	HNRNPH1
HNRNPH1	TAB2
PIN1	HNRNPH1
HNRNPH1	KHSRP
HNRNPA1	HNRNPH1
HNRNPH1	DDX5
UBC	HNRNPF
SUMO1	HNRNPF
NFKB1	HNRNPF
HNRNPF	NCBP2
HNRNPF	HNRNPUL1
HNRNPH3	HNRNPF
HNRNPH1	HNRNPF
HNRNPF	HNRNPD
HNRNPF	HNRNPA0
VHL	HNRNPD
UBC	HNRNPD
PTBP1	HNRNPD
LSM5	HNRNPD
HSPA8	HNRNPD
HNRNPA1	HNRNPD
EIF4G1	HNRNPD
HNRNPC	RALYL
HNRNPC	PIN1
HNRNPC	NFKB2
HNRNPC	LMO3
HNRNPC	HNRNPA1
HNRNPC	DHX9
CCDC85B	HNRNPC
HNRNPC	AP1M1
UBC	HNRNPAB
TP63	HNRNPAB
YBX1	HNRNPA2B1
VHL	HNRNPA2B1
UBC	HNRNPA2B1
SNRNP70	HNRNPA2B1
PTBP1	HNRNPA2B1
HNRNPH1	HNRNPA2B1
HNRNPA1	HNRNPA2B1
UBC	HNRNPA1L2
HNRNPA1	DHX9
HNRNPA1	DDX5
HNRNPA0	DDX5

TP53	HNF4A
SUB1	HNF4A
PPARGC1A	HNF4A
NR2C2	HNF4A
NCOA6	HNF4A
MED1	HNF4A
TLE1	HNF1A
STAT3	HNF1A
KAT2B	HNF1A
HNF4A	HNF1A
FOS	HNF1A
HNF1A	DYRK1B
ALB	HNF1A
UBC	HN1L
UBC	HMOX2
SETDB1	HMOX2
MED31	HMOX2
HAP1	HMOX2
EEF1G	HMOX2
COPS6	HMOX2
UBC	HMOX1
NOS1	HMOX1
UBC	HMGN3
UBC	HMGN2
RPS6KA4	HMGN1
PRKCA	HMGN1
HMGN1	HMGN2
UBC	HMGCS2
UBC	HMGCS1
INSIG1	HMGCR
HMGCR	ERLIN2
MS4A7	HMGCL
UNC119	HMGB1
UBC	HMGB1
TP73	HMGB1
TP53	HMGB1
TLE1	HMGB1
TBP	HMGB1
RB1	HMGB1
PLG	HMGB1
PLAT	HMGB1
NCAN	HMGB1
HOXD9	HMGB1
HNRNPK	HMGB1

CSNK1A1	HMGB1
CRMP1	HMGB1
C14orf1	HMGB1
AGER	HMGB1
AESHMGB1
PXN	HMGA2
NPM1	HMGA2
UBC	HMGA1
SUFU	HMGA1
PPARG	HMGA1
HMG20A	TESC
HMG20A	RBM7
HDAC2	HMG20A
DTNB	HMG20A
CCNK	HMG20A
UBE2N	HLTF
UBC	HLTF
HLTF	RAD18
HLFJUND
HLFBATF3
HLA-G	LILRB1
UBC	HLA-F
LILRB1	HLA-F
CALR	HLA-F
UBC	HLA-E
KLRD1	HLA-E
CD8A	HLA-E
CALR	HLA-E
B2M	HLA-E
UBC	HLA-DRB1
HLA-DRA	HLA-DRB1
UBC	HLA-DRA
HSP90B1	HLA-DRA
CD74	HLA-DRA
HLA-DRA	CD63
HLA-DRA	CD1D
HLA-DQB1	HLA-DQA2
HLA-DPB1	HLA-DPA1
HLA-DRA	HLA-DMB
HLA-DMA	HLA-DMB
CD74	HLA-DMB
CD63	HLA-DMB
HLA-DRA	HLA-DMA
HLA-DMA	HLA-DOA

CD74	HLA-DMA
UBC	HLA-C
KIR2DL3	HLA-C
UBC	HLA-A
TAPBP	HLA-A
TAP1	HLA-A
HLA-A	PDIA3
HLA-A	LILRB2
HLA-A	LILRB1
DERL1	HLA-A
HLA-A	CD8A
HLA-A	B2M
HK3	HPGDS
UBQLN1	HK2
UBC	HK2
UBC	HJURP
STT3A	HJURP
HIST4H4	HJURP
CENPA	HJURP
UBC	HIVEP3
VCL	HIVEP1
UBC	HIVEP1
SMAD3	HIVEP1
ATXN1	HIVEP1
UBC	HIST4H4
TRIM24	HIST4H4
HIST4H4	RBBP4
PRMT5	HIST4H4
PRMT1	HIST4H4
NPM1	HIST4H4
NCOR2	HIST4H4
KAT7	HIST4H4
HIST4H4	KAT6A
KAT5	HIST4H4
HIST4H4	KAT2B
HIST4H4	HIST3H3
HIST4H4	HIRA
HIST4H4	H3F3A
HIST4H4	GNB2L1
HIST4H4	DAXX
CHAF1A	HIST4H4
HIST4H4	ASF1B
HIST3H3	UHRF1
UBC	HIST3H3

TNFRSF1A	HIST3H3
HIST3H3	TAF3
HIST3H3	RBBP4
PRKCA	HIST3H3
HIST3H3	DNTTIP2
HIST3H3	ASF1B
UBC	HIST3H2BB
UBC	HIST3H2A
UHRF1	HIST2H3C
UBC	HIST2H3C
KDM2A	HIST2H3C
KDM1A	HIST2H3C
KAT5	HIST2H3C
KAT2B	HIST2H3C
ING1	HIST2H3C
EHMT2	HIST2H3C
UBC	HIST2H2BE
UBR2	HIST2H2AC
UBC	HIST2H2AC
TAF1B	HIST2H2AC
RNF8	HIST2H2AC
RNF2	HIST2H2AC
RNF168	HIST2H2AC
RBBP4	HIST2H2AC
HIST4H4	HIST2H2AC
HIST1H3A	HIST2H2AC
HIST1H2BA	HIST2H2AC
BRCA1	HIST2H2AC
UBC	HIST2H2AB
UBC	HIST2H2AA4
YWHAE	HIST1H3A
HIST1H3A	WDR5
UBC	HIST1H3A
HIST1H3A	SET
HIST1H3A	RBBP4
PRMT5	HIST1H3A
HIST1H3A	PELP1
NPM1	HIST1H3A
HIST1H3A	KAT2B
HIST4H4	HIST1H3A
HIST1H3A	EHMT2
HIST1H3A	EHMT1
CHAF1A	HIST1H3A
HIST1H3A	CBX7

HIST1H3A	CARM1
HIST1H3A	BRD7
HIST1H3A	ASH1L
HIST1H3A	ASF1B
UBC	HIST1H2BO
TNPO1	HIST1H2BO
KPNA1	HIST1H2BO
HIRIP3	HIST1H2BO
UBC	HIST1H2BN
UBC	HIST1H2BL
CUL1	HIST1H2BL
UBC	HIST1H2BJ
UBC	HIST1H2BH
UBC	HIST1H2BB
TNFRSF1A	HIST1H2BB
NFKB2	HIST1H2BB
UBC	HIST1H2BA
UBC	HIST1H2AH
UBC	HIST1H2AE
TNFRSF1A	HIST1H2AE
SUMO1	HIST1H2AE
NEDD8	HIST1H2AE
HIST1H3A	HIST1H2AE
UBC	HIST1H2AC
UBC	HIST1H2AA
UBC	HIST1H1T
UBC	HIST1H1E
MAP3K14	HIST1H1E
UBC	HIST1H1A
PRKCD	HIST1H1A
PRKCA	HIST1H1A
CDK4	HIST1H1A
CDK2	HIST1H1A
CCNE1	HIST1H1A
CCNB1	HIST1H1A
CCNA2	HIST1H1A
HIST4H4	HIRIP3
HIST3H3	HIRIP3
HIRA	HIRIP3
ABCF3	HIRIP3
HIRA	H3F3A
HIRA	CABIN1
UBC	HIPK3
HIPK3	TGFB1I1

HIPK3	SIAH2
HIPK3	SIAH1
HIPK3	MTMR4
HIPK3	LIMS2
HIPK3	LIMD1
HIPK3	GORASP1
HIPK3	FADD
HIPK3	C1QA
HIPK3	ARRB2
UBC	HIPK2
TP63	HIPK2
TP53	HIPK2
MYB	HIPK2
MDM2	HIPK2
HIPK2	HMGA1
DDX39B	HIPK2
UBC	HIPK1
TP53	HIPK1
SKIL	HIPK1
UBC	HIP1
RPS10	HIP1
PFDN1	HIP1
IFT57	HIP1
HTT	HIP1
HIP1R	HIP1
CLTCL1	HIP1
CLTC	HIP1
AP2A1	HIP1
UBC	HINT3
TRAF2	HINFP
NPAT	HINFP
UBC	HIGD1A
UBC	HIF1AN
INVS	HIF1AN
HDAC2	HIF1AN
ASB13	HIF1AN
VHL	HIF1A
UBXN7	HIF1A
UBC	HIF1A
TP53	HIF1A
TCEB2	HIF1A
TCEB1	HIF1A
SUMO2	HIF1A
SUMO1	HIF1A

STAT3 HIF1A
SP1 HIF1A
PSMC3 HIF1A
HIF1A PSMA7
OS9 HIF1A
NCOA1 HIF1A
MDM2 HIF1A
MAPK3 HIF1A
HIF1A HSP90AB1
HSP90AA1 HIF1A
HIF1A HIF1AN
HDAC4 HIF1A
HDAC3 HIF1A
HDAC1 HIF1A
GNB2L1 HIF1A
HIF1A EGLN3
HIF1A EGLN2
HIF1A EGLN1
CXCR4 HIF1A
CUL2 HIF1A
CREBBP HIF1A
COPS5 HIF1A
ARNT HIF1A
RAD51 HID1
CBS HID1
UBE2I HIC1
SIRT1 HIC1
HIC1 CTBP2
CTBP1 HIC1
UBC HIBADH
UBC HIATL1
IHH HHIP
EFEMP2 HHEX
SDC2 HGF
CLEC3B HGF
TFRC HFE
TFR2 HFE
B2M HFE
ZNF512B HEYL
SMAD9 HEYL
SMAD7 HEYL
SMAD3 HEYL
SKIL HEYL
PPP1CA HEYL

LAPTM5 HEYL
HIPK3 HEYL
SIRT1 HEY2
HEY2 HES1
HDAC1 HEY2
SKIL HEY1
PRKD2 HEY1
CDK9 HEXIM2
UBC HEXIM1
RILPL2 HEXDC
PARK2 HEXDC
GLRX3 HEXDC
C7orf25 HEXDC
UBC HEXB
USP22 HEXA
GM2A HEXA
UBC HES1
TLE1 HES1
SIRT1 HES1
FOXG1 HES1
UBC HERPUD1
HERPUD1 STARD9
PSEN1 HERPUD1
IMPA2 HERPUD1
AMFR HERPUD1
UBC HERC6
UBC HERC5
UBC HERC3
XPAHERC2
UBE3A HERC2
UBC HERC2
SUMO1 HERC2
RNF8 HERC2
RNF11 HERC2
UBC HENMT1
UBC HELZ2
UBC HELLS
HDAC2 HELLS
RPA1 HELB
POLA2 HELB
UBC HECW2
SMAD1 HECW2
UBC HECW1
UBC HECTD3

ZRANB1 HECTD1
UBC HECTD1
UBC HEBP2
PDCD6 HEBP2
LRPPRC HEBP2
UBC HEBP1
UBC HEATR6
TNFRSF1B HEATR6
ZBTB16 HEATR2
UBC HEATR2
SUMO2 HEATR2
FEZ1 HEATR2
UBC HEATR1
ILK HEATR1
XPOT HDLBP
UBC HDLBP
SMAD4 HDLBP
TRIP13 HDHD3
UBC HDHD2
UBC HDGF
SMAD4 HDGF
NMI HDDC3
YWHAE HDAC9
SUMO1 HDAC9
HDAC4 HDAC9
CTBP1 HDAC9
BCL6 HDAC9
UBC HDAC7
HDAC7 SFN
PRKD1 HDAC7
NCOR2 HDAC7
ZBTB16 HDAC5
YY1 HDAC5
YWHAQ HDAC5
YWHAH HDAC5
YWHAG HDAC5
YWHAE HDAC5
YWHAB HDAC5
TBL1XR1 HDAC5
SMAD7 HDAC5
RUVBL2 HDAC5
RUNX2 HDAC5
PRKD1 HDAC5
PPP2R2A HDAC5

PPP2CA	HDAC5
PHB	HDAC5
NCOR2	HDAC5
NCOR1	HDAC5
MEF2D	HDAC5
MEF2A	HDAC5
HDAC4	HDAC5
HDAC3	HDAC5
GPS2	HDAC5
GABARAP	HDAC5
EEF1G	HDAC5
CAMK1	HDAC5
BCL6	HDAC5
ANKRA2	HDAC5
HDAC4	RUNX2
NCOR2	HDAC4
YY1	HDAC3
XPO1	HDAC3
UBC	HDAC3
HDAC3	THRA
TCP1	HDAC3
HDAC3	TBL1XR1
STAT3	HDAC3
HDAC3	SOX2
RUNX2	HDAC3
RUNX1T1	HDAC3
HDAC3	RELA
RBBP4	HDAC3
HDAC3	PRAM1
PPARG	HDAC3
HDAC3	PPARD
NRIP1	HDAC3
NR2C2	HDAC3
HDAC3	NR2C1
NFKBIA	HDAC3
NFKB1	HDAC3
NCOR2	HDAC3
MYC	HDAC3
KDM1A	HDAC3
HDAC3	HDAC9
HDAC7	HDAC3
HDAC4	HDAC3
HDAC1	HDAC3
GTF2IRD1	HDAC3

GTF2I	HDAC3
ESR1	HDAC3
CCT5	HDAC3
HDAC3	ANKRD12
HDAC3	ANKRD11
HDAC2	ZNF217
UBC	HDAC2
HDAC2	TWIST1
TP53	HDAC2
HDAC2	TCF3
HDAC2	TAL1
HDAC2	SUDS3
HDAC2	SRA1
HDAC2	SNW1
HDAC2	SNAI1
HDAC2	SMARCB1
HDAC2	SMARCA2
HDAC2	RREB1
HDAC2	RERE
HDAC2	RCOR3
HDAC2	RCOR2
HDAC2	RBBP4
HDAC2	RARA
HDAC2	PPARD
HDAC2	NCOR2
HDAC2	MYC
HDAC2	MTA2
HDAC2	MIER1
HDAC2	ING1
HDAC2	IFRD1
HDAC2	H2AFY
HDAC2	GTF2I
HDAC2	GSE1
HDAC2	CTBP1
HDAC2	CIR1
HDAC2	CIITA
HDAC2	CHD4
HDAC1	YY1
VHL	HDAC1
HDAC1	UHRF1
UBC	HDAC1
HDAC1	TXNIP
TRIM28	HDAC1
HDAC1	TRIM27

HDAC1	TRERF1
HDAC1	TP73
TP53	HDAC1
HDAC1	TLE1
HDAC1	TAL1
HDAC1	SUDS3
HDAC1	STAT3
HDAC1	SPI1
HDAC1	SPEN
HDAC1	SP1
HDAC1	SNAI1
HDAC1	SMARCA2
SMAD2	HDAC1
HDAC1	SIN3B
HDAC1	SIN3A
HDAC1	SFPQ
HDAC1	SAP30L
HDAC1	SAP18
HDAC1	SAP130
HDAC1	RUVBL2
HDAC1	RUNX2
HDAC1	RFC1
HDAC1	REST
HDAC1	RERE
HDAC1	RCOR3
HDAC1	RBL2
HDAC1	RBL1
HDAC1	RBBP4
HDAC1	PPARD
HDAC1	PPA1
HDAC1	PHF21A
HDAC1	PHF12
HDAC1	PHB
HDAC1	PFDN5
HDAC1	NFKBIA
HDAC1	NFKB1
HDAC1	NCOR2
HDAC1	MYC
HDAC1	MTA2
HDAC1	MORF4L1
HDAC1	MIER1
HDAC1	MECOM
HDAC1	MDM2
HDAC1	KLF11

HDAC1	KDM5B
HDAC1	ING2
HDAC1	ING1
HDAC1	IKZF1
HDAC1	HR
HDAC1	HMG20A
HDAC1	HIST4H4
HDAC1	HIF1AN
HDAC1	HELLS
HDAC1	HDAC9
HDAC2	HDAC1
HDAC1	H2AFY
HDAC1	GSE1
HDAC1	GATAD2B
HDAC1	FOXG1
HDAC1	EED
HDAC1	DNTTIP1
HDAC1	DNMT3A
HDAC1	DNMT1
HDAC1	DDX5
HDAC1	DDB2
HDAC1	DAXX
HDAC1	CTBP1
HDAC1	CREB1
HDAC1	CNTN2
HDAC1	CHD4
HDAC1	CHD1
HDAC1	CDYL
HDAC1	CCDC6
HDAC1	BRMS1L
HDAC1	BRMS1
HDAC1	BHLHE40
HDAC1	BCL6
HDAC1	BCL11B
HDAC1	BAZ2A
HDAC1	ARID4B
HCRT	HCRTR1
CNR1	HCRTR1
HCRT	HCRTR2
HCN2	HCN1
SH2D4A	HCLS1
UBC	HCK
TNK2	HCK
HCK	SKAP2

PLCG2	HCK
HCK	PLAUR
HCK	PDCD6IP
HCK	IL6ST
HCK	AGK
HCK	ABL2
UBC	HBS1L
UBC	HBP1
SMAD1	HBP1
SMAD5	HBG2
HBA1	HBG2
HBEGF	ERBB4
EGFR	HBEGF
BCL6	HBEGF
UBC	HBB
HBZ	HBB
HBA1	HBB
AHSP	HBA1
UBC	HAX1
TNFRSF1B	HAX1
TNFRSF1A	HAX1
EIF3F	HAX1
HAX1	DGKD
UBC	HAUS6
UBC	HAUS5
UBC	HAUS4
UBC	HAUS3
UBC	HAUS2
FXYD6-FXYD2	HAUS2
UBC	HAUS1
ORC2	HAUS1
MCM4	HAUS1
MCM3	HAUS1
UBC	HAT1
IPO4	HAT1
HIST4H4	HAT1
HIST3H3	HAT1
HIST1H3A	HAT1
H3F3A	HAT1
BAP1	HAT1
UBC	HARS2
UBC	HARS
TARS	HARS
RPL5	HARS

HAP1	NEUROD1
UBC	HAND2
TCF3	HAND2
PHOX2A	HAND2
HAND1	HAND2
GATA4	HAND2
COPS5	HAND2
CENPB	HAND2
GATA4	HAMP
VCL	HADHB
UBC	HADHB
RIPK2	HADHB
NFKB2	HADHB
MAP3K1	HADHB
TNFRSF1A	HADHA
NFKB2	HADHA
UBC	HADH
HACL1	ZMYND19
COL4A1	HABP2
CABIN1	H3F3A
UBC	H2AFY2
WRN	H2AFX
UBC	H2AFX
TRIM28	H2AFX
H2AFX	TP53BP1
H2AFX	SUPT16H
H2AFX	SMARCA2
H2AFX	RNF8
H2AFX	RNF168
H2AFX	PPM1D
PARP1	H2AFX
H2AFX	NBN
H2AFX	MRE11A
H2AFX	MDC1
KAT5	H2AFX
HIST4H4	H2AFX
H2AFX	HIST2H2BE
HIST1H3A	H2AFX
H2AFX	BRCA1
H2AFX	ATM
UBC	H2AFV
UBC	H2AFJ
UBC	H1FX
UBC	H1F0

RAD51B	H1F0
NOA1	H1F0
CDK2	H1F0
SET	GZMK
SET	GZMA
HMGB2	GZMA
UBC	GYS2
UBC	GYS1
PDIA3	GYPC
RHAG	GYPB
UBC	GYG1
TRIM7	GYG1
GYS1	GYG1
UBC	GXYLT1
UBC	GUSB
ITGB2	GULP1
UBC	GUK1
GUCA2B	GUCY2D
HSP90AA1	GUCY1B3
GUCY1A2	GUCY1B3
STUB1	GUCY1A3
DLG1	GUCY1A2
GUCY2C	GUCA2A
UBC	GTSF1
UBC	GTPBP4
SUMO2	GTPBP4
NECAB2	GTPBP10
GTF3C5	GTF3C6
UBC	GTF3C4
GTF3C4	GTF3C6
GTF3C5	GTF3C4
GTF3C3	GTF3C4
GTF3C2	GTF3C4
MYC	GTF3C3
GTF3C5	GTF3C2
GTF3C3	GTF3C2
USF1	GTF3C1
UBC	GTF3C1
TBC1D17	GTF3C1
SUMO2	GTF3C1
SUB1	GTF3C1
GTF3C1	NHP2L1
NFKB2	GTF3C1
MAPK10	GTF3C1

IGFBP3	GTF3C1
GTF3C4	GTF3C1
GTF3C2	GTF3C1
EZH2	GTF3C1
CPE	GTF3C1
ASCC2	GTF3C1
AKTIP	GTF3C1
OPTN	GTF3A
GTF3C2	GTF3A
UBC	GTF2IRD1
GTF2I	USF1
GTF2I	STAT1
GTF2I	NFKB2
MYC	GTF2I
UBC	GTF2H5
ERCC3	GTF2H5
UBC	GTF2H4
GTF2H3	GTF2H4
GTF2E1	GTF2H4
ERCC3	GTF2H4
XPC	GTF2H1
GTF2H1	XPA
UBC	GTF2H1
TP53	GTF2H1
PSMC2	GTF2H1
GTF2H1	GTF2H4
GTF2H3	GTF2H1
GTF2H1	GTF2E1
GTF2H1	GPN1
ESR1	GTF2H1
GTF2H1	ERCC6
GTF2H1	ERCC5
ERCC4	GTF2H1
GTF2H1	ERCC3
CDK7	GTF2H1
GTF2H1	CCNH
GTF2F2	URI1
UBC	GTF2F2
GTF2F2	POLR2J
GTF2F2	POLR2H
MSX2	GTF2F2
UBC	GTF2F1
TBP	GTF2F1
SRF	GTF2F1

PSMC2	GTF2F1
GTF2F1	POLR2J
MSX2	GTF2F1
MED21	GTF2F1
GTF2F1	GTF2F2
GTF2F1	GTF2E2
GTF2F1	GTF2E1
GTF2B	GTF2F1
FEZ1	GTF2F1
GTF2F1	CTDP1
UBC	GTF2E2
MDM2	GTF2E2
GTF2F2	GTF2E2
GTF2E2	ERCC3
TRIM24	GTF2E1
GTF2E1	TAF6
GTF2E1	GTF2F2
GTF2E1	GTF2E2
GTF2B	GTF2E1
GTF2A2	GTF2E1
GTF2E1	ERCC3
CDK7	GTF2E1
AES	GTF2E1
UBC	GTF2B
GTF2B	TAF9
GTF2B	POLR2J
GTF2B	GTF2F2
GTF2B	GTF2E2
DHX9	GTF2B
GTF2A2	GTF2A1L
TRIP4	GTF2A1
TBPL1	GTF2A1
TBP	GTF2A1
GTF2F1	GTF2A1
GTF2E2	GTF2A1
GTF2E1	GTF2A1
GTF2A2	GTF2A1
UBC	GSTP1
FANCC	GSTP1
UBC	GSTO1
RBM48	GSTO1
KAT5	GSTO1
EEF1G	GSTO1
UBC	GSTM5

UBC	GSTM4
TP53	GSTM4
UBC	GSTM3
GSTM2	GSTM3
UBC	GSTM2
GSTM1	GSTM2
UBC	GSS
UBC	GSR
VCL	GSN
VASP	GSN
UBC	GSN
GSN	TOM1L1
PXN	GSN
GSN	PTK2B
FN1	GSN
ESR1	GSN
CASP3	GSN
GSN	ACTN4
ACTG1	GSN
ACTB	GSN
ACTA1	GSN
GSK3B	GSKIP
GSK3B	STRAP
GSK3B	NIN
MYC	GSK3B
GSK3B	MUC1
MAPT	GSK3B
GSK3A	MAPT
RBM48	GSE1
UBC	GSDMD
SMAD2	GSC
UBC	GRWD1
MAP3K14	GRWD1
UBC	GRTP1
UBC	GRSF1
DPP4	GRP
SORT1	GRN
SMAD9	GRN
HOXA1	GRN
HK3	GRN
FAM131C	GRN
GNAQ	GRM7
ADRBK1	GRM1
ADORA1	GRM1

UBC	GRK6
RHO	GRK6
GRK6	RCVRN
GRK6	CHRM3
TACR1	GRK5
SNCG	GRK5
SNCA	GRK5
RHO	GRK5
RCVRN	GRK5
CHRM2	GRK5
AVPR1B	GRK5
RHO	GRK1
RCVRN	GRK1
PPFIA1	GRIP2
PRKD2	GRIP1
ERBB2IP	GRIN2C
PRKCG	GRIN2B
PRKCB	GRIN2B
PRKCA	GRIN2B
GNB2L1	GRIN2B
FYN	GRIN2B
GRIN2B	DLG4
GRIN2B	DLG2
DLG1	GRIN2B
GRIN2B	CAMK2G
GRIN2B	CAMK2D
GRIN2B	CAMK2B
GRIN2B	ARHGAP32
PTK2B	GRIN2A
FYN	GRIN2A
GRIN2A	DLGAP1
GRIN2A	DLG2
DLG1	GRIN2A
MLF1	GRIK5
LRSAM1	GRIK5
GRIK3	GRIK5
GRIK2	GRIK5
GRIK2	GRIK4
GRIP1	GRIK2
DLG1	GRIK2
UBC	GRIK1
SDCBP	GRIK1
PICK1	GRIK1
HOMER1	GRIK1

DLG4	GRIK1
PTPN4	GRID2
GRIK5	GRID2
GRIK2	GRID2
GRIA1	GRID2
GRIA4	EPB41L1
PRKCA	GRIA2
GRIP1	GRIA2
GRIA1	GRIA2
NEDD4	GRIA1
GRIA1	DLG1
UBC	GRHPR
GRB2	ZAP70
WASL	GRB2
GRB2	WASF2
GRB2	WASF1
GRB2	VAV3
VAV1	GRB2
USP8	GRB2
UBC	GRB2
GRB2	TSG101
TP63	GRB2
GRB2	TOM1L1
TNK2	GRB2
TNFRSF1A	GRB2
GRB2	TEK
GRB2	SYNJ2
GRB2	SYNJ1
GRB2	SYK
GRB2	STAMBP
GRB2	SOS2
GRB2	SIT1
GRB2	SIGLEC7
GRB2	SHB
GRB2	SH2B3
GRB2	SGCA
GRB2	SF3B4
GRB2	SELL
RBBP6	GRB2
GRB2	RAB8B
PTPN6	GRB2
GRB2	PTPN23
GRB2	PTPN22
GRB2	PTPN12

GRB2 PTK2B
GRB2 PSTPIP2
GRB2 PRKAB1
GRB2 PPP3CA
GRB2 PLCG1
GRB2 PIK3R2
PIK3R1 GRB2
GRB2 PIK3C2B
GRB2 PDGFRB
GRB2 PDGFRA
GRB2 PDE6G
GRB2 PDCD6IP
GRB2 PAK1
GRB2 NTRK1
NPM1 GRB2
GRB2 NEU3
GRB2 NCKIPSD
GRB2 MUC1
GRB2 MERTK
GRB2 MED28
GRB2 MAPK9
MAPK14 GRB2
GRB2 MAP4K5
GRB2 MAP2
GRB2 LRRK1
LCP2 GRB2
GRB2 LAX1
LAT GRB2
GRB2 KIT
GRB2 KIRREL
KHDRBS1 GRB2
GRB2 KDR
JAK2 GRB2
GRB2 JAK1
GRB2 ITK
IRS2 GRB2
IRS1 GRB2
GRB2 INPPL1
GRB2 INPP5D
HSPA5 GRB2
GRB2 GHR
GRB2 GAREM
GRB2 GAB2
GRB2 GAB1

GRB2	FLT4
GRB2	ERRFI1
ERBB3	GRB2
GRB2	EPHB1
EGFR	GRB2
GRB2	DTX1
GRB2	DRD4
GRB2	DOCK4
GRB2	DNM2
GRB2	DLGAP1
GRB2	DCTN1
DAG1	GRB2
GRB2	DAB2
GRB2	CSF3R
CSF1R	GRB2
CRK	GRB2
GRB2	CD28
GRB2	CD19
CBLB	GRB2
GRB2	BLNK
GRB2	BCL2A1
GRB2	AXL
GRB2	APP
GRB2	ANXA2
GRB2	ALOX5
GRB2	ALK
ADAM12	GRB2
GRB2	ACTG1
RET	GRB10
RAF1	GRB10
PDGFRB	GRB10
NEDD4	GRB10
GRB10	MEGF10
MAP2K1	GRB10
KIT	GRB10
JAK2	GRB10
IRS1	GRB10
GHR	GRB10
FYN	GRB10
EPHB1	GRB10
EGFR	GRB10
BCR	GRB10
ABL1	GRB10
GRAP2	SOS2

GRAP2	SHB
GRAP2	GAB1
GRAP2	BLNK
HTT	GRAP
UBC	GRAMD1A
UBC	GPX8
UBC	GPX4
UBC	GPSM2
NUMA1	GPSM2
HRAS	GPSM2
GNAI3	GPSM2
GNAI2	GPSM2
TP53	GPS2
GPS2	TBL1XR1
SETDB1	GPS2
PBK	GPS2
MAP3K7CL	GPS2
HDAC3	GPS2
CHD3	GPS2
ATF4	GPS2
UBC	GPS1
TBC1D17	GPS1
DDB2	GPS1
CUL1	GPS1
COPS7A	GPS1
COPS6	GPS1
COPS5	GPS1
COPS2	GPS1
UBC	GPRC5C
ABHD16A	GPRC5C
UBC	GPRC5A
UBC	GPR89B
HTR1A	GPR26
YWHAB	GPR15
UBC	GPR108
LRSAM1	GPR108
XPA	GPN1
UBC	GPN1
GM2A	GPLD1
ALPP	GPLD1
UBC	GPI
PFN1	GPHN
DYNLL1	GPHN
GPD1	MDH1

VEGFA GPC1
SLIT2 GPC1
SLIT1 GPC1
PLEKHF2 GPBP1L1
MCRS1 GPBP1
UBC GPATCH8
UBC GPATCH11
UBC GPANK1
CCDC85B GPANK1
GP1BB GP9
APPBP2 GP9
VWF GP1BA
F2 GP1BA
GOT2 ZDHHC6
UBC GOT2
HSPA8 GOT2
UBC GOT1
TMEM120A GOT1
USO1 GOSR2
STX5 GOSR2
YKT6 GOSR1
UBC GOSR1
STX5 GOSR1
NSF GOSR1
NAPA GOSR1
BET1L GOSR1
TSC22D4 GORASP2
TRAF2 GORASP2
TGFA GORASP2
RAD54B GORASP2
RAB2A GORASP2
RAB1A GORASP2
PCBD1 GORASP2
NUP62 GORASP2
NQO2 GORASP2
NME1 GORASP2
MIF GORASP2
MAPK1 GORASP2
CBLB GORASP2
BLZF1 GORASP2
PRKD2 GORASP1
PLK1 GORASP1
UBC GON4L
UBC GOLT1B

UBC	GOLPH3
UBC	GOLM1
SYT1	GOLM1
GOLM1	SLC40A1
GOLM1	RPL13A
NRSN2	GOLM1
MED22	GOLM1
GOLM1	LSM12
GOLM1	GRIK5
GNB2L1	GOLM1
GEMIN7	GOLM1
GOLM1	FTH1
ELAC2	GOLM1
EIF3J	GOLM1
DEXI	GOLM1
GOLM1	DCTN2
GOLM1	DCK
CLK1	GOLM1
GOLM1	CIAO1
GOLM1	C1orf174
GOLM1	B3GNT4
VTI1B	GOLGB1
USO1	GOLGB1
UBC	GOLGB1
SKIL	GOLGB1
PFN2	GOLGB1
GOLGB1	CAV2
BRMS1	GOLGB1
GOLGB1	ACBD3
UBC	GOLGA7
RAB1A	GOLGA5
UBC	GOLGA4
CASP2	GOLGA3
ZNF594	GOLGA2
ZNF250	GOLGA2
USO1	GOLGA2
UBC	GOLGA2
TRIM29	GOLGA2
TMED2	GOLGA2
STK25	GOLGA2
RAB33B	GOLGA2
RAB2A	GOLGA2
RAB1A	GOLGA2
GPS2	GOLGA2

GORASP2	GOLGA2
GORASP1	GOLGA2
CDK1	GOLGA2
CCNH	GOLGA2
ABLIM1	GOLGA2
UBC	GNS
UPF1	GNPTG
UBC	GNPNAT1
UBC	GNPDA2
UBC	GNPDA1
UBC	GNPAT
PEX5	GNPAT
PLEKHF2	GNMT
NUDT18	GNMT
ASCC2	GNL3
UBC	GNG5
GNB4	GNG5
GNB3	GNG5
GNB2	GNG5
GNB5	GNG4
GNB3	GNG4
GNB2	GNG4
GNB3	GNG3
GNB2	GNG3
GNB5	GNG2
GNB4	GNG2
GNB3	GNG2
GNB2	GNG2
GNB1	GNG2
UBC	GNG12
GNG12	GNB4
GNB2	GNG12
GNB2	GNG10
GNB1	GNG10
UBC	GNE
RGS6	GNB5
OLFM2	GNB5
EIF3J	GNB5
UBC	GNB4
GNG7	GNB4
GNB4	GNG4
GNB4	GNG3
GNB4	GNG13
GNG7	GNB3

GNB3	GNG13
VCL	GNB2L1
UBC	GNB2L1
GNB2L1	TYK2
TSC22D4	GNB2L1
GNB2L1	TP73
TP63	GNB2L1
TNFRSF1A	GNB2L1
STAT1	GNB2L1
PRKCE	GNB2L1
PRKCA	GNB2L1
GNB2L1	NSMAF
JAK1	GNB2L1
ITGB5	GNB2L1
ITGB2	GNB2L1
ITGB1	GNB2L1
GNB2L1	IL4R
IL2RB	GNB2L1
HABP4	GNB2L1
GRAP2	GNB2L1
FYN	GNB2L1
DYNLL1	GNB2L1
COPS5	GNB2L1
GNB2	GNGT2
GNG7	GNB2
UBC	GNB1
GNB1	KCNJ3
GNB1	GNGT2
GNB1	GNGT1
GNG7	GNB1
GNB1	GNG5
GNB1	GNG4
GNB1	GNG3
GNB1	GNG13
GNG12	GNB1
GNG11	GNB1
GNB2L1	GNB1
GNAS	GNB1
GNAI3	GNB1
GNAI2	GNB1
GNAI1	GNB1
ADRBK1	GNB1
UBC	GNAT2
UBC	GNAT1

RGS16	GNAT1
UBC	GNAS
ADCY5	GNAS
VIPR1	GNAQ
SLC9A3R1	GNAQ
GNAQ	S1PR2
RGS7	GNAQ
GNAQ	FFAR2
GNAQ	CHRM3
UBC	GNAO1
SCN8A	GNAO1
S1PR5	GNAO1
RGS7	GNAO1
RGS5	GNAO1
RGS4	GNAO1
RGS19	GNAO1
RGS16	GNAO1
RGS14	GNAO1
OPRD1	GNAO1
GPSM2	GNAO1
FFAR3	GNAO1
FFAR2	GNAO1
DCTN2	GNAO1
ADRA2C	GNAO1
ADRA2A	GNAO1
ADORA1	GNAO1
UBC	GNAL
UBC	GNAI3
GNAI3	S1PR1
RGS7	GNAI3
GNAI3	RGS5
RGS19	GNAI3
RGS16	GNAI3
GNAI3	RGS14
GNAI3	NUCB1
GNAI3	CNR1
CD59	GNAI3
GNAI3	CD48
UBC	GNAI2
GNAI2	RGS5
RGS19	GNAI2
RGS16	GNAI2
GNAI2	RGS14
GNAI2	P2RY12

GNAI2	NUCB1
GNAI2	LPAR3
EYA2	GNAI2
EGFR	GNAI2
GNAI2	EDNRA
GNAI2	CXCR1
GNAI2	CNR1
GNAI2	CHRM1
UBC	GNAI1
GNAI1	SSTR3
SSTR2	GNAI1
RIC8A	GNAI1
RGS7	GNAI1
RGS19	GNAI1
GNAI1	RGS18
GNAI1	RGS14
OPRM1	GNAI1
GNAI1	GPR1
GNAI1	FFAR1
CRHR1	GNAI1
GNAI1	CNR1
ADCY5	GNAI1
UBC	GNA13
TGFBR1	GNA13
GNA13	S1PR3
GNA13	S1PR2
RGS16	GNA13
PTK2B	GNA13
PRKCE	GNA13
PRKCD	GNA13
PRKCB	GNA13
GNA12	GNA13
GNA13	F2R
GNA13	DRD5
CTNNB1	GNA13
CDH2	GNA13
ARHGEF12	GNA13
GNA13	ARHGEF1
AKAP3	GNA13
UBC	GNA12
TEC	GNA12
UBC	GNA11
TRPC3	GNA11
GNA11	PTGIR

GNA11	HTR2B
GNA11	EDNRA
GNA11	CHRM2
GNA11	ADRA1B
UBC	GMPS
UBC	GMNN
SIX3	GMNN
MCM6	GMNN
CDC20	GMNN
UBC	GMFG
UBC	GMDS
GM2A	PLD2
RBM48	GLYAT
PTN	GLYAT
UBC	GLUL
SKIL	GLUL
PLEKHF2	GLUL
NUDT18	GLUL
HSPD1	GLUL
DYNLL1	GLUL
UBC	GLUD1
MAP3K14	GLUD1
UBC	GLTSCR2
HAP1	GLTSCR2
UBC	GLTP
UBC	GLT8D1
UBC	GLS
UBC	GLRX3
GLRX3	PRKD2
GLRX3	ADAMTSL4
UBC	GLRX
NFIC	GLRX
MAP3K5	GLRX
UBC	GLOD4
UBC	GLO1
SUFU	GLIS3
UBC	GLIPR2
GLI1	ZIC2
GLI1	ZIC1
UBC	GLI1
SUFU	GLI1
NFKB2	GLG1
UBC	GLE1
UBC	GLB1

FXR2	GKAP1
UBC	GK5
UBC	GJC1
TJP1	GJC1
MIP	GJA8
GJA1	GJA5
AAR2	GJA5
TJP1	GJA3
MIP	GJA3
GJA1	GJA3
CAV1	GJA3
UBQLN4	GJA1
UBC	GJA1
TJP1	GJA1
GJA1	S100A1
PRKCE	GJA1
PRKCA	GJA1
NEDD4	GJA1
MAPK7	GJA1
MAPK3	GJA1
MAPK1	GJA1
PXN	GIT2
ARHGEF7	GIT2
WDR33	GIT1
UBC	GIT1
TXNDC9	GIT1
TRIB3	GIT1
TGFB1I1	GIT1
TERF1	GIT1
SRRT	GIT1
SRC	GIT1
RGS2	GIT1
PXN	GIT1
PTK2	GIT1
PPFIA4	GIT1
PPFIA3	GIT1
PPFIA2	GIT1
PPFIA1	GIT1
PLCG1	GIT1
PFDN1	GIT1
MAN2A2	GIT1
LAMTOR5	GIT1
KIF1A	GIT1
HMOX2	GIT1

GRK5	GIT1
GRB2	GIT1
DDX24	GIT1
CCDC113	GIT1
C8orf33	GIT1
ARHGEF7	GIT1
ADRBK2	GIT1
ADRBK1	GIT1
GIP	GIPR
GIPC2	IGSF21
UBC	GIPC1
GIPC1	TYRP1
GIPC1	TPBG
GIPC1	RGS19
GIPC1	NTRK2
GIPC1	NTRK1
GIPC1	NRP1
GIPC1	ITGA6
GIPC1	CD93
UBC	GINS3
GINS4	GINS3
GINS2	GINS3
UBC	GINS2
GINS4	GINS2
UBC	GINS1
GINS4	GINS1
UBC	GINM1
CPSF6	GIN1
UBC	GIGYF2
IGF1R	GIGYF1
GRB10	GIGYF1
UBC	GID8
GHRL	GHSR
GHRHR	GHRH
UBC	GHITM
GHR	GH1
UBC	GGPS1
UBC	GGH
UBC	GGCX
UBC	GGCT
UBC	GGA3
RABEP1	GGA3
ARF6	GGA3
UBC	GGA2

TRIM23	GGA2
SYNRG	GGA2
SPG21	GGA2
SORT1	GGA2
SORL1	GGA2
RABEP1	GGA2
PIN1	GGA2
CLTC	GGA2
CLINT1	GGA2
BACE1	GGA2
GGA2	ARF3
UBC	GGA1
GGA1	SYNRG
GGA1	SORT1
GGA1	SORL1
RNF11	GGA1
RABEP1	GGA1
GGA1	M6PR
CLTC	GGA1
GGA1	BACE1
ARF6	GGA1
GGA1	ARF3
NRTN	GFRA2
NRTN	GFRA1
UBC	GFPT1
UBC	GFM2
UBC	GFM1
GPS1	GFER
COPS5	GFER
COPS2	GFER
BNIPL	GFER
ASCC2	GFER
VIM	GFAP
GFAP	ROCK1
PSEN1	GFAP
PRKCA	GFAP
GFAP	PDLIM1
GRAP2	GFAP
GFAP	CEP76
UBC	GET4
GET4	TTR
GET4	TMEM256
SNCAIP	GET4
GET4	PRKCSH

GET4	PRKAB2
PLK1	GET4
GET4	KIFC2
KAT5	GET4
EEF1G	GET4
GET4	CAPNS1
ATP5H	GET4
GEMIN6	GEMIN7
UBC	GEMIN5
SNRPE	GEMIN5
SNRPD3	GEMIN5
SNRPD2	GEMIN5
SNRPB	GEMIN5
GEMIN4	GEMIN5
GEMIN2	GEMIN5
DDX20	GEMIN5
UBC	GEMIN4
SRRT	GEMIN4
GEMIN4	SPRY1
SNRPB	GEMIN4
RIPK2	GEMIN4
PIAS3	GEMIN4
NFKB2	GEMIN4
MAP3K1	GEMIN4
GEMIN4	LGALS3
LGALS1	GEMIN4
GEMIN6	GEMIN4
GEMIN4	DICER1
DHX9	GEMIN4
DDX20	GEMIN4
CALCOCO2	GEMIN4
AGO2	GEMIN4
GEMIN6	GEMIN2
DHX9	GEMIN2
UBC	GDPD1
GFRA2	GDNF
GFRA1	GDNF
UBC	GDI2
RAB4A	GDI2
BMPR1A	GDF9
BMPR1A	GDF5
ACVR2B	GDF5
ACVR2B	GDF11
UBC	GDA

DLG1	GDA
GCSH	ING5
TNFRSF1A	GCN1L1
MYC	GCN1L1
UBC	GCM1
SUMO1	GCM1
FBXW2	GCM1
GCLC	GCLM
GCKR	GCK
GCHFR	GCH1
AHSA1	GCH1
GLP2R	GCG
GLP1R	GCG
CPE	GCG
UBC	GCC2
NINL	GCC1
CPSF7	GCC1
CCDC85B	GCC1
SERF2	GC
ACTC1	GC
ACTA1	GC
SERPINB9	GBP2
SEPHS1	GBP2
RPS6KA1	GBP2
LSM2	GBP2
HSPE1	GBP2
GBP2	FXR1
BTBD2	GBP2
YWHAG	GBF1
UBC	GBF1
TNFRSF1B	GBF1
UBC	GBE1
GABARAPL2	GBAS
UBC	GATAD2B
SMAD2	GATAD2B
RBBP4	GATAD2B
MTA2	GATAD2B
HDAC2	GATAD2B
UBC	GATAD2A
GATAD2A	SOX2
RBBP4	GATAD2A
HDAC2	GATAD2A
HDAC1	GATAD2A
CHD4	GATAD2A

SP1	GATA6
HNF1A	GATA5
GATA4	ZFPM2
GATA4	TBX5
NFATC4	GATA4
JARID2	GATA4
SPI1	GATA2
SMAD4	GATA2
CP	GAST
TYRO3	GAS6
MERTK	GAS6
AXL	GAS6
CAPN2	GAS2
UBC	GART
UBC	GARS
NARS	GARS
IARS	GARS
DARS	GARS
CELSR2	GARS
AARS	GARS
UBC	GAR1
YWHAB	GAPVD1
UBC	GAPVD1
VCL	GAPDH
UBC	GAPDH
TRAF1	GAPDH
TNFRSF1B	GAPDH
TK1	GAPDH
TBK1	GAPDH
SUMO1	GAPDH
SIAH1	GAPDH
SERPINB9	GAPDH
RPA2	GAPDH
RIPK2	GAPDH
PXN	GAPDH
PSMD11	GAPDH
PRPF40A	GAPDH
PRKCI	GAPDH
PRDX1	GAPDH
PLD2	GAPDH
PDIA2	GAPDH
PAFAH1B3	GAPDH
NPM1	GAPDH
MAP3K3	GAPDH

MAP3K14	GAPDH
LAMTOR5	GAPDH
LAMA4	GAPDH
HTT	GAPDH
GAS7	GAPDH
ESR1	GAPDH
EP300	GAPDH
EGFR	GAPDH
EEF1A1	GAPDH
BTBD2	GAPDH
BPGM	GAPDH
ATXN1	GAPDH
SPTAN1	GAP43
RABEP1	GAP43
PRKCB	GAP43
CALM1	GAP43
UBC	GANAB
PTPRC	GANAB
PRKCSH	GANAB
MYC	GANAB
UBC	GAMT
TRIP13	GALT
GNAQ	GALR2
GAL	GALR1
UBC	GALNT2
MUC1	GALNT2
MUC1	GALNT1
UBC	GALK2
UBC	GALK1
GAL	GALR2
UBC	GAK
HSPA8	GAK
CLTC	GAK
CDK5	GAK
CCNG1	GAK
AXIN1	GAK
AP2M1	GAK
AP1M2	GAK
AP1M1	GAK
UBC	GADD45GIP1
TSNAX	GADD45G
RXRA	GADD45G
RWDD2B	GADD45G
PPARG	GADD45G

PPARA GADD45G
PCNA GADD45G
MAP3K4 GADD45G
GADD45GIP1 GADD45G
GADD45A GADD45G
ESR1 GADD45G
EEF1A1 GADD45G
CDK1 GADD45G
CCNB1 GADD45G
PPARG GADD45B
PPARA GADD45B
MAP3K4 GADD45B
GADD45B GADD45GIP1
GADD45B GADD45A
ESR1 GADD45B
CCNB1 GADD45B
UBC GADD45A
RXRA GADD45A
HIST4H4 GADD45A
GADD45A GADD45GIP1
FTL GADD45A
GADD45A DCTN2
HAAO GAD1
PRKCA GABRR1
PRKCG GABRA1
DRD4 GABRA1
LMO3 GABPB1
UBC GABPA
SUMO2 GABPA
SP3 GABPA
SP1 GABPA
KEAP1 GABPA
GABPB1 GABPA
SH3GL3 GABBR2
GABBR1 GABBR2
ATF5 GABBR2
ATF4 GABBR2
GABARAPL2 ULK1
UBC GABARAPL2
TBC1D5 GABARAPL2
GABARAPL2 BCL2L13
UBC GABARAPL1
SQSTM1 GABARAPL1
GABARAPL1 NBR1

CALCOCO2	GABARAPL1
GABARAPL1	ATG7
VPS28	GABARAP
GABARAP	ULK1
UBC	GABARAP
SQSTM1	GABARAP
NSF	GABARAP
GABARAP	MPC1
GABARAP	GPHN
GABARAP	GABRG2
GABARAP	ATG7
GABARAP	AES
PLCG2	GAB2
GRAP2	GAB2
CD247	GAB2
VCL	G3BP1
UBC	G3BP1
PXN	G3BP1
PIN1	G3BP1
EIF4G1	G3BP1
UBC	G2E3
LST1	G2E3
WNT1	FZD8
LRP6	FZD8
UBC	FZD7
UBC	FZD6
SFRP1	FZD6
FZD5	LRP6
UBC	FZD2
WNT5A	FZD1
WNT3A	FZD1
UBC	FZD1
UBC	FYTTD1
VAV1	FYN
UBC	FYN
FYN	SKAP2
FYN	PTPN5
PRKCD	FYN
FYN	MCAM
LCK	FYN
FYN	ITK
GP6	FYN
CD36	FYN
VASP	FYB

SKAP2	FYB
SKAP1	FYB
LCP2	FYB
FYN	FYB
RBM48	FXYD6-FXYD2
FXYD1	ATP1B1
ATP1A1	FXYD1
UBC	FXR2
FXR2	TSC22D4
TRAF2	FXR2
FXR2	THAP1
FXR2	RTN3
FXR2	RPIA
FXR2	RALYL
FXR2	PAICS
FXR2	MCRS1
FXR2	KCTD4
HNRNPC	FXR2
FXR2	FBP1
FXR2	DPPA2
CCDC85B	FXR2
FXR2	CBS
FXR2	CALCOCO2
FXR2	AP2M1
FXR2	AP1M1
VCL	FXR1
UBC	FXR1
FXR2	FXR1
C14orf1	FXR1
RSG1	FUZ
ITGB2	FUT4
FUT4	CR1
FUS	YBX1
VCL	FUS
UBC	FUS
RXRA	FUS
FUS	PTBP1
PACS1	FURIN
UBC	FUK
VCL	FUCA1
UBC	FUBP3
VIM	FUBP1
UBC	FUBP1
CELF1	FUBP1

UBC	FTSJ3
UBC	FTSJ2
UBC	FTO
UBC	FTL
SMAD9	FTL
PTN	FTL
MPHOSPH6	FTL
FTL	IGDCC3
FTL	FTH1
FTL	CP
UBR1	FTH1
UBC	FTH1
FXR2	FTH1
FTH1	ELL3
DAXX	FTH1
BRD7	FTH1
SMAD4	FSTL3
MSTN	FSTL3
INHBA	FSTL3
CD14	FSTL1
CGB	FSHB
CGA	FSHB
UBC	FSD1
YWHAG	FRY
UBC	FRY
SOS1	FRS2
RET	FRS2
FRS2	RAPGEF1
PTPRF	FRS2
PTPN11	FRS2
NTRK1	FRS2
GRB2	FRS2
FRS2	FGFR1
EGFR	FRS2
DDR1	FRS2
CBL	FRS2
UBC	FRMD8
YWHAG	FRMD6
YWHAB	FRMD6
UBC	FRMD6
TSC22D4	FRMD6
RASSF8	FRMD6
MLH1	FRMD6
UBC	FRMD5

GSK3B	FRAT2
GSK3B	FRAT1
GNAI2	FPR1
ANXA1	FPR1
ADRBK1	FPR1
UBC	FPGS
UBC	FOXRED2
OS9	FOXRED2
SUMO2	FOXP4
UBC	FOXO3
TP53	FOXO3
SMAD2	FOXO3
FOXO3	SIRT1
MDM2	FOXO3
FOXO3	FOXG1
FOXO3	CHUK
UBC	FOXO1
SMAD4	FOXO1
FOXO1	SIRT2
FOXO1	SIRT1
FOXO1	HOXA5
FOXO1	HNF4A
FOXO1	G6PC
FOXO1	FOXG1
FOXO1	ATG7
EXOSC8	FOXN3
CCNE1	FOXM1
BAP1	FOXK2
BAP1	FOXK1
MED23	FOXJ3
SMAD2	FOXH1
FOXG1	FOXH1
DRAP1	FOXH1
SMAD1	FOXG1
EEF1G	FOXG1
TLE1	FOXE1
SUMO2	FOXC2
TLE1	FOXA2
OTX2	FOXA2
ONECUT1	FOXA2
NCOA1	FOXA2
MIP	FOXA2
HOXA5	FOXA2
HIST3H3	FOXA1

UBC	FOSL2
FOSL2	TEF
FOSL2	MAFB
JUND	FOSL2
JUNB	FOSL2
FOS	FOSL2
USF1	FOSL1
FOSB	MAFB
JUND	FOSB
JUNB	FOSB
FOSL1	FOSB
UBC	FOS
SUMO1	FOS
RUNX2	FOS
FOS	RPS6KA4
RPS6KA1	FOS
NCOR2	FOS
NCOA1	FOS
FOS	MMP1
FOS	MAPK1
FOS	MAFB
FOS	MAF
FOS	JUND
FOS	JUNB
FOS	GTF2F2
FOS	GATA4
FOS	ETS1
FOS	EPHB2
UBC	FOLR1
VCL	FOCAD
UBC	FOCAD
UBC	FNTB
RHOB	FNTB
HRAS	FNTB
GBP1	FNTB
FNTA	FNTB
UBC	FNTA
TGFBR1	FNTA
ACVR1	FNTA
UBC	FNDC3B
SUMO2	FNBP4
PRPF40A	FNBP4
PLXNA2	FNBP4
KHDRBS1	FNBP4

UBC	FNBP1L
UBC	FNBP1
FNBP1	RND2
FN1	FBLN1
FN1	COL1A1
TGFB3	FMOD
FMNL1	GAS7
SETDB1	FLYWCH1
ERBB3	FLYWCH1
UBC	FLVCR1
PFN2	FLT3LG
UBC	FLT3
SOCS1	FLT3
PIK3R1	FLT3
FLT3LG	FLT3
UBC	FLOT2
CAV2	FLOT2
UBC	FLOT1
SORBS1	FLOT1
NGB	FLOT1
GOLM1	FLOT1
FLOT2	FLOT1
CBL	FLOT1
CAV1	FLOT1
UBC	FLNC
SGCG	FLNC
SGCD	FLNC
NPHP1	FLNC
MYOZ1	FLNC
MYOT	FLNC
MAP2K4	FLNC
INPPL1	FLNC
HIPK3	FLNC
FURIN	FLNC
CAPN3	FLNC
UBC	FLNB
SMURF2	FLNB
PSEN2	FLNB
PSEN1	FLNB
NPHP1	FLNB
GP1BA	FLNB
ESR1	FLNB
UBC	FLII
TRAIP	FLII

OPTN	FLII
ACTL6A	FLII
ACTB	FLII
SRF	FLI1
SMAD9	FLI1
ERG	FLI1
UBC	FKBP5
PGR	FKBP5
CHUK	FKBP5
UBC	FKBP4
UBC	FKBP3
HDAC2	FKBP3
HDAC1	FKBP3
UBC	FKBP1A
TGFBR1	FKBP1A
RPTOR	FKBP1A
FKBP1A	PPP3R1
FKBP1A	PPP3CA
UBC	FKBP15
NRL	FIZ1
UBC	FIS1
UBC	FIP1L1
FIP1L1	CPSF4
UBC	FIGNL1
UBC	FIG4
HTT	FICD
UBC	FHL3
FHL3	RFX6
FHL3	RBM42
FHL3	MAPK1
LASP1	FHL3
ADAMTSL4	FHL3
ZFYVE9	FHL2
UBC	FHL2
TTN	FHL2
SFPQ	FHL2
REV1	FHL2
PSEN2	FHL2
MCM7	FHL2
ITGB6	FHL2
ITGB2	FHL2
ITGB1	FHL2
ITGA7	FHL2
ITGA3	FHL2

IGFBP5	FHL2
FHL5	FHL2
FHL3	FHL2
CTNNB1	FHL2
CARD8	FHL2
BRCA1	FHL2
UBC	FH
UBC	FGR
FGR	STAT3
FGR	SNCA
FGR	SLAMF1
FGR	PLAUR
FGR	KHDRBS1
FGR	HSP90AA1
FGR	DOK1
FGR	DAB2
FGR	CCR3
VTN	FGG
SERPINA5	FGG
ITGAM	FGG
ICAM1	FGG
FGB	FGG
FGA	FGG
UBC	FGFR4
STAT1	FGFR4
FGFR4	FGF6
FGFR4	FGF19
UBC	FGFR2
PLCG1	FGFR2
GRB2	FGFR2
FGF9	FGFR2
FGF8	FGFR2
FGF6	FGFR2
FGF10	FGFR2
FGF1	FGFR2
STK24	FGFR1OP2
MOB4	FGFR1OP2
UBC	FGFR1
PLCG1	FGFR1
NEDD4	FGFR1
HTR1A	FGFR1
FRS3	FGFR1
FGF5	FGFR1
FGF1	FGFR1

FIBP	FGFBP1
FGF1	FGFBP1
HSPG2	FGF7
FGFR2	FGF7
VTN	FGF2
SDC3	FGF2
RPS19	FGF2
PF4	FGF2
HSPG2	FGF2
FGFR4	FGF2
FGFR2	FGF2
FGFR1	FGF2
FGFBP1	FGF2
CXCL13	FGF2
CSNK2A2	FGF2
CD44	FGF2
FIBP	FGF1
FGF1	FGFR4
ACVR1	FGD6
FBLN1	FGB
FGA	SERPINA5
FGA	FGB
FGA	F2
FGA	CDH5
GNAQ	FFAR1
UBC	FEZ2
FEZ1	TXNDC9
FEZ1	PTH
FEZ1	PAM16
FEZ1	P4HB
FEZ1	OLFML3
FEZ1	COL9A2
UBC	FES
EGFR	FES
UBC	FERMT2
FBLIM1	FERMT2
WRN	FEN1
VCL	FEN1
UBC	FEN1
RAD1	FEN1
EEF1G	FEN1
BLM	FEN1
UBC	FEM1C
UBC	FEM1B

TCEB2 FEM1B
TCEB1 FEM1B
CUL2 FEM1B
COPS6 FEM1B
APAF1 FEM1B
UBC FEM1A
UBC FECH
UBC FDXR
FHIT FDXR
FDX1 FDXR
UBC FDFT1
RUVBL2 FDFT1
FDFT1 ARL6IP1
FDFT1 ANXA5
MASP1 FCN1
CCDC85B FCHSD2
CCDC158 FCHSD2
ITSN2 FCHSD1
UBC FCHO2
FCHO1 ACVR1
CA6FCGRT
ALB FCGRT
ZAP70 FCGR3A
PTPRC FCGR3A
LCK FCGR3A
GP6 FCGR3A
CD38 FCGR3A
INPPL1 FCGR2B
C14orf1 FCGR2B
UBC FCGR2A
HCK FCGR2A
GP6 FCGR2A
FYN FCER2
SYK FCER1G
PLCG2 FCER1G
NCR1 FCER1G
GP6 FCER1G
FCER1A FCER1G
CD79A FCAR
UBC FBXW9
COPS6 FBXW9
SKP1 FBXW8
CUL7 FBXW8
CUL1 FBXW8

SUMF2	FBXW7
SREBF1	FBXW7
SKP1	FBXW7
NFKB2	FBXW7
NFE2L1	FBXW7
MYC	FBXW7
MMS22L	FBXW7
CDC34	FBXW7
CCNE1	FBXW7
UBC	FBXW5
SKP1	FBXW5
MDFI	FBXW5
CUL4A	FBXW5
CUL1	FBXW5
SKP1	FBXW4
CUL1	FBXW4
SKP1	FBXW2
CUL1	FBXW2
YAP1	FBXW11
WEE1	FBXW11
USP47	FBXW11
UBC	FBXW11
SKP1	FBXW11
REST	FBXW11
PDCD4	FBXW11
NFKBIE	FBXW11
NFKBIB	FBXW11
NFKBIA	FBXW11
NFKB2	FBXW11
KDR	FBXW11
IFNAR1	FBXW11
FAF1	FBXW11
DEPTOR	FBXW11
CUL7	FBXW11
CUL1	FBXW11
CTNNB1	FBXW11
CLSPN	FBXW11
CDC25B	FBXW11
CDC25A	FBXW11
ATF4	FBXW11
SKP1	FBXO9
UBC	FBXO7
COPS6	FBXO7
BIRC2	FBXO7

SKP1	FBXO6
UBC	FBXO5
SKP1	FBXO5
FZR1	FBXO5
CDC27	FBXO5
CDC20	FBXO5
SKP1	FBXO46
UBC	FBXO45
SKP1	FBXO44
CUL1	FBXO44
COPS6	FBXO44
SKP1	FBXO4
CUL1	FBXO4
UBC	FBXO34
TGFBR1	FBXO34
SMAD4	FBXO34
SKP1	FBXO34
SKP1	FBXO33
CUL1	FBXO33
SMAD4	FBXO30
SMAD1	FBXO30
ACVR1	FBXO30
UBC	FBXO3
TGFBR1	FBXO3
SMAD3	FBXO3
SMAD2	FBXO3
SMAD1	FBXO3
SKP1	FBXO3
CUL1	FBXO3
UBC	FBXO28
SKP1	FBXO28
TGFBR1	FBXO24
SMAD1	FBXO24
UBC	FBXO22
COPS5	FBXO22
UBE2D2	FBXO2
SKP1	FBXO2
ITGB1	FBXO2
CUL1	FBXO2
AHSG	FBXO2
SKP1	FBXO17
CUL1	FBXO17
COPS6	FBXO17
COPS5	FBXO17

UBC	FBXO11
SKP1	FBXO11
CUL1	FBXO11
COPS6	FBXO11
COPS5	FBXO11
SKP1	FBXL7
UBC	FBXL6
SKP1	FBXL6
UBC	FBXL3
SKP1	FBXL3
UBC	FBXL18
SKP1	FBXL15
CUL1	FBXL15
ASCC2	FBP1
ZNF8	FBN2
SMAD2	FBN2
ELN	FBN2
VCAN	FBN1
FBN1	MYOC
FBN1	MFAP5
FBN1	MFAP2
FBN1	LOX
FBN2	FBN1
FBLN2	FBN1
ELN	FBN1
DCN	FBN1
CALR	FBN1
FBLN5	LOXL1
VCAN	FBLN2
ELN	FBLN2
COL4A1	FBLN2
UBC	FBL
TBC1D17	FBL
TAF9	FBL
SUMO1	FBL
SNRPN	FBL
PSMB6	FBL
PRMT1	FBL
NPM1	FBL
NOP56	FBL
MAP3K14	FBL
ILK	FBL
DDX5	FBL
COPS5	FBL

ARRB2	FBL
GCC1	FBF1
UBC	FAU
RPS9	FAU
RPS6	FAU
RPS23	FAU
RPS16	FAU
RPS13	FAU
UBC	FAT1
UBC	FASTKD5
CCDC85B	FASTKD5
NFKB2	FASN
TNFRSF1A	FASLG
SUMO1	FASLG
SRGAP2	FASLG
PTPN13	FASLG
PSTPIP1	FASLG
PIK3R1	FASLG
FASLG	PDCD6
FASLG	PACSIN2
FASLG	LYN
LCK	FASLG
ITSN2	FASLG
GRB2	FASLG
GRAP2	FASLG
FASLG	GRAP
FYN	FASLG
FNBP1	FASLG
FADD	FASLG
DAXX	FASLG
FAS	YES1
UBE2I	FAS
UBC	FAS
SUMO1	FAS
RIPK1	FAS
PTPN13	FAS
FAS	PDCD6
LCK	FAS
HIPK3	FAS
FYN	FAS
FASLG	FAS
FAF1	FAS
FADD	FAS
FAS	EZR

DAXX FAS
FAS CFLAR
CAV1 FAS
FAS CASP8
FAS CASP10
C14orf1 FAS
UBC FARSB
FARSA FARSB
UBC FARP1
UBC FAR1
FANCL FANCM
FANCD2 FANCL
FANCL C17orf70
UBC FANCI
TNFRSF1B FANCI
TNFRSF1A FANCI
SUMO2 FANCI
ILK FANCI
FANCL FANCI
FANCD2 FANCI
UBC FANCG
FANCG SAMD3
KRT19 FANCG
FANCG GABPB1
FANCG FANCM
FANCG FANCL
FANCG FANCC
OLFM2 FANCF
FANCM FANCF
FANCL FANCF
FANCG FANCF
FANCF FANCE
FANCC FANCF
UBC FANCE
FANCM FANCE
FANCG FANCE
FANCD2 FANCE
FANCC FANCE
TNFRSF1A FANCD2
RAD51 FANCD2
FANCD2 RAD18
FANCD2 FAN1
FANCC ZBTB32
STAT1 FANCC

HSPA8	FANCC
FANCM	FANCC
FANCL	FANCC
FANCD2	FANCC
UBC	FANCA
FANCA	TOP3A
SPTAN1	FANCA
SMARCD3	FANCA
SMARCC1	FANCA
RPA1	FANCA
FANCM	FANCA
FANCL	FANCA
FANCG	FANCA
FANCA	FANCF
FANCA	FANCE
FANCA	FANCC
CHUK	FANCA
FANCA	C19orf40
FANCA	C17orf70
BRCA1	FANCA
BLM	FANCA
UBC	FAM98B
UBC	FAM98A
UBC	FAM96B
MMS19	FAM96B
CIAO1	FAM96B
UBC	FAM86A
PRTFDC1	FAM86A
UBC	FAM83D
CSNK1E	FAM83D
RAB2A	FAM71C
UBC	FAM63B
UBC	FAM60A
GTPBP3	FAM60A
CCDC85B	FAM50B
UBC	FAM49B
UBC	FAM46C
DAZAP2	FAM46C
UBC	FAM46A
FAM46A	SPINT1
COL4A2	FAM46A
BAG6	FAM46A
ATXN1	FAM46A
UBC	FAM21A

CCDC85B	FAM214B
UBC	FAM213B
UBC	FAM213A
UBC	FAM208B
CCDC85B	FAM208B
UBC	FAM195B
UBC	FAM192A
UBC	FAM189B
VCL	FAM179B
ZNF598	FAM178B
LRSAM1	FAM178B
SMURF2	FAM175B
UIMC1	FAM175A
UBC	FAM175A
SUMO2	FAM175A
BRE	FAM175A
BRCA1	FAM175A
BABAM1	FAM175A
UBC	FAM172A
UBC	FAM169A
SMAD2	FAM161B
PPM1F	FAM161A
UBC	FAM160B1
RTN3	FAM160A2
PLOD3	FAM153A
CCDC85B	FAM13C
UBC	FAM136A
KAT5	FAM135B
UBC	FAM134C
PHYHIP	FAM131A
ZNF512B	FAM129B
UBC	FAM129B
UBC	FAM129A
CCDC85B	FAM124B
TRAF2	FAM120B
UBC	FAM120A
UBC	FAM118B
RASGRP2	FAM118B
UBC	FAM115A
SF3B4	FAM115A
UBC	FAM114A2
UBC	FAM114A1
UBC	FAM111B
UBC	FAM111A

CSNK1E FAM110C
CSNK1E FAM110A
VIM FAM107A
TRAF2 FAM107A
TADA2A FAM107A
KRT19 FAM107A
KRT15 FAM107A
EFEMP2 FAM107A
CCDC85B FAM107A
CALCOCO2 FAM107A
UBC FAM105A
UBC FAH
VCPIP1 FAF2
INSIG1 FAF2
FAF2 AMFR
VCP FAF1
UBC FAF1
SKP1 FAF1
SKIL FAF1
NPLOC4 FAF1
FADD FAF1
ASCC2 FAF1
UBC FADS3
UBC FADS2
UBC FADS1
TNFRSF1A FADD
FADD TNFRSF10B
FADD TNFRSF10A
FADD MYD88
FADD ABCA1
VIM FABP4
LIPE FABP4
VPS28 FABP3
PPARA FABP1
PXN FAAH
F5 PROS1
F2 F5
F10 F5
UBC F3
PLG F3
F7 F3
JAM3 F2RL2
F2 F2RL2
UBC F2RL1

F2RL1 ST14
F2RL1 PLG
UBC F2R
PDCD6IP F2R
GNAI2 F2R
GNA15 F2R
GNA12 F2R
F2 F2R
CAV1 F2R
F2 SERPINE2
FGG F13B
HSPB1 F13A1
FGG F13A1
F13B F13A1
KRT1 F12
GP1BA F12
CD93 F12
APP F12
APOH F12
UBC F11R
TJP1 F11R
SGTA F11R
SERPINF2 F11
SERPINA5 F11
HGF F11
GP1BA F11
F2 F11
F10 PROS1
UBC EZR
TSC1 EZR
SYK EZR
SLC9A3R2 EZR
SLC9A3R1 EZR
SELL EZR
SDC2 EZR
SCYL3 EZR
EZR ROCK1
PRKCA EZR
PIK3R1 EZR
PALLD EZR
LCK EZR
ICAM1 EZR
FASLG EZR
FADD EZR

EGFR EZR
DLG1 EZR
EZRCLIC5
CFTR EZR
CD44 EZR
ARHGDIA EZR
ACTB EZR
YY1 EZH2
EZH2 WSB2
VAV1 EZH2
UBC EZH2
EZH2 SNAI1
SIRT1 EZH2
RBBP4 EZH2
EZH2 PPP1R8
POLA2 EZH2
NINL EZH2
EZH2 KLHDC2
HIST3H3 EZH2
HIST1H3A EZH2
HDAC2 EZH2
HDAC1 EZH2
EZH2 EZH1
EED EZH2
E2F6 EZH2
EZH2 DNMT3B
DNMT1 EZH2
CTNNB1 EZH2
CCDC85B EZH2
ATP1A1 EZH2
UBC EZH1
EED EZH1
SIX1 EYA2
SIX1 EYA1
UBC EXT2
EXT2 GALNT5
EXT2 EXT1
DIS3 EXOSC9
DDX39B EXOSC9
ZFP36 EXOSC8
EXOSC8XRN2
UPF2 EXOSC8
UBC EXOSC8
EXOSC8RPP14

EXOSC8RASSF1
EXOSC8MKRN1
EXOSC8LSM7
EXOSC8EXOSC9
EXOSC8EXOSC5
EXOSC10 EXOSC8
EXOSC8EXOSC1
DIS3 EXOSC8
EXOSC8CWC22
EXOSC9EXOSC7
EXOSC5ZNF558
UBC EXOSC5
SFPQ EXOSC5
EXOSC5PA2G4
NUP210 EXOSC5
LSM5 EXOSC5
KIAA1217 EXOSC5
EXOSC9EXOSC5
EXOSC2EXOSC5
EXOSC10 EXOSC5
EXOSC1EXOSC5
EXOSC5CDK5RAP1
UBC EXOSC3
KHSRP EXOSC3
EXOSC9EXOSC3
EXOSC8EXOSC3
EXOSC5EXOSC3
EXOSC2EXOSC3
EXOSC10 EXOSC3
DIS3 EXOSC3
CDK5RAP1 EXOSC3
UBC EXOSC2
EXOSC2KHSRP
EXOSC2EXOSC9
EXOSC2EXOSC7
UBC EXOSC10
EXOSC10 SCRIB
RUVBL2 EXOSC10
EXOSC10 PTGES2
EXOSC10 NOMO1
MPHOSPH6 EXOSC10
EXOSC10 MAP3K14
EXOSC10 HIATL1
EXOSC10 FOXRED1

EXOSC10	FERMT3
EXOSC10	EXOSC7
DIS3	EXOSC10
EXOSC10	CHPF
EXOSC10	C1D
UBC	EXOSC1
EXOSC1	EXOSC9
EXOSC1	EXOSC7
UBC	EXOC8
TADA2A	EXOC8
KRT19	EXOC8
KRT15	EXOC8
EXOC8	EXOC5
CCDC85B	EXOC8
EXOC8	BLOC1S6
EXOC7	IFT20
EXOC7	EXOC4
CCDC85B	EXOC7
UBC	EXOC6B
UBC	EXOC6
UBC	EXOC5
ARF6	EXOC5
EXOC5	EXOC3
EXOC4	EXOC3
UBC	EXOC2
SERGEF	EXOC2
RALA	EXOC2
EXOC8	EXOC2
EXOC7	EXOC2
EXOC3	EXOC2
EXOC4	EXOC1
DAZAP2	EXD3
UBC	EXD2
EWSR1	ZNF165
EWSR1	ZDHHC3
EWSR1	YY1AP1
EWSR1	WWP2
EWSR1	WT1
EWSR1	WDR37
WBP4	EWSR1
EWSR1	VPS72
UBC	EWSR1
EWSR1	TULP2
EWSR1	TSPAN3

EWSR1	TRPV5
TRIM37	EWSR1
EWSR1	TPGS2
EWSR1	SUV39H2
EWSR1	SSBP2
EWSR1	SNRPC
SMAD4	EWSR1
SMAD1	EWSR1
EWSR1	SLC22A24
EWSR1	SLC1A1
EWSR1	RPS15A
EWSR1	RPL31
EWSR1	RNF183
EWSR1	RMND5B
EWSR1	RASL11B
RALYL	EWSR1
EWSR1	RAD23A
EWSR1	RAB37
PXN	EWSR1
EWSR1	PTK2B
EWSR1	PRTFDC1
EWSR1	POU4F1
EWSR1	PLSCR1
EWSR1	PGLS
EWSR1	PDHX
EWSR1	NTNG2
EWSR1	NSUN4
EWSR1	NPPB
NINL	EWSR1
EWSR1	NDUFB1
EWSR1	NBPF3
EWSR1	MYOZ2
EWSR1	MYO1F
EWSR1	MYL6
EWSR1	MVK
EWSR1	MTMR9
EWSR1	MSC
EWSR1	MRPS18B
EWSR1	MNS1
MDFI	EWSR1
EWSR1	MATK
EWSR1	MAPK1IP1L
EWSR1	LILRA3
EWSR1	KXD1

EWSR1 KRR1
EWSR1 KHDRBS2
KEL EWSR1
EWSR1 KCNMB1
EWSR1 HMGA1
EWSR1 HERPUD1
EWSR1 GPBP1L1
EWSR1 GNPDA1
EWSR1 FASN
EWSR1 FAM131C
EWSR1 ELAVL4
EWSR1 ELAVL3
DYNLL2 EWSR1
EWSR1 DMRTB1
DFFA EWSR1
EWSR1 CXADR
EWSR1 CUEDC2
EWSR1 CPSF7
CPSF6 EWSR1
EWSR1 CNST
EWSR1 CFDP1
EWSR1 CETP
EWSR1 CD177
EWSR1 CCDC91
EWSR1 C19orf57
EWSR1 C10orf12
EWSR1 BNIP3L
EWSR1 BARD1
BAD EWSR1
EWSR1 AGT
ACTL6A EWSR1
UBC EVPL
SPRR3 EVPL
PPL EVPL
KRT5 EVPL
KRT2 EVPL
KRT1 EVPL
IVL EVPL
DSP EVPL
TCERG1 EVL
PRPF40A EVL
APBB1 EVL
NUDT18 EVI5L
UBC EVA1A

PTNEVA1A
RFWD2 ETV5
UBC ETV4
RFWD2 ETV4
EP300 ETV4
UBC ETV3
RPS6KA1 ETV1
RFWD2 ETV1
UBC ETS2
ETS2 STAT5B
ETS2 GATA3
ETS2 ETS1
ETS2 CDK10
UBC ETS1
TP53 ETS1
ETS1 STAT5B
ETS1 SRSF11
MAPK7 ETS1
ETS1 MAF
ETS1 GATA3
UBC ETNK1
UBC ETFA
ETFB ETFA
UBC ETF1
GSPT1 ETF1
CSNK1A1 ETF1
CORO1C ETF1
UBC ESYT2
UBC ESYT1
SRCESRRA
PPARGC1A ESRRA
NRIP1 ESRRA
NCOA3 ESRRA
ESRRA LYST
ESRRG ESRRA
ESR2 ZBTB17
UBC ESR2
TRIM24 ESR2
ESR2 STAT5A
SP1 ESR2
ESR2 REXO4
ESR2 RBM39
ESR2 PPARGC1A
ESR2 PIAS3

ESR2	PIAS1
ESR2	PELP1
ESR2	OXT
ESR2	NCOA6
NCOA3	ESR2
NCOA2	ESR2
NCOA1	ESR2
ESR2	MED17
MED1	ESR2
ESR2	MAD2L1
ESR1	ESR2
ESR2	DNTTIP2
CALM1	ESR2
ESR1	YBX3
ESR1	YBX1
ESR1	WDR5
UBC	ESR1
ESR1	TUBB
ESR1	TUBA1A
ESR1	TRRAP
ESR1	TRIP4
TRIM28	ESR1
ESR1	TRIM25
TRIM24	ESR1
ESR1	TRAM1
TP53	ESR1
ESR1	TDG
ESR1	TAB2
ESR1	SYNCRIP
ESR1	STUB1
ESR1	STAT5A
ESR1	SP1
SMAD4	ESR1
SMAD2	ESR1
ESR1	SIN3A
ESR1	SF3B1
RXRA	ESR1
ESR1	RUVBL1
ESR1	RPS9
ESR1	RPS6KA1
ESR1	RPS3A
ESR1	RPS24
ESR1	RPS2
ESR1	RPS16

ESR1	RPS14
ESR1	RPS13
ESR1	RPL7A
ESR1	RPL7
ESR1	RPL36
ESR1	RPL18
ESR1	RPL13
ESR1	RFX4
ESR1	REXO4
ESR1	RBM39
ESR1	RBBP4
ESR1	RARA
ESR1	PRDX2
ESR1	PRDM2
ESR1	PPARGC1A
ESR1	PIK3R1
ESR1	PIAS1
ESR1	PHB
ESR1	PFN1
ESR1	PELP1
ESR1	PAK6
ESR1	PAK1
ESR1	OTUB1
ESR1	NUMA1
ESR1	NR2C2
ESR1	NPM1
ESR1	NOP58
ESR1	NME2
ESR1	NFKB1
NCOR2	ESR1
ESR1	NCOA6
ESR1	NCOA3
ESR1	NCOA2
ESR1	NCOA1
ESR1	MYO1B
ESR1	MYL6
ESR1	MYH9
ESR1	MTDH
ESR1	MTCH2
ESR1	MTA2
ESR1	MED24
ESR1	MED1
ESR1	MDM2
ESR1	MAPK1

KAT5	ESR1
JUP ESR1	
ESR1	JUNB
ESR1	ISL1
ESR1	HSPA8
ESR1	HSP90AA1
ESR1	HNRNPA3
ESR1	HNRNPA2B1
ESR1	HDAC4
HDAC2	ESR1
HDAC1	ESR1
ESR1	GTF2B
ESR1	GRIP1
ESR1	FOXO1
ESR1	FLII
ESR1	EIF4G3
ESR1	EIF4A1
ESR1	EIF3I
EGFR	ESR1
ESR1	DNTTIP2
ESR1	DHX9
ESR1	DHX15
ESR1	DDX5
ESR1	CUEDC2
ESR1	CLTCL1
ESR1	CLTC
ESR1	CEBPB
ESR1	CDK7
ESR1	CAV1
ESR1	BRI3BP
ESR1	BLOC1S1
ESR1	BCAR1
ESR1	ATP5B
ESR1	ANXA2
ESR1	AKT2
ESR1	AKAP13
ESR1	ACTN4
ACTN1	ESR1
ESR1	ACTB
UBC	ESPL1
PTTG1	ESPL1
ITGB2	ESM1
UBC	ESF1
UBC	ESD

UBC	ESCO2
SLC1A5	ERVW-1
SLC1A4	ERVW-1
EGFR	ERRFI1
UBC	ERP29
HSPA5	ERP29
UBC	ERO1L
PDIA3	ERO1L
PDIA2	ERO1L
P4HB	ERO1L
TRAF2	ERN1
SYVN1	ERN1
MAP3K5	ERN1
HSPA5	ERN1
COPS5	ERN1
UBC	ERMP1
AMFR	ERLIN2
UBC	ERLIN1
TRAF6	ERLIN1
ERLIN2	ERLIN1
TNFRSF1A	ERLEC1
UBC	ERI3
UBC	ERI1
TP53	ERH
RBM48	ERH
UBC	ERGIC3
UBC	ERGIC2
UBC	ERGIC1
UBC	ERG
HSPA8	ERG
ETS2	ERG
UBC	ERF
EGFR	EREG
COPS6	ERCC8
COPS5	ERCC8
COPS4	ERCC8
XPAERCC6	
UBC	ERCC6
TP53	ERCC6
SUMO2	ERCC6
RNF11	ERCC6
GTF2E2	ERCC6
ERCC8	ERCC6
ERCC6	ERCC5

CHEK2	ERCC6
UBC	ERCC5
ERCC3	ERCC5
XPC	ERCC3
UBC	ERCC3
TP53	ERCC3
GTF2H3	ERCC3
UBC	ERCC2
TP53	ERCC2
ERCC2	MMS19
GTF2H3	ERCC2
GTF2H1	ERCC2
ERCC5	ERCC2
ERCC3	ERCC2
CDK7	ERCC2
CCNH	ERCC2
ERCC1	XPA
UBC	ERCC1
ERCC1	SLX4
ERCC1	ERCC4
RIMS1	ERC2
UBC	ERC1
UBC	ERBB3
ERBB3	TNS3
ERBB3	SH2B3
ERBB3	PTK6
ERBB3	PIK3R2
ERBB3	NRG1
ERBB3	MUC1
ERBB3	JAK3
ERBB3	ITK
ERBB3	IL6ST
ERBB3	HCK
ERBB3	FER
ERBB3	ERBB4
EGFR	ERBB3
ERBB3	EGF
ERBB3	DAB1
ERBB3	CRK
ERBB3	CHN2
ERBB3	ABL2
UBC	ERBB2IP
SMAD2	ERBB2IP
SMAD1	ERBB2IP

ERBB2IP NOD2
ERBB2IP LRRC1
ERBB2IP CTNND1
ERBB2IP APC
ERBB2 VAV3
VAV2 ERBB2
VAV1 ERBB2
UBC ERBB2
UBA52 ERBB2
ERBB2 TXK
ERBB2 TOB1
ERBB2 TNS3
ERBB2 TENC1
TECERBB2
ERBB2 SYNGAP1
SYK ERBB2
STUB1 ERBB2
STAT3 ERBB2
STAT1 ERBB2
ERBB2 SOCS1
ERBB2 SLA2
ERBB2 SH3BP2
ERBB2 SH2D2A
ERBB2 SH2D1B
ERBB2 SH2B2
ERBB2 SERPINA3
ERBB2 RIN2
PTPN12 ERBB2
ERBB2 PTPN11
PTK2B ERBB2
PLCG2 ERBB2
PLCG1 ERBB2
ERBB2 PIK3R3
ERBB2 PIK3R2
PIK3R1 ERBB2
ERBB2 PAK1
NCK2 ERBB2
NCK1 ERBB2
ERBB2 MUC4
MUC1 ERBB2
ERBB2 MATK
ERBB2 MAPK8IP2
ERBB2 MAPK8IP1
LCK ERBB2

JAK2	ERBB2
JAK1	ERBB2
ITK	ERBB2
IRS1	ERBB2
IL6ST	ERBB2
HSPA4	ERBB2
HSP90AA1	ERBB2
GRB2	ERBB2
GRAP2	ERBB2
FGR	ERBB2
ERBB2	ERRFI1
ERBB4	ERBB2
ERBB3	ERBB2
ERBB2IP	ERBB2
EGFR	ERBB2
ERBB2	DOK6
ERBB2	DOK4
ERBB2	DOK1
DAB1	ERBB2
CTNNB1	ERBB2
CRKL	ERBB2
BLNK	ERBB2
ERBB2	BLK
ERBB2	BCAR3
ERBB2	ANKS1A
ERBB2	ACPP
ERBB2	ABL2
ABL1	ERBB2
UBC	ERAL1
EPS8	USP6NL
EPS8	SOS1
SHB	EPS8
EPS8	ITGB7
ITGB3	EPS8
ITGB2	EPS8
ITGB1	EPS8
EGFR	EPS8
CCDC85B	EPS8
EPS8	BLOC1S6
EPS8	BAIAP2L1
BAIAP2	EPS8
ACTA1	EPS8
EPS8	ABI1
UBC	EPS15L1

STON2	EPS15L1
ITSN2	EPS15L1
UBQLN1	EPS15
UBC	EPS15
STON2	EPS15
STAM2	EPS15
STAM	EPS15
SNAP91	EPS15
SH3BP4	EPS15
RNF11	EPS15
NUMBL	EPS15
NUMB	EPS15
LAPTM5	EPS15
ITSN1	EPS15
GRB2	EPS15
GJA1	EPS15
FCHO2	EPS15
EPN2	EPS15
EPN1	EPS15
ELF3	EPS15
EGFR	EPS15
DNM2	EPS15
DNM1	EPS15
CRK	EPS15
CDC40	EPS15
AP2A2	EPS15
AP2A1	EPS15
AP1B1	EPS15
AGFG2	EPS15
AGFG1	EPS15
UBC	EPRS
SYNCRIP	EPRS
RIPK3	EPRS
RELB	EPRS
RARS	EPRS
NFKB2	EPRS
NELFCD	EPRS
MARS	EPRS
MAP3K7	EPRS
MAP3K14	EPRS
LARS	EPRS
KARS	EPRS
IKBKB	EPRS
IARS	EPRS

HSP90AA1	EPRS
ARL4D	EPRS
AIMP2	EPRS
UBC	EPPK1
TNFRSF1B	EPPK1
TNFRSF1A	EPPK1
MAP3K3	EPPK1
MAP3K1	EPPK1
EGFR	EPPK1
VAV1	EPOR
UBC	EPOR
SYK	EPOR
STAT5B	EPOR
STAT5A	EPOR
PTPN6	EPOR
PLCG2	EPOR
PLCG1	EPOR
PIK3R1	EPOR
MAPK1	EPOR
EPOR	LYN
KIT	EPOR
JAK2	EPOR
IRS2	EPOR
EPOR	INPP5D
GRB2	EPOR
GRAP	EPOR
GNB2L1	EPOR
GAB2	EPOR
EPOR	CSF2RB
CRKL	EPOR
ATXN2L	EPOR
GNB2L1	EPO
EPOR	EPO
UBC	EPN3
UBC	EPN2
RNF11	EPN2
LAPTM5	EPN2
UBC	EPN1
EGFR	EPN1
AP2A1	EPN1
UBC	EPM2A
NHLRC1	EPM2A
UBC	EPHX1
UBC	EPHB4

EFNB2 EPHB4
FYN EPHB3
CRK EPHB3
UBC EPHB1
PDGFRB EPHB1
EPHB6 EPHB1
EFNB2 EPHB1
EPHB1 EFNA5
EFNA1 EPHB1
ACP1 EPHB1
PIK3CG EPHA8
EFNA5 EPHA8
UBC EPHA7
EPHA7 EFNA1
EFNA5 EPHA6
EFNA3 EPHA6
EFNA1 EPHA6
EFNA1 EPHA5
UBC EPHA4
FYN EPHA4
EFNB2 EPHA4
EFNA5 EPHA4
EFNA3 EPHA4
EFNA1 EPHA4
EFNB2 EPHA3
EFNA5 EPHA3
EFNA1 EPHA3
UBC EPHA2
TNFAIP1 EPHA2
SLAEPHA2
PTPN11 EPHA2
PIK3R1 EPHA2
EFNA5 EPHA2
EFNA4 EPHA2
EFNA3 EPHA2
EFNA1 EPHA2
CBL EPHA2
UBC EPHA1
EFNA1 EPHA1
UBC EPCAM
MRGBP EPC1
MORF4L1 EPC1
KAT5 EPC1
ING3 EPC1

DMAP1 EPC1
STX12 EPB42
SPINK7 EPB42
SLC4A2 EPB42
YWHAG EPB41L3
YWHABEPB41L3
EPB41L3 UNC13A
UBC EPB41L3
EPB41L3 IGSF21
EPB41L3 CNTNAP2
EPB41L3 CEP152
UBC EPB41L2
EPB41L2 NUMA1
EPB41L2 GRIA1
EPB41L2 DRD1
UBC EPB41L1
NUMA1 EPB41L1
GRIA1 EPB41L1
UBC EPB41
TJP2 EPB41
SLC4A1 EPB41
KPNA2 EPB41
GYPC EPB41
EPB42 EPB41
DRD2 EPB41
VHL EPAS1
UBC EPAS1
SUMO1 EPAS1
SP1 EPAS1
EPAS1 EGLN3
EPAS1 EGLN1
EPAS1 ARNT2
ARNT EPAS1
TRRAP EP400
EP400 TBC1D4
RUVBL2 EP400
MYC EP400
ZRANB2 EP300
EP300 ZNF148
EP300 ZBTB17
YY1 EP300
XRCC6 EP300
VHL EP300
EP300 USF2

UBE2I	EP300
UBC	EP300
EP300	TWIST1
TSG101	EP300
TRIP4	EP300
EP300	TP73
TP53	EP300
EP300	TFAP2A
TDG	EP300
EP300	TCF4
TCF3	EP300
EP300	TCF12
TBP	EP300
EP300	TAL1
TADA3	EP300
SUB1	EP300
STAT6	EP300
EP300	STAT5B
EP300	STAT5A
EP300	STAT3
EP300	STAT2
STAT1	EP300
EP300	SS18L1
SREBF2	EP300
SREBF1	EP300
EP300	SP3
SP1	EP300
SNIP1	EP300
SMAD7	EP300
SMAD5	EP300
SMAD4	EP300
SMAD3	EP300
SMAD2	EP300
SMAD1	EP300
SKP2	EP300
SIRT2	EP300
SIRT1	EP300
EP300	SENP3
RUNX2	EP300
EP300	RELA
EP300	RB1
RARA	EP300
RAD23A	EP300
EP300	PPARG

EP300	PPARA
POLR2A	EP300
EP300	PELP1
EP300	PCNA
PARP1	EP300
EP300	NR4A1
EP300	NOTCH1
NFYB	EP300
EP300	NFYA
NFKB1	EP300
NFATC2	EP300
EP300	NEUROD1
NCOA6	EP300
NCOA3	EP300
NCOA2	EP300
NCOA1	EP300
NBN	EP300
NAP1L1	EP300
EP300	MYOD1
MYC	EP300
MYBL2	EP300
MYB	EP300
EP300	MEF2D
EP300	MEF2C
EP300	MEF2A
MDM4	EP300
MDM2	EP300
EP300	MAX
EP300	MAML1
EP300	MAF
EP300	LEF1
KLF5	EP300
EP300	KLF4
KAT2B	EP300
EP300	JUND
EP300	JUNB
JUN	EP300
EP300	JMY
IRF3	EP300
EP300	IRF1
ING5	EP300
EP300	ING2
ING1	EP300
EP300	IL12B

EP300 HNF1A
EP300 HMGN2
HIST4H4 EP300
EP300 HIST2H2BE
EP300 HIST2H2AC
EP300 HIST2H2AB
HIST1H3A EP300
EP300 HIST1H2BB
EP300 HIST1H2AE
EP300 HIF1A
EP300 HDAC3
HDAC1 EP300
EP300 HAND2
GTF2B EP300
GRIP1 EP300
GRB2 EP300
EP300 GATA6
EP300 GATA4
EP300 GATA2
EP300 GABPA
FOXO3 EP300
EP300 FOSL1
FOS EP300
EP300 FEN1
ETS2 EP300
ETS1 EP300
ESR2 EP300
ESR1 EP300
EP300 EPAS1
EP300 EID1
EP300 EGR1
EBF1 EP300
E2F5 EP300
DTX1 EP300
DDX5 EP300
EP300 DDX17
EP300 DDIT3
EP300 DDB2
CTNNB1 EP300
CTBP1 EP300
CREBBP EP300
CREB1 EP300
COPS6 EP300
EP300 CITED4

EP300	CITED2
EP300	CEBPB
EP300	CDX2
CDK2	EP300
EP300	CARM1
EP300	CALCOCO1
BRCA1	EP300
ATF4	EP300
APEX1	EP300
EP300	ALX1
EP300	AHR
AGT	ENPEP
ENOX1	SULT1E1
EEF1G	ENOX1
UBC	ENO2
UBC	ENO1
PXN	ENO1
ENO1	PLG
FYN	ENO1
UBC	ENKUR
TGFBR2	ENG
TGFBR1	ENG
TGFB3	ENG
TGFB1	ENG
BMP2	ENG
ACVRL1	ENG
ACVR2A	ENG
ACVR1	ENG
UBC	ENDOG
UBC	ENC1
NFE2L2	ENC1
UBC	EML3
MTMR9	EMILIN1
ITGB1	EMILIN1
SLX4	EME1
EMC2	EMC9
UBC	EMC7
UBC	EMC3
UBC	EMC2
EMC8	EMC2
UBC	ELP5
IKBKAP	ELP4
ELP3	ELP4
ELP2	ELP4

UBC	ELP3
SMAD1	ELP3
IKBKAP	ELP3
ELP2	ELP3
UBC	ELOVL5
UBC	ELOVL1
ATP5C1	ELOF1
FBLN1	ELN
UBC	ELMSAN1
HDAC2	ELMSAN1
HDAC1	ELMSAN1
UBC	ELMOD2
THG1L	ELMOD1
UBC	ELL2
MLLT1	ELL2
AFF4	ELL2
TP53	ELL
SNF8	ELL
ELL	EAF2
UBC	ELK3
CTBP1	ELK3
UBC	ELFN2
UBC	ELF3
NFKB1	ELF3
MED23	ELF3
UBC	ELF2
UBC	ELAVL2
VHL	ELAVL1
UBC	ELAVL1
TNPO2	ELAVL1
TNPO1	ELAVL1
PRKCA	ELAVL1
HNRNPD	ELAVL1
UBC	ELAC2
RING1	ELAC2
NDUFA7	ELAC2
ELAC2	CDYL
ASCC2	ELAC2
XRN2	EIF6
UBC	EIF6
TUFM	EIF6
PRKCB	EIF6
ITGB4	EIF6
GTPBP4	EIF6

GNB2L1	EIF6
CSNK2B	EIF6
UBC	EIF5B
UBC	EIF5
EIF4G1	EIF5
EIF4G3	EIF3J
UBC	EIF4G2
SKIL	EIF4G2
EIF4A2	EIF4G2
EIF4A1	EIF4G2
EIF3A	EIF4G2
UBC	EIF4G1
EIF4G1	EIF4A1
SMAD3	EIF4ENIF1
EIF4E2	EIF4ENIF1
EIF4E2	EIF4EBP3
EIF4E2	EIF4EBP1
VCL	EIF4E
UBC	EIF4E
PML	EIF4E
PABPC1	EIF4E
MKNK2	EIF4E
MKNK1	EIF4E
ELAVL1	EIF4E
EIF4G3	EIF4E
EIF4G1	EIF4E
EIF4ENIF1	EIF4E
EIF4EBP3	EIF4E
EIF4EBP2	EIF4E
EIF4EBP1	EIF4E
EIF4A1	EIF4E
ANKHD1-EIF4EBP3	EIF4E
UBC	EIF4B
PABPC1	EIF4B
EIF4G3	EIF4B
EIF3A	EIF4B
CASP3	EIF4B
YWHAQ	EIF4A3
YWHAG	EIF4A3
UPF1	EIF4A3
UBC	EIF4A3
THRAP3	EIF4A3
EIF4A3	SRSF5
SRSF3	EIF4A3

EIF4A3	SRSF1
EIF4A3	SRRM2
SRRM1	EIF4A3
SMG1	EIF4A3
RPS24	EIF4A3
RPS11	EIF4A3
RNPS1	EIF4A3
MAGOH	EIF4A3
ILF2	EIF4A3
EIF4A3	CWC22
CDC5L	EIF4A3
EIF4A3	CCDC174
EIF4A3	CASC3
UBC	EIF4A2
EIF4G3	EIF4A2
EIF4G1	EIF4A2
CLNS1A	EIF4A2
EIF4G3	EIF4A1
UBC	EIF3M
EXOSC10	EIF3M
EIF3H	EIF3M
EIF3F	EIF3M
EIF3E	EIF3M
EIF3D	EIF3M
EIF3B	EIF3M
EIF3A	EIF3M
SMAD2	EIF3L
EIF3L	DNM2
UBC	EIF3K
EIF3K	EIF3M
EIF3L	EIF3K
EIF3J	EIF3K
EIF3K	EIF3H
EIF3F	EIF3K
EIF3K	EIF3E
EIF3K	EIF3D
EIF3K	EIF3B
EIF3A	EIF3K
EIF3J	EIF3A
EIF3I	THRB
TGFBR2	EIF3I
EIF3I	EIF3F
EIF3I	EIF3A
UBC	EIF3H

EIF3L	EIF3H
EIF3J	EIF3H
EIF3I	EIF3H
EIF3F	EIF3H
EIF3H	EIF3D
EIF3B	EIF3H
EIF3A	EIF3H
CCDC85B	EIF3H
ABI3	EIF3H
UBC	EIF3G
MPHOSPH6	EIF3G
EPB41	EIF3G
EIF5	EIF3G
EIF3K	EIF3G
EIF3I	EIF3G
EIF3H	EIF3G
EIF3F	EIF3G
EIF3E	EIF3G
EIF3D	EIF3G
EIF3B	EIF3G
EIF3A	EIF3G
EIF1B	EIF3G
VCL	EIF3F
EIF3L	EIF3F
EIF3J	EIF3F
EIF3F	EIF3A
UBC	EIF3E
SMAD9	EIF3E
NSF	EIF3E
NPM1	EIF3E
EIF3E	IFIT1
HAP1	EIF3E
EIF3E	GPBP1L1
EIF3L	EIF3E
EIF3J	EIF3E
EIF3I	EIF3E
EIF3E	EIF3H
EIF3F	EIF3E
EIF3E	EIF3D
EIF3E	EIF3B
EIF3A	EIF3E
DDX24	EIF3E
EIF3E	COPS7B
UBC	EIF3D

EIF3L	EIF3D
EIF3J	EIF3D
EIF3I	EIF3D
EIF3F	EIF3D
EIF3A	EIF3D
UBC	EIF3B
RPTOR	EIF3B
EIF3B	EIF5
EIF4A1	EIF3B
EIF3L	EIF3B
EIF3J	EIF3B
EIF3I	EIF3B
EIF3F	EIF3B
EIF3B	EIF3D
EIF3A	EIF3B
KRT7	EIF3A
EIF3L	EIF3A
ZBTB16	EIF2S2
UNC119	EIF2S2
UBC	EIF2S2
TP53	EIF2S2
PRKDC	EIF2S2
PLEKHM1	EIF2S2
NCK1	EIF2S2
EIF2S2	EIF5
EIF2S1	EIF2S2
EIF2S2	EIF2B5
EIF2S2	EIF2B4
EIF2B1	EIF2S2
CSNK2B	EIF2S2
CSNK2A1	EIF2S2
CRMP1	EIF2S2
CCDC90B	EIF2S2
UBC	EIF2S1
EIF2S1	EIF2B1
UBC	EIF2B5
GSK3A	EIF2B5
EIF2B1	EIF2B5
UBC	EIF2B4
EIF2S1	EIF2B4
EIF2B5	EIF2B4
UBC	EIF2B3
STC2	EIF2B3
NDUFB8	EIF2B3

GOLM1	EIF2B3
EIF2B5	EIF2B3
UBC	EIF2B1
NFE2L2	EIF2AK3
HSPA5	EIF2AK3
HSP90AA1	EIF2AK3
EIF2S1	EIF2AK3
DNAJC3	EIF2AK3
UBC	EIF2AK2
TP53	EIF2AK2
TOP1	EIF2AK2
STAT1	EIF2AK2
EIF2AK2	PDGFRB
NOP2	EIF2AK2
ILF3	EIF2AK2
EIF2AK2	ILF2
HSP90AA1	EIF2AK2
HNRNPC	EIF2AK2
HIST1H1C	EIF2AK2
GNL3	EIF2AK2
EIF2AK2	FANCC
EIF2AK2	EIF2S1
EIF2AK2	EBNA1BP2
EIF2AK2	DNAJC3
DHX9	EIF2AK2
CHUK	EIF2AK2
CDC42	EIF2AK2
UBC	EIF2AK1
EIF2AK1	EIF2S1
UBC	EIF2A
UBC	EIF1B
EIF3I	EIF1B
EIF3A	EIF1B
UBC	EIF1AD
STAT1	EIF1AD
UBC	EIF1
EEF1G	EID2B
UBC	EID1
UBC	EI24
EHMT2	UHRF1
EHMT2	H3F3A
UBC	EHHADH
SIAH1	EHHADH
PXN	EHHADH

PEX5	EHHADH
MYC	EHHADH
GRM7	EHHADH
UBC	EHD4
UBC	EHBP1
TP53	EGR1
SUMO1	EGR1
SP1	EGR1
NFATC2	EGR1
EGR1	NFATC1
NAB2	EGR1
NAB1	EGR1
MAPK1	EGR1
JUN	EGR1
EGR1	EGR2
CREBBP	EGR1
CEBPB	EGR1
BAX	EGR1
UBC	EGLN3
SIAH2	EGLN3
EGLN1	EGLN3
SIAH2	EGLN2
MOB4	EGLN2
FAM46A	EGLN2
EGLN3	EGLN2
UBC	EGLN1
SIAH2	EGLN1
OSR1	EGLN1
FKBP8	EGLN1
VAV1	EGFR
UBC	EGFR
EGFR	TNK2
EGFR	STAT1
EGFR	STAM2
EGFR	SOS2
EGFR	SH2B3
EGFR	SEC61A1
EGFR	RIN2
EGFR	PRKCA
EGFR	PRKAR1A
EGFR	PLCG2
EGFR	PDCD6IP
EGFR	MUC1
EGFR	MAP3K14

EGFR	LRIG1
EGFR	KRT7
EGFR	KRT17
EGFR	JAK1
EGFR	INPPL1
EGFR	HTT
EGFR	HSPA8
EGFR	GRB14
EGFR	GAB1
EGFR	FER
EGFR	DOK2
EGFR	CBLB
EGFR	CASP1
EGFR	CAMLG
EGFR	ARF4
EGFR	ABL2
VTN	EGF
EGFR	EGF
EFTUD2	SNRNP40
TRAPPC6A	EFS
EFS	SH2D3C
PTK2B	EFS
BCAR1	EFS
UBC	EFR3A
EPHB3	EFNB3
EFNB3	EPHA4
UBC	EFNB2
EFNB2	EPHB6
EPHB2	EFNB2
EPHA7	EFNA5
EFNA5	EPHA5
EPHA7	EFNA4
EFNA4	EPHA6
EFNA4	EPHA5
EFNA4	EPHA4
EPHA7	EFNA3
EFNA3	EPHA5
EEF1G	EFNA1
UBC	EFHD2
UBC	EFHD1
EFEMP2	ZNF638
EFEMP2	TP53
EFEMP2	SGTB
EFEMP2	PLSCR4

EFEMP2	LOX
GLRX3	EFEMP2
EFEMP2	FAM124B
EFEMP2	FAM115A
EFEMP2	CCNK
EFEMP2	CATSPER1
EFEMP2	BAG6
EFEMP2	AQP1
SGTA	EFEMP1
UBC	EFCAB11
UBC	EEF2
SUMO1	EEF2
RPS6KA1	EEF2
PPP2R1B	EEF2
HSPD1	EEF2
EEF1A1	EEF2
EEF1G	VARS
SKIL	EEF1G
EEF1G	PSMD11
EEF1G	NUDT21
UBC	EEF1E1
MARS	EEF1E1
IARS	EEF1E1
DARS	EEF1E1
AIMP2	EEF1E1
AIMP1	EEF1E1
VARS	EEF1D
UBC	EEF1D
TPT1	EEF1D
SIAH1	EEF1D
RIPK3	EEF1D
KTN1	EEF1D
KARS	EEF1D
HEXDC	EEF1D
EEF1G	EEF1D
CSNK2A1	EEF1D
CRMP1	EEF1D
CDK1	EEF1D
CD48	EEF1D
ASCC2	EEF1D
EEF1B2	VARS
EEF1G	EEF1B2
EEF1A1	EEF1B2
UBC	EEF1A2

TRAF6	EEF1A2
TRAF2	EEF1A2
TNFRSF1B	EEF1A2
TAB2	EEF1A2
RIPK3	EEF1A2
NFKB2	EEF1A2
NFKB1	EEF1A2
NEDD8	EEF1A2
EEF1B2	EEF1A2
EEF1A1	VCL
EEF1A1	SMAD2
EEF1A1	MDM2
EEF1A1	EEF1G
UBC	EED
EED	RBBP4
EDNRB	EDN3
EDNRA	EDN3
EDNRB	EDN1
EDNRA	EDN1
ECE1	EDN1
COPS6	EDN1
BAG6	EDN1
UBC	EDIL3
RPS3A	EDEM2
SEL1L	EDEM1
EDARADD	EDAR
UBC	ECT2
RACGAP1	ECT2
SETDB1	ECSIT
IMMT	ECSIT
UBC	ECHS1
CAPN1	ECHS1
UBC	ECHDC1
SERPINB9	ECH1
PEX5	ECH1
EEF1G	ECH1
UBC	ECE1
UBC	ECD
S100P	ECD
UBC	EBNA1BP2
NOP58	EBNA1BP2
FTSJ3	EBNA1BP2
UBC	EARS2
ELL2	EAF1

ELL	EAF1
CDK9	EAF1
TP53	E4F1
SMAD9	E4F1
HDAC1	E4F1
UBC	E2F6
TFDP1	E2F6
PCGF6	E2F6
EHMT1	E2F6
E2F5	TFDP1
YY1	E2F3
TFDP1	E2F3
SP1	E2F3
RBL1	E2F3
MYBL2	E2F3
E2F3	CDK3
E2F2	TFDP1
SP1	E2F2
UBC	DZIP3
UBC	DYRK2
RAD54B	DYRK2
HSP90AA1	DYRK2
HIST3H3	DYRK2
HIST1H2BO	DYRK2
DYRK1B	MBP
UBC	DYRK1A
UBC	DYNLT1
DYNLT1	HSPB9
UBC	DYNLRB2
UBC	DYNLRB1
MPHOSPH6	DYNLRB1
DYNC1I2	DYNLRB1
UBC	DYNLL2
DYNLL2	THAP10
DYNLL2	MYO5A
DYNLL2	HOMER3
GPRIN2	DYNLL2
DYNLL2	GPHN
DYNLL2	DNAL4
DYNLL2	BMF
DYNLL1	MYO5A
DYNLL1	ME2
DYNLL1	DNM2
DYNLL1	ACTG1

UBC	DYNC2LI1
DYNC2H1	DYNC2LI1
DYNC1LI1	DYNC1LI2
DYNC1H1	DYNC1LI2
UBC	DYNC1LI1
PAFAH1B1	DYNC1I1
DYNLL1	DYNC1I1
UBC	DYNC1H1
PAFAH1B1	DYNC1H1
NFKB2	DYNC1H1
NDEL1	DYNC1H1
DYNLL1	DYNC1H1
DYNC1H1	DYNC1LI1
UBC	DYM
USHBP1	DYDC1
NME5	DYDC1
ZNF512B	DVL3
DVL3	ZNF408
DVL3	ZBTB48
WT1	DVL3
UBC	DVL3
DVL3	TNFAIP8L1
DVL3	SNX22
PRPF3	DVL3
LY6H	DVL3
LNX1	DVL3
KLHL12	DVL3
KAT7	DVL3
DPPA2	DVL3
CTNNB1	DVL3
CSNK1D	DVL3
CCNK	DVL3
DVL3	BAHD1
AXIN1	DVL3
DVL2	ZNF250
UBC	DVL2
DVL2	U2AF2
TRAF2	DVL2
TP53	DVL2
DVL2	TIFA
THAP1	DVL2
DVL2	TCEB3B
SNIP1	DVL2
DVL2	SNF8

DVL2	RNPS1
DVL2	MCRS1
DVL2	DPPA2
DVL2	DDI1
DVL2	CSNK1E
DVL2	CPSF7
DVL2	BAG3
DVL2	AP2M1
DVL2	AP1M1
UBC	DUT
PPARG	DUT
DUT	PPARA
NUDT18	DUT
TGFBR1	DUSP6
DUSP6	MAPK3
DUSP6	MAPK1
UBC	DUSP5
UBC	DUSP4
UBC	DUSP3
UBC	DUSP22
MAPK8	DUSP22
UBC	DUSP2
MAPK14	DUSP2
MAPK1	DUSP2
MAPK9	DUSP16
MAPK8IP1	DUSP16
MAPK14	DUSP16
MAPK1	DUSP16
UBC	DUSP14
CD28	DUSP14
CCDC85B	DUSP13
DUSP12	MBD6
UBC	DUSP1
UBC	DUS3L
UBC	DTYMK
DTX2	VSNL1
UBE2D4	DTX2
UBC	DTX2
TRIP13	DTX2
RFX6	DTX2
PLSCR1	DTX2
NCALD	DTX2
DTX2	HPCAL1
DTX2	ALKBH4

NOTCH2	DTX1
DGCR6L	DTNBP1
ABI3	DTNBP1
DTNB	PPFIBP2
UTRN	DTNA
SYNM	DTNA
SNTB2	DTNA
SNTB1	DTNA
SNTA1	DTNA
KCNJ12	DTNA
DTNBP1	DTNA
UBC	DTL
COPS6	DTL
COPS5	DTL
UBC	DSTN
SMAD9	DSTN
ACTG1	DSTN
ACTB	DSTN
DSP	KRT5
DSP	DSC1
JUP	DSG3
UBC	DSG2
PKP2	DSG2
DSC2	DSG2
DSC1	DSG2
DSG1	KLK5
UBC	DSCR3
UBC	DSCC1
CHTF18	DSCC1
MAGI2	DSCAML1
UBC	DSC3
ZNF512B	DROSHA
UBC	DROSHA
SP1	DROSHA
DGCR8	DROSHA
DDX5	DROSHA
UBC	DRG2
RWDD1	DRG2
UBC	DRG1
GNA12	DRD5
DRD5	GABRG2
KLHL12	DRD4
GNAI3	DRD2
GNAI2	DRD2

DRAP1	TAF9
UBC	DPYSL4
SH3GL3	DPYSL4
UBC	DPYSL3
UBC	DPYSL2
UBC	DPYD
UBC	DPY30
PRPF3	DPY30
DYDC1	DPY30
UBC	DPY19L4
UBC	DPY19L1
LOX	DPT
BAG6	DPT
SH3GL1	DPPA4
DPPA2	DPPA4
CRK	DPPA4
ZNF250	DPPA2
DPPA2	AP2M1
UBC	DPP9
UBC	DPP8
UBC	DPP7
CCL5	DPP4
CCL22	DPP4
ZBTB16	DPM1
UBC	DPM1
ILK	DPM1
DPM3	DPM1
UBC	DPH5
UBC	DPF2
SMARCA2	DPF2
NFKB1	DPF2
RPN1	DPF1
RUVBL2	DPCD
UBC	DPAGT1
SIRT1	DOT1L
DOT1L	MLLT3
MLLT1	DOT1L
DOT1L	HIST2H3C
UBC	DOPEY2
MUSK	DOK7
EGFR	DOK6
EGFR	DOK5
CCDC85B	DOK5
BNIP3L	DOK5

EGFR	DOK4
LCK	DOK2
TECDOK1
DOK1	PTPRH
PIK3R1	DOK1
DOK1	INPP5D
CRKL	DOK1
SMAD2	DOCK8
YWHAG	DOCK7
UBC	DOCK7
UBC	DOCK6
VAV1	DOCK2
LURAP1DOCK10
DOCK1	WASF2
UBC	DOCK1
GRB2	DOCK1
CRK	DOCK1
UBC	DNTTIP2
RXRA	DNTTIP2
UBC	DNTTIP1
HDAC2	DNTTIP1
UBC	DNPEP
ADAMTSL4	DNPEP
RSL24D1	DNMT3L
HDAC1	DNMT3L
DNMT3B	DNMT3L
DNMT3A	DNMT3L
UHRF1	DNMT3B
UBE2I	DNMT3B
UBC	DNMT3B
SUMO1	DNMT3B
SMARCA5	DNMT3B
HDAC1	DNMT3B
EED	DNMT3B
DNMT3A	DNMT3B
DNMT1	DNMT3B
CBX1	DNMT3B
DNMT3A	UHRF1
UBC	DNMT3A
MYC	DNMT3A
DNMT3A	HIST1H3A
DNMT3A	DNMT1
UHRF1	DNMT1
UBC	DNMT1

TSG101	DNMT1
TRIM28	DNMT1
TRIM27	DNMT1
SIRT1	DNMT1
HDAC2	DNMT1
EED	DNMT1
DYNLL1	DNMT1
DNMT1	DAXX
UBC	DNMBP
UBC	DNM3
GRB2	DNM3
MPHOSPH6	DNM2
UBC	DNM1L
GSK3B	DNM1L
UBC	DNM1
SNX9	DNM1
SH3GL2	DNM1
SH3GL1	DNM1
PACSIN3	DNM1
MED31	DNM1
ITSN1	DNM1
GRB2	DNM1
DYRK1A	DNM1
DNM1	ASB13
FHL5	DNAL4
UBC	DNAJC9
UBC	DNAJC8
SF3A2	DNAJC8
SMAD2	DNAJC7
CLTC	DNAJC6
UBC	DNAJC5
CFTR	DNAJC5
UBC	DNAJC3
PRKRIR	DNAJC3
HSPA1A	DNAJC3
UBC	DNAJC21
UBC	DNAJC2
UBC	DNAJC17
UBC	DNAJC16
DRD1	DNAJC14
DDX39B	DNAJC14
UBC	DNAJC13
UBC	DNAJC11
UBC	DNAJC10

HSPA5	DNAJC10
SERPINA3	DNAJC1
HSPA5	DNAJC1
ABHD16A	DNAJC1
TCEA2	DNAJB9
DNAJB9	SLC16A8
PLK1	DNAJB9
MYL6	DNAJB9
DYNLL1	DNAJB9
UBC	DNAJB6
KRT18	DNAJB6
HDAC4	DNAJB6
UBC	DNAJB5
UBC	DNAJB4
UBC	DNAJB3
EWSR1	DNAJB3
TNFRSF1A	DNAJB12
UBC	DNAJB1
SOD1	DNAJB1
NQO1	DNAJB1
MAP3K3	DNAJB1
HSPA8	DNAJB1
FANCC	DNAJB1
DNAJC3	DNAJB1
ZBTB22	DNAJA3
VHL	DNAJA3
UBC	DNAJA3
TNFRSF1B	DNAJA3
TNFRSF1A	DNAJA3
SMAD9	DNAJA3
RELA	DNAJA3
NFKB2	DNAJA3
NFKB1	DNAJA3
JAK2	DNAJA3
HSPA8	DNAJA3
HSPA1A	DNAJA3
FAM131C	DNAJA3
EGFR	DNAJA3
UBC	DNAJA1
TRAF6	DNAJA1
TRAF2	DNAJA1
TRAF1	DNAJA1
TNFRSF1B	DNAJA1
TNFRSF1A	DNAJA1

TAB2	DNAJA1
TAB1	DNAJA1
SUMO2	DNAJA1
RIPK3	DNAJA1
RIPK1	DNAJA1
PTTG1	DNAJA1
PARK2	DNAJA1
NFKB2	DNAJA1
NFKB1	DNAJA1
MAP3K7	DNAJA1
MAP3K14	DNAJA1
LSM2	DNAJA1
HSPA8	DNAJA1
FZR1	DNAJA1
CFTR	DNAJA1
ARL4D	DNAJA1
PAFAH1B1	DNAI1
UBC	DNAH7
UBC	DNA2
RAB3A	DMXL2
USP46	DMWD
USP12	DMWD
UBC	DMWD
PPBP	DMWD
VASP	DMRTB1
UBC	DMPK
PPP1R12A	DMPK
GABARAP	DMPK
CD44	DMP1
RAD54B	DMC1
RAD51	DMC1
SFTPD	DMBT1
DMAP1	YEATS4
VPS72	DMAP1
TSG101	DMAP1
TRRAP	DMAP1
RUVBL2	DMAP1
RUVBL1	DMAP1
RGS6	DMAP1
DMAP1	MORF4L1
KAT5	DMAP1
DMAP1	ING3
HDAC2	DMAP1
DNMT1	DMAP1

DAXX	DMAP1
BRD8	DMAP1
SPEN	DLX5
MSX2	DLX5
MSX2	DLX2
SMAD4	DLX1
UBC	DLST
DLST	RUSC2
DLST	PFN2
DLST	OGDH
DLST	FLII
DLST	CCDC86
NOTCH4	DLL4
NOTCH1	DLL4
UBC	DLL1
NOTCH3	DLL1
NOTCH2	DLL1
ZNF8	DLK1
UBC	DLGAP5
TRAF2	DLGAP5
UBC	DLGAP4
DLG4	DLGAP2
SHANK2	DLGAP1
DYNLL1	DLGAP1
DLG1	DLGAP1
APC	DLGAP1
UBC	DLG5
PFN1	DLG5
DLST	DLG5
CTNNB1	DLG5
CD81	DLG5
BTBD10	DLG5
DLG4	SHANK2
DLG4	SEMA4F
DLG4	SEMA4C
PTK2B	DLG4
PRKCA	DLG4
NOS1	DLG4
DLG4	MAP3K10
MAP1A	DLG4
LYN	DLG4
LRRC1	DLG4
KIF1B	DLG4
KCNJ4	DLG4

KCNJ12	DLG4
KCNA5	DLG4
KCNA4	DLG4
DLG4	KCNA3
DLG4	KCNA2
GRIN2D	DLG4
DLG4	GRIN2C
GRIN2A	DLG4
DLG4	GRIK5
DLG4	GRIK2
DLG4	FZD1
FYN	DLG4
EXOC4	DLG4
ERBB4	DLG4
DYNLL1	DLG4
DLG4	DLGAP1
DLG4	DAP
CD46	DLG4
DLG4	CACNG2
BEGAIN	DLG4
DLG4	ADRB1
NOS1	DLG2
ERBB4	DLG2
DLG1	DLG2
ATP2B2	DLG2
LCK	DLG1
UBC	DLD
DLAT	DLD
ASS1	DLD
UBC	DLAT
DLAT	PDK2
DLAT	PDHX
SMAD9	DKK1
LRP6	DKK1
TRAF3IP1	DISC1
SYNE1	DISC1
SMARCE1	DISC1
DISC1	NDEL1
MAP1A	DISC1
DISC1	KALRN
ITSN1	DISC1
DISC1	IMMT
FEZ1	DISC1
DISC1	EIF3H

CEP63	DISC1
ATF5	DISC1
ATF4	DISC1
DISC1	AKAP9
VCL	DIS3L2
UBC	DIS3L
EXOSC3	DIS3L
EXOSC2	DIS3L
UBC	DIRAS2
UBC	DIRAS1
UBC	DIP2C
UBC	DIP2B
UBC	DIP2A
UBC	DIMT1
DIDO1	WWP2
UBC	DIDO1
DIDO1	HNRNPK
DIDO1	DVL3
UBC	DICER1
TARBP2	DICER1
EIF2AK2	DICER1
ALOX5	DICER1
AGO1	DICER1
UBC	DIAPH3
WBP4	DIAPH1
UBC	DIAPH1
TCERG1	DIAPH1
PRPF40A	DIAPH1
PKD2	DIAPH1
GAS7	DIAPH1
CENPA	DIAPH1
APBB1	DIAPH1
UBC	DIABLO
TRAF2	DIABLO
DIABLO	LTBR
DIABLO	BIRC7
DIABLO	BIRC6
DIABLO	BIRC2
WRN	DHX9
DHX9	SNRPB2
DHX9	NXF1
UBC	DHX40
UBC	DHX38
UBC	DHX35

UBC	DHX33
UBC	DHX32
UBC	DHX30
SKIL	DHX30
EIF2AK2	DHX30
UBC	DHX29
TBC1D17	DHX29
UBC	DHX16
MEGF10	DHX16
UBC	DHX15
DHX15	SUPT16H
DHX15	SNW1
DHX15	SF3A2
DHX15	RBM5
PIN1	DHX15
DHX15	NOP58
DHX15	NOP56
DHX15	EBNA1BP2
UBC	DHRS7B
UBC	DHRS7
PEX5	DHRS4
UBC	DHRS3
UBC	DHRS2
UBC	DHRS13
UBC	DHRS1
MDFI	DHRS1
UBC	DHPS
RPL9	DHPS
NUDT18	DHPS
NIF3L1	DHPS
EIF5A2	DHPS
UBC	DHFR
DHFR	THBS1
DHFR	HSPD1
DHFR	ASGR1
UBC	DHCR7
UBC	DHCR24
UBC	DGUOK
DGKZ	SNTB2
DGKZ	RASGRP1
PRKCA	DGKZ
PLCG1	DGKZ
HRAS	DGKZ
IGSF21	DGKD

KRT19	DGCR6L
KRT15	DGCR6L
SMURF2	DGCR2
LYST	DGCR14
GNB2L1	DGCR14
UBC	DFNA5
UBC	DFFA
DFFA	DFFB
RFWD2	DET1
UBC	DESI1
UBC	DES
S100B	DES
DES	S100A1
DSP	DES
VIMP	DERL2
UBC	DERL2
DERL2	SERPINA1
DERL2	SEL1L
DERL2	AMFR
VIMP	DERL1
VCP	DERL1
SERPINA1	DERL1
SEL1L	DERL1
RNF5	DERL1
FAF2	DERL1
DERL2	DERL1
CFTR	DERL1
APOB	DERL1
AMFR	DERL1
UBC	DERA
RPTOR	DEPTOR
UBC	DEPDC1
UBC	DENR
MARCH5	DENR
UBC	DENND5B
UBC	DENND5A
UBC	DENND4C
YWHAE	DENND4A
UBC	DENND4A
DENND4A	TP73
UBC	DENND3
UBC	DEK
HIST4H4	DEK
AP2A1	DEK

UBC	DEGS1
SERPINF2	DEFA1
DEFA1	DEFA3
DEFA1	C1QB
UBC	DEF8
FADD	DEDD
DEDD	DEDD2
CFLAR	DEDD
CASP3	DEDD
DEDD	CASP10
PEX5	DECR2
ASCC2	DEAF1
UBC	DDX60L
UPF1	DDX6
UBC	DDX6
U2AF2	DDX6
DCP1A	DDX6
UBC	DDX58
DDX58	IRF3
IKBKE	DDX58
UBC	DDX56
DDX56	RRP12
DDX56	GNL2
DDX56	FTSJ3
UBC	DDX55
ESR1	DDX54
UBC	DDX51
HAP1	DDX49
VCL	DDX46
UBC	DDX46
SF3A2	DDX46
UBC	DDX42
SF3A2	DDX42
RECQL5	DDX42
DDX39B	DNM2
DDX39B	ALYREF
DDX39B	DDX39A
ALYREF	DDX39A
ZNF512B	DDX27
UBC	DDX27
PPP1R16A	DDX27
VDAC3	DDX24
UBC	DDX24
TMEM9B	DDX24

SUMO1 DDX24
DDX24 RAE1
DDX24 PRF1
POLA2 DDX24
PIN1 DDX24
DDX24 MKS1
FADD DDX24
DDX24 EIF4A2
DDX24 C9orf9
UBC DDX23
UBC DDX21
NPM1 DDX21
NFKB2 DDX21
MAP3K14 DDX21
UBC DDX20
SNRPG DDX20
DDX20 GEMIN7
GEMIN6DDX20
GEMIN2DDX20
DHX9 DDX20
UBC DDX19B
SKP1 DDX19B
DDX19B RWDD2B
NUP214 DDX19B
DDX19B GNB2L1
DDX19B DCK
DDX19B B3GALT4
UBC DDX19A
UBC DDX18
INADL DDX18
WBP11 DDX17
UBC DDX17
SUMO2 DDX17
SUMO1 DDX17
SF1 DDX17
PIN1 DDX17
LNX1 DDX17
HNRNPA1 DDX17
HDAC1 DDX17
ESR1 DDX17
DDX5 DDX17
UBC DDX11
PFN2 DDX11
KAT7 DDX11

UBC	DDX10
VCL	DDX1
UBC	DDX1
PXN	DDX1
MRE11A	DDX1
HNRNPA1	DDX1
DDX1	C14orf166
UBC	DDTL
UBC	DDR1
SNRNP40	DDR1
UBC	DDOST
TNFRSF1B	DDOST
TNFRSF1A	DDOST
SNRPG	DDIT4L
UBC	DDIT4
DDIT3	TEF
SPOP	DDIT3
RPS3A	DDIT3
NFIL3	DDIT3
MAPK14	DDIT3
MAFK	DDIT3
JUND	DDIT3
HSD17B14	DDIT3
HLF	DDIT3
FOSL2	DDIT3
DDIT3	FOSL1
DDIT3	FOSB
FOS	DDIT3
CREB5	DDIT3
DDIT3	CEBPG
CEBPE	DDIT3
CEBPB	DDIT3
BATF3	DDIT3
DDIT3	BATF
ATF4	DDIT3
UBC	DDHD2
XPC	DDB2
UBC	DDB2
COPS5	DDB2
UBC	DDAH2
TRIP13	DDAH2
WDTC1	DDA1
VPRBP	DDA1
UBC	DDA1

DET1	DDA1
DCAF5	DDA1
DCAF17	DDA1
DCAF11	DDA1
CUL4A	DDA1
COPS6	DDA1
COPS5	DDA1
UBC	DCUN1D1
DAZAP2	DCUN1D1
CUL5	DCUN1D1
CUL2	DCUN1D1
CUL1	DCUN1D1
DCUN1D1	CAND1
WDYHV1	DCTPP1
UBC	DCTPP1
NOA1	DCTPP1
NIF3L1	DCTPP1
UBC	DCTN6
UBC	DCTN5
NINL	DCTN5
UBC	DCTN4
PXN	DCTN4
ACTR1A	DCTN4
UBC	DCTN3
PBX2	DCTN3
DCTN2	DCTN3
UBC	DCTN2
ACTR1A	DCTN2
DCTN1	ZNF8
ZNF512B	DCTN1
UBC	DCTN1
PGAM1	DCTN1
PAFAH1B1	DCTN1
HAP1	DCTN1
DCTN1	DYNC1I1
DCTN1	DYNC1H1
DCTN1	DCTN2
DCTN1	CLIP1
CASP3	DCTN1
ACTR1A	DCTN1
NUDT18	DCTD
DCTD	CEP76
VCL	DCT
UBC	DCPS

DCP2	NAGK
EDC3	DCP1B
DCP1B	DCP2
UBC	DCP1A
EDC3	DCP1A
DCP2	DCP1A
DCP1B	DCP1A
FN1	DCN
DCN	ELN
EGFR	DCN
COL1A1	DCN
UBC	DCK
UBC	DCD
ILK	DCD
SIAH2	DCC
SIAH1	DCC
PTK2	DCC
NTN1	DCC
NCK1	DCC
MAZ	DCC
MAPK3	DCC
CASP9	DCC
CASP3	DCC
APPL1	DCC
UBC	DCBLD2
UBC	DCBLD1
UBC	DCAF8
MDFI	DCAF8
GTPBP3	DCAF8
CUL4A	DCAF8
YWHAG	DCAF7
UBC	DCAF7
DYRK1B	DCAF7
DYRK1A	DCAF7
UBC	DCAF6
UBC	DCAF5
UBC	DCAF4
COPS6	DCAF4
COPS5	DCAF4
UBC	DCAF16
COPS6	DCAF16
UBC	DCAF13
UBC	DCAF12
TGFBR1	DCAF12

UBC	DCAF11
COPS6	DCAF11
COPS5	DCAF11
UBC	DCAF10
TCEA2	DCAF10
TNFRSF1B	DBT
PXN	DBT
UBC	DBR1
TEF	DBP
JUN	DBP
HLF	DBP
DDIT3	DBP
CEBPG	DBP
CEBPB	DBP
CEBPA	DBP
BATF3	DBP
BATF	DBP
UBC	DBNL
SH2D4A	DBNL
ZNF598	DBN1
UBC	DBN1
RIPK3	DBN1
DBN1	NIPBL
MAP3K3	DBN1
UBC	DBI
PPCDC	DBI
ORC2	DBF4
MCM7	DBF4
MCM4	DBF4
MCM2	DBF4
CDC7	DBF4
UBC	DAZAP1
DAZL	DAZAP1
UBC	DAXX
DAXX	TP73
TP63	DAXX
TP53	DAXX
DAXX	TGFBR2
SNW1	DAXX
DAXX	RASSF1
MDM2	DAXX
MCRS1	DAXX
MAP3K5	DAXX
DAXX	HIPK2

DAXX	HIPK1
HDAC2	DAXX
DAXX	H3F3A
DAXX	ETS1
DAXX	CFLAR
UBC	DARS
MARS	DARS
MAP3K7	DARS
KARS	DARS
IARS	DARS
HSP90AA1	DARS
EEF1A1	DARS
AIMP2	DARS
AIMP1	DARS
AARS	DARS
ERBB3	DAPP1
UBC	DAPK3
DAXX	DAPK3
DAPK1	TNFRSF1A
DAPK1	FADD
UBC	DAP3
TNFRSF10A	DAP3
PPARA	DAP3
NFKB2	DAP3
HSP90AA1	DAP3
FADD	DAP3
HDAC1	DACT1
DVL2	DACT1
DACH1	NAGK
TRAF2	DAB2IP
RIPK1	DAB2IP
MAP3K5	DAB2IP
TGFBR2	DAB2
SMURF2	DAB2
SMAD2	DAB2
DAB1	APP
UBC	DAAM1
CYTH1	CYTIP
UBC	CYTH3
CNKSR1	CYTH3
UBC	CYTH2
TRIM23	CYTH2
ARRB2	CYTH2
ARRB1	CYTH2

ARF6	CYTH2
UBC	CYTH1
ITGB2	CYTH1
UBC	CYR61
ITGB5	CYR61
ITGAV	CYR61
UBC	CYP51A1
AMFR	CYP3A4
FANCG	CYP2E1
UBC	CYP1B1
FANCG	CYP19A1
FDX1	CYP11B2
CYP11A1	CYP11B2
SMAD2	CYP11A1
CYP11A1	FDX1
UBC	CYLD
TRAF6	CYLD
TRAF2	CYLD
RIPK1	CYLD
MAVS	CYLD
IKBKE	CYLD
DDX58	CYLD
WASF2	CYFIP2
WASF1	CYFIP2
UBC	CYFIP2
TCERG1	CYFIP2
PRPF40A	CYFIP2
NCKAP1	CYFIP2
GAS7	CYFIP2
FXR2	CYFIP2
FXR1	CYFIP2
APBB1	CYFIP2
ABI1	CYFIP2
CYCS	VDAC2
VCL	CYCS
UBC	CYCS
SNCA	CYCS
CYCS	HSPB1
CYCS	FDX1
CYCS	CYB5R3
CYCS	CYB5R1
CYCS	CYB5A
CASP9	CYCS
CYCS	BCL2L1

APAF1	CYCS
UBC	CYB5R3
CYB5R3	CYB5A
TRIP13	CYB5R2
UBC	CYB5R1
UBC	CYB5B
UBC	CYB5A
SCD	CYB5A
CYB5A	CYP4A11
CYB5A	CYP1A1
UBC	CYB561
SKIL	CXXC5
WDR82	CXXC1
WDR5	CXXC1
CCL5	CXCR5
CCL3	CXCR5
PPBP	CXCR2
GRIA1	CXCR2
GNA15	CXCR2
CXCR2	CXCL5
CXCR2	CXCL3
CXCR2	CXCL2
CXCR2	CXCL1
GNA15	CXCR1
CXCR1	CXCL2
CXCR1	CXCL1
DPP4	CXCL9
CCR3	CXCL9
MMP9	CXCL6
CXCR2	CXCL6
CXCR1	CXCL6
MMP9	CXCL5
MMP1	CXCL5
CXCR1	CXCL5
CXCR1	CXCL3
PLG	CXCL2
DPP4	CXCL2
CXCR6	CXCL16
EEF1G	CXCL13
CXCL13	CXCR5
CXCL13	CCL19
FN1	CXCL12
DPP4	CXCL12
CXCR4	CXCL12

DPP4	CXCL11
CCR3	CXCL11
VCAN	CXCL10
NFKB1	CXCL10
IRF7	CXCL10
IRF1	CXCL10
IGFBP7	CXCL10
DPP4	CXCL10
CCR3	CXCL10
UBC	CXADR
CX3CR1	CX3CL1
UBC	CWF19L1
TOM1L1	CWF19L1
CCDC85B	CWC25
CTNNBL1	CWC15
UBC	CUX1
SUMO2	CUX1
SOX2	CUX1
SATB1	CUX1
RB1	CUX1
KAT2B	CUX1
CDC25A	CUX1
UBC	CUTC
NUDT18	CUTC
ASCC2	CUTC
UBC	CUTA
UBE2L3	CUL9
UBC	CUL9
TP53	CUL9
UBC	CUL7
TP53	CUL7
SKP1	CUL7
OBSL1	CUL7
CUL9	CUL7
CUL5	UBE2F
UBC	CUL5
CUL5	TGFBR1
TCEB2	CUL5
TCEB1	CUL5
CUL5	SOCS6
SMAD2	CUL5
CUL5	RNF7
CUL5	RAB40C
CUL5	RAB40B

CUL5	PCMTD2
CUL5	NEDD8
HSPA1A	CUL5
CUL5	HSP90AA1
CUL5	GPR75-ASB3
CUL5	FEM1B
CUL5	COPS7A
COPS6	CUL5
CUL5	COPS5
CUL5	COPS4
CUL5	CBFB
CUL5	CAND1
CUL5	ASB7
CUL5	ASB2
CUL5	ASB1
CUL4A	WDTC1
VPRBP	CUL4A
UBXN7	CUL4A
UBE2E3	CUL4A
UBE2D3	CUL4A
UBC	CUL4A
TRPC4AP	CUL4A
TOR1AIP2	CUL4A
SKP1	CUL4A
RFWD2	CUL4A
CUL4A	PWP1
PHIP	CUL4A
NEDD8	CUL4A
MDM2	CUL4A
HSPA1A	CUL4A
HSP90AA1	CUL4A
CUL4A	GRWD1
CUL4A	GPS1
ERCC8	CUL4A
DTL	CUL4A
CUL4A	DET1
DDB2	CUL4A
DCUN1D1	CUL4A
CUL4A	DCAF6
CUL4A	DCAF5
CUL4A	DCAF4
CUL4A	DCAF16
CUL4A	DCAF12
CUL4A	DCAF11

CUL4A	DCAF10
CUL4A	CRBN
CUL4A	COPS8
CUL4A	COPS7B
COPS7A	CUL4A
COPS6	CUL4A
COPS5	CUL4A
COPS4	CUL4A
COPS2	CUL4A
CDKN1B	CUL4A
CUL4A	CAND1
CUL3	WNK4
CUL3	UBXN7
UBE2D2	CUL3
UBC	CUL3
TCEB1	CUL3
SPOP	CUL3
CUL3	SHKBP1
SF3B3	CUL3
CUL3	RHOBTB1
CUL3	RCBTB1
PSMD2	CUL3
CUL3	NFE2L2
NEDD8	CUL3
NCOA3	CUL3
CUL3	MYEOV2
CUL3	KLHL9
CUL3	KLHL7
CUL3	KLHL25
CUL3	KLHL22
CUL3	KLHL20
CUL3	KLHL18
KLHL12	CUL3
CUL3	KLHL11
CUL3	KCTD6
CUL3	KCTD10
CUL3	KBTBD7
CUL3	KBTBD6
CUL3	HSPD1
CUL3	HSP90AB1
CUL3	HSP90AA1
CUL3	GAN
ESR1	CUL3
CUL3	ENC1

DCUN1D1	CUL3
COPS7A	CUL3
COPS6	CUL3
COPS5	CUL3
COPS4	CUL3
CUL3	COPS2
CUL3	CDC34
CCNE1	CUL3
CUL3	CAND1
BTBD2	CUL3
CUL3	BTBD10
CUL3	BTBD1
CUL3	ACBD3
CUL3	ABTB2
CUL2	TCEB2
CUL2	COMMD1
CUL1	YBX1
CUL1	UBE2E3
UBC	CUL1
SNRNP70	CUL1
SKP1	CUL1
CUL1	SF3B3
CUL1	SENP8
CUL1	RNF7
CUL1	RAC2
CUL1	PSMB4
CUL1	PSMA7
CUL1	PSMA6
CUL1	PSMA2
CUL1	NFKBIB
CUL1	NFKBIA
CUL1	MYEOV2
CUL1	FTSJ3
CUL1	FBXW7
CUL1	FBXO7
CUL1	FBXO6
CUL1	FBXO42
CUL1	FBXO31
CUL1	FBXO27
CUL1	FBXO25
CUL1	FBXO18
CUL1	FBXL5
CUL1	FAF1
EEF1G	CUL1

CUL1	COPS7A
COPS6	CUL1
CUL1	COPS5
CUL1	COPS2
CUL1	COMMD1
CUL1	CDC34
CUL1	CDC25A
CUL1	CAND2
UBC	CUEDC1
SMURF2	CUEDC1
SCGB1A1	CUBN
CUBN	GIF
CUBN	GC
ALB	CUBN
URM1	CTU1
ZRANB1	CTTNBP2NL
UBC	CTTNBP2NL
STK25	CTTNBP2NL
STK24	CTTNBP2NL
PPP2CB	CTTNBP2NL
MOB4	CTTNBP2NL
ZRANB1	CTTNBP2
WASL	CTTN
UBC	CTTN
TJP1	CTTN
SYK	CTTN
GRB2	CTTN
FER	CTTN
DNM2	CTTN
DNM1	CTTN
ASAP1	CTTN
CTTN	ACTR2
ACTA1	CTTN
UBC	CTSZ
SERPINB4	CTSS
SERPINB3	CTSS
PLCG1	CTSS
KNG1	CTSS
CST5	CTSS
CST3	CTSS
CTSG	SERPINA3
SERPINA1	CTSG
CTSG	PPBP
CTSG	F2RL2

INS	CTSE
UBC	CTSD
SERPINA4	CTSD
PSAP	CTSD
FN1	CTSD
CTSA	CTSD
CSTB	CTSD
APP	CTSD
UBC	CTSB
SPHK1	CTSB
CTSB	SERPINB13
CTSB	MARCKS
CTSB	CSTB
CTSB	CSTA
BAG6	CTSB
ANXA2	CTSB
UBC	CTSA
SERPINA3	CTRL
SERPINA3	CTRC
CTR9	WDR61
UBC	CTR9
SUPT16H	CTR9
SSRP1	CTR9
SMAD9	CTR9
LEO1	CTR9
CTR9	CNTNAP2
CDC73	CTR9
CTR9	BLK
UBC	CTPS1
UBC	CTNND2
PSEN1	CTNND2
CTNND2	PDZD2
LCK	CTNND2
ERBB2IP	CTNND2
ABL1	CTNND2
UBC	CTNND1
CTNND1	FYN
CTNND1	CDH3
UBC	CTNNBL1
SPP1	CTNNBL1
CTNNBL1	AICDA
UBC	CTNNBIP1
JUP	CTNNBIP1
CTNNB1	CTNNBIP1

BEND5 CTNNBIP1
ZFYVE9 CTNNB1
YAP1 CTNNB1
WWTR1 CTNNB1
UBC CTNNB1
CTNNB1 TRRAP
TOP2A CTNNB1
TGFBR1 CTNNB1
CTNNB1 TCF7L1
CTNNB1 TCF7
TCF3 CTNNB1
CTNNB1 TAX1BP3
SMAD4 CTNNB1
SMAD2 CTNNB1
CTNNB1 SLC9A3R1
SKP1 CTNNB1
RUVBL2 CTNNB1
RUVBL1 CTNNB1
CTNNB1 PYGO1
CTNNB1 PTPRZ1
CTNNB1 PTPRM
PTPRF CTNNB1
CTNNB1 PTPRC
PTPN6 CTNNB1
CTNNB1 PTPN14
CTNNB1 PSEN1
CTNNB1 PLK1
CTNNB1 PKP2
CTNNB1 PITX2
PIN1 CTNNB1
PIK3R1 CTNNB1
CTNNB1 NR5A2
CTNNB1 NR5A1
CTNNB1 NFKB1
NCOA2 CTNNB1
MYC CTNNB1
CTNNB1 MUC1
MEN1 CTNNB1
CTNNB1 MAGI2
CTNNB1 LEF1
KAT2A CTNNB1
JUP CTNNB1
CTNNB1 IQGAP1
HNRNPA1 CTNNB1

CTNNB1	HNF1A
HDAC7	CTNNB1
HDAC1	CTNNB1
GSK3B	CTNNB1
FLT1	CTNNB1
CTNNB1	FAF1
CTNNB1	EZR
ERBB2IP	CTNNB1
CTNNB1	EP400
EGFR	CTNNB1
DVL2	CTNNB1
CTNNB1	DSC3
CTNND1	CTNNB1
CTNNB1	CTNNA3
CTNNB1	CTNNA1
CTNNB1	CSNK1D
CTNNB1	CSNK1A1
CTNNB1	CHUK
CHD8	CTNNB1
CTNNB1	CEBPA
CTNNB1	CDK5R1
CTNNB1	CDK5
CTNNB1	CDH8
CTNNB1	CDH5
CTNNB1	CDH3
CTNNB1	CDH2
CTNNB1	CDH15
CTNNB1	CDH1
CDC73	CTNNB1
CTNNB1	CBY1
CTNNB1	CACYBP
CTNNB1	CA9
CTNNB1	BCL9
CTNNB1	AXIN2
AXIN1	CTNNB1
CTNNB1	APC2
CTNNB1	APC
UBC	CTNNAL1
NEK1	CTNNAL1
AKAP13	CTNNAL1
UBC	CTNNA2
VCL	CTNNA1
UBC	CTNNA1
TJP2	CTNNA1

DLG1	CTNNA1
CDH5	CTNNA1
CDH3	CTNNA1
CDH2	CTNNA1
APC	CTNNA1
ACTN1	CTNNA1
PIK3R1	CTLA4
LCK	CTLA4
FYN	CTLA4
AP2M1	CTLA4
AP1M1	CTLA4
TGFB3	CTGF
TGFB1	CTGF
UBC	CTDSPL2
MBP	CTDSP1
GTF2F1	CTDSP1
CDCA3	CTDSP1
WDR77	CTDP1
CTDP1	POLR2J
UBC	CTC1
TEN1	CTC1
OBFC1	CTC1
ZNF512B	CTBP2
ZNF217	CTBP2
CTBP2	ZFPM2
CTBP2	ZFPM1
ZEB1	CTBP2
UBC	CTBP2
CTBP2	TGIF1
CTBP2	TEAD3
NRIP1	CTBP2
NOL4	CTBP2
MDM2	CTBP2
CTBP2	KLF3
FHL3	CTBP2
ELAC2	CTBP2
EHMT2	CTBP2
CTBP1	CTBP2
CBX4	CTBP2
BRCA1	CTBP2
CTBP2	BAZ2B
AKTIP	CTBP2
CTBP1	ZNF217
UBC	CTBP1

CTBP1	LCOR
CTBP1	KAT2B
CTBP1	EHMT2
CTBP1	EHMT1
CTBP1	APC
UBQLN1	CSTF2T
UBC	CSTF2T
UBC	CSTF1
UBC	CSTB
UBC	CSTA
CTSB	CST7
CTSB	CST3
UBC	CSRP2
UBC	CSRP1
PPP1CA	CSRNP2
UBC	CSPP1
TNK2	CSPG4
CSNK2B	ZNHIT1
CSNK2B	USO1
UBC	CSNK2B
CSNK2B	TP53
CSNK2B	SUPT16H
CSNK2B	SSRP1
CSNK2B	RPL5
CSNK2B	RPL41
CSNK2B	RNF7
CSNK2B	RNF2
CSNK2B	PRKCZ
CSNK2B	PRKAB2
CSNK2B	PIN1
CSNK2B	NOLC1
CSNK2B	NFKBIA
CSNK2B	MBD4
CSNK2B	LYST
CSNK2B	LYN
CSNK2B	HSP90AA1
CSNK2B	GADD45GIP1
CSNK2B	FIBP
CSNK2B	FGF1
CSNK2B	FAF1
CSNK2B	CSNK2A2
CSNK2B	CDC34
CSNK2B	CALM1
CSNK2B	BRCA1

UBC CSNK2A2
CSNK2A2 TGFBR1
CSNK2A2 STX1A
CSNK2A2 SNCA
RNPS1 CSNK2A2
RNF2 CSNK2A2
CSNK2A2 RGS19
PIN1 CSNK2A2
CSNK2A2 PCGF3
CSNK2A2 PAK1
MAPK14 CSNK2A2
CSNK2A2 HSPH1
CSNK2A2 HSP90B1
CSNK2A2 HSP90AA1
CSNK2A2 HMGA1
HDAC2 CSNK2A2
CSNK2A2 FOS
CSNK2A2 FGF1
CSNK2A2 ERH
CSNK2A2 EIF4EBP1
CSNK2A2 EIF3H
CSNK2A2 EEF1B2
CSNK2A2 CSN3
CSNK2A2 ATF1
CSNK2A2 ACACA
YWHAB CSNK2A1
XRCC1 CSNK2A1
VHL CSNK2A1
CSNK2A1 VAMP4
UBC CSNK2A1
TP53 CSNK2A1
TOP2A CSNK2A1
SUB1 CSNK2A1
STX1A CSNK2A1
STMN1 CSNK2A1
SSRP1 CSNK2A1
SSB CSNK2A1
SRF CSNK2A1
SPIB CSNK2A1
SNCA CSNK2A1
SIRT1 CSNK2A1
SIN3A CSNK2A1
CSNK2A1 RRAD
RPL5 CSNK2A1

RNF7	CSNK2A1
RGS19	CSNK2A1
RELA	CSNK2A1
PTPN1	CSNK2A1
PTEN	CSNK2A1
CSNK2A1	PSEN2
PML	CSNK2A1
PIN1	CSNK2A1
PAK1	CSNK2A1
PACS1	CSNK2A1
NCL	CSNK2A1
CSNK2A1	NAP1L4
MAX	CSNK2A1
CSNK2A1	LIG1
CSNK2A1	LGALS3
LEO1	CSNK2A1
JUN	CSNK2A1
HSPH1	CSNK2A1
HSP90B1	CSNK2A1
HSP90AA1	CSNK2A1
HNRNPA2B1	CSNK2A1
HMGA1	CSNK2A1
CSNK2A1	HIRIP3
HDAC3	CSNK2A1
HDAC2	CSNK2A1
HDAC1	CSNK2A1
CSNK2A1	HCLS1
GMFB	CSNK2A1
FIBP	CSNK2A1
FGF1	CSNK2A1
FAF1	CSNK2A1
EIF4EBP1	CSNK2A1
EEF1B2	CSNK2A1
CSNK2A1	DDIT3
CTNNB1	CSNK2A1
CSNK2B	CSNK2A1
CSNK2A1	CSN2
CSNK2A1	CSN1S1
CREBBP	CSNK2A1
COPS2	CSNK2A1
CDC34	CSNK2A1
CALM1	CSNK2A1
UBC	CSNK1G3
UBC	CSNK1G2

COL4A3BP CSNK1G2
UBC CSNK1G1
CSNK1E SDCCAG3
CSNK1E MCC
CSNK1E APC
UBC CSNK1D
TP53 CSNK1D
SNCA CSNK1D
SMAD2 CSNK1D
MDM2 CSNK1D
CSNK1D AKAP9
UBC CSNK1A1L
YWHAE CSNK1A1
UBC CSNK1A1
TP53 CSNK1A1
TNFRSF1B CSNK1A1
SNCA CSNK1A1
RCC1 CSNK1A1
PPP2R5A CSNK1A1
MDM2 CSNK1A1
KPNA2 CSNK1A1
KPNA1 CSNK1A1
HSP90AA1 CSNK1A1
HMGB2 CSNK1A1
ERF CSNK1A1
CSNK1A1 CHRM3
CSNK1A1 AP3B1
CSN2 CSN3
COPS7A CSN3
UBC CSMD3
UBC CSK
SRC CSK
SIT1 CSK
CSK SDC3
RGS16 CSK
RASA1 CSK
PXN CSK
PTPRC CSK
PTPN12 CSK
CSK PLD2
PAG1 CSK
LCK CSK
JUN CSK
IGF1R CSK

HNRNPK	CSK
GJA1	CSK
FYN	CSK
FGR	CSK
CSK	DOK3
CD44	CSK
CD247	CSK
CBL	CSK
CAV1	CSK
ARRB1	CSK
SMAD2	CSH1
UBC	CSF3R
JAK1	CSF3R
HCK	CSF3R
CSF3R	CSF3
PTPN6	CSF2RB
MAD2L1	CSF2RB
LCK	CSF2RB
JAK1	CSF2RB
GNB2L1	CSF2RB
SDC2	CSF2
PRG2	CSF2
FOS	CSF2
CSF3R	CSF2
CSF2RB	CSF2
UBC	CSF1R
CSF1R	FYN
TNF	CSF1
SOCS1	CSF1
CSF1R	CSF1
YWHAG	CSE1L
UBC	CSE1L
TNFRSF1B	CSE1L
SUMO2	CSE1L
STAT1	CSE1L
CSE1L	RPL22
RAN	CSE1L
KPNA4	CSE1L
KPNA2	CSE1L
HNRNPL	CSE1L
CSE1L	CNDP2
STT3A	CSDE1
UBC	CS
TAB2	CS

MDH2 CS
DYNLL1 CS
UBC CRYZ
CRYAA CRYZ
CRYAA CRYGD
CRYGC CRYBB2
CRYGC CRYAA
CRYBA1 CRYBB1
VEGFA CRYAB
UBC CRYAB
TP53 CRYAB
SNCA CRYAB
HSPB1 CRYAB
CTNNB1 CRYAB
CS CRYAB
CRYGD CRYAB
CRYGC CRYAB
CRYBB2 CRYAB
CRYBA1 CRYAB
CRYAA CRYAB
AVPCRYAB
APPCRYAB
CRYAA MIP
CRYAA LALBA
CRYBB2 CRYAA
UBC CRY2
TIMELESS CRY1
CSNK1ECRY1
CBLB CRY1
CRX NRL
CRX M1AP
UBC CRTC2
CRTC2 SIK2
UBC CRTAP
SNRNP70 CRP
NFKB1 CRP
FCN2 CRP
FCGR2BCRP
FCGR2A CRP
CRMP1 ZNF24
VIM CRMP1
TRIP13 CRMP1
TK1CRMP1
CRMP1 TFG

SNRPG	CRMP1
SERPINB9	CRMP1
SEPHS1	CRMP1
RTN4	CRMP1
RGL2	CRMP1
PSMD11	CRMP1
PPP1R8	CRMP1
PLA2G2A	CRMP1
PFN1	CRMP1
CRMP1	PAFAH1B3
CRMP1	NVL
CRMP1	NAT9
MRPS12	CRMP1
MOB4	CRMP1
LSM2	CRMP1
LRRC1	CRMP1
CRMP1	KLHL20
CRMP1	IL33
HSPE1	CRMP1
CRMP1	HNRNPUL1
HNRNPH3	CRMP1
CRMP1	FXR1
CRMP1	FUBP1
CRMP1	FTH1
FAS	CRMP1
EPN1	CRMP1
CRMP1	DPYSL2
DDX18	CRMP1
CDK5RAP3	CRMP1
CDK5RAP2	CRMP1
CCT7	CRMP1
CCL18	CRMP1
CCDC106	CRMP1
BTBD2	CRMP1
AXIN1	CRMP1
ALDH2	CRMP1
CRMP1	AGR2
UBC	CRKL
CRKL	TYK2
CRKL	TGFB1I1
CRKL	STAT5B
CRKL	STAT5A
PXN	CRKL
CRKL	PIK3R2

CRKL	PDGFRA
CRKL	MAP4K5
CRKL	KIT
CRKL	IFNAR1
CRKL	GAB2
CRKL	GAB1
ERBB3	CRKL
EGFR	CRKL
CRKL	DOCK2
CRKL	CRK
CRKL	CD34
CRKL	CBLB
VAV1	CRK
UBC	CRK
CRK	STAT5B
CRK	SOS2
CRK	SHB
CRK	SH3BP1
PXN	CRK
CRK	PDGFRB
CRK	MAP4K5
CRK	KIT
CRK	GAB1
CRK	FLT1
EGFR	CRK
CRK	DOCK3
CRK	BUB1
ATXN1	CRK
CRK	ABL2
TUBB2A	CRIPT
DLG4	CRIPT
DLG2	CRIPT
DLG1	CRIPT
SH3GL3	CRIP1
HAP1	CRIP1
UBC	CRIM1
UCN3	CRHR2
UCN	CRHR2
UCN	CRHBP
CREM	FHL5
TBP	CREG1
RBL2	CREG1
RBL1	CREG1
RB1	CREG1

CREBZFNFE2L1
CREBBP YWHAH
CREBBP WT1
UBC CREBBP
CREBBP TP73
TP53 CREBBP
CREBBP TDG
CREBBP TCF3
CREBBP STAT6
CREBBP STAT3
CREBBP STAT2
CREBBP STAT1
CREBBP SS18L1
CREBBP SRF
CREBBP SREBF1
SP1 CREBBP
SNIP1 CREBBP
CREBBP SMARCB1
SMAD2 CREBBP
SMAD1 CREBBP
RPS6KA1 CREBBP
CREBBP RELA
CREBBP PPARG
CREBBP PELP1
CREBBP PCMT1
CREBBP ONECUT1
CREBBP NUP98
CREBBP NLK
CREBBP NKX2-1
CREBBP NFE2L2
NFATC4 CREBBP
NFATC2 CREBBP
CREBBP NEUROG1
NCOA6 CREBBP
NCOA3 CREBBP
NCOA2 CREBBP
NCOA1 CREBBP
MYBL2 CREBBP
CREBBP MYBL1
CREBBP MYB
CREBBP MSX1
MDM2 CREBBP
CREBBP MDC1
CREBBP MAML1

KHDRBS1	CREBBP
CREBBP	KAT6A
CREBBP	KAT2B
CREBBP	IRF3
CREBBP	IRF1
CREBBP	IFNB1
CREBBP	IFI6
CREBBP	HTT
CREBBP	HOXA9
CREBBP	HNF4A
CREBBP	HNF1A
CREBBP	HMGA1
HIST4H4	CREBBP
HIST1H3A	CREBBP
CREBBP	HIPK2
HDAC2	CREBBP
HDAC1	CREBBP
CREBBP	GTF2B
GLI3	CREBBP
CREBBP	GCM1
FOXO3	CREBBP
FOXO1	CREBBP
CREBBP	FOS
ETS2	CREBBP
CREBBP	ETS1
ESR1	CREBBP
EBF1	CREBBP
CREBBP	CTNNB1
CTBP1	CREBBP
CSNK2A2	CREBBP
CREBBP	CREB1
CREBBP	CNTN2
CREBBP	CHUK
CREBBP	CDC20
CREBBP	CARM1
CREBBP	BRCA1
CREBBP	ATXN3
CREBBP	ANAPC7
CREBBP	AIRE
UBC	CREB5
THRA	CREB5
NFE2L1	CREB5
MAFK	CREB5
JUND	CREB5

JUNB	CREB5
FOSL2	CREB5
CREB5	FOSL1
CREB5	FOSB
FOS	CREB5
CREB5	CEBPG
CEBPE	CREB5
CEBPB	CREB5
BATF3	CREB5
CREB5	BATF
ATF4	CREB5
UBC	CREB3L4
CREB3L3	CREB3L4
CREB3L1	CREB3L4
CEBPG	CREB3L4
BATF3	CREB3L4
UBC	CREB3L2
SUMO2	CREB3L2
NFIL3	CREB3L1
CREB3L3	CREB3L1
BATF3	CREB3L1
STX8	CREB3
MALL	CREB3
DDIT3	CREB3
CREB3L3	CREB3
CREB3L1	CREB3
CEBPG	CREB3
APPBP2	CREB3
VIM	CREB1
UBC	CREB1
TP53	CREB1
RPS6KA4	CREB1
PRKG1	CREB1
HTT	CREB1
CREB1	HPS6
HDAC2	CREB1
CREB1	GTF2F2
GLI2	CREB1
CREB1	FHL5
CREM	CREB1
C14orf1	CREB1
ATM	CREB1
ATF1	CREB1
VIM	CRCT1

IGSF21	CRCT1
UBC	CRBN
COPS6	CRBN
INADL	CRB1
PEX5	CRAT
C14orf1	CRADD
UBC	CRABP2
TP53	CR2
IFNA1	CR2
FCER2	CR2
CR2	CR1
CD19	CR2
NUDT21	CPSF7
UBC	CPSF6
CPSF6	TCERG1
CPSF6	NUDT21
NEDD4	CPSF6
CPSF6	KLHL12
CPSF6	ITCH
UBC	CPSF3
UBC	CPSF2
CPSF2	PPARGC1A
CPSF3	CPSF2
UBC	CPS1
UBC	CPOX
UBC	CPNE9
UBC	CPNE8
CALCOCO2	CPNE7
AK5	CPNE6
UBC	CPNE5
UBC	CPNE3
UBC	CPNE2
ACTB	CPNE2
UBC	CPNE1
MAP2K1	CPNE1
CPNE4	CPNE1
ACTB	CPNE1
EGF	CPM
SNAP25	CPLX1
RPA1	CPE
CPE	ROBO2
POLA2	CPE
CPE	PMCH
UBC	CPD

UBC	COX7A2L
UBC	COX7A2
UBC	COX6C
PTN	COX6C
UBC	COX6B1
UBC	COX4I1
RBM48	COX17
C14orf1	COX17
CORT	SSTR4
CORT	SSTR2
SSTR1	CORT
CORT	GHSR
UBC	CORO7-PAM16
VCL	CORO2B
TSC22D1	CORO2B
UBC	CORO1B
PRKCA	CORO1B
ARPC2	CORO1B
UBC	CORO1A
ACTL6B	COQ9
UBC	COQ2
UBC	COPZ1
COPB1	COPZ1
UBC	COPS8
RFWD2	COPS8
GPS1	COPS8
COPS8	GFER
DDB2	COPS8
CUL5	COPS8
CUL1	COPS8
COPS7A	COPS8
COPS6	COPS8
COPS5	COPS8
COPS4	COPS8
COPS2	COPS8
UBC	COPS7B
GPS1	COPS7B
COPS6	COPS7B
COPS5	COPS7B
COPS4	COPS7B
COPS2	COPS7B
UBC	COPS7A
COPS7A	TOM1L1
COPS7A	DDB2

CUL2	COPS7A
COPS6	COPS7A
COPS7A	COPS5
UBC	COPS6
COPS6	TRIB3
COPS6	TOR1AIP2
COPS6	TDGF1
COPS6	TCEB2
COPS6	SULT1E1
COPS6	STX5
COPS6	SNRPG
COPS6	SERPINB9
COPS6	RFWD2
COPS6	RFC5
COPS6	PTEN
COPS6	PSMD11
COPS6	PSAP
PHYHIP	COPS6
COPS6	PBX2
COPS6	MAP7D1
COPS6	KLHL9
COPS6	KLHL22
COPS6	ERH
COPS6	DDB2
CUL2	COPS6
COPS6	COX5A
COPS6	COX17
COPS6	COPS5
COPS6	BTBD9
BTBD2	COPS6
COPS6	BTBD1
COPS5	UCHL1
UBC	COPS5
TP53	COPS5
SMAD2	COPS5
COPS5	S100A7
COPS5	KLHL9
COPS5	KLHL24
COPS5	KLHL22
CUL2	COPS5
COPS5	CHUK
BTBD2	COPS5
COPS5	BTBD1
UBC	COPS4

COPS4	GPS1
COPS4	DDB2
CUL2	COPS4
CUL1	COPS4
COPS7A	COPS4
COPS6	COPS4
COPS5	COPS4
COPS4	COPS2
CCDC85B	COPS4
VPRBP	COPS3
UBC	COPS3
NEDD8	COPS3
ITGB2	COPS3
HIF1A	COPS3
GPS1	COPS3
ERCC8	COPS3
DDB2	COPS3
COPS3	DDA1
CUL5	COPS3
CUL4A	COPS3
CUL3	COPS3
CUL2	COPS3
CUL1	COPS3
COPS3	COPS8
COPS3	COPS7B
COPS7A	COPS3
COPS6	COPS3
COPS5	COPS3
COPS4	COPS3
COPS2	COPS3
UBC	COPS2
THRB	COPS2
RXRA	COPS2
RFWD2	COPS2
COPS2	DDB2
CUL2	COPS2
COPS7A	COPS2
COPS6	COPS2
COPS5	COPS2
UBC	COPG2
DRD1	COPG2
COPZ1	COPG2
COPG1	COPG2
COPE	COPG2

COPB1	COPG2
UBC	COPG1
DRD1	COPG1
COPG1	COPZ2
COPZ1	COPG1
COPE	COPG1
COPB1	COPG1
COPB1	COPE
UBC	COPB2
COPB2	RGS4
PRKCE	COPB2
NFKB1	COPB2
COPZ1	COPB2
COPB2	COPG2
COPB2	COPG1
COPE	COPB2
COPB1	COPB2
RHOQ	COPB1
KCNK3	COPB1
COPB1	HLA-G
UBD	COPA
UBC	COPA
STX8	COPA
SLC25A11	COPA
PDGFRB	COPA
COPZ1	COPA
COPG2	COPA
COPG1	COPA
COPE	COPA
COPB2	COPA
COPB1	COPA
ARCN1	COPA
XRN2	COMT
UBC	COMT
TRIP13	COMT
LITAF	COMT
ACE	COMT
UBC	COMMD8
UBC	COMMD6
RELA	COMMD6
COMMD1	COMMD6
ZNF337	COMMD3
UBC	COMMD3
COMMD8	COMMD3

HAP1	COL9A2
VCL	COL8A1
PXN	COL8A1
KLHL12	COL8A1
ITGA2	COL8A1
ITGA1	COL8A1
EFEMP2	COL8A1
COL8A2	COL8A1
PXN	COL6A3
ITGB1	COL6A3
UBC	COL6A2
PXN	COL6A2
ABCD3	COL6A2
UBC	COL6A1
PXN	COL6A1
LGALS3BP	COL6A1
LAMB1	COL6A1
FN1	COL6A1
COL5A1	COL5A3
COL1A2	COL5A3
PXN	COL5A2
UBC	COL5A1
PXN	COL5A1
LGALS3BP	COL5A1
TGFBI	COL4A4
OSM	COL4A4
MMP9	COL4A4
MATN2	COL4A4
HABP2	COL4A4
FN1	COL4A4
FBLN2	COL4A4
DCN	COL4A4
CD93	COL4A4
ANTXR2	COL4A4
VHL	COL4A3
UBC	COL4A3
OSM	COL4A3
MMP9	COL4A3
MATN2	COL4A3
ITGB3	COL4A3
FN1	COL4A3
DCN	COL4A3
CD47	COL4A3
APP	COL4A3

VHL	COL4A2
SKIL	COL4A2
COL4A2	OSM
MMP9	COL4A2
FN1	COL4A2
COL4A2	FBLN2
DCN	COL4A2
COL4A1	COL4A2
APP	COL4A2
COL4A1	SERPINE2
FN1	COL4A1
DDR2	COL3A1
DDR1	COL3A1
TNFRSF10A	COL2A1
FN1	COL2A1
APP	COL25A1
SPARC	COL1A2
PXN	COL1A2
COL1A2	P4HB
MMP9	COL1A2
COL1A2	ITGA2
FN1	COL1A2
DCN	COL1A2
COL1A2	CD93
CD44	COL1A2
CD36	COL1A2
SPARC	COL1A1
DDR2	COL1A1
CD36	COL1A1
NCL	COL18A1
KDR	COL18A1
GPC1	COL18A1
FBLN2	COL18A1
DCN	COL14A1
SPARC	COL13A1
COL13A1	ITGA1
UBC	COIL
TAF9	COIL
NOLC1	COIL
ATXN1	COIL
UBC	COG8
COG3	COG8
UBC	COG7
COG4	COG7

UBC	COG5
COG7	COG5
COG4	COG5
COG3	COG7
COG3	COG6
COG4	COG3
COG3	COG2
UBC	COG2
COG4	COG2
UBC	COG1
COG8	COG1
COG4	COG1
COG3	COG1
UBC	COASY
KRT20	COASY
UBC	COA1
UBC	CNTROB
PXN	CNTROB
UBC	CNTRL
CNTNAP2	ZMIZ1
CNTN2	CNTNAP2
RTN4	CNTNAP1
PTPRB	CNTNAP1
UBC	CNTN2
NCAN	CNTN2
KAT2B	CNTN2
CHUK	CNTN2
CNTN1	TNR
SCNN1B	CNTN1
CNTN1	CNTN2
UBC	CNTLN
PLSCR1	CNTFR
LIFR	CNTFR
IL6ST	CNTFR
CRLF1	CNTFR
CNTF	CNTFR
CLCF1	CNTFR
APPBP2	CNTFR
LIFR	CNTF
IL6ST	CNTF
PPP1CA	CNST
GNA15	CNR2
GNA15	CNR1
ARL4D	CNPY4

UBC	CNPY2
UBC	CNP
TUBA1A	CNP
UBC	CNOT8
CNOT4	CNOT8
CNOT2	CNOT8
BTG3	CNOT8
UBC	CNOT7
TNRC6C	CNOT6L
CNOT7	CNOT6L
CNOT1	CNOT6L
UBC	CNOT6
RQCD1	CNOT6
CNOT8	CNOT6
CNOT7	CNOT6
CNOT2	CNOT6
CNOT1	CNOT6
UBC	CNOT4
UBC	CNOT3
TNRC6C	CNOT3
CNOT8	CNOT3
CNOT2	CNOT3
UBC	CNOT11
UBC	CNOT10
TNRC6C	CNOT10
UBC	CNOT1
TNRC6C	CNOT1
CNOT1	CNOT8
CNOT7	CNOT1
CNOT4	CNOT1
CNOT2	CNOT1
UBC	CNN3
LCP1	CNN3
UBC	CNN2
SMAD4	CNKSR1
UBC	CNIH4
ABCA4	CNGB1
SLC24A1	CNGA1
UBC	CNDP2
UBC	CNBP
UBC	CMTM6
UBC	CMSS1
UBC	CMPK1
UBC	CMIP

UBC	CMAS
SERPINA3	CMA1
UBC	CLUH
UBC	CLUAP1
UBC	CLU
CLU	TGFBR2
CLU	PRNP
CLU	PON1
CLU	COMMD1
CLU	C8B
CLU	BCL2L1
CLU	BAX
CLU	APP
UBC	CLTCL1
TNFRSF1A	CLTCL1
UBC	CLTC
TRAF2	CLTC
TOM1L1	CLTC
CLTC	TOM1
TNK2	CLTC
TNFRSF1B	CLTC
TNFRSF1A	CLTC
TANK	CLTC
TAB2	CLTC
CLTC	STAMBP
NFKB2	CLTC
NFKB1	CLTC
NCALD	CLTC
LDLRAP1	CLTC
KIT	CLTC
IKBKE	CLTC
HIP1R	CLTC
CLTC	EPN1
CLINT1	CLTC
CLTC	C10orf88
ARRB2	CLTC
AP1G1	CLTC
UBC	CLTB
HIP1R	CLTB
HIP1	CLTB
CSNK2A2	CLTB
CLTC	CLTB
UBC	CLTA
HIP1R	CLTA

CLTC	CLTA
CLSTN1	SLK
APP	CLSTN1
CLSTN1	APBA2
FZR1	CLSPN
CLPX	CLPP
CLPS	LIPF
UBC	CLPP
SMAD9	CLPB
UBC	CLP1
UBC	CLN6
UBC	CLN5
XPO1	CLN3
UBC	CLN3
CLN3	SLC25A11
CLN3	SLC25A10
PRKDC	CLN3
NUP205	CLN3
IMMT	CLN3
CLN3	HSD17B12
GCN1L1	CLN3
CLN3	DDOST
CPT1A	CLN3
CLN3	COX15
AUP1	CLN3
YWHAG	CLK3
UBC	CLK3
CLK2	CLK3
YWHAG	CLK2
FANCM	CLK2
PPIG	CLK1
CLK1	MBP
UBC	CLIP3
PAFAH1B1	CLIP1
UBC	CLIC4
ACTB	CLIC4
UBC	CLIC1
PTPN6	CLEC4A
NINL	CLEC1B
TJP2	CLDN1
MMP2	CLDN1
INADL	CLDN1
CLDN1	CLDN3
UBC	CLCN7

GOPC	CLCN3
CRLF1	CLCF1
UBC	CLCC1
ITGB4	CLCA2
UBC	CLASP2
YWHAG	CLASP1
UBC	CLASP1
UBC	CKS2
TSC22D4	CKS2
CKS2	CCNA2
CKMT2	OLFML3
UBC	CKM
TRIM63	CKM
UBC	CKAP5
TACC1	CKAP5
PTPRN2	CKAP5
VCL	CKAP4
UBC	CKAP4
PXN	CKAP4
ASCC2	CKAP4
UBC	CKAP2L
CITED2	TFAP2C
CITED2	LHX2
CREBBP	CITED2
UBC	CIT
UBC	CISD2
UTP15	CIRH1A
UBC	CIRH1A
SRSF2	CIR1
NCOR2	CIR1
DFFA	CIDEB
ATXN1	CIC
MYOD1	CIB2
UBR5	CIB1
UBC	CIB1
TERT	CIB1
CIB1	RAC3
CIB1	PSEN2
PRKDC	CIB1
CIB1	PLK3
PLK2	CIB1
ITGB3	CIB1
FEZ1	CIB1
EXOSC10	CIB1

UBC	CIAPIN1
GLRX3	CIAPIN1
UBC	CIAO1
FAM96A	CIAO1
TUBB	CHUK
TRAF1	CHUK
TNFRSF1A	CHUK
NFKB2	CHUK
NCOR2	CHUK
CHUK	MYO1D
CHUK	MYH9
MYC	CHUK
BCL10	CHUK
UBC	CHTOP
DSCC1	CHTF8
SFN	CHST1
CHRNA5	CHRNB4
CHRNA4	CHRNB4
CHRNA3	CHRNB4
CHRNA4	CHRNB2
COPS6	CHRNB1
PIK3R1	CHRNA7
UBC	CHRNA5
CHRNA4	VSNL1
CHRNB4	CHRNA2
CHRNB2	CHRNA2
ITGA7	CHRNA1
CHRNG	CHRNA1
CHRNE	CHRNA1
CHRND	CHRNA1
CHRM2	SAG
GNA15	CHRM2
GNA14	CHRM2
BMP1	CHRD
SMARCA5	CHRAC1
POLE3	CHRAC1
SKIL	CHPF
UBC	CHP1
STK17B	CHP1
SLC9A2	CHP1
CHP1	SLC9A1
UBC	CHORDC1
RPS3A	CHN1
GTF3C1	CHN1

VPS25 CHMP6
SNF8 CHMP6
CHMP4B CHMP6
VTA1 CHMP5
UBC CHMP5
STAMBP CHMP5
APPBP2 CHMP5
USP8 CHMP4C
PDCD6IP CHMP4C
CHMP4B CHMP4C
UBC CHMP4B
CHMP4B PDCD6IP
VPS4A CHMP4A
CHMP4A STAMBP
PDCD6IP CHMP4A
TRAF2 CHMP2B
VPS4B CHMP2A
VPS4A CHMP2A
UBC CHMP2A
STAMBP CHMP2A
RNF103-CHMP3 CHMP2A
VPS4B CHMP1B
USP8 CHMP1B
UBC CHMP1B
VPS4B CHMP1A
VPS4A CHMP1A
USP8 CHMP1A
UBC CHMP1A
STAMBP CHMP1A
CHMP1B CHMP1A
RAB3A CHML
RAB1A CHML
UBC CHID1
UBC CHIC2
U2AF2 CHIC2
PLEKHF2 CHIC2
CATSPER1 CHIC2
CHGB PTEN
CHGB POLR2E
CHGB POLD1
CHGB CBFB
PLGCHGA
CHFR WDR47
UBE2N CHFR

UBE2D2	CHFR
UBE2D1	CHFR
UBC	CHFR
CHFR	GABBR1
EEF1G	CHFR
BRD4	CHFR
UBC	CHERP
SF3A2	CHERP
UBC	CHEK2
TP53	CHEK2
PLK1	CHEK2
NBN	CHEK2
MDM2	CHEK2
MDC1	CHEK2
CDC25A	CHEK2
ATM	CHEK2
XRCC6	CHEK1
UBC	CHEK1
TP53	CHEK1
TLK1	CHEK1
SMAD4	CHEK1
RELA	CHEK1
PRKDC	CHEK1
MDM4	CHEK1
HSP90AB1	CHEK1
HSP90AA1	CHEK1
DTL	CHEK1
DNMT1	CHEK1
CUL4A	CHEK1
CUL1	CHEK1
CSNK2B	CHEK1
CSNK2A1	CHEK1
CLSPN	CHEK1
CDC25C	CHEK1
CDC25B	CHEK1
CDC25A	CHEK1
BRCA1	CHEK1
ATR	CHEK1
ATM	CHEK1
SUMO2	CHD9
UBC	CHD7
CHD7	SOX2
ZFYVE9	CHD6
UBC	CHD5

CHD4	RBBP4
CHD4	MTA2
CHD3	YAE1D1
CHD3	XRCC4
UBC	CHD3
CHD3	TXNDC9
TRIM28	CHD3
TP53	CHD3
CHD3	TNFRSF14
CHD3	TAL1
SUMO1	CHD3
SKIL	CHD3
CHD3	SIRT6
CHD3	SERF2
CHD3	SERBP1
RBBP4	CHD3
RAD51	CHD3
PUF60	CHD3
PSME1	CHD3
CHD3	PRG2
PAICS	CHD3
MTA2	CHD3
CHD3	MAN2A2
KDM1A	CHD3
CHD3	IVNS1ABP
IK	CHD3
HSPH1	CHD3
HIST1H3A	CHD3
HDAC2	CHD3
HDAC1	CHD3
HABP4	CHD3
CHD3	FUBP1
CHD3	FBP2
CHD3	CREB1
CHD3	CRCT1
CPE	CHD3
CHD4	CHD3
BHLHE40	CHD3
CHD3	ATPIF1
CHD3	ADH5
SUMO1	CHD2
UBC	CHD1L
SRSF3	CHD1
UBC	CHCHD6

UBC	CHCHD3
CCDC85B	CHCHD3
UBC	CHAF1B
HIST1H3A	CHAF1B
CHAF1A	CHAF1B
ASF1B	CHAF1B
ASF1A	CHAF1B
ITGB1	CHAD
COL2A1	CHAD
YWHAG	CGN
UBC	CGN
TJP2	CGN
TJP1	CGN
SUV39H2	CGGBP1
CGA	TSHB
PTPN12	CGA
PTNCGA	
LHB	CGA
CGB	CGA
CFTR	VCP
UBC	CFTR
CFTR	STUB1
SLC9A3R2	CFTR
CFTR	SLC9A3R1
CFTR	RNF5
PRKAA1	CFTR
CFTR	PPP2R1B
HSPA8	CFTR
GOPC	CFTR
AMFR	CFTR
CFTR	AHSA1
UBC	CFLAR
TRAF2	CFLAR
TRAF1	CFLAR
TP53	CFLAR
TNFSF10	CFLAR
TNFRSF10B	CFLAR
TNFRSF10A	CFLAR
ITCH	CFLAR
FADD	CFLAR
CFLAR	DEDD2
UBC	CFL2
ACTG1	CFL2
ACTB	CFL2

UBC	CFL1
TESK2	CFL1
TESK1	CFL1
SSH1	CFL1
CFL1	MYCBP
CFL1	LIMK1
HSPH1	CFL1
H2AFX	CFL1
CFL1	ATP1A1
ARRB2	CFL1
CFL1	ARRB1
ACTG1	CFL1
ACTC1	CFL1
ACTB	CFL1
CFL1	ACTA2
ACTA1	CFL1
SELL	CFH
CRP	CFH
CFI	CFH
UBC	CFDP1
LPL	CETP
UBC	CETN3
CETN3	PDE6D
UBC	CETN1
GUSB	CES1
UBC	CERS6
UBC	CERK
UBC	CERCAM
UBC	CEP89
UBC	CEP78
PAICS	CEP76
UBC	CEP70
CEP70	TCEB3B
CEP70	SUV39H2
CEP70	RCOR3
CEP70	KAT7
CEP70	KANSL1
CCDC85B	CEP70
UBC	CEP68
UBC	CEP57
UBC	CEP55
TSG101	CEP55
PDCD6IP	CEP55
ZNF250	CEP44

MCRS1	CEP44
CEP44	ABI3
UBC	CEP350
UBC	CEP290
CCP110	CEP290
UBC	CEP250
UBC	CEP164
TTBK2	CEP164
UBC	CEP152
UBC	CEP128
UBC	CEP120
UBC	CENPQ
UBC	CENPP
CENPN	CENPO
UBC	CENPK
YWHAG	CENPJ
STAT5B	CENPJ
STAT5A	CENPJ
UBC	CENPH
UBC	CENPF
NDEL1	CENPF
CENPE	CENPF
BRAP	CENPF
UBC	CENPE
NUF2	CENPE
BUB1B	CENPE
UBC	CENPB
RUVBL1	CENPA
NPM1	CENPA
HIST4H4	CENPA
HIST1H3A	CENPA
H2AFY	CENPA
CENPN	CENPA
CENPA	CENPM
CENPA	CENPH
CENPB	CENPA
SWAP70	CELSR3
SGSM2	CELSR3
UBC	CELSR2
PHF10	CELSR2
PSAP	CELSR1
UBC	CELF2
UBC	CELF1
TOM1L1	CELF1

CELA1	LPA
A2M	CELA1
UBC	CECR5
UBC	CEBPZ
TP73	CEBPZ
TP53	CEBPZ
NFYB	CEBPZ
UBC	CEBPG
TEF	CEBPG
NFIL3	CEBPG
MLLT6	CEBPG
MAFK	CEBPG
HLF	CEBPG
FOSL2	CEBPG
FOSL1	CEBPG
FOS	CEBPG
CREB3L3	CEBPG
CREB3L1	CEBPG
CEBPB	CEBPG
BATF3	CEBPG
BATF	CEBPG
ATF5	CEBPG
ATF4	CEBPG
CEBPE	CEBPG
CEBPB	CEBPE
CEBPA	CEBPE
BATF3	CEBPE
CEBPE	BATF
ATF4	CEBPE
UBC	CEBPD
SUMO2	CEBPD
RELA	CEBPD
RB1	CEBPD
HDAC3	CEBPD
HDAC1	CEBPD
DDIT3	CEBPD
CEBPG	CEBPD
CEBPE	CEBPD
CEBPB	CEBPD
CEBPA	CEBPD
UBC	CEBPB
TRIM28	CEBPB
STAT6	CEBPB
CEBPB	STAT5A

SRF	CEBPB
SPI1	CEBPB
SP1	CEBPB
SMARCA2	CEBPB
SMAD4	CEBPB
NOLC1	CEBPB
NFKB1	CEBPB
MED23	CEBPB
CEBPB	MAPK1
CEBPB	IL1B
CEBPB	HMGA1
HDAC1	CEBPB
FOXO1	CEBPB
EGFR	CEBPB
CEBPB	CRP
BATF3	CEBPB
CEBPB	BATF
ATF5	CEBPB
UBC	CEBPA
SPI1	CEBPA
RFC1	CEBPA
MSX2	CEBPA
CEBPA	MRTO4
CEBPA	HNF1A
CEBPA	GRIN3A
CEBPA	CEBPG
CEBPB	CEBPA
CEBPA	CDKN3
CDK4	CEBPA
BATF3	CEBPA
CEBPA	BATF
ATF5	CEBPA
ATF4	CEBPA
CEACAM6	CEACAM8
CEACAM1	CEACAM8
EWSR1	CEACAM5
PXN	CEACAM1
CEACAM1	CEACAM6
ANXA2	CEACAM1
UBC	CDYL
HDAC2	CDYL
EHMT2	CDYL
PAX6	CDX2
HNF1A	CDX2

CREBBP	CDX2
CDX2	ACAT2
UBC	CDS2
UBC	CDS1
UBC	CDKN3
CDC25A	CDKN3
TBC1D17	CDKN2D
CDK6	CDKN2D
TP53	CDKN2C
RBM48	CDKN2C
DRAP1	CDKN2C
COPS6	CDKN2C
CDKN2C	CDK6
CDKN2C	CCDC90B
CDKN2B	IKBKAP
CDKN2B	CDK8
CDKN2B	CCDC90B
UBC	CDKN2AIP
MDM2	CDKN2AIP
VHL	CDKN2A
UBC	CDKN2A
TRIP12	CDKN2A
TP53	CDKN2A
SERTAD1	CDKN2A
PSMC3	CDKN2A
PPP1R9B	CDKN2A
PIAS2	CDKN2A
ORC4	CDKN2A
NPM1	CDKN2A
MYC	CDKN2A
MDM2	CDKN2A
MCM5	CDKN2A
MCM2	CDKN2A
MCM10	CDKN2A
HAUS1	CDKN2A
GMNN	CDKN2A
CDKN2AIP	CDKN2A
CDK6	CDKN2A
CDK5RAP3	CDKN2A
CDK4	CDKN2A
CDC7	CDKN2A
CDC6	CDKN2A
CDC5L	CDKN2A
CDC45	CDKN2A

CCND2	CDKN2A
ACTG1	CDKN2A
UBC	CDKN1C
YWHAE	CDKN1B
YWHAB	CDKN1B
CDKN1B	UCHL1
UBC	CDKN1B
TRAF2	CDKN1B
CDKN1B	SPDYA
SKP1	CDKN1B
CDKN1B	NUP50
CDKN1B	KPNA5
CDKN1B	KPNA2
CDKN1B	GRB2
CDKN1B	COPS5
CDKN1B	CDK6
CDKN1B	CDK5
CDKN1B	CDK4
CDKN1B	CDC34
CDKN1B	CCNE1
CDKN1B	CCNA2
CDKN1B	ARHGDIA
UBC	CDKN1A
TSG101	CDKN1A
TP53	CDKN1A
TK1	CDKN1A
STAT3	CDKN1A
SLC25A11	CDKN1A
SKP2	CDKN1A
SET	CDKN1A
RB1	CDKN1A
PSMA3	CDKN1A
POLD2	CDKN1A
PIM1	CDKN1A
PCNA	CDKN1A
PARP1	CDKN1A
NMRK2	CDKN1A
MDM4	CDKN1A
MDM2	CDKN1A
MCM10	CDKN1A
MAPK8	CDKN1A
HDAC11	CDKN1A
HDAC1	CDKN1A
HAUS1	CDKN1A

GNB2L1	CDKN1A
GMNN	CDKN1A
GET4	CDKN1A
GADD45G	CDKN1A
GADD45B	CDKN1A
GADD45A	CDKN1A
ESR1	CDKN1A
EP300	CDKN1A
ELAVL1	CDKN1A
DTL	CDKN1A
CSNK2B	CDKN1A
CREBBP	CDKN1A
CEBPA	CDKN1A
CDKN1B	CDKN1A
CDK6	CDKN1A
CDK5	CDKN1A
CDK4	CDKN1A
CDK2	CDKN1A
CDK1	CDKN1A
CDC7	CDKN1A
CDC6	CDKN1A
CDC5L	CDKN1A
CDC45	CDKN1A
CCNE2	CDKN1A
CCNE1	CDKN1A
CCND3	CDKN1A
CCND2	CDKN1A
CCNB2	CDKN1A
CCNB1	CDKN1A
CCNA2	CDKN1A
CCNA1	CDKN1A
CCDC85B	CDKN1A
CASP3	CDKN1A
C1orf123	CDKN1A
BCCIP	CDKN1A
BAG6	CDKN1A
UBC	CDKAL1
CDK9	ZMYM6
UBC	CDK9
TRIM28	CDK9
SKP2	CDK9
CDK9	RB1
PPP1CC	CDK9
PIN1	CDK9

NBN	CDK9
MYC	CDK9
MLLT1	CDK9
CDK9	MEPCE
CDK9	MED21
CDK9	MBP
CDK9	HSPD1
HSPA5	CDK9
HSPA1A	CDK9
CDK9	HSP90AB1
CDK9	HSP90AA1
CDK9	HNRNPA2B1
CDK9	HEXIM1
CDK9	GTF2F1
CDK9	FKBP5
CDK9	ELL2
CUL1	CDK9
CDK9	CTDP1
CDK7	CDK9
CDK9	CDK5R1
CDK9	CDC37
CCT8	CDK9
CCT5	CDK9
CCT4	CDK9
CDK9	CCT3
CDK9	CCNT2
CCNK	CDK9
CDK9	BRD4
CDK9	ATP1A1
CDK9	AFF4
AFF1	CDK9
SREBF1	CDK8
MED1	CDK8
UBC	CDK7
TP53	CDK7
CDK7	RARA
CDK7	GTF2H3
CDK7	ERCC3
CDK7	CCNH
CDK7	CCNA2
UBC	CDK6
CDK6	CDC37
CCNE1	CDK6
UBC	CDK5RAP3

CDK5RAP3	DDRGK1
CDK5RAP2	STARD9
CDK5RAP2	RBM48
CDK5RAP2	HAP1
CDK5RAP2	EEF1G
CDK5RAP2	C14orf1
UBC	CDK5RAP1
TSC22D4	CDK5R1
PAK1	CDK5R1
CDK5R1	NES
CDK5RAP3	CDK5R1
CDK5RAP2	CDK5R1
CDK5RAP1	CDK5R1
CDK5	CDK5R1
UBC	CDK5
TP53	CDK5
CDK5	NES
MAPT	CDK5
CDK5	CDK5R2
CDK5	CDC37
CCNG1	CDK5
CDK5	CABLES2
APP	CDK5
ACTN1	CDK5
UBC	CDK4
SMAD2	CDK4
CDK4	RFC1
CDK4	RBL1
PPP1CA	CDK4
MYC	CDK4
CDK4	MCM2
HSPA8	CDK4
CDK4	CDKN2D
CDK4	CDKN2C
CDKN2B	CDK4
CDK4	CDK6
CDK4	CDC7
CDK4	CDC37
BAG6	CDK4
UBC	CDK3
E2F2	CDK3
CDK3	CDKN3
CCNE1	CDK3
CDK3	CCNC

CCNA2	CDK3
WARS	CDK2AP2
CDK2AP1	CDK2AP2
HDAC2	CDK2AP1
HDAC1	CDK2AP1
CDK2	CDK2AP1
CDK2	UBTF
UBC	CDK2
CDK2	TUFM
TP53	CDK2
CDK2	TOP1
TCP1	CDK2
CDK2	TAGLN2
CDK2	SUB1
CDK2	SPDYA
SMAD3	CDK2
SMAD2	CDK2
CDK2	SLC25A3
SKP2	CDK2
SKP1	CDK2
S100A8	CDK2
RPS3A	CDK2
RPS3	CDK2
CDK2	RPS27A
CDK2	RPS27
CDK2	RPS23
CDK2	RPS19
CDK2	RPS18
CDK2	RPS16
CDK2	RPS14
CDK2	RPS11
CDK2	RPL35
CDK2	RPL23
CDK2	RPL22
CDK2	RPL19
RPA1	CDK2
CDK2	RBL2
CDK2	RBL1
CDK2	RB1
RAN	CDK2
CDK2	PSMA6
PRKDC	CDK2
CDK2	PRKCH
CDK2	PRDX3

CDK2	PHGDH
PGR	CDK2
ORC2	CDK2
NPM1	CDK2
CDK2	NPAT
MYBL2	CDK2
CDK2	MS4A3
CDK2	MCM4
CDK2	MCM3
CDK2	MCM2
CDK2	LYN
CDK2	LIG1
CDK2	KAT2B
CDK2	HSPD1
HSPA9	CDK2
HSPA8	CDK2
HSPA5	CDK2
CDK2	HSP90AB1
CDK2	HSP90AA1
CDK2	HRNR
HIST4H4	CDK2
CDK2	HIST1H1B
CDK2	GOT2
CDK2	FZR1
FUS	CDK2
CDK2	FOXM1
CDK2	FLG2
CDK2	FEN1
CDK2	DCD
CUL1	CDK2
COPS5	CDK2
CKS2	CDK2
CDK2	CHAF1B
CDK2	CDKN3
CDKN1B	CDK2
CDK7	CDK2
CDK2	CDK6
CDK2	CDC7
CDK2	CDC6
CDC25B	CDK2
CDC25A	CDK2
CDK2	CCT8
CCT7	CDK2
CDK2	CCT6A

CCT5	CDK2
CDK2	CCT4
CDK2	CCT3
CDK2	CCT2
CCNK	CDK2
CDK2	CCNH
CDK2	CCNE2
CDK2	CCNE1
CDK2	CCND3
CDK2	CCNB1
CDK2	CCNA2
CDK2	BRCA1
ATP5A1	CDK2
CDK2	ATP1A1
CDK2	ATIC
MED9	CDK19
MED28	CDK19
MED26	CDK19
MED1	CDK19
EIF3F	CDK19
CCDC85B	CDK18
YWHAE	CDK14
RB1	CDK14
CCNY	CDK14
UBC	CDK13
UBC	CDK12
PIN1	CDK12
CDK11B	URM1
UBC	CDK11B
CDK11B	PAK1
KAT7	CDK11B
HSP90AA1	CDK11B
CDK11B	CCNL2
UBC	CDK11A
PIN1	CDK11A
CCNL1	CDK11A
UBC	CDK10
WEE1	CDK1
VIM	CDK1
CDK1	USP16
UBC	CDK1
TSC1	CDK1
TP53	CDK1
TOP2A	CDK1

TK1	CDK1
SUMO2	CDK1
STMN2	CDK1
STMN1	CDK1
SFN	CDK1
RPS6KB1	CDK1
CDK1	RPA2
RPA1	CDK1
CDK1	RGCC
RB1	CDK1
PTPN1	CDK1
PKMYT1	CDK1
PITPNM1	CDK1
PCNA	CDK1
NPM1	CDK1
CDK1	NES
CDK1	MAP4
LYN	CDK1
CDK1	LMNB1
LMNA	CDK1
CDK1	KRT18
CDK1	KIF11
CDK1	ITPR1
HIST1H1B	CDK1
HIST1H1A	CDK1
CDK1	H1F0
GFAP	CDK1
GADD45B	CDK1
GADD45A	CDK1
FANCC	CDK1
FANCA	CDK1
EZH2	CDK1
CDK1	ERCC2
EGFR	CDK1
DAB2	CDK1
CSNK2A1	CDK1
CKS2	CDK1
CDK1	CHAF1B
CDK1	CDKN3
CDK7	CDK1
CDC6	CDK1
CDC25C	CDK1
CDC25B	CDK1
CCNE1	CDK1

CDK1	CCNB2
CCNB1	CDK1
CCNA2	CDK1
CDK1	CCNA1
BRCA1	CDK1
BIRC5	CDK1
BCL2	CDK1
UBC	CDIPT
TNFRSF1B	CDIPT
TNFRSF1A	CDIPT
UBC	CDIP1
CDH19	CDH6
CDH18	CDH6
CDH10	CDH6
VIM	CDH5
DSP	CDH5
CTNND1	CDH5
CDH2	CDH4
USH1C	CDH23
UBC	CDH2
GRIK2	CDH2
GNA12	CDH2
CTNND1	CDH2
GNA12	CDH15
COPS6	CDH10
VCL	CDH1
UBC	CDH1
SKP2	CDH1
CDH1	PTPRM
PSEN1	CDH1
JUP	CDH1
EGFR	CDH1
CTNND1	CDH1
CDH1	CTNNA1
CDH1	CBLL1
ANAPC7	CDH1
PRKCD	CDCP1
UBC	CDCA8
UBC	CDCA7L
UBC	CDCA3
TRAF2	CDCA3
SKP1	CDCA3
CUL1	CDCA3
UBC	CDC73

UBC	CDC7
ORC5	CDC7
MCM7	CDC7
MCM4	CDC7
MCM2	CDC7
CDC7	DBF4B
UBC	CDC6
ORC5	CDC6
CDC6	ORC3
MYC	CDC6
CDC6	MCM7
CDC6	MCM2
CCNA2	CDC6
YWHAG	CDC5L
UBC	CDC5L
U2AF2	CDC5L
CDC5L	TTF2
SUMO2	CDC5L
CDC5L	SRRM2
CDC5L	SRRM1
SNRPA1	CDC5L
SNRNP200	CDC5L
SFPQ	CDC5L
CDC5L	RPL12
CDC5L	PRPF3
CDC5L	PPP1R8
PPP1CA	CDC5L
PLRG1	CDC5L
ORC5	CDC5L
ORC2	CDC5L
MCM5	CDC5L
MCM4	CDC5L
MCM3	CDC5L
CDC5L	CWC15
CDC5L	CTNNBL1
CRNKL1	CDC5L
CDC5L	BCAS2
UBC	CDC45
ORC5	CDC45
MCM7	CDC45
MCM2	CDC45
UBC	CDC42SE2
UBC	CDC42SE1
CDC42	CDC42SE1

CDC42	CDC42EP5
UBC	CDC42EP4
RHOQ	CDC42EP4
RHOQ	CDC42EP3
RHOQ	CDC42EP2
CDC42	CDC42EP2
UBC	CDC42EP1
MAPKAP1	CDC42EP1
FHL3	CDC42EP1
CDC42	CDC42EP1
UBC	CDC42BPA
CDC42	CDC42BPA
VAV1	CDC42
CDC42	UBR1
UBC	CDC42
TP53	CDC42
TNK2	CDC42
CDC42	PDE6D
CDC42	PARD6B
PARD3	CDC42
CDC42	PAK2
CDC42	MAP3K4
ITSN1	CDC42
CDC42	IQGAP2
EEF1G	CDC42
CDC42	CSPG4
CDC42	CDC42EP3
C14orf1	CDC42
BAIAP2	CDC42
ARHGEF7	CDC42
CDC42	ARHGAP1
SMAD2	CDC40
DHX38	CDC40
UBC	CDC37
TBK1	CDC37
STIP1	CDC37
MAP3K14	CDC37
EGFR	CDC37
CHUK	CDC37
UBC	CDC34
SKP1	CDC34
CREM	CDC34
ATF5	CDC34
CDC27	UBE2C

UBC	CDC27
TTK	CDC27
RB1	CDC27
PTTG1	CDC27
PIN1	CDC27
MDC1	CDC27
MAD2L1	CDC27
HOXC10	CDC27
FZR1	CDC27
CUEDC2	CDC27
CREBBP	CDC27
CDH1	CDC27
CDC27	CDC26
CDC20	CDC27
CDC16	CDC27
CDC27	CCNB1
CCNA2	CDC27
BUB3	CDC27
BUB1B	CDC27
CDC27	APC2
ANAPC7	CDC27
ANAPC5	CDC27
ANAPC4	CDC27
CDC27	ANAPC16
CDC27	ANAPC13
CDC27	ANAPC10
ANAPC1	CDC27
CDC16	CDC26
YWHAQ	CDC25C
YWHAH	CDC25C
YWHAE	CDC25C
YWHAB	CDC25C
UBC	CDC25C
TP53	CDC25C
PPP2R5D	CDC25C
PLK1	CDC25C
PIN1	CDC25C
NEDD4	CDC25C
MAPK14	CDC25C
CHEK2	CDC25C
CDK2	CDC25C
YWHAE	CDC25B
UBC	CDC25B
CDC25B	SFN

RAF1	CDC25B
CDC25B	HRAS
UBC	CDC20
PTTG1	CDC20
CDC20	PLK1
MDC1	CDC20
CDC20	MAD2L1
FZR1	CDC20
CDC20	CDC16
CCNA2	CDC20
CDC20	BUB1B
CDC20	APC
ANAPC5	CDC20
CDC20	ANAPC4
CDC20	ANAPC1
TP53	CDC14A
ZNF512B	CDC123
UBC	CDC123
ASF1B	CDAN1
CD93	MBL2
CD93	COL4A3
COL4A2	CD93
COL4A1	CD93
COL1A1	CD93
CALR	CD93
C1QA	CD93
UBC	CD9
CD9TSPAN4
SERPINH1	CD9
CD9PTGFRN
PRKCA	CD9
KIT CD9
ITGB3	CD9
ITGB1	CD9
CD9ITGA7
ITGA3	CD9
CD9ITGA2
CD81	CD9
CD9CD63
CD59	CD9
CD9CD53
CD36	CD9
CD19	CD9
CD9ADAM2

UBC	CD8A
PTPRC	CD8A
LAT	CD8A
HLA-G	CD8A
CD8A	CD8B
CD8A	CD1A
UBC	CD86
CTLA4	CD86
CD28	CD86
UBC	CD82
TAB2	CD82
CD82	NUP88
ITGB1	CD82
CD82	HLA-DMA
HBEGF	CD82
CD19	CD82
CANX	CD82
UBC	CD81
CD81	PTGFRN
CD81	ITGB1
CD81	ITGA4
CTLA4	CD80
CD28	CD80
UBC	CD79B
SYK	CD79B
CD79A	CD79B
LCK	CD79A
CD79A	BLNK
UBC	CD74
SEMA4D	CD72
PTPN6	CD72
CD27	CD70
UBC	CD63
CD63	PTGFRN
CD63	PI4K2A
ITGB1	CD63
CD81	CD63
ALCAM	CD6
CD59	CD2
HERPUD1	CD58
DNAJA1	CD58
CD2	CD58
LCK	CD55
GPLD1	CD55

CR1	CD55
CD97	CD55
CD14	CD55
CD53	MS4A1
ITGB1	CD53
IL4	CD53
CD53	GGT1
CD2	CD53
RASA1	CD5
PIK3R1	CD5
LCK	CD5
CSNK2A1	CD5
CD72	CD5
CD4	CD5
CD247	CD5
CD2	CD5
CBL	CD5
LCK	CD48
CD2	CD48
UBC	CD47
CD47	RHAG
UBC	CD46
CD46	TSPAN4
CD44	VCAN
UBC	CD44
CD44	MMP9
LCK	CD44
FYN	CD44
ERBB4	CD44
EGFR	CD44
CD44	CD74
CD44	ANK1
TRAF1	CD40
CD40	MS4A1
JAK3	CD40
BIRC2	CD40
UBC	CD4
CD4	TNFRSF10A
SELL	CD4
CD4	PTPRC
CD4	PIP
PIK3R1	CD4
CD4	LGALS1
LCK	CD4

LAT	CD4
CD4	IL2RA
CD4	HLA-DPA1
CD4	FCGR3A
CD4	CXCR4
CD4	CXCL12
CD4	CD53
CD44	CD4
CD4	CD38
CD4	CD28
CD4	CD2
CD4	CCR5
CD3D	CD3G
POLR1A	CD3EAP
CD3EAP	NFKBIB
ZAP70	CD3E
TOP2B	CD3E
SYK	CD3E
PIK3R1	CD3E
NCK2	CD3E
NCK1	CD3E
LCK	CD3E
EPS8L1	CD3E
CD79B	CD3E
CD3G	CD3E
CD3D	CD3E
CD247	CD3E
VCP	CD3D
UBC	CD3D
CD8B	CD3D
CD247	CD3D
AMFR	CD3D
LCK	CD38
UBC	CD33
UBC	CD320
USP39	CD2BP2
SNRNP40	CD2BP2
NEDD4	CD2BP2
UBC	CD2AP
RAB4A	CD2AP
CD2AP	PKD2
PIK3R1	CD2AP
CD2AP	PDCD6IP
CD2AP	NPHS2

CD2AP	MVB12A
CD2AP	GZMB
CD2AP	GRB2
CD2AP	FYN
CD2AP	CD2
CBLB	CD2AP
CD2AP	CAPZB
CD2AP	BCAR1
LCK	CD28
ITK	CD28
GRAP2	CD28
CD247	CD28
UBC	CD276
UBC	CD247
CD247	SH2B3
CD247	NCR1
LCK	CD247
CD247	JAK3
CD247	FYN
CD247	DOCK2
CD247	CD2
CD244	CD48
PVRL2	CD226
ITGB2	CD226
CD200	CD200R1
UBC	CD2
LCK	CD2
FYN	CD2
CD2BP2	CD2
PTPRC	CD1D
CD74	CD1D
CALR	CD1D
B2M	CD1D
VAV1	CD19
PLCG2	CD19
PIK3R1	CD19
FYN	CD19
CD81	CD19
CD19	CD79A
LY86	CD180
KCNIP3	CD177
CD151	ITGA7
LGALS3BP	CD14
CD14	LBP

ITGB2	CD14
CD14	ITGAM
UBC	CCZ1B
TCP1	CCT8
TBK1	CCT8
PSMC2	CCT8
CCT8	PPP2R2C
CCT7	CCT8
EEF1G	CCT7
UBC	CCT6B
UBC	CCT6A
TCP1	CCT6A
TBK1	CCT6A
PPP2R2C	CCT6A
CCT8	CCT6A
CCT7	CCT6A
CCT4	CCT6A
VHL	CCT5
CCT5	VCP
UBC	CCT5
TP53	CCT5
CCT5	TCP1
CCT5	TBK1
CCT5	TBC1D17
CCT5	PPP4C
CCT5	PPP2R2C
CCT5	IKBKE
CCT5	IKBKAP
CCT5	CCT8
CCT5	CCT7
CCT5	ACTB
CCT5	ACTA2
CCT5	ACTA1
TCP1	CCT4
TBK1	CCT4
CCT4	PPP2R2C
CCT7	CCT4
VHL	CCT3
UBC	CCT3
TCP1	CCT3
TBK1	CCT3
RAF1	CCT3
PPP4C	CCT3
PPP2R2C	CCT3

MOB4	CCT3
HNRNPA0	CCT3
CCT7	CCT3
CCT3	CCT6A
CCT4	CCT3
VCP	CCT2
UBC	CCT2
TCP1	CCT2
TBK1	CCT2
RIPK3	CCT2
PRPF40A	CCT2
PPP4C	CCT2
PPP2R2C	CCT2
PPP2R2B	CCT2
CCT8	CCT2
CCT7	CCT2
CCT6A	CCT2
CCT5	CCT2
CCT4	CCT2
CCT3	CCT2
ACTB	CCT2
TCEA2	CCSER2
STX1A	CCSER2
PSMD11	CCSER2
GTF2H1	CCSER2
CRADD	CCSER2
UBC	CCS
APBA1	CCS
CCL25	CCR9
CCR5	TXK
LCK	CCR5
CXCR4	CCR5
CCR5	CCL8
CCL5	CCR5
CCR5	CCL2
CCL11	CCR5
CXCL12	CCR4
CCL5	CCR4
CCL3	CCR4
CCL22	CCR4
CCL17	CCR4
ADRBK2	CCR4
ADRBK1	CCR4
HCK	CCR3

CCL5	CCR3
CCR3	CCL26
CCR3	CCL15
CCL11	CCR3
GNA14	CCR1
CCL5	CCR1
CCL2	CCR1
CCL15	CCR1
CCL1	CCR1
UBC	CCPG1
UBC	CCP110
CEP97	CCP110
CCP110	CEP76
CALM1	CCP110
UBC	CCNY
UBC	CCNT1
SKP2	CCNT1
MYC	CCNT1
CCNT1	HEXIM1
CCNT1	GRN
CDK9	CCNT1
CCNT1	BRD4
CCNT1	AFF4
AFF1	CCNT1
VPRBP	CCNO
UBC	CCNK
TSC22D4	CCNK
PIN1	CCNK
CCNK	FKBP6
CCNK	CEP76
CDK18	CCNK
CCDC85B	CCNK
UBC	CCNI
CCNH	ERCC3
MDM2	CCNG1
UBC	CCNE2
CDKN1B	CCNE2
UBC	CCNE1
CCNE1	HIST1H1B
CCNE1	COIL
CDC25ACCNE1
CCNDBP1	ZNF334
UBC	CCNDBP1
RB1CCND3

EIF3K	CCND3
EFEMP2	CCND3
DTNBP1	CCND3
CDKN1B	CCND3
CDK6	CCND3
CDK4	CCND3
CDK11B	CCND3
ATP5C1	CCND3
CCND3	AREG
UBC	CCND2
RB1	CCND2
HIST1H1A	CCND2
CDKN1B	CCND2
CDK6	CCND2
CDK5	CCND2
CDK4	CCND2
CDK2	CCND2
UBC	CCNC
CDK8	CCNC
UBC	CCNB2
TGFBR2	CCNB2
MCM2	CCNB2
CKS2	CCNB2
CDK2	CCNB2
UBC	CCNB1IP1
CCNB1	ZC3HC1
UBC	CCNB1
TSC1	CCNB1
TGFBR2	CCNB1
RPA1	CCNB1
PTCH1	CCNB1
PLK1	CCNB1
PKMYT1	CCNB1
PIN1	CCNB1
HIST1H1B	CCNB1
FZR1	CCNB1
CKS2	CCNB1
CDC25A	CCNB1
CDC20	CCNB1
CDC16	CCNB1
BRCA1	CCNB1
ANAPC7	CCNB1
ANAPC5	CCNB1
ANAPC4	CCNB1

ANAPC1	CCNB1
CCNA2	HIST1H1B
CCNA2	FZR1
XRCC6	CCNA1
SKP2	CCNA1
RPA1	CCNA1
CCNA1	RBM4
RB1	CCNA1
MYBL2	CCNA1
MCM6	CCNA1
MCM4	CCNA1
HIST1H1B	CCNA1
HIST1H1A	CCNA1
GPS2	CCNA1
GNB2L1	CCNA1
FZR1	CCNA1
CUL1	CCNA1
CDKN1B	CCNA1
CDK2	CCNA1
CDC25A	CCNA1
CDC20	CCNA1
BRCA1	CCNA1
VCAN	CCL8
CCR3	CCL8
CCL8	CCR1
MMP2	CCL7
CCL7	MMP14
MMP1	CCL7
FEZ1	CCL7
CCR5	CCL7
CCL7	CCR3
CCL7	CCR1
AES	CCL7
SRGN	CCL3
CEBPB	CCL3
CCR5	CCL3
CCR3	CCL3
CCL3	CCR1
CCR3	CCL28
CCR3	CCL24
CCR1	CCL23
VCAN	CCL21
CCL21	CCR7
VCAN	CCL20

CCL20 CCR6
VCAN CCL2
MMP1 CCL2
CCL2 CCR3
NFKB1 CCL19
CCL19 CCR7
TP53 CCL18
C14orf1 CCL18
CCL17 CCR8
CXCR5 CCL16
CCR8 CCL16
CCR5 CCL16
CCR1 CCL16
CCR5 CCL14
CCR1 CCL14
HNRNPH3 CCL13
CCR5 CCL13
CCR3 CCL13
CCL11 DPP4
CCL1 CCR8
CCK CCKBR
CPE CCK
UBC CCHCR1
UBC CCDC93
CHGB CCDC92
TESC CCDC91
GGA1 CCDC91
SERPINB9 CCDC90B
RNF10 CCDC90B
PSMD11 CCDC90B
PRDX5 CCDC90B
FXYD6-FXYD2 CCDC90B
BTBD2 CCDC90B
CCDC90B AGR2
PBX2 CCDC88B
CCDC85B ZNF250
CCDC85B VPS72
CCDC85B TEAD4
CCDC85B SLU7
CCDC85B SIX1
CCDC85B RBM7
CCDC85B RALYL
CCDC85B NUP54
CCDC85B MCRS1

CCDC85B	LMO3
CCDC85B	KRT17
CCDC85B	DTNB
CCDC85B	CCDC116
CCDC85B	CCDC112
CCDC85B	CCDC11
CCDC85B	C8orf48
CCDC85B	C21orf91
CCDC85B	ALS2CR11
UBC	CCDC77
UBC	CCDC71L
IFIT3	CCDC67
CCDC85B	CCDC67
UBC	CCDC66
UBC	CCDC6
PPP4C	CCDC6
OPTN	CCDC53
MCRS1	CCDC53
KRT6A	CCDC53
ABI2	CCDC53
UBC	CCDC50
RPS3A	CCDC50
UBC	CCDC43
TSC22D4	CCDC42
USHBP1	CCDC33
TSC22D4	CCDC33
TRAF2	CCDC33
CCDC33	SSSCA1
RAD54B	CCDC33
PLSCR1	CCDC33
NINL	CCDC33
FXR2	CCDC33
EHMT2	CCDC33
DVL2	CCDC33
CCDC85B	CCDC33
CALCOCO2	CCDC33
PPP1CA	CCDC181
TCEB3B	CCDC169-SOHLH2
UBC	CCDC168
NAGK	CCDC158
UBC	CCDC149
ZNF408	CCDC136
UBC	CCDC134
TRAF2	CCDC130

NINL	CCDC130
UBC	CCDC127
RNF11	CCDC127
CRNKL1	CCDC12
PFN2	CCDC113
HAP1	CCDC113
TP53	CCDC106
RBM48	CCDC106
EEF1G	CCDC106
COPS6	CCDC106
C14orf1	CCDC106
UBC	CCDC104
SUPT3H	CCDC101
KRT15	CCDC101
CCDC101	CCDC158
UBC	CCBL2
UBC	CCAR1
SUMO2	CCAR1
HAP1	CBX8
EEF1G	CBX8
PCGF6	CBX7
HDAC2	CBX7
UBC	CBX6
RNF2	CBX6
RING1	CBX6
CBX6	MOV10
HIST3H3	CBX6
XRCC6	CBX5
UBC	CBX5
TRIM28	CBX5
TRIM24	CBX5
TCEB3B	CBX5
CBX5	SUV420H2
SUMO2	CBX5
SMARCA2	CBX5
CBX5	SGOL1
RPSA	CBX5
RBBP4	CBX5
RB1	CBX5
CBX5	PRR14
NSL1	CBX5
CBX5	NIPBL
MKI67	CBX5
MIS12	CBX5

MBD1	CBX5
LRIF1	CBX5
LBR	CBX5
HIST4H4	CBX5
HIST3H3	CBX5
HIST1H3A	CBX5
CBX5	HDAC9
HDAC4	CBX5
H3F3A	CBX5
FSHR	CBX5
EHMT2	CBX5
DSN1	CBX5
CBX5	DNMT3B
DNMT3A	CBX5
DNMT1	CBX5
CBX5	CHAF1B
CHAF1A	CBX5
CBX5	CBX1
CBX5	BCL11B
ADNP	CBX5
YWHAB	CBX4
UBC	CBX4
SMAD1	CBX4
SKIL	CBX4
RNF2	CBX4
RING1	CBX4
PHC1	CBX4
CTBP1	CBX4
CBX8	CBX4
CBX4	CBX2
YAF2	CBX3
UBC	CBX3
TRIM28	CBX3
TRIM24	CBX3
SP100	CBX3
POLR2A	CBX3
PIM1	CBX3
PCGF6	CBX3
LBR	CBX3
L3MBTL2	CBX3
HIST4H4	CBX3
HIST1H3A	CBX3
HIST1H1E	CBX3
H3F3A	CBX3

EHMT2	CBX3
DNMT3B	CBX3
CBX5	CBX3
CBX1	CBX3
RING1	CBX2
UCHL1	CBX1
UBC	CBX1
TRIM28	CBX1
CBX1	SUV420H2
RPL12	CBX1
HIST3H3	CBX1
HIST1H3A	CBX1
EHMT2	CBX1
DNMT3A	CBX1
DNMT1	CBX1
CHAF1A	CBX1
C11orf30	CBX1
CBS	HTT
UBC	CBR3
UBC	CBR1
PRKAB1	CBR1
ERCC8	CBR1
CBLN1	CBLN3
UBC	CBLC
EGFR	CBLC
CRK	CBLC
VAV1	CBLB
CBL	ZAP70
YWHAQ	CBL
YWHAB	CBL
CBL	VAV2
VAV1	CBL
UBE2L3	CBL
CBL	UBE2G2
UBE2D3	CBL
UBE2D2	CBL
UBC	CBL
CBL	UBASH3B
CBL	TYK2
TUBB	CBL
CBL	SYK
CBL	STAT5A
CBL	STAP2
CBL	SPRY2

CBL	SPRY1
SORBS2	CBL
CBL	SORBS1
CBL	SLA
CBL	SH3GL3
CBL	SH3GL2
CBL	SH3BP2
CBL	RET
PXN	CBL
CBL	PTPN22
CBL	PTK2B
PRKCA	CBL
PLCG1	CBL
CBL	PIK3R2
PIK3R1	CBL
PIK3CB	CBL
CBL	PDGFRB
NCK1	CBL
CBL	MYH9
MAPK14	CBL
CBL	LYN
LCP2	CBL
LCK	CBL
CBL	KIT
KDR	CBL
ITSN1	CBL
ITK CBL	
ITCH	CBL
CBL	INPPL1
HSPA5	CBL
HCK	CBL
GRB2	CBL
FYN	CBL
FGR	CBL
CBL	EPOR
CBL	EPHB6
CBL	EPHB1
EGFR	CBL
CSF1R	CBL
CRKL	CBL
CRK	CBL
CFTR	CBL
CDC42	CBL
CBL	CD38

CD2AP	CBL
CD19	CBL
BCR	CBL
ARHGEF7	CBL
ACTB	CBL
ABL1	CBL
TAL1	CBFA2T3
RUNX1T1	CBFA2T3
HDAC3	CBFA2T3
HDAC1	CBFA2T3
NCOR2	CBFA2T2
CBFA2T2	CBFB
NOS1	CAV3
EGFR	CAV3
CAV3	DYSF
DAG1	CAV3
VCP	CAV1
UBC	CAV1
TRPC1	CAV1
TRAF2	CAV1
CAV1	SDPR
CAV1	S1PR1
CAV1	RCVRN
PTPRF	CAV1
PTPN6	CAV1
PTEN	CAV1
CAV1	PRNP
PPP1CA	CAV1
CAV1	NOS3
CAV1	NEU3
CAV1	HRAS
CAV1	GJB2
CAV1	GJA1
CAV1	FLOT2
EGFR	CAV1
CAV1	EDNRB
CAV1	CAV2
CAV1	BST1
UBC	CAT
PEX5	CAT
ATM	CAT
CAPN1	CAST
PRKCB	CASR
VIM	CASP9

UBC	CASP9
CASP9	GZMB
CASP9	BIRC8
CASP9	BIRC7
CASP9	BIRC6
CASP9	BIRC2
CASP9	BCL10
CASP9	APAF1
UBC	CASP8AP2
TRAF2	CASP8AP2
SUMO1	CASP8AP2
FADD	CASP8AP2
VIM	CASP8
UBC	CASP8
TRAF6	CASP8
TRAF2	CASP8
TRAF1	CASP8
TNFSF10	CASP8
TNFRSF10B	CASP8
TNFRSF10A	CASP8
TFCP2	CASP8
RIPK2	CASP8
RIPK1	CASP8
CASP8	PICALM
PEA15	CASP8
MAP4K4	CASP8
MAP3K14	CASP8
CASP8	IRF1
IFT57	CASP8
GZMB	CASP8
FASLG	CASP8
FADD	CASP8
CASP8	DEDD2
CASP8	DEDD
CUL3	CASP8
CTNNB1	CASP8
CRADD	CASP8
CHUK	CASP8
CHD3	CASP8
CFLAR	CASP8
CASP9	CASP8
CASP8AP2	CASP8
CASP7	CASP8
CASP6	CASP8

CASP3	CASP8
CASP8	CASP2
CASP8	CASP14
CASP8	CASP10
BIRC2	CASP8
BID	CASP8
BCL2L1	CASP8
BCL2	CASP8
ATN1	CASP8
APP	CASP8
APAF1	CASP8
VIM	CASP7
UBC	CASP7
TNFRSF1A	CASP7
CASP7	RAD51
PARP1	CASP7
HSPA5	CASP7
CASP7	GZMB
CASP7	BIRC6
CASP7	BIRC3
CASP7	BIRC2
CASP7	ATN1
VIM	CASP6
UBC	CASP6
LMNA	CASP6
CASP6	EIF2S1
CASP6	CHD3
CASP6	CASP3
NLRP1	CASP5
SERPINB9	CASP4
IL18	CASP4
APAF1	CASP4
VIM	CASP3
UBC	CASP3
CASP3	TRAF1
CASP3	SRP72
CASP3	ROCK1
CASP3	RFC1
CASP3	RAD51
CASP3	PARG
CASP3	PAK2
MDM2	CASP3
MAPT	CASP3
CASP3	KCNIP3

CASP3 IL18
CASP3 HTT
HSPE1 CASP3
CASP3 HCLS1
CASP3 GZMB
CASP3 GORASP1
CASP3 EIF2S1
DFFA CASP3
CASP3 CFLAR
CASP3 CDK11A
CASP9 CASP3
CASP3 CASP4
CASP3 BIRC7
CASP3 BIRC6
CASP3 BIRC2
BID CASP3
CASP3 ARHGDIA
CASP3 APP
ACIN1 CASP3
UBC CASP2
CASP2 MZB1
CRADD CASP2
CASP3 CASP2
CASP2 CASP14
CASP1 CASP2
BID CASP2
TNFRSF1A CASP10
TNFRSF10B CASP10
RYBP CASP10
GZMB CASP10
FADD CASP10
CFLAR CASP10
CASP10 CASP14
PYCARD CASP1
NOD2 CASP1
IL18 CASP1
CASP1 CASP14
CASP1 CARD16
UBC CASC5
CASC5 SUMO2
CASC5 PPP1CA
NSL1 CASC5
MIS12 CASC5
CASC5 BUB1B

CASC5	BUB1
UBC	CASC3
CASC3	STAU1
MAGOH	CASC3
UBC	CARS
TRIP12	CARNS1
UBC	CARM1
TP53	CARM1
NCOA3	CARM1
NCOA2	CARM1
GRIP1	CARM1
UBC	CARHSP1
STC2	CARHSP1
CCDC85B	CARD9
BCL10	CARD9
CASP9	CARD8
CASP1	CARD8
CARD16	CARD8
RIPK2	CARD6
NOD1	CARD6
CARD8	CARD18
BCL10	CARD14
UBC	CARD11
PRKCQ	CARD11
MAP3K7	CARD11
CHUK	CARD11
BCL10	CARD11
UBC	CARD10
BCL10	CARD10
UBC	CAPZB
UBC	CAPZA2
PXN	CAPZA2
CAPZB	CAPZA2
UBC	CAPZA1
RIPK3	CAPZA1
MAP3K3	CAPZA1
CD2AP	CAPZA1
CAPZB	CAPZA1
UBC	CAPRIN1
PIN1	CAPRIN1
CAPN2	CAPNS2
UBC	CAPNS1
GNB2	CAPNS1
DRG1	CAPNS1

CDK4	CAPNS1
CAPN2	CAPNS1
CAPNS1	CAPN1
UBC	CAPN7
UBC	CAPN5
UBC	CAPN1
NCAPH	CAPG
UBC	CANX
TNFRSF1A	CANX
CANX	TG
SSR1	CANX
SMURF2	CANX
CANX	SLC4A1
CANX	PDIA3
CANX	MBTPS1
CANX	LPA
CANX	LCT
KCNH2	CANX
ITGB1	CANX
GRIA1	CANX
FAM107A	CANX
CANX	EDEM1
CFTR	CANX
CANX	CD9
CANX	CD3D
CANX	CD1D
APOB	CANX
CANX	ABCC2
UBC	CAND1
SKP1	CAND1
CUL2	CAND1
CUL1	CAND1
COPS5	CAND1
SMAD9	CAMSAP1
SMAD1	CAMSAP1
SKIL	CAMSAP1
TCEA2	CAMKV
UBC	CAMKK2
CAMK1	CAMKK2
CALM1	CAMKK2
CAMK4	CAMKK1
CAMK1	CAMKK1
NOS1	CAMK4
CAMK4	CAMKK2

UBC	CAMK2G
TIAM1	CAMK2G
CAMK2G	TH
STAT1	CAMK2G
SMAD4	CAMK2G
SMAD3	CAMK2G
CAMK2G	RRAD
CAMK2G	PEA15
GRIA1	CAMK2G
EGFR	CAMK2G
CALM1	CAMK2G
CAMK2G	CABP2
CAMK2G	ADCY3
ACTN4	CAMK2G
VIM	CAMK2D
UBC	CAMK2D
SMAD4	CAMK2D
HDAC4	CAMK2D
UBC	CAMK2B
UBC	CAMK2A
CAMK2A	SYNGAP1
SRF	CAMK2A
PPM1F	CAMK2A
PDC	CAMK2A
NOS1	CAMK2A
MAPT	CAMK2A
CAMK2A	KRT18
ITPKA	CAMK2A
GRIN2B	CAMK2A
GRIN2A	CAMK2A
GFAP	CAMK2A
EGFR	CAMK2A
DAPK2	CAMK2A
CAMK2A	CAMK2D
CALM1	CAMK2A
ACTN1	CAMK2A
NOS1	CAMK1
UBC	CALU
CALR	PRF1
CALR	PDIA2
CALR	NKX2-1
CALR	FGB
APOB	CALR
VCL	CALML5

UBC	CALML5
MYO10	CALML3
KIAA1683	CALML3
YWHAE	CALM1
UBC	CALM1
TRAF6	CALM1
TRAF2	CALM1
CALM1	TNFRSF1B
CALM1	TNFRSF1A
CALM1	TBK1
CALM1	TANK
CALM1	TAB2
CALM1	TAB1
CALM1	SYK
CALM1	SNCA
CALM1	REL
CALM1	RAB3B
CALM1	PPP3R1
CALM1	PDE1A
CALM1	OPRM1
CALM1	NOS3
CALM1	NOS2
CALM1	NFKB2
CALM1	NFKB1
CALM1	MYF6
CALM1	MIP
CALM1	MAP3K14
CALM1	KIAA1683
CALM1	KCNQ5
CALM1	KCNQ3
CALM1	KCNN2
CALM1	KCNH1
CALM1	IQGAP2
CALM1	IQGAP1
CALM1	IQCB1
CALM1	IKBKE
CALM1	HSP90AA1
CALM1	GRM7
CALM1	GRM5
CALM1	GRK1
CALM1	GP6
FKBP8	CALM1
CALM1	FER
ESR1	CALM1

EGFR	CALM1
CALM1	DRD2
CALM1	DDX5
CALM1	DAPK1
CALM1	CSNK2A2
CALM1	CNN1
CALM1	CHUK
CALM1	CCNE1
CALM1	CAMK1
CALM1	ASCL2
CALM1	ADCY8
UBC	CALD1
POT1	CALD1
GRB2	CALD1
CALM1	CALD1
CALCOCO2	ZNF638
CALCOCO2	ZNF101
CALCOCO2	VPS72
UBC	CALCOCO2
TRAF2	CALCOCO2
CALCOCO2	TCL1A
CALCOCO2	TCEB3B
CALCOCO2	TBKBP1
CALCOCO2	TBK1
CALCOCO2	RABL6
CALCOCO2	PTBP2
CALCOCO2	PTBP1
CALCOCO2	LSM4
CALCOCO2	KAT7
CALCOCO2	KANSL1
CALCOCO2	GABARAPL2
CALCOCO2	DBNDD2
DAZAP2	CALCOCO2
CALCOCO2	AP5B1
CALCOCO2	ABLIM1
KRT15	CALCOCO1
CALCOCO1	FOSL1
CALCOCO1	CYTH3
CALCOCO1	CRYBA2
CALCRL	CALCA
CALB2	KRT1
CALB1	IMPA1
SDCBP	CADM1
EPB41L3	CADM1

UBC CACYBP
SKP1 CACYBP
UBC CACUL1
GRIA1 CACNG2
UBC CACNB1
SRI CACNA1S
RYR1 CACNA1S
CACNA1C PRKACA
CALM1 CACNA1C
CACNA1C CABP1
PRKCE CACNA1B
UBC CACNA1A
SYT1 CACNA1A
GNB1 CACNA1A
CACNB4 CACNA1A
CABP1 CACNA1A
CACHD1 ZMIZ1
CACHD1 RANBP10
CACHD1 GLTSCR1L
TP53 CABLES1
CDK5 CABLES1
CDK3 CABLES1
UBC CAB39
STRADB CAB39
CA2SLC4A3
DYNLL1 CA2
ANKZF1 CA2
CA12 MRPL9
CA12 ATP5C1
UBC C9orf64
UBC C9orf41
UBC C9orf40
UBC C9orf114
CLU C9
CD59 C9
UBC C8orf59
HAP1 C8orf33
C8B C5
CD59 C8A
C9 C8A
C8G C8A
C8B C8A
UBC C7orf50
CCDC85B C7orf50

ZBTB48 C7orf34
FZR1 C7orf25
BRD4 C7orf25
PLGC7
CLU C7
C5 C7
PTPN6 C6orf25
STRAP C6orf203
C6orf165 RNF138
F2 C6
C5 C6
TRAF2 C5orf55
UBC C5orf51
UBC C5orf24
WBP11 C5orf22
UBC C5orf22
ELOF1 C5orf22
UBC C5orf15
C5AR1 C5AR2
C5 C5AR1
UBC C4orf27
PROS1 C4BPA
PAPPA C3
C3 OLFM4
C3 ITGAX
C3 ITGAM
C3 CR2
C3 CR1
C3 CPN1
C3 CFI
C3 CFHR5
C3 CFHR4
C3 CFH
C3 CFB
CD46 C3
C3 C5
C3 C3AR1
C3 C2
ABL1 C3
PRKAR1A C2orf88
UBC C2orf47
RUVBL2 C2orf44
RUVBL1 C2orf44
MED31 C2orf44

CSNK1E	C2orf44
UBC	C2orf43
UBC	C21orf59
RALYL	C20orf27
PHKB	C20orf27
UBC	C20orf24
C5	C2
C1S	C2
C1S	C1QA
C1S	C1R
C1QA	C1R
SGTA	C1QTNF1
PTX3	C1QC
YWHAE	C1QBP
UBC	C1QBP
C1QBP	TRAF1
C1QBP	TNFRSF1B
C1QBP	TNFRSF1A
C1QBP	TAB2
PRKD1	C1QBP
PRKCD	C1QBP
PRKCA	C1QBP
C1QBP	NFKB1
C1QBP	MAP3K14
C1QBP	HRK
C1QBP	HNRNPD
C1QBP	HABP4
C1QBP	GAB1
C1QB	PTX3
C1QB	C1QC
C1QA	C1QB
C1QA	PTX3
C1QA	CR1
C1QA	C1QC
USO1	C1orf94
DAZAP2	C1orf94
UBC	C1orf52
UBC	C1orf35
THAP1	C1orf35
MTUS2	C1orf216
UBC	C1orf198
KAT5	C1orf174
EEF1G	C1orf174
COPS6	C1orf174

UBC	C1orf123
HOMER1	C1orf116
UBC	C1orf112
CCDC85B	C1orf111
UBC	C1orf109
UBC	C19orf70
MDFI	C19orf60
VCL	C19orf57
TRIM23	C19orf57
RUNX1T1	C19orf57
RABL6	C19orf57
GPS2	C19orf57
COPS4	C19orf57
AIMP2	C19orf57
UBC	C19orf53
UBC	C19orf52
FANCM	C19orf40
CCDC85B	C19orf25
C19orf57	C19orf25
UBC	C19orf10
BAG6	C19orf10
UBC	C18orf32
UBE2I	C18orf25
SUMO2	C18orf25
FANCM	C17orf70
UBC	C16orf80
UBC	C16orf58
UBC	C15orf38-AP3S2
UBC	C14orf80
VCL	C14orf166
UBC	C14orf166
NIN	C14orf166
C14orf1	SNRPG
C14orf1	SERPINB9
C14orf1	MPHOSPH6
C14orf1	HSPE1
DYNLL1	C14orf1
BTBD2	C14orf1
C14orf1	BAIAP2
UBC	C12orf57
UBC	C12orf5
UBC	C12orf4
UBC	C12orf29
UBC	C12orf10

UBC	C11orf83
UBC	C11orf80
EIF4H	C11orf68
UBC	C11orf58
A2M	C11orf58
UBC	C11orf54
TRIP13	C11orf54
UBC	C11orf49
ARHGEF15	C11orf49
UBC	C11orf48
EWSR1	C11orf16
UBC	C10orf76
FXR2	C10orf62
UBC	C10orf54
EHMT2	C10orf12
KRT15	C10orf10
CCDC85B	C10orf10
UBC	BZW2
UBC	BZW1
WDR3	BYSL
UBC	BYSL
MAP3K14	BYSL
CSNK1E	BYSL
UBC	BUD31
UBC	BUB3
BUB3	CDC20
BUB3	BUB1B
BUB3	BUB1
BUB3	ANAPC4
ANAPC4	BUB1B
UBC	BUB1
BUB1B	BUB1
APC	BUB1
ZC3H12A	BTRC
YAP1	BTRC
USP47	BTRC
UBE2D2	BTRC
UBC	BTRC
TRIM9	BTRC
TP53	BTRC
SUFU	BTRC
SNAI1	BTRC
SMAD4	BTRC
SKP1	BTRC

RELA	BTRC
RASSF1	BTRC
PSMD4	BTRC
PHLPP1	BTRC
PER1	BTRC
PDCD4	BTRC
NFKBIA	BTRC
NFKB2	BTRC
NFKB1	BTRC
NEDD8	BTRC
MAPK14	BTRC
KDR	BTRC
JUP	BTRC
IKBKB	BTRC
IFNAR1	BTRC
GLI3	BTRC
GHR	BTRC
FBXW11	BTRC
DEPTOR	BTRC
CUL1	BTRC
CTNNB1	BTRC
CLSPN	BTRC
CHUK	BTRC
CDC34	BTRC
CDC25B	BTRC
CDC25A	BTRC
CD4	BTRC
CCNE1	BTRC
BST2	BTRC
BORA	BTRC
BCL2L11	BTRC
AXIN1	BTRC
ATF4	BTRC
APC	BTRC
UBC	BTN3A1
UBC	BTN2A1
XDH	BTN1A1
BTLA	VTCN1
UBC	BTG2
PRMT1	BTG2
HOXC8	BTG2
BTG2	HOXB9
BTG2	CNOT8
CNOT7	BTG2

PRMT1	BTG1
CNOT8	BTG1
CNOT7	BTG1
UBC	BTF3L4
UBC	BTF3
MMP9	BTC
EGFR	BTC
UBC	BTBD3
UBC	BTAF1
TBP	BTAF1
UBC	BST2
UBC	BSCL2
TMEM19	BSCL2
SMIM3	BSCL2
UBC	BRWD1
CUL4A	BRWD1
UBC	BRSK2
KAT6A	BRPF1
ING5	BRPF1
UBC	BROX
CHMP4B	BROX
HDAC2	BRMS1
UBC	BRK1
BRIX1	UBA2
BRIX1	NOP56
BRIX1	EBNA1BP2
BRIX1	CD48
UBC	BRI3BP
TRAF6	BRI3BP
BRF2	RBL2
UBC	BRF1
TP53	BRF1
TBP	BRF1
RB1	BRF1
CSNK2A1	BRF1
UIMC1	BRE
UBC	BRE
FAM175B	BRE
BRCA1	BRE
BRE	BABAM1
SERPINB9	BRD7
PSMD11	BRD7
BTBD2	BRD7
BRD7	AGR2

UBC	BRD4
TCERG1	BRD4
MED1	BRD4
HIST4H4	BRD4
BRD4	HEXIM1
BRD4	CLDN1
HIST4H4	BRD2
E2F2	BRD2
ZNF512B	BRD1
UBC	BRD1
XRCC3	BRCA2
UBC	BRCA2
TP53	BRCA2
SMAD3	BRCA2
SHFM1	BRCA2
RAD51	BRCA2
PLK1	BRCA2
PALB2	BRCA2
MCPH1	BRCA2
KAT2B	BRCA2
H2AFX	BRCA2
FANCG	BRCA2
FANCD2	BRCA2
DMC1	BRCA2
CHEK2	BRCA2
BUB1B	BRCA2
BRCA1	BRCA2
BRCA1	ZNF350
VCP	BRCA1
BRCA1	USP2
BRCA1	USF2
BRCA1	UIMC1
UBE2W	BRCA1
UBE2N	BRCA1
UBE2L3	BRCA1
UBE2K	BRCA1
BRCA1	UBE2E3
UBE2E1	BRCA1
UBE2D3	BRCA1
UBC	BRCA1
BRCA1	TP53BP1
TP53	BRCA1
TOPBP1	BRCA1
SUMO1	BRCA1

BRCA1	STAT5A
BRCA1	STAT3
SP1	BRCA1
RPA1	BRCA1
BRCA1	RFC1
RBBP4	BRCA1
RAD51	BRCA1
PPP1CA	BRCA1
BRCA1	POLR2K
PIAS1	BRCA1
PGR	BRCA1
BRCA1	PALB2
BRCA1	NMI
BRCA1	NELFB
NCOA3	BRCA1
NCOA2	BRCA1
BRCA1	NBN
MYC	BRCA1
BRCA1	MRPL36
BRCA1	MRE11A
BRCA1	MED17
MED1	BRCA1
MDC1	BRCA1
LMO4	BRCA1
KPNA1	BRCA1
BRCA1	JUND
BRCA1	JUNB
JAK2	BRCA1
BRCA1	JAK1
HDAC2	BRCA1
HDAC1	BRCA1
FANCD2	BRCA1
ESR1	BRCA1
DHX9	BRCA1
CTBP1	BRCA1
BRCA1	CSTF1
BRCA1	CREB1
BRCA1	CLSPN
BRCA1	CHEK2
CDK4	BRCA1
CCNA2	BRCA1
BRCA1	BRIP1
BRCA1	BRAP
BLM	BRCA1

BRCA1	BAP1
BRCA1	BABAM1
BRCA1	ATM
BRCA1	ATF1
BRCA1	ANTXR1
BRCA1	ACACA
ABL1	BRCA1
UBC	BRAP
HRAS	BRAP
YWHAB	BRAF
UBC	BRAF
BRAF	TERF2IP
BRAF	SGK1
BRAF	RAP1GAP
BRAF	RAP1A
RAF1	BRAF
BRAF	MRAS
BRAF	MAPK1
BRAF	MAP2K2
BRAF	MAP2K1
BRAF	HRAS
USF1	BPTF
UBC	BPTF
SUMO2	BPTF
MAZ	BPTF
UBC	BPNT1
UBC	BPGM
PLK1	BORA
ARHGAP1	BNIPL
UBC	BNIP3L
STEAP3	BNIP3L
BNIP3	BNIP3L
UBC	BNIP2
CREB3	BNIP2
CDC42	BNIP2
BNIPL	BNIP2
BCL2	BNIP2
ARHGAP1	BNIP2
UBC	BNIP1
UBC	BMS1
SUMO2	BMS1
UBC	BMPR2
TGFBR1	BMPR2
LIMK1	BMPR2

GDF9	BMPR2
GDF5	BMPR2
BMPR1A	BMPR2
BMP7	BMPR2
BMP6	BMPR2
BMP4	BMPR2
BMP2	BMPR2
ACVR1	BMPR2
VEPH1	BMPR1B
BMPR1B	USP45
BMPR1B	UBE2Z
UBA52	BMPR1B
TTC27	BMPR1B
BMPR1B	TTC17
TGFBR1	BMPR1B
STUB1	BMPR1B
STK35	BMPR1B
SQSTM1	BMPR1B
SOCS6	BMPR1B
SMAD6	BMPR1B
RRAS2	BMPR1B
RHOJ	BMPR1B
RHOBTB1	BMPR1B
RHEBL1	BMPR1B
RASL12	BMPR1B
RASD2	BMPR1B
RAP2A	BMPR1B
RAN	BMPR1B
RAB6B	BMPR1B
BMPR1B	RAB38
BMPR1B	RAB25
PPP3CC	BMPR1B
PPP2CA	BMPR1B
PLEKHB1	BMPR1B
BMPR1B	PAK1
OXSR1	BMPR1B
LZTR1	BMPR1B
ILKAP	BMPR1B
GDF9	BMPR1B
BMPR1B	GDF5
FKBP1B	BMPR1B
BMPR1B	FBXO3
BMPR1B	FBXO24
BMPR1B	DCAF7

BMPR1B	DCAF6
BMPR1B	DCAF12
CDK4	BMPR1B
BMPR1B	BMPR2
BMPR1A	BMPR1B
BMPR1B	BMP7
BMPR1B	BMP6
BMP4	BMPR1B
BMP2	BMPR1B
BMPR1B	AMHR2
SMAD1	BMPR1A
BMPR1A	HOXC8
BMPR1A	BMP6
UBC	BMP7
BMPR1A	BMP7
ACVR2B	BMP7
ACVR2A	BMP7
SMAD5	BMP6
BMP4	BMPR1A
BMP4	BMP8B
BMP2	SOSTDC1
BMP2	MGP
COL2A1	BMP2
BMPR1A	BMP2
BMP2	ASCL1
ACVR2B	BMP2
ACVR2A	BMP2
ACVR1	BMP2
BCL2L2	BMF
BCL2L1	BMF
WDYHV1	BLOC1S6
PLEKHF2	BLOC1S6
LURAP1	BLOC1S6
BLOC1S6	CCDC53
BLOC1S6	BLOC1S5
UBC	BLOC1S1
PBX2	BLOC1S1
MAX	BLOC1S1
UBC	BLMH
BLMH	TRIO
WRN	BLM
TP53	BLM
BLM	RPA1
BLM	RMI2

BLM	RAD51
BLM	FANCM
BLM	ATM
UBE3A	BLK
PLCG2	BLK
EGFR	BLK
CBL	BLK
BCR	BLK
BCL2	BLK
BCAS2	BLK
BIRC2	BIRC7
UBC	BIRC5
DIABLO	BIRC5
BIRC5	CDK4
BIRC5	CDCA8
CASP9	BIRC5
CASP7	BIRC5
CASP3	BIRC5
BIRC5	BIRC2
UBE2D3	BIRC3
UBC	BIRC3
TRAF6	BIRC3
TRAF2	BIRC3
TRAF1	BIRC3
TNFRSF1A	BIRC3
RIPK1	BIRC3
NFKB1	BIRC3
HTRA2	BIRC3
GSPT1	BIRC3
DIABLO	BIRC3
CASP9	BIRC3
CASP3	BIRC3
BCL10	BIRC3
BIRC2	TRAF1
TNFRSF1A	BIRC2
BIRC2	TANK
BIRC2	LTBR
GSPT1	BIRC2
BIRC2	BCL10
SYNM	BIN1
SYNJ1	BIN1
SNX4	BIN1
MYC	BIN1
ITGA3	BIN1

DNM1	BIN1
AP2A2	BIN1
BCL2L2	BIK
BCL2L1	BIK
UBC	BID
BID	GZMB
BID	CASP1
BID	BCL2L2
BID	BAK1
BID	ATM
RAB6A	BICD2
DCTN1	BICD2
HDAC1	BHLHE41
BHLHE40	BHLHE41
BHLHE40	VIM
UBC	BHLHE40
TP53	BHLHE40
BHLHE40	BRD7
GGCX	BGLAP
BFSP1	BFSP2
UBE2D2	BFAR
UBC	BFAR
YKT6	BET1L
UBC	BET1L
STX5	BET1L
UBC	BET1
PPP2R5A	BEST1
U2AF2	BEND7
SDCBP2	BEND7
PSMF1	BEND7
NIF3L1	BEND7
USP2	BEND5
GRAP2	BEND5
DDR1	BEND5
BEGAIN	ZNF250
BEGAIN	USP2
BEGAIN	TRIM27
BEGAIN	RIBC2
BEGAIN	RBL1
BEGAIN	HID1
BEGAIN	FAM107A
BEGAIN	DTNB
BEGAIN	DLG1
BEGAIN	CATSPER1

BEGAIN	ABLIM1
RB1	BDP1
NTRK2	BDNF
NTF4	BDNF
NTF3	BDNF
NOS3	BDNF
BDKRB2	NOS3
NOS1	BDKRB2
AGTR1	BDKRB2
UBC	BDH2
UBC	BCS1L
DDX24	BCS1L
VAV1	BCR
UBC	BCR
TP53	BCR
BCR	SOS1
PTPN6	BCR
BCR	PIK3CG
JAK2	BCR
BCR	IGSF21
BCR	HSP90AA1
HCK	BCR
GRB2	BCR
BCR	FES
BCR	ERCC3
CRKL	BCR
CRK	BCR
BCR	ABL2
ABL1	BCR
YWHAG	BCLAF1
UBC	BCLAF1
PIN1	BCLAF1
ESR1	BCLAF1
PYGO1	BCL9
RELB	BCL7A
UBC	BCL6
NCOR2	BCL6
BCL6	HDAC4
UBC	BCL3
TBP	BCL3
RXRA	BCL3
RUVBL2	BCL3
RELB	BCL3
NFKB2	BCL3

NFKB1	BCL3
NCOA1	BCL3
KAT5	BCL3
JUN	BCL3
HDAC3	BCL3
HDAC1	BCL3
GTF2A1	BCL3
FYN	BCL3
FOS	BCL3
CTBP2	BCL3
CTBP1	BCL3
CREBBP	BCL3
COPS5	BCL3
BARD1	BCL3
UBC	BCL2L2-PABPN1
UBC	BCL2L12
UBC	BCL2L11
DYNLL1	BCL2L11
CUL2	BCL2L11
BCL2L11	BCL2L2
BCL2L11	BCL2L1
BCL2L11	BCL2A1
BCL2L11	BCL2
BAX	BCL2L11
APAF1	BCL2L10
UBC	BCL2L1
TP53	BCL2L1
BCL2L1	PSEN1
PPP1CA	BCL2L1
PMAIP1	BCL2L1
BCL2L1	HRK
FKBP8	BCL2L1
BCL2L1	CFLAR
BCL2L1	BNIPL
BID	BCL2L1
BCL2L1	BCL2L14
BAK1	BCL2L1
BCL2L1	AVEN
APAF1	BCL2L1
BCL2L1	ANTXR1
BIK	BCL2A1
BID	BCL2A1
BAX	BCL2A1
BAK1	BCL2A1

UBC BCL2
TP53BP2 BCL2
TP53 BCL2
BCL2 TMBIM6
SF1 BCL2
RTN4 BCL2
RRAS BCL2
RHEB BCL2
RAF1 BCL2
BCL2 RAD9A
PSEN1 BCL2
BCL2 PPP3CA
PPP2R5A BCL2
PMAIP1 BCL2
PIN1 BCL2
PARP1 BCL2
BCL2 NLRP1
BCL2 MOAP1
BCL2 MAPK1
HSP90AA1 BCL2
BCL2 HRK
FKBP8 BCL2
BCL2 CEBPB
CASP3 BCL2
BCL2 BNIPL
BCL2 BNIP3L
BNIP3 BCL2
BCL2 BMF
BCL2 BIK
BID BCL2
BCL2L1 BCL2
BAX BCL2
BAK1 BCL2
BCL2 BAG4
BCL2 BAG3
BAD BCL2
ATP2A2 BCL2
HDAC2 BCL11B
UBC BCL11A
NR2F1 BCL11A
HDAC1 BCL11A
BCL6 BCL11A
BCKDHA BCKDHB
UBC BCCIP

ITSN2	BCCIP
UBC	BCAT2
UBC	BCAT1
UBC	BCAS2
PLRG1	BCAS2
DYNLL1	BCAS1
ERBB3	BCAR3
BCAR1	VPS11
UBC	BCAR1
PXN	BCAR1
BCAR1	PTPRH
PTPRF	BCAR1
PTPN12	BCAR1
BCAR1	PTK2B
BCAR1	PPP1R15A
PIK3R1	BCAR1
NPHP1	BCAR1
BCAR1	LYN
LCK	BCAR1
INPPL1	BCAR1
HSPA5	BCAR1
HCK	BCAR1
GRB2	BCAR1
FYN	BCAR1
FXYD6-FXYD2	BCAR1
BCAR1	DOCK1
CSPG4	BCAR1
CRKL	BCAR1
CRK	BCAR1
UBC	BCAP29
BCAN	MMP8
MMP2	BCAN
BCAN	MMP10
LAMA5	BCAM
BBS5	BBS9
UBC	BBS7
CCT2	BBS7
BBS2	BBS7
BBS12	BBS7
BBS1	BBS7
DCTN1	BBS4
UBC	BBS2
BBS2	BBS9
UBC	BBS10

RAB3IP	BBS1
PARK7	BBS1
BBS1	BBS9
HSPB3	BBOX1
UBC	BAZ2A
UBC	BAZ1B
UBC	BAZ1A
SMAD2	BAZ1A
POLE3	BAZ1A
UBC	BAX
BAX	SH3GLB1
BAX	SFN
BID	BAX
BAX	BCL2L2
BAX	BCL2L1
BAX	BAK1
BATF3	CREB3L3
ATF5	BATF3
JUNB	BATF2
DAZAP2	BATF2
BATF	TEF
SNAPC5	BATF
NFIL3	BATF
JUND	BATF
JUNB	BATF
HLF	BATF
FOS	BATF
BATF3	BATF
ATF5	BATF
UBC	BASP1
RIPK3	BASP1
XRCC6	BARD1
XRCC5	BARD1
UIMC1	BARD1
UBE2D3	BARD1
UBE2D1	BARD1
UBC	BARD1
TP53	BARD1
BARD1	RBBP8
POU2F1	BARD1
POLR2A	BARD1
NR1H3	BARD1
MSH6	BARD1
BARD1	HIST2H2AC

H2AFX	BARD1
BARD1	FAM175A
CSTF1	BARD1
BARD1	BRCA2
BRCA1	BARD1
AURKB	BARD1
UBC	BAP1
UBC	BANF1
TMPO	BANF1
PXN	BANF1
EHMT2	BANF1
BAMBI	SOX30
UBC	BAIAP2L1
SHANK2	BAI2
SHANK1	BAI2
MAGI3	BAI1
MAGI1	BAI1
DLG4	BAI1
BAIAP3	BAI1
BAIAP2	BAI1
TRAF2	BAHD1
MDFI	BAHD1
DVL2	BAHD1
BEGAIN	BAHD1
BAG6	STX5
BAG6	SPP1
BAG6	PTH
BAG6	NOMO1
BAG6	KLHL12
BAG6	IQCG
BAG6	FAF2
BAG6	DNAJB11
BAG6	CHN2
BAG6	AMFR
UBC	BAG5
VCL	BAG4
TNFRSF1A	BAG4
UBC	BAG3
MCM7	BAG3
HSPA8	BAG3
BAG3	FAM189A2
DAZAP2	BAG3
UBC	BAG2
TRAF2	BAG2

TRAF1	BAG2
TNFRSF1B	BAG2
TNFRSF1A	BAG2
TAB2	BAG2
STUB1	BAG2
MAP3K14	BAG2
HSPA8	BAG2
VDR	BAG1
UBC	BAG1
STUB1	BAG1
SIAH1	BAG1
RB1	BAG1
RARA	BAG1
RAF1	BAG1
PPP1R15A	BAG1
PDGFRB	BAG1
NR3C1	BAG1
MET	BAG1
HSPA8	BAG1
HSPA4	BAG1
HSPA1A	BAG1
HBEGF	BAG1
ESR1	BAG1
BCL2L1	BAG1
BCL2	BAG1
YWHAE	BAD
BAD	SFN
RAF1	BAD
BAD	BCL2L2
BAD	BCL2L1
BAD	BCL2A1
UBC	BACH2
MAFK	BACH2
BACH2	MAFB
CREB5	BACH2
UBC	BACH1
MAFK	BACH1
MAFB	BACH1
HMMR	BACH1
CREB5	BACH1
BRCA1	BACH1
BARD1	BACH1
ATF1	BACH1
UIMC1	BABAM1

UBC BABAM1
FAM175B BABAM1
MDFI BAALC
UBC B9D1
EXOSC10 B9D1
UBC B4GALT1
LALBA B4GALT1
UBC B3GNTL1
VCL B2M
UBC B2M
B2M LILRB1
HLA-G B2M
B2M FCGRT
B2M CD1B
B2M CD1A
CALR B2M
BAG6 B2M
UBC AZIN1
UBC AZI2
TBK1 AZI2
UBC AZGP1
LCK AXL
UBC AXIN2
GSK3B AXIN2
APC AXIN2
AXIN2 ANKRD6
UBC AXIN1
TP53 AXIN1
SUMO1 AXIN1
AXIN1 SH3GL1
AXIN1 PPP2R5A
LRP6 AXIN1
JUP AXIN1
AXIN1 HIPK2
GSK3B AXIN1
GSK3A AXIN1
DVL2 AXIN1
CSNK1EAXIN1
AXIN1 CSNK1A1
AXIN1 APC
AVPR1BAVP
GRK5 AVPR1A
AVPOXTR
APAF1 AVEN

UBC	AURKC
UBC	AURKB
TGFBR1	AURKB
SUMO2	AURKB
SUMO1	AURKB
AURKB	SEPT1
RASA1	AURKB
RACGAP1	AURKB
MAPRE1	AURKB
KLHL9	AURKB
AURKB	INCENP
HIST3H3	AURKB
AURKB	HIST2H3C
HIST1H3A	AURKB
AURKB	CENPA
AURKB	CDCA8
CBX5	AURKB
BIRC5	AURKB
ANKZF1	AURKB
AURKA	AURKAIP1
UBE2N	AURKA
UBC	AURKA
TPX2	AURKA
TP53	AURKA
TACC1	AURKA
PLK1	AURKA
NME1	AURKA
MYCN	AURKA
LATS2	AURKA
HIST3H3	AURKA
FBXW7	AURKA
CDC20	AURKA
BRCA1	AURKA
AUP1	UBE2J1
UBC	AUP1
AUP1	SEL1L
KAT2A	ATXN7L3
TRRAP	ATXN7
ATXN7	TAF12
ATXN7	PSMC1
KAT2A	ATXN7
ATXN7	CRX
ATXN7L3	ATXN7
UBE2L3	ATXN3

GSK3B ATXN3
UBC ATXN2L
UBC ATXN2
SH3GL3 ATXN2
SH3GL2 ATXN2
PABPC1 ATXN2
CHGB ATXN2
ATXN1 ATXN1L
UBC ATXN10
ATXN1 OTX2
UBC ATRN
ATRXPA
WRN ATR
UBC ATR
TP53 ATR
TELO2 ATR
RPA1 ATR
ATRRAD17
NBN ATR
ATRMSH6
H2AFX ATR
CLSPN ATR
ATRCLK2
CHEK2 ATR
CHD4 ATR
BRCA1 ATR
BID ATR
ATM ATR
ABL1 ATR
ATP5B ATPAF1
UBC ATP9A
UBC ATP7B
COMMD1 ATP7B
CLU ATP7B
ATOX1 ATP7B
UBC ATP6V1H
ETHE1 ATP6V1H
ATP6V1E1 ATP6V1G1
UBC ATP6V1E1
ATP6V1E1 SOS1
ATP6V1E1 ATP6V1H
ATP6V1E1 ATP6V1B2
ATP6V1E1 ATP6V1B1
ATP6V1E1 ALDOB

ATP6V1E1	ALDOA
ATP6V1E1	ATP6V1D
ATP6V1B2	ATP6V1D
ATP6V1B1	ATP6V1D
UBC	ATP6V1C1
SARS	ATP6V1C1
ATP6V1E1	ATP6V1C1
ATP6V1B2	ATP6V1B1
UBC	ATP6V1A
PFKM	ATP6V0A4
UBC	ATP6V0A2
UBC	ATP6V0A1
ATP6V1B1	ATP6V0A1
UBC	ATP5O
ATP5B	ATP5O
ATP5A1	ATP5O
UBC	ATP5L
UBC	ATP5J2
ATP5J2	RPLP1
ATP5J2	PVRL2
ATP5J2	PRPF4
ATP5J2	MYC
ATP5J2	LPP
KDM1A	ATP5J2
HAP1	ATP5J2
ATP5J2	FBP1
ATP5J2	CAPNS1
UBC	ATP5J
UBC	ATP5G1
UBC	ATP5F1
ATP5C1	ZNF337
UBC	ATP5C1
POLA2	ATP5C1
ATP5C1	NDUFB3
EGFR	ATP5C1
DPP3	ATP5C1
CCT7	ATP5C1
ATP5B	VCL
UBC	ATP5B
SUMO1	ATP5B
ATP5B	PLG
UBC	ATP5A1
ATP5A1	TNFRSF1B
ATP5A1	TNFRSF1A

ATP5A1	TBK1
SUMO1	ATP5A1
ATP5A1	MAP3K14
ATP5A1	ATP5B
UBC	ATP4A
UBC	ATP2C1
UBC	ATP2B4
DLG1	ATP2B2
UBC	ATP2A2
TRAF6	ATP2A2
TNFRSF1B	ATP2A2
TNFRSF1A	ATP2A2
ATP2A2	S100A1
ATP2A2	PLN
UBC	ATP2A1
TNFRSF1B	ATP2A1
SLN	ATP2A1
PLN	ATP2A1
IRS2	ATP2A1
IRS1	ATP2A1
UBC	ATP1B3
UBC	ATP1B1
UBC	ATP1A4
UBC	ATP1A3
UBC	ATP1A2
UBC	ATP1A1
ATP1A1	TPT1
TNFRSF1B	ATP1A1
TNFRSF1A	ATP1A1
ATP1A1	FXYD7
UBC	ATP13A3
UBC	ATP13A2
UBC	ATP13A1
UBC	ATP12A
UBC	ATP11B
UBC	ATP10D
VIM	ATN1
ATN1	SIAH1
RUNX1T1	ATN1
ATN1	RERE
ATN1	PDCD6IP
ATN1	MAGI1
KAT7	ATN1
CASP3	ATN1

ATN1	CASP1
BAIAP2	ATN1
WRN	ATM
TELO2	ATM
NBN	ATM
FANCD2	ATM
ATM	EIF4EBP1
ATM	AP1B1
UBC	ATL3
UBC	ATL2
UBC	ATL1
UBC	ATIC
UBC	ATG7
GABARAPL2	ATG7
UBC	ATG5
TECPR1	ATG5
ATG16L1	ATG5
ATG12	ATG5
UBC	ATG4B
GABARAPL2	ATG4B
GABARAPL1	ATG4B
GABARAP	ATG4B
UBC	ATG3
GABARAPL2	ATG3
GABARAPL1	ATG3
GABARAP	ATG3
ATG7	ATG3
ATG12	ATG3
ATG13	ULK1
GABARAPL2	ATG13
UBC	ATG12
ATG12	TECPR1
ATG12	ATG7
ATG12	ATG16L1
ATG12	ATG10
UBC	ATF7IP
SUMO2	ATF7IP
SP1	ATF7IP
SMAD4	ATF7IP
SETDB1	ATF7IP
MBD1	ATF7IP
GTF2H1	ATF7IP
GTF2F1	ATF7IP
GTF2E2	ATF7IP

UBC	ATF7
ATF7	PTP4A1
ATF7	MAPK9
ATF6B	PHOX2A
UBC	ATF6
UBC	ATF4
ATF4	TRIB3
ATF4	PTCD3
NFE2L1	ATF4
ATF4	MAF
ATF4	HLF
ATF4	GTF2F2
ATF4	FOSL1
ATF4	FOSB
FOS	ATF4
CREBZF	ATF4
ATF4	CEBPB
CCDC106	ATF4
ATF4	BATF3
ATF4	BATF
UBC	ATF3
TP53	ATF3
SMARCA4	ATF3
SMAD3	ATF3
NUF2	ATF3
MDM2	ATF3
JUND	ATF3
JUNB	ATF3
JUN	ATF3
IGSF21	ATF3
HDAC1	ATF3
ATF3	ELAVL1
DDIT3	ATF3
CREB5	ATF3
ATF3	CEBPG
CEBPE	ATF3
BATF3	ATF3
ATF4	ATF3
YY1	ATF2
UBE2I	ATF2
UBC	ATF2
SMAD4	ATF2
RUVBL2	ATF2
RB1	ATF2

NCOA6	ATF2
MAPK9	ATF2
MAPK8	ATF2
MAPK14	ATF2
MAPK13	ATF2
JUND	ATF2
JUNB	ATF2
JUN	ATF2
JDP2	ATF2
HMGA1	ATF2
GTF2F2	ATF2
FOSL2	ATF2
FOSL1	ATF2
FOSB	ATF2
FOS	ATF2
EP300	ATF2
DDIT3	ATF2
CSNK2A2	ATF2
CREBBP	ATF2
CREB5	ATF2
CEBPG	ATF2
CEBPB	ATF2
CEBPA	ATF2
BATF3	ATF2
BATF	ATF2
BACH1	ATF2
ATM	ATF2
ATF3	ATF2
UBC	ATE1
USP1	ATAD5
UBC	ATAD5
UBC	ATAD3A
TNFRSF1A	ATAD3A
UBC	ATAD2
SUMO2	ATAD2
UBC	ATAD1
UBC	ASXL2
ASXL2	BAP1
UBC	ASXL1
RARA	ASXL1
BAP1	ASXL1
ZNF655	ASUN
UBC	ASUN
UBC	ASS1

ASCC2	ASS1
AKTIP	ASS1
UBC	ASPH
UBC	ASNS
MC1R	ASIP
PICK1	ASIC2
ASIC1	ASIC2
WDR5	ASH2L
USF1	ASH2L
UNC119	ASH2L
UBC	ASH2L
TGFBR1	ASH2L
SMAD9	ASH2L
ASH2L	SETD1A
RBBP5	ASH2L
PAXIP1	ASH2L
NCOA6	ASH2L
MEN1	ASH2L
HIST3H3	ASH2L
ASH2L	HCFC2
ESR1	ASH2L
ASH2L	DPY30
CXXC1	ASH2L
CTNNB1	ASH2L
UBC	ASH1L
PPIA	ASGR2
ERLEC1	ASGR2
CERS2	ASGR2
ASGR1	ASGR2
TG	ASGR1
HSP90B1	ASGR1
HSP90AA1	ASGR1
CERS2	ASGR1
MMS22L	ASF1B
H3F3A	ASF1B
CHAF1A	ASF1B
RBBP4	ASF1A
HIST4H4	ASF1A
HIST3H3	ASF1A
HIST1H3A	ASF1A
HIRA	ASF1A
H3F3A	ASF1A
ASF1B	ASF1A
UBC	ASCC3

TRIP4	ASCC3
SKIL	ASCC3
UBC	ASCC1
TCEB1	ASB8
UBC	ASB6
CUL5	ASB6
UBC	ASB2
TCEB2	ASB2
RNF7	ASB2
TRIP13	ASB13
UBC	ASAP2
RAN	ASAP2
PXN	ASAP2
ASAP2	PTK2B
GRB2	ASAP2
ARF6	ASAP2
ARF5	ASAP2
UBC	ASAP1
PXN	ASAP1
PACSIN3	ASAP1
ASAP1	PACSIN2
HCK	ASAP1
GRB2	ASAP1
FYN	ASAP1
CRK	ASAP1
UBC	ASAH1
TSC22D1	ASAH1
SETDB1	ASAH1
UBC	AS3MT
UBC	ARVCF
LNX1	ARVCF
ERBB2IP	ARVCF
CDH2	ARVCF
CDH15	ARVCF
UBC	ARV1
UBC	ARRDC4
NEDD4	ARRDC3
UBC	ARRDC1
ARRDC1	TSG101
ARRDC1	ITCH
UBC	ARRB2
ARRB2	TCOF1
ARRB2	PTAFR
NOLC1	ARRB2

ARRB2	NFKBIA
ARRB2	NDUFS7
ARRB2	MED8
MDM2	ARRB2
ARRB2	MAPK10
MAP3K5	ARRB2
ARRB2	MAP2K4
ITCH	ARRB2
ARRB2	ARF6
ARRB2	AP1B1
ARRB2	AGTR1
ZRANB2	ARRB1
ZBTB43	ARRB1
UBC	ARRB1
TRHR	ARRB1
TRAF6	ARRB1
ARRB1	TCOF1
ARRB1	PIK3R2
NOLC1	ARRB1
NFKBIA	ARRB1
MDM2	ARRB1
MAPK3	ARRB1
MAPK10	ARRB1
MAPK1	ARRB1
MAP3K5	ARRB1
ARRB1	GNMT
CLTC	ARRB1
ARRB1	CCR5
ARRB2	ARRB1
ARF6	ARRB1
ARRB1	ADRBK1
UBC	ARPC5L
ARPC4-TTLL3	ARPC5L
UBC	ARPC5
MAPKAPK2	ARPC5
ARPC4-TTLL3	ARPC5
ARPC1A	ARPC5
UBC	ARPC4-TTLL3
UBC	ARPC3
PRMT1	ARPC3
ARPC3	LTA4H
ARPC3	CETP
CDKN2B	ARPC3
BAG6	ARPC3

ARPC3	ARPC5L
ARPC3	ARPC5
ARPC3	ARPC4-TTLL3
ARPC3	ARPC1B
ARAP1	ARPC3
ARPC3	ACTR3B
UBC	ARPC2
ARPC2	ARPC5L
ARPC2	ARPC5
ARPC4-TTLL3	ARPC2
ARPC3	ARPC2
ARPC1B	ARPC2
ARPC1A	ARPC2
ARPC2	ACTR3B
ACTR2	ARPC2
WASL	ARPC1B
UBC	ARPC1B
ARPC1B	ARPC5
ARPC1B	ARPC4-TTLL3
ARPC1B	ACTB
CLOCK	ARNTL2
HSP90AA1	ARNTL
EPAS1	ARNTL
ARNTL	CLOCK
BHLHE40	ARNTL
ARNT	SIM2
ARNT	SIM1
GTF2F2	ARNT
KCTD13	ARMC7
CPSF6	ARMC7
UBC	ARMC6
UBC	ARMC1
UBC	ARL6IP5
SLC1A1	ARL6IP5
UBC	ARL6IP1
FXR2	ARL6IP1
FBXW7	ARL6IP1
BBS1	ARL6
UBC	ARL5B
SMAD4	ARL5B
ARL4D	UBR1
UBC	ARL4D
TGFBR1	ARL4D
SMAD2	ARL4D

SMAD1	ARL4D
ARL4D	PRKCSH
PGAM1	ARL4D
ARL4D	MAPK8IP3
ARL4D	EML4
ARL4D	EIF2B1
ARL4D	ARL6IP1
UBC	ARL4C
ARL3	UBR1
UBC	ARL3
ARL3	TUBA1A
TP53	ARL3
ARL3	PDE6D
ARL2	ARL2BP
UBC	ARL15
UBC	ARL14EP
UBC	ARL11
TRIP13	ARL11
UBC	ARL1
GOLGA4	ARL1
ARFIP2	ARL1
UBE2L6	ARIH2
UBE2L3	ARIH2
UBC	ARIH2
TP53	ARIH2
ARIH2	SLC1A6
ARIH2	SGCE
ARIH2	SCAMP2
RPL8	ARIH2
PTNARIH2	
KAT5	ARIH2
IL4R	ARIH2
ENSA	ARIH2
ARIH2	EML4
EEF1G	ARIH2
EEF1A1	ARIH2
DLST	ARIH2
CBR3	ARIH2
BUB1	ARIH2
ARAP1	ARIH2
UBE2L6	ARIH1
UBE2L3	ARIH1
UBC	ARIH1
EIF4E2	ARIH1

UBC	ARID5A
ATXN1	ARID5A
HDAC2	ARID4B
SIN3A	ARID4A
HDAC2	ARID4A
HDAC1	ARID4A
ARID4A	BRMS1
TP53	ARID3A
UBC	ARID2
SMARCE1	ARID2
SMARCC2	ARID2
SMARCA4	ARID2
UBC	ARID1B
SMARCE1	ARID1B
SMARCC1	ARID1B
SMARCB1	ARID1B
SMARCA4	ARID1B
SMARCA2	ARID1B
SMAD9	ARID1B
RELB	ARID1B
ARID1A	ARID1B
ARHGEF7	SHANK2
ARHGEF7	SCRIB
ARHGEF7	PPM1F
ARHGEF7	PAK2
APC	ARHGEF4
UBC	ARHGEF39
DVL2	ARHGEF39
UBC	ARHGEF2
MYC	ARHGEF2
UBC	ARHGEF12
ARHGEF12	GNA12
ABCA1	ARHGEF12
ARHGEF11	SLC1A6
CDC42	ARHGEF11
ABCA1	ARHGEF11
UBC	ARHGEF1
PRKCA	ARHGEF1
RHOB	ARHGDIG
PSMA2	ARHGDIG
CDC42	ARHGDIG
VAV1	ARHGDIB
UBC	ARHGDIB
ARHGDIB	RAC2

EZRARHGDIB
CDC42 ARHGDIB
CASP3 ARHGDIB
UBC ARHGDIA
SUMO1 ARHGDIA
ARHGDIA SH3GL3
NEDD4L ARHGDIA
ARHGDIA FEN1
ARHGDIA CDC42
UBC ARHGAP9
SMAD9 ARHGAP9
UBC ARHGAP8
HN1 ARHGAP44
UBC ARHGAP35
CRK ARHGAP32
CDC42 ARHGAP32
BCAR1 ARHGAP32
UBC ARHGAP26
UBC ARHGAP23
MPHOSPH6 ARHGAP18
SRPK2 ARGLU1
ARG1 ARG2
SMAD4 ARFRP1
CYTH1 ARFRP1
ARFIP2 RAC2
DTNBP1ARFIP2
ARF6 ARFIP2
ARF5 ARFIP2
ARFIP2 ARF3
UBC ARFIP1
UBC ARFGEF2
PRPF40A ARFGEF2
PRKAR1A ARFGEF2
ARFGEF1 ARFGEF2
VCL ARFGEF1
UBC ARFGEF1
PRKAR1A ARFGEF1
KIF21A ARFGEF1
UBC ARFGAP3
SMAD2 ARFGAP3
KIF23 ARF6
ARF6 CHRM3
UBC ARF3
GGA3 ARF3

ARF3	ARFIP1
UBC	ARF1
RALA	ARF1
PLD2	ARF1
PIP5K1A	ARF1
NPM1	ARF1
MDM4	ARF1
KDELR1	ARF1
GGA3	ARF1
GGA1	ARF1
CYTH2	ARF1
CYTH1	ARF1
COPE	ARF1
COPB1	ARF1
ASAP2	ARF1
ARHGAP21	ARF1
ARFIP2	ARF1
AP4M1	ARF1
AP3D1	ARF1
MMP9	AREG
EGFR	AREG
UBC	ARCN1
COPZ1	ARCN1
COPG2	ARCN1
COPG1	ARCN1
COPB2	ARCN1
COPB1	ARCN1
UBC	ARC
KRT15	ARC
UBC	ARAP3
UBC	AQR
MDFI	AQP5
PLD2	AQP3
CCDC85B	AQP1
APTX	XRCC1
RAB22A	APPL2
APPL1	APPL2
UBC	APPL1
TRAF2	APPL1
RAB5A	APPL1
MTA2	APPL1
HDAC2	APPL1
FSHR	APPL1
EGFR	APPL1

AKT2	APPL1
ADIPOR1	APPL1
APPBP2	WNT5B
UBC	APPBP2
APPBP2	SLC22A6
APPBP2	RRS1
APPBP2	PCSK5
APPBP2	MUL1
APPBP2	KIF6
APPBP2	FSTL1
APPBP2	EID3
CUL2	APPBP2
UBC	APP
APP	PRSS3
GSK3B	APP
FBLN1	APP
APP	BACE1
APP	APBB2
APP	APBA2
APP	A2M
KLF6	APOM
UBC	APOL6
UBC	APOL2
PLG	APOH
F10	APOH
MAPT	APOE
APOE	MAP2
APOE	LRP8
APOE	CTSB
APOE	APP
APOE	ALB
APOE	A2M
UBC	APOBEC3G
UBC	APOBEC3F
UBC	APOBEC3C
APOBEC1	APOBEC2
DNAJB11	APOBEC1
APOBEC1	CELF2
BAG4	APOBEC1
A1CF	APOBEC1
APOF	APOA2
APOD	APOA2
APOC1	APOA2
UBC	APOA1BP

APOA1 TTR
TNFRSF10C APOA1
APOA1 PCMT1
MAP7D1 APOA1
APOA1 APOC1
APOB APOA1
APOA1 APOA2
ABCA1 APOA1
UBC APMAP
ZNF512B APLP2
UBC APLP2
APLP2 APBB3
APBB2 APLP2
APLP1 ZNF24
TK1 APLP1
APLP1 TCF21
STX5 APLP1
PNP APLP1
PHF10 APLP1
MPHOSPH6 APLP1
HSPE1 APLP1
GSTM4 APLP1
APLP1 EIF2S2
APLP1 CRCT1
CDKN2C APLP1
APBB1 APLP1
APLF XRCC1
UBC APITD1-CORT
VPS25 APIP
VIM APIP
TRAF2 APIP
TRAF1 APIP
NUDT18 APIP
UBC API5
UBC APH1B
NCSTN APH1B
PSENEN APH1A
PSEN1 APH1A
NCSTN APH1A
APEX1 XRCC1
UBE2I APEX1
UBC APEX1
TP53 APEX1
APEX1 SET

APEX1	NME1
HSPA1A	APEX1
APEX1	HNRNPL
APEX1	GZMA
APEX1	FEN1
TG	APCS
FN1	APCS
FCGR3A	APCS
CRP	APCS
CALU	APCS
C4BPA	APCS
C1QA	APCS
RAB4A	APBB3
EGFR	APBB3
APP	APBB3
VASP	APBB1IP
GAS7	APBB1IP
APBB1	YBX1
APBB1	WBP11
APBB1	WASL
APBB1	WASF2
VASP	APBB1
UBC	APBB1
APBB1	U2AF2
THRAP3	APBB1
APBB1	TFCP2
APBB1	SFPQ
APBB1	SF3B4
SF3B3	APBB1
APBB1	SF3B1
APBB1	SF3A3
APBB1	SF3A2
APBB1	SF1
APBB1	RPL4
APBB1	RBM17
APBB1	PTBP1
APBB1	KHSRP
APBB1	KHDRBS1
KAT5	APBB1
HNRNPH1	APBB1
APBB1	FASLG
ENAH	APBB1
EGFR	APBB1
APBB1	DHX9

DHX15	APBB1
APBB1	DDX46
APBB1	DDX17
APBB1	CPSF7
APBB1	CPSF6
APBB1	CLSTN1
APBB1	CHERP
APBB1	CCDC97
APBB1	APP
APBB1	APLP2
APBB1	ABI1
EGFR	APBA3
APP	APBA3
PSEN1	APBA1
APBA1	KCNJ12
APP	APBA1
UBC	AP5B1
UBC	AP4S1
UBC	AP4M1
CD63	AP4M1
AP4M1	AP4B1
UBC	AP4B1
AP4B1	AP4E1
UBC	AP3S2
AP3S1	AP3S2
AP3D1	AP3S2
AP3B1	AP3S2
UBC	AP3M2
UBC	AP3M1
UBC	AP3D1
ARF6	AP3D1
AP3S1	AP3D1
AP3B1	AP3D1
AP3S1	AP3B2
UBC	AP3B1
ATM	AP3B1
AP3S1	AP3B1
STON2	AP2M1
UBC	AP2B1
LDLRAP1	AP2B1
ITSN1	AP2B1
CLTC	AP2B1
ARRB2	AP2B1
AP2S1	AP2B1

AP2M1	AP2B1
AP1M1	AP2B1
UBC	AP2A2
SMAD1	AP2A2
EPN1	AP2A2
DAB2	AP2A2
CLTC	AP2A2
AP2A2	AP2B1
UBC	AP1S1
AP1G2	AP1S1
AP1G1	AP1S1
UBC	AP1M2
AP1M2	AP2B1
AP1M1	AP1M2
AP1B1	AP1M2
UBC	AP1G2
RABEP1	AP1G2
AP1G1	CLINT1
AP1G1	AP2M1
AP1G1	AP1M1
AP1G1	AP1B1
CLINT1	AP1B1
AP2M1	AP1B1
AP1M1	AP1B1
UBC	AP1AR
UBC	ANXA6
S100A1	ANXA6
ANXA6	CR2
UBC	ANXA5
SUPT4H1	ANXA5
KDR	ANXA5
ITGB5	ANXA5
EIF4G1	ANXA5
EED	ANXA5
COL2A1	ANXA5
ANXA5	COL10A1
ACTG1	ANXA5
UBC	ANXA4
ANXA3	UBR1
UBC	ANXA3
TP53	ANXA3
VCL	ANXA2
UBC	ANXA2
PXN	ANXA2

PLGANXA2
PHB	ANXA2
ANXA2	APOH
NEDD4	ANXA13
UBC	ANXA11
S100A6	ANXA11
PLSCR1	ANXA11
PDCD6	ANXA11
HNRNPH3	ANXA11
ALG2	ANXA11
UBC	ANXA1
S100A11	ANXA1
EGFR	ANXA1
UBC	ANP32B
SUMO2	ANP32B
ELAVL1	ANP32B
UBC	ANP32A
TRAF1	ANP32A
THRB	ANP32A
SET	ANP32A
RARA	ANP32A
NSFL1C	ANP32A
HSPB3	ANP32A
HIST4H4	ANP32A
HIST1H3A	ANP32A
ESR1	ANP32A
ANP32A	ELAVL1
AXIN1	ANP32A
ATXN1	ANP32A
COQ9	ANO8
UBC	ANO6
LRP2	ANKS1B
ITGB1	ANKS1B
ERBB2	ANKS1B
EGFR	ANKS1B
YWHAG	ANKS1A
EGFR	ANKS1A
UBC	ANKRD46
UBC	ANKRD42
UBC	ANKRD40
UBC	ANKRD32
ZNF512B	ANKRD28
UBC	ANKRD28
UBC	ANKRD27

SMAD1	ANKRD27
UNC119	ANKRD24
UBC	ANKRD17
BAP1	ANKRD17
UBC	ANKRD13B
UBC	ANKRD13A
SMAD5	ANKRD13A
LAPTM5	ANKRD13A
EGFR	ANKRD13A
ANKRD1	MYPN
HDAC4	ANKRA2
ZNF512B	ANKLE2
ZFYVE9	ANKLE2
UBC	ANKLE1
UBC	ANKIB1
UBC	ANKHD1-EIF4EBP3
UBC	ANKH
UBC	ANKFY1
UBC	ANK3
SMAD2	ANK3
UBC	ANK2
ANK2	SLC8A1
TIAM1	ANK1
ANK1	SLC4A3
TEK	ANGPTL1
TEK	ANGPT4
TEK	ANGPT2
TEK	ANGPT1
ANGPT2	ANGPT1
UBC	ANGEL2
PTEN	ANG
UBC	ANAPC7
PTTG1	ANAPC7
MDC1	ANAPC7
CDC20	ANAPC7
CDC16	ANAPC7
ANAPC7	BUB1B
ANAPC5	ANAPC7
ANAPC7	ANAPC4
ANAPC7	ANAPC1
CDC16	ANAPC4
ANAPC5	ANAPC4
ANAPC4	ANAPC15
UBC	ANAPC10

SMAD2 ANAPC10
CDC20 ANAPC10
CDC16 ANAPC10
ANAPC4 ANAPC10
PTTG1 ANAPC1
MDC1 ANAPC1
CDC16 ANAPC1
ANAPC5 ANAPC1
ANAPC4 ANAPC1
VTN AMPH
AMPH SYNJ1
AMPH SH3GL2
AMPH PLD2
DNM2 AMPH
AMPH DNM1
CLTC AMPH
AMPH CDK5R1
AMPH AP2A2
UBC AMPD2
MYH2 AMPD1
UBC AMOTL2
KRT15 AMOTL2
GNG11 AMOTL2
GCC1 AMOTL2
FXR2 AMOTL2
UBC AMOTL1
NEDD4 AMOTL1
VIMP AMFR
INSIG1 AMFR
UBC AMD1
ULK1 AMBRA1
UBC AMBRA1
DDA1 AMBRA1
CUL4A AMBRA1
F2 AMBP
CD79A AMBP
ALB AMBP
A2M AMBP
UBC AMACR
IPO13 ALX1
ALX4 ALX1
PIGK ALPP
UBC ALOXE3
ALOX5 ALOX5AP

MAPKAPK2	ALOX5
COTL1	ALOX5
KRT5	ALOX12B
LMNA	ALOX12
ITGB4	ALOX12
UBC	ALKBH5
UBC	ALKBH2
UBC	ALG8
UBC	ALG5
UBC	ALG3
ANXA7	ALG2
UBC	ALG14
UBC	ALG1
UBC	ALDOC
ALDOA	ALDOC
ALDOA	GPD2
DYNLL1	ALDOA
UBC	ALDH9A1
UBC	ALDH7A1
RECQL5	ALDH7A1
UBC	ALDH2
ALDH2	IGSF21
HSPE1	ALDH2
ALDH2	HSPD1
C14orf1	ALDH2
UBC	ALDH1A3
UBC	ALDH18A1
SUMO1	ALDH18A1
UBC	ALB
ALB	SRGN
ALB	CRYAA
UBC	ALAS1
RPA1	AKTIP
POLA2	AKTIP
AKTIP	HOOK3
AKTIP	HOOK1
EXOC7	AKTIP
AKTIP	C10orf2
UBC	AKT3
AKT3	TCL1A
UBC	AKT2
AKT2	TCL1A
CHUK	AKT2
AKT1S1	AKT2

AKT1S1	TCEA2
AKT1S1	RPTOR
YBX1	AKT1
VCP	AKT1
UBC	AKT1
TUBB	AKT1
TSC1	AKT1
AKT1	THEM4
TERT	AKT1
AKT1	TCL1B
AKT1	TCL1A
STUB1	AKT1
AKT1	STK4
STK3	AKT1
SMAD4	AKT1
SKP2	AKT1
RPS6KB1	AKT1
AKT1	RICTOR
RAF1	AKT1
PTPN1	AKT1
PTEN	AKT1
PRKDC	AKT1
AKT1	PRKCZ
AKT1	PRKCQ
PRG2	AKT1
PPP2R4	AKT1
PPP2CA	AKT1
PPL	AKT1
PKN2	AKT1
PIK3R1	AKT1
AKT1	PHLPP1
PEA15	AKT1
PAK1	AKT1
AKT1	NOS3
NEDD4	AKT1
MXD1	AKT1
MTOR	AKT1
MDM2	AKT1
MAPKAP1	AKT1
MAPK8IP1	AKT1
MAPK14	AKT1
MAP3K8	AKT1
MAP3K11	AKT1
AKT1	MAP2K4

KRT10 AKT1
IRS1 AKT1
ILK AKT1
HTT AKT1
HSPB1 AKT1
HSP90AA1 AKT1
GSK3B AKT1
GSK3A AKT1
AKT1 GRB10
GAB2 AKT1
FOXO3 AKT1
FOXO1 AKT1
FANCA AKT1
EP300 AKT1
CTNNB1 AKT1
COMMD1 AKT1
CHUK AKT1
CDKN1B AKT1
CASP3 AKT1
BRCA1 AKT1
BRAF AKT1
BAD AKT1
ATXN1 AKT1
ARRB2 AKT1
ARRB1 AKT1
APPL1 AKT1
AKTIP AKT1
AKT2 AKT1
AKT1S1 AKT1
UBC AKR7A2
AKR7A3 AKR7A2
AKR1C3 UBE2W
AKR1C3 ACIN1
UBC AKR1B1
SMAD1 AKR1B1
UBC AKR1A1
UBC AKNA
SPG21 AKIRIN2
CCDC85B AKIRIN2
UBC AKAP9
CSNK1E AKAP9
CLIC1 AKAP9
UBC AKAP8L
TMPO AKAP8L

RELA	AKAP8L
MAP3K3	AKAP8L
DHX9	AKAP8L
PRKCA	AKAP5
AKAP5	PRKAR2B
AKAP5	KCNJ2
IQGAP1	AKAP5
AKAP5	ADRB2
UBC	AKAP13
AKAP13	RXRB
AKAP13	GNAQ
AKAP13	GNA12
UBC	AKAP12
PRKAR1A	AKAP11
GSK3B	AKAP11
SLC9A3R1	AKAP10
PRKAR1A	AKAP10
UBC	AKAP1
AKAP1	PRKAR2B
AKAP1	PRKAR1B
PRKAR1A	AKAP1
MYCBP	AKAP1
UGDH	AK5
AK5	NUCKS1
AK5	C7orf26
UBC	AK2
NME2	AK2
UBC	AK1
FHL2	AK1
NUB1	AIPL1
UBC	AIP
PTGES3	AIP
HSP90AB1	AIP
HSP90AA1	AIP
CSNK2A1	AIP
ARNT	AIP
AHR	AIP
UBC	AIMP2
RARS	AIMP2
PARK2	AIMP2
MARS	AIMP2
LNX1	AIMP2
LMO3	AIMP2
IARS	AIMP2

AIMP2	FUBP1
FHL3	AIMP2
EPS8	AIMP2
AIMP2	AIMP1
UBC	AIMP1
RARS	AIMP1
MARS	AIMP1
IARS	AIMP1
PYCARD	AIM2
UBC	AIFM2
UBC	AIF1
RPA1	AICDA
KPNA1	AICDA
UBC	AHSA1
STK24	AHSA1
XPO1	AHR
UBC	AHR
TBP	AHR
STUB1	AHR
SP1	AHR
SMARCA4	AHR
RELA	AHR
RB1	AHR
PTGES3	AHR
NRIP1	AHR
AHR	NR2F1
NEDD8	AHR
NCOR2	AHR
NCOA2	AHR
NCOA1	AHR
HSP90AA1	AHR
GTF2F2	AHR
ESR1	AHR
DAP3	AHR
CCNT1	AHR
ARNTL	AHR
ARNT	AHR
UBC	AHNAK2
UBC	AHNAK
ESR1	AHNAK
UBC	AHCYL2
UBC	AHCYL1
UBC	AHCY
UBC	AHCTF1

SNCAIP	AGXT
AGTR1	NOS3
UBC	AGTPBP1
REN	AGT
PRG2	AGT
AGT	PRCP
MEP1A	AGT
AGT	MAS1
CTSG	AGT
AGTR1	AGT
AGT	ACE
UBC	AGRN
LYPD3	AGR3
UBC	AGPS
GNPAT	AGPS
UBC	AGPAT9
UBC	AGPAT5
UBC	AGPAT3
UBC	AGPAT2
UBC	AGO2
AGO2	TNRC6C
AGO2	TNRC6B
AGO2	TARBP2
AGO2	LRRK2
HSP90AA1	AGO2
AGO2	DICER1
AGO2	DDX20
DCP1A	AGO2
AGO1	AGO2
UBC	AGO1
UBC	AGL
S100B	AGER
S100A1	AGER
UBC	AGAP3
HOMER1	AGAP2
EPB41L1	AGAP2
SGSM2	AFP
CCR5	AFP
UBC	AFG3L2
TNFRSF1A	AFG3L2
UBC	AFF4
TRAF2	AFF4
MLLT1	AFF4
AFF1	AFF4

PRKCE	AFAP1
PRKCB	AFAP1
AESSIX1	
PTRH2	AES
RALYL	AEBP2
EED	AEBP2
UBC	ADSS
UBC	ADSL
UCHL5	ADRM1
UBQLN1	ADRM1
UBC	ADRM1
RAD23B	ADRM1
RAD23A	ADRM1
PSMD7	ADRM1
PSMD4	ADRM1
PSMD2	ADRM1
PSMD14	ADRM1
PSMD1	ADRM1
PSMA6	ADRM1
UBC	ADRBK2
CXCR4	ADRBK2
UBC	ADRBK1
RHO	ADRBK1
ADRBK1	RCVRN
ADRBK1	PIK3CG
MDM2	ADRBK1
GNAQ	ADRBK1
GNAS	ADRB3
UBC	ADRB2
SLC9A3R2	ADRB2
SLC9A3R1	ADRB2
PRKCA	ADRB2
OPRD1	ADRB2
NEDD4	ADRB2
KCNJ9	ADRB2
ADRB2	KCNJ6
IL1B	ADRB2
GRK6	ADRB2
GRB2	ADRB2
GNB1	ADRB2
GNAS	ADRB2
GNA15	ADRB2
GNA14	ADRB2
GNA11	ADRB2

EIF2B1	ADRB2
ARRDC3	ADRB2
AKAP12	ADRB2
ADRBK1	ADRB2
MAGI2	ADRB1
GOPC	ADRB1
GIPC1	ADRB1
ARRB1	ADRB1
EIF2B1	ADRA2C
GNAI2	ADRA2B
EIF2B1	ADRA2B
ADRA1B	SAG
GNAI2	ADORA1
EPB41L2	ADORA1
ADORA1	DRD1
ADA	ADORA1
UBC	ADNP2
UBC	ADNP
RAMP2	ADM
NPPC	ADM
GPR182	ADM
EDN1	ADM
UBC	ADK
ADIPOR2	ADIPOQ
UBC	ADH5
UBC	ADD3
ADD2	RPH3A
ADD1	ADD2
CASP2	ADCYAP1
ADCYAP1R1	ADCYAP1
UBC	ADCY9
UBC	ADCY7
UBC	ADCY6
PRKCA	ADCY5
UBC	ADCY3
UBC	ADCY2
ADCY5	ADCY2
ADCY1	ADCY2
ADCY10	PTGIR
UBC	ADCK4
UBC	ADCK3
UBC	ADAT1
UBC	ADAR
EEF1G	ADAP2

SUB1	ADAP1
SRSF2	ADAP1
RPS9	ADAP1
PRKD1	ADAP1
PRKCA	ADAP1
NCL	ADAP1
CSNK1A1	ADAP1
ADAP1	AP3B2
ADAP1	ANP32A
ADAMTSL4	DLK2
ADAMTSL4	CATSPER1
ACAN	ADAMTS4
VWF	ADAMTS13
VCAN	ADAMTS1
PRKCD	ADAM9
ITGA4	ADAM28
ADAM2	CLGN
FURIN	ADAM19
COPB1	ADAM19
UBC	ADAM17
TNF	ADAM17
TIMP3	ADAM17
NOTCH1	ADAM17
MAD2L1	ADAM17
EPHB2	ADAM17
DLG1	ADAM17
UBC	ADAM15
TRIP13	ADAM15
SRC	ADAM15
SNX9	ADAM15
SH3PXD2A	ADAM15
SH3GL2	ADAM15
PACSIN3	ADAM15
NCK1	ADAM15
MAD2L2	ADAM15
LCK	ADAM15
HCK	ADAM15
GRB2	ADAM15
FYN	ADAM15
UBC	ADAM10
AXL	ADAM10
ADA	DRD1
ADA	DPP4
UBC	ACY1

TTC1	ACY1
TGFBR2	ACVRL1
ACVRL1	TGFB3
ACVR2B	INHBA
ACVR2B	BMP6
UBC	ACVR2A
ACVR2A	MAGI2
ACVR2A	INHBC
ACVR2A	INHBA
GDF9	ACVR2A
ACVR2A	GDF5
ACVR2A	BMP6
ACVRL1	ACVR2A
ACVR2B	ACVR2A
UBC	ACVR1B
ACVR1B	TDGF1
SMURF1	ACVR1B
ACVR1B	INHBB
ACVR1B	INHBA
ACVR1B	FKBP1A
ACVR2B	ACVR1B
ACVR2A	ACVR1B
ACVR1	ACVR1B
ACVR1	USP39
ACVR1	UBE2E3
ACVR1	TTC27
ACVR1	STX8
SMAD5	ACVR1
SMAD1	ACVR1
ACVR1	RHOJ
ACVR1	RHEBL1
ACVR1	RASL12
ACVR1	RASD2
ACVR1	RAP2A
ACVR1	RAB6B
ACVR1	RAB3B
ACVR1	RAB33B
ACVR1	PLEKHB1
ACVR1	NRAS
ACVR1	INHBC
ACVR1	INHBB
ACVR1	INHBA
ACVR1	ILKAP
ACVR1	GDF5

ACVR1	FKBP4
ACVR1	DCAF6
ACVR1	BMP7
ACVR1	ACVR2A
YY1	ACTR8
UCHL5	ACTR8
UBC	ACTR8
RUVBL2	ACTR8
INO80E	ACTR8
INO80	ACTR8
ACTR5	ACTR8
UBC	ACTR6
RUVBL2	ACTR5
ARPC5L	ACTR3B
ARPC4-TTLL3	ACTR3B
ARPC1A	ACTR3B
WASF3	ACTR3
WASF2	ACTR3
UBC	ACTR3
ESR1	ACTR3
CTTN	ACTR3
CDC42	ACTR3
ARPC5L	ACTR3
ARPC5	ACTR3
ARPC4-TTLL3	ACTR3
ARPC3	ACTR3
ARPC2	ACTR3
ARPC1B	ACTR3
ACTR2	ACTR3
WASF2	ACTR2
WASF1	ACTR2
UBC	ACTR2
ESR1	ACTR2
ACTR2	ARPC5
ARPC4-TTLL3	ACTR2
ARPC3	ACTR2
ARPC1B	ACTR2
ACTR2	ACTR3B
ACTC1	ACTR2
UBC	ACTR10
DCTN4	ACTR10
ACTR1A	ACTR10
UBC	ACTN4
SLC9A3R2	ACTN4

ACTN4	NOS3
ACTN4	MYOZ1
ACTN4	MAGI1
ITGB1	ACTN4
ACTN4	COL17A1
ACTN4	ATP6V1B1
ACTN1	ACTN4
UBC	ACTN3
UBC	ACTN2
TTN	ACTN2
SUMO1	ACTN2
SELE	ACTN2
PKN1	ACTN2
PDLIM1	ACTN2
NOS3	ACTN2
MYOZ1	ACTN2
KCNA5	ACTN2
KCNA4	ACTN2
GRIN2B	ACTN2
DISC1	ACTN2
ADAM12	ACTN2
ACTN4	ACTN2
UBC	ACTN1
ACTN1	MYOZ1
ACTN1	MYOT
ACTN1	FBP1
ACTN1	CSRP3
ACTN1	CDK5R2
UBC	ACTL8
TES	ACTL7A
ACTL6A	TRRAP
ACTL6A	SMARCA2
RUVBL2	ACTL6A
MYC	ACTL6A
UBC	ACTG2
ACTG1	DNASE1
VCL	ACTC1
UBC	ACTC1
SUMO1	ACTC1
ACTC1	PRKCE
LASP1	ACTC1
ACTC1	EZR
ESR1	ACTC1
DYNLL1	ACTC1

ACTC1	ANG
ACTC1	ACTN4
ACTC1	ACTB
UBC	ACTBL2
UBC	ACTB
ACTB	SPTBN2
RUVBL2	ACTB
PFN2	ACTB
PFN1	ACTB
DYNLL1	ACTB
ACTB	CPNE4
ACTL6A	ACTB
ACTG1	ACTB
UBC	ACTA2
ACTA2	DNASE1
CCT4	ACTA2
VASP	ACTA1
UBC	ACTA1
ACTA1	TRIM63
ACTA1	PRKCE
OCLN	ACTA1
ACTA1	MAP1A
ACTA1	MACF1
ACTA1	FGD4
EGFR	ACTA1
ACTA1	DNASE1
ACTA1	COTL1
ACTA1	CNN1
ACTA1	CCT4
UBC	ACSS2
UBC	ACSL5
UBC	ACSL3
UBC	ACSL1
GNG7	ACP6
ACP5	TNFSF10
ACP5	SNRNP40
ACP5	PDCD5
ACP5	EGR2
UBC	ACP2
UBC	ACP1
UBC	ACOX3
SMURF2	ACOX3
PEX5	ACOX1
PEX5	ACOT8

UBC	ACOT7
UBC	ACOT2
UBC	ACOT1
UBC	ACO2
UBC	ACO1
UBC	ACLY
ACIN1	BTBD10
LAMA1	ACHE
COLQ	ACHE
APP	ACHE
UBC	ACE
BDKRB2	ACE
UBC	ACBD6
UBC	ACBD5
UBC	ACBD3
SMURF2	ACBD3
UBC	ACAT2
UBC	ACAT1
EIF1B	ACAT1
IFFO1	ACAP1
UBC	ACADVL
UBC	ACADSB
UBC	ACACB
PRKAA2	ACACB
UBC	ACACA
PRKAA1	ACACA
SMAD9	ABTB1
SMAD2	ABTB1
UBC	ABT1
KRT15	ABLIM1
ERBB4	ABL2
EPHB2	ABL2
ABL1	ZAP70
ABL1	WASF1
UBC	ABL1
ABL1	TUB
ABL1	TP73
TP53	ABL1
ABL1	ST5
ABL1	SOS2
ABL1	SORBS2
ABL1	SORBS1
ABL1	SH3BP1
ABL1	ROS1

ABL1	ROBO1
ABL1	RIN1
ABL1	RAD51
PXN	ABL1
PTPN6	ABL1
ABL1	PTPN12
ABL1	PSTPIP1
ABL1	PLCG1
ABL1	PIK3R1
ABL1	PARK2
ABL1	PAK2
ABL1	NTRK1
ABL1	NCSTN
ABL1	JAK2
ABL1	JAK1
ABL1	INPPL1
ABL1	HSP90AA1
ABL1	HCK
ABL1	GRIN2D
ABL1	GRB2
ABL1	ERBB4
ERBB3	ABL1
ABL1	EPHB2
EGFR	ABL1
ABL1	DOK1
CRKL	ABL1
CRK	ABL1
ABL1	CAV1
ABL1	BCAR1
ABL1	ATM
ABL1	ABL2
ABL1	ABI2
ABL1	ABI1
ABI1	WASF2
UBC	ABI1
TCERG1	ABI1
NIN	ABI1
NEB	ABI1
HSPA8	ABI1
GAS7	ABI1
ENAH	ABI1
CYFIP1	ABI1
CBLB	ABI1
UBC	ABHD6

UBC	ABHD5
PLIN1	ABHD5
UBC	ABHD4
UBC	ABHD3
UBC	ABHD2
UBC	ABHD17C
UBC	ABHD17B
UBC	ABHD16A
ABHD16A	RNF5
ABHD16A	HM13
ABHD16A	ATP5G3
UBC	ABHD11
UBC	ABHD10
ABCG5	ABCG8
UBC	ABCG2
UBC	ABCF3
SPP1	ABCF3
ACIN1	ABCF3
UBC	ABCF2
UBC	ABCD4
UBC	ABCD2
ABCD3	ABCD2
KCNJ8	ABCC9
KCNJ8	ABCC8
KCNJ11	ABCC8
CRYBB1	ABCC8
UBC	ABCC5
UBC	ABCC4
UBC	ABCC3
UBC	ABCC2
PDZK1	ABCC2
UBC	ABCC10
UBC	ABCC1
UBC	ABCB8
UBC	ABCB6
UBC	ABCB10
UBC	ABCB1
UBC	ABCA3
ALDH5A1	ABAT
UBC	AATF
SUMO2	AATF
SP1	AATF
RB1	AATF
POLR2J	AATF

MPHOSPH6	AATF
MAPT	AATF
DAZAP2	AATF
UBC	AASS
PXN	AASS
UBC	AASDHPPT
UBC	AARS2
UBC	AAR2
EAPP	AAR2
UBC	AAGAB
UBC	AACS

Supplementary Table S2. Detailed information about module genes and sigMHC-genes

Category	GeneSymbol	Chr	Start	Stop	Pvalue	FDR	GeneName
module-gene	**STAT4**	2	191894302	192016322	1E-07	0.000336613	signal transducer and activator of transcription 4
module-gene	**GTF2I**	7	74071994	74175026	2.3E-06	0.006120227	general transcription factor IIi
module-gene	ZNF208	19	22115760	22193751	6.2E-06	0.010434988	zinc finger protein 208
module-gene	PTTG1	5	159848829	159855748	0.00143	0.583461667	pituitary tumor-transforming 1
module-gene	ERBB4	2	212240446	213403565	0.00241	0.638602	v-erb-a erythroblastic leukemia viral oncogene homolog 4 (avian)
module-gene	STAT1	2	191829084	191885686	0.00267	0.65399	signal transducer and activator of transcription 1, 91kDa
module-gene	ARID3A	19	925781	975939	0.00282	0.65399	AT rich interactive domain 3A (BRIGHT-like)
module-gene	**NFKB1**	4	103422486	103538459	0.0046	0.664721105	nuclear factor of kappa light polypeptide gene enhancer in B-cells 1
module-gene	VTA1	6	142468367	142542085	0.00495	0.667196119	Vps20-associated 1 homolog (S. cerevisiae)
module-gene	CFLAR	2	201980827	202029033	0.00594	0.690401934	CASP8 and FADD-like apoptosis regulator
module-gene	EED	11	85955586	85989855	0.00735	0.730362177	embryonic ectoderm development
module-gene	TRIM24	7	138145079	138274738	0.008	0.732719302	tripartite motif containing 24
module-gene	NFKBIE	6	44225903	44233500	0.00801	0.732719302	nuclear factor of kappa light polypeptide gene enhancer in B-cells inhibitor, epsilon
module-gene	WWOX	16	78133310	79246564	0.00839	0.734656781	WW domain containing oxidoreductase
module-gene	RPA1	17	1732996	1803376	0.00839	0.734656781	replication protein A1, 70kDa
module-gene	FXR2	17	7494548	7518189	0.0086	0.734656781	fragile X mental retardation, autosomal homolog 2
module-gene	PSMD7	16	74330673	74340186	0.0087	0.734656781	proteasome (prosome, macropain) 26S subunit, non-ATPase, 7
module-gene	PSMA1	11	14515329	14665181	0.01169	0.778329444	proteasome (prosome, macropain) subunit, alpha type, 1
module-gene	HSF1	8	145515280	145538385	0.01244	0.778329444	heat shock transcription factor 1
module-gene	CBS	21	44473301	44497053	0.01323	0.778329444	cystathionine-beta-synthase
module-gene	NIF3L1	2	201754050	201768655	0.01471	0.778329444	NIF3 NGG1 interacting factor 3-like 1 (S. cerevisiae)
module-gene	MBL2	10	54525140	54531460	0.01471	0.778329444	mannose-binding lectin (protein C) 2, soluble
module-gene	HR	8	21971928	21990897	0.02041	0.800454768	hairless homolog (mouse)
module-gene	TSC22D4	7	100060982	100076902	0.0283	0.821427542	TSC22 domain family, member 4
module-gene	SUMO2	17	73163408	73179078	0.06652	0.847394439	SMT3 suppressor of mif two 3 homolog 2 (S. cerevisiae)
module-gene	CCDC90B	11	82970139	82997450	0.107	0.892321893	coiled-coil domain containing 90B

module-gene	TP53	17	7565097	7590856	0.12	0.89489892	tumor protein p53
module-gene	UHRF2	9	6413151	6507054	0.132	0.897825176	ubiquitin-like with PHD and ring finger domains 2, E3 ubiquitin protein ligase
module-gene	DMRTB1	1	53925072	53933161	0.214	0.918298273	DMRT-like family B with proline-rich C-terminal, 1
module-gene	HDAC1	1	32757687	32799236	0.246	0.921030479	histone deacetylase 1
module-gene	LAMA4	6	112429963	112576141	0.253	0.922550711	laminin, alpha 4
module-gene	MAD2L1	4	120980577	120988229	0.28	0.92961657	MAD2 mitotic arrest deficient-like 1 (yeast)
module-gene	PRMT2	21	48055079	48085036	0.372	0.947501852	protein arginine methyltransferase 2
module-gene	PAFAH1B3	19	42801185	42807698	0.451	0.953145425	platelet-activating factor acetylhydrolase 1b, catalytic subunit 3 (29kDa)
module-gene	UBC	12	125396150	125401914	0.463	0.953692688	ubiquitin C
module-gene	ERH	14	69846848	69865344	0.525	0.962250781	enhancer of rudimentary homolog (Drosophila)
module-gene	PCNP	3	101292939	101313281	0.713	0.975889905	PEST proteolytic signal containing nuclear protein
module-gene	CDKN2C	1	51426417	51440305	0.885	0.988281584	cyclin-dependent kinase inhibitor 2C (p18, inhibits CDK4)
module-gene	JUN	1	59246465	59249785	0.918	0.991052838	jun proto-oncogene
module-gene	NMI	2	152126979	152146571	0.924	0.991540578	N-myc (and STAT) interactor
sigMHC-genes	HLA-DPA1	6	33032346	33048552	0	0	major histocompatibility complex, class II, DP alpha 1
sigMHC-genes	**HLA-DPB1**	6	33043703	33054978	0	0	major histocompatibility complex, class II, DP beta 1
sigMHC-genes	HLA-DRA	6	32407619	32412823	2.5E-06	0.006120227	major histocompatibility complex, class II, DR alpha
sigMHC-genes	HLA-DQB1	6	32627244	32636160	2.9E-06	0.006507842	major histocompatibility complex, class II, DQ beta 1
sigMHC-genes	RXRB	6	33161365	33168630	9.56E-05	0.117875577	retinoid X receptor, beta
sigMHC-genes	SLC39A7	6	33168222	33172216	9.63E-05	0.117875577	solute carrier family 39 (zinc transporter), member 7
sigMHC-genes	RING1	6	33176272	33180499	0.001095	0.524406842	ring finger protein 1
sigMHC-genes	HLA-DMB	6	32902406	32908847	0.001143	0.530454871	major histocompatibility complex, class II, DM beta
sigMHC-genes	CCHCR1	6	31110216	31126015	0.0013	0.555677778	coiled-coil alpha-helical rod protein 1
sigMHC-genes	TAP2	6	32789610	32806557	0.00194	0.638602	transporter 2, ATP-binding cassette, sub-family B (MDR/TAP)
sigMHC-genes	PSMB8	6	32808494	32812480	0.00391	0.664721105	proteasome (prosome, macropain) subunit, beta type, 8 (large multifunctional peptidase 7)
sigMHC-genes	HLA-DMA	6	32916390	32936871	0.00453	0.664721105	major histocompatibility complex, class II, DM alpha
sigMHC-genes	PSMB9	6	32811913	32827362	0.0046	0.664721105	proteasome (prosome, macropain) subunit, beta type, 9 (large multifunctional peptidase 2)

sigMHC-genes	TAP1	6	32812986	32821755	0.00493	0.667196119	transporter 1, ATP-binding cassette, sub-family B (MDR/TAP)
sigMHC-genes	HLA-DQA2	6	32709119	32714992	0.00876	0.73488486	major histocompatibility complex, class II, DQ alpha 2
sigMHC-genes	CSNK2B	6	31633013	31638120	0.01315	0.778329444	casein kinase 2, beta polypeptide
sigMHC-genes	HIST1H1B	6	27834570	27835359	0.01786	0.778329444	histone cluster 1, H1b
sigMHC-genes	**HLA-DRB1**	6	32546546	32557625	0.01952	0.78967473	major histocompatibility complex, class II, DR beta 1
sigMHC-genes	TRIM31	6	30070674	30080883	0.01975	0.793802612	tripartite motif containing 31
sigMHC-genes	AGER	6	32148745	32152101	0.02094	0.800502285	advanced glycosylation end product-specific receptor
sigMHC-genes	RNF5	6	32146131	32151930	0.02111	0.800502285	ring finger protein 5, E3 ubiquitin protein ligase
sigMHC-genes	ABHD16A	6	31654726	31671221	0.02235	0.806077124	abhydrolase domain containing 16A
sigMHC-genes	ZNF165	6	28048753	28057341	0.02433	0.818974742	zinc finger protein 165
sigMHC-genes	NOTCH4	6	32162620	32191844	0.02451	0.818974742	notch 4
sigMHC-genes	TRIM26	6	30152232	30181204	0.02473	0.818974742	tripartite motif containing 26
sigMHC-genes	PBX2	6	32151966	32157963	0.02576	0.818974742	pre-B-cell leukemia homeobox 2
sigMHC-genes	UBD	6	29523292	29527702	0.03807	0.836239287	ubiquitin D
sigMHC-genes	APOM	6	31620193	31625987	0.04097	0.847394439	apolipoprotein M
sigMHC-genes	GPANK1	6	31629006	31634060	0.04135	0.847394439	G patch domain and ankyrin repeats 1
sigMHC-genes	POU5F1	6	31130253	31148508	0.04216	0.847394439	POU class 5 homeobox 1
sigMHC-genes	HLA-F	6	29690552	29706305	0.0491	0.847394439	major histocompatibility complex, class I, F

Supplementary Table 3. GO terms enriched by module genes.

GO Terms		No. of genes[a]	*P*-value[b]	P_{BH}-value[c]
	biological process			
GO:0010605	negative regulation of macromolecule metabolic process	11	1.90E-05	0.028534
GO:0007049	cell cycle	11	3.07E-05	0.046132
GO:0032269	negative regulation of cellular protein metabolic process	6	1.22E-04	0.1826
GO:0006351	transcription, DNA-dependent	7	1.27E-04	0.190651
GO:0032774	RNA biosynthetic process	7	1.37E-04	0.205367
GO:0051248	negative regulation of protein metabolic process	6	1.46E-04	0.218367
GO:0043161	proteasomal ubiquitin-dependent protein catabolic process	5	1.66E-04	0.249149
GO:0010498	proteasomal protein catabolic process	5	1.66E-04	0.249149
	molecular function			
GO:0030528	transcription regulator activity	17	1.78E-07	2.04E-04
GO:0003700	transcription factor activity	13	2.43E-06	0.002784
GO:0003677	DNA binding	17	5.83E-05	0.066784
	cellular component			
GO:0031981	nuclear lumen	12	2.36E-05	0.026507
GO:0043232	intracellular non-membrane-bounded organelle	15	4.84E-05	0.054357
GO:0043228	non-membrane-bounded organelle	15	4.84E-05	0.054357
GO:0044427	chromosomal part	7	6.19E-05	0.069525
GO:0070013	intracellular organelle lumen	12	1.60E-04	0.1796
GO:0005694	chromosome	7	1.63E-04	0.18288
GO:0043233	organelle lumen	12	1.97E-04	0.221109

[a] Number of genes in the 40 module genes and also in the category.

[b] *P*-value were calculated by hypergeometric test.

[c] P_{BH}-value were adjusted by Benjamini & Hochberg (BH) method. Only GO terms with P_{BH}-value < 0.25 were shown.
